# Supplementary material for: Intrinsic Disorder as a Natural Preservative: High Levels of Intrinsic Disorder in Proteins Found in the 2600-Year-Old Human Brain
Source: Biology (Basel). 2022 Nov 25;11(12):1704. doi: 10.3390/biology11121704 (PMC9775155; doi:10.3390/biology11121704)
Supplement: Supplementary file 1 [file biology-11-01704-s001.zip › biology-1969384-supplementary.pdf]

Supplementary Materials

Intrinsic disorder as a natural preservative: High levels of in-trinsic disorder in proteins found in the 2,600-year-old human brain

Aaron Mohammed [1] and Vladimir N. Uversky [1,2,*]

Figure S1. Amino acid sequences of the Heslington brain proteins analyzed in this study

>sp|P35527|K1C9_HUMAN Keratin, type I cytoskeletal 9 OS=Homo sapiens OX=9606 GN=KRT9 PE=1 SV=3
MSCRQFSSSYLSRSGGGGGGGLGSGGSIRSSYSRFSSSGGGGGGGRFSSSSGYGGGSSRV
CGRGGGGSFGYSYGGGSGGGFSASSLGGGFGGGSRGFGGASGGGYSSSGGFGGGFGGGSG
GGFGGGYGSGFGGFGGFGGGAGGGDGGILTANEKSTMQELNSRLASYLDKVQALEEANND
LENKIQDWYDKKGPAAIQKNYSPYYNTIDDLKDQIVDLTVGNNKTLLDIDNTRMTLDDFR
IKFEMEQNLRQGVDADINGLRQVLDNLTMEKSDLEMQYETLQEELMALKKNHKEEMSQLT
GQNSGDVNVEINVAPGKDLTKTLNDMRQEYEQLIAKNRKDIENQYETQITQIEHEVSSSG
QEVQSSAKEVTQLRHGVQELEIELQSQLSKKAALEKSLEDTKNRYCGQLQMIQEQISNLE
AQITDVRQEIECQNQEYSLLLSIKMRLEKEIETYHNLLEGGQEDFESSGAGKIGLGGRGG
SGGSYGRGSRGGSGGSYGGGGSGGGYGGGSGSRGGSGGSYGGGSGSGGGSGGGYGGGSGG
GHSGGSGGGHSGGSGGNYGGGSGSGGGSGGGYGGGSGSRGGSGGSHGGGSGFGGESGGSY
GGGEEASGSGGGYGGGSGKSSHS
>sp|P15924|DESP_HUMAN Desmoplakin OS=Homo sapiens OX=9606 GN=DSP PE=1 SV=3
MSCNGGSHPRINTLGRMIRAESGPDLRYEVTSGGGGTSRMYYSRRGVITDQNSDGYCQTG
TMSRHQNQNTIQELLQNCSDCLMRAELIVQPELKYGDGIQLTRSRELDECFAQANDQMEI
LDSLIREMRQMGQPCDAYQKRLLQLQEQMRALYKAISVPRVRRASSKGGGGYTCQSGSGW
DEFTKHVTSECLGWMRQQRAEMDMVAWGVDLASVEQHINSHRGIHNSIGDYRWQLDKIKA
DLREKSAIYQLEEEYENLLKASFERMDHLRQLQNIIQATSREIMWINDCEEEELLYDWSD
KNTNIAQKQEAFSIRMSQLEVKEKELNKLKQESDQLVLNQHPASDKIEAYMDTLQTQWSW
ILQITKCIDVHLKENAAYFQFFEEAQSTEAYLKGLQDSIRKKYPCDKNMPLQHLLEQIKE
LEKEREKILEYKRQVQNLVNKSKKIVQLKPRNPDYRSNKPIILRALCDYKQDQKIVHKGD
ECILKDNNERSKWYVTGPGGVDMLVPSVGLIIPPPNPLAVDLSCKIEQYYEAILALWNQL
YINMKSLVSWHYCMIDIEKIRAMTIAKLKTMRQEDYMKTIADLELHYQEFIRNSQGSEMF
GDDDKRKIQSQFTDAQKHYQTLVIQLPGYPQHQTVTTTEITHHGTCQDVNHNKVIETNRE
NDKQETWMLMELQKIRRQIEHCEGRMTLKNLPLADQGSSHHITVKINELKSVQNDSQAIA
EVLNQLKDMLANFRGSEKYCYLQNEVFGLFQKLENINGVTDGYLNSLCTVRALLQAILQT
EDMLKVYEARLTEEETVCLDLDKVEAYRCGLKKIKNDLNLKKSLLATMKTELQKAQQIHS
QTSQQYPLYDLDLGKFGEKVTQLTDRWQRIDKQIDFRLWDLEKQIKQLRNYRDNYQAFCK
WLYDAKRRQDSLESMKFGDSNTVMRFLNEQKNLHSEISGKRDKSEEVQKIAELCANSIKD
YELQLASYTSGLETLLNIPIKRTMIQSPSGVILQEAADVHARYIELLTRSGDYYRFLSEM
LKSLEDLKLKNTKIEVLEEELRLARDANSENCNKNKFLDQNLQKYQAECSQFKAKLASLE
ELKRQAELDGKSAKQNLDKCYGQIKELNEKITRLTYEIEDEKRRRKSVEDRFDQQKNDYD
QLQKARQCEKENLGWQKLESEKAIKEKEYEIERLRVLLQEEGTRKREYENELAKVRNHYN
EEMSNLRNKYETEINITKTTIKEISMQKEDDSKNLRNQLDRLSRENRDLKDEIVRLNDSI
LQATEQRRRAEENALQQKACGSEIMQKKQHLEIELKQVMQQRSEDNARHKQSLEEAAKTI
QDKNKEIERLKAEFQEEAKRRWEYENELSKVRNNYDEEIISLKNQFETEINITKTTIHQL
TMQKEEDTSGYRAQIDNLTRENRSLSEEIKRLKNTLTQTTENLRRVEEDIQQQKATGSEV
SQRKQQLEVELRQVTQMRTEESVRYKQSLDDAAKTIQDKNKEIERLKQLIDKETNDRKCL
EDENARLQRVQYDLQKANSSATETINKLKVQEQELTRLRIDYERVSQERTVKDQDITRFQ
NSLKELQLQKQKVEEELNRLKRTASEDSCKRKKLEEELEGMRRSLKEQAIKITNLTQQLE
QASIVKKRSEDDLRQQRDVLDGHLREKQRTQEELRRLSSEVEALRRQLLQEQESVKQAHL
RNEHFQKAIEDKSRSLNESKIEIERLQSLTENLTKEHLMLEEELRNLRLEYDDLRRGRSE
ADSDKNATILELRSQLQISNNRTLELQGLINDLQRERENLRQEIEKFQKQALEASNRIQE

SKNQCTQVVQERESLLVKIKVLEQDKARLQRLEDELNRAKSTLEAETRVKQRLECEKQQI
QNDLNQWKTQYSRKEEAIRKIESEREKSEREKNSLRSEIERLQAEIKRIEERCRRKLEDS
TRETQSQLETERSRYQREIDKLRQRPYGSHRETQTECEWTVDTSKLVFDGLRKKVTAMQL
YECQLIDKTTLDKLLKGKKSVEEVASEIQPFLRGAGSIAGASASPKEKYSLVEAKRKKLI
SPESTVMLLEAQAATGGIIDPHRNEKLTVDSAIARDLIDFDDRQQIYAAEKAITGFDDPF
SGKTVSVSEAIKKNLIDRETGMRLLEAQIASGGVVDPVNSVFLPKDVALARGLIDRDLYR
SLNDPRDSQKNFVDPVTKKKVSYVQLKERCRIEPHTGLLLLSVQKRSMSFQGIRQPVTVT
ELVDSGILRPSTVNELESGQISYDEVGERIKDFLQGSSCIAGIYNETTKQKLGIYEAMKI
GLVRPGTALELLEAQAATGFIVDPVSNLRLPVEEAYKRGLVGIEFKEKLLSAERAVTGYN
DPETGNIISLFQAMNKELIEKGHGIRLLEAQIATGGIIDPKESHRLPVDIAYKRGYFNEE
LSEILSDPSDDTKGFFDPNTEENLTYLQLKERCIKDEETGLCLLPLKEKKKQVQTSQKNT
LRKRRVVIVDPETNKEMSVQEAYKKGLIDYETFKELCEQECEWEEITITGSDGSTRVVLV
DRKTGSQYDIQDAIDKGLVDRKFFDQYRSGSLSLTQFADMISLKNGVGTSSSMGSGVSDD
VFSSSRHESVSKISTISSVRNLTIRSSSFSDTLEESSPIAAIFDTENLEKISITEGIERG
IVDSITGQRLLEAQACTGGIIHPTTGQKLSLQDAVSQGVIDQDMATRLKPAQKAFIGFEG
VKGKKKMSAAEAVKEKWLPYEAGQRFLEFQYLTGGLVDPEVHGRISTEEAIRKGFIDGRA
AQRLQDTSSYAKILTCPKTKLKISYKDAINRSMVEDITGLRLLEAASVSSKGLPSPYNMS
SAPGSRSGSRSGSRSGSRSGSRSGSRRGSFDATGNSSYSYSYSYSFSSSSIGH
>sp|Q9UPN3|MACF1_HUMAN Microtubule-actin cross-linking factor 1, isoforms 1/2/3/4/5 OS=Homo sapiens OX=9606 GN=MACF1 PE=1 SV=4
MSSSDEETLSERSCRSERSCRSERSYRSERSGSLSPCPPGDTLPWNLPLHEQKKRKSQDS
VLDPAERAVVRVADERDRVQKKTFTKWVNKHLMKVRKHINDLYEDLRDGHNLISLLEVLS
GIKLPREKGRMRFHRLQNVQIALDFLKQRQVKLVNIRNDDITDGNPKLTLGLIWTIILHF
QISDIYISGESGDMSAKEKLLLWTQKVTAGYTGIKCTNFSSCWSDGKMFNALIHRYRPDL
VDMERVQIQSNRENLEQAFEVAERLGVTRLLDAEDVDVPSPDEKSVITYVSSIYDAFPKV
PEGGEGISATEVDSRWQEYQSRVDSLIPWIKQHTILMSDKTFPQNPVELKALYNQYIHFK
ETEILAKEREKGRIEELYKLLEVWIEFGRIKLPQGYHPNDVEEEWGKLIIEMLEREKSLR
PAVERLELLLQIANKIQNGALNCEEKLTLAKNTLQADAAHLESGQPVQCESDVIMYIQEC
EGLIRQLQVDLQILRDENYYQLEELAFRVMRLQDELVTLRLECTNLYRKGHFTSLELVPP
STLTTTHLKAEPLTKATHSSSTSWFRKPMTRAELVAISSSEDEGNLRFVYELLSWVEEMQ
MKLERAEWGNDLPSVELQLETQQHIHTSVEELGSSVKEARLYEGKMSQNFHTSYAETLGK
LETQYCKLKETSSFRMRHLQSLHKFVSRATAELIWLNEKEEEELAYDWSDNNSNISAKRN
YFSELTMELEEKQDVFRSLQDTAELLSLENHPAKQTVEAYSAAVQSQLQWMKQLCLCVEQ
HVKENTAYFQFFSDARELESFLRNLQDSIKRKYSCDHNTSLSRLEDLLQDSMDEKEQLIQ
SKSSVASLVGRSKTIVQLKPRSPDHVLKNTISVKAVCDYRQIEITICKNDECVLEDNSQR
TKWKVISPTGNEAMVPSVCFLIPPPNKDAIEMASRVEQSYQKVMALWHQLHVNTKSLISW
NYLRKDLDLVQTWNLEKLRSSAPGECHQIMKNLQAHYEDFLQDSRDSVLFSVADRLRLEE
EVEACKARFQHLMKSMENEDKEETVAKMYISELKNIRLRLEEYEQRVVKRIQSLASSRTD
RDAWQDNALRIAEQEHTQEDLQQLRSDLDAVSMKCDSFLHQSPSSSSVPTLRSELNLLVE
KMDHVYGLSTVYLNKLKTVDVIVRSIQDAELLVKGYEIKLSQEEVVLADLSALEAHWSTL
RHWLSDVKDKNSVFSVLDEEIAKAKVVAEQMSRLTPERNLDLERYQEKGSQLQERWHRVI
AQLEIRQSELESIQEVLGDYRACHGTLIKWIEETTAQQEMMKPGQAEDSRVLSEQLSQQT
ALFAEIERNQTKLDQCQKFSQQYSTIVKDYELQLMTYKAFVESQQKSPGKRRRMLSSSDA
ITQEFMDLRTRYTALVTLTTQHVKYISDALRRLEEEEKVVEEEKQEHVEKVKELLGWVST
LARNTQGKATSSETKESTDIEKAILEQQVLSEELTTKKEQVSEAIKTSQIFLAKHGHKLS
EKEKKQISEQLNALNKAYHDLCDGSANQLQQLQSQLAHQTEQKECRAVAGVIDLGTVEIF
PIFKAMQKGLLDQDTGLVLLESQVIMSGLIAPETGENLSLEEGIARNLINPQMYQQLREL

QDALALISRLTESRGPLSVVEAIEKRIISETVGLKILEAHLATGGFSLSPSENCINLEEA
FHQGLISAWLHSVLESYLRTSKNLIDPNTAEKIGLLDLMQRCIVHQESGFKLLPVKQLAG
GMVSLKSGRKVSIFRAVQEGLIDRQVTVRLLEAQLFAGGIVDPRTGHRLTVEEAVRHNLI
DQDMACAILIRQLQTGGIIDTVTGQRLTIDEAVSNDLVAAKIALVILESLWSFMGLLWPE
SGEILPITDALEQGIVSTELAHKILSNRQHIKALFLPATTEILSWKKAIESGILDRDLAN
NLKSICIPDVMPHMQLADSAEQNINPGAAVLPCSKSHPKATASQSENLLFQLMTHSYINV
QNGQRLLLLDKELMETLTSRDEYQTSPPKVVEIGHQRQKTPEGLQESANVKISGTFSSGW
TVRLPEFQFSSQNKEYPDREDCTTEKGKKTTVETEDSSVENPEQDLFVEQKERNPNIDAL
KVINKVKLEVQRQLIGTQREDQTAVSVRENASRGHLLTIPPAEAEGVPLVVDKDVFSVET
PKKEHQPLRNTSFTCQNEQAHTLETEYIHDETGGSHIKPQSKKLQVQVKKTLGIKLELKS
ETDGNVHPLDKKEMLKKTFLAKDDHKESQEAQNIAGGSMMMSEKTDEEDSGREIFLSCSH
PLELLEEATLNVLSAQLLDGGIFHEQTGQKLLLNEAISRGIVPSHTAVKLMEKLNMFQGF
FDSQTCESLTTEEVINEGLMDEKLLHNVLMADKAISGVLDPRTQTLCSVKDAVTVGLLDK
ETATRILERQVVTGGIIDLKRGKKVSVTLASTLGLVDVADQPELINLEKASKGRDAEKTV
RERLISLQMETTGLIDPDSKAPLTVVQSIDRGLLEREEAVRLLTKQVVDGGIIHHISGMR
LSVDNAFRHGLIGEDLAEKLKRVENLNIHQIFNPETKENISLPKAIKLDLITSDLKREIQ
EVQAFTGNFVDLISGQRLTLAEAKKEGLLTNEAVLSPGMMHGIVDPENCRIVPYSELVKK
CKIDIESGQRYLEVIPFSDIKDGVSDKVLTLSQAIQLGKVDFASTLKVLEAQANTGGIID
TATGKRLTLASALEEKLVDENMVRIIASHQVLNGGIVDIFSDQRVTLVEAIEKRLISPEL
ANMIQIDSSEFSDHRAQIEKQEGIEVCALQNEFLGKDMLIACNQTAEMSCNKVEESERLF
QVENQSAQEKVKVRVSDGEQAKKSREISLKEFGCKDQRKPRMSSDAKEFISIINPHNLKG
KSLGQVSLTHPYSECDFKLKEVARNNMGNDTNEEQEKAVTKIEIISHMKQSTSCLDSEEI
RENQGEVILEVQETYCETSGKLPSEQVLQQPMNARVKSKREKREVIVEESIRTCKPAFLS
EEKLYQETAIRDEHDSHIKSQPREMTSSEKGKEADTEMGFSITFKIEESSSQVVPQGISV
KHLDALTLFSSKQANEGKVNNLSLCLTLKPEENLSREIACGAQSEPFPCMTPRPEGLHYQ
ESDGKAQVTGPSQISKTDKSFQGTTRQETNYQDSWVTSKTKETKHQISSSNECKEKSYQE
VSFDPARGLKLEEITVSRPDSKEVRYLEFSDRKDLHHQGSKSDDKLCGTLKSEIATQELT
GEKFLEMANPNVAGLEAGSIEDIVTQRGSRVLGSFLPEKLFKGVSQKENTGQQNAIISPT
VLETSEEKTVSLTVCSAVKTEKTPQEKLRESPGSEQTPFMTAPEGKGNGGVNPEPFRATQ
NVFTRQLCLEHDEKLVSYLSLLRNIEMRTKQIQPLELNLAELQDLLCQAKVLERELKDLT
TLVSQELECVNQIIISQPQEVPAQLLKALEKDAKNLQKSLSSVSDTWNSRLLHFQNAVEI
EKTKVLNQHTQLEGRLQDLRAWVGNKNLILNSKGSNSEIDVDSLNLCLQQYEDLKQPMAE
RKAQLDALAFDIQFFISEHAQDLSPQQNRQMLRLLNELQRSFQDILEQTAAQVDALQGHL
QQMEQEALVKTLQKQQNTCHQQLEDLCSWVGQAERALAGHQGRTTQQDLSALQKNQSDLK
DLQDDIQNRATSFATVVKDIEGFMEENQTKLSPRELTALREKLHQAKEQYEALQEETRVA
QKELEEAVTSALQQETEKSKAAKELAENKKKIDALLDWVTSVGSSGGQLLTNLPGMEQLS
GASLEKGALDTTDGYMGVNQAPEKLDKQCEMMKARHQELLSQQQNFILATQSAQAFLDQH
GHNLTPEEQQMLQQKLGELKEQYSTSLAQSEAELKQVQTLQDELQKFLQDHKEFESWLER
SEKELENMHKGGSSPETLPSLLKRQGSFSEDVISHKGDLRFVTISGQKVLDMENSFKEGK
EPSEIGNLVKDKLKDATERYTALHSKCTRLGSHLNMLLGQYHQFQNSADSLQAWMQACEA
NVEKLLSDTVASDPGVLQEQLATTKQLQEELAEHQVPVEKLQKVARDIMEIEGEPAPDHR
HVQETTDSILSHFQSLSYSLAERSSLLQKAIAQSQSVQESLESLLQSIGEVEQNLEGKQV
SSLSSGVIQEALATNMKLKQDIARQKSSLEATREMVTRFMETADSTTAAVLQGKLAEVSQ
RFEQLCLQQQEKESSLKKLLPQAEMFEHLSGKLQQFMENKSRMLASGNQPDQDITHFFQQ
IQELNLEMEDQQENLDTLEHLVTELSSCGFALDLCQHQDRVQNLRKDFTELQKTVKEREK
DASSCQEQLDEFRKLVRTFQKWLKETEGSIPPTETSMSAKELEKQIEHLKSLLDDWASKG
TLVEEINCKGTSLENLIMEITAPDSQGKTGSILPSVGSSVGSVNGYHTCKDLTEIQCDMS

DVNLKYEKLGGVLHERQESLQAILNRMEEVHKEANSVLQWLESKEEVLKSMDAMSSPTKT
ETVKAQAESNKAFLAELEQNSPKIQKVKEALAGLLVTYPNSQEAENWKKIQEELNSRWER
ATEVTVARQRQLEESASHLACFQAAESQLRPWLMEKELMMGVLGPLSIDPNMLNAQKQQV
QFMLKEFEARRQQHEQLNEAAQGILTGPGDVSLSTSQVQKELQSINQKWVELTDKLNSRS
SQIDQAIVKSTQYQELLQDLSEKVRAVGQRLSVQSAISTQPEAVKQQLEETSEIRSDLEQ
LDHEVKEAQTLCDELSVLIGEQYLKDELKKRLETVALPLQGLEDLAADRINRLQAALAST
QQFQQMFDELRTWLDDKQSQQAKNCPISAKLERLQSQLQENEEFQKSLNQHSGSYEVIVA
EGESLLLSVPPGEEKRTLQNQLVELKNHWEELSKKTADRQSRLKDCMQKAQKYQWHVEDL
VPWIEDCKAKMSELRVTLDPVQLESSLLRSKAMLNEVEKRRSLLEILNSAADILINSSEA
DEDGIRDEKAGINQNMDAVTEELQAKTGSLEEMTQRLREFQESFKNIEKKVEGAKHQLEI
FDALGSQACSNKNLEKLRAQQEVLQALEPQVDYLRNFTQGLVEDAPDGSDASQLLHQAEV
AQQEFLEVKQRVNSGCVMMENKLEGIGQFHCRVREMFSQLADLDDELDGMGAIGRDTDSL
QSQIEDVRLFLNKIHVLKLDIEASEAECRHMLEEEGTLDLLGLKRELEALNKQCGKLTER
GKARQEQLELTLGRVEDFYRKLKGLNDATTAAEEAEALQWVVGTEVEIINQQLADFKMFQ
KEQVDPLQMKLQQVNGLGQGLIQSAGKDCDVQGLEHDMEEINARWNTLNKKVAQRIAQLQ
EALLHCGKFQDALEPLLSWLADTEELIANQKPPSAEYKVVKAQIQEQKLLQRLLDDRKAT
VDMLQAEGGRIAQSAELADREKITGQLESLESRWTELLSKAAARQKQLEDILVLAKQFHE
TAEPISDFLSVTEKKLANSEPVGTQTAKIQQQIIRHKALNEEIVNRKKNVDQAIKNGQAL
LKQTTGEEVLLIQEKLDGIKTRYADITVTSSKALRTLEQARQLATKFQSTYEELTGWLRE
VEEELATSGGQSPTGEQIPQFQQRQKELKKEVMEHRLVLDTVNEVSRALLELVPWRAREG
LDKLVSDANEQYKLVSDTIGQRVDEIDAAIQRSQQYEQAADAELAWVAETKRKLMALGPI
RLEQDQTTAQLQVQKAFSIDIIRHKDSMDELFSHRSEIFGTCGEEQKTVLQEKTESLIQQ
YEAISLLNSERYARLERAQVLVNQFWETYEELSPWIEETRALIAQLPSPAIDHEQLRQQQ
EEMRQLRESIAEHKPHIDKLLKIGPQLKELNPEEGEMVEEKYQKAENMYAQIKEEVRQRA
LALDEAVSQSTQITEFHDKIEPMLETLENLSSRLRMPPLIPAEVDKIRECISDNKSATVE
LEKLQPSFEALKRRGEELIGRSQGADKDLAAKEIQDKLDQMVFFWEDIKARAEEREIKFL
DVLELAEKFWYDMAALLTTIKDTQDIVHDLESPGIDPSIIKQQVEAAETIKEETDGLHEE
LEFIRILGADLIFACGETEKPEVRKSIDEMNNAWENLNKTWKERLEKLEDAMQAAVQYQD
TLQAMFDWLDNTVIKLCTMPPVGTDLNTVKDQLNEMKEFKVEVYQQQIEMEKLNHQGELM
LKKATDETDRDIIREPLTELKHLWENLGEKIAHRQHKLEGALLALGQFQHALEELMSWLT
HTEELLDAQRPISGDPKVIEVELAKHHVLKNDVLAHQATVETVNKAGNELLESSAGDDAS
SLRSRLEAMNQCWESVLQKTEEREQQLQSTLQQAQGFHSEIEDFLLELTRMESQLSASKP
TGGLPETAREQLDTHMELYSQLKAKEETYNQLLDKGRLMLLSRDDSGSGSKTEQSVALLE
QKWHVVSSKMEERKSKLEEALNLATEFQNSLQEFINWLTLAEQSLNIASPPSLILNTVLS
QIEEHKVFANEVNAHRDQIIELDQTGNQLKFLSQKQDVVLIKNLLVSVQSRWEKVVQRSI
ERGRSLDDARKRAKQFHEAWKKLIDWLEDAESHLDSELEISNDPDKIKLQLSKHKEFQKT
LGGKQPVYDTTIRTGRALKEKTLLPEDSQKLDNFLGEVRDKWDTVCGKSVERQHKLEEAL
LFSGQFMDALQALVDWLYKVEPQLAEDQPVHGDLDLVMNLMDAHKVFQKELGKRTGTVQV
LKRSGRELIENSRDDTTWVKGQLQELSTRWDTVCKLSVSKQSRLEQALKQAEVFRDTVHM
LLEWLSEAEQTLRFRGALPDDTEALQSLIDTHKEFMKKVEEKRVDVNSAVAMGEVILAVC
HPDCITTIKHWITIIRARFEEVLTWAKQHQQRLETALSELVANAELLEELLAWIQWAETT
LIQRDQEPIPQNIDRVKALIAEHQTFMEEMTRKQPDVDRVTKTYKRKNIEPTHAPFIEKS
RSGGRKSLSQPTPPPMPILSQSEAKNPRINQLSARWQQVWLLALERQRKLNDALDRLEEL
KEFANFDFDVWRKKYMRWMNHKKSRVMDFFRRIDKDQDGKITRQEFIDGILASKFPTTKL
EMTAVADIFDRDGDGYIDYYEFVAALHPNKDAYRPTTDADKIEDEVTRQVAQCKCAKRFQ
VEQIGENKYRFGDSQQLRLVRILRSTVMVRVGGGWMALDEFLVKNDPCRARGRTNIELRE
KFILPEGASQGMTPFRSRGRRSKPSSRAASPTRSSSSASQSNHSCTSMPSSPATPASGTK

VIPSSGSKLKRPTPTFHSSRTSLAGDTSNSSSPASTGAKTNRADPKKSASRPGSRAGSRA
GSRASSRRGSDASDFDLLETQSACSDTSESSAAGGQGNSRRGLNKPSKIPTMSKKTTTAS
PRTPGPKR
>sp|Q8TDY2|RBCC1_HUMAN RB1-inducible coiled-coil protein 1 OS=Homo sapiens OX=9606 GN=RB1CC1 PE=1 SV=3
MKLYVFLVNTGTTLTFDTELTVQTVADLKHAIQSKYKIAIQHQVLVVNGGECMAADRRVC
TYSAGTDTNPIFLFNKEMILCDRPPAIPKTTFSTENDMEIKVEESLMMPAVFHTVASRTQ
LALEMYEVAKKLCSFCEGLVHDEHLQHQGWAAIMANLEDCSNSYQKLLFKFESIYSNYLQ
SIEDIKLKLTHLGTAVSVMAKIPLLECLTRHSYRECLGRLDSLPEHEDSEKAEMKRSTEL
VLSPDMPRTTNESLLTSFPKSVEHVSPDTADAESGKEIRESCQSTVHQQDETTIDTKDGD
LPFFNVSLLDWINVQDRPNDVESLVRKCFDSMSRLDPRIIRPFIAECRQTIAKLDNQNMK
AIKGLEDRLYALDQMIASCGRLVNEQKELAQGFLANQKRAENLKDASVLPDLCLSHANQL
MIMLQNHRKLLDIKQKCTTAKQELANNLHVRLKWCCFVMLHADQDGEKLQALLRLVIELL
ERVKIVEALSTVPQMYCLAVVEVVRRKMFIKHYREWAGALVKDGKRLYEAEKSKRESFGK
LFRKSFLRNRLFRGLDSWPPSFCTQKPRKFDCELPDISLKDLQFLQSFCPSEVQPFLRVP
LLCDFEPLHQHVLALHNLVKAAQSLDEMSQTITDLLSEQKASVSQTSPQSASSPRMESTA
GITTTTSPRTPPPLTVQDPLCPAVCPLEELSPDSIDAHTFDFETIPHPNIEQTIHQVSLD
LDSLAESPESDFMSAVNEFVIEENLSSPNPISDPQSPEMMVESLYSSVINAIDSRRMQDT
NVCGKEDFGDHTSLNVQLERCRVVAQDSHFSIQTIKEDLCHFRTFVQKEQCDFSNSLKCT
AVEIRNIIEKVKCSLEITLKEKHQKELLSLKNEYEGKLDGLIKETEENENKIKKLKGELV
CLEEVLQNKDNEFALVKHEKEAVICLQNEKDQKLLEMENIMHSQNCEIKELKQSREIVLE
DLKKLHVENDEKLQLLRAELQSLEQSHLKELEDTLQVRHIQEFEKVMTDHRVSLEELKKE
NQQIINQIQESHAEIIQEKEKQLQELKLKVSDLSDTRCKLEVELALKEAETDEIKILLEE
SRAQQKETLKSLLEQETENLRTEISKLNQKIQDNNENYQVGLAELRTLMTIEKDQCISEL
ISRHEEESNILKAELNKVTSLHNQAFEIEKNLKEQIIELQSKLDSELSALERQKDEKITQ
QEEKYEAIIQNLEKDRQKLVSSQEQDREQLIQKLNCEKDEAIQTALKEFKLEREVVEKEL
LEKVKHLENQIAKSPAIDSTRGDSSSLVAELQEKLQEEKAKFLEQLEEQEKRKNEEMQNV
RTSLIAEQQTNFNTVLTREKMRKENIINDLSDKLKSTMQQQERDKDLIESLSEDRARLLE
EKKKLEEEVSKLRSSSFVPSPYVATAPELYGACAPELPGESDRSAVETADEGRVDSAMET
SMMSVQENIHMLSEEKQRIMLLERTLQLKEEENKRLNQRLMSQSMSSVSSRHSEKIAIRD
FQVGDLVLIILDERHDNYVLFTVSPTLYFLHSESLPALDLKPGEGASGASRRPWVLGKVM
EKEYCQAKKAQNRFKVPLGTKFYRVKAVSWNKKV
>sp|P04264|K2C1_HUMAN Keratin, type II cytoskeletal 1 OS=Homo sapiens OX=9606 GN=KRT1 PE=1 SV=6
MSRQFSSRSGYRSGGGFSSGSAGIINYQRRTTSSSTRRSGGGGGRFSSCGGGGGSFGAGG
GFGSRSLVNLGGSKSISISVARGGGRGSGFGGGYGGGGFGGGGFGGGGFGGGGIGGGGFG
GFGSGGGGFGGGGFGGGGYGGGYGPVCPPGGIQEVTINQSLLQPLNVEIDPEIQKVKSRE
REQIKSLNNQFASFIDKVRFLEQQNQVLQTKWELLQQVDTSTRTHNLEPYFESFINNLRR
RVDQLKSDQSRLDSELKNMQDMVEDYRNKYEDEINKRTNAENEFVTIKKDVDGAYMTKVD
LQAKLDNLQQEIDFLTALYQAELSQMQTQISETNVILSMDNNRSLDLDSIIAEVKAQYED
IAQKSKAEAESLYQSKYEELQITAGRHGDSVRNSKIEISELNRVIQRLRSEIDNVKKQIS
NLQQSISDAEQRGENALKDAKNKLNDLEDALQQAKEDLARLLRDYQELMNTKLALDLEIA
TYRTLLEGEESRMSGECAPNVSVSVSTSHTTISGGGSRGGGGGGYGSGGSSYGSGGGSYG
SGGGGGGGRGSYGSGGSSYGSGGGSYGSGGGGGGHGSYGSGSSSGGYRGGSGGGGGGSSG
GRGSGGGSSGGSIGGRGSSSGGVKSSGGSSSVKFVSTTYSGVTR
>sp|P08779|K1C16_HUMAN Keratin, type I cytoskeletal 16 OS=Homo sapiens OX=9606 GN=KRT16 PE=1 SV=4

MTTCSRQFTSSSSMKGSCGIGGGIGGGSSRISSVLAGGSCRAPSTYGGGLSVSSRFSSGG
ACGLGGGYGGGFSSSSSFGSGFGGGYGGGLGAGFGGGLGAGFGGGFAGGDGLLVGSEKVT
MQNLNDRLASYLDKVRALEEANADLEVKIRDWYQRQRPSEIKDYSPYFKTIEDLRNKIIA
ATIENAQPILQIDNARLAADDFRTKYEHELALRQTVEADVNGLRRVLDELTLARTDLEMQ
IEGLKEELAYLRKNHEEEMLALRGQTGGDVNVEMDAAPGVDLSRILNEMRDQYEQMAEKN
RRDAETWFLSKTEELNKEVASNSELVQSSRSEVTELRRVLQGLEIELQSQLSMKASLENS
LEETKGRYCMQLSQIQGLIGSVEEQLAQLRCEMEQQSQEYQILLDVKTRLEQEIATYRRL
LEGEDAHLSSQQASGQSYSSREVFTSSSSSSSRQTRPILKEQSSSSFSQGQSS
>sp|Q86YZ3|HORN_HUMAN Hornerin OS=Homo sapiens OX=9606 GN=HRNR
PE=1 SV=2
MPKLLQGVITVIDVFYQYATQHGEYDTLNKAELKELLENEFHQILKNPNDPDTVDIILQS
LDRDHNKKVDFTEYLLMIFKLVQARNKIIGKDYCQVSGSKLRDDTHQHQEEQEETEKEEN
KRQESSFSHSSWSAGENDSYSRNVRGSLKPGTESISRRLSFQRDFSGQHNSYSGQSSSYG
EQNSDSHQSSGRGQCGSGSGQSPNYGQHGSGSGQSSSNDTHGSGSGQSSGFSQHKSSSGQ
SSGYSQHGSGSGHSSGYGQHGSRSGQSSRGERHRSSSGSSSSYGQHGSGSRQSLGHGRQG
SGSRQSPSHVRHGSGSGHSSSHGQHGSGSSYSYSRGHYESGSGQTSGFGQHESGSGQSSG
YSKHGSGSGHSSSQGQHGSTSGQASSSGQHGSSSRQSSSYGQHESASRHSSGRGQHSSGS
GQSPGHGQRGSGSGQSPSSGQHGTGFGRSSSSGPYVSGSGYSSGFGHHESSSEHSSGYTQ
HGSGSGHSSGHGQHGSRSGQSSRGERQGSSAGSSSSYGQHGSGSRQSLGHSRHGSGSGQS
PSPSRGRHESGSRQSSSYGPHGYGSGRSSSRGPYESGSGHSSGLGHQESRSGQSSGYGQH
GSSSGHSSTHGQHGSTSGQSSSCGQHGATSGQSSSHGQHGSGSSQSSRYGQQGSGSGQSP
SRGRHGSDFGHSSSYGQHGSGSGWSSSNGPHGSVSGQSSGFGHKSGSGQSSGYSQHGSGS
SHSSGYRKHGSRSGQSSRSEQHGSSSGLSSSYGQHGSGSHQSSGHGRQGSGSGHSPSRVR
HGSSSGHSSSHGQHGSGTSCSSSCGHYESGSGQASGFGQHESGSGQGYSQHGSASGHFSS
QGRHGSTSGQSSSSGQHDSSSGQSSSYGQHESASHHASGRGRHGSGSGQSPGHGQRGSGS
GQSPSYGRHGSGSGRSSSSGRHGSGSGQSSGFGHKSSSGQSSGYTQHGSGSGHSSSYEQH
GSRSGQSSRSEQHGSSSGSSSSYGQHGSGSRQSLGHGQHGSGSGQSPSPSRGRHGSGSGQ
SSSYGPYRSGSGWSSSRGPYESGSGHSSGLGHRESRSGQSSGYGQHGSSSGHSSTHGQHG
STSGQSSSCGQHGASSGQSSSHGQHGSGSSQSSGYGRQGSGSGQSPGHGQRGSGSRQSPS
YGRHGSGSGRSSSSGQHGSGLGESSGFGHHESSSGQSSSYSQHGSGSGHSSGYGQHGSRS
GQSSRGERHGSSSGSSSHYGQHGSGSRQSSGHGRQGSGSGHSPSRGRHGSGLGHSSSHGQ
HGSGSGRSSSRGPYESRSGHSSVFGQHESGSGHSSAYSQHGSGSGHFCSQGQHGSTSGQS
STFDQEGSSTGQSSSYGHRGSGSSQSSGYGRHGAGSGQSPSRGRHGSGSGHSSSYGQHGS
GSGWSSSSGRHGSGSGQSSGFGHHESSSWQSSGCTQHGSGSGHSSSYEQHGSRSGQSSRG
ERHGSSSGSSSSYGQHGSGSRQSLGHGQHGSGSGQSPSPSRGRHGSGSGQSSSYSPYGSG
SGWSSSRGPYESGSSHSSGLGHRESRSGQSSGYGQHGSSSGHSSTHGQHGSTSGQSSSCG
QHGASSGQSSSHGQHGSGSSQSSGYGRQGSGSGQSPGHGQRGSGSRQSPSYGRHGSGSGR
SSSSGQHGSGLGESSGFGHHESSSGQSSSYSQHGSGSGHSSGYGQHGSRSGQSSRGERHG
SSSRSSSRYGQHGSGSRQSSGHGRQGSGSGQSPSRGRHGSGLGHSSSHGQHGSGSGRSSS
RGPYESRSGHSSVFGQHESGSGHSSAYSQHGSGSGHFCSQGQHGSTSGQSSTFDQEGSST
GQSSSHGQHGSGSSQSSSYGQQGSGSGQSPSRGRHGSGSGHSSSYGQHGSGSGWSSSSGR
HGSGSGQSSGFGHHESSSWQSSGYTQHGSGSGHSSSYEQHGSRSGQSSRGEQHGSSSGSS
SSYGQHGSGSRQSLGHGQHGSGSGQSPSPSRGRHGSGSGQSSSYGPYGSGSGWSSSRGPY
ESGSGHSSGLGHRESRSGQSSGYGQHGSSSGHSSTHGQHGSASGQSSSCGQHGASSGQSS
SHGQHGSGSSQSSGYGRQGSGSGQSPGHGQRGSGSRQSPSYGRHGSGSGRSSSSGQHGPG
LGESSGFGHHESSSGQSSSYSQHGSGSGHSSGYGQHGSRSGQSSRGERHGSSSGSSSRYG
QHGSGSRQSSGHGRQGSGSGHSPSRGRHGSGSGHSSSHGQHGSGSGRSSSRGPYESRSGH

SSVFGQHESGSGHSSAYSQHGSGSGHFCSQGQHGSTSGQSSTFDQEGSSTGQSSSHGQHG
SGSSQSSSYGQQGSGSGQSPSRGRHGSGSGHSSSYGQHGSGSGWSSSSGRHGSGSGQSSG
FGHHESSSWQSSGYTQHGSGSGHSSSYEQHGSRSGQSSRGERHGSSSGSSSSYGQHGSGS
RQSLGHGQHGSGSGQSPSPSRGRHGSGSGQSSSYSPYGSGSGWSSSRGPYESGSGHSSGL
GHRESRSGQSSGYGQHGSSSGHSSTHGQHGSTSGQSSSCGQHGASSGQSSSHGQHGSGSS
QSSGYGRQGSGSGQSPGHGQRGSGSRQSPSYGRHGSGSGRSSSSGQHGSGLGESSGFGHH
ESSSGQSSSYSQHGSGSGHSSGYGQHGSRSGQSSRGERHGSSSGSSSHYGQHGSGSRQSS
GHGRQGSGSGQSPSRGRHGSGLGHSSSHGQHGSGSGRSSSRGPYESRLGHSSVFGQHESG
SGHSSAYSQHGSGSGHFCSQGQHGSTSGQSSTFDQEGSSTGQSSSYGHRGSGSSQSSGYG
RHGAGSGQSLSHGRHGSGSGQSSSYGQHGSGSGQSSGYSQHGSGSGQDGYSYCKGGSNHD
GGSSGSYFLSFPSSTSPYEYVQEQRCYFYQ
>sp|P02538|K2C6A_HUMAN Keratin, type II cytoskeletal 6A OS=Homo sapiens OX=9606 GN=KRT6A PE=1 SV=3
MASTSTTIRSHSSSRRGFSANSARLPGVSRSGFSSVSVSRSRGSGGLGGACGGAGFGSRS
LYGLGGSKRISIGGGSCAISGGYGSRAGGSYGFGGAGSGFGFGGGAGIGFGLGGGAGLAG
GFGGPGFPVCPPGGIQEVTVNQSLLTPLNLQIDPTIQRVRAEEREQIKTLNNKFASFIDK
VRFLEQQNKVLETKWTLLQEQGTKTVRQNLEPLFEQYINNLRRQLDSIVGERGRLDSELR
GMQDLVEDFKNKYEDEINKRTAAENEFVTLKKDVDAAYMNKVELQAKADTLTDEINFLRA
LYDAELSQMQTHISDTSVVLSMDNNRNLDLDSIIAEVKAQYEEIAQRSRAEAESWYQTKY
EELQVTAGRHGDDLRNTKQEIAEINRMIQRLRSEIDHVKKQCANLQAAIADAEQRGEMAL
KDAKNKLEGLEDALQKAKQDLARLLKEYQELMNVKLALDVEIATYRKLLEGEECRLNGEG
VGQVNISVVQSTVSSGYGGASGVGSGLGLGGGSSYSYGSGLGVGGGFSSSSGRAIGGGLS
SVGGGSSTIKYTTTSSSSRKSYKH
>sp|P04259|K2C6B_HUMAN Keratin, type II cytoskeletal 6B OS=Homo sapiens OX=9606 GN=KRT6B PE=1 SV=5
MASTSTTIRSHSSSRRGFSANSARLPGVSRSGFSSISVSRSRGSGGLGGACGGAGFGSRS
LYGLGGSKRISIGGGSCAISGGYGSRAGGSYGFGGAGSGFGFGGGAGIGFGLGGGAGLAG
GFGGPGFPVCPPGGIQEVTVNQSLLTPLNLQIDPAIQRVRAEEREQIKTLNNKFASFIDK
VRFLEQQNKVLDTKWTLLQEQGTKTVRQNLEPLFEQYINNLRRQLDNIVGERGRLDSELR
NMQDLVEDLKNKYEDEINKRTAAENEFVTLKKDVDAAYMNKVELQAKADTLTDEINFLRA
LYDAELSQMQTHISDTSVVLSMDNNRNLDLDSIIAEVKAQYEEIAQRSRAEAESWYQTKY
EELQITAGRHGDDLRNTKQEIAEINRMIQRLRSEIDHVKKQCANLQAAIADAEQRGEMAL
KDAKNKLEGLEDALQKAKQDLARLLKEYQELMNVKLALDVEIATYRKLLEGEECRLNGEG
VGQVNISVVQSTVSSGYGGASGVGSGLGLGGGSSYSYGSGLGVGGGFSSSSGRATGGGLS
SVGGGSSTIKYTTTSSSSRKSYKH
>sp|P48668|K2C6C_HUMAN Keratin, type II cytoskeletal 6C OS=Homo sapiens OX=9606 GN=KRT6C PE=1 SV=3
MASTSTTIRSHSSSRRGFSANSARLPGVSRSGFSSISVSRSRGSGGLGGACGGAGFGSRS
LYGLGGSKRISIGGGSCAISGGYGSRAGGSYGFGGAGSGFGFGGGAGIGFGLGGGAGLAG
GFGGPGFPVCPPGGIQEVTVNQSLLTPLNLQIDPAIQRVRAEEREQIKTLNNKFASFIDK
VRFLEQQNKVLDTKWTLLQEQGTKTVRQNLEPLFEQYINNLRRQLDSIVGERGRLDSELR
NMQDLVEDLKNKYEDEINKRTAAENEFVTLKKDVDAAYMNKVELQAKADTLTDEINFLRA
LYDAELSQMQTHISDTSVVLSMDNNRNLDLDSIIAEVKAQYEEIAQRSRAEAESWYQTKY
EELQVTAGRHGDDLRNTKQEIAEINRMIQRLRSEIDHVKKQCASLQAAIADAEQRGEMAL
KDAKNKLEGLEDALQKAKQDLARLLKEYQELMNVKLALDVEIATYRKLLEGEECRLNGEG
VGQVNVSVVQSTISSGYGGASGVGSGLGLGGGSSYSYGSGLGIGGGFSSSSGRAIGGGLS
SVGGGSSTIKYTTTSSSSRKSYKH

>sp|P13645|K1C10_HUMAN Keratin, type I cytoskeletal 10 OS=Homo sapiens OX=9606 GN=KRT10 PE=1 SV=6
MSVRYSSSKHYSSSRSGGGGGGGGCGGGGGVSSLRISSSKGSLGGGFSSGGFSGGSFSRG
SSGGGCFGGSSGGYGGLGGFGGGSFRGSYGSSSFGGSYGGIFGGGSFGGGSFGGGSFGGG
GFGGGGFGGGFGGGFGGDGGLLSGNEKVTMQNLNDRLASYLDKVRALEESNYELEGKIKE
WYEKHGNSHQGEPRDYSKYYKTIDDLKNQILNLTTDNANILLQIDNARLAADDFRLKYEN
EVALRQSVEADINGLRRVLDELTLTKADLEMQIESLTEELAYLKKNHEEEMKDLRNVSTG
DVNVEMNAAPGVDLTQLLNNMRSQYEQLAEQNRKDAEAWFNEKSKELTTEIDNNIEQISS
YKSEITELRRNVQALEIELQSQLALKQSLEASLAETEGRYCVQLSQIQAQISALEEQLQQ
IRAETECQNTEYQQLLDIKIRLENEIQTYRSLLEGEGSSGGGGRGGGSFGGGYGGGSSGG
GSSGGGHGGGHGGSSGGGYGGGSSGGGSSGGGYGGGSSSGGHGGSSSGGYGGGSSGGGGG
GYGGGSSGGGSSSGGGYGGGSSSGGHKSSSSGSVGESSSKGPRY
>sp|P35908|K22E_HUMAN Keratin, type II cytoskeletal 2 epidermal OS=Homo sapiens OX=9606 GN=KRT2 PE=1 SV=2
MSCQISCKSRGRGGGGGGFRGFSSGSAVVSGGSRRSTSSFSCLSRHGGGGGGFGGGGFGS
RSLVGLGGTKSISISVAGGGGGFGAAGGFGGRGGGFGGGSSFGGGSGFSGGGFGGGGFGG
GRFGGFGGPGGVGGLGGPGGFGPGGYPGGIHEVSVNQSLLQPLNVKVDPEIQNVKAQERE
QIKTLNNKFASFIDKVRFLEQQNQVLQTKWELLQQMNVGTRPINLEPIFQGYIDSLKRYL
DGLTAERTSQNSELNNMQDLVEDYKKKYEDEINKRTAAENDFVTLKKDVDNAYMIKVELQ
SKVDLLNQEIEFLKVLYDAEISQIHQSVTDTNVILSMDNSRNLDLDSIIAEVKAQYEEIA
QRSKEEAEALYHSKYEELQVTVGRHGDSLKEIKIEISELNRVIQRLQGEIAHVKKQCKNV
QDAIADAEQRGEHALKDARNKLNDLEEALQQAKEDLARLLRDYQELMNVKLALDVEIATY
RKLLEGEECRMSGDLSSNVTVSVTSSTISSNVASKAAFGGSGGRGSSSGGGYSSGSSSYG
SGGRQSGSRGGSGGGGSISGGGYGSGGGSGGRYGSGGGSKGGSISGGGYGSGGGKHSSGG
GSRGGSSSGGGYGSGGGGSSSVKGSSGEAFGSSVTFSFR
>sp|P02533|K1C14_HUMAN Keratin, type I cytoskeletal 14 OS=Homo sapiens OX=9606 GN=KRT14 PE=1 SV=4
MTTCSRQFTSSSSMKGSCGIGGGIGGGSSRISSVLAGGSCRAPSTYGGGLSVSSSRFSSG
GACGLGGGYGGGFSSSSSSFGSGFGGGYGGGLGAGLGGGFGGGFAGGDGLLVGSEKVTMQ
NLNDRLASYLDKVRALEEANADLEVKIRDWYQRQRPAEIKDYSPYFKTIEDLRNKILTAT
VDNANVLLQIDNARLAADDFRTKYETELNLRMSVEADINGLRRVLDELTLARADLEMQIE
SLKEELAYLKKNHEEEMNALRGQVGGDVNVEMDAAPGVDLSRILNEMRDQYEKMAEKNRK
DAEEWFFTKTEELNREVATNSELVQSGKSEISELRRTMQNLEIELQSQLSMKASLENSLE
ETKGRYCMQLAQIQEMIGSVEEQLAQLRCEMEQQNQEYKILLDVKTRLEQEIATYRRLLE
GEDAHLSSSQFSSGSQSSRDVTSSSRQIRTKVMDVHDGKVVSTHEQVLRTKN
>sp|P58107|EPIPL_HUMAN Epiplakin OS=Homo sapiens OX=9606 GN=EPPK1 PE=1 SV=3
MSGHTLPPLPVPGTNSTEQASVPRAMAATLGAGTPPRPQARSIAGVYVEASGQAQSVYAA
MEQGLLPAGLGQALLEAQAATGGLVDLARGQLLPVSKALQQGLVGLELKEKLLAAERATT
GYPDPYGGEKLALFQAIGKEVVDRALGQSWLEVQLATGGLVDPAQGVLVAPEPACHQGLL
DRETWHKLSELEPGTGDLRFLDPNTLERLTYHQLLERCVRAPGSGLALLPLKITFRSMGG
AVSAAELLEVGILDEQAVQGLREGRLAAVDVSARAEVRRYLEGTGSVAGVVLLPEGHKKS
FFQAATEHLLPMGTALPLLEAQAATHTLVDPITGQRLWVDEAVRAGLVSPELHEQLLVAE
QAVTGHHDPFSGSQIPLFQAMKKGLVDRPLALRLLDAQLATGGLVCPARRLRLPLEAALR
CGCLDEDTQRQLSQAGSFSDGTHGGLRYEQLLALCVTDPETGLAFLPLSGGPRGGEPQGP
PFIKYSTRQALSTATATVSVGKFRGRPVSLWELLFSEAISSEQRAMLAQQYQEGTLSVEK
LAAKLSATLEQAAATARVTFSGLRDTVTPGELLKAEIIDQDLYERLEHGQATAKDVGSLA

SVQRYLQGTGCIAGLLLPGSQERLSIYEARCKGLLRPGTALILLEAQAATGFIIDPKANK
GHSVEEALRAAVIGPDVFAKLLSAERAVTGYTDPYTGQQISLFQAMQKGLIVREHGIRLL
EAQIATGGVIDPVHSHRVPVDVAYRRGYFDQMLNLILLDPSDDTKGFFDPNTHENLTYLQ
LLERCVRDPETGLYLLPLSSTQSPLVDSATQQAFQNLLLSVKYGRFQGQRVSAWELINSE
YFSEGRRRQLLRRYRQREVTLGQVAKLLEAETQRQADIMLPALRSRVTVHQLLEAGIIDQ
QLLDQVLAGTISPEALLLMDGVRRYLCGLGAVGGVRLLPSGQRLSLYQAMRQKLLGPRVA
LALLEAQAATGTIMDPHSPESLSVDEAVRRGVVGPELYGRLKRAEGAIAGFRDPFSGKQV
SVFQAMKKGLIPWEQAARLLEAQVATGGIIDPTSHHHLPMPVAIQRGYVDQEMETALSSS
SETFPTPDGQGRTSYAQLLEECPRDETSGLHLLPLPESAPALPTEEQVQRSLQAVPGAKD
GTSLWDLLSSCHFTEEQRRGLLEDVQEGRTTVPQLLASVQRWVQETKLLAQARVMVPGPR
GEVPAVWLLDAGIITQETLEALAQGTQSPAQVAEQPAVKACLWGTGCVAGVLLQPSGAKA
SIAQAVRDGLLPTGLGQRLLEAQVASGFLVDPLNNQRLSVEDAVKVGLVGRELSEQLGQA
ERAAAGYPDPYSRASLSLWQAMEKGLVPQNEGLPLLQVQLATGGVVDPVHGVHLPQAAAC
RLGLLDTQTSQVLTAVDKDNKFFFDPSARDQVTYQQLRERCVCDSETGLLLLPLPSDTVL
EVDDHTAVALRAMKVPVSTGRFKGCSVSLWDLLLSEYVGADKRRELVALCRSGRAAALRQ
VVSAVTTLVEAAERQPLQATFRGLRKQVSARDLFRAQLISRKTLDELSQGTTTVKEVAEM
DSVKRSLEGGNFIAGVLIQGTQERMSIPEALRRHILRPGTALVLLEAQAATGFIIDPVEN
RKLTVEEAFKAGMFGKETYVKLLSAERAVTGYTDPYTGQQISLFQAMQKDLIVREHGIRL
LEAQIATGGIIDPVHSHRVPVDVAYRCGYFDEEMNRILADPSDDTKGFFDPNTHENLTYL
QLLERCVEDPETGLYLLQIIKKGENYVYINEATRHVLQSRTAKMRVGRFADQVVSFWDLL
SSPYFTEDRKRELIQEYGAQSGGLEKLLEIITTTIEETETQNQGIKVAAIRGEVTAADLF
NSRVIDQKTLHTLRVGRTGGQALSTLECVKPYLEGSGCIAGVTVPSTREVMSLHEASRKE
LIPAAFATWLLEAQAATGFLLDPCTRQKLSVDEAVDVGLVNEELRERLLKAERAATGYRD
PATGDTIPLFQAMQKQLIEKAEALRLLEVQVATGGVIDPQHHHRLPLETAYRRGCLHKDI
YALISDQKHMRKRFVDPNTQEKVSYRELQERCRPQEDTGWLLFPVNKAARDSEHIDDETR
RALEAEQVEITVGRFRGQKPTLWALLNSEYVTEEKKLQLVRMYRTHTRRALQTVAQLILE
LIEKQETSNKHLWFQGIRRQITASELLSSAIITEEMLQDLETGRSTTQELMEDDRVKRYL
EGTSCIAGVLVPAKDQPGRQEKMSIYQAMWKGVLRPGTALVLLEAQAATGFVIDPVRNLR
LSVEEAVAAGVVGGEIQEKLLSAERAVTGYTDPYTGQQISLFQAMQKDLIVREHGIRLLE
AQIATGGVIDPVHSHRVPVDVAYRRGYFDEEMNRVLADPSDDTKGFFDPNTHENLTYVQL
LRRCVPDPDTGLYMLQLAGRGSAVHQLSEELRCALRDARVTPGSGALQGQSVSVWELLFY
REVSEDRRQDLLSRYRAGTLTVEELGATLTSLLAQAQAQARAEAEAGSPRPDPREALRAA
TMEVKVGRLRGRAVPVWDVLASGYVSRAAREELLAEFGSGTLDLPALTRRLTAIIEEAEE
APGARPQLQDAWRGPREPGPAGRGDGDSGRSQREGQGEGETQEAAAAAAAAAARRQEQTL
RDATMEVQRGQFQGRPVSVWDVLFSSYLSEARRDELLAQHAAGALGLPDLVAVLTRVIEE
TEERLSKVSFRGLRRQVSASELHTSGILGPETLRDLAQGTKTLQEVTEMDSVKRYLEGTS
CIAGVLVPAKDQPGRQEKMSIYQAMWKGVLRPGTALVLLEAQAATGFVIDPVRNLRLSVE
EAVAAGVVGGEIQEKLLSAERAVTGYTDPYTGQQISLFQAMQKDLIVREHGIRLLEAQIA
TGGVIDPVHSHRVPVDVAYQRGYFDEEMNRVLADPSDDTKGFFDPNTHENLTYVQLLRRC
VPDPDTGLYMLQLAGRGSAVHQLSEELRCALRDARVTPGSGALQGQSVSVWELLFYREVS
EDRRQDLLSRYRAGTLTVEELGATLTSLLAQAQAQARAEAEAGSPRPDPREALRAATMEV
KVGRLRGRAVPVWDVLASGYVSRAAREELLAEFGSGTLDLPALTRRLTAIIEEAEEAPGA
RPQLQDAWRGPREPGPAGRGDGDSGRSQREGQGEGETQEAAAAARRQEQTLRDATMEVQR
GQFQGRPVSVWDVLFSSYLSEARRDELLAQHAAGALGLPDLVAVLTRVIEETEERLSKVS
FRGLRCQVSASELHTSGILGPETLRDLAQGTKTLQEVTEMDSVKRYLEGTSCIAGVLVPA
KDQPGRQEKMSIYQAMWKGVLRPGTALVLLEAQAATGFVIDPVRNLRLSVEEAVAAGVVG
GEIQEKLLSAERAVTGYTDPYTGQQISLFQAMQKDLIVREHGIRLLEAQIATGGVIDPVH

SHRVPVDVAYRRGYFDEEMNRVLADPSDDTKGFFDPNTHENLTYVQLLRRCVPDPDTGLY
MLQLAGRGSAVHQLSEELRCALRDARVTPGSGALQGQSVSVWELLFYREVSEDRRQDLLS
RYRAGTLTVEELGATLTSLLAQAQAQARAEAEAGSPRPDPREALRAATMEVKVGRLRGRA
VPVWDVLASGYVSRAAREELLAEFGSGTLDLPALTRRLTAIIEEAEEAPGARPQLQDAWR
GPREPGPAGRGDGDSGRSQREGQGEGETQEAAAATAAARRQEQTLRDATMEVQRGQFQGR
PVSVWDVLFSSYLSEARRDELLAQHAAGALGLPDLVAVLTRVIEETEERLSKVSFRGLRR
QVSASELHTSGILGPETLRDLAQGTKTLQEVTEMDSVKRYLEGTSCIAGVLVPAKDQPGR
QEKMSIYQAMWKGVLRPGTALVLLEAQAATGFVIDPVRNLRLSVEEAVAAGVVGGEIQEK
LLSAERAVTGYTDPYTGQQISLFQAMQKDLIVREHGIRLLEAQIATGGVIDPVHSHRVPV
DVAYRRGYFDEEMNRVLADPSDDTKGFFDPNTHENLTYVQLLRRCVPDPDTGLYMLQLAG
RGSAVHQLSEELRCALRDARVTPGSGALQGQSVSVWELLFYREVSEDRRQDLLSRYRAGT
LTVEELGATLTSLLAQAQAQARAEAEAGSPRPDPREALRAATMEVKVGRLRGRAVPVWDV
LASGYVSRAAREELLAEFGSGTLDLPALTRRLTAIIEEAEEAPGARPQLQDAWRGPREPG
PAGRGDGDSGRSQREGQGEGETQEAAAATAAARRQEQTLRDATMEVQRGQFQGRPVSVWD
VLFSSYLSEARRDELLAQHAAGALGLPDLVAVLTRVIEETEERLSKVSFRGLRRQVSASE
LHTSGILGPETLRDLAQGTKTLQEVTEMDSVKRYLEGTSCIAGVLVPAKDQPGHQEKMSI
YQAMWKGVLRPGTALVLLEAQAATGFVIDPVRNLRLSVEEAVAAGVVGGEIQEKLLSAER
AVTGYTDPYTGQQISLFQAMQKDLIVREHGIRLLEAQIATGGVIDPVHSHRVPVDVAYRR
GYFDEEMNRVLAHPSDDTKGFFDPNTHENLTYVQLLRRCVPDPDTGLYMLQLAGRGSAVH
QLSEELRCALRDARVMPGSGALQGQSVSVWELLFYREVSEDRRQDLLSRYRAGTLTVEEL
GATLTSLLAQAQAQARAEAEAEAGSPRPDPREALRAATMEVKVGRLRGRAVPVWDVLASG
YVSGAAREELLAEFGSGTLDLPALTRRLTAIIEEAEEAPGARPQLQDAWRGPREPGPAGR
GDGDSGRSQREGQGEGETQEAAAAARRQEQTLRDATMEVQRGQFQGRPVSVWDVLFSSYL
SEAHRDELLAQHAAGALGLPDLVAVLTRVIEETEERLSKVSFRGLRRQVSASELHTSGIL
GPETLRDLAQGTKTLQEVTEMDSVKRYLEGTSCIAGVLVPAKDQPGRQEKMSIYQAMWKG
VLRPGTALVLLEAQAATGFVIDPVRNLRLSVEEAVAAGVVGGEIQEKLLSAERAVTGYTD
PYTGQQISLFQAMQKDLIVREHGIRLLEAQIATGGVIDPVHSHRVPVDVAYRRGYFDEEM
NRVLADPSDDTKGFFDPNTHENLTYLQLLQRATLDPETGLLFLSLSLQ
>sp|Q5D862|FILA2_HUMAN Filaggrin-2 OS=Homo sapiens OX=9606 GN=FLG2
PE=1 SV=1
MTDLLRSVVTVIDVFYKYTKQDGECGTLSKGELKELLEKELHPVLKNPDDPDTVDVIMHM
LDRDHDRRLDFTEFLLMIFKLTMACNKVLSKEYCKASGSKKHRRGHRHQEEESETEEDEE
DTPGHKSGYRHSSWSEGEEHGYSSGHSRGTVKCRHGSNSRRLGRQGNLSSSGNQEGSQKR
YHRSSCGHSWSGGKDRHGSSSVELRERINKSHISPSRESGEEYESGSGSNSWERKGHGGL
SCGLETSGHESNSTQSRIREQKLGSSCSGSGDSGRRSHACGYSNSSGCGRPQNASSSCQS
HRFGGQGNQFSYIQSGCQSGIKGGQGHGCVSGGQPSGCGQPESNPCSQSYSQRGYGAREN
GQPQNCGGQWRTGSSQSSCCGQYGSGGSQSCSNGQHEYGSCGRFSNSSSSNEFSKCDQYG
SGSSQSTSFEQHGTGLSQSSGFEQHVCGSGQTCGQHESTSSQSLGYDQHGSSSGKTSGFG
QHGSGSGQSSGFGQCGSGSGQSSGFGQHGSVSGQSSGFGQHGSVSGQSSGFGQHESRSRQ
SSYGQHGSGSSQSSGYGQYGSRETSGFGQHGLGSGQSTGFGQYGSGSGQSSGFGQHGSGS
GQSSGFGQHESRSGQSSYGQHSSGSSQSSGYGQHGSRQTSGFGQHGSGSSQSTGFGQYGS
GSGQSSGFGQHVSGSGQSSGFGQHESRSGHSSYGQHGFGSSQSSGYGQHGSSSGQTSGFG
QHELSSGQSSSFGQHGSGSGQSSGFGQHGSGSGQSSGFGQHESRSGQSSYGQHSSGSSQS
SGYGQHGSRQTSGFGQHGSGSSQSTGFGQYGSGSGQSAGFGQHGSGSGQSSGFGQHESRS
HQSSYGQHGSGSSQSSGYGQHGSSSGQTSGFGQHRSSSGQYSGFGQHGSGSGQSSGFGQH
GTGSGQYSGFGQHESRSHQSSYGQHGSGSSQSSGYGQHGSSSGQTFGFGQHRSGSGQSSG
FGQHGSGSGQSSGFGQHESGSGKSSGFGQHESRSSQSNYGQHGSGSSQSSGYGQHGSSSG

QTTGFGQHRSSSGQYSGFGQHGSGSDQSSGFGQHGTGSGQSSGFGQYESRSRQSSYGQHG
SGSSQSSGYGQHGSNSGQTSGFGQHRPGSGQSSGFGQYGSGSGQSSGFGQHGSGTGKSSG
FAQHEYRSGQSSYGQHGTGSSQSSGCGQHESGSGPTTSFGQHVSGSDNFSSSGQHISDSG
QSTGFGQYGSGSGQSTGLGQGESQQVESGSTVHGRQETTHGQTINTTRHSQSGQGQSTQT
GSRVTRRRRSSQSENSDSEVHSKVSHRHSEHIHTQAGSHYPKSGSTVRRRQGTTHGQRGD
TTRHGHSGHGQSTQTGSRTSGRQRFSHSDATDSEVHSGVSHRPHSQEQTHSQAGSQHGES
ESTVHERHETTYGQTGEATGHGHSGHGQSTQRGSRTTGRRGSGHSESSDSEVHSGGSHRP
QSQEQTHGQAGSQHGESGSTVHGRHGTTHGQTGDTTRHAHYHHGKSTQRGSSTTGRRGSG
HSESSDSEVHSGGSHTHSGHTHGQSGSQHGESESIIHDRHRITHGQTGDTTRHSYSGHEQ
TTQTGSRTTGRQRTSHSESTDSEVHSGGSHRPHSREHTYGQAGSQHEEPEFTVHERHGTT
HGQIGDTTGHSHSGHGQSTQRGSRTTGRQRSSHSESSDSEVHSGVSHTHTGHTHGQAGSQ
HGQSESIVPERHGTTHGQTGDTTRHAHYHHGLTTQTGSRTTGRRGSGHSEYSDSEGYSGV
SHTHSGHTHGQARSQHGESESIVHERHGTIHGQTGDTTRHAHSGHGQSTQTGSRTTGRRS
SGHSEYSDSEGHSGFSQRPHSRGHTHGQAGSQHGESESIVDERHGTTHGQTGDTSGHSQS
GHGQSTQSGSSTTGRRRSGHSESSDSEVHSGGSHTHSGHTHSQARSQHGESESTVHKRHQ
TTHGQTGDTTEHGHPSHGQTIQTGSRTTGRRGSGHSEYSDSEGPSGVSHTHSGHTHGQAG
SHYPESGSSVHERHGTTHGQTADTTRHGHSGHGQSTQRGSRTTGRRASGHSEYSDSEGHS
GVSHTHSGHAHGQAGSQHGESGSSVHERHGTTHGQTGDTTRHAHSGHGQSTQRGSRTAGR
RGSGHSESSDSEVHSGVSHTHSGHTYGQARSQHGESGSAIHGRQGTIHGQTGDTTRHGQS
GHGQSTQTGSRTTGRQRSSHSESSDSEVHSEASPTHSGHTHSQAGSRHGQSGSSGHGRQG
TTHGQTGDTTRHAHYGYGQSTQRGSRTTGRRGSGHSESSDSEVHSWGSHTHSGHIQGQAG
SQQRQPGSTVHGRLETTHGQTGDTTRHGHSGYGQSTQTGSRSSRASHFQSHSSERQRHGS
SQVWKHGSYGPAEYDYGHTGYGPSGGSRKSISNSHLSWSTDSTANKQLSRH
>sp|Q04695|K1C17_HUMAN Keratin, type I cytoskeletal 17 OS=Homo sapiens OX=9606 GN=KRT17 PE=1 SV=2
MTTSIRQFTSSSSIKGSSGLGGGSSRTSCRLSGGLGAGSCRLGSAGGLGSTLGGSSYSSC
YSFGSGGGYGSSFGGVDGLLAGGEKATMQNLNDRLASYLDKVRALEEANTELEVKIRDWY
QRQAPGPARDYSQYYRTIEELQNKILTATVDNANILLQIDNARLAADDFRTKFETEQALR
LSVEADINGLRRVLDELTLARADLEMQIENLKEELAYLKKNHEEEMNALRGQVGGEINVE
MDAAPGVDLSRILNEMRDQYEKMAEKNRKDAEDWFFSKTEELNREVATNSELVQSGKSEI
SELRRTMQALEIELQSQLSMKASLEGNLAETENRYCVQLSQIQGLIGSVEEQLAQLRCEM
EQQNQEYKILLDVKTRLEQEIATYRRLLEGEDAHLTQYKKEPVTTRQVRTIVEEVQDGKV
ISSREQVHQTTR
>sp|P13647|K2C5_HUMAN Keratin, type II cytoskeletal 5 OS=Homo sapiens OX=9606 GN=KRT5 PE=1 SV=3
MSRQSSVSFRSGGSRSFSTASAITPSVSRTSFTSVSRSGGGGGGGFGRVSLAGACGVGGY
GSRSLYNLGGSKRISISTSGGSFRNRFGAGAGGGYGFGGGAGSGFGFGGGAGGGFGLGGG
AGFGGGFGGPGFPVCPPGGIQEVTVNQSLLTPLNLQIDPSIQRVRTEEREQIKTLNNKFA
SFIDKVRFLEQQNKVLDTKWTLLQEQGTKTVRQNLEPLFEQYINNLRRQLDSIVGERGRL
DSELRNMQDLVEDFKNKYEDEINKRTTAENEFVMLKKDVDAAYMNKVELEAKVDALMDEI
NFMKMFFDAELSQMQTHVSDTSVVLSMDNNRNLDLDSIIAEVKAQYEEIANRSRTEAESW
YQTKYEELQQTAGRHGDDLRNTKHEISEMNRMIQRLRAEIDNVKKQCANLQNAIADAEQR
GELALKDARNKLAELEEALQKAKQDMARLLREYQELMNTKLALDVEIATYRKLLEGEECR
LSGEGVGPVNISVVTSSVSSGYGSGSGYGGGLGGGLGGGLGGGLAGGSSGSYYSSSSGGV
GLGGGLSVGGSGFSASSGRGLGVGFGSGGGSSSSVKFVSTTSSSRKSFKS
>sp|Q02413|DSG1_HUMAN Desmoglein-1 OS=Homo sapiens OX=9606 GN=DSG1 PE=1 SV=2

MDWSFFRVVAMLFIFLVVVEVNSEFRIQVRDYNTKNGTIKWHSIRRQKREWIKFAAACRE
GEDNSKRNPIAKIHSDCAANQQVTYRISGVGIDQPPYGIFVINQKTGEINITSIVDREVT
PFFIIYCRALNSMGQDLERPLELRVRVLDINDNPPVFSMATFAGQIEENSNANTLVMILN
ATDADEPNNLNSKIAFKIIRQEPSDSPMFIINRNTGEIRTMNNFLDREQYGQYALAVRGS
DRDGGADGMSAECECNIKILDVNDNIPYMEQSSYTIEIQENTLNSNLLEIRVIDLDEEFS
ANWMAVIFFISGNEGNWFEIEMNERTNVGILKVVKPLDYEAMQSLQLSIGVRNKAEFHHS
IMSQYKLKASAISVTVLNVIEGPVFRPGSKTYVVTGNMGSNDKVGDFVATDLDTGRPSTT
VRYVMGNNPADLLAVDSRTGKLTLKNKVTKEQYNMLGGKYQGTILSIDDNLQRTCTGTIN
INIQSFGNDDRTNTEPNTKITTNTGRQESTSSTNYDTSTTSTDSSQVYSSEPGNGAKDLL
SDNVHFGPAGIGLLIMGFLVLGLVPFLMICCDCGGAPRSAAGFEPVPECSDGAIHSWAVE
GPQPEPRDITTVIPQIPPDNANIIECIDNSGVYTNEYGGREMQDLGGGERMTGFELTEGV
KTSGMPEICQEYSGTLRRNSMRECREGGLNMNFMESYFCQKAYAYADEDEGRPSNDCLLI
YDIEGVGSPAGSVGCCSFIGEDLDDSFLDTLGPKFKKLADISLGKESYPDLDPSWPPQST
EPVCLPQETEPVVSGHPPISPHFGTTTVISESTYPSGPGVLHPKPILDPLGYGNVTVTES
YTTSDTLKPSVHVHDNRPASNVVVTERVVGPISGADLHGMLEMPDLRDGSNVIVTERVIA
PSSSLPTSLTIHHPRESSNVVVTERVIQPTSGMIGSLSMHPELANAHNVIVTERVVSGAG
VTGISGTTGISGGIGSSGLVGTSMGAGSGALSGAGISGGGIGLSSLGGTASIGHMRSSSD
HHFNQTIGSASPSTARSRITKYSTVQYSK
>sp|P14923|PLAK_HUMAN Junction plakoglobin OS=Homo sapiens OX=9606 GN=JUP PE=1 SV=3
MEVMNLMEQPIKVTEWQQTYTYDSGIHSGANTCVPSVSSKGIMEEDEACGRQYTLKKTTT
YTQGVPPSQGDLEYQMSTTARAKRVREAMCPGVSGEDSSLLLATQVEGQATNLQRLAEPS
QLLKSAIVHLINYQDDAELATRALPELTKLLNDEDPVVVTKAAMIVNQLSKKEASRRALM
GSPQLVAAVVRTMQNTSDLDTARCTTSILHNLSHHREGLLAIFKSGGIPALVRMLSSPVE
SVLFYAITTLHNLLLYQEGAKMAVRLADGLQKMVPLLNKNNPKFLAITTDCLQLLAYGNQ
ESKLIIILANGGPQALVQIMRNYSYEKLLWTTSRVLKVLSVCPSNKPAIVEAGGMQALGKH
LTSNSPRLVQNCLWTLRNLSDVATKQEGLESVLKILVNQLSVDDVNVLTCATGTLSNLTC
NNSKNKTLVTQNSGVEALIHAILRAGDKDDITEPAVCALRHLTSRHPEAEMAQNSVRLNY
GIPAIVKLLNQPNQWPLVKATIGLIRNLALCPANHAPLQEAAVIPRLVQLLVKAHQDAQR
HVAAGTQQPYTDGVRMEEIVEGCTGALHILARDPMNRMEIFRLNTIPLFVQLLYSSVENI
QRVAAGVLCELAQDKEAADAIDAEGASAPLMELLHSRNEGTATYAAAVLFRISEDKNPDY
RKRVSVELTNSLFKHDPAAWEAAQSMIPINEPYGDDMDATYRPMYSSDVPLDPLEMHMDM
DGDYPIDTYSDGLRPPYPTADHMLA
>sp|Q6UWP8|SBSN_HUMAN Suprabasin OS=Homo sapiens OX=9606 GN=SBSN PE=1 SV=2
MHLARLVGSCSLLLLLGALSGWAASDDPIEKVIEGINRGLSNAEREVGKALDGINSGITH
AGREVEKVFNGLSNMGSHTGKELDKGVQGLNHGMDKVAHEINHGIGQAGKEAEKLGHGVN
NAAGQVGKEADKLIHHGVHHGANQAGSEAGKFGQGVDNAAGQAGNEAGRFGQGVHHAAGQ
AGNEAGRFGQGVHHAAGQAGNEAGRFGQGAHHGLSEGWKETEKFGQGIHHAAGQVGKEAE
KFGQGAHHAAGQAGNEAGRFGQGVHHGLSEGWKETEKFGQGVHHTAGQVGKEAEKFGQGA
HHAAGQAGNEAGRFGQGAHHAAGQAGNEAGRFGQGVHHGLSEGWKETEKFGQGVHHAASQ
FGKETEKLGHGVHHGVNEAWKEAEKFGQGVHHAASQVGKEEDRVVQGLHHGVSQAGREAG
QFGHDIHHTAGQAGKEGDIAVHGVQPGVHEAGKEAGQFGQGVHHTLEQAGKEADKAVQGF
HTGVHQAGKEAEKLGQGVNHAADQAGKEVEKLGQGAHHAAGQAGKELQNAHNGVNQASKE
ANQLLNGNHQSGSSSHQGGATTTPLASGASVNTPFINLPALWRSVANIMP
>sp|O43790|KRT86_HUMAN Keratin, type II cuticular Hb6 OS=Homo sapiens OX=9606 GN=KRT86 PE=1 SV=1

MTCGSYCGGRAFSCISACGPRPGRCCITAAPYRGISCYRGLTGGFGSHSVCGGFRAGSCG
RSFGYRSGGVCGPSPPCITTVSVNESLLTPLNLEIDPNAQCVKQEEKEQIKSLNSRFAAF
IDKVRFLEQQNKLLETKLQFYQNRECCQSNLEPLFEGYIETLRREAECVEADSGRLASEL
NHVQEVLEGYKKKYEEEVSLRATAENEFVALKKDVDCAYLRKSDLEANVEALIQEIDFLR
RLYEEEIRVLQSHISDTSVVVKLDNSRDLNMDCIIAEIKAQYDDIVTRSRAEAESWYRSK
CEEMKATVIRHGETLRRTKEEINELNRMIQRLTAEVENAKCQNSKLEAAVAQSEQQGEAA
LSDARCKLAELEGALQKAKQDMACLIREYQEVMNSKLGLDIEIATYRRLLEGEEQRLCEG
VGSVNVCVSSSRGGVVCGDLCASTTAPVVSTRVSSVPSNSNVVVGTTNACAPSARVGVCG
GSCKRC
>sp|P78386|KRT85_HUMAN Keratin, type II cuticular Hb5 OS=Homo sapiens OX=9606 GN=KRT85 PE=1 SV=1
MSCRSYRISSGCGVTRNFSSCSAVAPKTGNRCCISAAPYRGVSCYRGLTGFGSRSLCNLG
SCGPRIAVGGFRAGSCGRSFGYRSGGVCGPSPPCITTVSVNESLLTPLNLEIDPNAQCVK
QEEKEQIKSLNSRFAAFIDKVRFLEQQNKLLETKWQFYQNQRCCESNLEPLFSGYIETLR
REAECVEADSGRLASELNHVQEVLEGYKKKYEEEVALRATAENEFVVLKKDVDCAYLRKS
DLEANVEALVEESSFLRRLYEEEIRVLQAHISDTSVIVKMDNSRDLNMDCIIAEIKAQYD
DVASRSRAEAESWYRSKCEEMKATVIRHGETLRRTKEEINELNRMIQRLTAEIENAKCQR
AKLEAAVAEAEQQGEAALSDARCKLAELEGALQKAKQDMACLLKEYQEVMNSKLGLDIEI
ATYRRLLEGEEHRLCEGVGSVNVCVSSSRGGVSCGGLSYSTTPGRQITSGPSAIGGSITV
VAPDSCAPCQPRSSSFSCGSSRSVRFA
>sp|Q14533|KRT81_HUMAN Keratin, type II cuticular Hb1 OS=Homo sapiens OX=9606 GN=KRT81 PE=1 SV=3
MTCGSGFGGRAFSCISACGPRPGRCCITAAPYRGISCYRGLTGGFGSHSVCGGFRAGSCG
RSFGYRSGGVCGPSPPCITTVSVNESLLTPLNLEIDPNAQCVKQEEKEQIKSLNSRFAAF
IDKVRFLEQQNKLLETKLQFYQNRECCQSNLEPLFEGYIETLRREAECVEADSGRLASEL
NHVQEVLEGYKKKYEEEVSLRATAENEFVALKKDVDCAYLRKSDLEANVEALIQEIDFLR
RLYEEEILILQSHISDTSVVVKLDNSRDLNMDCIIAEIKAQYDDIVTRSRAEAESWYRSK
CEEMKATVIRHGETLRRTKEEINELNRMIQRLTAEVENAKCQNSKLEAAVAQSEQQGEAA
LSDARCKLAELEGALQKAKQDMACLIREYQEVMNSKLGLDIEIATYRRLLEGEEQRLCEG
IGAVNVCVSSSRGGVVCGDLCVSGSRPVTGSVCSAPCNGNVAVSTGLCAPCGQLNTTCGG
GSCGVGSCGISSLGVGSCGSSCRKC
>sp|Q9NSB2|KRT84_HUMAN Keratin, type II cuticular Hb4 OS=Homo sapiens OX=9606 GN=KRT84 PE=2 SV=2
MSCRSYRVSSGHRVGNFSSCSAMTPQNLNRFRANSVSCWSGPGFRGLGSFGSRSVITFGS
YSPRIAAVGSRPIHCGVRFGAGCGMGFGDGRGVGLGPRADSCVGLGFGAGSGIGYGFGGP
GFGYRVGGVGVPAAPSITAVTVNKSLLTPLNLEIDPNAQRVKKDEKEQIKTLNNKFASFI
DKVRFLEQQNKLLETKWSFLQEQKCIRSNLEPLFESYITNLRRQLEVLVSDQARLQAERN
HLQDVLEGFKKKYEEEVVCRANAENEFVALKKDVDAAFMNKSDLEANVDTLTQEIDFLKT
LYMEEIQLLQSHISETSVIVKMDNSRDLNLDGIIAEVKAQYEEVARRSRADAEAWYQTKY
EEMQVTAGQHCDNLRNIRNEINELTRLIQRLKAEIEHAKAQRAKLEAAVAEAEQQGEATL
SDAKCKLADLECALQQAKQDMARQLCEYQELMNAKLGLDIEIATYRRLLEGEESRLCEGV
GPVNISVSSSRGGLVCGPEPLVAGSTLSRGGVTFSGSSSVCATSGVLASCGPSLGGARVA
PATGDLLSTGTRSGSMLISEACVPSVPCPLPTQGGFSSCSGGRSSSVRFVSTTTSCRTKY
>sp|Q14525|KT33B_HUMAN Keratin, type I cuticular Ha3-II OS=Homo sapiens OX=9606 GN=KRT33B PE=1 SV=3
MPYNFCLPSLSCRTSCSSRPCVPPSCHGYTLPGACNIPANVSNCNWFCEGSFNGSEKETM
QFLNDRLASYLEKVRQLERDNAELENLIRERSQQQEPLLCPSYQSYFKTIEELQQKILCS

KSENARLVVQIDNAKLAADDFRTKYQTEQSLRQLVESDINSLRRILDELTLCRSDLEAQM
ESLKEELLSLKQNHEQEVNTLRCQLGDRLNVEVDAAPAVDLNQVLNETRNQYEALVETNR
REVEQWFATQTEELNKQVVSSSEQLQSYQAEIIELRRTVNALEIELQAQHNLRYSLENTL
TESEARYSSQLSQVQSLITNVESQLAEIRSDLERQNQEYQVLLDVRARLECEINTYRSLL
ESEDCKLPSNPCATTNACEKPIGSCVTNPCGPRSRCGPCNTFGY
>sp|Q15323|K1H1_HUMAN Keratin, type I cuticular Ha1 OS=Homo sapiens OX=9606 GN=KRT31 PE=1 SV=3
MPYNFCLPSLSCRTSCSSRPCVPPSCHSCTLPGACNIPANVSNCNWFCEGSFNGSEKETM
QFLNDRLASYLEKVRQLERDNAELENLIRERSQQQEPLLCPSYQSYFKTIEELQQKILCT
KSENARLVVQIDNAKLAADDFRTKYQTELSLRQLVESDINGLRRILDELTLCKSDLEAQV
ESLKEELLCLKSNHEQEVNTLRCQLGDRLNVEVDAAPTVDLNRVLNETRSQYEALVETNR
REVEQWFTTQTEELNKQVVSSSEQLQSYQAEIIELRRTVNALEIELQAQHNLRDSLENTL
TESEARYSSQLSQVQSLITNVESQLAEIRSDLERQNQEYQVLLDVRARLECEINTYRSLL
ESEDCNLPSNPCATTNACSKPIGPCLSNPCTSCVPPAPCTPCAPRPRCGPCNSFVR
>sp|P78385|KRT83_HUMAN Keratin, type II cuticular Hb3 OS=Homo sapiens OX=9606 GN=KRT83 PE=1 SV=2
MTCGFNSIGCGFRPGNFSCVSACGPRPSRCCITAAPYRGISCYRGLTGGFGSHSVCGGFR
AGSCGRSFGYRSGGVCGPSPPCITTVSVNESLLTPLNLEIDPNAQCVKQEEKEQIKSLNS
RFAAFIDKVRFLEQQNKLLETKLQFYQNRECCQSNLEPLFAGYIETLRREAECVEADSGR
LASELNHVQEVLEGYKKKYEEEVALRATAENEFVALKKDVDCAYLRKSDLEANVEALIQE
IDFLRRLYEEEIRILQSHISDTSVVVKLDNSRDLNMDCIVAEIKAQYDDIATRSRAEAES
WYRSKCEEMKATVIRHGETLRRTKEEINELNRMIQRLTAEVENAKCQNSKLEAAVAQSEQ
QGEAALSDARCKLAELEGALQKAKQDMACLIREYQEVMNSKLGLDIEIATYRRLLEGEEQ
RLCEGVEAVNVCVSSSRGGVVCGDLCVSGSRPVTGSVCSAPCNGNLVVSTGLCKPCGQLN
TTCGGGSCGQGRH
>sp|P29508|SPB3_HUMAN Serpin B3 OS=Homo sapiens OX=9606 GN=SERPINB3 PE=1 SV=2
MNSLSEANTKFMFDLFQQFRKSKENNIFYSPISITSALGMVLLGAKDNTAQQIKKVLHFD
QVTENTTGKAATYHVDRSGNVHHQFQKLLTEFNKSTDAYELKIANKLFGEKTYLFLQEYL
DAIKKFYQTSVESVDFANAPEESRKKINSWVESQTNEKIKNLIPEGNIGSNTTLVLVNAI
YFKGQWEKKFNKEDTKEEKFWPNKNTYKSIQMMRQYTSFHFASLEDVQAKVLEIPYKGKD
LSMIVLLPNEIDGLQKLEEKLTAEKLMEWTSLQNMRETRVDLHLPRFKVEESYDLKDTLR
TMGMVDIFNGDADLSGMTGSRGLVLSGVLHKAFVEVTEEGAEAAAATAVVGFGSSPTSTN
EEFHCNHPFLFFIRQNKTNSILFYGRFSSP
>sp|O76011|KRT34_HUMAN Keratin, type I cuticular Ha4 OS=Homo sapiens OX=9606 GN=KRT34 PE=1 SV=2
MLYAKPPPTINGIKGLQRKERLKPAHIHLQQLTCFSITCSSTMSYSCCLPSLGCRTSCSS
RPCVPPSCHGYTLPGACNIPANVSNCNWFCEGSFNGSEKETMQFLNDRLASYLEKVRQLE
RDNAELEKLIQERSQQQEPLLCPSYQSYFKTIEELQQKILCAKAENARLVVNIDNAKLAS
DDFRSKYQTEQSLRLLVESDINSIRRILDELTLCKSDLESQVESLREELICLKKNHEEEV
NTLRSQLGDRLNVEVDTAPTVDLNQVLNETRSQYEALVEINRREVEQWFATQTEELNKQV
VSSSEQLQSCQAEIIELRRTVNALEIELQAQHNLRDSLENTLTESEAHYSSQLSQVQSLI
TNVESQLAEIRCDLERQNQEYQVLLDVRARLECEINTYRSLLESEDCKLPCNPCATTNAS
GNSCGPCGTSQKGCCN
>sp|Q08554|DSC1_HUMAN Desmocollin-1 OS=Homo sapiens OX=9606 GN=DSC1 PE=1 SV=2
MALASAAPGSIFCKQLLFSLLVLTLLCDACQKVYLRVPSHLQAETLVGKVNLEECLKSAS

LIRSSDPAFRILEDGSIYTTHDLILSSERKSFSIFLSDGQRREQQEIKVVLSARENKSPK
KRHTKDTALKRSKRRWAPIPASLMENSLGPFPQHVQQIQSDAAQNYTIFYSISGPGVDKE
PFNLFYIEKDTGDIFCTRSIDREKYEQFALYGYATTADGYAPEYPLPLIIKIEDDNDNAP
YFEHRVTIFTVPENCRSGTSVGKVTATDLDEPDTLHTRLKYKILQQIPDHPKHFSIHPDT
GVITTTTPFLDREKCDTYQLIMEVRDMGGQPFGLFNTGTITISLEDENDNPPSFTETSYV
TEVEENRIDVEILRMKVQDQDLPNTPHSKAVYKILQGNENGNFIISTDPNTNEGVLCVVK
PLNYEVNRQVILQVGVINEAQFSKAASSQTPTMCTTTVTVKIIDSDEGPECHPPVKVIQS
QDGFPAGQELLGYKALDPEISSGEGLRYQKLGDEDNWFEINQHTGDLRTLKVLDRESKFV
KNNQYNISVVAVDAVGRSCTGTLVVHLDDYNDHAPQIDKEVTICQNNEDFAVLKPVDPDG
PENGPPFQFFLDNSASKNWNIEEKDGKTAILRQRQNLDYNYYSVPIQIKDRHGLVATHML
TVRVCDCSTPSECRMKDKSTRDVRPNVILGRWAILAMVLGSVLLLCILFTCFCVTAKRTV
KKCFPEDIAQQNLIVSNTEGPGEEVTEANIRLPMQTSNICDTSMSVGTVGGQGIKTQQSF
EMVKGGYTLDSNKGGGHQTLESVKGVGQGDTGRYAYTDWQSFTQPRLGEKVYLCGQDEEH
KHCEDYVCSYNYEGKGSLAGSVGCCSDRQEEEGLEFLDHLEPKFRTLAKTCIKK
>sp|P07476|INVO_HUMAN Involucrin OS=Homo sapiens OX=9606 GN=IVL PE=1 SV=2
MSQQHTLPVTLSPALSQELLKTVPPPVNTHQEQMKQPTPLPPPCQKVPVELPVEVPSKQE
EKHMTAVKGLPEQECEQQQKEPQEQELQQQHWEQHEEYQKAENPEQQLKQEKTQRDQQLN
KQLEEEKKLLDQQLDQELVKRDEQLGMKKEQLLELPEQQEGHLKHLEQQEGQLKHPEQQE
GQLELPEQQEGQLELPEQQEGQLELPEQQEGQLELPEQQEGQLELPEQQEGQLELPQQQE
GQLELSEQQEGQLELSEQQEGQLKHLEHQEGQLEVPEEQMGQLKYLEQQEGQLKHLDQQE
KQPELPEQQMGQLKHLEQQEGQPKHLEQQEGQLEQLEEQEGQLKHLEQQEGQLEHLEHQE
GQLGLPEQQVLQLKQLEKQQGQPKHLEEEEGQLKHLVQQEGQLKHLVQQEGQLEQQERQV
EHLEQQVGQLKHLEEQEGQLKHLEQQQGQLEVPEQQVGQPKNLEQEEKQLELPEQQEGQV
KHLEKQEAQLELPEQQVGQPKHLEQQEKHLEHPEQQDGQLKHLEQQEGQLKDLEQQKGQL
EQPVFAPAPGQVQDIQPALPTKGEVLLPVEHQQQKQEVQWPPKHK
>sp|P36952|SPB5_HUMAN Serpin B5 OS=Homo sapiens OX=9606 GN=SERPINB5 PE=1 SV=2
MDALQLANSAFAVDLFKQLCEKEPLGNVLFSPICLSTSLSLAQVGAKGDTANEIGQVLHF
ENVKDVPFGFQTVTSDVNKLSSFYSLKLIKRLYVDKSLNLSTEFISSTKRPYAKELETVD
FKDKLEETKGQINNSIKDLTDGHFENILADNSVNDQTKILVVNAAYFVGKWMKKFSESET
KECPFRVNKTDTKPVQMMNMEATFCMGNIDSINCKIIELPFQNKHLSMFILLPKDVEDES
TGLEKIEKQLNSESLSQWTNPSTMANAKVKLSIPKFKVEKMIDPKACLENLGLKHIFSED
TSDFSGMSETKGVALSNVIHKVCLEITEDGGDSIEVPGARILQHKDELNADHPFIYIIRH
NKTRNIIFFGKFCSP
>sp|P06733|ENOA_HUMAN Alpha-enolase OS=Homo sapiens OX=9606 GN=ENO1 PE=1 SV=2
MSILKIHAREIFDSRGNPTVEVDLFTSKGLFRAAVPSGASTGIYEALELRDNDKTRYMGK
GVSKAVEHINKTIAPALVSKKLNVTEQEKIDKLMIEMDGTENKSKFGANAILGVSLAVCK
AGAVEKGVPLYRHIADLAGNSEVILPVPAFNVINGGSHAGNKLAMQEFMILPVGAANFRE
AMRIGAEVYHNLKNVIKEKYGKDATNVGDEGGFAPNILENKEGLELLKTAIGKAGYTDKV
VIGMDVAASEFFRSGKYDLDFKSPDDPSRYISPDQLADLYKSFIKDYPVVSIEDPFDQDD
WGAWQKFTASAGIQVVGDDLTVTNPKRIAKAVNEKSCNCLLLKVNQIGSVTESLQACKLA
QANGWGVMVSHRSGETEDTFIADLVVGLCTGQIKTGAPCRSERLAKYNQLLRIEEELGSK
AKFAGRNFRNPLAK
>sp|P13929|ENOB_HUMAN Beta-enolase OS=Homo sapiens OX=9606 GN=ENO3 PE=1 SV=5

MAMQKIFAREILDSRGNPTVEVDLHTAKGRFRAAVPSGASTGIYEALELRDGDKGRYLGK
GVLKAVENINNTLGPALLQKKLSVVDQEKVDKFMIELDGTENKSKFGANAILGVSLAVCK
AGAAEKGVPLYRHIADLAGNPDLILPVPAFNVINGGSHAGNKLAMQEFMILPVGASSFKE
AMRIGAEVYHHLKGVIKAKYGKDATNVGDEGGFAPNILENNEALELLKTAIQAAGYPDKV
VIGMDVAASEFYRNGKYDLDFKSPDDPARHITGEKLGELYKSFIKNYPVVSIEDPFDQDD
WATWTSFLSGVNIQIVGDDLTVTNPKRIAQAVEKKACNCLLLKVNQIGSVTESIQACKLA
QSNGWGVMVSHRSGETEDTFIADLVVGLCTGQIKTGAPCRSERLAKYNQLMRIEEALGDK
AIFAGRKFRNPKAK
>sp|P09104|ENOG_HUMAN Gamma-enolase OS=Homo sapiens OX=9606 GN=ENO2 PE=1 SV=3
MSIEKIWAREILDSRGNPTVEVDLYTAKGLFRAAVPSGASTGIYEALELRDGDKQRYLGK
GVLKAVDHINSTIAPALISSGLSVVEQEKLDNLMLELDGTENKSKFGANAILGVSLAVCK
AGAAERELPLYRHIAQLAGNSDLILPVPAFNVINGGSHAGNKLAMQEFMILPVGAESFRD
AMRLGAEVYHTLKGVIKDKYGKDATNVGDEGGFAPNILENSEALELVKEAIDKAGYTEKI
VIGMDVAASEFYRDGKYDLDFKSPTDPSRYITGDQLGALYQDFVRDYPVVSIEDPFDQDD
WAAWSKFTANVGIQIVGDDLTVTNPKRIERAVEEKACNCLLLKVNQIGSVTEAIQACKLA
QENGWGVMVSHRSGETEDTFIADLVVGLCTGQIKTGAPCRSERLAKYNQLMRIEEELGDE
ARFAGHNFRNPSVL
>sp|O76009|KT33A_HUMAN Keratin, type I cuticular Ha3-I OS=Homo sapiens OX=9606 GN=KRT33A PE=2 SV=2
MSYSCGLPSLSCRTSCSSRPCVPPSCHGCTLPGACNIPANVSNCNWFCEGSFNGSEKETM
QFLNDRLASYLEKVRQLERDNAELENLIRERSQQQEPLVCASYQSYFKTIEELQQKILCS
KSENARLVVQIDNAKLASDDFRTKYETELSLRQLVESDINGLRRILDELTLCRSDLEAQV
ESLKEELLCLKQNHEQEVNTLRCQLGDRLNVEVDAAPTVDLNQVLNETRSQYEALVETNR
REVEQWFATQTEELNKQVVSSSEQLQSYQAEIIELRRTVNALEIELQAQHNLRDSLENTL
TESEARYSSQLSQVQRLITNVESQLAEIRSDLERQNQEYQVLLDVRARLECEINTYRSLL
ESEDCKLPSNPCATTNACDKSTGPCISNPCGLRARCGPCNTFGY
>sp|P14618|KPYM_HUMAN Pyruvate kinase PKM OS=Homo sapiens OX=9606 GN=PKM PE=1 SV=4
MSKPHSEAGTAFIQTQQLHAAMADTFLEHMCRLDIDSPPITARNTGIICTIGPASRSVET
LKEMIKSGMNVARLNFSHGTHEYHAETIKNVRTATESFASDPILYRPVAVALDTKGPEIR
TGLIKGSGTAEVELKKGATLKITLDNAYMEKCDENILWLDYKNICKVVEVGSKIYVDDGL
ISLQVKQKGADFLVTEVENGGSLGSKKGVNLPGAAVDLPAVSEKDIQDLKFGVEQDVDMV
FASFIRKASDVHEVRKVLGEKGKNIKIISKIENHEGVRRFDEILEASDGIMVARGDLGIE
IPAEKVFLAQKMMIGRCNRAGKPVICATQMLESMIKKPRPTRAEGSDVANAVLDGADCIM
LSGETAKGDYPLEAVRMQHLIAREAEAAIYHLQLFEELRRLAPITSDPTEATAVGAVEAS
FKCCSGAIIVLTKSGRSAHQVARYRPRAPIIAVTRNPQTARQAHLYRGIFPVLCKDPVQE
AWAEDVDLRVNFAMNVGKARGFFKKGDVVIVLTGWRPGSGFTNTMRVVPVP
>sp|P30613|KPYR_HUMAN Pyruvate kinase PKLR OS=Homo sapiens OX=9606 GN=PKLR PE=1 SV=2
MSIQENISSLQLRSWVSKSQRDLAKSILIGAPGGPAGYLRRASVAQLTQELGTAFFQQQQ
LPAAMADTFLEHLCLLDIDSEPVAARSTSIIATIGPASRSVERLKEMIKAGMNIARLNFS
HGSHEYHAESIANVREAVESFAGSPLSYRPVAIALDTKGPEIRTGILQGGPESEVELVKG
SQVLVTVDPAFRTRGNANTVWVDYPNIVRVVPVGGRIYIDDGLISLVVQKIGPEGLVTQV
ENGGVLGSRKGVNLPGAQVDLPGLSEQDVRDLRFGVEHGVDIVFASFVRKASDVAAVRAA
LGPEGHGIKIISKIENHEGVKRFDEILEVSDGIMVARGDLGIEIPAEKVFLAQKMMIGRC
NLAGKPVVCATQMLESMITKPRPTRAETSDVANAVLDGADCIMLSGETAKGNFPVEAVKM

QHAIAREAEAAVYHRQLFEELRRAAPLSRDPTEVTAIGAVEAAFKCCAAAIIVLTTTGRS
AQLLSRYRPRAAVIAVTRSAQAARQVHLCRGVFPLLYREPPEAIWADDVDRRVQFGIESG
KLRGFLRVGDLVIVVTGWRPGSGYTNIMRVLSIS
>sp|O95678|K2C75_HUMAN Keratin, type II cytoskeletal 75 OS=Homo sapiens OX=9606 GN=KRT75 PE=1 SV=2
MSRQSSITFQSGSRRGFSTTSAITPAAGRSRFSSVSVARSAAGSGGLGRISSAGASFGSR
SLYNLGGAKRVSINGCGSSCRSGFGGRASNRFGVNSGFGYGGGVGGGFSGPSFPVCPPGG
IQEVTVNQSLLTPLHLQIDPTIQRVRAEEREQIKTLNNKFASFIDKVRFLEQQNKVLETK
WALLQEQGSRTVRQNLEPLFDSYTSELRRQLESITTERGRLEAELRNMQDVVEDFKVRYE
DEINKRTAAENEFVALKKDVDAAYMNKVELEAKVKSLPEEINFIHSVFDAELSQLQTQVG
DTSVVLSMDNNRNLDLDSIIAEVKAQYEDIANRSRAEAESWYQTKYEELQVTAGRHGDDL
RNTKQEISEMNRMIQRLRAEIDSVKKQCSSLQTAIADAEQRGELALKDARAKLVDLEEAL
QKAKQDMARLLREYQELMNIKLALDVEIATYRKLLEGEECRLSGEGVSPVNISVVTSTLS
SGYGSGSSIGGGNLGLGGGSGYSFTTSGGHSLGAGLGGSGFSATSNRGLGGSGSSVKFVS
TTSSSQKSYTH
>sp|P02768|ALBU_HUMAN Albumin OS=Homo sapiens OX=9606 GN=ALB PE=1 SV=2
MKWVTFISLLFLFSSAYSRGVFRRDAHKSEVAHRFKDLGEENFKALVLIAFAQYLQQCPF
EDHVKLVNEVTEFAKTCVADESAENCDKSLHTLFGDKLCTVATLRETYGEMADCCAKQEP
ERNECFLQHKDDNPNLPRLVRPEVDVMCTAFHDNEETFLKKYLYEIARRHPYFYAPELLF
FAKRYKAAFTECCQAADKAACLLPKLDELRDEGKASSAKQRLKCASLQKFGERAFKAWAV
ARLSQRFPKAEFAEVSKLVTDLTKVHTECCHGDLLECADDRADLAKYICENQDSISSKLK
ECCEKPLLEKSHCIAEVENDEMPADLPSLAADFVESKDVCKNYAEAKDVFLGMFLYEYAR
RHPDYSVVLLLRLAKTYETTLEKCCAAADPHECYAKVFDEFKPLVEEPQNLIKQNCELFE
QLGEYKFQNALLVRYTKKVPQVSTPTLVEVSRNLGKVGSKCCKHPEAKRMPCAEDYLSVV
LNQLCVLHEKTPVSDRVTKCCTESLVNRRPCFSALEVDETYVPKEFNAETFTFHADICTL
SEKERQIKKQTALVELVKHKPKATKEQLKAVMDDFAAFVEKCCKADDKETCFAEEGKKLV
AASQAALGL
>sp|P07355|ANXA2_HUMAN Annexin A2 OS=Homo sapiens OX=9606 GN=ANXA2 PE=1 SV=2
MSTVHEILCKLSLEGDHSTPPSAYGSVKAYTNFDAERDALNIETAIKTKGVDEVTIVNIL
TNRSNAQRQDIAFAYQRRTKKELASALKSALSGHLETVILGLLKTPAQYDASELKASMKG
LGTDEDSLIEIICSRTNQELQEINRVYKEMYKTDLEKDIISDTSGDFRKLMVALAKGRRA
EDGSVIDYELIDQDARDLYDAGVKRKGTDVPKWISIMTERSVPHLQKVFDRYKSYSPYDM
LESIRKEVKGDLENAFLNLVQCIQNKPLYFADRLYDSMKGKGTRDKVLIRIMVSRSEVDM
LKIRSEFKRKYGKSLYYYIQQDTKGDYQKALLYLCGGDD
>sp|A6NMY6|AXA2L_HUMAN Putative annexin A2-like protein OS=Homo sapiens OX=9606 GN=ANXA2P2 PE=5 SV=2
MSTVHEILCKLSLEGDHSTPPSAYGSVKAYTNFDAERDALNIETAIKTKGVDEVTIVNIV
TNRDNAQRQDIVFSYQRRTKKELASALKSALSGHLETVILGLLKTPAQYDASELKASMKG
LGTDEDSLIEIICSRTNQELQEINRVYKEMYKTDLEKDIISDTSGDFRKLMVALAKGRRA
EDGSVIDYELIDQDAQDLYDAGVKRKGTDVPKWISIMTERSVPHLQKVFDRYKSYSPYDM
LESIRKEVKGDLENAFLNLVQRIQNKPLYFADQLYDSMKGKGTRDKVLIRIMVSRSEVDM
LKIRSEFKRKYGKSLYYYIQQDTKGDYQKALLYLCGGDD
>sp|Q01469|FABP5_HUMAN Fatty acid-binding protein 5 OS=Homo sapiens OX=9606 GN=FABP5 PE=1 SV=3
MATVQQLEGRWRLVDSKGFDEYMKELGVGIALRKMGAMAKPDCIITCDGKNLTIKTESTL

KTTQFSCTLGEKFEETTADGRKTQTVCNFTDGALVQHQEWDGKESTITRKLKDGKLVVEC
VMNNVTCTRIYEKVE
>sp|A8MUU1|FB5L3_HUMAN Putative fatty acid-binding protein 5-like protein 3 OS=Homo sapiens OX=9606 GN=FABP5P3 PE=5 SV=1
MGAMAKPDCIITCDSKNLTIKTESTLKTTQFSGTLGEKFEENTADGRRTQTVCNFTDGAL
VQHQEWDGKESTITRKLKDGKLVVERVMNHVACTRIYEKAQ
>sp|P04406|G3P_HUMAN Glyceraldehyde-3-phosphate dehydrogenase OS=Homo sapiens OX=9606 GN=GAPDH PE=1 SV=3
MGKVKVGVNGFGRIGRLVTRAAFNSGKVDIVAINDPFIDLNYMVYMFQYDSTHGKFHGTV
KAENGKLVINGNPITIFQERDPSKIKWGDAGAEYVVESTGVFTTMEKAGAHLQGGAKRVI
ISAPSADAPMFVMGVNHEKYDNSLKIISNASCTTNCLAPLAKVIHDNFGIVEGLMTTVHA
ITATQKTVDGPSGKLWRDGRGALQNIIPASTGAAKAVGKVIPELNGKLTGMAFRVPTANV
SVVDLTCRLEKPAKYDDIKKVVKQASEGPLKGILGYTEHQVVSSDFNSDTHSSTFDAGAG
IALNDHFVKLISWYDNEFGYSNRVVDLMAHMASKE
>sp|O14556|G3PT_HUMAN Glyceraldehyde-3-phosphate dehydrogenase, testis-specific OS=Homo sapiens OX=9606 GN=GAPDHS PE=1 SV=2
MSKRDIVLTNVTVVQLLRQPCPVTRAPPPPEPKAEVEPQPQPEPTPVREEIKPPPPPLPP
HPATPPPKMVSVARELTVGINGFGRIGRLVLRACMEKGVKVVAVNDPFIDPEYMVYMFKY
DSTHGRYKGSVEFRNGQLVVDNHEISVYQCKEPKQIPWRAVGSPYVVESTGVYLSIQAAS
DHISAGAQRVVISAPSPDAPMFVMGVNENDYNPGSMNIVSNASCTTNCLAPLAKVIHERF
GIVEGLMTTVHSYTATQKTVDGPSRKAWRDGRGAHQNIIPASTGAAKAVTKVIPELKGKL
TGMAFRVPTPDVSVVDLTCRLAQPAPYSAIKEAVKAAAKGPMAGILAYTEDEVVSTDFLG
DTHSSIFDAKAGIALNDNFVKLISWYDNEYGYSHRVVDLLRYMFSRDK
>sp|P11142|HSP7C_HUMAN Heat shock cognate 71 kDa protein OS=Homo sapiens OX=9606 GN=HSPA8 PE=1 SV=1
MSKGPAVGIDLGTTYSCVGVFQHGKVEIIANDQGNRTTPSYVAFTDTERLIGDAAKNQVA
MNPTNTVFDAKRLIGRRFDDAVVQSDMKHWPFMVVNDAGRPKVQVEYKGETKSFYPEEVS
SMVLTKMKEIAEAYLGKTVTNAVVTVPAYFNDSQRQATKDAGTIAGLNVLRIINEPTAAA
IAYGLDKKVGAERNVLIFDLGGGTFDVSILTIEDGIFEVKSTAGDTHLGGEDFDNRMVNH
FIAEFKRKHKKDISENKRAVRRLRTACERAKRTLSSSTQASIEIDSLYEGIDFYTSITRA
RFEELNADLFRGTLDPVEKALRDAKLDKSQIHDIVLVGGSTRIPKIQKLLQDFFNGKELN
KSINPDEAVAYGAAVQAAILSGDKSENVQDLLLLDVTPLSLGIETAGGVMTVLIKRNTTI
PTKQTQTFTTYSDNQPGVLIQVYEGERAMTKDNNLLGKFELTGIPPAPRGVPQIEVTFDI
DANGILNVSAVDKSTGKENKITITNDKGRLSKEDIERMVQEAEKYKAEDEKQRDKVSSKN
SLESYAFNMKATVEDEKLQGKINDEDKQKILDKCNEIINWLDKNQTAEKEEFEHQQKELE
KVCNPIITKLYQSAGGMPGGMPGGFPGGGAPPSGGASSGPTIEEVD
>sp|P60709|ACTB_HUMAN Actin, cytoplasmic 1 OS=Homo sapiens OX=9606 GN=ACTB PE=1 SV=1
MDDDIAALVVDNGSGMCKAGFAGDDAPRAVFPSIVGRPRHQGVMVGMGQKDSYVGDEAQS
KRGILTLKYPIEHGIVTNWDDMEKIWHHTFYNELRVAPEEHPVLLTEAPLNPKANREKMT
QIMFETFNTPAMYVAIQAVLSLYASGRTTGIVMDSGDGVTHTVPIYEGYALPHAILRLDL
AGRDLTDYLMKILTERGYSFTTTAEREIVRDIKEKLCYVALDFEQEMATAASSSSLEKSY
ELPDGQVITIGNERFRCPEALFQPSFLGMESCGIHETTFNSIMKCDVDIRKDLYANTVLS
GGTTMYPGIADRMQKEITALAPSTMKIKIIAPPERKYSVWIGGSILASLSTFQQMWISKQ
EYDESGPSIVHRKCF
>sp|Q5T749|KPRP_HUMAN Keratinocyte proline-rich protein OS=Homo sapiens OX=9606 GN=KPRP PE=1 SV=1

MCDQQQIQCRLPLQQCCVKGPSFCSSQSPFAQSQVVVQAPCEMQIVDCPASCPVQVCQVS
DQAPCQSQTTQVKCQSKTKQVKGQAQCQSKTTQVKGQAASQSQTSSVQSQAPCQSEVSYV
QCEASQPVQTCFVECAPVCYTETCYVECPVQNYVPCPAPQPVQMYRGRPAVCQPQGRFST
QCQYQGSYSSCGPQFQSRATCNNYTPQFQLRPSYSSCFPQYRSRTSFSPCVPQCQTQGSY
GSFTEQHRSRSTSRCLPPPRRLQLFPRSCSPPRRFEPCSSSYLPLRPSEGFPNYCTPPRR
SEPIYNSRCPRRPISSCSQRRGPKCRIEISSPCCPRQVPPQRCPVEIPPIRRRSQSCGPQ
PSWGASCPELRPHVEPRPLPSFCPPRRLDQCPESPLQRCPPPAPRPRLRPEPCISLEPRP
RPLPRQLSEPCLYPEPLPALRPTPRPVPLPRPGQCEIPEPRPCLQPCEHPEPCPRPEPIP
LPAPCPSPEPCRETWRSPSPCWGPNPVPYPGDLGCHESSPHRLDTEAPYCGPSSYNQGQE
SGAGCGPGDVFPERRGQDGHGDQGNAFAGVKGEAKSAYF
>sp|P63261|ACTG_HUMAN Actin, cytoplasmic 2 OS=Homo sapiens OX=9606 GN=ACTG1 PE=1 SV=1
MEEEIAALVIDNGSGMCKAGFAGDDAPRAVFPSIVGRPRHQGVMVGMGQKDSYVGDEAQS
KRGILTLKYPIEHGIVTNWDDMEKIWHHTFYNELRVAPEEHPVLLTEAPLNPKANREKMT
QIMFETFNTPAMYVAIQAVLSLYASGRTTGIVMDSGDGVTHTVPIYEGYALPHAILRLDL
AGRDLTDYLMKILTERGYSFTTTAEREIVRDIKEKLCYVALDFEQEMATAASSSSLEKSY
ELPDGQVITIGNERFRCPEALFQPSFLGMESCGIHETTFNSIMKCDVDIRKDLYANTVLS
GGTTMYPGIADRMQKEITALAPSTMKIKIIAPPERKYSVWIGGSILASLSTFQQMWISKQ
EYDESGPSIVHRKCF
>sp|Q9NZT1|CALL5_HUMAN Calmodulin-like protein 5 OS=Homo sapiens OX=9606 GN=CALML5 PE=1 SV=2
MAGELTPEEEAQYKKAFSAVDTDGNGTINAQELGAALKATGKNLSEAQLRKLISEVDSDG
DGEISFQEFLTAAKKARAGLEDLQVAFRAFDQDGDGHITVDELRRAMAGLGQPLPQEELD
AMIREADVDQDGRVNYEEFARMLAQE
>sp|Q13835|PKP1_HUMAN Plakophilin-1 OS=Homo sapiens OX=9606 GN=PKP1 PE=1 SV=2
MNHSPLKTALAYECFQDQDNSTLALPSDQKMKTGTSGRQRVQEQVMMTVKRQKSKSSQSS
TLSHSNRGSMYDGLADNYNYGTTSRSSYYSKFQAGNGSWGYPIYNGTLKREPDNRRFSSY
SQMENWSRHYPRGSCNTTGAGSDICFMQKIKASRSEPDLYCDPRGTLRKGTLGSKGQKTT
QNRYSFYSTCSGQKAIKKCPVRPPSCASKQDPVYIPPISCNKDLSFGHSRASSKICSEDI
ECSGLTIPKAVQYLSSQDEKYQAIGAYYIQHTCFQDESAKQQVYQLGGICKLVDLLRSPN
QNVQQAAAGALRNLVFRSTTNKLETRRQNGIREAVSLLRRTGNAEIQKQLTGLLWNLSST
DELKEELIADALPVLADRVIIPFSGWCDGNSNMSREVVDPEVFFNATGCLRKRLGMRELL
ALVPQRATSSRVNLSSADAGRQTMRNYSGLIDSLMAYVQNCVAASRCDDKSVENCMCVLH
NLSYRLDAEVPTRYRQLEYNARNAYTEKSSTGCFSNKSDKMMNNNYDCPLPEEETNPKGS
GWLYHSDAIRTYLNLMGKSKKDATLEACAGALQNLTASKGLMSSGMSQLIGLKEKGLPQI
ARLLQSGNSDVVRSGASLLSNMSRHPLLHRVMGNQVFPEVTRLLTSHTGNTSNSEDILSS
ACYTVRNLMASQPQLAKQYFSSSMLNNIINLCRSSASPKAAEAARLLLSDMWSSKELQGV
LRQQGFDRNMLGTLAGANSLRNFTSRF
>sp|Q9H4B8|DPEP3_HUMAN Dipeptidase 3 OS=Homo sapiens OX=9606 GN=DPEP3 PE=1 SV=2
MQPTGREGSRALSRRYLRRLLLLLLLLLLRQPVTRAETTPGAPRALSTLGSPSLFTTPGV
PSALTTPGLTTPGTPKTLDLRGRAQALMRSFPLVDGHNDLPQVLRQRYKNVLQDVNLRNF
SHGQTSLDRLRDGLVGAQFWSASVSCQSQDQTAVRLALEQIDLIHRMCASYSELELVTSA
EGLNSSQKLACLIGVEGGHSLDSSLSVLRSFYVLGVRYLTLTFTCSTPWAESSTKFRHHM
YTNVSGLTSFGEKVVEELNRLGMMIDLSYASDTLIRRVLEVSQAPVIFSHSAARAVCDNL
LNVPDDILQLLKKNGGIVMVTLSMGVLQCNLLANVSTVADHFDHIRAVIGSEFIGIGGNY

DGTGRFPQGLEDVSTYPVLIEELLSRSWSEEELQGVLRGNLLRVFRQVEKVREESRAQSP
VEAEFPYGQLSTSCHSHLVPQNGHQATHLEVTKQPTNRVPWRSSNASPYLVPGLVAAATI
PTFTQWLC
>sp|Q9H4A9|DPEP2_HUMAN Dipeptidase 2 OS=Homo sapiens OX=9606 GN=DPEP2 PE=1 SV=3
MQPSGLEGPGTFGRWPLLSLLLLLLLLQPVTCAYTTPGPPRALTTLGAPRAHTMPGTYAP
STTLSSPSTQGLQEQARALMRDFPLVDGHNDLPLVLRQVYQKGLQDVNLRNFSYGQTSLD
RLRDGLVGAQFWSAYVPCQTQDRDALRLTLEQIDLIRRMCASYSELELVTSAKALNDTQK
LACLIGVEGGHSLDNSLSILRTFYMLGVRYLTLTHTCNTPWAESSAKGVHSFYNNISGLT
DFGEKVVAEMNRLGMMVDLSHVSDAVARRALEVSQAPVIFSHSAARGVCNSARNVPDDIL
QLLKKNGGVVMVSLSMGVIQCNPSANVSTVADHFDHIKAVIGSKFIGIGGDYDGAGKFPQ
GLEDVSTYPVLIEELLSRGWSEEELQGVLRGNLLRVFRQVEKVQEENKWQSPLEDKFPDE
QLSSSCHSDLSRLRQRQSLTSGQELTEIPIHWTAKLPAKWSVSESSPHMAPVLAVVATFP
VLILWL
>sp|P35579|MYH9_HUMAN Myosin-9 OS=Homo sapiens OX=9606 GN=MYH9 PE=1 SV=4
MAQQAADKYLYVDKNFINNPLAQADWAAKKLVWVPSDKSGFEPASLKEEVGEEAIVELVE
NGKKVKVNKDDIQKMNPPKFSKVEDMAELTCLNEASVLHNLKERYYSGLIYTYSGLFCVV
INPYKNLPIYSEEIVEMYKGKKRHEMPPHIYAITDTAYRSMMQDREDQSILCTGESGAGK
TENTKKVIQYLAYVASSHKSKKDQGELERQLLQANPILEAFGNAKTVKNDNSSRFGKFIR
INFDVNGYIVGANIETYLLEKSRAIRQAKEERTFHIFYYLLSGAGEHLKTDLLLEPYNKY
RFLSNGHVTIPGQQDKDMFQETMEAMRIMGIPEEEQMGLLRVISGVLQLGNIVFKKERNT
DQASMPDNTAAQKVSHLLGINVTDFTRGILTPRIKVGRDYVQKAQTKEQADFAIEALAKA
TYERMFRWLVLRINKALDKTKRQGASFIGILDIAGFEIFDLNSFEQLCINYTNEKLQQLF
NHTMFILEQEEYQREGIEWNFIDFGLDLQPCIDLIEKPAGPPGILALLDEECWFPKATDK
SFVEKVMQEQGTHPKFQKPKQLKDKADFCIIHYAGKVDYKADEWLMKNMDPLNDNIATLL
HQSSDKFVSELWKDVDRIIGLDQVAGMSETALPGAFKTRKGMFRTVGQLYKEQLAKLMAT
LRNTNPNFVRCIIPNHEKKAGKLDPHLVLDQLRCNGVLEGIRICRQGFPNRVVFQEFRQR
YEILTPNSIPKGFMDGKQACVLMIKALELDSNLYRIGQSKVFFRAGVLAHLEEERDLKIT
DVIIGFQACCRGYLARKAFAKRQQQLTAMKVLQRNCAAYLKLRNWQWWRLFTKVKPLLQV
SRQEEEMMAKEEELVKVREKQLAAENRLTEMETLQSQLMAEKLQLQEQLQAETELCAEAE
ELRARLTAKKQELEEICHDLEARVEEEEERCQHLQAEKKKMQQNIQELEEQLEEEESARQ
KLQLEKVTTEAKLKKLEEEQIILEDQNCKLAKEKKLLEDRIAEFTTNLTEEEEKSKSLAK
LKNKHEAMITDLEERLRREEKQRQELEKTRRKLEGDSTDLSDQIAELQAQIAELKMQLAK
KEEELQAALARVEEEAAQKNMALKKIRELESQISELQEDLESERASRNKAEKQKRDLGEE
LEALKTELEDTLDSTAAQQELRSKREQEVNILKKTLEEEAKTHEAQIQEMRQKHSQAVEE
LAEQLEQTKRVKANLEKAKQTLENERGELANEVKVLLQGKGDSEHKRKKVEAQLQELQVK
FNEGERVRTELADKVTKLQVELDNVTGLLSQSDSKSSKLTKDFSALESQLQDTQELLQEE
NRQKLSLSTKLKQVEDEKNSFREQLEEEEEAKHNLEKQIATLHAQVADMKKKMEDSVGCL
ETAEEVKRKLQKDLEGLSQRHEEKVAAYDKLEKTKTRLQQELDDLLVDLDHQRQSACNLE
KKQKKFDQLLAEEKTISAKYAEERDRAEAEAREKETKALSLARALEEAMEQKAELERLNK
QFRTEMEDLMSSKDDVGKSVHELEKSKRALEQQVEEMKTQLEELEDELQATEDAKLRLEV
NLQAMKAQFERDLQGRDEQSEEKKKQLVRQVREMEAELEDERKQRSMAVAARKKLEMDLK
DLEAHIDSANKNRDEAIKQLRKLQAQMKDCMRELDDTRASREEILAQAKENEKKLKSMEA
EMIQLQEELAAAERAKRQAQQERDELADEIANSSGKGALALEEKRRLEARIAQLEEELEE
EQGNTELINDRLKKANLQIDQINTDLNLERSHAQKNENARQQLERQNKELKVKLQEMEGT
VKSKYKASITALEAKIAQLEEQLDNETKERQAACKQVRRTEKKLKDVLLQVDDERRNAEQ

YKDQADKASTRLKQLKRQLEEAEEEAQRANASRRKLQRELEDATETADAMNREVSSLKNK
LRRGDLPFVVPRRMARKGAGDGSDEEVDGKADGAEAKPAE
>sp|P35580|MYH10_HUMAN Myosin-10 OS=Homo sapiens OX=9606 GN=MYH10 PE=1 SV=3
MAQRTGLEDPERYLFVDRAVIYNPATQADWTAKKLVWIPSERHGFEAASIKEERGDEVMV
ELAENGKKAMVNKDDIQKMNPPKFSKVEDMAELTCLNEASVLHNLKDRYYSGLIYTYSGL
FCVVINPYKNLPIYSENIIEMYRGKKRHEMPPHIYAISESAYRCMLQDREDQSILCTGES
GAGKTENTKKVIQYLAHVASSHKGRKDHNIPGELERQLLQANPILESFGNAKTVKNDNSS
RFGKFIRINFDVTGYIVGANIETYLLEKSRAVRQAKDERTFHIFYQLLSGAGEHLKSDLL
LEGFNNYRFLSNGYIPIPGQQDKDNFQETMEAMHIMGFSHEEILSMLKVVSSVLQFGNIS
FKKERNTDQASMPENTVAQKLCHLLGMNVMEFTRAILTPRIKVGRDYVQKAQTKEQADFA
VEALAKATYERLFRWLVHRINKALDRTKRQGASFIGILDIAGFEIFELNSFEQLCINYTN
EKLQQLFNHTMFILEQEEYQREGIEWNFIDFGLDLQPCIDLIERPANPPGVLALLDEECW
FPKATDKTFVEKLVQEQGSHSKFQKPRQLKDKADFCIIHYAGKVDYKADEWLMKNMDPLN
DNVATLLHQSSDRFVAELWKDVDRIVGLDQVTGMTETAFGSAYKTKKGMFRTVGQLYKES
LTKLMATLRNTNPNFVRCIIPNHEKRAGKLDPHLVLDQLRCNGVLEGIRICRQGFPNRIV
FQEFRQRYEILTPNAIPKGFMDGKQACERMIRALELDPNLYRIGQSKIFFRAGVLAHLEE
ERDLKITDIIIFFQAVCRGYLARKAFAKKQQQLSALKVLQRNCAAYLKLRHWQWWRVFTK
VKPLLQVTRQEEELQAKDEELLKVKEKQTKVEGELEEMERKHQQLLEEKNILAEQLQAET
ELFAEAEEMRARLAAKKQELEEILHDLESRVEEEEERNQILQNEKKKMQAHIQDLEEQLD
EEEGARQKLQLEKVTAEAKIKKMEEEILLLEDQNSKFIKEKKLMEDRIAECSSQLAEEEE
KAKNLAKIRNKQEVMISDLEERLKKEEKTRQELEKAKRKLDGETTDLQDQIAELQAQIDE
LKLQLAKKEEELQGALARGDDETLHKNNALKVVRELQAQIAELQEDFESEKASRNKAEKQ
KRDLSEELEALKTELEDTLDTTAAQQELRTKREQEVAELKKALEEETKNHEAQIQDMRQR
HATALEELSEQLEQAKRFKANLEKNKQGLETDNKELACEVKVLQQVKAESEHKRKKLDAQ
VQELHAKVSEGDRLRVELAEKASKLQNELDNVSTLLEEAEKKGIKFAKDAASLESQLQDT
QELLQEETRQKLNLSSRIRQLEEEKNSLQEQQEEEEEARKNLEKQVLALQSQLADTKKKV
DDDLGTIESLEEAKKKLLKDAEALSQRLEEKALAYDKLEKTKNRLQQELDDLTVDLDHQR
QVASNLEKKQKKFDQLLAEEKSISARYAEERDRAEAEAREKETKALSLARALEEALEAKE
EFERQNKQLRADMEDLMSSKDDVGKNVHELEKSKRALEQQVEEMRTQLEELEDELQATED
AKLRLEVNMQAMKAQFERDLQTRDEQNEEKKRLLIKQVRELEAELEDERKQRALAVASKK
KMEIDLKDLEAQIEAANKARDEVIKQLRKLQAQMKDYQRELEEARASRDEIFAQSKESEK
KLKSLEAEILQLQEELASSERARRHAEQERDELADEITNSASGKSALLDEKRRLEARIAQ
LEEELEEEQSNMELLNDRFRKTTLQVDTLNAELAAERSAAQKSDNARQQLERQNKELKAK
LQELEGAVKSKFKATISALEAKIGQLEEQLEQEAKERAAANKLVRRTEKKLKEIFMQVED
ERRHADQYKEQMEKANARMKQLKRQLEEAEEEATRANASRRKLQRELDDATEANEGLSRE
VSTLKNRLRRGGPISFSSSRSGRRQLHLEGASLELSDDDTESKTSDVNETQPPQSE
>sp|P20930|FILA_HUMAN Filaggrin OS=Homo sapiens OX=9606 GN=FLG PE=1 SV=3
MSTLLENIFAIINLFKQYSKKDKNTDTLSKKELKELLEKEFRQILKNPDDPDMVDVFMDH
LDIDHNKKIDFTEFLLMVFKLAQAYYESTRKENLPISGHKHRKHSHHDKHEDNKQEENKE
NRKRPSSLERRNNRKGNKGRSKSPRETGGKRHESSSEKKERKGYSPTHREEEYGKNHHNS
SKKEKNKTENTRLGDNRKRLSERLEEKEDNEEGVYDYENTGRMTQKWIQSGHIATYYTIQ
DEAYDTTDSLLEENKIYERSRSSDGKSSSQVNRSRHENTSQVPLQESRTRKRRGSRVSQD
RDSEGHSEDSERHSGSASRNHHGSAWEQSRDGSRHPRSHDEDRASHGHSADSSRQSGTRH
AETSSRGQTASSHEQARSSPGERHGSGHQQSADSSRHSATGRGQASSAVSDRGHRGSSGS
QASDSEGHSENSDTQSVSGHGKAGLRQQSHQESTRGRSGERSGRSGSSLYQVSTHEQPDS

AHGRTGTSTGGRQGSHHEQARDSSRHSASQEGQDTIRGHPGSSRGGRQGSHHEQSVNRSG
HSGSHHSHTTSQGRSDASHGQSGSRSASRQTRNEEQSGDGTRHSGSRHHEASSQADSSRH
SQVGQGQSSGPRTSRNQGSSVSQDSDSQGHSEDSERWSGSASRNHHGSAQEQSRDGSRHP
RSHHEDRAGHGHSADSSRKSGTRHTQNSSSGQAASSHEQARSSAGERHGSRHQLQSADSS
RHSGTGHGQASSAVRDSGHRGSSGSQATDSEGHSEDSDTQSVSGHGQAGHHQQSHQESAR
DRSGERSRRSGSFLYQVSTHKQSESSHGWTGPSTGVRQGSHHEQARDNSRHSASQDGQDT
IRGHPGSSRRGRQGSHHEQSVDRSGHSGSHHSHTTSQGRSDASRGQSGSRSASRTTRNEE
QSRDGSRHSGSRHHEASSHADISRHSQAGQGQSEGSRTSRRQGSSVSQDSDSEGHSEDSE
RWSGSASRNHRGSAQEQSRHGSRHPRSHHEDRAGHGHSADSSRQSGTPHAETSSGGQAAS
SHEQARSSPGERHGSRHQQSADSSRHSGIPRRQASSAVRDSGHWGSSGSQASDSEGHSEE
SDTQSVSGHGQDGPHQQSHQESARDWSGGRSGRSGSFIYQVSTHEQSESAHGRTRTSTGR
RQGSHHEQARDSSRHSASQEGQDTIRAHPGSRRGGRQGSHHEQSVDRSGHSGSHHSHTTS
QGRSDASHGQSGSRSASRQTRKDKQSGDGSRHSGSRHHEAASWADSSRHSQVGQEQSSGS
RTSRHQGSSVSQDSDSERHSDDSERLSGSASRNHHGSSREQSRDGSRHPGFHQEDRASHG
HSADSSRQSGTHHTESSSHGQAVSSHEQARSSPGERHGSRHQQSADSSRHSGIGHRQASS
AVRDSGHRGSSGSQVTNSEGHSEDSDTQSVSAHGQAGPHQQSHKESARGQSGESSGRSRS
FLYQVSSHEQSESTHGQTAPSTGGRQGSRHEQARNSSRHSASQDGQDTIRGHPGSSRGGR
QGSYHEQSVDRSGHSGYHHSHTTPQGRSDASHGQSGPRSASRQTRNEEQSGDGSRHSGSR
HHEPSTRAGSSRHSQVGQGESAGSKTSRRQGSSVSQDRDSEGHSEDSERRSESASRNHYG
SAREQSRHGSRNPRSHQEDRASHGHSAESSRQSGTRHAETSSGGQAASSQEQARSSPGER
HGSRHQQSADSSTDSGTGRRQDSSVVGDSGNRGSSGSQASDSEGHSEESDTQSVSAHGQA
GPHQQSHQESTRGQSGERSGRSGSFLYQVSTHEQSESAHGRTGPSTGGRQRSRHEQARDS
SRHSASQEGQDTIRGHPGSSRGGRQGSHYEQSVDSSGHSGSHHSHTTSQERSDVSRGQSG
SRSVSRQTRNEKQSGDGSRHSGSRHHEASSRADSSRHSQVGQGQSSGPRTSRNQGSSVSQ
DSDSQGHSEDSERWSGSASRNHLGSAWEQSRDGSRHPGSHHEDRAGHGHSADSSRQSGTR
HTESSSRGQAASSHEQARSSAGERHGSHHQLQSADSSRHSGIGHGQASSAVRDSGHRGYS
GSQASDSEGHSEDSDTQSVSAQGKAGPHQQSHKESARGQSGESSGRSGSFLYQVSTHEQS
ESTHGQSAPSTGGRQGSHYDQAQDSSRHSASQEGQDTIRGHPGPSRGGRQGSHQEQSVDR
SGHSGSHHSHTTSQGRSDASRGQSGSRSASRKTYDKEQSGDGSRHSGSHHHEASSWADSS
RHSLVGQGQSSGPRTSRPRGSSVSQDSDSEGHSEDSERRSGSASRNHHGSAQEQSRDGSR
HPRSHHEDRAGHGHSAESSRQSGTHHAENSSGGQAASSHEQARSSAGERHGSHHQQSADS
SRHSGIGHGQASSAVRDSGHRGSSGSQASDSEGHSEDSDTQSVSAHGQAGPHQQSHQEST
RGRSAGRSGRSGSFLYQVSTHEQSESAHGRTGTSTGGRQGSHHKQARDSSRHSTSQEGQD
TIHGHPGSSSGGRQGSHYEQLVDRSGHSGSHHSHTTSQGRSDASHGHSGSRSASRQTRND
EQSGDGSRHSGSRHHEASSRADSSGHSQVGQGQSEGPRTSRNWGSSFSQDSDSQGHSEDS
ERWSGSASRNHHGSAQEQLRDGSRHPRSHQEDRAGHGHSADSSRQSGTRHTQTSSGGQAA
SSHEQARSSAGERHGSHHQQSADSSRHSGIGHGQASSAVRDSGHRGYSGSQASDNEGHSE
DSDTQSVSAHGQAGSHQQSHQESARGRSGETSGHSGSFLYQVSTHEQSESSHGWTGPSTR
GRQGSRHEQAQDSSRHSASQDGQDTIRGHPGSSRGGRQGYHHEHSVDSSGHSGSHHSHTT
SQGRSDASRGQSGSRSASRTTRNEEQSGDGSRHSGSRHHEASTHADISRHSQAVQGQSEG
SRRSRRQGSSVSQDSDSEGHSEDSERWSGSASRNHHGSAQEQLRDGSRHPRSHQEDRAGH
GHSADSSRQSGTRHTQTSSGGQAASSHEQARSSAGERHGSHHQQSADSSRHSGIGHGQAS
SAVRDSGHRGYSGSQASDNEGHSEDSDTQSVSAHGQAGSHQQSHQESARGRSGETSGHSG
SFLYQVSTHEQSESSHGWTGPSTRGRQGSRHEQAQDSSRHSASQYGQDTIRGHPGSSRGG
RQGYHHEHSVDSSGHSGSHHSHTTSQGRSDASRGQSGSRSASRTTRNEEQSGDSSRHSVS
RHHEASTHADISRHSQAVQGQSEGSRRSRRQGSSVSQDSDSEGHSEDSERWSGSASRNHR
GSVQEQSRHGSRHPRSHHEDRAGHGHSADRSRQSGTRHAETSSGGQAASSHEQARSSPGE

RHGSRHQQSADSSRHSGIPRGQASSAVRDSRHWGSSGSQASDSEGHSEESDTQSVSGHGQ
AGPHQQSHQESARDRSGGRSGRSGSFLYQVSTHEQSESAHGRTRTSTGRRQGSHHEQARD
SSRHSASQEGQDTIRGHPGSSRRGRQGSHYEQSVDRSGHSGSHHSHTTSQGRSDASRGQS
GSRSASRQTRNDEQSGDGSRHSWSHHHEASTQADSSRHSQSGQGQSAGPRTSRNQGSSVS
QDSDSQGHSEDSERWSGSASRNHRGSAQEQSRDGSRHPTSHHEDRAGHGHSAESSRQSGT
HHAENSSGGQAASSHEQARSSAGERHGSHHQQSADSSRHSGIGHGQASSAVRDSGHRGSS
GSQASDSEGHSEDSDTQSVSAHGQAGPHQQSHQESTRGRSAGRSGRSGSFLYQVSTHEQS
ESAHGRAGPSTGGRQGSRHEQARDSSRHSASQEGQDTIRGHPGSRRGGRQGSYHEQSVDR
SGHSGSHHSHTTSQGRSDASHGQSGSRSASRETRNEEQSGDGSRHSGSRHHEASTQADSS
RHSQSGQGESAGSRRSRRQGSSVSQDSDSEAYPEDSERRSESASRNHHGSSREQSRDGSR
HPGSSHRDTASHVQSSPVQSDSSTAKEHGHFSSLSQDSAYHSGIQSRGSPHSSSSYHYQS
EGTERQKGQSGLVWRHGSYGSADYDYGESGFRHSQHGSVSYNSNPVVFKERSDICKASAF
GKDHPRYYATYINKDPGLCGHSSDISKQLGFSQSQRYYYYE
>sp|Q8N1N4|K2C78_HUMAN Keratin, type II cytoskeletal 78 OS=Homo sapiens OX=9606 GN=KRT78 PE=1 SV=2
MSLSPCRAQRGFSARSACSARSRGRSRGGFSSRGGFSSRSLNSFGGCLEGSRGSTWGSGG
RLGVRFGEWSGGPGLSLCPPGGIQEVTINQNLLTPLKIEIDPQFQVVRTQETQEIRTLNN
QFASFIDKVRFLEQQNKVLETKWHLLQQQGLSGSQQGLEPVFEACLDQLRKQLEQLQGER
GALDAELKACRDQEEEYKSKYEEEAHRRATLENDFVVLKKDVDGVFLSKMELEGKLEALR
EYLYFLKHLNEEELGQLQTQASDTSVVLSMDNNRYLDFSSIITEVRARYEEIARSSKAEA
EALYQTKYQELQVSAQLHGDRMQETKVQISQLHQEIQRLQSQTENLKKQNASLQAAITDA
EQRGELALKDAQAKVDELEAALRMAKQNLARLLCEYQELTSTKLSLDVEIATYRRLLEGE
ECRMSGECTSQVTISSVGGSAVMSGGVGGGLGSTCGLGSGKGSPGSCCTSIVTGGSNIIL
GSGKDPVLDSCSVSGSSAGSSCHTILKKTVESSLKTSITY
>sp|Q71U36|TBA1A_HUMAN Tubulin alpha-1A chain OS=Homo sapiens OX=9606 GN=TUBA1A PE=1 SV=1
MRECISIHVGQAGVQIGNACWELYCLEHGIQPDGQMPSDKTIGGGDDSFNTFFSETGAGK
HVPRAVFVDLEPTVIDEVRTGTYRQLFHPEQLITGKEDAANNYARGHYTIGKEIIDLVLD
RIRKLADQCTGLQGFLVFHSFGGGTGSGFTSLLMERLSVDYGKKSKLEFSIYPAPQVSTA
VVEPYNSILTTHTTLEHSDCAFMVDNEAIYDICRRNLDIERPTYTNLNRLIGQIVSSITA
SLRFDGALNVDLTEFQTNLVPYPRIHFPLATYAPVISAEKAYHEQLSVAEITNACFEPAN
QMVKCDPRHGKYMACCLLYRGDVVPKDVNAAIATIKTKRTIQFVDWCPTGFKVGINYQPP
TVVPGGDLAKVQRAVCMLSNTTAIAEAWARLDHKFDLMYAKRAFVHWYVGEGMEEGEFSE
AREDMAALEKDYEEVGVDSVEGEGEEEGEEY
>sp|P0DPH7|TBA3C_HUMAN Tubulin alpha-3C chain OS=Homo sapiens OX=9606 GN=TUBA3C PE=1 SV=1
MRECISIHVGQAGVQIGNACWELYCLEHGIQPDGQMPSDKTIGGGDDSFNTFFSETGAGK
HVPRAVFVDLEPTVVDEVRTGTYRQLFHPEQLITGKEDAANNYARGHYTIGKEIVDLVLD
RIRKLADLCTGLQGFLIFHSFGGGTGSGFASLLMERLSVDYGKKSKLEFAIYPAPQVSTA
VVEPYNSILTTHTTLEHSDCAFMVDNEAIYDICRRNLDIERPTYTNLNRLIGQIVSSITA
SLRFDGALNVDLTEFQTNLVPYPRIHFPLATYAPVISAEKAYHEQLSVAEITNACFEPAN
QMVKCDPRHGKYMACCMLYRGDVVPKDVNAAIATIKTKRTIQFVDWCPTGFKVGINYQPP
TVVPGGDLAKVQRAVCMLSNTTAIAEAWARLDHKFDLMYAKRAFVHWYVGEGMEEGEFSE
AREDLAALEKDYEEVGVDSVEAEAEEGEEY
>sp|P0DPH8|TBA3D_HUMAN Tubulin alpha-3D chain OS=Homo sapiens OX=9606 GN=TUBA3D PE=1 SV=1
MRECISIHVGQAGVQIGNACWELYCLEHGIQPDGQMPSDKTIGGGDDSFNTFFSETGAGK

HVPRAVFVDLEPTVVDEVRTGTYRQLFHPEQLITGKEDAANNYARGHYTIGKEIVDLVLD
RIRKLADLCTGLQGFLIFHSFGGGTGSGFASLLMERLSVDYGKKSKLEFAIYPAPQVSTA
VVEPYNSILTTHTTLEHSDCAFMVDNEAIYDICRRNLDIERPTYTNLNRLIGQIVSSITA
SLRFDGALNVDLTEFQTNLVPYPRIHFPLATYAPVISAEKAYHEQLSVAEITNACFEPAN
QMVKCDPRHGKYMACCMLYRGDVVPKDVNAAIATIKTKRTIQFVDWCPTGFKVGINYQPP
TVVPGGDLAKVQRAVCMLSNTTAIAEAWARLDHKFDLMYAKRAFVHWYVGEGMEEGEFSE
AREDLAALEKDYEEVGVDSVEAEAEEGEEY
>sp|Q6PEY2|TBA3E_HUMAN Tubulin alpha-3E chain OS=Homo sapiens OX=9606 GN=TUBA3E PE=1 SV=2
MRECISIHVGQAGVQIGNACWELYCLEHGIQPDGQMPSDKTIGGGDDSFNTFFSETGAGK
HVPRAVFVDLEPTVVDEVRTGTYRQLFHPEQLITGKEDAASNYARGHYTIGKEIVDLVLD
RIRKLADLCTGLQGFLIFHSFGGGTGSGFASLLMERLSVDYSKKSKLEFAIYPAPQVSTA
VVEPYNSILTTHTTLEHSDCAFMVDNEAIYDICRRNLDIERPTYTNLNRLIGQIVSSITA
SLRFDGALNVDLTEFQTNLVPYPRIHFPLATYAPVISAEKAYHEQLSVAEITNACFEPAN
QMVKCDPRHGKYMACCMLYRGDVVPKDVNAAIATIKTKRTIQFVDWCPTGFKVGINYQPP
TVVPGGDLAKVQRAVCMLSNTTAIAEAWARLVHKFDLMYAKWAFVHWYVGEGMEEGEFSE
AREDLAALEKDCEEVGVDSVEAEAEEGEAY
>sp|P47929|LEG7_HUMAN Galectin-7 OS=Homo sapiens OX=9606 GN=LGALS7B PE=1 SV=2
MSNVPHKSSLPEGIRPGTVLRIRGLVPPNASRFHVNLLCGEEQGSDAALHFNPRLDTSEV
VFNSKEQGSWGREERGPGVPFQRGQPFEVLIIASDDGFKAVVGDAQYHHFRHRLPLARVR
LVEVGGDVQLDSVRIF
>sp|P48594|SPB4_HUMAN Serpin B4 OS=Homo sapiens OX=9606 GN=SERPINB4 PE=1 SV=2
MNSLSEANTKFMFDLFQQFRKSKENNIFYSPISITSALGMVLLGAKDNTAQQISKVLHFD
QVTENTTEKAATYHVDRSGNVHHQFQKLLTEFNKSTDAYELKIANKLFGEKTYQFLQEYL
DAIKKFYQTSVESTDFANAPEESRKKINSWVESQTNEKIKNLFPDGTIGNDTTLVLVNAI
YFKGQWENKFKKENTKEEKFWPNKNTYKSVQMMRQYNSFNFALLEDVQAKVLEIPYKGKD
LSMIVLLPNEIDGLQKLEEKLTAEKLMEWTSLQNMRETCVDLHLPRFKMEESYDLKDTLR
TMGMVNIFNGDADLSGMTWSHGLSVSKVLHKAFVEVTEEGVEAAAATAVVVVELSSPSTN
EEFCCNHPFLFFIRQNKTNSILFYGRFSSP
>sp|Q15149|PLEC_HUMAN Plectin OS=Homo sapiens OX=9606 GN=PLEC PE=1 SV=3
MVAGMLMPRDQLRAIYEVLFREGVMVAKKDRRPRSLHPHVPGVTNLQVMRAMASLRARGL
VRETFAWCHFYWYLTNEGIAHLRQYLHLPPEIVPASLQRVRRPVAMVMPARRTPHVQAVQ
GPLGSPPKRGPLPTEEQRVYRRKELEEVSPETPVVPATTQRTLARPGPEPAPATDERDRV
QKKTFTKWVNKHLIKAQRHISDLYEDLRDGHNLISLLEVLSGDSLPREKGRMRFHKLQNV
QIALDYLRHRQVKLVNIRNDDIADGNPKLTLGLIWTIILHFQISDIQVSGQSEDMTAKEK
LLLWSQRMVEGYQGLRCDNFTSSWRDGRLFNAIIHRHKPLLIDMNKVYRQTNLENLDQAF
SVAERDLGVTRLLDPEDVDVPQPDEKSIITYVSSLYDAMPRVPDVQDGVRANELQLRWQE
YRELVLLLLQWMRHHTAAFEERRFPSSFEEIEILWSQFLKFKEMELPAKEADKNRSKGIY
QSLEGAVQAGQLKVPPGYHPLDVEKEWGKLHVAILEREKQLRSEFERLECLQRIVTKLQM
EAGLCEEQLNQADALLQSDVRLLAAGKVPQRAGEVERDLDKADSMIRLLFNDVQTLKDGR
HPQGEQMYRRVYRLHERLVAIRTEYNLRLKAGVAAPATQVAQVTLQSVQRRPELEDSTLR
YLQDLLAWVEENQHRVDGAEWGVDLPSVEAQLGSHRGLHQSIEEFRAKIERARSDEGQLS
PATRGAYRDCLGRLDLQYAKLLNSSKARLRSLESLHSFVAAATKELMWLNEKEEEEVGFD
WSDRNTNMTAKKESYSALMRELELKEKKIKELQNAGDRLLREDHPARPTVESFQAALQTQ

WSWMLQLCCCIEAHLKENAAYFQFFSDVREAEGQLQKLQEALRRKYSCDRSATVTRLEDL
LQDAQDEKEQLNEYKGHLSGLAKRAKAVVQLKPRHPAHPMRGRLPLLAVCDYKQVEVTVH
KGDECQLVGPAQPSHWKVLSSSGSEAAVPSVCFLVPPPNQEAQEAVTRLEAQHQALVTLW
HQLHVDMKSLLAWQSLRRDVQLIRSWSLATFRTLKPEEQRQALHSLELHYQAFLRDSQDA
GGFGPEDRLMAEREYGSCSHHYQQLLQSLEQGAQEESRCQRCISELKDIRLQLEACETRT
VHRLRLPLDKEPARECAQRIAEQQKAQAEVEGLGKGVARLSAEAEKVLALPEPSPAAPTL
RSELELTLGKLEQVRSLSAIYLEKLKTISLVIRGTQGAEEVLRAHEEQLKEAQAVPATLP
ELEATKASLKKLRAQAEAQQPTFDALRDELRGAQEVGERLQQRHGERDVEVERWRERVAQ
LLERWQAVLAQTDVRQRELEQLGRQLRYYRESADPLGAWLQDARRRQEQIQAMPLADSQA
VREQLRQEQALLEEIERHGEKVEECQRFAKQYINAIKDYELQLVTYKAQLEPVASPAKKP
KVQSGSESVIQEYVDLRTHYSELTTLTSQYIKFISETLRRMEEEERLAEQQRAEERERLA
EVEAALEKQRQLAEAHAQAKAQAEREAKELQQRMQEEVVRREEAAVDAQQQKRSIQEELQ
QLRQSSEAEIQAKARQAEAAERSRLRIEEEIRVVRLQLEATERQRGGAEGELQALRARAE
EAEAQKRQAQEEAERLRRQVQDESQRKRQAEVELASRVKAEAEAAREKQRALQALEELRL
QAEEAERRLRQAEVERARQVQVALETAQRSAEAELQSKRASFAEKTAQLERSLQEEHVAV
AQLREEAERRAQQQAEAERAREEAERELERWQLKANEALRLRLQAEEVAQQKSLAQAEAE
KQKEEAEREARRRGKAEEQAVRQRELAEQELEKQRQLAEGTAQQRLAAEQELIRLRAETE
QGEQQRQLLEEELARLQREAAAATQKRQELEAELAKVRAEMEVLLASKARAEEESRSTSE
KSKQRLEAEAGRFRELAEEAARLRALAEEAKRQRQLAEEDAARQRAEAERVLAEKLAAIG
EATRLKTEAEIALKEKEAENERLRRLAEDEAFQRRRLEEQAAQHKADIEERLAQLRKASD
SELERQKGLVEDTLRQRRQVEEEILALKASFEKAAAGKAELELELGRIRSNAEDTLRSKE
QAELEAARQRQLAAEEERRRREAEERVQKSLAAEEEAARQRKAALEEVERLKAKVEEARR
LRERAEQESARQLQLAQEAAQKRLQAEEKAHAFAVQQKEQELQQTLQQEQSVLDQLRGEA
EAARRAAEEAEEARVQAEREAAQSRRQVEEAERLKQSAEEQAQARAQAQAAAEKLRKEAE
QEAARRAQAEQAALRQKQAADAEMEKHKKFAEQTLRQKAQVEQELTTLRLQLEETDHQKN
LLDEELQRLKAEATEAARQRSQVEEELFSVRVQMEELSKLKARIEAENRALILRDKDNTQ
RFLQEEAEKMKQVAEEAARLSVAAQEAARLRQLAEEDLAQQRALAEKMLKEKMQAVQEAT
RLKAEAELLQQQKELAQEQARRLQEDKEQMAQQLAEETQGFQRTLEAERQRQLEMSAEAE
RLKLRVAEMSRAQARAEEDAQRFRKQAEEIGEKLHRTELATQEKVTLVQTLEIQRQQSDH
DAERLREAIAELEREKEKLQQEAKLLQLKSEEMQTVQQEQLLQETQALQQSFLSEKDSLL
QRERFIEQEKAKLEQLFQDEVAKAQQLREEQQRQQQMEQERQRLVASMEEARRRQHEAE
EGVRRKQEELQQLEQQRRQQEELLAEENQRLREQLQLLEEQHRAALAHSEEVTASQVAAT
KTLPNGRDALDGPAAEAEPEHSFDGLRRKVSAQRLQEAGILSAEELQRLAQGHTTVDELA
RREDVRHYLQGRSSIAGLLLKATNEKLSVYAALQRQLLSPGTALILLEAQAASGFLLDPV
RNRRLTVNEAVKEGVVGPELHHKLLSAERAVTGYKDPYTGQQISLFQAMQKGLIVREHGI
RLLEAQIATGGVIDPVHSHRVPVDVAYRRGYFDEEMNRVLADPSDDTKGFFDPNTHENLT
YLQLLERCVEDPETGLCLLPLTDKAAKGGELVYTDSEARDVFEKATVSAPFGKFQGKTVT
IWEIINSEYFTAEQRRDLLRQFRTGRITVEKIIKIIITVVEEQEQKGRLCFEGLRSLVPA
AELLESRVIDRELYQQLQRGERSVRDVAEVDTVRRALRGANVIAGVWLEEAGQKLSIYNA
LKKDLLPSDMAVALLEAQAGTGHIIDPATSARLTVDEAVRAGLVGPEFHEKLLSAEKAVT
GYRDPYTGQSVSLFQALKKGLIPREQGLRLLDAQLSTGGIVDPSKSHRVPLDVACARGCL
DEETSRALSAPRADAKAYSDPSTGEPATYGELQQRCRPDQLTGLSLLPLSEKAARARQEE
LYSELQARETFEKTPVEVPVGGFKGRTVTVWELISSEYFTAEQRQELLRQFRTGKVTVEK
VIKILITIVEEVETLRQERLSFSGLRAPVPASELLASGVLSRAQFEQLKDGKTTVKDLSE
LGSVRTLLQGSGCLAGIYLEDTKEKVSIYEAMRRGLLRATTAALLLEAQAATGFLVDPVR
NQRLYVHEAVKAGVVGPELHEQLLSAEKAVTGYRDPYSGSTISLFQAMQKGLVLRQHGIR
LLEAQIATGGIIDPVHSHRVPVDVAYQRGYFSEEMNRVLADPSDDTKGFFDPNTHENLTY

RQLLERCVEDPETGLRLLPLKGAEKAEVVETTQVYTEEETRRAFEETQIDIPGGGSHGGS
TMSLWEVMQSDLIPEEQRAQLMADFQAGRVTKERMIIIIIEIIEKTEIIRQQGLASYDYV
RRRLTAEDLFEARIISLETYNLLREGTRSLREALEAESAWCYLYGTGSVAGVYLPGSRQT
LSIYQALKKGLLSAEVARLLLEAQAATGFLLDPVKGERLTVDEAVRKGLVGPELHDRLLS
AERAVTGYRDPYTEQTISLFQAMKKELIPTEEALRLLDAQLATGGIVDPRLGFHLPLEVA
YQRGYLNKDTHDQLSEPSEVRSYVDPSTDERLSYTQLLRRCRRDDGTGQLLLPLSDARKL
TFRGLRKQITMEELVRSQVMDEATALQLREGLTSIEEVTKNLQKFLEGTSCIAGVFVDAT
KERLSVYQAMKKGIIRPGTAFELLEAQAATGYVIDPIKGLKLTVEEAVRMGIVGPEFKDK
LLSAERAVTGYKDPYSGKLISLFQAMKKGLILKDHGIRLLEAQIATGGIIDPEESHRLPV
EVAYKRGLFDEEMNEILTDPSDDTKGFFDPNTEENLTYLQLMERCITDPQTGLCLLPLKE
KKRERKTSSKSSVRKRRVVIVDPETGKEMSVYEAYRKGLIDHQTYLELSEQECEWEEITI
SSSDGVVKSMIIDRRSGRQYDIDDAIAKNLIDRSALDQYRAGTLSITEFADMLSGNAGGF
RSRSSSVGSSSSYPISPAVSRTQLASWSDPTEETGPVAGILDTETLEKVSITEAMHRNLV
DNITGQRLLEAQACTGGIIDPSTGERFPVTDAVNKGLVDKIMVDRINLAQKAFCGFEDPR
TKTKMSAAQALKKGWLYYEAGQRFLEVQYLTGGLIEPDTPGRVPLDEALQRGTVDARTAQ
KLRDVGAYSKYLTCPKTKLKISYKDALDRSMVEEGTGLRLLEAAAQSTKGYYSPYSVSGS
GSTAGSRTGSRTGSRAGSRRGSFDATGSGFSMTFSSSSYSSSGYGRRYASGSSASLGGPE
SAVA
>sp|P04792|HSPB1_HUMAN Heat shock protein beta-1 OS=Homo sapiens OX=9606 GN=HSPB1 PE=1 SV=2
MTERRVPFSLLRGPSWDPFRDWYPHSRLFDQAFGLPRLPEEWSQWLGGSSWPGYVRPLPP
AAIESPAVAAPAYSRALSRQLSSGVSEIRHTADRWRVSLDVNHFAPDELTVKTKDGVVEI
TGKHEERQDEHGYISRCFTRKYTLPPGVDPTQVSSSLSPEGTLTVEAPMPKLATQSNEIT
IPVTFESRAQLGGPEAAKSDETAAK
>sp|P68366|TBA4A_HUMAN Tubulin alpha-4A chain OS=Homo sapiens OX=9606 GN=TUBA4A PE=1 SV=1
MRECISVHVGQAGVQMGNACWELYCLEHGIQPDGQMPSDKTIGGGDDSFTTFFCETGAGK
HVPRAVFVDLEPTVIDEIRNGPYRQLFHPEQLITGKEDAANNYARGHYTIGKEIIDPVLD
RIRKLSDQCTGLQGFLVFHSFGGGTGSGFTSLLMERLSVDYGKKSKLEFSIYPAPQVSTA
VVEPYNSILTTHTTLEHSDCAFMVDNEAIYDICRRNLDIERPTYTNLNRLISQIVSSITA
SLRFDGALNVDLTEFQTNLVPYPRIHFPLATYAPVISAEKAYHEQLSVAEITNACFEPAN
QMVKCDPRHGKYMACCLLYRGDVVPKDVNAAIAAIKTKRSIQFVDWCPTGFKVGINYQPP
TVVPGGDLAKVQRAVCMLSNTTAIAEAWARLDHKFDLMYAKRAFVHWYVGEGMEEGEFSE
AREDMAALEKDYEEVGIDSYEDEDEGEE
>sp|Q13885|TBB2A_HUMAN Tubulin beta-2A chain OS=Homo sapiens OX=9606 GN=TUBB2A PE=1 SV=1
MREIVHIQAGQCGNQIGAKFWEVISDEHGIDPTGSYHGDSDLQLERINVYYNEAAGNKYV
PRAILVDLEPGTMDSVRSGPFGQIFRPDNFVFGQSGAGNNWAKGHYTEGAELVDSVLDVV
RKESESCDCLQGFQLTHSLGGGTGSGMGTLLISKIREEYPDRIMNTFSVMPSPKVSDTVV
EPYNATLSVHQLVENTDETYSIDNEALYDICFRTLKLTTPTYGDLNHLVSATMSGVTTCL
RFPGQLNADLRKLAVNMVPFPRLHFFMPGFAPLTSRGSQQYRALTVPELTQQMFDSKNMM
AACDPRHGRYLTVAAIFRGRMSMKEVDEQMLNVQNKNSSYFVEWIPNNVKTAVCDIPPRG
LKMSATFIGNSTAIQELFKRISEQFTAMFRRKAFLHWYTGEGMDEMEFTEAESNMNDLVS
EYQQYQDATADEQGEFEEEEGEDEA
>sp|Q9BVA1|TBB2B_HUMAN Tubulin beta-2B chain OS=Homo sapiens OX=9606 GN=TUBB2B PE=1 SV=1
MREIVHIQAGQCGNQIGAKFWEVISDEHGIDPTGSYHGDSDLQLERINVYYNEATGNKYV

PRAILVDLEPGTMDSVRSGPFGQIFRPDNFVFGQSGAGNNWAKGHYTEGAELVDSVLDVV
RKESESCDCLQGFQLTHSLGGGTGSGMGTLLISKIREEYPDRIMNTFSVMPSPKVSDTVV
EPYNATLSVHQLVENTDETYCIDNEALYDICFRTLKLTTPTYGDLNHLVSATMSGVTTCL
RFPGQLNADLRKLAVNMVPFPRLHFFMPGFAPLTSRGSQQYRALTVPELTQQMFDSKNMM
AACDPRHGRYLTVAAIFRGRMSMKEVDEQMLNVQNKNSSYFVEWIPNNVKTAVCDIPPRG
LKMSATFIGNSTAIQELFKRISEQFTAMFRRKAFLHWYTGEGMDEMEFTEAESNMNDLVS
EYQQYQDATADEQGEFEEEEGEDEA
>sp|A6NNZ2|TBB8B_HUMAN Tubulin beta 8B OS=Homo sapiens OX=9606 GN=TUBB8B PE=1 SV=1
MREIVLTQTGQCGNQIGAKFWEVISDEHAIDSAGTYHGDSHLQLERINVHHHEASGGRYV
PRAVLVDLEPGTMDSVHSGPFGQVFRPDNFISGQCGAGNNWAKGRYTEGAELTESVMDVV
RKEAESCDCLQGFQLTHSLGGGTGSGMGTLLISKIREEYPDRIINTFSILPSPKVSDTVV
EPYNATLSVHQLIENADETFCIDNEALYDICSRTLKLPTPTYGDLNHLVSATMSGVTTCL
RFPGQLNADLRKLAVNMVPFPRLHFFMPGFAPLTSRGSQQYRALTVAELTQQMFDAKNMM
AACDPRHGCYLTVAAIFRGRMPMREVDEQMFNIQDKNSSYFADWFPDNVKTAVCDIPPRG
LKMSATFIGNNAAIQELFTCVSEQFTAMFRRKAFLHWYTGEGMDEMEFTEAESNMNDLVS
EYQQYQDATAEEEEDEEYAEEEVA
>sp|P13639|EF2_HUMAN Elongation factor 2 OS=Homo sapiens OX=9606 GN=EEF2 PE=1 SV=4
MVNFTVDQIRAIMDKKANIRNMSVIAHVDHGKSTLTDSLVCKAGIIASARAGETRFTDTR
KDEQERCITIKSTAISLFYELSENDLNFIKQSKDGAGFLINLIDSPGHVDFSSEVTAALR
VTDGALVVVDCVSGVCVQTETVLRQAIAERIKPVLMMNKMDRALLELQLEPEELYQTFQR
IVENVNVIISTYGEGESGPMGNIMIDPVLGTVGFGSGLHGWAFTLKQFAEMYVAKFAAKG
EGQLGPAERAKKVEDMMKKLWGDRYFDPANGKFSKSATSPEGKKLPRTFCQLILDPIFKV
FDAIMNFKKEETAKLIEKLDIKLDSEDKDKEGKPLLKAVMRRWLPAGDALLQMITIHLPS
PVTAQKYRCELLYEGPPDDEAAMGIKSCDPKGPLMMYISKMVPTSDKGRFYAFGRVFSGL
VSTGLKVRIMGPNYTPGKKEDLYLKPIQRTILMMGRYVEPIEDVPCGNIVGLVGVDQFLV
KTGTITTFEHAHNMRVMKFSVSPVVRVAVEAKNPADLPKLVEGLKRLAKSDPMVQCIIEE
SGEHIIAGAGELHLEICLKDLEEDHACIPIKKSDPVVSYRETVSEESNVLCLSKSPNKHN
RLYMKARPFPDGLAEDIDKGEVSARQELKQRARYLAEKYEWDVAEARKIWCFGPDGTGPN
ILTDITKGVQYLNEIKDSVVAGFQWATKEGALCEENMRGVRFDVHDVTLHADAIHRGGGQ
IIPTARRCLYASVLTAQPRLMEPIYLVEIQCPEQVVGGIYGVLNRKRGHVFEESQVAGTP
MFVVKAYLPVNESFGFTADLRSNTGGQAFPQCVFDHWQILPGDPFDNSSRPSQVVAETRK
RKGLKEGIPALDNFLDKL
>sp|Q15029|U5S1_HUMAN 116 kDa U5 small nuclear ribonucleoprotein component OS=Homo sapiens OX=9606 GN=EFTUD2 PE=1 SV=1
MDTDLYDEFGNYIGPELDSDEDDDELGRETKDLDEMDDDDDDDDVGDHDDDHPGMEVVLH
EDKKYYPTAEEVYGPEVETIVQEEDTQPLTEPIIKPVKTKKFTLMEQTLPVTVYEMDFLA
DLMDNSELIRNVTLCGHLHHGKTCFVDCLIEQTHPEIRKRYDQDLCYTDILFTEQERGVG
IKSTPVTVVLPDTKGKSYLFNIMDTPGHVNFSDEVTAGLRISDGVVLFIDAAEGVMLNTE
RLIKHAVQERLAVTVCINKIDRLILELKLPPTDAYYKLRHIVDEVNGLISMYSTDENLIL
SPLLGNVCFSSSQYSICFTLGSFAKIYADTFGDINYQEFAKRLWGDIYFNPKTRKFTKKA
PTSSSQRSFVEFILEPLYKILAQVVGDVDTSLPRTLDELGIHLTKEELKLNIRPLLRLVC
KKFFGEFTGFVDMCVQHIPSPKVGAKPKIEHTYTGGVDSDLGEAMSDCDPDGPLMCHTTK
MYSTDDGVQFHAFGRVLSGTIHAGQPVKVLGENYTLEDEEDSQICTVGRLWISVARYHIE
VNRVPAGNWVLIEGVDQPIVKTATITEPRGNEEAQIFRPLKFNTTSVIKIAVEPVNPSEL
PKMLDGLRKVNKSYPSLTTKVEESGEHVILGTGELYLDCVMHDLRKMYSEIDIKVADPVV

TFCETVVETSSLKCFAETPNKKNKITMIAEPLEKGLAEDIENEVVQITWNRKKLGEFFQT
KYDWDLLAARSIWAFGPDATGPNILVDDTLPSEVDKALLGSVKDSIVQGFQWGTREGPLC
DELIRNVKFKILDAVVAQEPLHRGGGQIIPTARRVVYSAFLMATPRLMEPYYFVEVQAPA
DCVSAVYTVLARRRGHVTQDAPIPGSPLYTIKAFIPAIDSFGFETDLRTHTQGQAFSLSV
FHHWQIVPGDPLDKSIVIRPLEPQPAPHLAREFMIKTRRRKGLSEDVSISKFFDDPMLLE
LAKQDVVLNYPM
>sp|P11021|BIP_HUMAN Endoplasmic reticulum chaperone BiP OS=Homo sapiens OX=9606 GN=HSPA5 PE=1 SV=2
MKLSLVAAMLLLLSAARAEEEDKKEDVGTVVGIDLGTTYSCVGVFKNGRVEIIANDQGNR
ITPSYVAFTPEGERLIGDAAKNQLTSNPENTVFDAKRLIGRTWNDPSVQQDIKFLPFKVV
EKKTKPYIQVDIGGGQTKTFAPEEISAMVLTKMKETAEAYLGKKVTHAVVTVPAYFNDAQ
RQATKDAGTIAGLNVMRIINEPTAAAIAYGLDKREGEKNILVFDLGGGTFDVSLLTIDNG
VFEVVATNGDTHLGGEDFDQRVMEHFIKLYKKKTGKDVRKDNRAVQKLRREVEKAKRALS
SQHQARIEIESFYEGEDFSETLTRAKFEELNMDLFRSTMKPVQKVLEDSDLKKSDIDEIV
LVGGSTRIPKIQQLVKEFFNGKEPSRGINPDEAVAYGAAVQAGVLSGDQDTGDLVLLDVC
PLTLGIETVGGVMTKLIPRNTVVPTKKSQIFSTASDNQPTVTIKVYEGERPLTKDNHLLG
TFDLTGIPPAPRGVPQIEVTFEIDVNGILRVTAEDKGTGNKNKITITNDQNRLTPEEIER
MVNDAEKFAEEDKKLKERIDTRNELESYAYSLKNQIGDKEKLGGKLSSEDKETMEKAVEE
KIEWLESHQDADIEDFKAKKKELEEIVQPIISKLYGSAGPPPTGEEDTAEKDEL
>sp|P68371|TBB4B_HUMAN Tubulin beta-4B chain OS=Homo sapiens OX=9606 GN=TUBB4B PE=1 SV=1
MREIVHLQAGQCGNQIGAKFWEVISDEHGIDPTGTYHGDSDLQLERINVYYNEATGGKYV
PRAVLVDLEPGTMDSVRSGPFGQIFRPDNFVFGQSGAGNNWAKGHYTEGAELVDSVLDVV
RKEAESCDCLQGFQLTHSLGGGTGSGMGTLLISKIREEYPDRIMNTFSVVPSPKVSDTVV
EPYNATLSVHQLVENTDETYCIDNEALYDICFRTLKLTTPTYGDLNHLVSATMSGVTTCL
RFPGQLNADLRKLAVNMVPFPRLHFFMPGFAPLTSRGSQQYRALTVPELTQQMFDAKNMM
AACDPRHGRYLTVAAVFRGRMSMKEVDEQMLNVQNKNSSYFVEWIPNNVKTAVCDIPPRG
LKMSATFIGNSTAIQELFKRISEQFTAMFRRKAFLHWYTGEGMDEMEFTEAESNMNDLVS
EYQQYQDATAEEEGEFEEEAEEEVA
>sp|P04350|TBB4A_HUMAN Tubulin beta-4A chain OS=Homo sapiens OX=9606 GN=TUBB4A PE=1 SV=2
MREIVHLQAGQCGNQIGAKFWEVISDEHGIDPTGTYHGDSDLQLERINVYYNEATGGNYV
PRAVLVDLEPGTMDSVRSGPFGQIFRPDNFVFGQSGAGNNWAKGHYTEGAELVDAVLDVV
RKEAESCDCLQGFQLTHSLGGGTGSGMGTLLISKIREEFPDRIMNTFSVVPSPKVSDTVV
EPYNATLSVHQLVENTDETYCIDNEALYDICFRTLKLTTPTYGDLNHLVSATMSGVTTCL
RFPGQLNADLRKLAVNMVPFPRLHFFMPGFAPLTSRGSQQYRALTVPELTQQMFDAKNMM
AACDPRHGRYLTVAAVFRGRMSMKEVDEQMLSVQSKNSSYFVEWIPNNVKTAVCDIPPRG
LKMAATFIGNSTAIQELFKRISEQFTAMFRRKAFLHWYTGEGMDEMEFTEAESNMNDLVS
EYQQYQDATAEEGEFEEEAEEEVA
>sp|Q3ZCM7|TBB8_HUMAN Tubulin beta-8 chain OS=Homo sapiens OX=9606 GN=TUBB8 PE=1 SV=2
MREIVLTQIGQCGNQIGAKFWEVISDEHAIDSAGTYHGDSHLQLERINVYYNEASGGRYV
PRAVLVDLEPGTMDSVRSGPFGQVFRPDNFIFGQCGAGNNWAKGHYTEGAELMESVMDVV
RKEAESCDCLQGFQLTHSLGGGTGSGMGTLLLSKIREEYPDRIINTFSILPSPKVSDTVV
EPYNATLSVHQLIENADETFCIDNEALYDICSKTLKLPTPTYGDLNHLVSATMSGVTTCL
RFPGQLNADLRKLAVNMVPFPRLHFFMPGFAPLTSRGSQQYRALTVAELTQQMFDAKNMM
AACDPRHGRYLTAAAIFRGRMPMREVDEQMFNIQDKNSSYFADWLPNNVKTAVCDIPPRG

LKMSATFIGNNTAIQELFKRVSEQFTAMFRRKAFLHWYTGEGMDEMEFTEAESNMNDLVS
EYQQYQDATAEEEEDEEYAEEEVA
>sp|P19013|K2C4_HUMAN Keratin, type II cytoskeletal 4 OS=Homo sapiens OX=9606 GN=KRT4 PE=1 SV=5
MIARQQCVRGGPRGFSCGSAIVGGGKRGAFSSVSMSGGAGRCSSGGFGSRSLYNLRGNKS
ISMSVAGSRQGACFGGAGGFGTGGFGGGFGGSFSGKGGPGFPVCPAGGIQEVTINQSLLT
PLHVEIDPEIQKVRTEEREQIKLLNNKFASFIDKVQFLEQQNKVLETKWNLLQQQTTTTS
SKNLEPLFETYLSVLRKQLDTLGNDKGRLQSELKTMQDSVEDFKTKYEEEINKRTAAEND
FVVLKKDVDAAYLNKVELEAKVDSLNDEINFLKVLYDAELSQMQTHVSDTSVVLSMDNNR
NLDLDSIIAEVRAQYEEIAQRSKAEAEALYQTKVQQLQISVDQHGDNLKNTKSEIAELNR
MIQRLRAEIENIKKQCQTLQVSVADAEQRGENALKDAHSKRVELEAALQQAKEELARMLR
EYQELMSVKLALDIEIATYRKLLEGEEYRMSGECQSAVSISVVSGSTSTGGISGGLGSGS
GFGLSSGFGSGSGSGFGFGGSVSGSSSSKIISTTTLNKRR
>sp|P14136|GFAP_HUMAN Glial fibrillary acidic protein OS=Homo sapiens OX=9606 GN=GFAP PE=1 SV=1
MERRRITSAARRSYVSSGEMMVGGLAPGRRLGPGTRLSLARMPPPLPTRVDFSLAGALNA
GFKETRASERAEMMELNDRFASYIEKVRFLEQQNKALAAELNQLRAKEPTKLADVYQAEL
RELRLRLDQLTANSARLEVERDNLAQDLATVRQKLQDETNLRLEAENNLAAYRQEADEAT
LARLDLERKIESLEEEIRFLRKIHEEEVRELQEQLARQQVHVELDVAKPDLTAALKEIRT
QYEAMASSNMHEAEEWYRSKFADLTDAAARNAELLRQAKHEANDYRRQLQSLTCDLESLR
GTNESLERQMREQEERHVREAASYQEALARLEEEGQSLKDEMARHLQEYQDLLNVKLALD
IEIATYRKLLEGEENRITIPVQTFSNLQIRETSLDTKSVSEGHLKRNIVVKTVEMRDGEV
IKESKQEHKDVM
>sp|P31947|1433S_HUMAN 14-3-3 protein sigma OS=Homo sapiens OX=9606 GN=SFN PE=1 SV=1
MERASLIQKAKLAEQAERYEDMAAFMKGAVEKGEELSCEERNLLSVAYKNVVGGQRAAWR
VLSSIEQKSNEEGSEEKGPEVREYREKVETELQGVCDTVLGLLDSHLIKEAGDAESRVFY
LKMKGDYYRYLAEVATGDDKKRIIDSARSAYQEAMDISKKEMPPTNPIRLGLALNFSVFH
YEIANSPEEAISLAKTTFDEAMADLHTLSEDSYKDSTLIMQLLRDNLTLWTADNAGEEGG
EAPQEPQS
>sp|Q8TF66|LRC15_HUMAN Leucine-rich repeat-containing protein 15 OS=Homo sapiens OX=9606 GN=LRRC15 PE=2 SV=2
MPLKHYLLLLVGCQAWGAGLAYHGCPSECTCSRASQVECTGARIVAVPTPLPWNAMSLQI
LNTHITELNESPFLNISALIALRIEKNELSRITPGAFRNLGSLRYLSLANNKLQVLPIGL
FQGLDSLESLLLSSNQLLQIQPAHFSQCSNLKELQLHGNHLEYIPDGAFDHLVGLTKLNL
GKNSLTHISPRVFQHLGNLQVLRLYENRLTDIPMGTFDGLVNLQELALQQNQIGLLSPGL
FHNNHNLQRLYLSNNHISQLPPSVFMQLPQLNRLTLFGNSLKELSPGIFGPMPNLRELWL
YDNHISSLPDNVFSNLRQLQVLILSRNQISFISPGAFNGLTELRELSLHTNALQDLDGNV
FRMLANLQNISLQNNRLRQLPGNIFANVNGLMAIQLQNNQLENLPLGIFDHLGKLCELRL
YDNPWRCDSDILPLRNWLLLNQPRLGTDTVPVCFSPANVRGQSLIIINVNVAVPSVHVPE
VPSYPETPWYPDTPSYPDTTSVSSTTELTSPVEDYTDLTTIQVTDDRSVWGMTQAQSGLA
IAAIVIGIVALACSLAACVGCCCCKKRSQAVLMQMKAPNEC
>sp|P07437|TBB5_HUMAN Tubulin beta chain OS=Homo sapiens OX=9606 GN=TUBB PE=1 SV=2
MREIVHIQAGQCGNQIGAKFWEVISDEHGIDPTGTYHGDSDLQLDRISVYYNEATGGKYV
PRAILVDLEPGTMDSVRSGPFGQIFRPDNFVFGQSGAGNNWAKGHYTEGAELVDSVLDVV
RKEAESCDCLQGFQLTHSLGGGTGSGMGTLLISKIREEYPDRIMNTFSVVPSPKVSDTVV

EPYNATLSVHQLVENTDETYCIDNEALYDICFRTLKLTTPTYGDLNHLVSATMSGVTTCL
RFPGQLNADLRKLAVNMVPFPRLHFFMPGFAPLTSRGSQQYRALTVPELTQQVFDAKNMM
AACDPRHGRYLTVAAVFRGRMSMKEVDEQMLNVQNKNSSYFVEWIPNNVKTAVCDIPPRG
LKMAVTFIGNSTAIQELFKRISEQFTAMFRRKAFLHWYTGEGMDEMEFTEAESNMNDLVS
EYQQYQDATAEEEEDFGEEAEEEA
>sp|Q9BQE3|TBA1C_HUMAN Tubulin alpha-1C chain OS=Homo sapiens OX=9606 GN=TUBA1C PE=1 SV=1
MRECISIHVGQAGVQIGNACWELYCLEHGIQPDGQMPSDKTIGGGDDSFNTFFSETGAGK
HVPRAVFVDLEPTVIDEVRTGTYRQLFHPEQLITGKEDAANNYARGHYTIGKEIIDLVLD
RIRKLADQCTGLQGFLVFHSFGGGTGSGFTSLLMERLSVDYGKKSKLEFSIYPAPQVSTA
VVEPYNSILTTHTTLEHSDCAFMVDNEAIYDICRRNLDIERPTYTNLNRLISQIVSSITA
SLRFDGALNVDLTEFQTNLVPYPRIHFPLATYAPVISAEKAYHEQLTVAEITNACFEPAN
QMVKCDPRHGKYMACCLLYRGDVVPKDVNAAIATIKTKRTIQFVDWCPTGFKVGINYQPP
TVVPGGDLAKVQRAVCMLSNTTAVAEAWARLDHKFDLMYAKRAFVHWYVGEGMEEGEFSE
AREDMAALEKDYEEVGADSADGEDEGEEY
>sp|P14735|IDE_HUMAN Insulin-degrading enzyme OS=Homo sapiens OX=9606 GN=IDE PE=1 SV=4
MRYRLAWLLHPALPSTFRSVLGARLPPPERLCGFQKKTYSKMNNPAIKRIGNHITKSPED
KREYRGLELANGIKVLLISDPTTDKSSAALDVHIGSLSDPPNIAGLSHFCEHMLFLGTKK
YPKENEYSQFLSEHAGSSNAFTSGEHTNYYFDVSHEHLEGALDRFAQFFLCPLFDESCKD
REVNAVDSEHEKNVMNDAWRLFQLEKATGNPKHPFSKFGTGNKYTLETRPNQEGIDVRQE
LLKFHSAYYSSNLMAVCVLGRESLDDLTNLVVKLFSEVENKNVPLPEFPEHPFQEEHLKQ
LYKIVPIKDIRNLYVTFPIPDLQKYYKSNPGHYLGHLIGHEGPGSLLSELKSKGWVNTLV
GGQKEGARGFMFFIINVDLTEEGLLHVEDIILHMFQYIQKLRAEGPQEWVFQECKDLNAV
AFRFKDKERPRGYTSKIAGILHYYPLEEVLTAEYLLEEFRPDLIEMVLDKLRPENVRVAI
VSKSFEGKTDRTEEWYGTQYKQEAIPDEVIKKWQNADLNGKFKLPTKNEFIPTNFEILPL
EKEATPYPALIKDTAMSKLWFKQDDKFFLPKACLNFEFFSPFAYVDPLHCNMAYLYLELL
KDSLNEYAYAAELAGLSYDLQNTIYGMYLSVKGYNDKQPILLKKIIEKMATFEIDEKRFE
IIKEAYMRSLNNFRAEQPHQHAMYYLRLLMTEVAWTKDELKEALDDVTLPRLKAFIPQLL
SRLHIEALLHGNITKQAALGIMQMVEDTLIEHAHTKPLLPSQLVRYREVQLPDRGWFVYQ
QRNEVHNNCGIEIYYQTDMQSTSENMFLELFCQIISEPCFNTLRTKEQLGYIVFSGPRRA
NGIQGLRFIIQSEKPPHYLESRVEAFLITMEKSIEDMTEEAFQKHIQALAIRRLDKPKKL
SAECAKYWGEIISQQYNFDRDNTEVAYLKTLTKEDIIKFYKEMLAVDAPRRHKVSVHVLA
REMDSCPVVGEFPCQNDINLSQAPALPQPEVIQNMTEFKRGLPLFPLVKPHINFMAAKL
>sp|P02545|LMNA_HUMAN Prelamin-A/C OS=Homo sapiens OX=9606 GN=LMNA PE=1 SV=1
METPSQRRATRSGAQASSTPLSPTRITRLQEKEDLQELNDRLAVYIDRVRSLETENAGLR
LRITESEEVVSREVSGIKAAYEAELGDARKTLDSVAKERARLQLELSKVREEFKELKARN
TKKEGDLIAAQARLKDLEALLNSKEAALSTALSEKRTLEGELHDLRGQVAKLEAALGEAK
KQLQDEMLRRVDAENRLQTMKEELDFQKNIYSEELRETKRRHETRLVEIDNGKQREFESR
LADALQELRAQHEDQVEQYKKELEKTYSAKLDNARQSAERNSNLVGAAHEELQQSRIRID
SLSAQLSQLQKQLAAKEAKLRDLEDSLARERDTSRRLLAEKEREMAEMRARMQQQLDEYQ
ELLDIKLALDMEIHAYRKLLEGEEERLRLSPSPTSQRSRGRASSHSSQTQGGGSVTKKRK
LESTESRSSFSQHARTSGRVAVEEVDEEGKFVRLRNKSNEDQSMGNWQIKRQNGDDPLLT
YRFPPKFTLKAGQVVTIWAAGAGATHSPPTDLVWKAQNTWGCGNSLRTALINSTGEEVAM
RKLVRSVTVVEDDEDEDGDDLLHHHHGSHCSSSGDPAEYNLRSRTVLCGTCGQPADKASA
SGSGAQVGGPISSGSSASSVTVTRSYRSVGGSGGGSFGDNLVTRSYLLGNSSPRTQSPQN

CSIM
>sp|Q08188|TGM3_HUMAN Protein-glutamine gamma-glutamyltransferase E OS=Homo sapiens OX=9606 GN=TGM3 PE=1 SV=4
MAALGVQSINWQTAFNRQAHHTDKFSSQELILRRGQNFQVLMIMNKGLGSNERLEFIVST
GPYPSESAMTKAVFPLSNGSSGGWSAVLQASNGNTLTISISSPASAPIGRYTMALQIFSQ
GGISSVKLGTFILLFNPWLNVDSVFMGNHAEREEYVQEDAGIIFVGSTNRIGMIGWNFGQ
FEEDILSICLSILDRSLNFRRDAATDVASRNDPKYVGRVLSAMINSNDDNGVLAGNWSGT
YTGGRDPRSWNGSVEILKNWKKSGFSPVRYGQCWVFAGTLNTALRSLGIPSRVITNFNSA
HDTDRNLSVDVYYDPMGNPLDKGSDSVWNFHVWNEGWFVRSDLGPSYGGWQVLDATPQER
SQGVFQCGPASVIGVREGDVQLNFDMPFIFAEVNADRITWLYDNTTGKQWKNSVNSHTIG
RYISTKAVGSNARMDVTDKYKYPEGSDQERQVFQKALGKLKPNTPFAATSSMGLETEEQE
PSIIGKLKVAGMLAVGKEVNLVLLLKNLSRDTKTVTVNMTAWTIIYNGTLVHEVWKDSAT
MSLDPEEEAEHPIKISYAQYEKYLKSDNMIRITAVCKVPDESEVVVERDIILDNPTLTLE
VLNEARVRKPVNVQMLFSNPLDEPVRDCVLMVEGSGLLLGNLKIDVPTLGPKEGSRVRFD
ILPSRSGTKQLLADFSCNKFPAIKAMLSIDVAE
>sp|P68104|EF1A1_HUMAN Elongation factor 1-alpha 1 OS=Homo sapiens OX=9606 GN=EEF1A1 PE=1 SV=1
MGKEKTHINIVVIGHVDSGKSTTTGHLIYKCGGIDKRTIEKFEKEAAEMGKGSFKYAWVL
DKLKAERERGITIDISLWKFETSKYYVTIIDAPGHRDFIKNMITGTSQADCAVLIVAAGV
GEFEAGISKNGQTREHALLAYTLGVKQLIVGVNKMDSTEPPYSQKRYEEIVKEVSTYIKK
IGYNPDTVAFVPISGWNGDNMLEPSANMPWFKGWKVTRKDGNASGTTLLEALDCILPPTR
PTDKPLRLPLQDVYKIGGIGTVPVGRVETGVLKPGMVVTFAPVNVTTEVKSVEMHHEALS
EALPGDNVGFNVKNVSVKDVRRGNVAGDSKNDPPMEAAGFTAQVIILNHPGQISAGYAPV
LDCHTAHIACKFAELKEKIDRRSGKKLEDGPKFLKSGDAAIVDMVPGKPMCVESFSDYPP
LGRFAVRDMRQTVAVGVIKAVDKKAAGAGKVTKSAQKAQKAK
>sp|Q5VTE0|EF1A3_HUMAN Putative elongation factor 1-alpha-like 3 OS=Homo sapiens OX=9606 GN=EEF1A1P5 PE=5 SV=1
MGKEKTHINIVVIGHVDSGKSTTTGHLIYKCGGIDKRTIEKFEKEAAEMGKGSFKYAWVL
DKLKAERERGITIDISLWKFETSKYYVTIIDAPGHRDFIKNMITGTSQADCAVLIVAAGV
GEFEAGISKNGQTREHALLAYTLGVKQLIVGVNKMDSTEPPYSQKRYEEIVKEVSTYIKK
IGYNPDTVAFVPISGWNGDNMLEPSANMPWFKGWKVTRKDGNASGTTLLEALDCILPPTR
PTDKPLRLPLQDVYKIGGIGTVPVGRVETGVLKPGMVVTFAPVNVTTEVKSVEMHHEALS
EALPGDNVGFKVKNVSVKDVRRGNVAGDSKNDPPMEAAGFTAQVIILNHPGQISAGYAPV
LDCHMAHIACKFAELKEKIDRRSGKKLEDGPKFLKSGDAAIVDMVPGKPMCVESFSDYPP
LGRFAVRDMRQTVAVGVIKAVDKKAAGAGKVTKSAQKAQKAK
>sp|Q05639|EF1A2_HUMAN Elongation factor 1-alpha 2 OS=Homo sapiens OX=9606 GN=EEF1A2 PE=1 SV=1
MGKEKTHINIVVIGHVDSGKSTTTGHLIYKCGGIDKRTIEKFEKEAAEMGKGSFKYAWVL
DKLKAERERGITIDISLWKFETTKYYITIIDAPGHRDFIKNMITGTSQADCAVLIVAAGV
GEFEAGISKNGQTREHALLAYTLGVKQLIVGVNKMDSTEPAYSEKRYDEIVKEVSAYIKK
IGYNPATVPFVPISGWHGDNMLEPSPNMPWFKGWKVERKEGNASGVSLLEALDTILPPTR
PTDKPLRLPLQDVYKIGGIGTVPVGRVETGILRPGMVVTFAPVNITTEVKSVEMHHEALS
EALPGDNVGFNVKNVSVKDIRRGNVCGDSKSDPPQEAAQFTSQVIILNHPGQISAGYSPV
IDCHTAHIACKFAELKEKIDRRSGKKLEDNPKSLKSGDAAIVEMVPGKPMCVESFSQYPP
LGRFAVRDMRQTVAVGVIKNVEKKSGGAGKVTKSAQKAQKAGK
>sp|Q14574|DSC3_HUMAN Desmocollin-3 OS=Homo sapiens OX=9606 GN=DSC3 PE=1 SV=3

MAAAGPRRSVRGAVCLHLLLTLVIFSRAGEACKKVILNVPSKLEADKIIGRVNLEECFRS
ADLIRSSDPDFRVLNDGSVYTARAVALSDKKRSFTIWLSDKRKQTQKEVTVLLEHQKKVS
KTRHTRETVLRRAKRRWAPIPCSMQENSLGPFPLFLQQVESDAAQNYTVFYSISGRGVDK
EPLNLFYIERDTGNLFCTRPVDREEYDVFDLIAYASTADGYSADLPLPLPIRVEDENDNH
PVFTEAIYNFEVLESSRPGTTVGVVCATDRDEPDTMHTRLKYSILQQTPRSPGLFSVHPS
TGVITTVSHYLDREVVDKYSLIMKVQDMDGQFFGLIGTSTCIITVTDSNDNAPTFRQNAY
EAFVEENAFNVEILRIPIEDKDLINTANWRVNFTILKGNENGHFKISTDKETNEGVLSVV
KPLNYEENRQVNLEIGVNNEAPFARDIPRVTALNRALVTVHVRDLDEGPECTPAAQYVRI
KENLAVGSKINGYKAYDPENRNGNGLRYKKLHDPKGWITIDEISGSIITSKILDREVETP
KNELYNITVLAIDKDDRSCTGTLAVNIEDVNDNPPEILQEYVVICKPKMGYTDILAVDPD
EPVHGAPFYFSLPNTSPEISRLWSLTKVNDTAARLSYQKNAGFQEYTIPITVKDRAGQAA
TKLLRVNLCECTHPTQCRATSRSTGVILGKWAILAILLGIALLFSVLLTLVCGVFGATKG
KRFPEDLAQQNLIISNTEAPGDDRVCSANGFMTQTTNNSSQGFCGTMGSGMKNGGQETIE
MMKGGNQTLESCRGAGHHHTLDSCRGGHTEVDNCRYTYSEWHSFTQPRLGEKLHRCNQNE
DRMPSQDYVLTYNYEGRGSPAGSVGCCSEKQEEDGLDFLNNLEPKFITLAEACTKR
>sp|P60174|TPIS_HUMAN Triosephosphate isomerase OS=Homo sapiens OX=9606 GN=TPI1 PE=1 SV=4
MAPSRKFFVGGNWKMNGRKQSLGELIGTLNAAKVPADTEVVCAPPTAYIDFARQKLDPKI
AVAAQNCYKVTNGAFTGEISPGMIKDCGATWVVLGHSERRHVFGESDELIGQKVAHALAE
GLGVIACIGEKLDEREAGITEKVVFEQTKVIADNVKDWSKVVLAYEPVWAIGTGKTATPQ
QAQEVHEKLRGWLKSNVSDAVAQSTRIIYGGSVTGATCKELASQPDVDGFLVGGASLKPE
FVDIINAKQ
>sp|P04083|ANXA1_HUMAN Annexin A1 OS=Homo sapiens OX=9606 GN=ANXA1 PE=1 SV=2
MAMVSEFLKQAWFIENEEQEYVQTVKSSKGGPGSAVSPYPTFNPSSDVAALHKAIMVKGV
DEATIIDILTKRNNAQRQQIKAAYLQETGKPLDETLKKALTGHLEEVVLALLKTPAQFDA
DELRAAMKGLGTDEDTLIEILASRTNKEIRDINRVYREELKRDLAKDITSDTSGDFRNAL
LSLAKGDRSEDFGVNEDLADSDARALYEAGERRKGTDVNVFNTILTTRSYPQLRRVFQKY
TKYSKHDMNKVLDLELKGDIEKCLTAIVKCATSKPAFFAEKLHQAMKGVGTRHKALIRIM
VSRSEIDMNDIKAFYQKMYGISLCQAILDETKGDYEKILVALCGGN
>sp|P54652|HSP72_HUMAN Heat shock-related 70 kDa protein 2 OS=Homo sapiens OX=9606 GN=HSPA2 PE=1 SV=1
MSARGPAIGIDLGTTYSCVGVFQHGKVEIIANDQGNRTTPSYVAFTDTERLIGDAAKNQV
AMNPTNTIFDAKRLIGRKFEDATVQSDMKHWPFRVVSEGGKPKVQVEYKGETKTFFPEEI
SSMVLTKMKEIAEAYLGGKVHSAVITVPAYFNDSQRQATKDAGTITGLNVLRIINEPTAA
AIAYGLDKKGCAGGEKNVLIFDLGGGTFDVSILTIEDGIFEVKSTAGDTHLGGEDFDNRM
VSHLAEEFKRKHKKDIGPNKRAVRRLRTACERAKRTLSSSTQASIEIDSLYEGVDFYTSI
TRARFEELNADLFRGTLEPVEKALRDAKLDKGQIQEIVLVGGSTRIPKIQKLLQDFFNGK
ELNKSINPDEAVAYGAAVQAAILIGDKSENVQDLLLLDVTPLSLGIETAGGVMTPLIKRN
TTIPTKQTQTFTTYSDNQSSVLVQVYEGERAMTKDNNLLGKFDLTGIPPAPRGVPQIEVT
FDIDANGILNVTAADKSTGKENKITITNDKGRLSKDDIDRMVQEAERYKSEDEANRDRVA
AKNALESYTYNIKQTVEDEKLRGKISEQDKNKILDKCQEVINWLDRNQMAEKDEYEHKQK
ELERVCNPIISKLYQGGPGGGSGGGGSGASGGPTIEEVD
>sp|P05089|ARGI1_HUMAN Arginase-1 OS=Homo sapiens OX=9606 GN=ARG1 PE=1 SV=2
MSAKSRTIGIIGAPFSKGQPRGGVEEGPTVLRKAGLLEKLKEQECDVKDYGDLPFADIPN
DSPFQIVKNPRSVGKASEQLAGKVAEVKKNGRISLVLGGDHSLAIGSISGHARVHPDLGV

IWVDAHTDINTPLTTTSGNLHGQPVSFLLKELKGKIPDVPGFSWVTPCISAKDIVYIGLR
DVDPGEHYILKTLGIKYFSMTEVDRLGIGKVMEETLSYLLGRKKRPIHLSFDVDGLDPSF
TPATGTPVVGGLTYREGLYITEEIYKTGLLSGLDIMEVNPSLGKTPEEVTRTVNTAVAIT
LACFGLAREGNHKPIDYLNPPK
>sp|Q6KB66|K2C80_HUMAN Keratin, type II cytoskeletal 80 OS=Homo sapiens OX=9606 GN=KRT80 PE=1 SV=2
MACRSCVVGFSSLSSCEVTPVGSPRPGTSGWDSCRAPGPGFSSRSLTGCWSAGTISKVTV
NPGLLVPLDVKLDPAVQQLKNQEKEEMKALNDKFASLIGKVQALEQRNQLLETRWSFLQG
QDSAIFDLGHLYEEYQGRLQEELRKVSQERGQLEANLLQVLEKVEEFRIRYEDEISKRTD
MEFTFVQLKKDLDAECLHRTELETKLKSLESFVELMKTIYEQELKDLAAQVKDVSVTVGM
DSRCHIDLSGIVEEVKAQYDAVAARSLEEAEAYSRSQLEEQAARSAEYGSSLQSSRSEIA
DLNVRIQKLRSQILSVKSHCLKLEENIKTAEEQGELAFQDAKTKLAQLEAALQQAKQDMA
RQLRKYQELMNVKLALDIEIATYRKLVEGEEGRMDSPSATVVSAVQSRCKTAASRSGLSK
APSRKKKGSKGPVIKITEMSEKYFSQESEVSE
>sp|P13646|K1C13_HUMAN Keratin, type I cytoskeletal 13 OS=Homo sapiens OX=9606 GN=KRT13 PE=1 SV=4
MSLRLQSSSASYGGGFGGGSCQLGGGRGVSTCSTRFVSGGSAGGYGGGVSCGFGGGAGSG
FGGGYGGGLGGGYGGGLGGGFGGGFAGGFVDFGACDGGLLTGNEKITMQNLNDRLASYLE
KVRALEEANADLEVKIRDWHLKQSPASPERDYSPYYKTIEELRDKILTATIENNRVILEI
DNARLAADDFRLKYENELALRQSVEADINGLRRVLDELTLSKTDLEMQIESLNEELAYMK
KNHEEEMKEFSNQVVGQVNVEMDATPGIDLTRVLAEMREQYEAMAERNRRDAEEWFHTKS
AELNKEVSTNTAMIQTSKTEITELRRTLQGLEIELQSQLSMKAGLENTVAETECRYALQL
QQIQGLISSIEAQLSELRSEMECQNQEYKMLLDIKTRLEQEIATYRSLLEGQDAKMIGFP
SSAGSVSPRSTSVTTTSSASVTTTSNASGRRTSDVRRP
>sp|P12035|K2C3_HUMAN Keratin, type II cytoskeletal 3 OS=Homo sapiens OX=9606 GN=KRT3 PE=1 SV=3
MSRQASKTSGGGSQGFSGRSAVVSGSSRMSCVAHSGGAGGGAYGFRSGAGGFGSRSLYNL
GGNKSISISVAAGGSRAGGFGGGRSSCAFAGGYGGGFGSGYGGGFGGGFGGGRGMGGGFG
GAGGFGGAGGFGGAGGFGGPGGFGGSGGFGGPGSLGSPGGFGPGGFPGGIQEVTINQSLL
QPLNVEIDPQIGQVKAQEREQIKTLNNKFASFIDKVRFLEQQNKVLETKWNLLQQQGTSS
ISGTNNLEPLFENHINYLRSYLDNILGERGRLDSELKNMEDLVEDFKKKYEDEINKRTAA
ENEFVTLKKDVDSAYMNKVELQAKVDALIDEIDFLRTLYDAELSQMQSHISDTSVVLSMD
NNRSLDLDSIIAEVRAQYEDIAQRSKAEAEALYQTKLGELQTTAGRHGDDLRNTKSEIIE
LNRMIQRLRAEIEGVKKQNANLQTAIAEAEQHGEMALKDANAKLQELQAALQQAKDDLAR
LLRDYQELMNVKLALDVEIATYRKLLEGEEYRMSGECPSAVSISVVSSSTTSASAGGYGG
GYGGGMGGGLGGGFSAGGGSGSGFGRGGGGGIGGGFGGGSSGFSGGSGFGSISGARYGVS
GGGFSSASNRGGSIKFSQSSQSSQRYSR
>sp|Q13509|TBB3_HUMAN Tubulin beta-3 chain OS=Homo sapiens OX=9606 GN=TUBB3 PE=1 SV=2
MREIVHIQAGQCGNQIGAKFWEVISDEHGIDPSGNYVGDSDLQLERISVYYNEASSHKYV
PRAILVDLEPGTMDSVRSGAFGHLFRPDNFIFGQSGAGNNWAKGHYTEGAELVDSVLDVV
RKECENCDCLQGFQLTHSLGGGTGSGMGTLLISKVREEYPDRIMNTFSVVPSPKVSDTVV
EPYNATLSIHQLVENTDETYCIDNEALYDICFRTLKLATPTYGDLNHLVSATMSGVTTSL
RFPGQLNADLRKLAVNMVPFPRLHFFMPGFAPLTARGSQQYRALTVPELTQQMFDAKNMM
AACDPRHGRYLTVATVFRGRMSMKEVDEQMLAIQSKNSSYFVEWIPNNVKAVCDIPPRG
LKMSSTFIGNSTAIQELFKRISEQFTAMFRRKAFLHWYTGEGMDEMEFTEAESNMNDLVS
EYQQYQDATAEEEGEMYEDDEEESEAQGPK

>sp|P27482|CALL3_HUMAN Calmodulin-like protein 3 OS=Homo sapiens OX=9606 GN=CALML3 PE=1 SV=2
MADQLTEEQVTEFKEAFSLFDKDGDGCITTRELGTVMRSLGQNPTEAELRDMMSEIDRDG
NGTVDFPEFLGMMARKMKDTDNEEEIREAFRVFDKDGNGFVSAAELRHVMTRLGEKLSDE
EVDEMIRAADTDGDGQVNYEEFVRVLVSK
>sp|P0DMV9|HS71B_HUMAN Heat shock 70 kDa protein 1B OS=Homo sapiens OX=9606 GN=HSPA1B PE=1 SV=1
MAKAAAIGIDLGTTYSCVGVFQHGKVEIIANDQGNRTTPSYVAFTDTERLIGDAAKNQVA
LNPQNTVFDAKRLIGRKFGDPVVQSDMKHWPFQVINDGDKPKVQVSYKGETKAFYPEEIS
SMVLTKMKEIAEAYLGYPVTNAVITVPAYFNDSQRQATKDAGVIAGLNVLRIINEPTAAA
IAYGLDRTGKGERNVLIFDLGGGTFDVSILTIDDGIFEVKATAGDTHLGGEDFDNRLVNH
FVEEFKRKHKKDISQNKRAVRRLRTACERAKRTLSSSTQASLEIDSLFEGIDFYTSITRA
RFEELCSDLFRSTLEPVEKALRDAKLDKAQIHDLVLVGGSTRIPKVQKLLQDFFNGRDLN
KSINPDEAVAYGAAVQAAILMGDKSENVQDLLLLDVAPLSLGLETAGGVMTALIKRNSTI
PTKQTQIFTTYSDNQPGVLIQVYEGERAMTKDNNLLGRFELSGIPPAPRGVPQIEVTFDI
DANGILNVTATDKSTGKANKITITNDKGRLSKEEIERMVQEAEKYKAEDEVQRERVSAKN
ALESYAFNMKSAVEDEGLKGKISEADKKKVLDKCQEVISWLDANTLAEKDEFEHKRKELE
QVCNPIISGLYQGAGGPGPGGFGAQGPKGGSGSGPTIEEVD
>sp|P0DMV8|HS71A_HUMAN Heat shock 70 kDa protein 1A OS=Homo sapiens OX=9606 GN=HSPA1A PE=1 SV=1
MAKAAAIGIDLGTTYSCVGVFQHGKVEIIANDQGNRTTPSYVAFTDTERLIGDAAKNQVA
LNPQNTVFDAKRLIGRKFGDPVVQSDMKHWPFQVINDGDKPKVQVSYKGETKAFYPEEIS
SMVLTKMKEIAEAYLGYPVTNAVITVPAYFNDSQRQATKDAGVIAGLNVLRIINEPTAAA
IAYGLDRTGKGERNVLIFDLGGGTFDVSILTIDDGIFEVKATAGDTHLGGEDFDNRLVNH
FVEEFKRKHKKDISQNKRAVRRLRTACERAKRTLSSSTQASLEIDSLFEGIDFYTSITRA
RFEELCSDLFRSTLEPVEKALRDAKLDKAQIHDLVLVGGSTRIPKVQKLLQDFFNGRDLN
KSINPDEAVAYGAAVQAAILMGDKSENVQDLLLLDVAPLSLGLETAGGVMTALIKRNSTI
PTKQTQIFTTYSDNQPGVLIQVYEGERAMTKDNNLLGRFELSGIPPAPRGVPQIEVTFDI
DANGILNVTATDKSTGKANKITITNDKGRLSKEEIERMVQEAEKYKAEDEVQRERVSAKN
ALESYAFNMKSAVEDEGLKGKISEADKKKVLDKCQEVISWLDANTLAEKDEFEHKRKELE
QVCNPIISGLYQGAGGPGPGGFGAQGPKGGSGSGPTIEEVD
>sp|Q86SJ6|DSG4_HUMAN Desmoglein-4 OS=Homo sapiens OX=9606 GN=DSG4 PE=1 SV=1
MDWLFFRNICLLIILMVVMEVNSEFIVEVKEFDIENGTTKWQTVRRQKREWIKFAAACRE
GEDNSKRNPIAKIRSDCESNQKITYRISGVGIDRPPYGVFTINPRTGEINITSVVDREIT
PLFLIYCRALNSRGEDLERPLELRVKVMDINDNAPVFSQSVYTASIEENSDANTLVVKLC
ATDADEENHLNSKIAYKIVSQEPSGAPMFILNRYTGEVCTMSSFLDREQHSMYNLVVRGS
DRDGAADGLSSECDCRIKVLDVNDNFPTLEKTSYSASIEENCLSSELIRLQAIDLDEEGT
DNWLAQYLILSGNDGNWFDIQTDPQTNEGILKVVKMLDYEQAPNIQLSIGVKNQADFHYS
VASQFQMHPTPVRIQVVDVREGPAFHPSTMAFSVREGIKGSSLLNYVLGTYTAIDLDTGN
PATDVRYIIGHDAGSWLKIDSRTGEIQFSREFDKKSKYIINGIYTAEILAIDDGSGKTAT
GTICIEVPDINDYCPNIFPERRTICIDSPSVLISVNEHSYGSPFTFCVVDEPPGIADMWD
VRSTNATSAILTAKQVLSPGFYEIPILVKDSYNRACELAQMVQLYACDCDDNHMCLDSGA
AGIYTEDITGDTYGPVTEDQAGVSNVGLGPAGIGMMVLGILLLILAPLLLLLCCCKQRQP
EGLGTRFAPVPEGGEGVMQSWRIEGAHPEDRDVSNICAPMTASNTQDRMDSSEIYTNTYA
AGGTVEGGVSGVELNTGMGTAVGLMAAGAAGASGAARKRSSTMGTLRDYADADINMAFLD
SYFSEKAYAYADEDEGRPANDCLLIYDHEGVGSPVGSIGCCSWIVDDLDESCMETLDPKF

RTLAEICLNTEIEPFPSHQACIPISTDLPLLGPNYFVNESSGLTPSEVEFQEEMAASEPV
VHGDIIVTETYGNADPCVQPTTIIFDPQLAPNVVVTEAVMAPVYDIQGNICVPAELADYN
NVIYAERVLASPGVPDMSNSSTTEGCMGPVMSGNILVGPEIQVMQMMSPDLPIGQTVGST
SPMTSRHRVTRYSNIHYTQQ
>sp|O43707|ACTN4_HUMAN Alpha-actinin-4 OS=Homo sapiens OX=9606 GN=ACTN4 PE=1 SV=2
MVDYHAANQSYQYGPSSAGNGAGGGGSMGDYMAQEDDWDRDLLLDPAWEKQQRKTFTAWC
NSHLRKAGTQIENIDEDFRDGLKLMLLLEVISGERLPKPERGKMRVHKINNVNKALDFIA
SKGVKLVSIGAEEIVDGNAKMTLGMIWTIILRFAIQDISVEETSAKEGLLLWCQRKTAPY
KNVNVQNFHISWKDGLAFNALIHRHRPELIEYDKLRKDDPVTNLNNAFEVAEKYLDIPKM
LDAEDIVNTARPDEKAIMTYVSSFYHAFSGAQKAETAANRICKVLAVNQENEHLMEDYEK
LASDLLEWIRRTIPWLEDRVPQKTIQEMQQKLEDFRDYRRVHKPPKVQEKCQLEINFNTL
QTKLRLSNRPAFMPSEGKMVSDINNGWQHLEQAEKGYEEWLLNEIRRLERLDHLAEKFRQ
KASIHEAWTDGKEAMLKHRDYETATLSDIKALIRKHEAFESDLAAHQDRVEQIAAIAQEL
NELDYYDSHNVNTRCQKICDQWDALGSLTHSRREALEKTEKQLEAIDQLHLEYAKRAAPF
NNWMESAMEDLQDMFIVHTIEEIEGLISAHDQFKSTLPDADREREAILAIHKEAQRIAES
NHIKLSGSNPYTTVTPQIINSKWEKVQQLVPKRDHALLEEQSKQQSNEHLRRQFASQANV
VGPWIQTKMEEIGRISIEMNGTLEDQLSHLKQYERSIVDYKPNLDLLEQQHQLIQEALIF
DNKHTNYTMEHIRVGWEQLLTTIARTINEVENQILTRDAKGISQEQMQEFRASFNHFDKD
HGGALGPEEFKACLISLGYDVENDRQGEAEFNRIMSLVDPNHSGLVTFQAFIDFMSRETT
DTDTADQVIASFKVLAGDKNFITAEELRRELPPDQAEYCIARMAPYQGPDAVPGALDYKS
FSTALYGESDL
>sp|P62937|PPIA_HUMAN Peptidyl-prolyl cis-trans isomerase A OS=Homo sapiens OX=9606 GN=PPIA PE=1 SV=2
MVNPTVFFDIAVDGEPLGRVSFELFADKVPKTAENFRALSTGEKGFGYKGSCFHRIIPGF
MCQGGDFTRHNGTGGKSIYGEKFEDENFILKHTGPGILSMANAGPNTNGSQFFICTAKTE
WLDGKHVVFGKVKEGMNIVEAMERFGSRNGKTSKKITIADCGQLE
>sp|A0A0B4J2A2|PAL4C_HUMAN Peptidyl-prolyl cis-trans isomerase A-like 4C OS=Homo sapiens OX=9606 GN=PPIAL4C PE=2 SV=1
MVNSVVFFDITVDGKPLGRISIKLFADKIPKTAENFRALSTGEKGFRYKGSCFHRIIPGF
MCQGGDFTRPNGTGDKSIYGEKFDDENLIRKHTGSGILSMANAGPNTNGSQFFICTAKTE
WLDGKHVAFGKVKERVNIVEAMEHFGYRNSKTSKKITIADCGQF
>sp|A0A075B759|PAL4E_HUMAN Peptidyl-prolyl cis-trans isomerase A-like 4E OS=Homo sapiens OX=9606 GN=PPIAL4E PE=3 SV=1
MVNSVVFFEITRDGKPLGRISIKLFADKIPKTAENFRALSTGEKGFRYKGSCFHRIIPGF
MCQGGDFTRPNGTGDKSIYGEKFDDENLIRKHTGSGILSMANAGPNTNGSQFFICAAKTE
WLDGKHVAFGKVKERVNIVEAMEHFGYRNSKTSKKITIADCGQF
>sp|F5H284|PAL4D_HUMAN Peptidyl-prolyl cis-trans isomerase A-like 4D OS=Homo sapiens OX=9606 GN=PPIAL4D PE=3 SV=1
MVNSVVFFEITRDGKPLGRISIKLFADKIPKTAENFRALSTGEKGFRYKGSCFHRIIPGF
MCQGGDFTRPNGTGDKSIYGEKFDDENLIRKHTGSGILSMANAGPNTNGSQFFICAAKTE
WLDGKHVAFGKVKERVNIVEATEHFGYRNSKTSKKITIADCGQF
>sp|P0DN26|PAL4F_HUMAN Peptidyl-prolyl cis-trans isomerase A-like 4F OS=Homo sapiens OX=9606 GN=PPIAL4F PE=3 SV=1
MVNSVVFFEITRDGKPLGRISIKLFADKIPKTAENFRALSTGEKGFRYKGSCFHRIIPGF
MCQGGDFTRPNGTGDKSIYGEKFDDENLIRKHTGSGILSMANAGPNTNGSQFFICAAKTE
WLDGKHVAFGKVKERVNIVEAMEHFGYRNSKTSKKITIADCGQF

>sp|Q9Y536|PAL4A_HUMAN Peptidyl-prolyl cis-trans isomerase A-like 4A OS=Homo sapiens OX=9606 GN=PPIAL4A PE=2 SV=1
MVNSVVFFDITVDGKPLGRISIKLFADKILKTAENFRALSTGEKGFRYKGSCFHRIIPGF
MCQGGDFTRHNGTGDKSIYGEKFDDENLIRKHTGSGILSMANAGPNTNGSQFFICAAKTE
WLDGKHVAFGKVKERVNIVEAMEHFGYRNSKTSKKITIADCGQF
>sp|P04040|CATA_HUMAN Catalase OS=Homo sapiens OX=9606 GN=CAT PE=1 SV=3
MADSRDPASDQMQHWKEQRAAQKADVLTTGAGNPVGDKLNVITVGPRGPLLVQDVVFTDE
MAHFDRERIPERVVHAKGAGAFGYFEVTHDITKYSKAKVFEHIGKKTPIAVRFSTVAGES
GSADTVRDPRGFAVKFYTEDGNWDLVGNNTPIFFIRDPILFPSFIHSQKRNPQTHLKDPD
MVWDFWSLRPESLHQVSFLFSDRGIPDGHRHMNGYGSHTFKLVNANGEAVYCKFHYKTDQ
GIKNLSVEDAARLSQEDPDYGIRDLFNAIATGKYPSWTFYIQVMTFNQAETFPFNPFDLT
KVWPHKDYPLIPVGKLVLNRNPVNYFAEVEQIAFDPSNMPPGIEASPDKMLQGRLFAYPD
THRHRLGPNYLHIPVNCPYRARVANYQRDGPMCMQDNQGGAPNYYPNSFGAPEQQPSALE
HSIQYSGEVRRFNTANDDNVTQVRAFYVNVLNEEQRKRLCENIAGHLKDAQIFIQKKAVK
NFTEVHPDYGSHIQALLDKYNAEKPKNAIHTFVQSGSHLAAREKANL
>sp|P00558|PGK1_HUMAN Phosphoglycerate kinase 1 OS=Homo sapiens OX=9606 GN=PGK1 PE=1 SV=3
MSLSNKLTLDKLDVKGKRVVMRVDFNVPMKNNQITNNQRIKAAVPSIKFCLDNGAKSVVL
MSHLGRPDGVPMPDKYSLEPVAVELKSLLGKDVLFLKDCVGPEVEKACANPAAGSVILLE
NLRFHVEEEGKGKDASGNKVKAEPAKIEAFRASLSKLGDVYVNDAFGTAHRAHSSMVGVN
LPQKAGGFLMKKELNYFAKALESPERPFLAILGGAKVADKIQLINNMLDKVNEMIIGGGM
AFTFLKVLNNMEIGTSLFDEEGAKIVKDLMSKAEKNGVKITLPVDFVTADKFDENAKTGQ
ATVASGIPAGWMGLDCGPESSKKYAEAVTRAKQIVWNGPVGVFEWEAFARGTKALMDEVV
KATSRGCITIIGGGDTATCCAKWNTEDKVSHVSTGGGASLELLEGKVLPGVDALSNI
>sp|P07205|PGK2_HUMAN Phosphoglycerate kinase 2 OS=Homo sapiens OX=9606 GN=PGK2 PE=1 SV=3
MSLSKKLTLDKLDVRGKRVIMRVDFNVPMKKNQITNNQRIKASIPSIKYCLDNGAKAVVL
MSHLGRPDGVPMPDKYSLAPVAVELKSLLGKDVLFLKDCVGAEVEKACANPAPGSVILLE
NLRFHVEEEGKGQDPSGKKIKAEPDKIEAFRASLSKLGDVYVNDAFGTAHRAHSSMVGVN
LPHKASGFLMKKELDYFAKALENPVRPFLAILGGAKVADKIQLIKNMLDKVNEMIIGGGM
AYTFLKVLNNMEIGASLFDEEGAKIVKDIMAKAQKNGVRITFPVDFVTGDKFDENAQVGK
ATVASGISPGWMGLDCGPESNKNHAQVVAQARLIVWNGPLGVFEWDAFAKGTKALMDEIV
KATSKGCITVIGGGDTATCCAKWNTEDKVSHVSTGGGASLELLEGKILPGVEALSNM
>sp|O76013|KRT36_HUMAN Keratin, type I cuticular Ha6 OS=Homo sapiens OX=9606 GN=KRT36 PE=1 SV=1
MATQTCTPTFSTGSIKGLCGTAGGISRVSSIRSVGSCRVPSLAGAAGYISSARSGLSGLG
SCLPGSYLSSECHTSGFVGSGGWFCEGSFNGSEKETMQFLNDRLANYLEKVRQLERENAE
LESRIQEWYEFQIPYICPDYQSYFKTIEDFQQKILLTKSENARLVLQIDNAKLAADDFRT
KYETELSLRQLVEADINGLRRILDELTLCKADLEAQVESLKEELMCLKKNHEEEVSVLRC
QLGDRLNVEVDAAPPVDLNKILEDMRCQYEALVENNRRDVEAWFNTQTEELNQQVVSSSE
QLQCCQTEIIELRRTVNALEIELQAQHSMRNSLESTLAETEARYSSQLAQMQCLISNVEA
QLSEIRCDLERQNQEYQVLLDVKARLEGEIATYRHLLEGEDCKLPPQPCATACKPVIRVP
SVPPVPCVPSVPCTPAPQVGTQIRTITEEIRDGKVISSREHVQSRPL
>sp|Q13228|SBP1_HUMAN Methanethiol oxidase OS=Homo sapiens OX=9606 GN=SELENBP1 PE=1 SV=2
MATKCGNCGPGYSTPLEAMKGPREEIVYLPCIYRNTGTEAPDYLATVDVDPKSPQYCQVI

HRLPMPNLKDELHHSGWNTCSSCFGDSTKSRTKLVLPSLISSRIYVVDVGSEPRAPKLHK
VIEPKDIHAKCELAFLHTSHCLASGEVMISSLGDVKGNGKGGFVLLDGETFEVKGTWERP
GGAAPLGYDFWYQPRHNVMISTEWAAPNVLRDGFNPADVEAGLYGSHLYVWDWQRHEIVQ
TLSLKDGLIPLEIRFLHNPDAAQGFVGCALSSTIQRFYKNEGGTWSVEKVIQVPPKKVKG
WLLPEMPGLITDILLSLDDRFLYFSNWLHGDLRQYDISDPQRPRLTGQLFLGGSIVKGGP
VQVLEDEELKSQPEPLVVKGKRVAGGPQMIQLSLDGKRLYITTSLYSAWDKQFYPDLIRE
GSVMLQVDVDTVKGGLKLNPNFLVDFGKEPLGPALAHELRYPGGDCSSDIWI
>sp|O60437|PEPL_HUMAN Periplakin OS=Homo sapiens OX=9606 GN=PPL PE=1 SV=4
MNSLFRKRNKGKYSPTVQTRSISNKELSELIEQLQKNADQVEKNIVDTEAKMQSDLARLQ
EGRQPEHRDVTLQKVLDSEKLLYVLEADAAIAKHMKHPQGDMIAEDIRQLKERVTNLRGK
HKQIYRLAVKEVDPQVNWAALVEEKLDKLNNQSFGTDLPLVDHQVEEHNIFHNEVKAIGP
HLAKDGDKEQNSELRAKYQKLLAASQARQQHLSSLQDYMQRCTNELYWLDQQAKGRMQYD
WSDRNLDYPSRRRQYENFINRNLEAKEERINKLHSEGDQLLAAEHPGRNSIEAHMEAVHA
DWKEYLNLLICEESHLKYMEDYHQFHEDVKDAQELLRKVDSDLNQKYGPDFKDRYQIELL
LRELDDQEKVLDKYEDVVQGLQKRGQQVVPLKYRRETPLKPIPVEALCDFEGEQGLISRG
YSYTLQKNNGESWELMDSAGNKLIAPAVCFVIPPTDPEALALADSLGSQYRSVRQKAAGS
KRTLQQRYEVLKTENPGDASDLQGRQLLAGLDKVASDLDRQEKAITGILRPPLEQGRAVQ
DSAERAKDLKNITNELLRIEPEKTRSTAEGEAFIQALPGSGTTPLLRTRVEDTNRKYEHL
LQLLDLAQEKVDVANRLEKSLQQSWELLATHENHLNQDDTVPESSRVLDSKGQELAAMAC
ELQAQKSLLGEVEQNLQAAKQCSSTLASRFQEHCPDLERQEAEVHKLGQRFNNLRQQVER
RAQSLQSAKAAYEHFHRGHDHVLQFLVSIPSYEPQETDSLSQMETKLKNQKNLLDEIASR
EQEVQKICANSQQYQQAVKDYELEAEKLRSLLDLENGRRSHVSKRARLQSPATKVKEEEA
ALAAKFTEVYAINRQRLQNLEFALNLLRQQPEVEVTHETLQRNRPDSGVEEAWKIRKELD
EETERRRQLENEVKSTQEEIWTLRNQGPQESVVRKEVLKKVPDPVLEESFQQLQRTLAEE
QHKNQLLQEELEALQLQLRALEQETRDGGQEYVVKEVLRIEPDRAQADEVLQLREELEAL
RRQKGAREAEVLLLQQRVAALAEEKSRAQEKVTEKEVVKLQNDPQLEAEYQQLQEDHQRQ
DQLREKQEEELSFLQDKLKRLEKERAMAEGKITVKEVLKVEKDAATEREVSDLTRQYEDE
AAKARASQREKTELLRKIWALEEENAKVVVQEKVREIVRPDPKAESEVANLRLELVEQER
KYRGAEEQLRSYQSELEALRRRGPQVEVKEVTKEVIKYKTDPEMEKELQRLREEIVDKTR
LIERCDLEIYQLKKEIQALKDTKPQVQTKEVVQEILQFQEDPQTKEEVASLRAKLSEEQK
KQVDLERERASQEEQIARKEEELSRVKERVVQQEVVRYEEEPGLRAEASAFAESIDVELR
QIDKLRAELRRLQRRRTELERQLEELERERQARREAEREVQRLQQRLAALEQEEAEAREK
VTHTQKVVLQQDPQQAREHALLRLQLEEEQHRRQLLEGELETLRRKLAALEKAEVKEKVV
LSESVQVEKGDTEQEIQRLKSSLEEESRSKRELDVEVSRLEARLSELEFHNSKSSKELDF
LREENHKLQLERQNLQLETRRLQSEINMAATETRDLRNMTVADSGTNHDSRLWSLERELD
DLKRLSKDKDLEIDELQKRLGSVAVKREQRENHLRRSIVVIHPDTGRELSPEEAHRAGLI
DWNMFVKLRSQECDWEEISVKGPNGESSVIHDRKSGKKFSIEEALQSGRLTPAQYDRYVN
KDMSIQELAVLVSGQK
>sp|P22735|TGM1_HUMAN Protein-glutamine gamma-glutamyltransferase K OS=Homo sapiens OX=9606 GN=TGM1 PE=1 SV=4
MMDGPRSDVGRWGGNPLQPPTTPSPEPEPEPDGRSRRGGGRSFWARCCGCCSCRNAADDD
WGPEPSDSRGRGSSSGTRRPGSRGSDSRRPVSRGSGVNAAGDGTIREGMLVVNGVDLLSS
RSDQNRREHHTDEYEYDELIVRRGQPFHMLLLLSRTYESSDRITLELLIGNNPEVGKGTH
VIIPVGKGGSGGWKAQVVKASGQNLNLRVHTSPNAIIGKFQFTVRTQSDAGEFQLPFDPR
NEIYILFNPWCPEDIVYVDHEDWRQEYVLNESGRIYYGTEAQIGERTWNYGQFDHGVLDA
CLYILDRRGMPYGGRGDPVNVSRVISAMVNSLDDNGVLIGNWSGDYSRGTNPSAWVGSVE

ILLSYLRTGYSVPYGQCWVFAGVTTTVLRCLGLATRTVTNFNSAHDTDTSLTMDIYFDEN
MKPLEHLNHDSVWNFHVWNDCWMKRPDLPSGFDGWQVVDATPQETSSGIFCCGPCSVESI
KNGLVYMKYDTPFIFAEVNSDKVYWQRQDDGSFKIVYVEEKAIGTLIVTKAISSNMREDI
TYLYKHPEGSDAERKAVETAAAHGSKPNVYANRGSAEDVAMQVEAQDAVMGQDLMVSVML
INHSSSRRTVKLHLYLSVTFYTGVSGTIFKETKKEVELAPGASDRVTMPVAYKEYRPHLV
DQGAMLLNVSGHVKESGQVLAKQHTFRLRTPDLSLTLLGAAVVGQECEVQIVFKNPLPVT
LTNVVFRLEGSGLQRPKILNVGDIGGNETVTLRQSFVPVRPGPRQLIASLDSPQLSQVHG
VIQVDVAPAPGDGGFFSDAGGDSHLGETIPMASRGGA
>sp|Q92817|EVPL_HUMAN Envoplakin OS=Homo sapiens OX=9606 GN=EVPL PE=1 SV=3
MFKGLSKGSQGKGSPKGSPAKGSPKGSPSRHSRAATQELALLISRMQANADQVERDILET
QKRLQQDRLNSEQSQALQHQQETGRSLKEAEVLLKDLFLDVDKARRLKHPQAEEIEKDIK
QLHERVTQECAEYRALYEKMVLPPDVGPRVDWARVLEQKQKQVCAGQYGPGMAELEQQIA
EHNILQKEIDAYGQQLRSLVGPDAATIRSQYRDLLKAASWRGQSLGSLYTHLQGCTRQLS
ALAEQQRRILQQDWSDLMADPAGVRREYEHFKQHELLSQEQSVNQLEDDGERMVELRHPA
VGPIQAHQEALKMEWQNFLNLCICQETQLQHVEDYRRFQEEADSVSQTLAKLNSNLDAKY
SPAPGGPPGAPTELLQQLEAEEKRLAVTERATGDLQRRSRDVAPLPQRRNPPQQPLHVDS
ICDWDSGEVQLLQGERYKLVDNTDPHAWVVQGPGGETKRAPAACFCIPAPDPDAVARASR
LASELQALKQKLATVQSRLKASAVESLRPSQQAPSGSDLANPQAQKLLTQMTRLDGDLGQ
IERQVLAWARAPLSRPTPLEDLEGRIHSHEGTAQRLQSLGTEKETAQKECEAFLSTRPVG
PAALQLPVALNSVKNKFSDVQVLCSLYGEKAKAALDLERQIQDADRVIRGFEATLVQEAP
IPAEPGALQERVSELQRQRRELLEQQTCVLRLHRALKASEHACAALQNNFQEFCQDLPRQ
QRQVRALTDRYHAVGDQLDLREKVVQDAALTYQQFKNCKDNLSSWLEHLPRSQVRPSDGP
SQIAYKLQAQKRLTQEIQSRERDRATASHLSQALQAALQDYELQADTYRCSLEPTLAVSA
PKRPRVAPLQESIQAQEKNLAKAYTEVAAAQQQLLQQLEFARKMLEKKELSEDIRRTHDA
KQGSESPAQAGRESEALKAQLEEERKRVARVQHELEAQRSQLLQLRTQRPLERLEEKEVV
EFYRDPQLEGSLSRVKAQVEEEGKRRAGLQADLEVAAQKVVQLESKRKTMQPHLLTKEVT
QVERDPGLDSQAAQLRIQIQQLRGEDAVISARLEGLKKELLALEKREVDVKEKVVVKEVV
KVEKNLEMVKAAQALRLQMEEDAARRKQAEEAVAKLQARIEDLERAISSVEPKVIVKEVK
KVEQDPGLLQESSRLRSLLEEERTKNATLARELSDLHSKYSVVEKQRPKVQLQERVHEIF
QVDPETEQEITRLKAKLQEMAGKRSGVEKEVEKLLPDLEVLRAQKPTVEYKEVTQEVVRH
ERSPEVLREIDRLKAQLNELVNSHGRSQEQLIRLQGERDEWRRERAKVETKTVSKEVVRH
EKDPVLEKEAERLRQEVREAAQKRRAAEDAVYELQSKRLLLERRKPEEKVVVQEVVVTQK
DPKLREEHSRLSGSLDEEVGRRRQLELEVQQLRAGVEEQEGLLSFQEDRSKKLAVERELR
QLTLRIQELEKRPPTVQEKIIMEEVVKLEKDPDLEKSTEALRWDLDQEKTQVTELNRECK
NLQVQIDVLQKAKSQEKTIYKEVIRVQKDRVLEDERARVWEMLNRERTARQAREEEARRL
RERIDRAETLGRTWSREESELQRARDQADQECGRLQQELRALERQKQQQTLQLQEESKLL
SQKTESERQKAAQRGQELSRLEAAILREKDQIYEKERTLRDLHAKVSREELSQETQTRET
NLSTKISILEPETGKDMSPYEAYKRGIIDRGQYLQLQELECDWEEVTTSGPCGEESVLLD
RKSGKQYSIEAALRCRRISKEEYHLYKDGHLPISEFALLVAGETKPSSSLSIGSIISKSP
LASPAPQSTSFFSPSFSLGLGDDSFPIAGIYDTTTDNKCSIKTAVAKNMLDPITGQKLLE
AQAATGGIVDLLSRERYSVHKAMERGLIENTSTQRLLNAQKAFTGIEDPVTKKRLSVGEA
VQKGWMPRESVLPHLQVQHLTGGLIDPKRTGRIPIQQALLSGMISEELAQLLQDESSYEK
DLTDPISKERLSYKEAMGRCRKDPLSGLLLLPAALEGYRCYRSASPTVPRSLR
>sp|O75369|FLNB_HUMAN Filamin-B OS=Homo sapiens OX=9606 GN=FLNB PE=1 SV=2
MPVTEKDLAEDAPWKKIQQNTFTRWCNEHLKCVNKRIGNLQTDLSDGLRLIALLEVLSQK

RMYRKYHQRPTFRQMQLENVSVALEFLDRESIKLVSIDSKAIVDGNLKLILGLVWTLILH
YSISMPVWEDEGDDDAKKQTPKQRLLGWIQNKIPYLPITNFNQNWQDGKALGALVDSCAP
GLCPDWESWDPQKPVDNAREAMQQADDWLGVPQVITPEEIIHPDVDEHSVMTYLSQFPKA
KLKPGAPLKPKLNPKKARAYGRGIEPTGNMVKQPAKFTVDTISAGQGDVMVFVEDPEGNK
EEAQVTPDSDKNKTYSVEYLPKVTGLHKVTVLFAGQHISKSPFEVSVDKAQGDASKVTAK
GPGLEAVGNIANKPTYFDIYTAGAGVGDIGVEVEDPQGKNTVELLVEDKGNQVYRCVYKP
MQPGPHVVKIFFAGDTIPKSPFVVQVGEACNPNACRASGRGLQPKGVRIRETTDFKVDTK
AAGSGELGVTMKGPKGLEELVKQKDFLDGVYAFEYYPSTPGRYSIAITWGGHHIPKSPFE
VQVGPEAGMQKVRAWGPGLHGGIVGRSADFVVESIGSEVGSLGFAIEGPSQAKIEYNDQN
DGSCDVKYWPKEPGEYAVHIMCDDEDIKDSPYMAFIHPATGGYNPDLVRAYGPGLEKSGC
IVNNLAEFTVDPKDAGKAPLKIFAQDGEGQRIDIQMKNRMDGTYACSYTPVKAIKHTIAV
VWGGVNIPHSPYRVNIGQGSHPQKVKVFGPGVERSGLKANEPTHFTVDCTEAGEGDVSVG
IKCDARVLSEDEEDVDFDIIHNANDTFTVKYVPPAAGRYTIKVLFASQEIPASPFRVKVD
PSHDASKVKAEGPGLSKAGVENGKPTHFTVYTKGAGKAPLNVQFNSPLPGDAVKDLDIID
NYDYSHTVKYTPTQQGNMQVLVTYGGDPIPKSPFTVGVAAPLDLSKIKLNGLENRVEVGK
DQEFTVDTRGAGGQGKLDVTILSPSRKVVPCLVTPVTGRENSTAKFIPREEGLYAVDVTY
DGHPVPGSPYTVEASLPPDPSKVKAHGPGLEGGLVGKPAEFTIDTKGAGTGGLGLTVEGP
CEAKIECSDNGDGTCSVSYLPTKPGEYFVNILFEEVHIPGSPFKADIEMPFDPSKVVASG
PGLEHGKVGEAGLLSVDCSEAGPGALGLEAVSDSGTKAEVSIQNNKDGTYAVTYVPLTAG
MYTLTMKYGGELVPHFPARVKVEPAVDTSRIKVFGPGIEGKDVFREATTDFTVDSRPLTQ
VGGDHIKAHIANPSGASTECFVTDNADGTYQVEYTPFEKGLHVVEVTYDDVPIPNSPFKV
AVTEGCQPSRVQAQGPGLKEAFTNKPNVFTVVTRGAGIGGLGITVEGPSESKINCRDNKD
GSCSAEYIPFAPGDYDVNITYGGAHIPGSPFRVPVKDVVDPSKVKIAGPGLGSGVRARVL
QSFTVDSSKAGLAPLEVRVLGPRGLVEPVNVVDNGDGTHTVTYTPSQEGPYMVSVKYADE
EIPRSPFKVKVLPTYDASKVTASGPGLSSYGVPASLPVDFAIDARDAGEGLLAVQITDQE
GKPKRAIVHDNKDGTYAVTYIPDKTGRYMIGVTYGGDDIPLSPYRIRATQTGDASKCLAT
GPGIASTVKTGEEVGFVVDAKTAGKGKVTCTVLTPDGTEAEADVIENEDGTYDIFYTAAK
PGTYVIYVRFGGVDIPNSPFTVMATDGEVTAVEEAPVNACPPGFRPWVTEEAYVPVSDMN
GLGFKPFDLVIPFAVRKGEITGEVHMPSGKTATPEIVDNKDGTVTVRYAPTEVGLHEMHI
KYMGSHIPESPLQFYVNYPNSGSVSAYGPGLVYGVANKTATFTIVTEDAGEGGLDLAIEG
PSKAEISCIDNKDGTCTVTYLPTLPGDYSILVKYNDKHIPGSPFTAKITDDSRRCSQVKL
GSAADFLLDISETDLSSLTASIKAPSGRDEPCLLKRLPNNHIGISFIPREVGEHLVSIKK
NGNHVANSPVSIMVVQSEIGDARRAKVYGRGLSEGRTFEMSDFIVDTRDAGYGGISLAVE
GPSKVDIQTEDLEDGTCKVSYFPTVPGVYIVSTKFADEHVPGSPFTVKISGEGRVKESIT
RTSRAPSVATVGSICDLNLKIPEINSSDMSAHVTSPSGRVTEAEIVPMGKNSHCVRFVPQ
EMGVHTVSVKYRGQHVTGSPFQFTVGPLGEGGAHKVRAGGPGLERGEAGVPAEFSIWTRE
AGAGGLSIAVEGPSKAEITFDDHKNGSCGVSYIAQEPGNYEVSIKFNDEHIPESPYLVPV
IAPSDDARRLTVMSLQESGLKVNQPASFAIRLNGAKGKIDAKVHSPSGAVEECHVSELEP
DKYAVRFIPHENGVHTIDVKFNGSHVVGSPFKVRVGEPGQAGNPALVSAYGTGLEGGTTG
IQSEFFINTTRAGPGTLSVTIEGPSKVKMDCQETPEGYKVMYTPMAPGNYLISVKYGGPN
HIVGSPFKAKVTGQRLVSPGSANETSSILVESVTRSSTETCYSAIPKASSDASKVTSKGA
GLSKAFVGQKSSFLVDCSKAGSNMLLIGVHGPTTPCEEVSMKHVGNQQYNVTYVVKERGD
YVLAVKWGEEHIPGSPFHVTVP
>sp|P63104|1433Z_HUMAN 14-3-3 protein zeta/delta OS=Homo sapiens OX=9606 GN=YWHAZ PE=1 SV=1
MDKNELVQKAKLAEQAERYDDMAACMKSVTEQGAELSNEERNLLSVAYKNVVGARRSSWR
VVSSIEQKTEGAEKKQQMAREYREKIETELRDICNDVLSLLEKFLIPNASQAESKVFYLK

MKGDYYRYLAEVAAGDDKKGIVDQSQQAYQEAFEISKKEMQPTHPIRLGLALNFSVFYYE
ILNSPEKACSLAKTAFDEAIAELDTLSEESYKDSTLIMQLLRDNLTLWTSDTQGDEAEAG
EGGEN
>sp|P31944|CASPE_HUMAN Caspase-14 OS=Homo sapiens OX=9606 GN=CASP14 PE=1 SV=2
MSNPRSLEEEKYDMSGARLALILCVTKAREGSEEDLDALEHMFRQLRFESTMKRDPTAEQ
FQEELEKFQQAIDSREDPVSCAFVVLMAHGREGFLKGEDGEMVKLENLFEALNNKNCQAL
RAKPKVYIIQACRGEQRDPGETVGGDEIVMVIKDSPQTIPTYTDALHVYSTVEGYIAYRH
DQKGSCFIQTLVDVFTKRKGHILELLTEVTRRMAEAELVQEGKARKTNPEIQSTLRKRLY
LQ
>sp|Q9UIV8|SPB13_HUMAN Serpin B13 OS=Homo sapiens OX=9606 GN=SERPINB13 PE=1 SV=2
MDSLGAVSTRLGFDLFKELKKTNDGNIFFSPVGILTAIGMVLLGTRGATASQLEEVFHSE
KETKSSRIKAEEKEVIENTEAVHQQFQKFLTEISKLTNDYELNITNRLFGEKTYLFLQKY
LDYVEKYYHASLEPVDFVNAADESRKKINSWVESKTNEKIKDLFPDGSISSSTKLVLVNM
VYFKGQWDREFKKENTKEEKFWMNKSTSKSVQMMTQSHSFSFTFLEDLQAKILGIPYKNN
DLSMFVLLPNDIDGLEKIIDKISPEKLVEWTSPGHMEERKVNLHLPRFEVEDGYDLEAVL
AAMGMGDAFSEHKADYSGMSSGSGLYAQKFLHSSFVAVTEEGTEAAAATGIGFTVTSAPG
HENVHCNHPFLFFIRHNESNSILFFGRFSSP
>sp|P05120|PAI2_HUMAN Plasminogen activator inhibitor 2 OS=Homo sapiens OX=9606 GN=SERPINB2 PE=1 SV=2
MEDLCVANTLFALNLFKHLAKASPTQNLFLSPWSISSTMAMVYMGSRGSTEDQMAKVLQF
NEVGANAVTPMTPENFTSCGFMQQIQKGSYPDAILQAQAADKIHSSFRSLSSAINASTGN
YLLESVNKLFGEKSASFREEYIRLCQKYYSSEPQAVDFLECAEEARKKINSWVKTQTKGK
IPNLLPEGSVDGDTRMVLVNAVYFKGKWKTPFEKKLNGLYPFRVNSAQRTPVQMMYLREK
LNIGYIEDLKAQILELPYAGDVSMFLLLPDEIADVSTGLELLESEITYDKLNKWTSKDKM
AEDEVEVYIPQFKLEEHYELRSILRSMGMEDAFNKGRANFSGMSERNDLFLSEVFHQAMV
DVNEEGTEAAAGTGGVMTGRTGHGGPQFVADHPFLFLIMHKITNCILFFGRFSSP
>sp|P04075|ALDOA_HUMAN Fructose-bisphosphate aldolase A OS=Homo sapiens OX=9606 GN=ALDOA PE=1 SV=2
MPYQYPALTPEQKKELSDIAHRIVAPGKGILAADESTGSIAKRLQSIGTENTEENRRFYR
QLLLTADDRVNPCIGGVILFHETLYQKADDGRPFPQVIKSKGGVVGIKVDKGVVPLAGTN
GETTTQGLDGLSERCAQYKKDGADFAKWRCVLKIGEHTPSALAIMENANVLARYASICQQ
NGIVPIVEPEILPDGDHDLKRCQYVTEKVLAAVYKALSDHHIYLEGTLLKPNMVTPGHAC
TQKFSHEEIAMATVTALRRTVPPAVTGITFLSGGQSEEEASINLNAINKCPLLKPWALTF
SYGRALQASALKAWGGKKENLKAAQEEYVKRALANSLACQGKYTPSGQAGAAASESLFVS
NHAY
>sp|Q8WVV4|POF1B_HUMAN Protein POF1B OS=Homo sapiens OX=9606 GN=POF1B PE=1 SV=3
MSSSYWSETSSSSCGTQQLPEVLQCQPQHYHCYHQSSQAQQPPEKNVVYERVRTYSGPMN
KVVQALDPFNSREVLSPLKTTSSYQNLVWSDHSQELHSPTLKISTCAPSTLHITQNTEQE
LHSPTVKLTTYPQTTIRKYVVQNPEQEPLSQFLRGSHFFPGNNVIYEKTIRKVEKLNTDQ
GCHPQAQCHHHIIQQPQVIHSAHWQQPDSSQQIQAITGNNPISTHIGNELCHSGSSQICE
QVIIQDDGPEKLDPRYFGELLADLSRKNTDLYHCLLEHLQRIGGSKQDFESTDESEDIES
LIPKGLSEFTKQQIRYILQMRGMSDKSLRLVLSTFSNIREELGHLQNDMTSLENDKMRLE
KDLSFKDTQLKEYEELLASVRANNHQQQQGLQDSSSKCQALEENNLSLRHTLSDMEYRLK
ELEYCKRNLEQENQNLRMQVSETCTGPMLQAKMDEIGNHYTEMVKNLRMEKDREICRLRS

QLNQYHKDVSKREGSCSDFQFKLHELTSLLEEKDSLIKRQSEELSKLRQEIYSSHNQPST
GGRTTITTKKYRTQYPILGLLYDDYEYIPPGSETQTIVIEKTEDKYTCP
>sp|Q01546|K22O_HUMAN Keratin, type II cytoskeletal 2 oral OS=Homo sapiens OX=9606 GN=KRT76 PE=1 SV=2
MNRQVCKKSFSGRSQGFSGRSAVVSGSSRMSCVARSGGAGGGACGFRSGAGSFGSRSLYN
LGSNKSISISVAAGSSRAGGFGGGRSSCGFAGGYGGGFGGSYGGGFGGGRGVGSGFGGAG
GFGGAGGFGGPGVFGGPGSFGGPGGFGPGGFPGGIQEVIVNQSLLQPLNVEIDPQIGQVK
AQEREQIKTLNNKFASFIDKVRFLEQQNKVLETKWELLQQQTTGSGPSSLEPCFESYISF
LCKQLDSLLGERGNLEGELKSMQDLVEDFKKKYEDEINKRTAAENEFVGLKKDVDAAFMN
KVELQAKVDSLTDEVSFLRTLYEMELSQMQSHASDTSVVLSMDNNRCLDLGSIIAEVRAQ
YEEIAQRSKSEAEALYQTKLGELQTTAGRHGDDLRNTKSEIMELNRMIQRLRAEIENVKK
QNANLQTAIAEAEQRGEMALKDANAKLQDLQTALQKAKDDLARLLRDYQELMNVKLALDV
EIATYRKLLEGEECRMSGECQSAVCISVVSNVTSTSGSSGSSRGVFGGVSGSGSGGYKGG
SSSSSSSGYGVSGGSGSGYGGVSSGSTGGRGSSGSYQSSSSGSRLGGAGSISVSHSGMGS
SSGSIQTSGGSGYKSGGGGSTSIRFSQTTSSSQHSSTK
>sp|P09211|GSTP1_HUMAN Glutathione S-transferase P OS=Homo sapiens OX=9606 GN=GSTP1 PE=1 SV=2
MPPYTVVYFPVRGRCAALRMLLADQGQSWKEEVVTVETWQEGSLKASCLYGQLPKFQDGD
LTLYQSNTILRHLGRTLGLYGKDQQEAALVDMVNDGVEDLRCKYISLIYTNYEAGKDDYV
KALPGQLKPFETLLSQNQGGKTFIVGDQISFADYNLLDLLLIHEVLAPGCLDAFPLLSAY
VGRLSARPKLKAFLASPEYVNLPINGNGKQ
>sp|A8K2U0|A2ML1_HUMAN Alpha-2-macroglobulin-like protein 1 OS=Homo sapiens OX=9606 GN=A2ML1 PE=1 SV=3
MWAQLLLGMLALSPAIAEELPNYLVTLPARLNFPSVQKVCLDLSPGYSDVKFTVTLETKD
KTQKLLEYSGLKKRHLHCISFLVPPPAGGTEEVATIRVSGVGNNISFEEKKKVLIQRQGN
GTFVQTDKPLYTPGQQVYFRIVTMDSNFVPVNDKYSMVELQDPNSNRIAQWLEVVPEQGI
VDLSFQLAPEAMLGTYTVAVAEGKTFGTFSVEEYVLPKFKVEVVEPKELSTVQESFLVKI
CCRYTYGKPMLGAVQVSVCQKANTYWYREVEREQLPDKCRNLSGQTDKTGCFSAPVDMAT
FDLIGYAYSHQINIVATVVEEGTGVEANATQNIYISPQMGSMTFEDTSNFYHPNFPFSGK
IRVRGHDDSFLKNHLVFLVIYGTNGTFNQTLVTDNNGLAPFTLETSGWNGTDVSLEGKFQ
MEDLVYNPEQVPRYYQNAYLHLRPFYSTTRSFLGIHRLNGPLKCGQPQEVLVDYYIDPAD
ASPDQEISFSYYLIGKGSLVMEGQKHLNSKKKGLKASFSLSLTFTSRLAPDPSLVIYAIF
PSGGVVADKIQFSVEMCFDNQVSLGFSPSQQLPGAEVELQLQAAPGSLCALRAVDESVLL
LRPDRELSNRSVYGMFPFWYGHYPYQVAEYDQCPVSGPWDFPQPLIDPMPQGHSSQRSII
WRPSFSEGTDLFSFFRDVGLKILSNAKIKKPVDCSHRSPEYSTAMGAGGGHPEAFESSTP
LHQAEDSQVRQYFPETWLWDLFPIGNSGKEAVHVTVPDAITEWKAMSFCTSQSRGFGLSP
TVGLTAFKPFFVDLTLPYSVVRGESFRLTATIFNYLKDCIRVQTDLAKSHEYQLESWADS
QTSSCLCADDAKTHHWNITAVKLGHINFTISTKILDSNEPCGGQKGFVPQKGRSDTLIKP
VLVKPEGVLVEKTHSSLLCPKGKVASESVSLELPVDIVPDSTKAYVTVLGDIMGTALQNL
DGLVQMPSGCGEQNMVLFAPIIYVLQYLEKAGLLTEEIRSRAVGFLEIGYQKELMYKHSN
GSYSAFGERDGNGNTWLTAFVTKCFGQAQKFIFIDPKNIQDALKWMAGNQLPSGCYANVG
NLLHTAMKGGVDDEVSLTAYVTAALLEMGKDVDDPMVSQGLRCLKNSATSTTNLYTQALL
AYIFSLAGEMDIRNILLKQLDQQAIISGESIYWSQKPTPSSNASPWSEPAAVDVELTAYA
LLAQLTKPSLTQKEIAKATSIVAWLAKQHNAYGGFSSTQDTVVALQALAKYATTAYMPSE
EINLVVKSTENFQRTFNIQSVNRLVFQQDTLPNVPGMYTLEASGQGCVYVQTVLRYNILP
PTNMKTFSLSVEIGKARCEQPTSPRSLTLTIHTSYVGSRSSSNMAIVEVKMLSGFSPMEG
TNQLLLQQPLVKKVEFGTDTLNIYLDELIKNTQTYTFTISQSVLVTNLKPATIKVYDYYL

PDEQATIQYSDPCE
>sp|Q5XKE5|K2C79_HUMAN Keratin, type II cytoskeletal 79 OS=Homo sapiens OX=9606 GN=KRT79 PE=1 SV=2
MRSSVSRQTYSTKGGFSSNSASGGSGSQARTSFSSVTVSRSSGSGGGAHCGPGTGGFGSR
SLYNLGGHKSISVSVAGGALLGRALGGFGFGSRAFMGQGAGRQTFGPACPPGGIQEVTVN
QSLLTPLHVEIDPEIQRVRTQEREQIKTLNNKFASFIDKVRFLEQQNKVLETKWALLQEQ
GQNLGVTRNNLEPLFEAYLGSMRSTLDRLQSERGRLDSELRNVQDLVEDFKNKYEDEINK
HTAAENEFVVLKKDVDAAYMGRMDLHGKVGTLTQEIDFLQQLYEMELSQVQTHVSNTNVV
LSMDNNRNLDLDSIIAEVKAQYELIAQRSRAEAEAWYQTKYEELQVTAGKHGDNLRDTKN
EIAELTRTIQRLQGEADAAKKQCQQLQTAIAEAEQRGELALKDAQKKLGDLDVALHQAKE
DLTRLLRDYQELMNVKLALDVEIATYRKLLESEESRMSGECPSAVSISVTGNSTTVCGGG
AASFGGGISLGGSGGATKGGFSTNVGYSTVKGGPVSAGTSILRKTTTVKTSSQRY
>sp|P19012|K1C15_HUMAN Keratin, type I cytoskeletal 15 OS=Homo sapiens OX=9606 GN=KRT15 PE=1 SV=3
MTTTFLQTSSSTFGGGSTRGGSLLAGGGGFGGGSLSGGGGSRSISASSARFVSSGSGGGY
GGGMRVCGFGGGAGSVFGGGFGGGVGGGFGGGFGGGDGGLLSGNEKITMQNLNDRLASYL
DKVRALEEANADLEVKIHDWYQKQTPTSPECDYSQYFKTIEELRDKIMATTIDNSRVILE
IDNARLAADDFRLKYENELALRQGVEADINGLRRVLDELTLARTDLEMQIEGLNEELAYL
KKNHEEEMKEFSSQLAGQVNVEMDAAPGVDLTRVLAEMREQYEAMAEKNRRDVEAWFFSK
TEELNKEVASNTEMIQTSKTEITDLRRTMQELEIELQSQLSMKAGLENSLAETECRYATQ
LQQIQGLIGGLEAQLSELRCEMEAQNQEYKMLLDIKTRLEQEIATYRSLLEGQDAKMAGI
AIREASSGGGGSSSNFHINVEESVDGQVVSSHKREI
>sp|P49327|FAS_HUMAN Fatty acid synthase OS=Homo sapiens OX=9606 GN=FASN PE=1 SV=3
MEEVVIAGMSGKLPESENLQEFWDNLIGGVDMVTDDDRRWKAGLYGLPRRSGKLKDLSRF
DASFFGVHPKQAHTMDPQLRLLLEVTYEAIVDGGINPDSLRGTHTGVWVGVSGSETSEAL
SRDPETLVGYSMVGCQRAMMANRLSFFFDFRGPSIALDTACSSSLMALQNAYQAIHSGQC
PAAIVGGINVLLKPNTSVQFLRLGMLSPEGTCKAFDTAGNGYCRSEGVVAVLLTKKSLAR
RVYATILNAGTNTDGFKEQGVTFPSGDIQEQLIRSLYQSAGVAPESFEYIEAHGTGTKVG
DPQELNGITRALCATRQEPLLIGSTKSNMGHPEPASGLAALAKVLLSLEHGLWAPNLHFH
SPNPEIPALLDGRLQVVDQPLPVRGGNVGINSFGFGGSNVHIILRPNTQPPPAPAPHATL
PRLLRASGRTPEAVQKLLEQGLRHSQDLAFLSMLNDIAAVPATAMPFRGYAVLGGERGGP
EVQQVPAGERPLWFICSGMGTQWRGMGLSLMRLDRFRDSILRSDEAVKPFGLKVSQLLLS
TDESTFDDIVHSFVSLTAIQIGLIDLLSCMGLRPDGIVGHSLGEVACGYADGCLSQEEAV
LAAYWRGQCIKEAHLPPGAMAAVGLSWEECKQRCPPGVVPACHNSKDTVTISGPQAPVFE
FVEQLRKEGVFAKEVRTGGMAFHSYFMEAIAPPLLQELKKVIREPKPRSARWLSTSIPEA
QWHSSLARTSSAEYNVNNLVSPVLFQEALWHVPEHAVVLEIAPHALLQAVLKRGLKPSCT
IIPLMKKDHRDNLEFFLAGIGRLHLSGIDANPNALFPPVEFPAPRGTPLISPLIKWDHSL
AWDVPAAEDFPNGSGSPSAAIYNIDTSSESPDHYLVDHTLDGRVLFPATGYLSIVWKTLA
RALGLGVEQLPVVFEDVVLHQATILPKTGTVSLEVRLLEASRAFEVSENGNLVVSGKVYQ
WDDPDPRLFDHPESPTPNPTEPLFLAQAEVYKELRLRGYDYGPHFQGILEASLEGDSGRL
LWKDNWVSFMDTMLQMSILGSAKHGLYLPTRVTAIHIDPATHRQKLYTLQDKAQVADVVV
SRWLRVTVAGGVHISGLHTESAPRRQQEQQVPILEKFCFTPHTEEGCLSERAALQEELQL
CKGLVQALQTKVTQQGLKMVVPGLDGAQIPRDPSQQELPRLLSAACRLQLNGNLQLELAQ
VLAQERPKLPEDPLLSGLLDSPALKACLDTAVENMPSLKMKVVEVLAGHGHLYSRIPGLL
SPHPLLQLSYTATDRHPQALEAAQAELQQHDVAQGQWDPADPAPSALGSADLLVCNCAVA
ALGDPASALSNMVAALREGGFLLLHTLLRGHPLGDIVAFLTSTEPQYGQGILSQDAWESL

FSRVSLRLVGLKKSFYGSTLFLCRRPTPQDSPIFLPVDDTSFRWVESLKGILADEDSSRP
VWLKAINCATSGVVGLVNCLRREPGGNRLRCVLLSNLSSTSHVPEVDPGSAELQKVLQGD
LVMNVYRDGAWGAFRHFLLEEDKPEEPTAHAFVSTLTRGDLSSIRWVCSSLRHAQPTCPG
AQLCTVYYASLNFRDIMLATGKLSPDAIPGKWTSQDSLLGMEFSGRDASGKRVMGLVPAK
GLATSVLLSPDFLWDVPSNWTLEEAASVPVVYSTAYYALVVRGRVRPGETLLIHSGSGGV
GQAAIAIALSLGCRVFTTVGSAEKRAYLQARFPQLDSTSFANSRDTSFEQHVLWHTGGKG
VDLVLNSLAEEKLQASVRCLATHGRFLEIGKFDLSQNHPLGMAIFLKNVTFHGVLLDAFF
NESSADWREVWALVQAGIRDGVVRPLKCTVFHGAQVEDAFRYMAQGKHIGKVVVQVLAEE
PEAVLKGAKPKLMSAISKTFCPAHKSYIIAGGLGGFGLELAQWLIQRGVQKLVLTSRSGI
RTGYQAKQVRRWRRQGVQVQVSTSNISSLEGARGLIAEAAQLGPVGGVFNLAVVLRDGLL
ENQTPEFFQDVCKPKYSGTLNLDRVTREACPELDYFVVFSSVSCGRGNAGQSNYGFANSA
MERICEKRRHEGLPGLAVQWGAIGDVGILVETMSTNDTIVSGTLPQRMASCLEVLDLFLN
QPHMVLSSFVLAEKAAAYRDRDSQRDLVEAVAHILGIRDLAAVNLDSSLADLGLDSLMSV
EVRQTLERELNLVLSVREVRQLTLRKLQELSSKADEASELACPTPKEDGLAQQQTQLNLR
SLLVNPEGPTLMRLNSVQSSERPLFLVHPIEGSTTVFHSLASRLSIPTYGLQCTRAAPLD
SIHSLAAYYIDCIRQVQPEGPYRVAGYSYGACVAFEMCSQLQAQQSPAPTHNSLFLFDGS
PTYVLAYTQSYRAKLTPGCEAEAETEAICFFVQQFTDMEHNRVLEALLPLKGLEERVAAA
VDLIIKSHQGLDRQELSFAARSFYYKLRAAEQYTPKAKYHGNVMLLRAKTGGAYGEDLGA
DYNLSQVCDGKVSVHVIEGDHRTLLEGSGLESIISIIHSSLAEPRVSVREG
>sp|Q9Y446|PKP3_HUMAN Plakophilin-3 OS=Homo sapiens OX=9606 GN=PKP3 PE=1 SV=1
MQDGNFLLSALQPEAGVCSLALPSDLQLDRRGAEGPEAERLRAARVQEQVRARLLQLGQQ
PRHNGAAEPEPEAETARGTSRGQYHTLQAGFSSRSQGLSGDKTSGFRPIAKPAYSPASWS
SRSAVDLSCSRRLSSAHNGGSAFGAAGYGGAQPTPPMPTRPVSFHERGGVGSRADYDTLS
LRSLRLGPGGLDDRYSLVSEQLEPAATSTYRAFAYERQASSSSSRAGGLDWPEATEVSPS
RTIRAPAVRTLQRFQSSHRSRGVGGAVPGAVLEPVARAPSVRSLSLSLADSGHLPDVHGF
NSYGSHRTLQRLSSGFDDIDLPSAVKYLMASDPNLQVLGAAYIQHKCYSDAAAKKQARSL
QAVPRLVKLFNHANQEVQRHATGAMRNLIYDNADNKLALVEENGIFELLRTLREQDDELR
KNVTGILWNLSSSDHLKDRLARDTLEQLTDLVLSPLSGAGGPPLIQQNASEAEIFYNATG
FLRNLSSASQATRQKMRECHGLVDALVTSINHALDAGKCEDKSVENAVCVLRNLSYRLYD
EMPPSALQRLEGRGRRDLAGAPPGEVVGCFTPQSRRLRELPLAADALTFAEVSKDPKGLE
WLWSPQIVGLYNRLLQRCELNRHTTEAAAGALQNITAGDRRWAGVLSRLALEQERILNPL
LDRVRTADHHQLRSLTGLIRNLSRNARNKDEMSTKVVSHLIEKLPGSVGEKSPPAEVLVN
IIAVLNNLVVASPIAARDLLYFDGLRKLIFIKKKRDSPDSEKSSRAASSLLANLWQYNKL
HRDFRAKGYRKEDFLGP
>sp|Q14CN4|K2C72_HUMAN Keratin, type II cytoskeletal 72 OS=Homo sapiens OX=9606 GN=KRT72 PE=1 SV=2
MSRQLTHFPRGERLGFSGCSAVLSGGIGSSSASFRARVKGSASFGSKSLSCLGGSRSLAL
SAAARRGGGRLGGFVGTAFGSAGLGPKCPSVCPPGGIPQVTVNKSLLAPLNVEMDPEIQR
VRAQEREQIKALNNKFASFIDKVRFLEQQNQVLETKWNLLQQLDLNNCRKNLEPIYEGYI
SNLQKQLEMLSGDGVRLDSELRNMQDLVEDYKKRYEVEINRRTAAENEFVVLKKDVDAAY
MNKVELQAKVDSLTDEIKFFKCLYEGEITQIQSHISDTSIVLSMDNNRDLDLDSIIAEVR
AQYEEIALKSKAEAETLYQTKIQELQVTAGQHGDDLKLTKAEISELNRLIQRIRSEIGNV
KKQCADLETAIADAEQRGDCALKDARAKLDELEGALHQAKEELARMLREYQELVSLKLAL
DMEIATYRKLLESEECRMSGEYPNSVSISVISSTNAGAGGAGFSMGFGASSSYSYKTAAA
DVKTKGSCGSELKDPLAKTSGSSCATKKASR

>sp|Q00610|CLH1_HUMAN Clathrin heavy chain 1 OS=Homo sapiens OX=9606 GN=CLTC PE=1 SV=5
MAQILPIRFQEHLQLQNLGINPANIGFSTLTMESDKFICIREKVGEQAQVVIIDMNDPSN
PIRRPISADSAIMNPASKVIALKAGKTLQIFNIEMKSKMKAHTMTDDVTFWKWISLNTVA
LVTDNAVYHWSMEGESQPVKMFDRHSSLAGCQIINYRTDAKQKWLLLTGISAQQNRVVGA
MQLYSVDRKVSQPIEGHAASFAQFKMEGNAEESTLFCFAVRGQAGGKLHIIEVGTPPTGN
QPFPKKAVDVFFPPEAQNDFPVAMQISEKHDVVFLITKYGYIHLYDLETGTCIYMNRISG
ETIFVTAPHEATAGIIGVNRKGQVLSVCVEEENIIPYITNVLQNPDLALRMAVRNNLAGA
EELFARKFNALFAQGNYSEAAKVAANAPKGILRTPDTIRRFQSVPAQPGQTSPLLQYFGI
LLDQGQLNKYESLELCRPVLQQGRKQLLEKWLKEDKLECSEELGDLVKSVDPTLALSVYL
RANVPNKVIQCFAETGQVQKIVLYAKKVGYTPDWIFLLRNVMRISPDQGQQFAQMLVQDE
EPLADITQIVDVFMEYNLIQQCTAFLLDALKNNRPSEGPLQTRLLEMNLMHAPQVADAIL
GNQMFTHYDRAHIAQLCEKAGLLQRALEHFTDLYDIKRAVVHTHLLNPEWLVNYFGSLSV
EDSLECLRAMLSANIRQNLQICVQVASKYHEQLSTQSLIELFESFKSFEGLFYFLGSIVN
FSQDPDVHFKYIQAACKTGQIKEVERICRESNCYDPERVKNFLKEAKLTDQLPLIIVCDR
FDFVHDLVLYLYRNNLQKYIEIYVQKVNPSRLPVVIGGLLDVDCSEDVIKNLILVVRGQF
STDELVAEVEKRNRLKLLLPWLEARIHEGCEEPATHNALAKIYIDSNNNPERFLRENPYY
DSRVVGKYCEKRDPHLACVAYERGQCDLELINVCNENSLFKSLSRYLVRRKDPELWGSVL
LESNPYRRPLIDQVVQTALSETQDPEEVSVTVKAFMTADLPNELIELLEKIVLDNSVFSE
HRNLQNLLILTAIKADRTRVMEYINRLDNYDAPDIANIAISNELFEEAFAIFRKFDVNTS
AVQVLIEHIGNLDRAYEFAERCNEPAVWSQLAKAQLQKGMVKEAIDSYIKADDPSSYMEV
VQAANTSGNWEELVKYLQMARKKARESYVETELIFALAKTNRLAELEEFINGPNNAHIQQ
VGDRCYDEKMYDAAKLLYNNVSNFGRLASTLVHLGEYQAAVDGARKANSTRTWKEVCFAC
VDGKEFRLAQMCGLHIVVHADELEELINYYQDRGYFEELITMLEAALGLERAHMGMFTEL
AILYSKFKPQKMREHLELFWSRVNIPKVLRAAEQAHLWAELVFLYDKYEEYDNAIITMMN
HPTDAWKEGQFKDIITKVANVELYYRAIQFYLEFKPLLLNDLLMVLSPRLDHTRAVNYFS
KVKQLPLVKPYLRSVQNHNNKSVNESLNNLFITEEDYQALRTSIDAYDNFDNISLAQRLE
KHELIEFRRIAAYLFKGNNRWKQSVELCKKDSLYKDAMQYASESKDTELAEELLQWFLQE
EKRECFGACLFTCYDLLRPDVVLETAWRHNIMDFAMPYFIQVMKEYLTKVDKLDASESLR
KEEEQATETQPIVYGQPQLMLTAGPSVAVPPQAPFGYGYTAPPYGQPQPGFGYSM
>sp|P53675|CLH2_HUMAN Clathrin heavy chain 2 OS=Homo sapiens OX=9606 GN=CLTCL1 PE=1 SV=2
MAQILPVRFQEHFQLQNLGINPANIGFSTLTMESDKFICIREKVGEQAQVTIIDMSDPMA
PIRRPISAESAIMNPASKVIALKAGKTLQIFNIEMKSKMKAHTMAEEVIFWKWVSVNTVA
LVTETAVYHWSMEGDSQPMKMFDRHTSLVGCQVIHYRTDEYQKWLLLVGISAQQNRVVGA
MQLYSVDRKVSQPIEGHAAAFAEFKMEGNAKPATLFCFAVRNPTGGKLHIIEVGQPAAGN
QPFVKKAVDVFFPPEAQNDFPVAMQIGAKHGVIYLITKYGYLHLYDLESGVCICMNRISA
DTIFVTAPHKPTSGIIGVNKKGQVLSVCVEEDNIVNYATNVLQNPDLGLRLAVRSNLAGA
EKLFVRKFNTLFAQGSYAEAAKVAASAPKGILRTRETVQKFQSIPAQSGQASPLLQYFGI
LLDQGQLNKLESLELCHLVLQQGRKQLLEKWLKEDKLECSEELGDLVKTTDPMLALSVYL
RANVPSKVIQCFAETGQFQKIVLYAKKVGYTPDWIFLLRGVMKISPEQGLQFSRMLVQDE
EPLANISQIVDIFMENSLIQQCTSFLLDALKNNRPAEGLLQTWLLEMNLVHAPQVADAIL
GNKMFTHYDRAHIAQLCEKAGLLQQALEHYTDLYDIKRAVVHTHLLNPEWLVNFFGSLSV
EDSVECLHAMLSANIRQNLQLCVQVASKYHEQLGTQALVELFESFKSYKGLFYFLGSIVN
FSQDPDVHLKYIQAACKTGQIKEVERICRESSCYNPERVKNFLKEAKLTDQLPLIIVCDR
FGFVHDLVLYLYRNNLQRYIEIYVQKVNPSRTPAVIGGLLDVDCSEEVIKHLIMAVRGQF
STDELVAEVEKRNRLKLLLPWLESQIQEGCEEPATHNALAKIYIDSNNSPECFLRENAYY

DSSVVGRYCEKRDPHLACVAYERGQCDLELIKVCNENSLFKSEARYLVCRKDPELWAHVL
EETNPSRRQLIDQVVQTALSETRDPEEISVTVKAFMTADLPNELIELLEKIVLDNSVFSE
HRNLQNLLILTAIKADRTRVMEYISRLDNYDALDIASIAVSSALYEEAFTVFHKFDMNAS
AIQVLIEHIGNLDRAYEFAERCNEPAVWSQLAQAQLQKDLVKEAINSYIRGDDPSSYLEV
VQSASRSNNWEDLVKFLQMARKKGRESYIETELIFALAKTSRVSELEDFINGPNNAHIQQ
VGDRCYEEGMYEAAKLLYSNVSNFARLASTLVHLGEYQAAVDNSRKASSTRTWKEVCFAC
MDGQEFRFAQLCGLHIVIHADELEELMCYYQDRGYFEELILLLEAALGLERAHMGMFTEL
AILYSKFKPQKMLEHLELFWSRVNIPKVLRAAEQAHLWAELVFLYDKYEEYDNAVLTMMS
HPTEAWKEGQFKDIITKVANVELCYRALQFYLDYKPLLINDLLLVLSPRLDHTWTVSFFS
KAGQLPLVKPYLRSVQSHNNKSVNEALNHLLTEEEDYQGLRASIDAYDNFDNISLAQQLE
KHQLMEFRCIAAYLYKGNNWWAQSVELCKKDHLYKDAMQHAAESRDAELAQKLLQWFLEE
GKRECFAACLFTCYDLLRPDMVLELAWRHNLVDLAMPYFIQVMREYLSKVDKLDALESLR
KQEEHVTEPAPLVFDFDGHE
>sp|P0DPA3|SNH28_HUMAN Putative uncharacterized protein SNHG28 OS=Homo sapiens OX=9606 GN=SNHG28 PE=5 SV=1
MGMLAPGPLQGRRPRKGHKGQEDAVAPGCKASGRGSRVTHLLGYPTQNVSRSLRRKYAPP
PCGGPEDVALAPCTAAAACEAGPSPVYVKVKSAEPADCAEGPVQCKNGLLVSSPHCEEPC
AHSCAHPGLPPHLVHKLPLSYLQTQDTDAASRRINAPLAAGWSWLRLWLVTLASGVDFPQ
VSAWMRALPSPDCPGLRTTGEQMQKLLLKENKVKTRKSKRRSGEGSHLTTSILEQ
>sp|P0DPA2|VSIG8_HUMAN V-set and immunoglobulin domain-containing protein 8 OS=Homo sapiens OX=9606 GN=VSIG8 PE=2 SV=1
MRVGGAFHLLLVCLSPALLSAVRINGDGQEVLYLAEGDNVRLGCPYVLDPEDYGPNGLDI
EWMQVNSDPAHHRENVFLSYQDKRINHGSLPHLQQRVRFAASDPSQYDASINLMNLQVSD
TATYECRVKKTTMATRKVIVTVQARPAVPMCWTEGHMTYGNDVVLKCYASGGSQPLSYKW
AKISGHHYPYRAGSYTSQHSYHSELSYQESFHSSINQGLNNGDLVLKDISRADDGLYQCT
VANNVGYSVCVVEVKVSDSRRIGVIIGIVLGSLLALGCLAVGIWGLVCCCCGGSGAGGAR
GAFGYGNGGGVGGGACGDLASEIREDAVAPGCKASGRGSRVTHLLGYPTQNVSRSLRRKY
APPPCGGPEDVALAPCTAAAACEAGPSPVYVKVKSAEPADCAEGPVQCKNGLLV
>sp|P02511|CRYAB_HUMAN Alpha-crystallin B chain OS=Homo sapiens OX=9606 GN=CRYAB PE=1 SV=2
MDIAIHHPWIRRPFFPFHSPSRLFDQFFGEHLLESDLFPTSTSLSPFYLRPPSFLRAPSW
FDTGLSEMRLEKDRFSVNLDVKHFSPEELKVKVLGDVIEVHGKHEERQDEHGFISREFHR
KYRIPADVDPLTITSSLSSDGVLTVNGPRKQVSGPERTIPITREEKPAVTAAPKK
>sp|P00338|LDHA_HUMAN L-lactate dehydrogenase A chain OS=Homo sapiens OX=9606 GN=LDHA PE=1 SV=2
MATLKDQLIYNLLKEEQTPQNKITVVGVGAVGMACAISILMKDLADELALVDVIEDKLKG
EMMDLQHGSLFLRTPKIVSGKDYNVTANSKLVIITAGARQQEGESRLNLVQRNVNIFKFI
IPNVVKYSPNCKLLIVSNPVDILTYVAWKISGFPKNRVIGSGCNLDSARFRYLMGERLGV
HPLSCHGWVLGEHGDSSVPVWSGMNVAGVSLKTLHPDLGTDKDKEQWKEVHKQVVESAYE
VIKLKGYTSWAIGLSVADLAESIMKNLRRVHPVSTMIKGLYGIKDDVFLSVPCILGQNGI
SDLVKVTLTSEEEARLKKSADTLWGIQKELQF
>sp|Q9BYZ2|LDH6B_HUMAN L-lactate dehydrogenase A-like 6B OS=Homo sapiens OX=9606 GN=LDHAL6B PE=1 SV=3
MSWTVPVVRASQRVSSVGANFLCLGMALCPRQATRIPLNGTWLFTPVSKMATVKSELIER
FTSEKPVHHSKVSIIGTGSVGMACAISILLKGLSDELALVDLDEDKLKGETMDLQHGSPF
TKMPNIVCSKDYFVTANSNLVIITAGARQEKGETRLNLVQRNVAIFKLMISSIVQYSPHC
KLIIVSNPVDILTYVAWKLSAFPKNRIIGSGCNLDTARFRFLIGQKLGIHSESCHGWILG

EHGDSSVPVWSGVNIAGVPLKDLNSDIGTDKDPEQWKNVHKEVTATAYEIIKMKGYTSWA
IGLSVADLTESILKNLRRIHPVSTIIKGLYGIDEEVFLSIPCILGENGITNLIKIKLTPE
EEAHLKKSAKTLWEIQNKLKL
>sp|P42357|HUTH_HUMAN Histidine ammonia-lyase OS=Homo sapiens OX=9606 GN=HAL PE=1 SV=1
MPRYTVHVRGEWLAVPCQDAQLTVGWLGREAVRRYIKNKPDNGGFTSVDDAHFLVRRCKG
LGLLDNEDRLEVALENNEFVEVVIEGDAMSPDFIPSQPEGVYLYSKYREPEKYIELDGDR
LTTEDLVNLGKGRYKIKLTPTAEKRVQKSREVIDSIIKEKTVVYGITTGFGKFARTVIPI
NKLQELQVNLVRSHSSGVGKPLSPERCRMLLALRINVLAKGYSGISLETLKQVIEMFNAS
CLPYVPEKGTVGASGDLAPLSHLALGLVGEGKMWSPKSGWADAKYVLEAHGLKPVILKPK
EGLALINGTQMITSLGCEAVERASAIARQADIVAALTLEVLKGTTKAFDTDIHALRPHRG
QIEVAFRFRSLLDSDHHPSEIAESHRFCDRVQDAYTLRCCPQVHGVVNDTIAFVKNIITT
ELNSATDNPMVFANRGETVSGGNFHGEYPAKALDYLAIGIHELAAISERRIERLCNPSLS
ELPAFLVAEGGLNSGFMIAHCTAAALVSENKALCHPSSVDSLSTSAATEDHVSMGGWAAR
KALRVIEHVEQVLAIELLAACQGIEFLRPLKTTTPLEKVYDLVRSVVRPWIKDRFMAPDI
EAAHRLLLEQKVWEVAAPYIEKYRMEHIPESRPLSPTAFSLQFLHKKSTKIPESEDL
>sp|Q7Z794|K2C1B_HUMAN Keratin, type II cytoskeletal 1b OS=Homo sapiens OX=9606 GN=KRT77 PE=1 SV=3
MSHQFSSQSAFSSMSRRVYSTSSSAGSGGGSPAVGSVCYARGRCGGGGYGIHGRGFGSRS
LYNLGGSRSISINLMGRSTSGFCQGGGVGGFGGGRGFGVGSTGAGGFGGGGFGGAGFGTS
NFGLGGFGPYCPPGGIQEVTINQSLLEPLHLEVDPEIQRIKTQEREQIMVLNNKFASFID
KVRFLEQQNQVLQTKWELLQQVNTSTGTNNLEPLLENYIGDLRRQVDLLSAEQMRQNAEV
RSMQDVVEDYKSKYEDEINKRTGSENDFVVLKKDVDAAYVSKVDLESRVDTLTGEVNFLK
YLFLTELSQVQTHISDTNVILSMDNNRSLDLDSIIDAVRTQYELIAQRSKDEAEALYQTK
YQELQITAGRHGDDLKNSKMEIAELNRTVQRLQAEISNVKKQIEQMQSLISDAEERGEQA
LQDAWQKLQDLEEALQQSKEELARLLRDYQAMLGVKLSLDVEIATYRQLLEGEESRMSGE
LQSHVSISVQNSQVSVNGGAGGGGSYGSGGYGGGSGGGYGGGRSYRGGGARGRSGGGYGS
GCGGGGGSYGGSGRSGRGSSRVQIIQTSTNTSHRRILE
>sp|Q7Z3Y8|K1C27_HUMAN Keratin, type I cytoskeletal 27 OS=Homo sapiens OX=9606 GN=KRT27 PE=1 SV=2
MSVRFSSTSRRLGSCGGTGSVRLSSGGAGFGAGNTCGVPGIGSGFSCAFGGSSSAGGYGG
GLGGGSASCAAFTGNEHGLLSGNEKVTMQNLNDRLASYLENVRALEEANADLEQKIKGWY
EKFGPGSCRGLDHDYSRYFPIIDELKNQIISATTSNAHVVLQNDNARLTADDFRLKFENE
LALHQSVEADINGLRRVLDELTLCRTDLEIQLETLSEELAYLKKNHEEEMKALQCAAGGN
VNVEMNAAPGVDLTVLLNNMRAEYEALAEQNRRDAEAWFNEKSASLQQQISDDAGATTSA
RNELIEMKRTLQTLEIELQSLLATKHSLECSLTETESNYCAQLAQIQAQIGALEEQLHQV
RTETEGQKLEYEQLLDIKVHLEKEIETYCLLIDGEDGSCSKSKGYGGPGNQTKDSSKTTI
VKTVVEEIDPRGKVLSSRVHTVEEKSTKVNNKNEQRVSS
>sp|Q13867|BLMH_HUMAN Bleomycin hydrolase OS=Homo sapiens OX=9606 GN=BLMH PE=1 SV=1
MSSSGLNSEKVAALIQKLNSDPQFVLAQNVGTTHDLLDICLKRATVQRAQHVFQHAVPQE
GKPITNQKSSGRCWIFSCLNVMRLPFMKKLNIEEFEFSQSYLFFWDKVERCYFFLSAFVD
TAQRKEPEDGRLVQFLLMNPANDGGQWDMLVNIVEKYGVIPKKCFPESYTTEATRRMNDI
LNHKMREFCIRLRNLVHSGATKGEISATQDVMMEEIFRVVCICLGNPPETFTWEYRDKDK
NYQKIGPITPLEFYREHVKPLFNMEDKICLVNDPRPQHKYNKLYTVEYLSNMVGGRKTLY
NNQPIDFLKKMVAASIKDGEAVWFGCDVGKHFNSKLGLSDMNLYDHELVFGVSLKNMNKA
ERLTFGESLMTHAMTFTAVSEKDDQDGAFTKWRVENSWGEDHGHKGYLCMTDEWFSEYVY

EVVVDRKHVPEEVLAVLEQEPIILPAWDPMGALAE
>sp|P68133|ACTS_HUMAN Actin, alpha skeletal muscle OS=Homo sapiens OX=9606 GN=ACTA1 PE=1 SV=1
MCDEDETTALVCDNGSGLVKAGFAGDDAPRAVFPSIVGRPRHQGVMVGMGQKDSYVGDEA
QSKRGILTLKYPIEHGIITNWDDMEKIWHHTFYNELRVAPEEHPTLLTEAPLNPKANREK
MTQIMFETFNVPAMYVAIQAVLSLYASGRTTGIVLDSGDGVTHNVPIYEGYALPHAIMRL
DLAGRDLTDYLMKILTERGYSFVTTAEREIVRDIKEKLCYVALDFENEMATAASSSSLEK
SYELPDGQVITIGNERFRCPETLFQPSFIGMESAGIHETTYNSIMKCDIDIRKDLYANNV
MSGGTTMYPGIADRMQKEITALAPSTMKIKIIAPPERKYSVWIGGSILASLSTFQQMWIT
KQEYDEAGPSIVHRKCF
>sp|P68032|ACTC_HUMAN Actin, alpha cardiac muscle 1 OS=Homo sapiens OX=9606 GN=ACTC1 PE=1 SV=1
MCDDEETTALVCDNGSGLVKAGFAGDDAPRAVFPSIVGRPRHQGVMVGMGQKDSYVGDEA
QSKRGILTLKYPIEHGIITNWDDMEKIWHHTFYNELRVAPEEHPTLLTEAPLNPKANREK
MTQIMFETFNVPAMYVAIQAVLSLYASGRTTGIVLDSGDGVTHNVPIYEGYALPHAIMRL
DLAGRDLTDYLMKILTERGYSFVTTAEREIVRDIKEKLCYVALDFENEMATAASSSSLEK
SYELPDGQVITIGNERFRCPETLFQPSFIGMESAGIHETTYNSIMKCDIDIRKDLYANNV
LSGGTTMYPGIADRMQKEITALAPSTMKIKIIAPPERKYSVWIGGSILASLSTFQQMWIS
KQEYDEAGPSIVHRKCF
>sp|P62736|ACTA_HUMAN Actin, aortic smooth muscle OS=Homo sapiens OX=9606 GN=ACTA2 PE=1 SV=1
MCEEEDSTALVCDNGSGLCKAGFAGDDAPRAVFPSIVGRPRHQGVMVGMGQKDSYVGDEA
QSKRGILTLKYPIEHGIITNWDDMEKIWHHSFYNELRVAPEEHPTLLTEAPLNPKANREK
MTQIMFETFNVPAMYVAIQAVLSLYASGRTTGIVLDSGDGVTHNVPIYEGYALPHAIMRL
DLAGRDLTDYLMKILTERGYSFVTTAEREIVRDIKEKLCYVALDFENEMATAASSSSLEK
SYELPDGQVITIGNERFRCPETLFQPSFIGMESAGIHETTYNSIMKCDIDIRKDLYANNV
LSGGTTMYPGIADRMQKEITALAPSTMKIKIIAPPERKYSVWIGGSILASLSTFQQMWIS
KQEYDEAGPSIVHRKCF
>sp|P63267|ACTH_HUMAN Actin, gamma-enteric smooth muscle OS=Homo sapiens OX=9606 GN=ACTG2 PE=1 SV=1
MCEEETTALVCDNGSGLCKAGFAGDDAPRAVFPSIVGRPRHQGVMVGMGQKDSYVGDEAQ
SKRGILTLKYPIEHGIITNWDDMEKIWHHSFYNELRVAPEEHPTLLTEAPLNPKANREKM
TQIMFETFNVPAMYVAIQAVLSLYASGRTTGIVLDSGDGVTHNVPIYEGYALPHAIMRLD
LAGRDLTDYLMKILTERGYSFVTTAEREIVRDIKEKLCYVALDFENEMATAASSSSLEKS
YELPDGQVITIGNERFRCPETLFQPSFIGMESAGIHETTYNSIMKCDIDIRKDLYANNVL
SGGTTMYPGIADRMQKEITALAPSTMKIKIIAPPERKYSVWIGGSILASLSTFQQMWISK
PEYDEAGPSIVHRKCF
>sp|Q6E0U4|DMKN_HUMAN Dermokine OS=Homo sapiens OX=9606 GN=DMKN PE=1 SV=3
MKFQGPLACLLLALCLGSGEAGPLQSGEESTGTNIGEALGHGLGDALSEGVGKAIGKEAG
GAAGSKVSEALGQGTREAVGTGVRQVPGFGVADALGNRVGEAAHALGNTGHEIGRQAEDV
IRHGADAVRGSWQGVPGHNGAWETSGGHGIFGSQGGLGGQGQGNPGGLGTPWVHGYPGNS
AGSFGMNPQGAPWGQGGNGGPPNFGTNTQGAVAQPGYGSVRASNQNEGCTNPPPSGSGGG
SSNSGGGSGSQSGSSGSGSNGDNNNGSSSGGSSSGSSSGGSSGGSSGGSSGNSGGSRGDS
GSESSWGSSTGSSSGNHGGSGGGNGHKPGCEKPGNEARGSGESGIQNSETSPGMFNFDTF
WKNFKSKLGFINWDAINKNQVPPPSTRALLYFSRLWEDFKQNTPFLNWKAIIEGADASSL
QKRAGRDDQNYNYNQHAYPTAYGGKYSVKTPAKGGVSPSSSASRVQPGLLQWVKFW

>sp|Q3SY84|K2C71_HUMAN Keratin, type II cytoskeletal 71 OS=Homo sapiens OX=9606 GN=KRT71 PE=1 SV=3
MSRQFTCKSGAAAKGGFSGCSAVLSGGSSSSFRAGSKGLSGGFGSRSLYSLGGVRSLNVA
SGSGKSGGYGFGRGRASGFAGSMFGSVALGPVCPTVCPPGGIHQVTVNESLLAPLNVELD
PEIQKVRAQEREQIKALNNKFASFIDKVRFLEQQNQVLETKWELLQQLDLNNCKNNLEPI
LEGYISNLRKQLETLSGDRVRLDSELRNVRDVVEDYKKRYEEEINKRTAAENEFVLLKKD
VDAAYANKVELQAKVESMDQEIKFFRCLFEAEITQIQSHISDMSVILSMDNNRNLDLDSI
IDEVRTQYEEIALKSKAEAEALYQTKFQELQLAAGRHGDDLKNTKNEISELTRLIQRIRS
EIENVKKQASNLETAIADAEQRGDNALKDARAKLDELEGALHQAKEELARMLREYQELMS
LKLALDMEIATYRKLLESEECRMSGEFPSPVSISIISSTSGGSVYGFRPSMVSGGYVANS
SNCISGVCSVRGGEGRSRGSANDYKDTLGKGSSLSAPSKKTSR
>sp|Q96QA5|GSDMA_HUMAN Gasdermin-A OS=Homo sapiens OX=9606 GN=GSDMA PE=1 SV=4
MTMFENVTRALARQLNPRGDLTPLDSLIDFKRFHPFCLVLRKRKSTLFWGARYVRTDYTL
LDVLEPGSSPSDPTDTGNFGFKNMLDTRVEGDVDVPKTVKVKGTAGLSQNSTLEVQTLSV
APKALETVQERKLAADHPFLKEMQDQGENLYVVMEVVETVQEVTLERAGKAEACFSLPFF
APLGLQGSINHKEAVTIPKGCVLAFRVRQLMVKGKDEWDIPHICNDNMQTFPPGEKSGEE
KVILIQASDVGDVHEGFRTLKEEVQRETQQVEKLSRVGQSSLLSSLSKLLGKKKELQDLE
LALEGALDKGHEVTLEALPKDVLLSKEAVGAILYFVGALTELSEAQQKLLVKSMEKKILP
VQLKLVESTMEQNFLLDKEGVFPLQPELLSSLGDEELTLTEALVGLSGLEVQRSGPQYMW
DPDTLPRLCALYAGLSLLQQLTKAS
>sp|P55072|TERA_HUMAN Transitional endoplasmic reticulum ATPase OS=Homo sapiens OX=9606 GN=VCP PE=1 SV=4
MASGADSKGDDLSTAILKQKNRPNRLIVDEAINEDNSVVSLSQPKMDELQLFRGDTVLLK
GKKRREAVCIVLSDDTCSDEKIRMNRVVRNNLRVRLGDVISIQPCPDVKYGKRIHVLPID
DTVEGITGNLFEVYLKPYFLEAYRPIRKGDIFLVRGGMRAVEFKVVETDPSPYCIVAPDT
VIHCEGEPIKREDEEESLNEVGYDDIGGCRKQLAQIKEMVELPLRHPALFKAIGVKPPRG
ILLYGPPGTGKTLIARAVANETGAFFFLINGPEIMSKLAGESESNLRKAFEEAEKNAPAI
IFIDELDAIAPKREKTHGEVERRIVSQLLTLMDGLKQRAHVIVMAATNRPNSIDPALRRF
GRFDREVDIGIPDATGRLEILQIHTKNMKLADDVDLEQVANETHGHVGADLAALCSEAAL
QAIRKKMDLIDLEDETIDAEVMNSLAVTMDDFRWALSQSNPSALRETVVEVPQVTWEDIG
GLEDVKRELQELVQYPVEHPDKFLKFGMTPSKGVLFYGPPGCGKTLLAKAIANECQANFI
SIKGPELLTMWFGESEANVREIFDKARQAAPCVLFFDELDSIAKARGGNIGDGGGAADRV
INQILTEMDGMSTKKNVFIIGATNRPDIIDPAILRPGRLDQLIYIPLPDEKSRVAILKAN
LRKSPVAKDVDLEFLAKMTNGFSGADLTEICQRACKLAIRESIESEIRRERERQTNPSAM
EVEEDDPVPEIRRDHFEEAMRFARRSVSDNDIRKYEMFAQTLQQSRGFGSFRFPSGNQGG
AGPSQGSGGGTGGSVYTEDNDDDLYG
>sp|P00491|PNPH_HUMAN Purine nucleoside phosphorylase OS=Homo sapiens OX=9606 GN=PNP PE=1 SV=2
MENGYTYEDYKNTAEWLLSHTKHRPQVAIICGSGLGGLTDKLTQAQIFDYGEIPNFPRST
VPGHAGRLVFGFLNGRACVMMQGRFHMYEGYPLWKVTFPVRVFHLLGVDTLVVTNAAGGL
NPKFEVGDIMLIRDHINLPGFSGQNPLRGPNDERFGDRFPAMSDAYDRTMRQRALSTWKQ
MGEQRELQEGTYVMVAGPSFETVAECRVLQKLGADAVGMSTVPEVIVARHCGLRVFGFSL
ITNKVIMDYESLEKANHEEVLAAGKQAAQKLEQFVSILMASIPLPDKAS
>sp|Q96P63|SPB12_HUMAN Serpin B12 OS=Homo sapiens OX=9606 GN=SERPINB12 PE=1 SV=1
MDSLVTANTKFCFDLFQEIGKDDRHKNIFFSPLSLSAALGMVRLGARSDSAHQIDEVLHF

NEFSQNESKEPDPCLKSNKQKAGSLNNESGLVSCYFGQLLSKLDRIKTDYTLSIANRLYG
EQEFPICQEYLDGVIQFYHTTIESVDFQKNPEKSRQEINFWVECQSQGKIKELFSKDAIN
AETVLVLVNAVYFKAKWETYFDHENTVDAPFCLNANENKSVKMMTQKGLYRIGFIEEVKA
QILEMRYTKGKLSMFVLLPSHSKDNLKGLEELERKITYEKMVAWSSSENMSEESVVLSFP
RFTLEDSYDLNSILQDMGITDIFDETRADLTGISPSPNLYLSKIIHKTFVEVDENGTQAA
AATGAVVSERSLRSWVEFNANHPFLFFIRHNKTQTILFYGRVCSP
>sp|P06396|GELS_HUMAN Gelsolin OS=Homo sapiens OX=9606 GN=GSN PE=1 SV=1
MAPHRPAPALLCALSLALCALSLPVRAATASRGASQAGAPQGRVPEARPNSMVVEHPEFL
KAGKEPGLQIWRVEKFDLVPVPTNLYGDFFTGDAYVILKTVQLRNGNLQYDLHYWLGNEC
SQDESGAAAIFTVQLDDYLNGRAVQHREVQGFESATFLGYFKSGLKYKKGGVASGFKHVV
PNEVVVQRLFQVKGRRVVRATEVPVSWESFNNGDCFILDLGNNIHQWCGSNSNRYERLKA
TQVSKGIRDNERSGRARVHVSEEGTEPEAMLQVLGPKPALPAGTEDTAKEDAANRKLAKL
YKVSNGAGTMSVSLVADENPFAQGALKSEDCFILDHGKDGKIFVWKGKQANTEERKAALK
TASDFITKMDYPKQTQVSVLPEGGETPLFKQFFKNWRDPDQTDGLGLSYLSSHIANVERV
PFDAATLHTSTAMAAQHGMDDDGTGQKQIWRIEGSNKVPVDPATYGQFYGGDSYIILYNY
RHGGRQGQIIYNWQGAQSTQDEVAASAILTAQLDEELGGTPVQSRVVQGKEPAHLMSLFG
GKPMIIYKGGTSREGGQTAPASTRLFQVRANSAGATRAVEVLPKAGALNSNDAFVLKTPS
AAYLWVGTGASEAEKTGAQELLRVLRAQPVQVAEGSEPDGFWEALGGKAAYRTSPRLKDK
KMDAHPPRLFACSNKIGRFVIEEVPGELMQEDLATDDVMLLDTWDQVFVWVGKDSQEEEK
TEALTSAKRYIETDPANRDRRTPITVVKQGFEPPSFVGWFLGWDDDYWSVDPLDRAMAEL
AA
>sp|Q09666|AHNK_HUMAN Neuroblast differentiation-associated protein AHNAK OS=Homo sapiens OX=9606 GN=AHNAK PE=1 SV=2
MEKEETTRELLLPNWQGSGSHGLTIAQRDDGVFVQEVTQNSPAARTGVVKEGDQIVGATI
YFDNLQSGEVTQLLNTMGHHTVGLKLHRKGDRSPEPGQTWTREVFSSCSSEVVLSGDDEE
YQRIYTTKIKPRLKSEDGVEGDLGETQSRTITVTRRVTAYTVDVTGREGAKDIDISSPEF
KIKIPRHELTEISNVDVETQSGKTVIRLPSGSGAASPTGSAVDIRAGAISASGPELQGAG
HSKLQVTMPGIKVGGSGVNVNAKGLDLGGRGGVQVPAVDISSSLGGRAVEVQGPSLESGD
HGKIKFPTMKVPKFGVSTGREGQTPKAGLRVSAPEVSVGHKGGKPGLTIQAPQLEVSVPS
ANIEGLEGKLKGPQITGPSLEGDLGLKGAKPQGHIGVDASAPQIGGSITGPSVEVQAPDI
DVQGPGSKLNVPKMKVPKFSVSGAKGEETGIDVTLPTGEVTVPGVSGDVSLPEIATGGLE
GKMKGTKVKTPEMIIQKPKISMQDVDLSLGSPKLKGDIKVSAPGVQGDVKGPQVALKGSR
VDIETPNLEGTLTGPRLGSPSGKTGTCRISMSEVDLNVAAPKVKGGVDVTLPRVEGKVKV
PEVDVRGPKVDVSAPDVEAHGPEWNLKMPKMKMPTFSTPGAKGEGPDVHMTLPKGDISIS
GPKVNVEAPDVNLEGLGGKLKGPDVKLPDMSVKTPKISMPDVDLHVKGTKVKGEYDVTVP
KLEGELKGPKVDIDAPDVDVHGPDWHLKMPKMKMPKFSVPGFKAEGPEVDVNLPKADVDI
SGPKIDVTAPDVSIEEPEGKLKGPKFKMPEMNIKVPKISMPDVDLHLKGPNVKGEYDVTM
PKVESEIKVPDVELKSAKMDIDVPDVEVQGPDWHLKMPKMKMPKFSMPGFKAEGPEVDVN
LPKADVDISGPKVGVEVPDVNIEGPEGKLKGPKFKMPEMNIKAPKISMPDVDLHMKGPKV
KGEYDMTVPKLEGDLKGPKVDVSAPDVEMQGPDWNLKMPKIKMPKFSMPSLKGEGPEFDV
NLSKANVDISAPKVDTNAPDLSLEGPEGKLKGPKFKMPEMHFRAPKMSLPDVDLDLKGPK
MKGNVDISAPKIEGEMQVPDVDIRGPKVDIKAPDVEGQGLDWSLKIPKMKMPKFSMPSLK
GEGPEVDVNLPKADVVVSGPKVDIEAPDVSLEGPEGKLKGPKFKMPEMHFKTPKISMPDV
DLHLKGPKVKGDVDVSVPKVEGEMKVPDVEIKGPKMDIDAPDVEVQGPDWHLKMPKMKMP
KFSMPGFKGEGREVDVNLPKADIDVSGPKVDVEVPDVSLEGPEGKLKGPKFKMPEMHFKA
PKISMPDVDLNLKGPKLKGDVDVSLPEVEGEMKVPDVDIKGPKVDISAPDVDVHGPDWHL

KMPKVKMPKFSMPGFKGEGPEVDVKLPKADVDVSGPKMDAEVPDVNIEGPDAKLKGPKFK
MPEMSIKPQKISIPDVGLHLKGPKMKGDYDVTVPKVEGEIKAPDVDIKGPKVDINAPDVE
VHGPDWHLKMPKVKMPKFSMPGFKGEGPEVDMNLPKADLGVSGPKVDIDVPDVNLEAPEG
KLKGPKFKMPSMNIQTHKISMPDVGLNLKAPKLKTDVDVSLPKVEGDLKGPEIDVKAPKM
DVNVGDIDIEGPEGKLKGPKFKMPEMHFKAPKISMPDVDLHLKGPKVKGDMDVSVPKVEG
EMKVPDVDIKGPKVDIDAPDVEVHDPDWHLKMPKMKMPKFSMPGFKAEGPEVDVNLPKAD
IDVSGPSVDTDAPDLDIEGPEGKLKGSKFKMPKLNIKAPKVSMPDVDLNLKGPKLKGEID
ASVPELEGDLRGPQVDVKGPFVEAEVPDVDLECPDAKLKGPKFKMPEMHFKAPKISMPDV
DLHLKGPKVKGDADVSVPKLEGDLTGPSVGVEVPDVELECPDAKLKGPKFKMPDMHFKAP
KISMPDVDLHLKGPKVKGDVDVSVPKLEGDLTGPSVGVEVPDVELECPDAKLKGPKFKMP
EMHFKTPKISMPDVDLHLKGPKVKGDMDVSVPKVEGEMKVPDVDIKGPKMDIDAPDVDVH
GPDWHLKMPKMKMPKFSMPGFKAEGPEVDVNLPKADVVVSGPKVDVEVPDVSLEGPEGKL
KGPKLKMPEMHFKAPKISMPDVDLHLKGPKVKGDVDVSLPKLEGDLTGPSVDVEVPDVEL
ECPDAKLKGPKFKMPEMHFKTPKISMPDVNLNLKGPKVKGDMDVSVPKVEGEMKVPDVDI
RGPKVDIDAPDVDVHGPDWHLKMPKMKMPKFSMPGFKGEGPEVDVNLPKADVDVSGPKVD
VEVPDVSLEGPEGKLKGPKFKMPEMHFKTPKISMPDVDFNLKGPKIKGDVDVSAPKLEGE
LKGPELDVKGPKLDADMPEVAVEGPNGKWKTPKFKMPDMHFKAPKISMPDLDLHLKSPKA
KGEVDVDVPKLEGDLKGPHVDVSGPDIDIEGPEGKLKGPKFKMPDMHFKAPNISMPDVDL
NLKGPKIKGDVDVSVPEVEGKLEVPDMNIRGPKVDVNAPDVQAPDWHLKMPKMKMPKFSM
PGFKAEGPEVDVNLPKADVDISGPKVDIEGPDVNIEGPEGKLKGPKLKMPEMNIKAPKIS
MPDFDLHLKGPKVKGDVDVSLPKVEGDLKGPEVDIKGPKVDINAPDVGVQGPDWHLKMPK
VKMPKFSMPGFKGEGPDGDVKLPKADIDVSGPKVDIEGPDVNIEGPEGKLKGPKFKMPEM
NIKAPKISMPDIDLNLKGPKVKGDVDVSLPKVEGDLKGPEVDIKGPKVDIDAPDVDVHGP
DWHLKMPKIKMPKISMPGFKGEGPDVDVNLPKADIDVSGPKVDVECPDVNIEGPEGKWKS
PKFKMPEMHFKTPKISMPDIDLNLTGPKIKGDVDVTGPKVEGDLKGPEVDLKGPKVDIDV
PDVNVQGPDWHLKMPKMKMPKFSMPGFKAEGPEVDVNLPKADVDVSGPKVDVEGPDVNIE
GPEGKLKGPKFKMPEMNIKAPKIPMPDFDLHLKGPKVKGDVDISLPKVEGDLKGPEVDIR
GPQVDIDVPDVGVQGPDWHLKMPKVKMPKFSMPGFKGEGPDVDVNLPKADLDVSGPKVDI
DVPDVNIEGPEGKLKGPKFKMPEMNIKAPKISMPDIDLNLKGPKVKGDMDVSLPKVEGDM
KVPDVDIKGPKVDINAPDVDVQGPDWHLKMPKIKMPKISMPGFKGEGPEVDVNLPKADLD
VSGPKVDVDVPDVNIEGPDAKLKGPKFKMPEMNIKAPKISMPDLDLNLKGPKMKGEVDVS
LANVEGDLKGPALDIKGPKIDVDAPDIDIHGPDAKLKGPKLKMPDMHVNMPKISMPEIDL
NLKGSKLKGDVDVSGPKLEGDIKAPSLDIKGPEVDVSGPKLNIEGKSKKSRFKLPKFNFS
GSKVQTPEVDVKGKKPDIDITGPKVDINAPDVEVQGKVKGSKFKMPFLSISSPKVSMPDV
ELNLKSPKVKGDLDIAGPNLEGDFKGPKVDIKAPEVNLNAPDVDVHGPDWNLKMPKMKMP
KFSVSGLKAEGPDVAVDLPKGDINIEGPSMNIEGPDLNVEGPEGGLKGPKFKMPDMNIKA
PKISMPDIDLNLKGPKVKGDVDISLPKLEGDLKGPEVDIKGPKVDINAPDVDVHGPDWHL
KMPKVKMPKFSMPGFKGEGPEVDVTLPKADIDISGPNVDVDVPDVNIEGPDAKLKGPKFK
MPEMNIKAPKISMPDFDLNLKGPKMKGDVVVSLPKVEGDLKGPEVDIKGPKVDIDTPDIN
IEGSEGKFKGPKFKIPEMHLKAPKISMPDIDLNLKGPKVKGDVDVSLPKMEGDLKGPEVD
IKGPKVDINAPDVDVQGPDWHLKMPKVKMPKFSMPGFKGEGPDVDVNLPKADLDVSGPKV
DIDVPDVNIEGPEGKLKGPKFKMPEMNIKAPKISMPDIDLNLKGPKVKGDMDVSLPKVEG
DMQVPDLDIKGPKVDINAPDVDVRGPDWHLKMPKIKMPKISMPGFKGEGPEVDVNLPKAD
LDVSGPKVDVDVPDVNIEGPDAKLKGPKFKMPEMNIKAPKISMPDFDLHLKGPKVKGDVD
VSLPKMEGDLKAPEVDIKGPKVDIDAPDVDVHGPDWHLKMPKVKMPKFSMPGFKGEGPEV
DVNLPKADIDVSGPKVDIDTPDIDIHGPEGKLKGPKFKMPDLHLKAPKISMPEVDLNLKG
PKMKGDVDVSLPKVEGDLKGPEVDIKGPKVDIDVPDVDVQGPDWHLKMPKVKMPKFSMPG

FKGEGPDVDVNLPKADLDVSGPKVDIDVPDVNIEGPDAKLKGPKFKMPEMNIKAPKISMP
DFDLHLKGPKVKGDVDVSLPKVEGDLKGPEVDIKGPKVDIDAPDVDVHGPDWHLKMPKVK
MPKFSMPGFKGEGPDVDVTLPKADIEISGPKVDIDAPDVSIEGPDAKLKGPKFKMPEMNI
KAPKISMPDIDFNLKGPKVKGDVDVSLPKVEGDLKGPEIDIKGPSLDIDTPDVNIEGPEG
KLKGPKFKMPEMNIKAPKISMPDFDLHLKGPKVKGDVDVSLPKVESDLKGPEVDIEGPEG
KLKGPKFKMPDVHFKSPQISMSDIDLNLKGPKIKGDMDISVPKLEGDLKGPKVDVKGPKV
GIDTPDIDIHGPEGKLKGPKFKMPDLHLKAPKISMPEVDLNLKGPKVKGDMDISLPKVEG
DLKGPEVDIRDPKVDIDVPDVDVQGPDWHLKMPKVKMPKFSMPGFKGEGPDVDVNLPKAD
IDVSGPKVDVDVPDVNIEGPDAKLKGPKFKMPEMSIKAPKISMPDIDLNLKGPKVKGDVD
VTLPKVEGDLKGPEADIKGPKVDINTPDVDVHGPDWHLKMPKVKMPKFSMPGFKGEGPDV
DVSLPKADIDVSGPKVDVDIPDVNIEGPDAKLKGPKFKMPEINIKAPKISIPDVDLDLKG
PKVKGDFDVSVPKVEGTLKGPEVDLKGPRLDFEGPDAKLSGPSLKMPSLEISAPKVTAPD
VDLHLKAPKIGFSGPKLEGGEVDLKGPKVEAPSLDVHMDSPDINIEGPDVKIPKFKKPKF
GFGAKSPKADIKSPSLDVTVPEAELNLETPEISVGGKGKKSKFKMPKIHMSGPKIKAKKQ
GFDLNVPGGEIDASLKAPDVDVNIAGPDAALKVDVKSPKTKKTMFGKMYFPDVEFDIKSP
KFKAEAPLPSPKLEGELQAPDLELSLPAIHVEGLDIKAKAPKVKMPDVDISVPKIEGDLK
GPKVQANLGAPDINIEGLDAKVKTPSFGISAPQVSIPDVNVNLKGPKIKGDVPSVGLEGP
DVDLQGPEAKIKFPKFSMPKIGIPGVKMEGGGAEVHAQLPSLEGDLRGPDVKLEGPDVSL
KGPGVDLPSVNLSMPKVSGPDLDLNLKGPSLKGDLDASVPSMKVHAPGLNLSGVGGKMQV
GGDGVKVPGIDATTKLNVGAPDVTLRGPSLQGDLAVSGDIKCPKVSVGAPDLSLEASEGS
IKLPKMKLPQFGISTPGSDLHVNAKGPQVSGELKGPGVDVNLKGPRISAPNVDFNLEGPK
VKGSLGATGEIKGPTVGGGLPGIGVQGLEGNLQMPGIKSSGCDVNLPGVNVKLPTGQISG
PEIKGGLKGSEVGFHGAAPDISVKGPAFNMASPESDFGINLKGPKIKGGADVSGGVSAPD
ISLGEGHLSVKGSGGEWKGPQVSSALNLDTSKFAGGLHFSGPKVEGGVKGGQIGLQAPGL
SVSGPQGHLESGSGKVTFPKMKIPKFTFSGRELVGREMGVDVHFPKAEASIQAGAGDGEW
EESEVKLKKSKIKMPKFNFSKPKGKGGVTGSPEASISGSKGDLKSSKASLGSLEGEAEAE
ASSPKGKFSLFKSKKPRHRSNSFSDEREFSGPSTPTGTLEFEGGEVSLEGGKVKGKHGKL
KFGTFGGLGSKSKGHYEVTGSDDETGKLQGSGVSLASKKSRLSSSSSNDSGNKVGIQLPE
VELSVSTKKE
>sp|Q86Y46|K2C73_HUMAN Keratin, type II cytoskeletal 73 OS=Homo sapiens OX=9606 GN=KRT73 PE=1 SV=1
MSRQFTYKSGAAAKGGFSGCSAVLSGGSSSSYRAGGKGLSGGFSSRSLYSLGGARSISFN
VASGSGWAGGYGFGRGRASGFAGSMFGSVALGSVCPSLCPPGGIHQVTINKSLLAPLNVE
LDPEIQKVRAQEREQIKVLNNKFASFIDKVRFLEQQNQVLETKWELLQQLDLNNCKNNLE
PILEGYISNLRKQLETLSGDRVRLDSELRSVREVVEDYKKRYEEEINKRTTAENEFVVLK
KDVDAAYTSKVELQAKVDALDGEIKFFKCLYEGETAQIQSHISDTSIILSMDNNRNLDLD
SIIAEVRAQYEEIARKSKAEAEALYQTKFQELQLAAGRHGDDLKHTKNEISELTRLIQRL
RSEIESVKKQCANLETAIADAEQRGDCALKDARAKLDELEGALQQAKEELARMLREYQEL
LSVKLSLDIEIATYRKLLEGEECRMSGEYTNSVSISVINSSMAGMAGTGAGFGFSNAGTY
GYWPSSVSGGYSMLPGGCVTGSGNCSPRGEARTRLGSASEFRDSQGKTLALSSPTKKTMR
>sp|P35222|CTNB1_HUMAN Catenin beta-1 OS=Homo sapiens OX=9606 GN=CTNNB1 PE=1 SV=1
MATQADLMELDMAMEPDRKAAVSHWQQQSYLDSGIHSGATTTAPSLSGKGNPEEEDVDTS
QVLYEWEQGFSQSFTQEQVADIDGQYAMTRAQRVRAAMFPETLDEGMQIPSTQFDAAHPT
NVQRLAEPSQMLKHAVVNLINYQDDAELATRAIPELTKLLNDEDQVVVNKAAVMVHQLSK
KEASRHAIMRSPQMVSAIVRTMQNTNDVETARCTAGTLHNLSHHREGLLAIFKSGGIPAL
VKMLGSPVDSVLFYAITTLHNLLLHQEGAKMAVRLAGGLQKMVALLNKTNVKFLAITTDC

LQILAYGNQESKLIILASGGPQALVNIMRTYTYEKLLWTTSRVLKVLSVCSSNKPAIVEA
GGMQALGLHLTDPSQRLVQNCLWTLRNLSDAATKQEGMEGLLGTLVQLLGSDDINVVTCA
AGILSNLTCNNYKNKMMVCQVGGIEALVRTVLRAGDREDITEPAICALRHLTSRHQEAEM
AQNAVRLHYGLPVVVKLLHPPSHWPLIKATVGLIRNLALCPANHAPLREQGAIPRLVQLL
VRAHQDTQRRTSMGGTQQQFVEGVRMEEIVEGCTGALHILARDVHNRIVIRGLNTIPLFV
QLLYSPIENIQRVAAGVLCELAQDKEAAEAIEAEGATAPLTELLHSRNEGVATYAAAVLF
RMSEDKPQDYKKRLSVELTSSLFRTEPMAWNETADLGLDIGAQGEPLGYRQDDPSYRSFH
SGGYGQDALGMDPMMEHEMGGHHPGADYPVDGLPDLGHAQDLMDGLPPGDSNQLAWFDTD
L
>sp|P46940|IQGA1_HUMAN Ras GTPase-activating-like protein IQGAP1 OS=Homo sapiens OX=9606 GN=IQGAP1 PE=1 SV=1
MSAADEVDGLGVARPHYGSVLDNERLTAEEMDERRRQNVAYEYLCHLEEAKRWMEACLGE
DLPPTTELEEGLRNGVYLAKLGNFFSPKVVSLKKIYDREQTRYKATGLHFRHTDNVIQWL
NAMDEIGLPKIFYPETTDIYDRKNMPRCIYCIHALSLYLFKLGLAPQIQDLYGKVDFTEE
EINNMKTELEKYGIQMPAFSKIGGILANELSVDEAALHAAVIAINEAIDRRIPADTFAAL
KNPNAMLVNLEEPLASTYQDILYQAKQDKMTNAKNRTENSERERDVYEELLTQAEIQGNI
NKVNTFSALANIDLALEQGDALALFRALQSPALGLRGLQQQNSDWYLKQLLSDKQQKRQS
GQTDPLQKEELQSGVDAANSAAQQYQRRLAAVALINAAIQKGVAEKTVLELMNPEAQLPQ
VYPFAADLYQKELATLQRQSPEHNLTHPELSVAVEMLSSVALINRALESGDVNTVWKQLS
SSVTGLTNIEEENCQRYLDELMKLKAQAHAENNEFITWNDIQACVDHVNLVVQEEHERIL
AIGLINEALDEGDAQKTLQALQIPAAKLEGVLAEVAQHYQDTLIRAKREKAQEIQDESAV
LWLDEIQGGIWQSNKDTQEAQKFALGIFAINEAVESGDVGKTLSALRSPDVGLYGVIPEC
GETYHSDLAEAKKKKLAVGDNNSKWVKHWVKGGYYYYHNLETQEGGWDEPPNFVQNSMQL
SREEIQSSISGVTAAYNREQLWLANEGLITRLQARCRGYLVRQEFRSRMNFLKKQIPAIT
CIQSQWRGYKQKKAYQDRLAYLRSHKDEVVKIQSLARMHQARKRYRDRLQYFRDHINDII
KIQAFIRANKARDDYKTLINAEDPPMVVVRKFVHLLDQSDQDFQEELDLMKMREEVITLI
RSNQQLENDLNLMDIKIGLLVKNKITLQDVVSHSKKLTKKNKEQLSDMMMINKQKGGLKA
LSKEKREKLEAYQHLFYLLQTNPTYLAKLIFQMPQNKSTKFMDSVIFTLYNYASNQREEY
LLLRLFKTALQEEIKSKVDQIQEIVTGNPTVIKMVVSFNRGARGQNALRQILAPVVKEIM
DDKSLNIKTDPVDIYKSWVNQMESQTGEASKLPYDVTPEQALAHEEVKTRLDSSIRNMRA
VTDKFLSAIVSSVDKIPYGMRFIAKVLKDSLHEKFPDAGEDELLKIIGNLLYYRYMNPAI
VAPDAFDIIDLSAGGQLTTDQRRNLGSIAKMLQHAASNKMFLGDNAHLSIINEYLSQSYQ
KFRRFFQTACDVPELQDKFNVDEYSDLVTLTKPVIYISIGEIINTHTLLLDHQDAIAPEH
NDPIHELLDDLGEVPTIESLIGESSGNLNDPNKEALAKTEVSLTLTNKFDVPGDENAEMD
ARTILLNTKRLIVDVIRFQPGETLTEILETPATSEQEAEHQRAMQRRAIRDAKTPDKMKK
SKSVKEDSNLTLQEKKEKIQTGLKKLTELGTVDPKNKYQELINDIARDIRNQRRYRQRRK
AELVKLQQTYAALNSKATFYGEQVDYYKSYIKTCLDNLASKGKVSKKPREMKGKKSKKIS
LKYTAARLHEKGVLLEIEDLQVNQFKNVIFEISPTEEVGDFEVKAKFMGVQMETFMLHYQ
DLLQLQYEGVAVMKLFDRAKVNVNLLIFLLNKKFYGK
>sp|P30041|PRDX6_HUMAN Peroxiredoxin-6 OS=Homo sapiens OX=9606 GN=PRDX6 PE=1 SV=3
MPGGLLLGDVAPNFEANTTVGRIRFHDFLGDSWGILFSHPRDFTPVCTTELGRAAKLAPE
FAKRNVKLIALSIDSVEDHLAWSKDINAYNCEEPTEKLPFPIIDDRNRELAILLGMLDPA
EKDEKGMPVTARVVFVFGPDKKLKLSILYPATTGRNFDEILRVVISLQLTAEKRVATPVD
WKDGDSVMVLPTIPEEEAKKLFPKGVFTKELPSGKKYLRYTPQP
>sp|P08865|RSSA_HUMAN 40S ribosomal protein SA OS=Homo sapiens OX=9606 GN=RPSA PE=1 SV=4

MSGALDVLQMKEEDVLKFLAAGTHLGGTNLDFQMEQYIYKRKSDGIYIINLKRTWEKLLL
AARAIVAIENPADVSVISSRNTGQRAVLKFAAATGATPIAGRFTPGTFTNQIQAAFREPR
LLVVTDPRADHQPLTEASYVNLPTIALCNTDSPLRYVDIAIPCNNKGAHSVGLMWWMLAR
EVLRMRGTISREHPWEVMPDLYFYRDPEEIEKEEQAAAEKAVTKEEFQGEWTAPAPEFTA
TQPEVADWSEGVQVPSVPIQQFPTEDWSAQPATEDWSAAPTAQATEWVGATTDWS
>sp|P06702|S10A9_HUMAN Protein S100-A9 OS=Homo sapiens OX=9606 GN=S100A9 PE=1 SV=1
MTCKMSQLERNIETIINTFHQYSVKLGHPDTLNQGEFKELVRKDLQNFLKKENKNEKVIE
HIMEDLDTNADKQLSFEEFIMLMARLTWASHEKMHEGDEGPGHHHKPGLGEGTP
>sp|P0DOX5|IGG1_HUMAN Immunoglobulin gamma-1 heavy chain OS=Homo sapiens OX=9606 PE=1 SV=2
QVQLVQSGGGVVQPGRSLRLSCAASGFTFSRYTIHWVRQAPGKGLEWVAVMSYNGNNKHY
ADSVNGRFTISRNDSKNTLYLNMNSLRPEDTAVYYCARIRDTAMFFAHWGQGTLVTVSSA
STKGPSVFPLAPSSKSTSGGTAALGCLVKDYFPEPVTVSWNSGALTSGVHTFPAVLQSSG
LYSLSSVVTVPSSSLGTQTYICNVNHKPSNTKVDKKVEPKSCDKTHTCPPCPAPELLGGP
SVFLFPPKPKDTLMISRTPEVTCVVVDVSHEDPEVKFNWYVDGVEVHNAKTKPREEQYNS
TYRVVSVLTVLHQDWLNGKEYKCKVSNKALPAPIEKTISKAKGQPREPQVYTLPPSRDEL
TKNQVSLTCLVKGFYPSDIAVEWESNGQPENNYKTTPPVLDSDGSFFLYSKLTVDKSRWQ
QGNVFSCSVMHEALHNHYTQKSLSLSPGK
>sp|P01857|IGHG1_HUMAN Immunoglobulin heavy constant gamma 1 OS=Homo sapiens OX=9606 GN=IGHG1 PE=1 SV=1
ASTKGPSVFPLAPSSKSTSGGTAALGCLVKDYFPEPVTVSWNSGALTSGVHTFPAVLQSS
GLYSLSSVVTVPSSSLGTQTYICNVNHKPSNTKVDKKVEPKSCDKTHTCPPCPAPELLGG
PSVFLFPPKPKDTLMISRTPEVTCVVVDVSHEDPEVKFNWYVDGVEVHNAKTKPREEQYN
STYRVVSVLTVLHQDWLNGKEYKCKVSNKALPAPIEKTISKAKGQPREPQVYTLPPSRDE
LTKNQVSLTCLVKGFYPSDIAVEWESNGQPENNYKTTPPVLDSDGSFFLYSKLTVDKSRW
QQGNVFSCSVMHEALHNHYTQKSLSLSPGK
>sp|Q9C075|K1C23_HUMAN Keratin, type I cytoskeletal 23 OS=Homo sapiens OX=9606 GN=KRT23 PE=1 SV=2
MNSGHSFSQTPSASFHGAGGGWGRPRSFPRAPTVHGGAGGARISLSFTTRSCPPPGGSWG
SGRSSPLLGGNGKATMQNLNDRLASYLEKVRALEEANMKLESRILKWHQQRDPGSKKDYS
QYEENITHLQEQIVDGKMTNAQIILLIDNARMAVDDFNLKYENEHSFKKDLEIEVEGLRR
TLDNLTIVTTDLEQEVEGMRKELILMKKHHEQEMEKHHVPSDFNVNVKVDTGPREDLIKV
LEDMRQEYELIIKKKHRDLDTWYKEQSAAMSQEAASPATVQSRQGDIHELKRTFQALEID
LQTQYSTKSALENMLSETQSRYSCKLQDMQEIISHYEEELTQLRHELERQNNEYQVLLGI
KTHLEKEITTYRRLLEGESEGTREESKSSMKVSATPKIKAITQETINGRLVLCQVNEIQK
HA
>sp|Q7Z3Z0|K1C25_HUMAN Keratin, type I cytoskeletal 25 OS=Homo sapiens OX=9606 GN=KRT25 PE=1 SV=1
MSLRLSSASRRSCPRPTTGSLRLYGGGTSFGTGNSCGISGIGSGFSSAFGGSSSGGNTGG
GNPCAGFTVNERGLLSGNEKVTMQNLNDRLASYLDSVHALEEANADLEQKIKGWYEKFGP
GSCRGLDHDYSRYFPIIDDLKNQIIASTTSNANAVLQIDNARLTADDFRLKYENELALHQ
SVEADVNGLRRVLDEITLCRTDLEIQYETLSEEMTYLKKNHKEEMQVLQCAAGGNVNVEM
NAAPGVDLTVLLNNMRAEYEALAEQNRRDAEAWFNEKSASLQQQISEDVGATTSARNELT
EMKRTLQTLEIELQSLLATKHSLECSLTETESNYCAQLAQIQAQIGALEEQLHQVRTETE
GQKLEYEQLLDIKLHLEKEIETYCLLIGGDDGACKSGGYKSKDYGSGNVGSQVKDPAKAI
VVKKVLEEVDQRSKILTTRLHSLEEKSQSN

>sp|Q14204|DYHC1_HUMAN Cytoplasmic dynein 1 heavy chain 1 OS=Homo sapiens OX=9606 GN=DYNC1H1 PE=1 SV=5
MSEPGGGGGEDGSAGLEVSAVQNVADVSVLQKHLRKLVPLLLEDGGEAPAALEAALEEKS
ALEQMRKFLSDPQVHTVLVERSTLKEDVGDEGEEEKEFISYNINIDIHYGVKSNSLAFIK
RTPVIDADKPVSSQLRVLTLSEDSPYETLHSFISNAVAPFFKSYIRESGKADRDGDKMAP
SVEKKIAELEMGLLHLQQNIEIPEISLPIHPMITNVAKQCYERGEKPKVTDFGDKVEDPT
FLNQLQSGVNRWIREIQKVTKLDRDPASGTALQEISFWLNLERALYRIQEKRESPEVLLT
LDILKHGKRFHATVSFDTDTGLKQALETVNDYNPLMKDFPLNDLLSATELDKIRQALVAI
FTHLRKIRNTKYPIQRALRLVEAISRDLSSQLLKVLGTRKLMHVAYEEFEKVMVACFEVF
QTWDDEYEKLQVLLRDIVKRKREENLKMVWRINPAHRKLQARLDQMRKFRRQHEQLRAVI
VRVLRPQVTAVAQQNQGEVPEPQDMKVAEVLFDAADANAIEEVNLAYENVKEVDGLDVSK
EGTEAWEAAMKRYDERIDRVETRITARLRDQLGTAKNANEMFRIFSRFNALFVRPHIRGA
IREYQTQLIQRVKDDIESLHDKFKVQYPQSQACKMSHVRDLPPVSGSIIWAKQIDRQLTA
YMKRVEDVLGKGWENHVEGQKLKQDGDSFRMKLNTQEIFDDWARKVQQRNLGVSGRIFTI
ESTRVRGRTGNVLKLKVNFLPEIITLSKEVRNLKWLGFRVPLAIVNKAHQANQLYPFAIS
LIESVRTYERTCEKVEERNTISLLVAGLKKEVQALIAEGIALVWESYKLDPYVQRLAETV
FNFQEKVDDLLIIEEKIDLEVRSLETCMYDHKTFSEILNRVQKAVDDLNLHSYSNLPIWV
NKLDMEIERILGVRLQAGLRAWTQVLLGQAEDKAEVDMDTDAPQVSHKPGGEPKIKNVVH
ELRITNQVIYLNPPIEECRYKLYQEMFAWKMVVLSLPRIQSQRYQVGVHYELTEEEKFYR
NALTRMPDGPVALEESYSAVMGIVSEVEQYVKVWLQYQCLWDMQAENIYNRLGEDLNKWQ
ALLVQIRKARGTFDNAETKKEFGPVVIDYGKVQSKVNLKYDSWHKEVLSKFGQMLGSNMT
EFHSQISKSRQELEQHSVDTASTSDAVTFITYVQSLKRKIKQFEKQVELYRNGQRLLEKQ
RFQFPPSWLYIDNIEGEWGAFNDIMRRKDSAIQQQVANLQMKIVQEDRAVESRTTDLLTD
WEKTKPVTGNLRPEEALQALTIYEGKFGRLKDDREKCAKAKEALELTDTGLLSGSEERVQ
VALEELQDLKGVWSELSKVWEQIDQMKEQPWVSVQPRKLRQNLDALLNQLKSFPARLRQY
ASYEFVQRLLKGYMKINMLVIELKSEALKDRHWKQLMKRLHVNWVVSELTLGQIWDVDLQ
KNEAIVKDVLLVAQGEMALEEFLKQIREVWNTYELDLVNYQNKCRLIRGWDDLFNKVKEH
INSVSAMKLSPYYKVFEEDALSWEDKLNRIMALFDVWIDVQRRWVYLEGIFTGSADIKHL
LPVETQRFQSISTEFLALMKKVSKSPLVMDVLNIQGVQRSLERLADLLGKIQKALGEYLE
RERSSFPRFYFVGDEDLLEIIGNSKNVAKLQKHFKKMFAGVSSIILNEDNSVVLGISSRE
GEEVMFKTPVSITEHPKINEWLTLVEKEMRVTLAKLLAESVTEVEIFGKATSIDPNTYIT
WIDKYQAQLVVLSAQIAWSENVETALSSMGGGDAAPLHSVLSNVEVTLNVLADSVLMEQ
PPLRRRKLEHLITELVHQRDVTRSLIKSKIDNAKSFEWLSQMRFYFDPKQTDVLQQLSIQ
MANAKFNYGFEYLGVQDKLVQTPLTDRCYLTMTQALEARLGGSPFGPAGTGKTESVKALG
HQLGRFVLVFNCDETFDFQAMGRIFVGLCQVGAWGCFDEFNRLEERMLSAVSQQVQCIQE
ALREHSNPNYDKTSAPITCELLNKQVKVSPDMAIFITMNPGYAGRSNLPDNLKKLFRSLA
MTKPDRQLIAQVMLYSQGFRTAEVLANKIVPFFKLCDEQLSSQSHYDFGLRALKSVLVSA
GNVKRERIQKIKREKEERGEAVDEGEIAENLPEQEILIQSVCETMVPKLVAEDIPLLFSL
LSDVFPGVQYHRGEMTALREELKKVCQEMYLTYGDGEEVGGMWVEKVLQLYQITQINHGL
MMVGPSGSGKSMAWRVLLKALERLEGVEGVAHIIDPKAISKDHLYGTLDPNTREWTDGLF
THVLRKIIDSVRGELQKRQWIVFDGDVDPEWVENLNSVLDDNKLLTLPNGERLSLPPNVR
IMFEVQDLKYATLATVSRCGMVWFSEDVLSTDMIFNNFLARLRSIPLDEGEDEAQRRRKG
KEDEGEEAASPMLQIQRDAATIMQPYFTSNGLVTKALEHAFQLEHIMDLTRLRCLGSLFS
MLHQACRNVAQYNANHPDFPMQIEQLERYIQRYLVYAILWSLSGDSRLKMRAELGEYIRR
ITTVPLPTAPNIPIIDYEVSISGEWSPWQAKVPQIEVETHKVAAPDVVVPTLDTVRHEAL
LYTWLAEHKPLVLCGPPGSGKTMTLFSALRALPDMEVVGLNFSSATTPELLLKTFDHYCE
YRRTPNGVVLAPVQLGKWLVLFCDEINLPDMDKYGTQRVISFIRQMVEHGGFYRTSDQTW

VKLERIQFVGACNPPTDPGRKPLSHRFLRHVPVVYVDYPGPASLTQIYGTFNRAMLRLIP
SLRTYAEPLTAAMVEFYTMSQERFTQDTQPHYIYSPREMTRWVRGIFEALRPLETLPVEG
LIRIWAHEALRLFQDRLVEDEERRWTDENIDTVALKHFPNIDREKAMSRPILYSNWLSKD
YIPVDQEELRDYVKARLKVFYEEELDVPLVLFNEVLDHVLRIDRIFRQPQGHLLLIGVSG
AGKTTLSRFVAWMNGLSVYQIKVHRKYTGEDFDEDLRTVLRRSGCKNEKIAFIMDESNVL
DSGFLERMNTLLANGEVPGLFEGDEYATLMTQCKEGAQKEGLMLDSHEELYKWFTSQVIR
NLHVVFTMNPSSEGLKDRAATSPALFNRCVLNWFGDWSTEALYQVGKEFTSKMDLEKPNY
IVPDYMPVVYDKLPQPPSHREAIVNSCVFVHQTLHQANARLAKRGGRTMAITPRHYLDFI
NHYANLFHEKRSELEEQQMHLNVGLRKIKETVDQVEELRRDLRIKSQELEVKNAAANDKL
KKMVKDQQEAEKKKVMSQEIQEQLHKQQEVIADKQMSVKEDLDKVEPAVIEAQNAVKSIK
KQHLVEVRSMANPPAAVKLALESICLLLGESTTDWKQIRSIIMRENFIPTIVNFSAEEIS
DAIREKMKKNYMSNPSYNYEIVNRASLACGPMVKWAIAQLNYADMLKRVEPLRNELQKLE
DDAKDNQQKANEVEQMIRDLEASIARYKEEYAVLISEAQAIKADLAAVEAKVNRSTALLK
SLSAERERWEKTSETFKNQMSTIAGDCLLSAAFIAYAGYFDQQMRQNLFTTWSHHLQQAN
IQFRTDIARTEYLSNADERLRWQASSLPADDLCTENAIMLKRFNRYPLIIDPSGQATEFI
MNEYKDRKITRTSFLDDAFRKNLESALRFGNPLLVQDVESYDPVLNPVLNREVRRTGGRV
LITLGDQDIDLSPSFVIFLSTRDPTVEFPPDLCSRVTFVNFTVTRSSLQSQCLNEVLKAE
RPDVDEKRSDLLKLQGEFQLRLRQLEKSLLQALNEVKGRILDDDTIITTLENLKREAAEV
TRKVEETDIVMQEVETVSQQYLPLSTACSSIYFTMESLKQIHFLYQYSLQFFLDIYHNVL
YENPNLKGVTDHTQRLSIITKDLFQVAFNRVARGMLHQDHITFAMLLARIKLKGTVGEPT
YDAEFQHFLRGNEIVLSAGSTPRIQGLTVEQAEAVVRLSCLPAFKDLIAKVQADEQFGIW
LDSSSPEQTVPYLWSEETPATPIGQAIHRLLLIQAFRPDRLLAMAHMFVSTNLGESFMSI
MEQPLDLTHIVGTEVKPNTPVLMCSVPGYDASGHVEDLAAEQNTQITSIAIGSAEGFNQA
DKAINTAVKSGRWVMLKNVHLAPGWLMQLEKKLHSLQPHACFRLFLTMEINPKVPVNLLR
AGRIFVFEPPPGVKANMLRTFSSIPVSRICKSPNERARLYFLLAWFHAIIQERLRYAPLG
WSKKYEFGESDLRSACDTVDTWLDDTAKGRQNISPDKIPWSALKTLMAQSIYGGRVDNEF
DQRLLNTFLERLFTTRSFDSEFKLACKVDGHKDIQMPDGIRREEFVQWVELLPDTQTPSW
LGLPNNAERVLLTTQGVDMISKMLKMQMLEDEDDLAYAETEKKTRTDSTSDGRPAWMRTL
HTTASNWLHLIPQTLSHLKRTVENIKDPLFRFFEREVKMGAKLLQDVRQDLADVVQVCEG
KKKQTNYLRTLINELVKGILPRSWSHYTVPAGMTVIQWVSDFSERIKQLQNISLAAASGG
AKELKNIHVCLGGLFVPEAYITATRQYVAQANSWSLEELCLEVNVTTSQGATLDACSFGV
TGLKLQGATCNNNKLSLSNAISTALPLTQLRWVKQTNTEKKASVVTLPVYLNFTRADLIF
TVDFEIATKEDPRSFYERGVAVLCTE
>sp|P05787|K2C8_HUMAN Keratin, type II cytoskeletal 8 OS=Homo sapiens OX=9606 GN=KRT8 PE=1 SV=7
MSIRVTQKSYKVSTSGPRAFSSRSYTSGPGSRISSSSFSRVGSSNFRGGLGGGYGGASGM
GGITAVTVNQSLLSPLVLEVDPNIQAVRTQEKEQIKTLNNKFASFIDKVRFLEQQNKMLE
TKWSLLQQQKTARSNMDNMFESYINNLRRQLETLGQEKLKLEAELGNMQGLVEDFKNKYE
DEINKRTEMENEFVLIKKDVDEAYMNKVELESRLEGLTDEINFLRQLYEEEIRELQSQIS
DTSVVLSMDNSRSLDMDSIIAEVKAQYEDIANRSRAEAESMYQIKYEELQSLAGKHGDDL
RRTKTEISEMNRNISRLQAEIEGLKGQRASLEAAIADAEQRGELAIKDANAKLSELEAAL
QRAKQDMARQLREYQELMNVKLALDIEIATYRKLLEGEESRLESGMQNMSIHTKTTSGYA
GGLSSAYGGLTSPGLSYSLGSSFGSGAGSSSFSRTSSSRAVVVKKIETRDGKLVSESSDV
LPK
>sp|P25311|ZA2G_HUMAN Zinc-alpha-2-glycoprotein OS=Homo sapiens OX=9606 GN=AZGP1 PE=1 SV=2
MVRMVPVLLSLLLLLGPAVPQENQDGRYSLTYIYTGLSKHVEDVPAFQALGSLNDLQFFR

YNSKDRKSQPMGLWRQVEGMEDWKQDSQLQKAREDIFMETLKDIVEYYNDSNGSHVLQGR
FGCEIENNRSSGAFWKYYYDGKDYIEFNKEIPAWVPFDPAAQITKQKWEAEPVYVQRAKA
YLEEECPATLRKYLKYSKNILDRQDPPSVVVTSHQAPGEKKKLKCLAYDFYPGKIDVHWT
RAGEVQEPELRGDVLHNGNGTYQSWVVVAVPPQDTAPYSCHVQHSSLAQPLVVPWEAS
>sp|P08729|K2C7_HUMAN Keratin, type II cytoskeletal 7 OS=Homo sapiens OX=9606 GN=KRT7 PE=1 SV=5
MSIHFSSPVFTSRSAAFSGRGAQVRLSSARPGGLGSSSLYGLGASRPRVAVRSAYGGPVG
AGIREVTINQSLLAPLRLDADPSLQRVRQEESEQIKTLNNKFASFIDKVRFLEQQNKLLE
TKWTLLQEQKSAKSSRLPDIFEAQIAGLRGQLEALQVDGGRLEAELRSMQDVVEDFKNKY
EDEINHRTAAENEFVVLKKDVDAAYMSKVELEAKVDALNDEINFLRTLNETELTELQSQI
SDTSVVLSMDNSRSLDLDGIIAEVKAQYEEMAKCSRAEAEAWYQTKFETLQAQAGKHGDD
LRNTRNEISEMNRAIQRLQAEIDNIKNQRAKLEAAIAEAEERGELALKDARAKQEELEAA
LQRGKQDMARQLREYQELMSVKLALDIEIATYRKLLEGEESRLAGDGVGAVNISVMNSTG
GSSSGGGIGLTLGGTMGSNALSFSSSAGPGLLKAYSIRTASASRRSARD
>sp|P02675|FIBB_HUMAN Fibrinogen beta chain OS=Homo sapiens OX=9606 GN=FGB PE=1 SV=2
MKRMVSWSFHKLKTMKHLLLLLLCVFLVKSQGVNDNEEGFFSARGHRPLDKKREEAPSLR
PAPPPISGGGYRARPAKAAATQKKVERKAPDAGGCLHADPDLGVLCPTGCQLQEALLQQE
RPIRNSVDELNNNVEAVSQTSSSSFQYMYLLKDLWQKRQKQVKDNENVVNEYSSELEKHQ
LYIDETVNSNIPTNLRVLRSILENLRSKIQKLESDVSAQMEYCRTPCTVSCNIPVVSGKE
CEEIIRKGGETSEMYLIQPDSSVKPYRVYCDMNTENGGWTVIQNRQDGSVDFGRKWDPYK
QGFGNVATNTDGKNYCGLPGEYWLGNDKISQLTRMGPTELLIEMEDWKGDKVKAHYGGFT
VQNEANKYQISVNKYRGTAGNALMDGASQLMGENRTMTIHNGMFFSTYDRDNDGWLTSDP
RKQCSKEDGGGWWYNRCHAANPNGRYYWGGQYTWDMAKHGTDDGVVWMNWKGSWYSMRKM
SMKIRPFFPQQ
>sp|P30086|PEBP1_HUMAN Phosphatidylethanolamine-binding protein 1 OS=Homo sapiens OX=9606 GN=PEBP1 PE=1 SV=3
MPVDLSKWSGPLSLQEVDEQPQHPLHVTYAGAAVDELGKVLTPTQVKNRPTSISWDGLDS
GKLYTLVLTDPDAPSRKDPKYREWHHFLVVNMKGNDISSGTVLSDYVGSGPPKGTGLHRY
VWLVYEQDRPLKCDEPILSNRSGDHRGKFKVASFRKKYELRAPVAGTCYQAEWDDYVPKL
YEQLSGK
>sp|Q14134|TRI29_HUMAN Tripartite motif-containing protein 29 OS=Homo sapiens OX=9606 GN=TRIM29 PE=1 SV=2
MEAADASRSNGSSPEARDARSPSGPSGSLENGTKADGKDAKTTNGHGGEAAEGKSLGSAL
KPGEGRSALFAGNEWRRPIIQFVESGDDKNSNYFSMDSMEGKRSPYAGLQLGAAKKPPVT
FAEKGELRKSIFSESRKPTVSIMEPGETRRNSYPRADTGLFSRSKSGSEEVLCDSCIGNK
QKAVKSCLVCQASFCELHLKPHLEGAAFRDHQLLEPIRDFEARKCPVHGKTMELFCQTDQ
TCICYLCMFQEHKNHSTVTVEEAKAEKETELSLQKEQLQLKIIEIEDEAEKWQKEKDRIK
SFTTNEKAILEQNFRDLVRDLEKQKEEVRAALEQREQDAVDQVKVIMDALDERAKVLHED
KQTREQLHSISDSVLFLQEFGALMSNYSLPPPLPTYHVLLEGEGLGQSLGNFKDDLLNVC
MRHVEKMCKADLSRNFIERNHMENGGDHRYVNNYTNSFGGEWSAPDTMKRYSMYLTPKGG
VRTSYQPSSPGRFTKETTQKNFNNLYGTKGNYTSRVWEYSSSIQNSDNDLPVVQGSSSFS
LKGYPSLMRSQSPKAQPQTWKSGKQTMLSHYRPFYVNKGNGIGSNEAP
>sp|Q02487|DSC2_HUMAN Desmocollin-2 OS=Homo sapiens OX=9606 GN=DSC2 PE=1 SV=1
MEAARPSGSWNGALCRLLLLTLAILIFASDACKNVTLHVPSKLDAEKLVGRVNLKECFTA
ANLIHSSDPDFQILEDGSVYTTNTILLSSEKRSFTILLSNTENQEKKKIFVFLEHQTKVL

KKRHTKEKVLRRAKRRWAPIPCSMLENSLGPFPLFLQQVQSDTAQNYTIYYSIRGPGVDQ
EPRNLFYVERDTGNLYCTRPVDREQYESFEIIAFATTPDGYTPELPLPLIIKIEDENDNY
PIFTEETYTFTIFENCRVGTTVGQVCATDKDEPDTMHTRLKYSIIGQVPPSPTLFSMHPT
TGVITTTSSQLDRELIDKYQLKIKVQDMDGQYFGLQTTSTCIINIDDVNDHLPTFTRTSY
VTSVEENTVDVEILRVTVEDKDLVNTANWRANYTILKGNENGNFKIVTDAKTNEGVLCVV
KPLNYEEKQQMILQIGVVNEAPFSREASPRSAMSTATVTVNVEDQDEGPECNPPIQTVRM
KENAEVGTTSNGYKAYDPETRSSSGIRYKKLTDPTGWVTIDENTGSIKVFRSLDREAETI
KNGIYNITVLASDQGGRTCTGTLGIILQDVNDNSPFIPKKTVIICKPTMSSAEIVAVDPD
EPIHGPPFDFSLESSTSEVQRMWRLKAINDTAARLSYQNDPPFGSYVVPITVRDRLGMSS
VTSLDVTLCDCITENDCTHRVDPRIGGGGVQLGKWAILAILLGIALLFCILFTLVCGASG
TSKQPKVIPDDLAQQNLIVSNTEAPGDDKVYSANGFTTQTVGASAQGVCGTVGSGIKNGG
QETIEMVKGGHQTSESCRGAGHHHTLDSCRGGHTEVDNCRYTYSEWHSFTQPRLGEKVYL
CNQDENHKHAQDYVLTYNYEGRGSVAGSVGCCSERQEEDGLEFLDNLEPKFRTLAEACMK
R
>sp|Q7RTS7|K2C74_HUMAN Keratin, type II cytoskeletal 74 OS=Homo sapiens OX=9606 GN=KRT74 PE=1 SV=2
MSRQLNIKSSGDKGNFSVHSAVVPRKAVGSLASYCAAGRGAGAGFGSRSLYSLGGNRRIS
FNVAGGGVRAGGYGFRPGSGYGGGRASGFAGSMFGSVALGPACLSVCPPGGIHQVTVNKS
LLAPLNVELDPEIQKVRAQEREQIKVLNDKFASFIDKVRFLEQQNQVLETKWELLQQLDL
NNCKKNLEPILEGYISNLRKQLETLSGDRVRLDSELRSMRDLVEDYKKRYEVEINRRTTA
ENEFVVLKKDADAAYAVKVELQAKVDSLDKEIKFLKCLYDAEIAQIQTHASETSVILSMD
NNRDLDLDSIIAEVRMHYEEIALKSKAEAEALYQTKIQELQLAASRHGDDLKHTRSEMVE
LNRLIQRIRCEIGNVKKQRASLETAIADAEQRGDNALKDAQAKLDELEGALHQAKEELAR
MLREYQELMSLKLALDMEIATYRKLLEGEECRMSGENPSSVSISVISSSSYSYHHPSSAG
VDLGASAVAGSSGSTQSGQTKTTEARGGDLKDTQGKSTPASIPARKATR
>sp|P07900|HS90A_HUMAN Heat shock protein HSP 90-alpha OS=Homo sapiens OX=9606 GN=HSP90AA1 PE=1 SV=5
MPEETQTQDQPMEEEEVETFAFQAEIAQLMSLIINTFYSNKEIFLRELISNSSDALDKIR
YESLTDPSKLDSGKELHINLIPNKQDRTLTIVDTGIGMTKADLINNLGTIAKSGTKAFME
ALQAGADISMIGQFGVGFYSAYLVAEKVTVITKHNDDEQYAWESSAGGSFTVRTDTGEPM
GRGTKVILHLKEDQTEYLEERRIKEIVKKHSQFIGYPITLFVEKERDKEVSDDEAEEKED
KEEEKEKEEKESEDKPEIEDVGSDEEEEKKDGDKKKKKKIKEKYIDQEELNKTKPIWTRN
PDDITNEEYGEFYKSLTNDWEDHLAVKHFSVEGQLEFRALLFVPRRAPFDLFENRKKKNN
IKLYVRRVFIMDNCEELIPEYLNFIRGVVDSEDLPLNISREMLQQSKILKVIRKNLVKKC
LELFTELAEDKENYKKFYEQFSKNIKLGIHEDSQNRKKLSELLRYYTSASGDEMVSLKDY
CTRMKENQKHIYYITGETKDQVANSAFVERLRKHGLEVIYMIEPIDEYCVQQLKEFEGKT
LVSVTKEGLELPEDEEEKKKQEEKKTKFENLCKIMKDILEKKVEKVVVSNRLVTSPCCIV
TSTYGWTANMERIMKAQALRDNSTMGYMAAKKHLEINPDHSIIETLRQKAEADKNDKSVK
DLVILLYETALLSSGFSLEDPQTHANRIYRMIKLGLGIDEDDPTADDTSAAVTEEMPPLE
GDDDTSRMEEVD
>sp|Q58FG0|HS905_HUMAN Putative heat shock protein HSP 90-alpha A5 OS=Homo sapiens OX=9606 GN=HSP90AA5P PE=2 SV=1
MGFHHVGQAGLELLTSGHPALERRPEYLEERRIKEIVKKHSQFIGYPITLFVEKKRNKQV
SDAEAEKKEDKRKKKKESNDKPEIEDVGSDEEEEKKDADKKKKKSKEKYIDQELNKTKPI
WTRNPDAITNEEYGEFHQSLTNNWEDHLAVKHFSVEGQLEELKDSRRVMKANQKHIYYIT
GETKDQVANSAFVECLQKHGLEVIYMIELIDKYCVQQLKELESKTVVSVAKEGLELPEDE
EEKKKQEEKKTKFENLCKIMKDMLEKKVKKVVVSNCMEDPQRHTNKIYRMIKLGLGVDEY

DPTANDINAAITKEMPPLRGGDDTSRMEEVGGSG
>sp|P07237|PDIA1_HUMAN Protein disulfide-isomerase OS=Homo sapiens OX=9606 GN=P4HB PE=1 SV=3
MLRRALLCLAVAALVRADAPEEEDHVLVLRKSNFAEALAAHKYLLVEFYAPWCGHCKALA
PEYAKAAGKLKAEGSEIRLAKVDATEESDLAQQYGVRGYPTIKFFRNGDTASPKEYTAGR
EADDIVNWLKKRTGPAATTLPDGAAAESLVESSEVAVIGFFKDVESDSAKQFLQAAEAID
DIPFGITSNSDVFSKYQLDKDGVVLFKKFDEGRNNFEGEVTKENLLDFIKHNQLPLVIEF
TEQTAPKIFGGEIKTHILLFLPKSVSDYDGKLSNFKTAAESFKGKILFIFIDSDHTDNQR
ILEFFGLKKEECPAVRLITLEEEMTKYKPESEELTAERITEFCHRFLEGKIKPHLMSQEL
PEDWDKQPVKVLVGKNFEDVAFDEKKNVFVEFYAPWCGHCKQLAPIWDKLGETYKDHENI
VIAKMDSTANEVEAVKVHSFPTLKFFPASADRTVIDYNGERTLDGFKKFLESGGQDGAGD
DDDLEDLEEAEEPDMEEDDDQKAVKDEL
>sp|P08727|K1C19_HUMAN Keratin, type I cytoskeletal 19 OS=Homo sapiens OX=9606 GN=KRT19 PE=1 SV=4
MTSYSYRQSSATSSFGGLGGGSVRFGPGVAFRAPSIHGGSGGRGVSVSSARFVSSSSSGA
YGGGYGGVLTASDGLLAGNEKLTMQNLNDRLASYLDKVRALEAANGELEVKIRDWYQKQG
PGPSRDYSHYYTTIQDLRDKILGATIENSRIVLQIDNARLAADDFRTKFETEQALRMSVE
ADINGLRRVLDELTLARTDLEMQIEGLKEELAYLKKNHEEEISTLRGQVGGQVSVEVDSA
PGTDLAKILSDMRSQYEVMAEQNRKDAEAWFTSRTEELNREVAGHTEQLQMSRSEVTDLR
RTLQGLEIELQSQLSMKAALEDTLAETEARFGAQLAHIQALISGIEAQLGDVRADSERQN
QEYQRLMDIKSRLEQEIATYRSLLEGQEDHYNNLSASKVL
>sp|P12814|ACTN1_HUMAN Alpha-actinin-1 OS=Homo sapiens OX=9606 GN=ACTN1 PE=1 SV=2
MDHYDSQQTNDYMQPEEDWDRDLLLDPAWEKQQRKTFTAWCNSHLRKAGTQIENIEEDFR
DGLKLMLLLEVISGERLAKPERGKMRVHKISNVNKALDFIASKGVKLVSIGAEEIVDGNV
KMTLGMIWTIILRFAIQDISVEETSAKEGLLLWCQRKTAPYKNVNIQNFHISWKDGLGFC
ALIHRHRPELIDYGKLRKDDPLTNLNTAFDVAEKYLDIPKMLDAEDIVGTARPDEKAIMT
YVSSFYHAFSGAQKAETAANRICKVLAVNQENEQLMEDYEKLASDLLEWIRRTIPWLENR
VPENTMHAMQQKLEDFRDYRRLHKPPKVQEKCQLEINFNTLQTKLRLSNRPAFMPSEGRM
VSDINNAWGCLEQVEKGYEEWLLNEIRRLERLDHLAEKFRQKASIHEAWTDGKEAMLRQK
DYETATLSEIKALLKKHEAFESDLAAHQDRVEQIAAIAQELNELDYYDSPSVNARCQKIC
DQWDNLGALTQKRREALERTEKLLETIDQLYLEYAKRAAPFNNWMEGAMEDLQDTFIVHT
IEEIQGLTTAHEQFKATLPDADKERLAILGIHNEVSKIVQTYHVNMAGTNPYTTITPQEI
NGKWDHVRQLVPRRDQALTEEHARQQHNERLRKQFGAQANVIGPWIQTKMEEIGRISIEM
HGTLEDQLSHLRQYEKSIVNYKPKIDQLEGDHQLIQEALIFDNKHTNYTMEHIRVGWEQL
LTTIARTINEVENQILTRDAKGISQEQMNEFRASFNHFDRDHSGTLGPEEFKACLISLGY
DIGNDPQGEAEFARIMSIVDPNRLGVVTFQAFIDFMSRETADTDTADQVMASFKILAGDK
NYITMDELRRELPPDQAEYCIARMAPYTGPDSVPGALDYMSFSTALYGESDL
>sp|P37802|TAGL2_HUMAN Transgelin-2 OS=Homo sapiens OX=9606 GN=TAGLN2 PE=1 SV=3
MANRGPAYGLSREVQQKIEKQYDADLEQILIQWITTQCRKDVGRPQPGRENFQNWLKDGT
VLCELINALYPEGQAPVKKIQASTMAFKQMEQISQFLQAAERYGINTTDIFQTVDLWEGK
NMACVQRTLMNLGGLAVARDDGLFSGDPNWFPKKSKENPRNFSDNQLQEGKNVIGLQMGT
NRGASQAGMTGYGMPRQIL
>sp|P0C7H8|KRA23_HUMAN Keratin-associated protein 2-3 OS=Homo sapiens OX=9606 GN=KRTAP2-3 PE=1 SV=2
MTGSCCGSTLSSLSYGGGCCQPCCCRDPCCCRPVTCQTTVCRPVTCVPRCTRPICEPCRR

PVCCDPCSLQEGCCRPITCCPSSCTAVVCRPCCWATTCCQPVSVQSPCCRPPCGQPTPCS
TTCRTSSC
>sp|Q9BYU5|KRA21_HUMAN Keratin-associated protein 2-1 OS=Homo sapiens OX=9606 GN=KRTAP2-1 PE=2 SV=2
MTGSCCGSTFSSLSYGGGCCQPCCCRDPCCCRPVTCQTTVCRPVTCVPRCTRPICEPCRR
PVCCDPCSLQEGCCRPITCCPSSCTAVVCRPCCWATTCCQPVSVQSPCCRPPCGQPTPCS
TTCRTSSC
>sp|Q9BYR9|KRA24_HUMAN Keratin-associated protein 2-4 OS=Homo sapiens OX=9606 GN=KRTAP2-4 PE=1 SV=1
MTGSCCGSTLSSLSYGGGCCQPCCCRDPCCCRPVTCQTTVCRPVTCVPRCTRPICEPCRR
PVCCDPCSLQEGCCRPITCCPSSCTAVVCRPCCWATTCCQPVSVQSPCCRPPCGQPTPCS
TTCRTSSC
>sp|Q9BYT5|KRA22_HUMAN Keratin-associated protein 2-2 OS=Homo sapiens OX=9606 GN=KRTAP2-2 PE=2 SV=3
MTGSCCGSTFSSLSYGGGCCQPCCCRDPCCCRPVTCQTTVCRPVTCVPRCTRPICEPCRR
PVCCDPCSLQEGCCRPITCCPSSCTAVVCRPCCWATTCCQPVSVQSPCGQPTPCSTTCRT
SSC
>sp|Q07283|TRHY_HUMAN Trichohyalin OS=Homo sapiens OX=9606 GN=TCHH PE=1 SV=2
MSPLLRSICDITEIFNQYVSHDCDGAALTKKDLKNLLEREFGAVLRRPHDPKTVDLILEL
LDLDSNGRVDFNEFLLFIFKVAQACYYALGQATGLDEEKRARCDGKESLLQDRRQEEDQR
RFEPRDRQLEEEPGQRRRQKRQEQERELAEGEEQSEKQERLEQRDRQRRDEELWRQRQEW
QEREERRAEEEQLQSCKGHETEEFPDEEQLRRRELLELRRKGREEKQQQRRERQDRVFQE
EEEKEWRKRETVLRKEEEKLQEEEPQRQRELQEEEEQLRKLERQELRRERQEEEQQQQRL
RREQQLRRKQEEERREQQEERREQQERREQQEERREQQLRREQEERREQQLRREQEEERR
EQQLRREQEEERREQQLRREQQLRREQQLRREQQLRREQQLRREQQLRREQQLRREQQLR
REQQLRREQEEERHEQKHEQERREQRLKREQEERRDWLKREEETERHEQERRKQQLKRDQ
EEERRERWLKLEEEERREQQERREQQLRREQEERREQRLKRQEEEERLQQRLRSEQQLRR
EQEERREQLLKREEEKRLEQERREQRLKREQEERRDQLLKREEERRQQRLKREQEERLEQ
RLKREEVERLEQEERREQRLKREEPEEERRQQLLKSEEQEERRQQQLRREQQERREQRLK
REEEEERLEQRLKREHEEERREQELAEEEQEQARERIKSRIPKWQWQLESEADARQSKVY
SRPRKQEGQRRRQEQEEKRRRRESELQWQEEERAHRQQQEEEQRRDFTWQWQAEEKSERG
RQRLSARPPLREQRERQLRAEERQQREQRFLPEEEEKEQRRRQRREREKELQFLEEEEQL
QRRERAQQLQEEEDGLQEDQERRRSQEQRRDQKWRWQLEEERKRRRHTLYAKPALQEQLR
KEQQLLQEEEEELQREEREKRRRQEQERQYREEEQLQQEEEQLLREEREKRRRQERERQY
RKDKKLQQKEEQLLGEEPEKRRRQEREKKYREEEELQQEEEQLLREEREKRRRQEWERQY
RKKDELQQEEEQLLREEREKRRLQERERQYREEEELQQEEEQLLGEERETRRRQELERQY
RKEEELQQEEEQLLREEPEKRRRQERERQCREEEELQQEEEQLLREEREKRRRQELERQY
REEEEVQQEEEQLLREEPEKRRRQELERQYREEEELQQEEEQLLREEQEKRRQERERQYR
EEEELQRQKRKQRYRDEDQRSDLKWQWEPEKENAVRDNKVYCKGRENEQFRQLEDSQLRD
RQSQQDLQHLLGEQQERDREQERRRWQQRDRHFPEEEQLEREEQKEAKRRDRKSQEEKQL
LREEREEKRRRQETDRKFREEEQLLQEREEQPLRRQERDRKFREEELRHQEQGRKFLEEE
QRLRRQERERKFLKEEQQLRCQEREQQLRQDRDRKFREEEQQLSRQERDRKFREEEQQVR
RQERERKFLEEEQQLRQERHRKFREEEQLLQEREEQQLHRQERDRKFLEEEQQLRRQERD
RKFREQELRSQEPERKFLEEEQQLHRQQRQRKFLQEEQQLRRQERGQQRRQDRDRKFREE
EQLRQEREEQQLSRQERDRKFRLEEQKVRRQEQERKFMEDEQQLRRQEGQQQLRQERDRK
FREDEQLLQEREEQQLHRQERDRKFLEEEPQLRRQEREQQLRHDRDRKFREEEQLLQEGE

EQQLRRQERDRKFREEEQQLRRQERERKFLQEEQQLRRQELERKFREEEQLRQETEQEQL
RRQERYRKILEEEQLRPEREEQQLRRQERDRKFREEEQLRQEREEQQLRSQESDRKFREE
EQLRQEREEQQLRPQQRDGKYRWEEEQLQLEEQEQRLRQERDRQYRAEEQFATQEKSRRE
EQELWQEEEQKRRQERERKLREEHIRRQQKEEQRHRQVGEIKSQEGKGHGRLLEPGTHQF
ASVPVRSSPLYEYIQEQRSQYRP
>sp|Q5VVQ6|OTU1_HUMAN Ubiquitin thioesterase OTU1 OS=Homo sapiens OX=9606 GN=YOD1 PE=1 SV=1
MFGPAKGRHFGVHPAPGFPGGVSQQAAGTKAGPAGAWPVGSRTDTMWRLRCKAKDGTHVL
QGLSSRTRVRELQGQIAAITGIAPGGQRILVGYPPECLDLSNGDTILEDLPIQSGDMLII
EEDQTRPRSSPAFTKRGASSYVRETLPVLTRTVVPADNSCLFTSVYYVVEGGVLNPACAP
EMRRLIAQIVASDPDFYSEAILGKTNQEYCDWIKRDDTWGGAIEISILSKFYQCEICVVD
TQTVRIDRFGEDAGYTKRVLLIYDGIHYDPLQRNFPDPDTPPLTIFSSNDDIVLVQALEL
ADEARRRRQFTDVNRFTLRCMVCQKGLTGQAEAREHAKETGHTNFGEV
>sp|P28838|AMPL_HUMAN Cytosol aminopeptidase OS=Homo sapiens OX=9606 GN=LAP3 PE=1 SV=3
MFLLPLPAAGRVVVRRLAVRRFGSRSLSTADMTKGLVLGIYSKEKEDDVPQFTSAGENFD
KLLAGKLRETLNISGPPLKAGKTRTFYGLHQDFPSVVLVGLGKKAAGIDEQENWHEGKEN
IRAAVAAGCRQIQDLELSSVEVDPCGDAQAAAEGAVLGLYEYDDLKQKKKMAVSAKLYGS
GDQEAWQKGVLFASGQNLARQLMETPANEMTPTRFAEIIEKNLKSASSKTEVHIRPKSWI
EEQAMGSFLSVAKGSDEPPVFLEIHYKGSPNANEPPLVFVGKGITFDSGGISIKASANMD
LMRADMGGAATICSAIVSAAKLNLPINIIGLAPLCENMPSGKANKPGDVVRAKNGKTIQV
DNTDAEGRLILADALCYAHTFNPKVILNAATLTGAMDVALGSGATGVFTNSSWLWNKLFE
ASIETGDRVWRMPLFEHYTRQVVDCQLADVNNIGKYRSAGACTAAAFLKEFVTHPKWAHL
DIAGVMTNKDEVPYLRKGMTGRPTRTLIEFLLRFSQDNA
>sp|P32119|PRDX2_HUMAN Peroxiredoxin-2 OS=Homo sapiens OX=9606 GN=PRDX2 PE=1 SV=5
MASGNARIGKPAPDFKATAVVDGAFKEVKLSDYKGKYVVLFFYPLDFTFVCPTEIIAFSN
RAEDFRKLGCEVLGVSVDSQFTHLAWINTPRKEGGLGPLNIPLLADVTRRLSEDYGVLKT
DEGIAYRGLFIIDGKGVLRQITVNDLPVGRSVDEALRLVQAFQYTDEHGEVCPAGWKPGS
DTIKPNVDDSKEYFSKHN
>sp|P21333|FLNA_HUMAN Filamin-A OS=Homo sapiens OX=9606 GN=FLNA PE=1 SV=4
MSSSHSRAGQSAAGAAPGGGVDTRDAEMPATEKDLAEDAPWKKIQQNTFTRWCNEHLKCV
SKRIANLQTDLSDGLRLIALLEVLSQKKMHRKHNQRPTFRQMQLENVSVALEFLDRESIK
LVSIDSKAIVDGNLKLILGLIWTLILHYSISMPMWDEEEDEEAKKQTPKQRLLGWIQNKL
PQLPITNFSRDWQSGRALGALVDSCAPGLCPDWDSWDASKPVTNAREAMQQADDWLGIPQ
VITPEEIVDPNVDEHSVMTYLSQFPKAKLKPGAPLRPKLNPKKARAYGPGIEPTGNMVKK
RAEFTVETRSAGQGEVLVYVEDPAGHQEEAKVTANNDKNRTFSVWYVPEVTGTHKVTVLF
AGQHIAKSPFEVYVDKSQGDASKVTAQGPGLEPSGNIANKTTYFEIFTAGAGTGEVEVVI
QDPMGQKGTVEPQLEARGDSTYRCSYQPTMEGVHTVHVTFAGVPIPRSPYTVTVGQACNP
SACRAVGRGLQPKGVRVKETADFKVYTKGAGSGELKVTVKGPKGEERVKQKDLGDGVYGF
EYYPMVPGTYIVTITWGGQNIGRSPFEVKVGTECGNQKVRAWGPGLEGGVVGKSADFVVE
AIGDDVGTLGFSVEGPSQAKIECDDKGDGSCDVRYWPQEAGEYAVHVLCNSEDIRLSPFM
ADIRDAPQDFHPDRVKARGPGLEKTGVAVNKPAEFTVDAKHGGKAPLRVQVQDNEGCPVE
ALVKDNGNGTYSCSYVPRKPVKHTAMVSWGGVSIPNSPFRVNVGAGSHPNKVKVYGPGVA
KTGLKAHEPTYFTVDCAEAGQGDVSIGIKCAPGVVGPAEADIDFDIIRNDNDTFTVKYTP
RGAGSYTIMVLFADQATPTSPIRVKVEPSHDASKVKAEGPGLSRTGVELGKPTHFTVNAK

AAGKGKLDVQFSGLTKGDAVRDVDIIDHHDNTYTVKYTPVQQGPVGVNVTYGGDPIPKSP
FSVAVSPSLDLSKIKVSGLGEKVDVGKDQEFTVKSKGAGGQGKVASKIVGPSGAAVPCKV
EPGLGADNSVVRFLPREEGPYEVEVTYDGVPVPGSPFPLEAVAPTKPSKVKAFGPGLQGG
SAGSPARFTIDTKGAGTGGLGLTVEGPCEAQLECLDNGDGTCSVSYVPTEPGDYNINILF
ADTHIPGSPFKAHVVPCFDASKVKCSGPGLERATAGEVGQFQVDCSSAGSAELTIEICSE
AGLPAEVYIQDHGDGTHTITYIPLCPGAYTVTIKYGGQPVPNFPSKLQVEPAVDTSGVQC
YGPGIEGQGVFREATTEFSVDARALTQTGGPHVKARVANPSGNLTETYVQDRGDGMYKVE
YTPYEEGLHSVDVTYDGSPVPSSPFQVPVTEGCDPSRVRVHGPGIQSGTTNKPNKFTVET
RGAGTGGLGLAVEGPSEAKMSCMDNKDGSCSVEYIPYEAGTYSLNVTYGGHQVPGSPFKV
PVHDVTDASKVKCSGPGLSPGMVRANLPQSFQVDTSKAGVAPLQVKVQGPKGLVEPVDVV
DNADGTQTVNYVPSREGPYSISVLYGDEEVPRSPFKVKVLPTHDASKVKASGPGLNTTGV
PASLPVEFTIDAKDAGEGLLAVQITDPEGKPKKTHIQDNHDGTYTVAYVPDVTGRYTILI
KYGGDEIPFSPYRVRAVPTGDASKCTVTVSIGGHGLGAGIGPTIQIGEETVITVDTKAAG
KGKVTCTVCTPDGSEVDVDVVENEDGTFDIFYTAPQPGKYVICVRFGGEHVPNSPFQVTA
LAGDQPSVQPPLRSQQLAPQYTYAQGGQQTWAPERPLVGVNGLDVTSLRPFDLVIPFTIK
KGEITGEVRMPSGKVAQPTITDNKDGTVTVRYAPSEAGLHEMDIRYDNMHIPGSPLQFYV
DYVNCGHVTAYGPGLTHGVVNKPATFTVNTKDAGEGGLSLAIEGPSKAEISCTDNQDGTC
SVSYLPVLPGDYSILVKYNEQHVPGSPFTARVTGDDSMRMSHLKVGSAADIPINISETDL
SLLTATVVPPSGREEPCLLKRLRNGHVGISFVPKETGEHLVHVKKNGQHVASSPIPVVIS
QSEIGDASRVRVSGQGLHEGHTFEPAEFIIDTRDAGYGGLSLSIEGPSKVDINTEDLEDG
TCRVTYCPTEPGNYIINIKFADQHVPGSPFSVKVTGEGRVKESITRRRRAPSVANVGSHC
DLSLKIPEISIQDMTAQVTSPSGKTHEAEIVEGENHTYCIRFVPAEMGTHTVSVKYKGQH
VPGSPFQFTVGPLGEGGAHKVRAGGPGLERAEAGVPAEFSIWTREAGAGGLAIAVEGPSK
AEISFEDRKDGSCGVAYVVQEPGDYEVSVKFNEEHIPDSPFVVPVASPSGDARRLTVSSL
QESGLKVNQPASFAVSLNGAKGAIDAKVHSPSGALEECYVTEIDQDKYAVRFIPRENGVY
LIDVKFNGTHIPGSPFKIRVGEPGHGGDPGLVSAYGAGLEGGVTGNPAEFVVNTSNAGAG
ALSVTIDGPSKVKMDCQECPEGYRVTYTPMAPGSYLISIKYGGPYHIGGSPFKAKVTGPR
LVSNHSLHETSSVFVDSLTKATCAPQHGAPGPGPADASKVVAKGLGLSKAYVGQKSSFTV
DCSKAGNNMLLVGVHGPRTPCEEILVKHVGSRLYSVSYLLKDKGEYTLVVKWGDEHIPGS
PYRVVVP
>sp|Q14315|FLNC_HUMAN Filamin-C OS=Homo sapiens OX=9606 GN=FLNC PE=1 SV=3
MMNNSGYSDAGLGLGDETDEMPSTEKDLAEDAPWKKIQQNTFTRWCNEHLKCVGKRLTDL
QRDLSDGLRLIALLEVLSQKRMYRKFHPRPNFRQMKLENVSVALEFLEREHIKLVSIDSK
AIVDGNLKLILGLIWTLILHYSISMPMWEDEDDEDARKQTPKQRLLGWIQNKVPQLPITN
FNRDWQDGKALGALVDNCAPGLCPDWEAWDPNQPVENAREAMQQADDWLGVPQVIAPEEI
VDPNVDEHSVMTYLSQFPKAKLKPGAPVRSKQLNPKKAIAYGPGIEPQGNTVLQPAHFTV
QTVDAGVGEVLVYIEDPEGHTEEAKVVPNNDKDRTYAVSYVPKVAGLHKVTVLFAGQNIE
RSPFEVNVGMALGDANKVSARGPGLEPVGNVANKPTYFDIYTAGAGTGDVAVVIVDPQGR
RDTVEVALEDKGDSTFRCTYRPAMEGPHTVHVAFAGAPITRSPFPVHVSEACNPNACRAS
GRGLQPKGVRVKEVADFKVFTKGAGSGELKVTVKGPKGTEEPVKVREAGDGVFECEYYPV
VPGKYVVTITWGGYAIPRSPFEVQVSPEAGVQKVRAWGPGLETGQVGKSADFVVEAIGTE
VGTLGFSIEGPSQAKIECDDKGDGSCDVRYWPTEPGEYAVHVICDDEDIRDSPFIAHILP
APPDCFPDKVKAFGPGLEPTGCIVDKPAEFTIDARAAGKGDLKLYAQDADGCPIDIKVIP
NGDGTFRCSYVPTKPIKHTIIISWGGVNVPKSPFRVNVGEGSHPERVKVYGPGVEKTGLK
ANEPTYFTVDCSEAGQGDVSIGIKCAPGVVGPAEADIDFDIIKNDNDTFTVKYTPPGAGR
YTIMVLFANQEIPASPFHIKVDPSHDASKVKAEGPGLNRTGVEVGKPTHFTVLTKGAGKA

KLDVQFAGTAKGEVVRDFEIIDNHDYSYTVKYTAVQQGNMAVTVTYGGDPVPKSPFVVNV
APPLDLSKIKVQGLNSKVAVGQEQAFSVNTRGAGGQGQLDVRMTSPSRRPIPCKLEPGGG
AEAQAVRYMPPEEGPYKVDITYDGHPVPGSPFAVEGVLPPDPSKVCAYGPGLKGGLVGTP
APFSIDTKGAGTGGLGLTVEGPCEAKIECQDNGDGSCAVSYLPTEPGEYTINILFAEAHI
PGSPFKATIRPVFDPSKVRASGPGLERGKVGEAATFTVDCSEAGEAELTIEILSDAGVKA
EVLIHNNADGTYHITYSPAFPGTYTITIKYGGHPVPKFPTRVHVQPAVDTSGVKVSGPGV
EPHGVLREVTTEFTVDARSLTATGGNHVTARVLNPSGAKTDTYVTDNGDGTYRVQYTAYE
EGVHLVEVLYDEVAVPKSPFRVGVTEGCDPTRVRAFGPGLEGGLVNKANRFTVETRGAGT
GGLGLAIEGPSEAKMSCKDNKDGSCTVEYIPFTPGDYDVNITFGGRPIPGSPFRVPVKDV
VDPGKVKCSGPGLGAGVRARVPQTFTVDCSQAGRAPLQVAVLGPTGVAEPVEVRDNGDGT
HTVHYTPATDGPYTVAVKYADQEVPRSPFKIKVLPAHDASKVRASGPGLNASGIPASLPV
EFTIDARDAGEGLLTVQILDPEGKPKKANIRDNGDGTYTVSYLPDMSGRYTITIKYGGDE
IPYSPFRIHALPTGDASKCLVTVSIGGHGLGACLGPRIQIGQETVITVDAKAAGEGKVTC
TVSTPDGAELDVDVVENHDGTFDIYYTAPEPGKYVITIRFGGEHIPNSPFHVLACDPLPH
EEEPSEVPQLRQPYAPPRPGARPTHWATEEPVVPVEPMESMLRPFNLVIPFAVQKGELTG
EVRMPSGKTARPNITDNKDGTITVRYAPTEKGLHQMGIKYDGNHIPGSPLQFYVDAINSR
HVSAYGPGLSHGMVNKPATFTIVTKDAGEGGLSLAVEGPSKAEITCKDNKDGTCTVSYLP
TAPGDYSIIVRFDDKHIPGSPFTAKITGDDSMRTSQLNVGTSTDVSLKITESDLSQLTAS
IRAPSGNEEPCLLKRLPNRHIGISFTPKEVGEHVVSVRKSGKHVTNSPFKILVGPSEIGD
ASKVRVWGKGLSEGHTFQVAEFIVDTRNAGYGGLGLSIEGPSKVDINCEDMEDGTCKVTY
CPTEPGTYIINIKFADKHVPGSPFTVKVTGEGRMKESITRRRQAPSIATIGSTCDLNLKI
PGNWFQMVSAQERLTRTFTRSSHTYTRTERTEISKTRGGETKREVRVEESTQVGGDPFPA
VFGDFLGRERLGSFGSITRQQEGEASSQDMTAQVTSPSGKVEAAEIVEGEDSAYSVRFVP
QEMGPHTVAVKYRGQHVPGSPFQFTVGPLGEGGAHKVRAGGTGLERGVAGVPAEFSIWTR
EAGAGGLSIAVEGPSKAEIAFEDRKDGSCGVSYVVQEPGDYEVSIKFNDEHIPDSPFVVP
VASLSDDARRLTVTSLQETGLKVNQPASFAVQLNGARGVIDARVHTPSGAVEECYVSELD
SDKHTIRFIPHENGVHSIDVKFNGAHIPGSPFKIRVGEQSQAGDPGLVSAYGPGLEGGTT
GVSSEFIVNTLNAGSGALSVTIDGPSKVQLDCRECPEGHVVTYTPMAPGNYLIAIKYGGP
QHIVGSPFKAKVTGPRLSGGHSLHETSTVLVETVTKSSSSRGSSYSSIPKFSSDASKVVT
RGPGLSQAFVGQKNSFTVDCSKAGTNMMMVGVHGPKTPCEEVYVKHMGNRVYNVTYTVKE
KGDYILIVKWGDESVPGSPFKVKVP
>sp|P08238|HS90B_HUMAN Heat shock protein HSP 90-beta OS=Homo sapiens OX=9606 GN=HSP90AB1 PE=1 SV=4
MPEEVHHGEEEVETFAFQAEIAQLMSLIINTFYSNKEIFLRELISNASDALDKIRYESLT
DPSKLDSGKELKIDIIPNPQERTLTLVDTGIGMTKADLINNLGTIAKSGTKAFMEALQAG
ADISMIGQFGVGFYSAYLVAEKVVVITKHNDDEQYAWESSAGGSFTVRADHGEPIGRGTK
VILHLKEDQTEYLEERRVKEVVKKHSQFIGYPITLYLEKEREKEISDDEAEEEKGEKEEE
DKDDEEKPKIEDVGSDEEDDSGKDKKKKTKKIKEKYIDQEELNKTKPIWTRNPDDITQEE
YGEFYKSLTNDWEDHLAVKHFSVEGQLEFRALLFIPRRAPFDLFENKKKKNNIKLYVRRV
FIMDSCDELIPEYLNFIRGVVDSEDLPLNISREMLQQSKILKVIRKNIVKKCLELFSELA
EDKENYKKFYEAFSKNLKLGIHEDSTNRRRLSELLRYHTSQSGDEMTSLSEYVSRMKETQ
KSIYYITGESKEQVANSAFVERVRKRGFEVVYMTEPIDEYCVQQLKEFDGKSLVSVTKEG
LELPEDEEEKKKMEESKAKFENLCKLMKEILDKKVEKVTISNRLVSSPCCIVTSTYGWTA
NMERIMKAQALRDNSTMGYMMAKKHLEINPDHPIVETLRQKAEADKNDKAVKDLVVLLFE
TALLSSGFSLEDPQTHSNRIYRMIKLGLGIDEDEVAAEEPNAAVPDEIPPLEGDEDASRM
EEVD

>sp|Q58FF8|H90B2_HUMAN Putative heat shock protein HSP 90-beta 2 OS=Homo sapiens OX=9606 GN=HSP90AB2P PE=1 SV=2
MPEEVHLGEKEVETFAFQAEIAQLMSLIINTFYSNKEIFLWELISNASDALDKIRYESLT
DPSKLDSGKELKIDIIPNTQEHTLTLVDTGIGMTKADLINNLGTIAKFQDQTEYLEEMQV
KEVVEKHSQFLGYPITLYLEKEREKEISDGKAEEEKGEKEEENKDDEEKPKIEDVGSDEE
DDSGKDKKKKTKKIKEKYIDQEELNKTKPIWTRNTEDITQEEYGEFYKSLTNDWKDHLAV
RYFSVEEYVSRMKEIQKSIYYITGESKEQVANSAFVEQVWKRDSRVVYMTEPIDGYQLKE
FDGKSLVSVTKEGLELPEDGEEKKRMEERKAKFENLCKFMKETLDKKVEMVTVSNRLVSS
SCCIVTSTYSWTANMEQIMKA
>sp|Q58FF7|H90B3_HUMAN Putative heat shock protein HSP 90-beta-3 OS=Homo sapiens OX=9606 GN=HSP90AB3P PE=5 SV=1
MPEEVHHGEEEVETFAFQAEIAQLISLIINTFYSNEEIFLQELISNASDALDKIRYESLT
DPSKLDSGKELKIDIIPNPQERTLALVDTGIGMTKADLINNLRTIAKSGTKACMEALQAE
KLVVITKHNDDEQYAWESSAGGSFTVHADHGEPIGRGTKVILHLKEDQTEYLEERRVKEV
VKKHSQFIGYPITLYLEKEQDKEISDDEAEEEKGEKEEEDKDDEEKPKIKDVGSDEEDDS
KEYGEFYKSLTSDWEDHLAVKHFSVEGQLEFRALLFSPRRAPFDLFENKKKKNNIKLYVR
RVFIMDSCDELIPEYLNFIHGVVDSEDLPLNISREMLQQSKILKYVSHMKETQKSTYYIT
GESKEQVANSAFVERVRKQGFEVVYMTEPIDEYCVQQLKEFDGKSLVSVTKEGLELPEDE
EEKKKMEESKEKFENLCKLMKEILDKKVEKVTISNRLVSSPCCIVTSTYGWTANMEQIMK
AQALRDNSTMGYMMAKKHLEINPDHPIMETLRQKAEADKNDKAVKDLVVLLFETALLSSG
FSLEDPQTHSNHIYHMIKLGLGTDEDEVAAEEPSDAVPDEIPPLEGDEDASRMEEVD
>sp|P07737|PROF1_HUMAN Profilin-1 OS=Homo sapiens OX=9606 GN=PFN1 PE=1 SV=2
MAGWNAYIDNLMADGTCQDAAIVGYKDSPSVWAAVPGKTFVNITPAEVGVLVGKDRSSFY
VNGLTLGGQKCSVIRDSLLQDGEFSMDLRTKSTGGAPTFNVTVTKTDKTLVLLMGKEGVH
GGLINKKCYEMASHLRRSQY
>sp|P16152|CBR1_HUMAN Carbonyl reductase [NADPH] 1 OS=Homo sapiens OX=9606 GN=CBR1 PE=1 SV=3
MSSGIHVALVTGGNKGIGLAIVRDLCRLFSGDVVLTARDVTRGQAAVQQLQAEGLSPRFH
QLDIDDLQSIRALRDFLRKEYGGLDVLVNNAGIAFKVADPTPFHIQAEVTMKTNFFGTRD
VCTELLPLIKPQGRVVNVSSIMSVRALKSCSPELQQKFRSETITEEELVGLMNKFVEDTK
KGVHQKEGWPSSAYGVTKIGVTVLSRIHARKLSEQRKGDKILLNACCPGWVRTDMAGPKA
TKSPEEGAETPVYLALLPPDAEGPHGQFVSEKRVEQW
>sp|O75828|CBR3_HUMAN Carbonyl reductase [NADPH] 3 OS=Homo sapiens OX=9606 GN=CBR3 PE=1 SV=3
MSSCSRVALVTGANRGIGLAIARELCRQFSGDVVLTARDVARGQAAVQQLQAEGLSPRFH
QLDIDDLQSIRALRDFLRKEYGGLNVLVNNAAVAFKSDDPMPFDIKAEMTLKTNFFATRN
MCNELLPIMKPHGRVVNISSLQCLRAFENCSEDLQERFHSETLTEGDLVDLMKKFVEDTK
NEVHEREGWPNSPYGVSKLGVTVLSRILARRLDEKRKADRILVNACCPGPVKTDMDGKDS
IRTVEEGAETPVYLALLPPDATEPQGQLVHDKVVQNW
>sp|P0DOX7|IGK_HUMAN Immunoglobulin kappa light chain OS=Homo sapiens OX=9606 PE=1 SV=1
DIQMTQSPSTLSASVGDRVTITCRASQSINTWLAWYQQKPGKAPKLLMYKASSLESGVPS
RFIGSGSGTEFTLTISSLQPDDFATYYCQQYNSDSKMFGQGTKVEVKGTVAAPSVFIFPP
SDEQLKSGTASVVCLLNNFYPREAKVQWKVDNALQSGNSQESVTEQDSKDSTYSLSSTLT
LSKADYEKHKVYACEVTHQGLSSPVTKSFNRGEC

>sp|P01834|IGKC_HUMAN Immunoglobulin kappa constant OS=Homo sapiens OX=9606 GN=IGKC PE=1 SV=2
RTVAAPSVFIFPPSDEQLKSGTASVVCLLNNFYPREAKVQWKVDNALQSGNSQESVTEQD
SKDSTYSLSSTLTLSKADYEKHKVYACEVTHQGLSSPVTKSFNRGEC
>sp|P18669|PGAM1_HUMAN Phosphoglycerate mutase 1 OS=Homo sapiens OX=9606 GN=PGAM1 PE=1 SV=2
MAAYKLVLIRHGESAWNLENRFSGWYDADLSPAGHEEAKRGGQALRDAGYEFDICFTSVQ
KRAIRTLWTVLDAIDQMWLPVVRTWRLNERHYGGLTGLNKAETAAKHGEAQVKIWRRSYD
VPPPPMEPDHPFYSNISKDRRYADLTEDQLPSCESLKDTIARALPFWNEEIVPQIKEGKR
VLIAAHGNSLRGIVKHLEGLSEEAIMELNLPTGIPIVYELDKNLKPIKPMQFLGDEETVR
KAMEAVAAQGKAKK
>sp|P15259|PGAM2_HUMAN Phosphoglycerate mutase 2 OS=Homo sapiens OX=9606 GN=PGAM2 PE=1 SV=3
MATHRLVMVRHGESTWNQENRFCGWFDAELSEKGTEEAKRGAKAIKDAKMEFDICYTSVL
KRAIRTLWAILDGTDQMWLPVVRTWRLNERHYGGLTGLNKAETAAKHGEEQVKIWRRSFD
IPPPPMDEKHPYYNSISKERRYAGLKPGELPTCESLKDTIARALPFWNEEIVPQIKAGKR
VLIAAHGNSLRGIVKHLEGMSDQAIMELNLPTGIPIVYELNKELKPTKPMQFLGDEETVR
KAMEAVAAQGKAK
>sp|Q8N0Y7|PGAM4_HUMAN Probable phosphoglycerate mutase 4 OS=Homo sapiens OX=9606 GN=PGAM4 PE=3 SV=1
MAAYKLVLIRHGESTWNLENRFSCWYDADLSPAGHEEAKRGGQALRDAGYEFDICLTSVQ
KRVIRTLWTVLDAIDQMWLPVVRTWRLNERHYGGLTGLNKAETAAKHGEAQVKIWRRSYD
VPPPPMEPDHPFYSNISKDRRYADLTEDQLPSYESPKDTIARALPFWNEEIVPQIKEGKR
VLIAAHGNSLQGIAKHVEGLSEEAIMELNLPTGIPIVYELDKNLKPIKPMQFLGDEETVC
KAIEAVAAQGKAKK
>sp|P22314|UBA1_HUMAN Ubiquitin-like modifier-activating enzyme 1 OS=Homo sapiens OX=9606 GN=UBA1 PE=1 SV=3
MSSSPLSKKRRVSGPDPKPGSNCSPAQSVLSEVPSVPTNGMAKNGSEADIDEGLYSRQLY
VLGHEAMKRLQTSSVLVSGLRGLGVEIAKNIILGGVKAVTLHDQGTAQWADLSSQFYLRE
EDIGKNRAEVSQPRLAELNSYVPVTAYTGPLVEDFLSGFQVVVLTNTPLEDQLRVGEFCH
NRGIKLVVADTRGLFGQLFCDFGEEMILTDSNGEQPLSAMVSMVTKDNPGVVTCLDEARH
GFESGDFVSFSEVQGMVELNGNQPMEIKVLGPYTFSICDTSNFSDYIRGGIVSQVKVPKK
ISFKSLVASLAEPDFVVTDFAKFSRPAQLHIGFQALHQFCAQHGRPPRPRNEEDAAELVA
LAQAVNARALPAVQQNNLDEDLIRKLAYVAAGDLAPINAFIGGLAAQEVMKACSGKFMPI
MQWLYFDALECLPEDKEVLTEDKCLQRQNRYDGQVAVFGSDLQEKLGKQKYFLVGAGAIG
CELLKNFAMIGLGCGEGGEIIVTDMDTIEKSNLNRQFLFRPWDVTKLKSDTAAAAVRQMN
PHIRVTSHQNRVGPDTERIYDDDFFQNLDGVANALDNVDARMYMDRRCVYYRKPLLESGT
LGTKGNVQVVIPFLTESYSSSQDPPEKSIPICTLKNFPNAIEHTLQWARDEFEGLFKQPA
ENVNQYLTDPKFVERTLRLAGTQPLEVLEAVQRSLVLQRPQTWADCVTWACHHWHTQYSN
NIRQLLHNFPPDQLTSSGAPFWSGPKRCPHPLTFDVNNPLHLDYVMAAANLFAQTYGLTG
SQDRAAVATFLQSVQVPEFTPKSGVKIHVSDQELQSANASVDDSRLEELKATLPSPDKLP
GFKMYPIDFEKDDDSNFHMDFIVAASNLRAENYDIPSADRHKSKLIAGKIIPAIATTTAA
VVGLVCLELYKVVQGHRQLDSYKNGFLNLALPFFGFSEPLAAPRHQYYNQEWTLWDRFEV
QGLQPNGEEMTLKQFLDYFKTEHKLEITMLSQGVSMLYSFFMPAAKLKERLDQPMTEIVS
RVSKRKLGRHVRALVLELCCNDESGEDVEVPYVRYTIR
>sp|P05388|RLA0_HUMAN 60S acidic ribosomal protein P0 OS=Homo sapiens OX=9606 GN=RPLP0 PE=1 SV=1

MPREDRATWKSNYFLKIIQLLDDYPKCFIVGADNVGSKQMQQIRMSLRGKAVVLMGKNTM
MRKAIRGHLENNPALEKLLPHIRGNVGFVFTKEDLTEIRDMLLANKVPAAARAGAIAPCE
VTVPAQNTGLGPEKTSFFQALGITTKISRGTIEILSDVQLIKTGDKVGASEATLLNMLNI
SPFSFGLVIQQVFDNGSIYNPEVLDITEETLHSRFLEGVRNVASVCLQIGYPTVASVPHS
IINGYKRVLALSVETDYTFPLAEKVKAFLADPSAFVAAAPVAAATTAAPAAAAAPAKVEA
KEESEESDEDMGFGLFD
>sp|Q8NHW5|RLA0L_HUMAN 60S acidic ribosomal protein P0-like OS=Homo sapiens OX=9606 GN=RPLP0P6 PE=5 SV=1
MPREDRATWKSNYFLKIIQLLDDYPKCFIVGADNVGSKQMQQIRMSLRGKVVVLMGKNTM
MRKAIRGHLENNPALEKLLPHIWGNVGFVFTKEDLTEIRDMLLANKVPAAARAGAIAPCE
VTVPAQNTGLGPEKTSFFQALGITTKISRGTIEILSDVQLIKTGDKVGASEATLLNMLNI
SPFSFGLVIQQVFDNGSIYNPEVLDKTEETLHSRFLEGVRNVASVCLQTGYPTVASVPHS
IINGYKRVLALSVETDYTFPLAENVKAFLADPSAFVAAAPVAADTTAAPAAAAAPAKVEA
KEESEESDEDMGFGLFD
>sp|P15090|FABP4_HUMAN Fatty acid-binding protein, adipocyte OS=Homo sapiens OX=9606 GN=FABP4 PE=1 SV=3
MCDAFVGTWKLVSSENFDDYMKEVGVGFATRKVAGMAKPNMIISVNGDVITIKSESTFKN
TEISFILGQEFDEVTADDRKVKSTITLDGGVLVHVQKWDGKSTTIKRKREDDKLVVECVM
KGVTSTRVYERA
>sp|P02689|MYP2_HUMAN Myelin P2 protein OS=Homo sapiens OX=9606 GN=PMP2 PE=1 SV=3
MSNKFLGTWKLVSSENFDDYMKALGVGLATRKLGNLAKPTVIISKKGDIITIRTESTFKN
TEISFKLGQEFEETTADNRKTKSIVTLQRGSLNQVQRWDGKETTIKRKLVNGKMVAECKM
KGVVCTRIYEKV
>sp|P29401|TKT_HUMAN Transketolase OS=Homo sapiens OX=9606 GN=TKT PE=1 SV=3
MESYHKPDQQKLQALKDTANRLRISSIQATTAAGSGHPTSCCSAAEIMAVLFFHTMRYKS
QDPRNPHNDRFVLSKGHAAPILYAVWAEAGFLAEAELLNLRKISSDLDGHPVPKQAFTDV
ATGSLGQGLGAACGMAYTGKYFDKASYRVYCLLGDGELSEGSVWEAMAFASIYKLDNLVA
ILDINRLGQSDPAPLQHQMDIYQKRCEAFGWHAIIVDGHSVEELCKAFGQAKHQPTAIIA
KTFKGRGITGVEDKESWHGKPLPKNMAEQIIQEIYSQIQSKKKILATPPQEDAPSVDIAN
IRMPSLPSYKVGDKIATRKAYGQALAKLGHASDRIIALDGDTKNSTFSEIFKKEHPDRFI
ECYIAEQNMVSIAVGCATRNRTVPFCSTFAAFFTRAFDQIRMAAISESNINLCGSHCGVS
IGEDGPSQMALEDLAMFRSVPTSTVFYPSDGVATEKAVELAANTKGICFIRTSRPENAII
YNNNEDFQVGQAKVVLKSKDDQVTVIGAGVTLHEALAAAELLKKEKINIRVLDPFTIKPL
DRKLILDSARATKGRILTVEDHYYEGGIGEAVSSAVVGEPGITVTHLAVNRVPRSGKPAE
LLKMFGIDRDAIAQAVRGLITKA
>sp|P60842|IF4A1_HUMAN Eukaryotic initiation factor 4A-I OS=Homo sapiens OX=9606 GN=EIF4A1 PE=1 SV=1
MSASQDSRSRDNGPDGMEPEGVIESNWNEIVDSFDDMNLSESLLRGIYAYGFEKPSAIQQ
RAILPCIKGYDVIAQAQSGTGKTATFAISILQQIELDLKATQALVLAPTRELAQQIQKVV
MALGDYMGASCHACIGGTNVRAEVQKLQMEAPHIIVGTPGRVFDMLNRRYLSPKYIKMFV
LDEADEMLSRGFKDQIYDIFQKLNSNTQVVLLSATMPSDVLEVTKKFMRDPIRILVKKEE
LTLEGIRQFYINVEREEWKLDTLCDLYETLTITQAVIFINTRRKVDWLTEKMHARDFTVS
AMHGDMDQKERDVIMREFRSGSSRVLITTDLLARGIDVQQVSLVINYDLPTNRENYIHRI
GRGGRFGRKGVAINMVTEEDKRTLRDIETFYNTSIEEMPLNVADLI

>sp|Q14240|IF4A2_HUMAN Eukaryotic initiation factor 4A-II OS=Homo sapiens OX=9606 GN=EIF4A2 PE=1 SV=2
MSGGSADYNREHGGPEGMDPDGVIESNWNEIVDNFDDMNLKESLLRGIYAYGFEKPSAIQ
QRAIIPCIKGYDVIAQAQSGTGKTATFAISILQQLEIEFKETQALVLAPTRELAQQIQKV
ILALGDYMGATCHACIGGTNVRNEMQKLQAEAPHIVVGTPGRVFDMLNRRYLSPKWIKMF
VLDEADEMLSRGFKDQIYEIFQKLNTSIQVVLLSATMPTDVLEVTKKFMRDPIRILVKKE
ELTLEGIKQFYINVEREEWKLDTLCDLYETLTITQAVIFLNTRRKVDWLTEKMHARDFTV
SALHGDMDQKERDVIMREFRSGSSRVLITTDLLARGIDVQQVSLVINYDLPTNRENYIHR
IGRGGRFGRKGVAINFVTEEDKRILRDIETFYNTTVEEMPMNVADLI
>sp|P38919|IF4A3_HUMAN Eukaryotic initiation factor 4A-III OS=Homo sapiens OX=9606 GN=EIF4A3 PE=1 SV=4
MATTATMATSGSARKRLLKEEDMTKVEFETSEEVDVTPTFDTMGLREDLLRGIYAYGFEK
PSAIQQRAIKQIIKGRDVIAQSQSGTGKTATFSISVLQCLDIQVRETQALILAPTRELAV
QIQKGLLALGDYMNVQCHACIGGTNVGEDIRKLDYGQHVVAGTPGRVFDMIRRRSLRTRA
IKMLVLDEADEMLNKGFKEQIYDVYRYLPPATQVVLISATLPHEILEMTNKFMTDPIRIL
VKRDELTLEGIKQFFVAVEREEWKFDTLCDLYDTLTITQAVIFCNTKRKVDWLTEKMREA
NFTVSSMHGDMPQKERESIMKEFRSGASRVLISTDVWARGLDVPQVSLIINYDLPNNREL
YIHRIGRSGRYGRKGVAINFVKNDDIRILRDIEQYYSTQIDEMPMNVADLI
>sp|P01860|IGHG3_HUMAN Immunoglobulin heavy constant gamma 3 OS=Homo sapiens OX=9606 GN=IGHG3 PE=1 SV=2
ASTKGPSVFPLAPCSRSTSGGTAALGCLVKDYFPEPVTVSWNSGALTSGVHTFPAVLQSS
GLYSLSSVVTVPSSSLGTQTYTCNVNHKPSNTKVDKRVELKTPLGDTTHTCPRCPEPKSC
DTPPPCPRCPEPKSCDTPPPCPRCPEPKSCDTPPPCPRCPAPELLGGPSVFLFPPKPKDT
LMISRTPEVTCVVVDVSHEDPEVQFKWYVDGVEVHNAKTKPREEQYNSTFRVVSVLTVLH
QDWLNGKEYKCKVSNKALPAPIEKTISKTKGQPREPQVYTLPPSREEMTKNQVSLTCLVK
GFYPSDIAVEWESSGQPENNYNTTPPMLDSDGSFFLYSKLTVDKSRWQQGNIFSCSVMHE
ALHNRFTQKSLSLSPGK
>sp|P11216|PYGB_HUMAN Glycogen phosphorylase, brain form OS=Homo sapiens OX=9606 GN=PYGB PE=1 SV=5
MAKPLTDSEKRKQISVRGLAGLGDVAEVRKSFNRHLHFTLVKDRNVATPRDYFFALAHTV
RDHLVGRWIRTQQHYYERDPKRIYYLSLEFYMGRTLQNTMVNLGLQNACDEAIYQLGLDL
EELEEIEEDAGLGNGGLGRLAACFLDSMATLGLAAYGYGIRYEFGIFNQKIVNGWQVEEA
DDWLRYGNPWEKARPEYMLPVHFYGRVEHTPDGVKWLDTQVVLAMPYDTPVPGYKNNTVN
TMRLWSAKAPNDFKLQDFNVGDYIEAVLDRNLAENISRVLYPNDNFFEGKELRLKQEYFV
VAATLQDIIRRFKSSKFGCRDPVRTCFETFPDKVAIQLNDTHPALSIPELMRILVDVEKV
DWDKAWEITKKTCAYTNHTVLPEALERWPVSMFEKLLPRHLEIIYAINQRHLDHVAALFP
GDVDRLRRMSVIEEGDCKRINMAHLCVIGSHAVNGVARIHSEIVKQSVFKDFYELEPEKF
QNKTNGITPRRWLLLCNPGLADTIVEKIGEEFLTDLSQLKKLLPLVSDEVFIRDVAKVKQ
ENKLKFSAFLEKEYKVKINPSSMFDVHVKRIHEYKRQLLNCLHVVTLYNRIKRDPAKAFV
PRTVMIGGKAAPGYHMAKLIIKLVTSIGDVVNHDPVVGDRLKVIFLENYRVSLAEKVIPA
ADLSQQISTAGTEASGTGNMKFMLNGALTIGTMDGANVEMAEEAGAENLFIFGLRVEDVE
ALDRKGYNAREYYDHLPELKQAVDQISSGFFSPKEPDCFKDIVNMLMHHDRFKVFADYEA
YMQCQAQVDQLYRNPKEWTKKVIRNIACSGKFSSDRTITEYAREIWGVEPSDLQIPPPNI
PRD
>sp|P13489|RINI_HUMAN Ribonuclease inhibitor OS=Homo sapiens OX=9606 GN=RNH1 PE=1 SV=2
MSLDIQSLDIQCEELSDARWAELLPLLQQCQVVRLDDCGLTEARCKDISSALRVNPALAE

LNLRSNELGDVGVHCVLQGLQTPSCKIQKLSLQNCCLTGAGCGVLSSTLRTLPTLQELHL
SDNLLGDAGLQLLCEGLLDPQCRLEKLQLEYCSLSAASCEPLASVLRAKPDFKELTVSNN
DINEAGVRVLCQGLKDSPCQLEALKLESCGVTSDNCRDLCGIVASKASLRELALGSNKLG
DVGMAELCPGLLHPSSRLRTLWIWECGITAKGCGDLCRVLRAKESLKELSLAGNELGDEG
ARLLCETLLEPGCQLESLWVKSCSFTAACCSHFSSVLAQNRFLLELQISNNRLEDAGVRE
LCQGLGQPGSVLRVLWLADCDVSDSSCSSLAATLLANHSLRELDLSNNCLGDAGILQLVE
SVRQPGCLLEQLVLYDIYWSEEMEDRLQALEKDKPSLRVIS
>sp|Q6A163|K1C39_HUMAN Keratin, type I cytoskeletal 39 OS=Homo sapiens OX=9606 GN=KRT39 PE=1 SV=2
MDTKGCTTTNSPSTPCQNCSRITNVSTISSNNGCHPGGLTVNNCQPAGHVLRIPWDQGCQ
PTPRFCRKPIYLMNNFNARFSLDDCSWYGEGINSNEKETMQILNERLANYLQKVRMLERE
NAELESKIQEESNKELPVLCPDYLSYYTTIEELQQKILCTKAENSRLVSQIDNTKLTADD
LRAKYEAEVSLRQLVESDANGLKQILNVLTLGKADLEAQVQSLKEELLCLKNNHKEEINS
LQCQLGERLDIEVTAAPSADLNQVLQEMRCQYEPIMETNRKDVEQWFNTQIEELNQQVVT
SSQQQQCCQKEIIELRRSVNTLEVELQAQHRMRDSQECILTETEARYTALLTQIQSLIDN
LEAQLAEIRCALERQNQEYEILLDVKSRLECEITTYRSLLESSDGKRPCYPRATKCEPSP
WTSCKSGAIESTAPACTSSSPCSLKEHCSACGPLSRILVKICTITKEIKDGKVISSYEHV
QPCFIIRPAKV
>sp|P01859|IGHG2_HUMAN Immunoglobulin heavy constant gamma 2 OS=Homo sapiens OX=9606 GN=IGHG2 PE=1 SV=2
ASTKGPSVFPLAPCSRSTSESTAALGCLVKDYFPEPVTVSWNSGALTSGVHTFPAVLQSS
GLYSLSSVVTVPSSNFGTQTYTCNVDHKPSNTKVDKTVERKCCVECPPCPAPPVAGPSVF
LFPPKPKDTLMISRTPEVTCVVVDVSHEDPEVQFNWYVDGVEVHNAKTKPREEQFNSTFR
VVSVLTVVHQDWLNGKEYKCKVSNKGLPAPIEKTISKTKGQPREPQVYTLPPSREEMTKN
QVSLTCLVKGFYPSDISVEWESNGQPENNYKTTPPMLDSDGSFFLYSKLTVDKSRWQQGN
VFSCSVMHEALHNHYTQKSLSLSPGK
>sp|P01861|IGHG4_HUMAN Immunoglobulin heavy constant gamma 4 OS=Homo sapiens OX=9606 GN=IGHG4 PE=1 SV=1
ASTKGPSVFPLAPCSRSTSESTAALGCLVKDYFPEPVTVSWNSGALTSGVHTFPAVLQSS
GLYSLSSVVTVPSSSLGTKTYTCNVDHKPSNTKVDKRVESKYGPPCPSCPAPEFLGGPSV
FLFPPKPKDTLMISRTPEVTCVVVDVSQEDPEVQFNWYVDGVEVHNAKTKPREEQFNSTY
RVVSVLTVLHQDWLNGKEYKCKVSNKGLPSSIEKTISKAKGQPREPQVYTLPPSQEEMTK
NQVSLTCLVKGFYPSDIAVEWESNGQPENNYKTTPPVLDSDGSFFLYSRLTVDKSRWQEG
NVFSCSVMHEALHNHYTQKSLSLSLGK
>sp|O00391|QSOX1_HUMAN Sulfhydryl oxidase 1 OS=Homo sapiens OX=9606 GN=QSOX1 PE=1 SV=3
MRRCNSGSGPPPSLLLLLLWLLAVPGANAAPRSALYSPSDPLTLLQADTVRGAVLGSRSA
WAVEFFASWCGHCIAFAPTWKALAEDVKAWRPALYLAALDCAEETNSAVCRDFNIPGFPT
VRFFKAFTKNGSGAVFPVAGADVQTLRERLIDALESHHDTWPPACPPLEPAKLEEIDGFF
ARNNEEYLALIFEKGGSYLGREVALDLSQHKGVAVRRVLNTEANVVRKFGVTDFPSCYLL
FRNGSVSRVPVLMESRSFYTAYLQRLSGLTREAAQTTVAPTTANKIAPTVWKLADRSKIY
MADLESALHYILRIEVGRFPVLEGQRLVALKKFVAVLAKYFPGRPLVQNFLHSVNEWLKR
QKRNKIPYSFFKTALDDRKEGAVLAKKVNWIGCQGSEPHFRGFPCSLWVLFHFLTVQAAR
QNVDHSQEAAKAKEVLPAIRGYVHYFFGCRDCASHFEQMAAASMHRVGSPNAAVLWLWSS
HNRVNARLAGAPSEDPQFPKVQWPPRELCSACHNERLDVPVWDVEATLNFLKAHFSPSNI
ILDFPAAGSAARRDVQNVAAAPELAMGALELESRNSTLDPGKPEMMKSPTNTTPHVPAEG
PEASRPPKLHPGLRAAPGQEPPEHMAELQRNEQEQPLGQWHLSKRDTGAALLAESRAEKN

RLWGPLEVRRVGRSSKQLVDIPEGQLEARAGRGRGQWLQVLGGGFSYLDISLCVGLYSLS
FMGLLAMYTYFQAKIRALKGHAGHPAA
>sp|P62258|1433E_HUMAN 14-3-3 protein epsilon OS=Homo sapiens OX=9606 GN=YWHAE PE=1 SV=1
MDDREDLVYQAKLAEQAERYDEMVESMKKVAGMDVELTVEERNLLSVAYKNVIGARRASW
RIISSIEQKEENKGGEDKLKMIREYRQMVETELKLICCDILDVLDKHLIPAANTGESKVF
YYKMKGDYHRYLAEFATGNDRKEAAENSLVAYKAASDIAMTELPPTHPIRLGLALNFSVF
YYEILNSPDRACRLAKAAFDDAIAELDTLSEESYKDSTLIMQLLRDNLTLWTSDMQGDGE
EQNKEALQDVEDENQ
>sp|P13797|PLST_HUMAN Plastin-3 OS=Homo sapiens OX=9606 GN=PLS3 PE=1 SV=4
MDEMATTQISKDELDELKEAFAKVDLNSNGFICDYELHELFKEANMPLPGYKVREIIQKL
MLDGDRNKDGKISFDEFVYIFQEVKSSDIAKTFRKAINRKEGICALGGTSELSSEGTQHS
YSEEEKYAFVNWINKALENDPDCRHVIPMNPNTDDLFKAVGDGIVLCKMINLSVPDTIDE
RAINKKKLTPFIIQENLNLALNSASAIGCHVVNIGAEDLRAGKPHLVLGLLWQIIKIGLF
ADIELSRNEALAALLRDGETLEELMKLSPEELLLRWANFHLENSGWQKINNFSADIKDSK
AYFHLLNQIAPKGQKEGEPRIDINMSGFNETDDLKRAESMLQQADKLGCRQFVTPADVVS
GNPKLNLAFVANLFNKYPALTKPENQDIDWTLLEGETREERTFRNWMNSLGVNPHVNHLY
ADLQDALVILQLYERIKVPVDWSKVNKPPYPKLGANMKKLENCNYAVELGKHPAKFSLVG
IGGQDLNDGNQTLTLALVWQLMRRYTLNVLEDLGDGQKANDDIIVNWVNRTLSEAGKSTS
IQSFKDKTISSSLAVVDLIDAIQPGCINYDLVKSGNLTEDDKHNNAKYAVSMARRIGARV
YALPEDLVEVKPKMVMTVFACLMGRGMKRV
>sp|P13796|PLSL_HUMAN Plastin-2 OS=Homo sapiens OX=9606 GN=LCP1 PE=1 SV=6
MARGSVSDEEMMELREAFAKVDTDGNGYISFNELNDLFKAACLPLPGYRVREITENLMAT
GDLDQDGRISFDEFIKIFHGLKSTDVAKTFRKAINKKEGICAIGGTSEQSSVGTQHSYSE
EEKYAFVNWINKALENDPDCRHVIPMNPNTNDLFNAVGDGIVLCKMINLSVPDTIDERTI
NKKKLTPFTIQENLNLALNSASAIGCHVVNIGAEDLKEGKPYLVLGLLWQVIKIGLFADI
ELSRNEALIALLREGESLEDLMKLSPEELLLRWANYHLENAGCNKIGNFSTDIKDSKAYY
HLLEQVAPKGDEEGVPAVVIDMSGLREKDDIQRAECMLQQAERLGCRQFVTATDVVRGNP
KLNLAFIANLFNRYPALHKPENQDIDWGALEGETREERTFRNWMNSLGVNPRVNHLYSDL
SDALVIFQLYEKIKVPVDWNRVNKPPYPKLGGNMKKLENCNYAVELGKNQAKFSLVGIGG
QDLNEGNRTLTLALIWQLMRRYTLNILEEIGGGQKVNDDIIVNWVNETLREAKKSSSISS
FKDPKISTSLPVLDLIDAIQPGSINYDLLKTENLNDDEKLNNAKYAISMARKIGARVYAL
PEDLVEVNPKMVMTVFACLMGKGMKRV
>sp|Q14651|PLSI_HUMAN Plastin-1 OS=Homo sapiens OX=9606 GN=PLS1 PE=1 SV=2
MENSTTTISREELEELQEAFNKIDIDNSGYVSDYELQDLFKEASLPLPGYKVREIVEKIL
SVADSNKDGKISFEEFVSLMQELKSKDISKTFRKIINKREGITAIGGTSTISSEGTQHSY
SEEEKVAFVNWINKALENDPDCKHLIPMNPNDDSLFKSLADGILLCKMINLSEPDTIDER
AINKKKLTPFTISENLNLALNSASAIGCTVVNIGASDLKEGKPHLVLGLLWQIIKVGLFA
DIEISRNEALIALLNEGEELEELMKLSPEELLLRWVNYHLTNAGWHTISNFSQDIKDSRA
YFHLLNQIAPKGGEDGPAIAIDLSGINETNDLKRAGLMLQEADKLGCKQFVTPADVVSGN
PKLNLAFVANLFNTYPCLHKPNNNDIDMNLLEGESKEERTFRNWMNSLGVNPYINHLYSD
LADALVIFQLYEMIRVPVNWSHVNKPPYPALGGNMKKIENCNYAVELGKNKAKFSLVGIA
GQDLNEGNSTLTLALVWQLMRRYTLNVLSDLGEGEKVNDEIIIKWVNQTLKSANKKTSIS
SFKDKSISTSLPVLDLIDAIAPNAVRQEMIRRENLSDEDKLNNAKYAISVARKIGARIYA

LPDDLVEVKPKMVMTVFACLMGKGLNRIK
>sp|Q52LG2|KR132_HUMAN Keratin-associated protein 13-2 OS=Homo sapiens OX=9606 GN=KRTAP13-2 PE=1 SV=1
MSYNCCSGNFSSRSCGDYLRYPASSRGFSYPSNLVYSTDLCSPSTCQLGSSLYRGCQEIC
WEPTSCQTSYVESSPCQTSCYRPRTSLLCSPCKTTYSGSLGFGSSSCRSLGYGSRSCYSV
GCGSSGVRSLGYGSCGFPSLGYGSGFCRPTYLASRSCQSPCYRPAYGSTFCRSTC
>sp|P63244|RACK1_HUMAN Receptor of activated protein C kinase 1 OS=Homo sapiens OX=9606 GN=RACK1 PE=1 SV=3
MTEQMTLRGTLKGHNGWVTQIATTPQFPDMILSASRDKTIIMWKLTRDETNYGIPQRALR
GHSHFVSDVVISSDGQFALSGSWDGTLRLWDLTTGTTTRRFVGHTKDVLSVAFSSDNRQI
VSGSRDKTIKLWNTLGVCKYTVQDESHSEWVSCVRFSPNSSNPIIVSCGWDKLVKVWNLA
NCKLKTNHIGHTGYLNTVTVSPDGSLCASGGKDGQAMLWDLNEGKHLYTLDGGDIINALC
FSPNRYWLCAATGPSIKIWDLEGKIIVDELKQEVISTSSKAEPPQCTSLAWSADGQTLFA
GYTDNLVRVWQVTIGTR
>sp|Q15517|CDSN_HUMAN Corneodesmosin OS=Homo sapiens OX=9606 GN=CDSN PE=1 SV=3
MGSSRAPWMGRVGGHGMMALLLAGLLLPGTLAKSIGTFSDPCKDPTRITSPNDPCLTGKG
DSSGFSSYSGSSSSGSSISSARSSGGGSSGSSSGSSIAQGGSAGSFKPGTGYSQVSYSSG
SGSSLQGASGSSQLGSSSSHSGNSGSHSGSSSSHSSSSSSFQFSSSSFQVGNGSALPTND
NSYRGILNPSQPGQSSSSSQTSGVSSSGQSVSSNQRPCSSDIPDSPCSGGPIVSHSGPYI
PSSHSVSGGQRPVVVVVDQHGSGAPGVVQGPPCSNGGLPGKPCPPITSVDKSYGGYEVVG
GSSDSYLVPGMTYSKGKIYPVGYFTKENPVKGSPGVPSFAAGPPISEGKYFSSNPIIPSQ
SAASSAIAFQPVGTGGVQLCGGGSTGSKGPCSPSSSRVPSSSSISSSSGSPYHPCGSASQ
SPCSPPGTGSFSSSSSSQSSGKIILQPCGSKSSSSGHPCMSVSSLTLTGGPDGSPHPDPS
AGAKPCGSSSAGKIPCRSIRDILAQVKPLGPQLADPEVFLPQGELLDSP
>sp|P81605|DCD_HUMAN Dermcidin OS=Homo sapiens OX=9606 GN=DCD PE=1 SV=2
MRFMTLLFLTALAGALVCAYDPEAASAPGSGNPCHEASAAQKENAGEDPGLARQAPKPRK
QRSSLLEKGLDGAKKAVGGLGKLGKDAVEDLESVGKGAVHDVKDVLDSVL
>sp|O00571|DDX3X_HUMAN ATP-dependent RNA helicase DDX3X OS=Homo sapiens OX=9606 GN=DDX3X PE=1 SV=3
MSHVAVENALGLDQQFAGLDLNSSDNQSGGSTASKGRYIPPHLRNREATKGFYDKDSSGW
SSSKDKDAYSSFGSRSDSRGKSSFFSDRGSGSRGRFDDRGRSDYDGIGSRGDRSGFGKFE
RGGNSRWCDKSDEDDWSKPLPPSERLEQELFSGGNTGINFEKYDDIPVEATGNNCPPHIE
SFSDVEMGEIIMGNIELTRYTRPTPVQKHAIPIIKEKRDLMACAQTGSGKTAAFLLPILS
QIYSDGPGEALRAMKENGRYGRRKQYPISLVLAPTRELAVQIYEEARKFSYRSRVRPCVV
YGGADIGQQIRDLERGCHLLVATPGRLVDMMERGKIGLDFCKYLVLDEADRMLDMGFEPQ
IRRIVEQDTMPPKGVRHTMMFSATFPKEIQMLARDFLDEYIFLAVGRVGSTSENITQKVV
WVEESDKRSFLLDLLNATGKDSLTLVFVETKKGADSLEDFLYHEGYACTSIHGDRSQRDR
EEALHQFRSGKSPILVATAVAARGLDISNVKHVINFDLPSDIEEYVHRIGRTGRVGNLGL
ATSFFNERNINITKDLLDLLVEAKQEVPSWLENMAYEHHYKGSSRGRSKSSRFSGGFGAR
DYRQSSGASSSSFSSSRASSSRSGGGGHGSSRGFGGGGYGGFYNSDGYGGNYNSQGVDWW
GN
>sp|O15523|DDX3Y_HUMAN ATP-dependent RNA helicase DDX3Y OS=Homo sapiens OX=9606 GN=DDX3Y PE=1 SV=2
MSHVVVKNDPELDQQLANLDLNSEKQSGGASTASKGRYIPPHLRNREASKGFHDKDSSGW
SCSKDKDAYSSFGSRDSRGKPGYFSERGSGSRGRFDDRGRSDYDGIGNRERPGFGRFERS

GHSRWCDKSVEDDWSKPLPPSERLEQELFSGGNTGINFEKYDDIPVEATGSNCPPHIENF
SDIDMGEIIMGNIELTRYTRPTPVQKHAIPIIKGKRDLMACAQTGSGKTAAFLLPILSQI
YTDGPGEALKAVKENGRYGRRKQYPISLVLAPTRELAVQIYEEARKFSYRSRVRPCVVYG
GADIGQQIRDLERGCHLLVATPGRLVDMMERGKIGLDFCKYLVLDEADRMLDMGFEPQIR
RIVEQDTMPPKGVRHTMMFSATFPKEIQMLARDFLDEYIFLAVGRVGSTSENITQKVVWV
EDLDKRSFLLDILGATGSDSLTLVFVETKKGADSLEDFLYHEGYACTSIHGDRSQRDREE
ALHQFRSGKSPILVATAVAARGLDISNVRHVINFDLPSDIEEYVHRIGRTGRVGNLGLAT
SFFNEKNMNITKDLLDLLVEAKQEVPSWLENMAYEHHYKGGSRGRSKSNRFSGGFGARDY
RQSSGSSSSGFGASRGSSSRSGGGGYGNSRGFGGGGYGGFYNSDGYGGNYNSQGVDWWGN
>sp|P60201|MYPR_HUMAN Myelin proteolipid protein OS=Homo sapiens OX=9606 GN=PLP1 PE=1 SV=2
MGLLECCARCLVGAPFASLVATGLCFFGVALFCGCGHEALTGTEKLIETYFSKNYQDYEY
LINVIHAFQYVIYGTASFFFLYGALLLAEGFYTTGAVRQIFGDYKTTICGKGLSATVTGG
QKGRGSRGQHQAHSLERVCHCLGKWLGHPDKFVGITYALTVVWLLVFACSAVPVYIYFNT
WTTCQSIAFPSKTSASIGSLCADARMYGVLPWNAFPGKVCGSNLLSICKTAEFQMTFHLF
IAAFVGAAATLVSLLTFMIAATYNFAVLKLMGRGTKF
>sp|P51178|PLCD1_HUMAN 1-phosphatidylinositol 4,5-bisphosphate phosphodiesterase delta-1 OS=Homo sapiens OX=9606 GN=PLCD1 PE=1 SV=2
MDSGRDFLTLHGLQDDEDLQALLKGSQLLKVKSSSWRRERFYKLQEDCKTIWQESRKVMR
TPESQLFSIEDIQEVRMGHRTEGLEKFARDVPEDRCFSIVFKDQRNTLDLIAPSPADAQH
WVLGLHKIIHHSGSMDQRQKLQHWIHSCLRKADKNKDNKMSFKELQNFLKELNIQVDDSY
ARKIFRECDHSQTDSLEDEEIEAFYKMLTQRVEIDRTFAEAAGSGETLSVDQLVTFLQHQ
QREEAAGPALALSLIERYEPSETAKAQRQMTKDGFLMYLLSADGSAFSLAHRRVYQDMGQ
PLSHYLVSSSHNTYLLEDQLAGPSSTEAYIRALCKGCRCLELDCWDGPNQEPIIYHGYTF
TSKILFCDVLRAIRDYAFKASPYPVILSLENHCTLEQQRVMARHLHAILGPMLLNRPLDG
VTNSLPSPEQLKGKILLKGKKLGGLLPPGGEGGPEATVVSDEDEAAEMEDEAVRSRVQHK
PKEDKLRLAQELSDMVIYCKSVHFGGFSSPGTPGQAFYEMASFSENRALRLLQESGNGFV
RHNVGHLSRIYPAGWRTDSSNYSPVEMWNGGCQIVALNFQTPGPEMDVYQGRFQDNGACG
YVLKPAFLRDPNGTFNPRALAQGPWWARKRLNIRVISGQQLPKVNKNKNSIVDPKVTVEI
HGVSRDVASRQTAVITNNGFNPWWDTEFAFEVVVPDLALIRFLVEDYDASSKNDFIGQST
IPLNSLKQGYRHVHLMSKNGDQHPSATLFVKISLQD
>sp|Q06830|PRDX1_HUMAN Peroxiredoxin-1 OS=Homo sapiens OX=9606 GN=PRDX1 PE=1 SV=1
MSSGNAKIGHPAPNFKATAVMPDGQFKDISLSDYKGKYVVFFFYPLDFTFVCPTEIIAFS
DRAEEFKKLNCQVIGASVDSHFCHLAWVNTPKKQGGLGPMNIPLVSDPKRTIAQDYGVLK
ADEGISFRGLFIIDDKGILRQITVNDLPVGRSVDETLRLVQAFQFTDKHGEVCPAGWKPG
SDTIKPDVQKSKEYFSKQK
>sp|Q13162|PRDX4_HUMAN Peroxiredoxin-4 OS=Homo sapiens OX=9606 GN=PRDX4 PE=1 SV=1
MEALPLLAATTPDHGRHRRLLLLPLLLFLLPAGAVQGWETEERPRTREEECHFYAGGQVY
PGEASRVSVADHSLHLSKAKISKPAPYWEGTAVIDGEFKELKLTDYRGKYLVFFFYPLDF
TFVCPTEIIAFGDRLEEFRSINTEVVACSVDSQFTHLAWINTPRRQGGLGPIRIPLLSDL
THQISKDYGVYLEDSGHTLRGLFIIDDKGILRQITLNDLPVGRSVDETLRLVQAFQYTDK
HGEVCPAGWKPGSETIIPDPAGKLKYFDKLN
>sp|Q8IUC0|KR131_HUMAN Keratin-associated protein 13-1 OS=Homo sapiens OX=9606 GN=KRTAP13-1 PE=2 SV=2

MSYNCCSGNFSSRSCGGYLHYPASSCGFSYPSNQVYSTDLCSPSTCQLGSSLYRGCQQTC
WEPTSCQTSYVESSPCQTSCYRPRTSLLCSPCQTTYSGSLGFGSSSCRSLGYGSRSCYSV
GCGSSGFRSLGYGGCGFPSLGYGVGFCRPTYLASRSCQSSCYRPTCGSGFYY
>sp|P67936|TPM4_HUMAN Tropomyosin alpha-4 chain OS=Homo sapiens OX=9606 GN=TPM4 PE=1 SV=3
MAGLNSLEAVKRKIQALQQQADEAEDRAQGLQRELDGERERREKAEGDVAALNRRIQLVE
EELDRAQERLATALQKLEEAEKAADESERGMKVIENRAMKDEEKMEIQEMQLKEAKHIAE
EADRKYEEVARKLVILEGELERAEERAEVSELKCGDLEEELKNVTNNLKSLEAASEKYSE
KEDKYEEEIKLLSDKLKEAETRAEFAERTVAKLEKTIDDLEEKLAQAKEENVGLHQTLDQ
TLNELNCI
>sp|P07951|TPM2_HUMAN Tropomyosin beta chain OS=Homo sapiens OX=9606 GN=TPM2 PE=1 SV=1
MDAIKKKMQMLKLDKENAIDRAEQAEADKKQAEDRCKQLEEEQQALQKKLKGTEDEVEKY
SESVKEAQEKLEQAEKKATDAEADVASLNRRIQLVEEELDRAQERLATALQKLEEAEKAA
DESERGMKVIENRAMKDEEKMELQEMQLKEAKHIAEDSDRKYEEVARKLVILEGELERSE
ERAEVAESKCGDLEEELKIVTNNLKSLEAQADKYSTKEDKYEEEIKLLEEKLKEAETRAE
FAERSVAKLEKTIDDLEDEVYAQKMKYKAISEELDNALNDITSL
>sp|P01040|CYTA_HUMAN Cystatin-A OS=Homo sapiens OX=9606 GN=CSTA PE=1 SV=1
MIPGGLSEAKPATPEIQEIVDKVKPQLEEKTNETYGKLEAVQYKTQVVAGTNYYIKVRAG
DNKYMHLKVFKSLPGQNEDLVLTGYQVDKNKDDELTGF
>sp|P22528|SPR1B_HUMAN Cornifin-B OS=Homo sapiens OX=9606 GN=SPRR1B PE=1 SV=2
MSSQQQKQPCTPPPQLQQQQVKQPCQPPPQEPCIPKTKEPCHPKVPEPCHPKVPEPCQPK
VPEPCHPKVPEPCPSIVTPAPAQQKTKQK
>sp|O75635|SPB7_HUMAN Serpin B7 OS=Homo sapiens OX=9606 GN=SERPINB7 PE=1 SV=1
MASLAAANAEFCFNLFREMDDNQGNGNVFFSSLSLFAALALVRLGAQDDSLSQIDKLLHV
NTASGYGNSSNSQSGLQSQLKRVFSDINASHKDYDLSIVNGLFAEKVYGFHKDYIECAEK
LYDAKVERVDFTNHLEDTRRNINKWVENETHGKIKNVIGEGGISSSAVMVLVNAVYFKGK
WQSAFTKSETINCHFKSPKCSGKAVAMMHQERKFNLSVIEDPSMKILELRYNGGINMYVL
LPENDLSEIENKLTFQNLMEWTNPRRMTSKYVEVFFPQFKIEKNYEMKQYLRALGLKDIF
DESKADLSGIASGGRLYISRMMHKSYIEVTEEGTEATAATGSNIVEKQLPQSTLFRADHP
FLFVIRKDDIILFSGKVSCP
>sp|P23528|COF1_HUMAN Cofilin-1 OS=Homo sapiens OX=9606 GN=CFL1 PE=1 SV=3
MASGVAVSDGVIKVFNDMKVRKSSTPEEVKKRKKAVLFCLSEDKKNIILEEGKEILVGDV
GQTVDDPYATFVKMLPDKDCRYALYDATYETKESKKEDLVFIFWAPESAPLKSKMIYASS
KDAIKKKLTGIKHELQANCYEEVKDRCTLAEKLGGSAVISLEGKPL
>sp|Q9Y281|COF2_HUMAN Cofilin-2 OS=Homo sapiens OX=9606 GN=CFL2 PE=1 SV=1
MASGVTVNDEVIKVFNDMKVRKSSTQEEIKKRKKAVLFCLSDDKRQIIVEEAKQILVGDI
GDTVEDPYTSFVKLLPLNDCRYALYDATYETKESKKEDLVFIFWAPESAPLKSKMIYASS
KDAIKKKFTGIKHEWQVNGLDDIKDRSTLGEKLGGNVVVSLEGKPL
>sp|P19971|TYPH_HUMAN Thymidine phosphorylase OS=Homo sapiens OX=9606 GN=TYMP PE=1 SV=2
MAALMTPGTGAPPAPGDFSGEGSQGLPDPSPEPKQLPELIRMKRDGGRLSEADIRGFVAA

VVNGSAQGAQIGAMLMAIRLRGMDLEETSVLTQALAQSGQQLEWPEAWRQQLVDKHSTGG
VGDKVSLVLAPALAACGCKVPMISGRGLGHTGGTLDKLESIPGFNVIQSPEQMQVLLDQA
GCCIVGQSEQLVPADGILYAARDVTATVDSLPLITASILSKKLVEGLSALVVDVKFGGAA
VFPNQEQARELAKTLVGVGASLGLRVAAALTAMDKPLGRCVGHALEVEEALLCMDGAGPP
DLRDLVTTLGGALLWLSGHAGTQAQGAARVAAALDDGSALGRFERMLAAQGVDPGLARAL
CSGSPAERRQLLPRAREQEELLAPADGTVELVRALPLALVLHELGAGRSRAGEPLRLGVG
AELLVDVGQRLRRGTPWLRVHRDGPALSGPQSRALQEALVLSDRAPFAAPSPFAELVLPP
QQ
>sp|Q7Z3Y7|K1C28_HUMAN Keratin, type I cytoskeletal 28 OS=Homo sapiens OX=9606 GN=KRT28 PE=1 SV=2
MSLQFSNGSRHVCLRSGAGSVRPLNGGAGFAGSSACGGSVAGSEFSCALGGGLGSVPGGS
HAGGALGNAACIGFAGSEGGLLSGNEKVTMQNLNDRLASYLDNVRALEEANAELERKIKG
WYEKYGPGSCRGLDHDYSRYHLTIEDLKNKIISSTTTNANVILQIDNARLAADDFRLKYE
NELTLHQNVEADINGLRRVLDELTLCRTDQELQYESLSEEMTYLKKNHEEEMKALQCAAG
GNVNVEMNAAPGVDLAVLLNNMRAEYEALAEQNRKDAEAWFNEKSASLQQQISHDSGAAT
FARSQLTEMRRTLQTLEIQLQSLMATKHSLECSLTETESNYCTQLAQIQAQIGALEEQLH
QVRTETEGQKLEYEHLLDVKVHLEKEIETYCRLIDGDGNSCSKSKGFGSGSPGNSSKDLS
KTTLVKTVVEELDQRGKVLSSRIHSIEEKTSKMTNGKTEQRVPF
>sp|P35321|SPR1A_HUMAN Cornifin-A OS=Homo sapiens OX=9606 GN=SPRR1A PE=1 SV=2
MNSQQQKQPCTPPPQPQQQQVKQPCQPPPQEPCIPKTKEPCHPKVPEPCHPKVPEPCQPK
VPEPCQPKVPEPCPSTVTPAPAQQKTKQK
>sp|P17931|LEG3_HUMAN Galectin-3 OS=Homo sapiens OX=9606 GN=LGALS3 PE=1 SV=5
MADNFSLHDALSGSGNPNPQGWPGAWGNQPAGAGGYPGASYPGAYPGQAPPGAYPGQAPP
GAYPGAPGAYPGAPAPGVYPGPPSGPGAYPSSGQPSATGAYPATGPYGAPAGPLIVPYNL
PLPGGVVPRMLITILGTVKPNANRIALDFQRGNDVAFHFNPRFNENNRRVIVCNTKLDNN
WGREERQSVFPFESGKPFKIQVLVEPDHFKVAVNDAHLLQYNHRVKKLNEISKLGISGDI
DLTSASYTMI
>sp|P02671|FIBA_HUMAN Fibrinogen alpha chain OS=Homo sapiens OX=9606 GN=FGA PE=1 SV=2
MFSMRIVCLVLSVVGTAWTADSGEGDFLAEGGGVRGPRVVERHQSACKDSDWPFCSDEDW
NYKCPSGCRMKGLIDEVNQDFTNRINKLKNSLFEYQKNNKDSHSLTTNIMEILRGDFSSA
NNRDNTYNRVSEDLRSRIEVLKRKVIEKVQHIQLLQKNVRAQLVDMKRLEVDIDIKIRSC
RGSCSRALAREVDLKDYEDQQKQLEQVIAKDLLPSRDRQHLPLIKMKPVPDLVPGNFKSQ
LQKVPPEWKALTDMPQMRMELERPGGNEITRGGSTSYGTGSETESPRNPSSAGSWNSGSS
GPGSTGNRNPGSSGTGGTATWKPGSSGPGSTGSWNSGSSGTGSTGNQNPGSPRPGSTGTW
NPGSSERGSAGHWTSESSVSGSTGQWHSESGSFRPDSPGSGNARPNNPDWGTFEEVSGNV
SPGTRREYHTEKLVTSKGDKELRTGKEKVTSGSTTTTRRSCSKTVTKTVIGPDGHKEVTK
EVVTSEDGSDCPEAMDLGTLSGIGTLDGFRHRHPDEAAFFDTASTGKTFPGFFSPMLGEF
VSETESRGSESGIFTNTKESSSHHPGIAEFPSRGKSSSYSKQFTSSTSYNRGDSTFESKS
YKMADEAGSEADHEGTHSTKRGHAKSRPVRDCDDVLQTHPSGTQSGIFNIKLPGSSKIFS
VYCDQETSLGGWLLIQQRMDGSLNFNRTWQDYKRGFGSLNDEGEGEFWLGNDYLHLLTQR
GSVLRVELEDWAGNEAYAEYHFRVGSEAEGYALQVSSYEGTAGDALIEGSVEEGAEYTSH
NNMQFSTFDRDADQWEENCAEVYGGGWWYNNCQAANLNGIYYPGGSYDPRNNSPYEIENG
VVWVSFRGADYSLRAVRMKIRPLVTQ

>sp|P01023|A2MG_HUMAN Alpha-2-macroglobulin OS=Homo sapiens OX=9606 GN=A2M PE=1 SV=3
MGKNKLLHPSLVLLLLVLLPTDASVSGKPQYMVLVPSLLHTETTEKGCVLLSYLNETVTV
SASLESVRGNRSLFTDLEAENDVLHCVAFAVPKSSSNEEVMFLTVQVKGPTQEFKKRTTV
MVKNEDSLVFVQTDKSIYKPGQTVKFRVVSMDENFHPLNELIPLVYIQDPKGNRIAQWQS
FQLEGGLKQFSFPLSSEPFQGSYKVVVQKKSGGRTEHPFTVEEFVLPKFEVQVTVPKIIT
ILEEEMNVSVCGLYTYGKPVPGHVTVSICRKYSDASDCHGEDSQAFCEKFSGQLNSHGCF
YQQVKTKVFQLKRKEYEMKLHTEAQIQEEGTVVELTGRQSSEITRTITKLSFVKVDSHFR
QGIPFFGQVRLVDGKGVPIPNKVIFIRGNEANYYSNATTDEHGLVQFSINTTNVMGTSLT
VRVNYKDRSPCYGYQWVSEEHEEAHHTAYLVFSPSKSFVHLEPMSHELPCGHTQTVQAHY
ILNGGTLLGLKKLSFYYLIMAKGGIVRTGTHGLLVKQEDMKGHFSISIPVKSDIAPVARL
LIYAVLPTGDVIGDSAKYDVENCLANKVDLSFSPSQSLPASHAHLRVTAAPQSVCALRAV
DQSVLLMKPDAELSASSVYNLLPEKDLTGFPGPLNDQDNEDCINRHNVYINGITYTPVSS
TNEKDMYSFLEDMGLKAFTNSKIRKPKMCPQLQQYEMHGPEGLRVGFYESDVMGRGHARL
VHVEEPHTETVRKYFPETWIWDLVVVNSAGVAEVGVTVPDTITEWKAGAFCLSEDAGLGI
SSTASLRAFQPFFVELTMPYSVIRGEAFTLKATVLNYLPKCIRVSVQLEASPAFLAVPVE
KEQAPHCICANGRQTVSWAVTPKSLGNVNFTVSAEALESQELCGTEVPSVPEHGRKDTVI
KPLLVEPEGLEKETTFNSLLCPSGGEVSEELSLKLPPNVVEESARASVSVLGDILGSAMQ
NTQNLLQMPYGCGEQNMVLFAPNIYVLDYLNETQQLTPEIKSKAIGYLNTGYQRQLNYKH
YDGSYSTFGERYGRNQGNTWLTAFVLKTFAQARAYIFIDEAHITQALIWLSQRQKDNGCF
RSSGSLLNNAIKGGVEDEVTLSAYITIALLEIPLTVTHPVVRNALFCLESAWKTAQEGDH
GSHVYTKALLAYAFALAGNQDKRKEVLKSLNEEAVKKDNSVHWERPQKPKAPVGHFYEPQ
APSAEVEMTSYVLLAYLTAQPAPTSEDLTSATNIVKWITKQQNAQGGFSSTQDTVVALHA
LSKYGAATFTRTGKAAQVTIQSSGTFSSKFQVDNNNRLLLQQVSLPELPGEYSMKVTGEG
CVYLQTSLKYNILPEKEEFPFALGVQTLPQTCDEPKAHTSFQISLSVSYTGSRSASNMAI
VDVKMVSGFIPLKPTVKMLERSNHVSRTEVSSNHVLIYLDKVSNQTLSLFFTVLQDVPVR
DLKPAIVKVYDYYETDEFAIAEYNAPCSKDLGNA
>sp|P20742|PZP_HUMAN Pregnancy zone protein OS=Homo sapiens OX=9606 GN=PZP PE=1 SV=4
MRKDRLLHLCLVLLLILLSASDSNSTEPQYMVLVPSLLHTEAPKKGCVLLSHLNETVTVS
ASLESGRENRSLFTDLVAEKDLFHCVSFTLPRISASSEVAFLSIQIKGPTQDFRKRNTVL
VLNTQSLVFVQTDKPMYKPGQTVRFRVVSVDENFRPRNELIPLIYLENPRRNRIAQWQSL
KLEAGINQLSFPLSSEPIQGSYRVVVQTESGGRIQHPFTVEEFVLPKFEVKVQVPKIISI
MDEKVNITVCGEYTYGKPVPGLATVSLCRKLSRVLNCDKQEVCEEFSQQLNSNGCITQQV
HTKMLQITNTGFEMKLRVEARIREEGTDLEVTANRISEITNIVSKLKFVKVDSHFRQGIP
FFAQVLLVDGKGVPIPNKLFFISVNDANYYSNATTNEQGLAQFSINTTSISVNKLFVRVF
TVHPNLCFHYSWVAEDHQGAQHTANRVFSLSGSYIHLEPVAGTLPCGHTETITAHYTLNR
QAMGELSELSFHYLIMAKGVIVRSGTHTLPVESGDMKGSFALSFPVESDVAPIARMFIFA
ILPDGEVVGDSEKFEIENCLANKVDLSFSPAQSPPASHAHLQVAAAPQSLCALRAVDQSV
LLMKPEAELSVSSVYNLLTVKDLTNFPDNVDQQEEEQGHCPRPFFIHNGAIYVPLSSNEA
DIYSFLKGMGLKVFTNSKIRKPKSCSVIPSVSAGAVGQGYYGAGLGVVERPYVPQLGTYN
VIPLNNEQSSGPVPETVRSYFPETWIWELVAVNSSGVAEVGVTVPDTITEWKAGAFCLSE
DAGLGISSTASLRAFQPFFVELTMPYSVIRGEVFTLKATVLNYLPKCIRVSVQLKASPAF
LASQNTKGEESYCICGNERQTLSWTVTPKTLGNVNFSVSAEAMQSLELCGNEVVEVPEIK
RKDTVIKTLLVEAEGIEQEKTFSSMTCASGANVSEQLSLKLPSNVVKESARASFSVLGDI
LGSAMQNIQNLLQMPYGCGEQNMVLFAPNIYVLNYLNETQQLTQEIKAKAVGYLITGYQR
QLNYKHQDGSYSTFGERYGRNQGNTWLTAFVLKTFAQARSYIFIDEAHITQSLTWLSQMQ

KDNGCFRSSGSLLNNAIKGGVEDEATLSAYVTIALLEIPLPVTNPIVRNALFCLESAWNV
AKEGTHGSHVYTKALLAYAFSLLGKQNQNREILNSLDKEAVKEDNLVHWERPQRPKAPVG
HLYQTQAPSAEVEMTSYVLLAYLTAQPAPTSGDLTSATNIVKWIMKQQNAQGGFSSTQDT
VVALHALSRYGAATFTRTEKTAQVTVQDSQTFSTNFQVDNNNLLLLQQISLPELPGEYVI
TVTGERCVYLQTSMKYNILPEKEDSPFALKVQTVPQTCDGHKAHTSFQISLTISYTGNRP
ASNMVIVDVKMVSGFIPLKPTVKMLERSSSVSRTEVSNNHVLIYVEQVTNQTLSFSFMVL
QDIPVGDLKPAIVKVYDYYETDESVVAEYIAPCSTDTEHGNV
>sp|P40121|CAPG_HUMAN Macrophage-capping protein OS=Homo sapiens OX=9606 GN=CAPG PE=1 SV=2
MYTAIPQSGSPFPGSVQDPGLHVWRVEKLKPVPVAQENQGVFFSGDSYLVLHNGPEEVSH
LHLWIGQQSSRDEQGACAVLAVHLNTLLGERPVQHREVQGNESDLFMSYFPRGLKYQEGG
VESAFHKTSTGAPAAIKKLYQVKGKKNIRATERALNWDSFNTGDCFILDLGQNIFAWCGG
KSNILERNKARDLALAIRDSERQGKAQVEIVTDGEEPAEMIQVLGPKPALKEGNPEEDLT
ADKANAQAAALYKVSDATGQMNLTKVADSSPFALELLISDDCFVLDNGLCGKIYIWKGRK
ANEKERQAALQVAEGFISRMQYAPNTQVEILPQGHESPIFKQFFKDWK
>sp|Q92820|GGH_HUMAN Gamma-glutamyl hydrolase OS=Homo sapiens OX=9606 GN=GGH PE=1 SV=2
MASPGCLLCVLGLLLCGAASLELSRPHGDTAKKPIIGILMQKCRNKVMKNYGRYYIAASY
VKYLESAGARVVPVRLDLTEKDYEILFKSINGILFPGGSVDLRRSDYAKVAKIFYNLSIQ
SFDDGDYFPVWGTCLGFEELSLLISGECLLTATDTVDVAMPLNFTGGQLHSRMFQNFPTE
LLLSLAVEPLTANFHKWSLSVKNFTMNEKLKKFFNVLTTNTDGKIEFISTMEGYKYPVYG
VQWHPEKAPYEWKNLDGISHAPNAVKTAFYLAEFFVNEARKNNHHFKSESEEEKALIYQF
SPIYTGNISSFQQCYIFD
>sp|P62805|H4_HUMAN Histone H4 OS=Homo sapiens OX=9606 GN=H4-16 PE=1 SV=2
MSGRGKGGKGLGKGGAKRHRKVLRDNIQGITKPAIRRLARRGGVKRISGLIYEETRGVLK
VFLENVIRDAVTYTEHAKRKTVTAMDVVYALKRQGRTLYGFGG
>sp|P07339|CATD_HUMAN Cathepsin D OS=Homo sapiens OX=9606 GN=CTSD PE=1 SV=1
MQPSSLLPLALCLLAAPASALVRIPLHKFTSIRRTMSEVGGSVEDLIAKGPVSKYSQAVP
AVTEGPIPEVLKNYMDAQYYGEIGIGTPPQCFTVVFDTGSSNLWVPSIHCKLLDIACWIH
HKYNSDKSSTYVKNGTSFDIHYGSGSLSGYLSQDTVSVPCQSASSASALGGVKVERQVFG
EATKQPGITFIAAKFDGILGMAYPRISVNNVLPVFDNLMQQKLVDQNIFSFYLSRDPDAQ
PGGELMLGGTDSKYYKGSLSYLNVTRKAYWQVHLDQVEVASGLTLCKEGCEAIVDTGTSL
MVGPVDEVRELQKAIGAVPLIQGEYMIPCEKVSTLPAITLKLGGKGYKLSPEDYTLKVSQ
AGKTLCLSGFMGMDIPPPSGPLWILGDVFIGRYYTVFDRDNNRVGFAEAARL
>sp|P12273|PIP_HUMAN Prolactin-inducible protein OS=Homo sapiens OX=9606 GN=PIP PE=1 SV=1
MRLLQLLFRASPATLLLVLCLQLGANKAQDNTRKIIIKNFDIPKSVRPNDEVTAVLAVQT
ELKECMVVKTYLISSIPLQGAFNYKYTACLCDDNPKTFYWDFYTNRTVQIAAVVDVIREL
GICPDDAAVIPIKNNRFYTIEILKVE
>sp|Q562R1|ACTBL_HUMAN Beta-actin-like protein 2 OS=Homo sapiens OX=9606 GN=ACTBL2 PE=1 SV=2
MTDNELSALVVDNGSGMCKAGFGGDDAPRAVFPSMIGRPRHQGVMVGMGQKDCYVGDEAQ
SKRGVLTLKYPIEHGVVTNWDDMEKIWYHTFYNELRVAPDEHPILLTEAPLNPKINREKM
TQIMFEAFNTPAMYVAIQAVLSLYASGRTTGIVMDSGDGVTHIVPIYEGYALPHAILRLD
LAGRDLTDYLMKILTERGYNFTTTAEREIVRDVKEKLCYVALDFEQEMVRAAASSSPERS

YELPDGQVITIGNERFRCPEAIFQPSFLGIESSGIHETTFNSIMKCDVDIRKDLYANTVL
SGGSTMYPGIADRMQKEIITLAPSTMKIKIIAPPERKYSVWIGGSILASLSTFQQMWISK
QEYDEAGPPIVHRKCF
>sp|P01876|IGHA1_HUMAN Immunoglobulin heavy constant alpha 1 OS=Homo sapiens OX=9606 GN=IGHA1 PE=1 SV=2
ASPTSPKVFPLSLCSTQPDGNVVIACLVQGFFPQEPLSVTWSESGQGVTARNFPPSQDAS
GDLYTTSSQLTLPATQCLAGKSVTCHVKHYTNPSQDVTVPCPVPSTPPTPSPSTPPTPSP
SCCHPRLSLHRPALEDLLLGSEANLTCTLTGLRDASGVTFTWTPSSGKSAVQGPPERDLC
GCYSVSSVLPGCAEPWNHGKTFTCTAAYPESKTPLTATLSKSGNTFRPEVHLLPPPSEEL
ALNELVTLTCLARGFSPKDVLVRWLQGSQELPREKYLTWASRQEPSQGTTTFAVTSILRV
AAEDWKKGDTFSCMVGHEALPLAFTQKTIDRLAGKPTHVNVSVVMAEVDGTCY
>sp|P01877|IGHA2_HUMAN Immunoglobulin heavy constant alpha 2 OS=Homo sapiens OX=9606 GN=IGHA2 PE=1 SV=4
ASPTSPKVFPLSLDSTPQDGNVVVACLVQGFFPQEPLSVTWSESGQNVTARNFPPSQDAS
GDLYTTSSQLTLPATQCPDGKSVTCHVKHYTNSSQDVTVPCRVPPPPPCCHPRLSLHRPA
LEDLLLGSEANLTCTLTGLRDASGATFTWTPSSGKSAVQGPPERDLCGCYSVSSVLPGCA
QPWNHGETFTCTAAHPELKTPLTANITKSGNTFRPEVHLLPPPSEELALNELVTLTCLAR
GFSPKDVLVRWLQGSQELPREKYLTWASRQEPSQGTTTYAVTSILRVAAEDWKKGETFSC
MVGHEALPLAFTQKTIDRMAGKPTHINVSVVMAEADGTCY
>sp|P0DOX2|IGA2_HUMAN Immunoglobulin alpha-2 heavy chain OS=Homo sapiens OX=9606 PE=1 SV=2
EVQLVETGGGLIQPGGSLRLSCAASGFTVSNHSMSWVRQAPGKALEWVSAIYRGGTTYYA
DSVKGRFTISRDDSRNTVYLQMNSLRAEDTAVYYCARDLAAARLFGKGTTVTVSSASPTS
PKVFPLSLDSTPQDGNVVVACLVQGFFPQEPLSVTWSESGQNVTARNFPPSQDASGDLYT
TSSQLTLPATQCPDGKSVTCHVKHYTNSSQDVTVPCRVPPPPPCCHPRLSLHRPALEDLL
LGSEANLTCTLTGLRDASGATFTWTPSSGKSAVEGPPERDLCGCYSVSSVLPGCAQPWNH
GETFTCTAAHPELKTPLTANITKSGNTFRPEVHLLPPPSEELALNELVTLTCLARGFSPK
DVLVRWLQGSQELPREKYLTWASRQEPSQGTTTYAVTSILRVAAEDWKKGETFSCMVGHE
ALPLAFTQKTIDRLAGKPTHINVSVVMAEADGTCY
>sp|P58876|H2B1D_HUMAN Histone H2B type 1-D OS=Homo sapiens OX=9606 GN=H2BC5 PE=1 SV=2
MPEPTKSAPAPKKGSKKAVTKAQKKDGKKRKRSRKESYSVYVYKVLKQVHPDTGISSKAM
GIMNSFVNDIFERIAGEASRLAHYNKRSTITSREIQTAVRLLLPGELAKHAVSEGTKAVT
KYTSSK
>sp|Q5QNW6|H2B2F_HUMAN Histone H2B type 2-F OS=Homo sapiens OX=9606 GN=H2BC18 PE=1 SV=3
MPDPAKSAPAPKKGSKKAVTKVQKKDGKKRKRSRKESYSVYVYKVLKQVHPDTGISSKAM
GIMNSFVNDIFERIAGEASRLAHYNKRSTITSREIQTAVRLLLPGELAKHAVSEGTKAVT
KYTSSK
>sp|Q93079|H2B1H_HUMAN Histone H2B type 1-H OS=Homo sapiens OX=9606 GN=H2BC9 PE=1 SV=3
MPDPAKSAPAPKKGSKKAVTKAQKKDGKKRKRSRKESYSVYVYKVLKQVHPDTGISSKAM
GIMNSFVNDIFERIAGEASRLAHYNKRSTITSREIQTAVRLLLPGELAKHAVSEGTKAVT
KYTSSK
>sp|Q99880|H2B1L_HUMAN Histone H2B type 1-L OS=Homo sapiens OX=9606 GN=H2BC13 PE=1 SV=3
MPELAKSAPAPKKGSKKAVTKAQKKDGKKRKRSRKESYSVYVYKVLKQVHPDTGISSKAM

GIMNSFVNDIFERIASEASRLAHYNKRSTITSREIQTAVRLLLPGELAKHAVSEGTKAVT
KYTSSK
>sp|P57053|H2BFS_HUMAN Histone H2B type F-S OS=Homo sapiens OX=9606 GN=H2BC12L PE=1 SV=2
MPEPAKSAPAPKKGSKKAVTKAQKKDGRKRKRSRKESYSVYVYKVLKQVHPDTGISSKAM
GIMNSFVNDIFERIAGEASRLPHYNKRSTITSREIQTAVRLLLPGELAKHAVSEGTKAVT
KYTSAK
>sp|P62807|H2B1C_HUMAN Histone H2B type 1-C/E/F/G/I OS=Homo sapiens OX=9606 GN=H2BC10 PE=1 SV=4
MPEPAKSAPAPKKGSKKAVTKAQKKDGKKRKRSRKESYSVYVYKVLKQVHPDTGISSKAM
GIMNSFVNDIFERIAGEASRLAHYNKRSTITSREIQTAVRLLLPGELAKHAVSEGTKAVT
KYTSSK
>sp|O60814|H2B1K_HUMAN Histone H2B type 1-K OS=Homo sapiens OX=9606 GN=H2BC12 PE=1 SV=3
MPEPAKSAPAPKKGSKKAVTKAQKKDGKKRKRSRKESYSVYVYKVLKQVHPDTGISSKAM
GIMNSFVNDIFERIAGEASRLAHYNKRSTITSREIQTAVRLLLPGELAKHAVSEGTKAVT
KYTSAK
>sp|Q99877|H2B1N_HUMAN Histone H2B type 1-N OS=Homo sapiens OX=9606 GN=H2BC15 PE=1 SV=3
MPEPSKSAPAPKKGSKKAVTKAQKKDGKKRKRSRKESYSVYVYKVLKQVHPDTGISSKAM
GIMNSFVNDIFERIAGEASRLAHYNKRSTITSREIQTAVRLLLPGELAKHAVSEGTKAVT
KYTSSK
>sp|Q99879|H2B1M_HUMAN Histone H2B type 1-M OS=Homo sapiens OX=9606 GN=H2BC14 PE=1 SV=3
MPEPVKSAPVPKKGSKKAINKAQKKDGKKRKRSRKESYSVYVYKVLKQVHPDTGISSKAM
GIMNSFVNDIFERIAGEASRLAHYNKRSTITSREIQTAVRLLLPGELAKHAVSEGTKAVT
KYTSSK
>sp|Q02878|RL6_HUMAN 60S ribosomal protein L6 OS=Homo sapiens OX=9606 GN=RPL6 PE=1 SV=3
MAGEKVEKPDTKEKKPEAKKVDAGGKVKKGNLKAKKPKKGKPHCSRNPVLVRGIGRYSRS
AMYSRKAMYKRKYSAAKSKVEKKKKEKVLATVTKPVGGDKNGGTRVVKLRKMPRYYPTED
VPRKLLSHGKKPFSQHVRKLRASITPGTILIILTGRHRGKRVVFLKQLASGLLLVTGPLV
LNRVPLRRTHQKFVIATSTKIDISNVKIPKHLTDAYFKKKKLRKPRHQEGEIFDTEKEKY
EITEQRKIDQKAVDSQILPKIKAIPQLQGYLRSVFALTNGIYPHKLVF
>sp|P12277|KCRB_HUMAN Creatine kinase B-type OS=Homo sapiens OX=9606 GN=CKB PE=1 SV=1
MPFSNSHNALKLRFPAEDEFPDLSAHNNHMAKVLTPELYAELRAKSTPSGFTLDDVIQTG
VDNPGHPYIMTVGCVAGDEESYEVFKDLFDPIIEDRHGGYKPSDEHKTDLNPDNLQGGDD
LDPNYVLSSRVRTGRSIRGFCLPPHCSRGERRAIEKLAVEALSSLDGDLAGRYYALKSMT
EAEQQQLIDDHFLFDKPVSPLLLASGMARDWPDARGIWHNDNKTFLVWVNEEDHLRVISM
QKGGNMKEVFTRFCTGLTQIETLFKSKDYEFMWNPHLGYILTCPSNLGTGLRAGVHIKLP
NLGKHEKFSEVLKRLRLQKRGTGGVDTAAVGGVFDVSNADRLGFSEVELVQMVVDGVKLL
IEMEQRLEQGQAIDDLMPAQK
>sp|P52907|CAZA1_HUMAN F-actin-capping protein subunit alpha-1 OS=Homo sapiens OX=9606 GN=CAPZA1 PE=1 SV=3
MADFDDRVSDEEKVRIAAKFITHAPPGEFNEVFNDVRLLLNNDNLLREGAAHAFAQYNMD
QFTPVKIEGYEDQVLITEHGDLGNSRFLDPRNKISFKFDHLRKEASDPQPEEADGGLKSW

RESCDSALRAYVKDHYSNGFCTVYAKTIDGQQTIIACIESHQFQPKNFWNGRWRSEWKFT
ITPPTAQVVGVLKIQVHYYEDGNVQLVSHKDVQDSLTVSNEAQTAKEFIKIIENAENEYQ
TAISENYQTMSDTTFKALRRQLPVTRTKIDWNKILSYKIGKEMQNA
>sp|Q53RT3|APRV1_HUMAN Retroviral-like aspartic protease 1 OS=Homo sapiens OX=9606 GN=ASPRV1 PE=1 SV=1
MGSPGASLGIKKALQSEQATALPASAPAVSQPTAPAPSCLPKAGQVIPTLLREAPFSSVI
APTLLCGFLFLAWVAAEVPEESSRMAGSGARSEEGRRQHAFVPEPFDGANVVPNLWLHSF
EVINDLNHWDHITKLRFLKESLRGEALGVYNRLSPQDQGDYGTVKEALLKAFGVPGAAPS
HLPKEIVFANSMGKGYYLKGKIGKVPVRFLVDSGAQVSVVHPNLWEEVTDGDLDTLQPFE
NVVKVANGAEMKILGVWDTAVSLGKLKLKAQFLVANASAEEAIIGTDVLQDHNAILDFEH
RTCTLKGKKFRLLPVGGSLEDEFDLELIEEDPSSEEGRQELSH
>sp|Q9NZH8|IL36G_HUMAN Interleukin-36 gamma OS=Homo sapiens OX=9606 GN=IL36G PE=1 SV=1
MRGTPGDADGGGRAVYQSMCKPITGTINDLNQQVWTLQGQNLVAVPRSDSVTPVTVAVIT
CKYPEALEQGRGDPIYLGIQNPEMCLYCEKVGEQPTLQLKEQKIMDLYGQPEPVKPFLFY
RAKTGRTSTLESVAFPDWFIASSKRDQPIILTSELGKSYNTAFELNIND
>sp|Q07065|CKAP4_HUMAN Cytoskeleton-associated protein 4 OS=Homo sapiens OX=9606 GN=CKAP4 PE=1 SV=2
MPSAKQRGSKGGHGAASPSEKGAHPSGGADDVAKKPPPAPQQPPPPPAPHPQQHPQQHPQ
NQAHGKGGHRGGGGGGGKSSSSSSASAAAAAAAASSSASCSRRLGRALNFLFYLALVAAA
AFSGWCVHHVLEEVQQVRRSHQDFSRQREELGQGLQGVEQKVQSLQATFGTFESILRSSQ
HKQDLTEKAVKQGESEVSRISEVLQKLQNEILKDLSDGIHVVKDARERDFTSLENTVEER
LTELTKSINDNIAIFTEVQKRSQKEINDMKAKVASLEESEGNKQDLKALKEAVKEIQTSA
KSREWDMEALRSTLQTMESDIYTEVRELVSLKQEQQAFKEAADTERLALQALTEKLLRSE
ESVSRLPEEIRRLEEELRQLKSDSHGPKEDGGFRHSEAFEALQQKSQGLDSRLQHVEDGV
LSMQVASARQTESLESLLSKSQEHEQRLAALQGRLEGLGSSEADQDGLASTVRSLGETQL
VLYGDVEELKRSVGELPSTVESLQKVQEQVHTLLSQDQAQAARLPPQDFLDRLSSLDNLK
ASVSQVEADLKMLRTAVDSLVAYSVKIETNENNLESAKGLLDDLRNDLDRLFVKVEKIHE
KV
>sp|P31946|1433B_HUMAN 14-3-3 protein beta/alpha OS=Homo sapiens OX=9606 GN=YWHAB PE=1 SV=3
MTMDKSELVQKAKLAEQAERYDDMAAAMKAVTEQGHELSNEERNLLSVAYKNVVGARRSS
WRVISSIEQKTERNEKKQQMGKEYREKIEAELQDICNDVLELLDKYLIPNATQPESKVFY
LKMKGDYFRYLSEVASGDNKQTTVSNSQQAYQEAFEISKKEMQPTHPIRLGLALNFSVFY
YEILNSPEKACSLAKTAFDEAIAELDTLNEESYKDSTLIMQLLRDNLTLWTSENQGDEGD
AGEGEN
>sp|P35749|MYH11_HUMAN Myosin-11 OS=Homo sapiens OX=9606 GN=MYH11 PE=1 SV=3
MAQKGQLSDDEKFLFVDKNFINSPVAQADWAAKRLVWVPSEKQGFEAASIKEEKGDEVVV
ELVENGKKVTVGKDDIQKMNPPKFSKVEDMAELTCLNEASVLHNLRERYFSGLIYTYSGL
FCVVVNPYKHLPIYSEKIVDMYKGKKRHEMPPHIYAIADTAYRSMLQDREDQSILCTGES
GAGKTENTKKVIQYLAVVASSHKGKKDTSITGELEKQLLQANPILEAFGNAKTVKNDNSS
RFGKFIRINFDVTGYIVGANIETYLLEKSRAIRQARDERTFHIFYYMIAGAKEKMRSDLL
LEGFNNYTFLSNGFVPIPAAQDDEMFQETVEAMAIMGFSEEEQLSILKVVSSVLQLGNIV
FKKERNTDQASMPDNTAAQKVCHLMGINVTDFTRSILTPRIKVGRDVVQKAQTKEQADFA
VEALAKATYERLFRWILTRVNKALDKTHRQGASFLGILDIAGFEIFEVNSFEQLCINYTN
EKLQQLFNHTMFILEQEEYQREGIEWNFIDFGLDLQPCIELIERPNNPPGVLALLDEECW

FPKATDKSFVEKLCTEQGSHPKFQKPKQLKDKTEFSIIHYAGKVDYNASAWLTKNMDPLN
DNVTSLLNASSDKFVADLWKDVDRIVGLDQMAKMTESSLPSASKTKKGMFRTVGQLYKEQ
LGKLMTTLRNTTPNFVRCIIPNHEKRSGKLDAFLVLEQLRCNGVLEGIRICRQGFPNRIV
FQEFRQRYEILAANAIPKGFMDGKQACILMIKALELDPNLYRIGQSKIFFRTGVLAHLEE
ERDLKITDVIMAFQAMCRGYLARKAFAKRQQQLTAMKVIQRNCAAYLKLRNWQWWRLFTK
VKPLLQVTRQEEEMQAKEDELQKTKERQQKAENELKELEQKHSQLTEEKNLLQEQLQAET
ELYAEAEEMRVRLAAKKQELEEILHEMEARLEEEEDRGQQLQAERKKMAQQMLDLEEQLE
EEEAARQKLQLEKVTAEAKIKKLEDEILVMDDQNNKLSKERKLLEERISDLTTNLAEEEE
KAKNLTKLKNKHESMISELEVRLKKEEKSRQELEKLKRKLEGDASDFHEQIADLQAQIAE
LKMQLAKKEEELQAALARLDDEIAQKNNALKKIRELEGHISDLQEDLDSERAARNKAEKQ
KRDLGEELEALKTELEDTLDSTATQQELRAKREQEVTVLKKALDEETRSHEAQVQEMRQK
HAQAVEELTEQLEQFKRAKANLDKNKQTLEKENADLAGELRVLGQAKQEVEHKKKKLEAQ
VQELQSKCSDGERARAELNDKVHKLQNEVESVTGMLNEAEGKAIKLAKDVASLSSQLQDT
QELLQEETRQKLNVSTKLRQLEEERNSLQDQLDEEMEAKQNLERHISTLNIQLSDSKKKL
QDFASTVEALEEGKKRFQKEIENLTQQYEEKAAAYDKLEKTKNRLQQELDDLVVDLDNQR
QLVSNLEKKQRKFDQLLAEEKNISSKYADERDRAEAEAREKETKALSLARALEEALEAKE
ELERTNKMLKAEMEDLVSSKDDVGKNVHELEKSKRALETQMEEMKTQLEELEDELQATED
AKLRLEVNMQALKGQFERDLQARDEQNEEKRRQLQRQLHEYETELEDERKQRALAAAAKK
KLEGDLKDLELQADSAIKGREEAIKQLRKLQAQMKDFQRELEDARASRDEIFATAKENEK
KAKSLEADLMQLQEDLAAAERARKQADLEKEELAEELASSLSGRNALQDEKRRLEARIAQ
LEEELEEEQGNMEAMSDRVRKATQQAEQLSNELATERSTAQKNESARQQLERQNKELRSK
LHEMEGAVKSKFKSTIAALEAKIAQLEEQVEQEAREKQAATKSLKQKDKKLKEILLQVED
ERKMAEQYKEQAEKGNARVKQLKRQLEEAEEESQRINANRRKLQRELDEATESNEAMGRE
VNALKSKLRRGNETSFVPSRRSGGRRVIENADGSEEETDTRDADFNGTKASE
>sp|P31949|S10AB_HUMAN Protein S100-A11 OS=Homo sapiens OX=9606 GN=S100A11 PE=1 SV=2
MAKISSPTETERCIESLIAVFQKYAGKDGYNYTLSKTEFLSFMNTELAAFTKNQKDPGVL
DRMMKKLDTNSDGQLDFSEFLNLIGGLAMACHDSFLKAVPSQKRT
>sp|P40926|MDHM_HUMAN Malate dehydrogenase, mitochondrial OS=Homo sapiens OX=9606 GN=MDH2 PE=1 SV=3
MLSALARPASAALRRSFSTSAQNNAKVAVLGASGGIGQPLSLLLKNSPLVSRLTLYDIAH
TPGVAADLSHIETKAAVKGYLGPEQLPDCLKGCDVVVIPAGVPRKPGMTRDDLFNTNATI
VATLTAACAQHCPEAMICVIANPVNSTIPITAEVFKKHGVYNPNKIFGVTTLDIVRANTF
VAELKGLDPARVNVPVIGGHAGKTIIPLISQCTPKVDFPQDQLTALTGRIQEAGTEVVKA
KAGAGSATLSMAYAGARFVFSLVDAMNGKEGVVECSFVKSQETECTYFSTPLLLGKKGIE
KNLGIGKVSSFEEKMISDAIPELKASIKKGEDFVKTLK
>sp|P06753|TPM3_HUMAN Tropomyosin alpha-3 chain OS=Homo sapiens OX=9606 GN=TPM3 PE=1 SV=2
MMEAIKKKMQMLKLDKENALDRAEQAEAEQKQAEERSKQLEDELAAMQKKLKGTEDELDK
YSEALKDAQEKLELAEKKAADAEAEVASLNRRIQLVEEELDRAQERLATALQKLEEAEKA
ADESERGMKVIENRALKDEEKMELQEIQLKEAKHIAEEADRKYEEVARKLVIIEGDLERT
EERAELAESKCSELEEELKNVTNNLKSLEAQAEKYSQKEDKYEEEIKILTDKLKEAETRA
EFAERSVAKLEKTIDDLEDELYAQKLKYKAISEELDHALNDMTSI
>sp|P23527|H2B1O_HUMAN Histone H2B type 1-O OS=Homo sapiens OX=9606 GN=H2BC17 PE=1 SV=3
MPDPAKSAPAPKKGSKKAVTKAQKKDGKKRKRSRKESYSIYVYKVLKQVHPDTGISSKAM
GIMNSFVNDIFERIAGEASRLAHYNKRSTITSREIQTAVRLLLPGELAKHAVSEGTKAVT

KYTSSK
>sp|P06899|H2B1J_HUMAN Histone H2B type 1-J OS=Homo sapiens OX=9606 GN=H2BC11 PE=1 SV=3
MPEPAKSAPAPKKGSKKAVTKAQKKDGKKRKRSRKESYSIYVYKVLKQVHPDTGISSKAM
GIMNSFVNDIFERIAGEASRLAHYNKRSTITSREIQTAVRLLLPGELAKHAVSEGTKAVT
KYTSAK
>sp|Q16778|H2B2E_HUMAN Histone H2B type 2-E OS=Homo sapiens OX=9606 GN=H2BC21 PE=1 SV=3
MPEPAKSAPAPKKGSKKAVTKAQKKDGKKRKRSRKESYSIYVYKVLKQVHPDTGISSKAM
GIMNSFVNDIFERIAGEASRLAHYNKRSTITSREIQTAVRLLLPGELAKHAVSEGTKAVT
KYTSSK
>sp|P33778|H2B1B_HUMAN Histone H2B type 1-B OS=Homo sapiens OX=9606 GN=H2BC3 PE=1 SV=2
MPEPSKSAPAPKKGSKKAITKAQKKDGKKRKRSRKESYSIYVYKVLKQVHPDTGISSKAM
GIMNSFVNDIFERIAGEASRLAHYNKRSTITSREIQTAVRLLLPGELAKHAVSEGTKAVT
KYTSSK
>sp|Q8N257|H2B3B_HUMAN Histone H2B type 3-B OS=Homo sapiens OX=9606 GN=H2BU1 PE=1 SV=3
MPDPSKSAPAPKKGSKKAVTKAQKKDGKKRKRGRKESYSIYVYKVLKQVHPDTGISSKAM
GIMNSFVNDIFERIASEASRLAHYNKRSTITSREVQTAVRLLLPGELAKHAVSEGTKAVT
KYTSSK
>sp|Q6DRA6|H2B2D_HUMAN Putative histone H2B type 2-D OS=Homo sapiens OX=9606 GN=H2BC19P PE=5 SV=3
MPEPAKFAPAPKKGSKKAVTKAQKKDGKKRKRSRKESYSIYVYKVLKRVHPDTGIWCKAM
GIMNSFLNDIFERIAGEASRLAHYNKRSTITSRRSRRPCACCCPASWPSTPCPRAPRRSP
STPAPSESLPGPGARSLPPSLPPRVAGCFVSKGSFQGHLTPLVK
>sp|Q6DN03|H2B2C_HUMAN Putative histone H2B type 2-C OS=Homo sapiens OX=9606 GN=H2BC20P PE=5 SV=3
MPEPAKFAPAPKKGSKKAVTKAQKKDGKKRKRSRKESYSIYVYKVLKRVHPDTGIWCKAM
GIMNSFLNDIFERIAGEASRLAHYNKRSTITSRRSRRPCACCCPASWPSTPCPRAPRRSP
STPAPSESLPGPGARSLPPSLPPRVAGCFVSKGSFQGHLTTSVKESFLCCQSQLMFLASR
LVNFRRAHNTKHR
>sp|P62987|RL40_HUMAN Ubiquitin-60S ribosomal protein L40 OS=Homo sapiens OX=9606 GN=UBA52 PE=1 SV=2
MQIFVKTLTGKTITLEVEPSDTIENVKAKIQDKEGIPPDQQRLIFAGKQLEDGRTLSDYN
IQKESTLHLVLRLRGGIIEPSLRQLAQKYNCDKMICRKCYARLHPRAVNCRKKKCGHTNN
LRPKKKVK
>sp|P62979|RS27A_HUMAN Ubiquitin-40S ribosomal protein S27a OS=Homo sapiens OX=9606 GN=RPS27A PE=1 SV=2
MQIFVKTLTGKTITLEVEPSDTIENVKAKIQDKEGIPPDQQRLIFAGKQLEDGRTLSDYN
IQKESTLHLVLRLRGGAKKRKKKSYTTPKKNKHKRKKVKLAVLKYYKVDENGKISRLRRE
CPSDECGAGVFMASHFDRHYCGKCCLTYCFNKPEDK
>sp|P0CG47|UBB_HUMAN Polyubiquitin-B OS=Homo sapiens OX=9606 GN=UBB PE=1 SV=1
MQIFVKTLTGKTITLEVEPSDTIENVKAKIQDKEGIPPDQQRLIFAGKQLEDGRTLSDYN
IQKESTLHLVLRLRGGMQIFVKTLTGKTITLEVEPSDTIENVKAKIQDKEGIPPDQQRLI
FAGKQLEDGRTLSDYNIQKESTLHLVLRLRGGMQIFVKTLTGKTITLEVEPSDTIENVKA

KIQDKEGIPPDQQRLIFAGKQLEDGRTLSDYNIQKESTLHLVLRLRGGC
>sp|P0CG48|UBC_HUMAN Polyubiquitin-C OS=Homo sapiens OX=9606 GN=UBC PE=1 SV=3
MQIFVKTLTGKTITLEVEPSDTIENVKAKIQDKEGIPPDQQRLIFAGKQLEDGRTLSDYN
IQKESTLHLVLRLRGGMQIFVKTLTGKTITLEVEPSDTIENVKAKIQDKEGIPPDQQRLI
FAGKQLEDGRTLSDYNIQKESTLHLVLRLRGGMQIFVKTLTGKTITLEVEPSDTIENVKA
KIQDKEGIPPDQQRLIFAGKQLEDGRTLSDYNIQKESTLHLVLRLRGGMQIFVKTLTGKT
ITLEVEPSDTIENVKAKIQDKEGIPPDQQRLIFAGKQLEDGRTLSDYNIQKESTLHLVLR
LRGGMQIFVKTLTGKTITLEVEPSDTIENVKAKIQDKEGIPPDQQRLIFAGKQLEDGRTL
SDYNIQKESTLHLVLRLRGGMQIFVKTLTGKTITLEVEPSDTIENVKAKIQDKEGIPPDQ
QRLIFAGKQLEDGRTLSDYNIQKESTLHLVLRLRGGMQIFVKTLTGKTITLEVEPSDTIE
NVKAKIQDKEGIPPDQQRLIFAGKQLEDGRTLSDYNIQKESTLHLVLRLRGGMQIFVKTL
TGKTITLEVEPSDTIENVKAKIQDKEGIPPDQQRLIFAGKQLEDGRTLSDYNIQKESTLH
LVLRLRGGMQIFVKTLTGKTITLEVEPSDTIENVKAKIQDKEGIPPDQQRLIFAGKQLED
GRTLSDYNIQKESTLHLVLRLRGGV
>sp|P07478|TRY2_HUMAN Trypsin-2 OS=Homo sapiens OX=9606 GN=PRSS2 PE=1 SV=1
MNLLLILTFVAAAVAAPFDDDDKIVGGYICEENSVPYQVSLNSGYHFCGGSLISEQWVVS
AGHCYKSRIQVRLGEHNIEVLEGNEQFINAAKIIRHPKYNSRTLDNDILLIKLSSPAVIN
SRVSAISLPTAPPAAGTESLISGWGNTLSSGADYPDELQCLDAPVLSQAECEASYPGKIT
NNMFCVGFLEGGKDSCQGDSGGPVVSNGELQGIVSWGYGCAQKNRPGVYTKVYNYVDWIK
DTIAANS
>sp|P07477|TRY1_HUMAN Serine protease 1 OS=Homo sapiens OX=9606 GN=PRSS1 PE=1 SV=1
MNPLLILTFVAAALAAPFDDDDKIVGGYNCEENSVPYQVSLNSGYHFCGGSLINEQWVVS
AGHCYKSRIQVRLGEHNIEVLEGNEQFINAAKIIRHPQYDRKTLNNDIMLIKLSSRAVIN
ARVSTISLPTAPPATGTKCLISGWGNTASSGADYPDELQCLDAPVLSQAKCEASYPGKIT
SNMFCVGFLEGGKDSCQGDSGGPVVCNGQLQGVVSWGDGCAQKNKPGVYTKVYNYVKWIK
NTIAANS
>sp|Q8NHM4|TRY6_HUMAN Putative trypsin-6 OS=Homo sapiens OX=9606 GN=PRSS3P2 PE=5 SV=2
MNPLLILAFVGAAVAVPFDDDDKIVGGYTCEENSVPYQVSLNSGSHFCGGSLISEQWVVS
AGHCYKPHIQVRLGEHNIEVLEGNEQFINAAKIIRHPKYNRIILNNDIMLIKLSTPAVIN
AHVSTISLPTAPPAAGTECLISGWGNTLSSGADYPDELQCLDAPVLTQAKCKASYPLKIT
SNMFCVGFLEGGKDSCQGDSGGPVVCNGQLQGIVSWGYGCAQKRRPGVYTKVYNYVDWIK
DTIAANS
>sp|P28074|PSB5_HUMAN Proteasome subunit beta type-5 OS=Homo sapiens OX=9606 GN=PSMB5 PE=1 SV=3
MALASVLERPLPVNQRGFFGLGGRADLLDLGPGSLSDGLSLAAPGWGVPEEPGIEMLHGT
TTLAFKFRHGVIVAADSRATAGAYIASQTVKKVIEINPYLLGTMAGGAADCSFWERLLAR
QCRIYELRNKERISVAAASKLLANMVYQYKGMGLSMGTMICGWDKRGPGLYYVDSEGNRI
SGATFSVGSGSVYAYGVMDRGYSYDLEVEQAYDLARRAIYQATYRDAYSGGAVNLYHVRE
DGWIRVSSDNVADLHEKYSGSTP
>sp|P09972|ALDOC_HUMAN Fructose-bisphosphate aldolase C OS=Homo sapiens OX=9606 GN=ALDOC PE=1 SV=2
MPHSYPALSAEQKKELSDIALRIVAPGKGILAADESVGSMAKRLSQIGVENTEENRRLYR
QVLFSADDRVKKCIGGVIFFHETLYQKDDNGVPFVRTIQDKGIVVGIKVDKGVVPLAGTD

GETTTQGLDGLSERCAQYKKDGADFAKWRCVLKISERTPSALAILENANVLARYASICQQ
NGIVPIVEPEILPDGDHDLKRCQYVTEKVLAAVYKALSDHHVYLEGTLLKPNMVTPGHAC
PIKYTPEEIAMATVTALRRTVPPAVPGVTFLSGGQSEEEASFNLNAINRCPLPRPWALTF
SYGRALQASALNAWRGQRDNAGAATEEFIKRAEVNGLAAQGKYEGSGEDGGAAAQSLYIA
NHAY
>sp|P49189|AL9A1_HUMAN 4-trimethylaminobutyraldehyde dehydrogenase OS=Homo sapiens OX=9606 GN=ALDH9A1 PE=1 SV=3
MSTGTFVVSQPLNYRGGARVEPADASGTEKAFEPATGRVIATFTCSGEKEVNLAVQNAKA
AFKIWSQKSGMERCRILLEAARIIREREDEIATMECINNGKSIFEARLDIDISWQCLEYY
AGLAASMAGEHIQLPGGSFGYTRREPLGVCVGIGAWNYPFQIASWKSAPALACGNAMVFK
PSPFTPVSALLLAEIYSEAGVPPGLFNVVQGGAATGQFLCQHPDVAKVSFTGSVPTGMKI
MEMSAKGIKPVTLELGGKSPLIIFSDCDMNNAVKGALMANFLTQGQVCCNGTRVFVQKEI
LDKFTEEVVKQTQRIKIGDPLLEDTRMGPLINRPHLERVLGFVKVAKEQGAKVLCGGDIY
VPEDPKLKDGYYMRPCVLTNCRDDMTCVKEEIFGPVMSILSFDTEAEVLERANDTTFGLA
AGVFTRDIQRAHRVVAELQAGTCFINNYNVSPVELPFGGYKKSGFGRENGRVTIEYYSQL
KTVCVEMGDVESAF
>sp|P05387|RLA2_HUMAN 60S acidic ribosomal protein P2 OS=Homo sapiens OX=9606 GN=RPLP2 PE=1 SV=1
MRYVASYLLAALGGNSSPSAKDIKKILDSVGIEADDDRLNKVISELNGKNIEDVIAQGIG
KLASVPAGGAVAVSAAPGSAAPAAGSAPAAAEEKKDEKKEESEESDDDMGFGLFD
>sp|P10599|THIO_HUMAN Thioredoxin OS=Homo sapiens OX=9606 GN=TXN PE=1 SV=3
MVKQIESKTAFQEALDAAGDKLVVVDFSATWCGPCKMIKPFFHSLSEKYSNVIFLEVDVD
DCQDVASECEVKCMPTFQFFKKGQKVGEFSGANKEKLEATINELV
>sp|P55786|PSA_HUMAN Puromycin-sensitive aminopeptidase OS=Homo sapiens OX=9606 GN=NPEPPS PE=1 SV=2
MWLAAAAPSLARRLLFLGPPPPPLLLLVFSRSSRRRLHSLGLAAMPEKRPFERLPADVSP
INYSLCLKPDLLDFTFEGKLEAAAQVRQATNQIVMNCADIDIITASYAPEGDEEIHATGF
NYQNEDEKVTLSFPSTLQTGTGTLKIDFVGELNDKMKGFYRSKYTTPSGEVRYAAVTQFE
ATDARRAFPCWDEPAIKATFDISLVVPKDRVALSNMNVIDRKPYPDDENLVEVKFARTPV
MSTYLVAFVVGEYDFVETRSKDGVCVRVYTPVGKAEQGKFALEVAAKTLPFYKDYFNVPY
PLPKIDLIAIADFAAGAMENWGLVTYRETALLIDPKNSCSSSRQWVALVVGHELAHQWFG
NLVTMEWWTHLWLNEGFASWIEYLCVDHCFPEYDIWTQFVSADYTRAQELDALDNSHPIE
VSVGHPSEVDEIFDAISYSKGASVIRMLHDYIGDKDFKKGMNMYLTKFQQKNAATEDLWE
SLENASGKPIAAVMNTWTKQMGFPLIYVEAEQVEDDRLLRLSQKKFCAGGSYVGEDCPQW
MVPITISTSEDPNQAKLKILMDKPEMNVVLKNVKPDQWVKLNLGTVGFYRTQYSSAMLES
LLPGIRDLSLPPVDRLGLQNDLFSLARAGIISTVEVLKVMEAFVNEPNYTVWSDLSCNLG
ILSTLLSHTDFYEEIQEFVKDVFSPIGERLGWDPKPGEGHLDALLRGLVLGKLGKAGHKA
TLEEARRRFKDHVEGKQILSADLRSPVYLTVLKHGDGTTLDIMLKLHKQADMQEEKNRIE
RVLGATLLPDLIQKVLTFALSEEVRPQDTVSVIGGVAGGSKHGRKAAWKFIKDNWEELYN
RYQGGFLISRLIKLSVEGFAVDKMAGEVKAFFESHPAPSAERTIQQCCENILLNAAWLKR
DAESIHQYLLQRKASPPTV
>sp|A6NEC2|PSAL_HUMAN Puromycin-sensitive aminopeptidase-like protein OS=Homo sapiens OX=9606 GN=NPEPPSL1 PE=2 SV=3
MWLAAAAPSLARRLLFLGPPPPPLLLLVFSRSSRRRLHSLGLAAMPEKRPFERLPADVSP
INCSLCLKPDLLDFTFEGKLEAAAQVRQATNQIVMNCADIDIITASYAPEGDEEIHATGF
NYQNEDEKVTLSFPSTLQTGTGTLKIDFVGELNDKMKGFYRSKYTTPSGEVRYAAVTQFE

ATDARRAFPCWDERAIKATFDISLVVPKDRVALSNMNVIDRKPYPDDENLVEVKFARTPV
TSTYLVAFVVGEYDFVETRSKDGVCVCVYTPVGKAEQGKFALEVAAKTLPFYKDYFNVPY
PLPKIDLIAIADFAAGAMENWDLVTYRETALLIDPKNSCSSSRQWVALVVGHELAHQWFG
NLVTMEWWTHLRLNEGFASWIEYLCVDHCFPEYDIWTQFVSADYTRAQELDALDNSHPIE
VSVGHPSEVDEIFDAISYSKGASVIRMLHDYIGDKDFKKGMNMYLTKFQQKNAAAGNL
>sp|P22531|SPR2E_HUMAN Small proline-rich protein 2E OS=Homo sapiens OX=9606 GN=SPRR2E PE=2 SV=2
MSYQQQQCKQPCQPPPVCPTPKCPEPCPPPKCPEPCPPPKCPQPCPPQQCQQKCPPVTPS
PPCQPKCPPKSK
>sp|P35325|SPR2B_HUMAN Small proline-rich protein 2B OS=Homo sapiens OX=9606 GN=SPRR2B PE=2 SV=1
MSYQQQQCKQPCQPPPVCPTPKCPEPCPPPKCPEPCPPPKCPQPCPPQQCQQKYPPVTPS
PPCQPKYPPKSK
>sp|P22532|SPR2D_HUMAN Small proline-rich protein 2D OS=Homo sapiens OX=9606 GN=SPRR2D PE=2 SV=2
MSYQQQQCKQPCQPPPVCPTPKCPEPCPPPKCPEPCPSPKCPQPCPPQQCQQKYPPVTPS
PPCQPKCPPKSK
>sp|P35326|SPR2A_HUMAN Small proline-rich protein 2A OS=Homo sapiens OX=9606 GN=SPRR2A PE=1 SV=1
MSYQQQQCKQPCQPPPVCPTPKCPEPCPPPKCPEPCPPPKCPQPCPPQQCQQKYPPVTPS
PPCQSKYPPKSK
>sp|Q9BYE4|SPR2G_HUMAN Small proline-rich protein 2G OS=Homo sapiens OX=9606 GN=SPRR2G PE=3 SV=1
MSYQQQQCKQPCQPPPVCPTPKCPEPCPPPKCPEPYLPPPCPPEHCPPPPCQDKCPPVQP
YPPCQQKYPPKSK
>sp|Q96RM1|SPR2F_HUMAN Small proline-rich protein 2F OS=Homo sapiens OX=9606 GN=SPRR2F PE=3 SV=1
MSYQQQQCKQPCQPPPVCPAPKCPEPCPPPKCPEPCPPSKCPQSCPPQQCQQKCPPVTPS
PPCQPKCPPKSK
>sp|P38606|VATA_HUMAN V-type proton ATPase catalytic subunit A OS=Homo sapiens OX=9606 GN=ATP6V1A PE=1 SV=2
MDFSKLPKILDEDKESTFGYVHGVSGPVVTACDMAGAAMYELVRVGHSELVGEIIRLEGD
MATIQVYEETSGVSVGDPVLRTGKPLSVELGPGIMGAIFDGIQRPLSDISSQTQSIYIPR
GVNVSALSRDIKWDFTPCKNLRVGSHITGGDIYGIVSENSLIKHKIMLPPRNRGTVTYIA
PPGNYDTSDVVLELEFEGVKEKFTMVQVWPVRQVRPVTEKLPANHPLLTGQRVLDALFPC
VQGGTTAIPGAFGCGKTVISQSLSKYSNSDVIIYVGCGERGNEMSEVLRDFPELTMEVDG
KVESIMKRTALVANTSNMPVAAREASIYTGITLSEYFRDMGYHVSMMADSTSRWAEALRE
ISGRLAEMPADSGYPAYLGARLASFYERAGRVKCLGNPEREGSVSIVGAVSPPGGDFSDP
VTSATLGIVQVFWGLDKKLAQRKHFPSVNWLISYSKYMRALDEYYDKHFTEFVPLRTKAK
EILQEEEDLAEIVQLVGKASLAETDKITLEVAKLIKDDFLQQNGYTPYDRFCPFYKTVGM
LSNMIAFYDMARRAVETTAQSDNKITWSIIREHMGDILYKLSSMKFKDPLKDGEAKIKSD
YAQLLEDMQNAFRSLED
>sp|P29373|RABP2_HUMAN Cellular retinoic acid-binding protein 2 OS=Homo sapiens OX=9606 GN=CRABP2 PE=1 SV=2
MPNFSGNWKIIRSENFEELLKVLGVNVMLRKIAVAAASKPAVEIKQEGDTFYIKTSTTVR
TTEINFKVGEEFEEQTVDGRPCKSLVKWESENKMVCEQKLLKGEGPKTSWTRELTNDGEL
ILTMTADDVVCTRVYVRE

>sp|Q2M2I5|K1C24_HUMAN Keratin, type I cytoskeletal 24 OS=Homo sapiens OX=9606 GN=KRT24 PE=1 SV=1
MSCSSRASSSRAGGSSSARVSAGGSSFSSGSRCGLGGSSAQGFRGGASSCSLSGGSSGAF
GGSFGGGFGSCSVGGGFGGASGSGTGFGGGSSFGGVSGFGRGSGFCGSSRFSSGATGGFY
SYGGGMGGGVGDGGLFSGGEKQTMQNLNDRLANYLDKVRALEEANTDLENKIKEWYDKYG
PGSGDGGSGRDYSKYYSIIEDLRNQIIAATVENAGIILHIDNARLAADDFRLKYENELCL
RQSVEADINGLRKVLDDLTMTRSDLEMQIESFTEELAYLRKNHEEEMKNMQGSSGGEVTV
EMNAAPGTDLTKLLNDMRAQYEELAEQNRREAEERFNKQSASLQAQISTDAGAATSAKNE
ITELKRTLQALEIELQSQLAMKSSLEGTLADTEAGYVAQLSEIQTQISALEEEICQIWGE
TKCQNAEYKQLLDIKTRLEVEIETYRRLLDGEGGGSSFAEFGGRNSGSVNMGSRDLVSGD
SRSGSCSGQGRDSSKTRVTKTIVEELVDGKVVSSQVSSISEVKVK
>sp|P07108|ACBP_HUMAN Acyl-CoA-binding protein OS=Homo sapiens OX=9606 GN=DBI PE=1 SV=2
MSQAEFEKAAEEVRHLKTKPSDEEMLFIYGHYKQATVGDINTERPGMLDFTGKAKWDAWN
ELKGTSKEDAMKAYINKVEELKKKYGI
>sp|O75223|GGCT_HUMAN Gamma-glutamylcyclotransferase OS=Homo sapiens OX=9606 GN=GGCT PE=1 SV=1
MANSGCKDVTGPDEESFLYFAYGSNLLTERIHLRNPSAAFFCVARLQDFKLDFGNSQGKT
SQTWHGGIATIFQSPGDEVWGVVWKMNKSNLNSLDEQEGVKSGMYVVIEVKVATQEGKEI
TCRSYLMTNYESAPPSPQYKKIICMGAKENGLPLEYQEKLKAIEPNDYTGKVSEEIEDII
KKGETQTL
>sp|P35030|TRY3_HUMAN Trypsin-3 OS=Homo sapiens OX=9606 GN=PRSS3 PE=1 SV=2
MCGPDDRCPARWPGPGRAVKCGKGLAAARPGRVERGGAQRGGAGLELHPLLGGRTWRAAR
DADGCEALGTVAVPFDDDDKIVGGYTCEENSLPYQVSLNSGSHFCGGSLISEQWVVSAAH
CYKTRIQVRLGEHNIKVLEGNEQFINAAKIIRHPKYNRDTLDNDIMLIKLSSPAVINARV
STISLPTTPPAAGTECLISGWGNTLSFGADYPDELKCLDAPVLTQAECKASYPGKITNSM
FCVGFLEGGKDSCQRDSGGPVVCNGQLQGVVSWGHGCAWKNRPGVYTKVYNYVDWIKDTI
AANS
>sp|Q9UJ70|NAGK_HUMAN N-acetyl-D-glucosamine kinase OS=Homo sapiens OX=9606 GN=NAGK PE=1 SV=4
MAAIYGGVEGGGTRSEVLLVSEDGKILAEADGLSTNHWLIGTDKCVERINEMVNRAKRKA
GVDPLVPLRSLGLSLSGGDQEDAGRILIEELRDRFPYLSESYLITTDAAGSIATATPDGG
VVLISGTGSNCRLINPDGSESGCGGWGHMMGDEGSAYWIAHQAVKIVFDSIDNLEAAPHD
IGYVKQAMFHYFQVPDRLGILTHLYRDFDKCRFAGFCRKIAEGAQQGDPLSRYIFRKAGE
MLGRHIVAVLPEIDPVLFQGKIGLPILCVGSVWKSWELLKEGFLLALTQGREIQAQNFFS
SFTLMKLRHSSALGGASLGARHIGHLLPMDYSANAIAFYSYTFS
>sp|P20073|ANXA7_HUMAN Annexin A7 OS=Homo sapiens OX=9606 GN=ANXA7 PE=1 SV=3
MSYPGYPPTGYPPFPGYPPAGQESSFPPSGQYPYPSGFPPMGGGAYPQVPSSGYPGAGGY
PAPGGYPAPGGYPGAPQPGGAPSYPGVPPGQGFGVPPGGAGFSGYPQPPSQSYGGGPAQV
PLPGGFPGGQMPSQYPGGQPTYPSQINTDSFSSYPVFSPVSLDYSSEPATVTQVTQGTIR
PAANFDAIRDAEILRKAMKGFGTDEQAIVDVVANRSNDQRQKIKAAFKTSYGKDLIKDLK
SELSGNMEELILALFMPPTYYDAWSLRKAMQGAGTQERVLIEILCTRTNQEIREIVRCYQ
SEFGRDLEKDIRSDTSGHFERLLVSMCQGNRDENQSINHQMAQEDAQRLYQAGEGRLGTD
ESCFNMILATRSFPQLRATMEAYSRMANRDLLSSVSREFSGYVESGLKTILQCALNRPAF
FAERLYYAMKGAGTDDSTLVRIVVTRSEIDLVQIKQMFAQMYQKTLGTMIAGDTSGDYRR

LLLAIVGQ
>sp|Q01518|CAP1_HUMAN Adenylyl cyclase-associated protein 1 OS=Homo sapiens OX=9606 GN=CAP1 PE=1 SV=5
MADMQNLVERLERAVGRLEAVSHTSDMHRGYADSPSKAGAAPYVQAFDSLLAGPVAEYLK
ISKEIGGDVQKHAEMVHTGLKLERALLVTASQCQQPAENKLSDLLAPISEQIKEVITFRE
KNRGSKLFNHLSAVSESIQALGWVAMAPKPGPYVKEMNDAAMFYTNRVLKEYKDVDKKHV
DWVKAYLSIWTELQAYIKEFHTTGLAWSKTGPVAKELSGLPSGPSAGSCPPPPPPCPPPP
PVSTISCSYESASRSSLFAQINQGESITHALKHVSDDMKTHKNPALKAQSGPVRSGPKPF
SAPKPQTSPSPKRATKKEPAVLELEGKKWRVENQENVSNLVIEDTELKQVAYIYKCVNTT
LQIKGKINSITVDNCKKLGLVFDDVVGIVEIINSKDVKVQVMGKVPTISINKTDGCHAYL
SKNSLDCEIVSAKSSEMNVLIPTEGGDFNEFPVPEQFKTLWNGQKLVTTVTEIAG
>sp|P02679|FIBG_HUMAN Fibrinogen gamma chain OS=Homo sapiens OX=9606 GN=FGG PE=1 SV=3
MSWSLHPRNLILYFYALLFLSSTCVAYVATRDNCCILDERFGSYCPTTCGIADFLSTYQT
KVDKDLQSLEDILHQVENKTSEVKQLIKAIQLTYNPDESSKPNMIDAATLKSRKMLEEIM
KYEASILTHDSSIRYLQEIYNSNNQKIVNLKEKVAQLEAQCQEPCKDTVQIHDITGKDCQ
DIANKGAKQSGLYFIKPLKANQQFLVYCEIDGSGNGWTVFQKRLDGSVDFKKNWIQYKEG
FGHLSPTGTTEFWLGNEKIHLISTQSAIPYALRVELEDWNGRTSTADYAMFKVGPEADKY
RLTYAYFAGGDAGDAFDGFDFGDDPSDKFFTSHNGMQFSTWDNDNDKFEGNCAEQDGSGW
WMNKCHAGHLNGVYYQGGTYSKASTPNGYDNGIIWATWKTRWYSMKKTTMKIIPFNRLTI
GEGQQHHLGGAKQVRPEHPAETEYDSLYPEDDL
>sp|Q8WUM4|PDC6I_HUMAN Programmed cell death 6-interacting protein OS=Homo sapiens OX=9606 GN=PDCD6IP PE=1 SV=1
MATFISVQLKKTSEVDLAKPLVKFIQQTYPSGGEEQAQYCRAAEELSKLRRAAVGRPLDK
HEGALETLLRYYDQICSIEPKFPFSENQICLTFTWKDAFDKGSLFGGSVKLALASLGYEK
SCVLFNCAALASQIAAEQNLDNDEGLKIAAKHYQFASGAFLHIKETVLSALSREPTVDIS
PDTVGTLSLIMLAQAQEVFFLKATRDKMKDAIIAKLANQAADYFGDAFKQCQYKDTLPKE
VFPVLAAKHCIMQANAEYHQSILAKQQKKFGEEIARLQHAAELIKTVASRYDEYVNVKDF
SDKINRALAAAKKDNDFIYHDRVPDLKDLDPIGKATLVKSTPVNVPISQKFTDLFEKMVP
VSVQQSLAAYNQRKADLVNRSIAQMREATTLANGVLASLNLPAAIEDVSGDTVPQSILTK
SRSVIEQGGIQTVDQLIKELPELLQRNREILDESLRLLDEEEATDNDLRAKFKERWQRTP
SNELYKPLRAEGTNFRTVLDKAVQADGQVKECYQSHRDTIVLLCKPEPELNAAIPSANPA
KTMQGSEVVNVLKSLLSNLDEVKKEREGLENDLKSVNFDMTSKFLTALAQDGVINEEALS
VTELDRVYGGLTTKVQESLKKQEGLLKNIQVSHQEFSKMKQSNNEANLREEVLKNLATAY
DNFVELVANLKEGTKFYNELTEILVRFQNKCSDIVFARKTERDELLKDLQQSIAREPSAP
SIPTPAYQSSPAGGHAPTPPTPAPRTMPPTKPQPPARPPPPVLPANRAPSATAPSPVGAG
TAAPAPSQTPGSAPPPQAQGPPYPTYPGYPGYCQMPMPMGYNPYAYGQYNMPYPPVYHQS
PGQAPYPGPQQPSYPFPQPPQQSYYPQQ
>sp|P11217|PYGM_HUMAN Glycogen phosphorylase, muscle form OS=Homo sapiens OX=9606 GN=PYGM PE=1 SV=6
MSRPLSDQEKRKQISVRGLAGVENVTELKKNFNRHLHFTLVKDRNVATPRDYYFALAHTV
RDHLVGRWIRTQQHYYEKDPKRIYYLSLEFYMGRTLQNTMVNLALENACDEATYQLGLDM
EELEEIEEDAGLGNGGLGRLAACFLDSMATLGLAAYGYGIRYEFGIFNQKISGGWQMEEA
DDWLRYGNPWEKARPEFTLPVHFYGHVEHTSQGAKWVDTQVVLAMPYDTPVPGYRNNVVN
TMRLWSAKAPNDFNLKDFNVGGYIQAVLDRNLAENISRVLYPNDNFFEGKELRLKQEYFV
VAATLQDIIRRFKSSKFGCRDPVRTNFDAFPDKVAIQLNDTHPSLAIPELMRILVDLERM
DWDKAWDVTVRTCAYTNHTVLPEALERWPVHLLETLLPRHLQIIYEINQRFLNRVAAAFP

GDVDRLRRMSLVEEGAVKRINMAHLCIAGSHAVNGVARIHSEILKKTIFKDFYELEPHKF
QNKTNGITPRRWLVLCNPGLAEVIAERIGEDFISDLDQLRKLLSFVDDEAFIRDVAKVKQ
ENKLKFAAYLEREYKVHINPNSLFDIQVKRIHEYKRQLLNCLHVITLYNRIKREPNKFFV
PRTVMIGGKAAPGYHMAKMIIRLVTAIGDVVNHDPAVGDRLRVIFLENYRVSLAEKVIPA
ADLSEQISTAGTEASGTGNMKFMLNGALTIGTMDGANVEMAEEAGEENFFIFGMRVEDVD
KLDQRGYNAQEYYDRIPELRQVIEQLSSGFFSPKQPDLFKDIVNMLMHHDRFKVFADYED
YIKCQEKVSALYKNPREWTRMVIRNIATSGKFSSDRTIAQYAREIWGVEPSRQRLPAPDE
AI
>sp|P11940|PABP1_HUMAN Polyadenylate-binding protein 1 OS=Homo sapiens OX=9606 GN=PABPC1 PE=1 SV=2
MNPSAPSYPMASLYVGDLHPDVTEAMLYEKFSPAGPILSIRVCRDMITRRSLGYAYVNFQ
QPADAERALDTMNFDVIKGKPVRIMWSQRDPSLRKSGVGNIFIKNLDKSIDNKALYDTFS
AFGNILSCKVVCDENGSKGYGFVHFETQEAAERAIEKMNGMLLNDRKVFVGRFKSRKERE
AELGARAKEFTNVYIKNFGEDMDDERLKDLFGKFGPALSVKVMTDESGKSKGFGFVSFER
HEDAQKAVDEMNGKELNGKQIYVGRAQKKVERQTELKRKFEQMKQDRITRYQGVNLYVKN
LDDGIDDERLRKEFSPFGTITSAKVMMEGGRSKGFGFVCFSSPEEATKAVTEMNGRIVAT
KPLYVALAQRKEERQAHLTNQYMQRMASVRAVPNPVINPYQPAPPSGYFMAAIPQTQNRA
AYYPPSQIAQLRPSPRWTAQGARPHPFQNMPGAIRPAAPRPPFSTMRPASSQVPRVMSTQ
RVANTSTQTMGPRPAAAAAAATPAVRTVPQYKYAAGVRNPQQHLNAQPQVTMQQPAVHVQ
GQEPLTASMLASAPPQEQKQMLGERLFPLIQAMHPTLAGKITGMLLEIDNSELLHMLESP
ESLRSKVDEAVAVLQAHQAKEAAQKAVNSATGVPTV
>sp|Q13310|PABP4_HUMAN Polyadenylate-binding protein 4 OS=Homo sapiens OX=9606 GN=PABPC4 PE=1 SV=1
MNAAASSYPMASLYVGDLHSDVTEAMLYEKFSPAGPVLSIRVCRDMITRRSLGYAYVNFQ
QPADAERALDTMNFDVIKGKPIRIMWSQRDPSLRKSGVGNVFIKNLDKSIDNKALYDTFS
AFGNILSCKVVCDENGSKGYAFVHFETQEAADKAIEKMNGMLLNDRKVFVGRFKSRKERE
AELGAKAKEFTNVYIKNFGEEVDDESLKELFSQFGKTLSVKVMRDPNGKSKGFGFVSYEK
HEDANKAVEEMNGKEISGKIIFVGRAQKKVERQAELKRKFEQLKQERISRYQGVNLYIKN
LDDTIDDEKLRKEFSPFGSITSAKVMLEDGRSKGFGFVCFSSPEEATKAVTEMNGRIVGS
KPLYVALAQRKEERKAHLTNQYMQRVAGMRALPANAILNQFQPAAGGYFVPAVPQAQGRP
PYYTPNQLAQMRPNPRWQQGGRPQGFQGMPSAIRQSGPRPTLRHLAPTGSECPDRLAMDF
GGAGAAQQGLTDSCQSGGVPTAVQNLAPRAAVAAAAPRAVAPYKYASSVRSPHPAIQPLQ
APQPAVHVQGQEPLTASMLAAAPPQEQKQMLGERLFPLIQTMHSNLAGKITGMLLEIDNS
ELLHMLESPESLRSKVDEAVAVLQAHHAKKEAAQKVGAVAAATS
>sp|Q9H361|PABP3_HUMAN Polyadenylate-binding protein 3 OS=Homo sapiens OX=9606 GN=PABPC3 PE=1 SV=2
MNPSTPSYPTASLYVGDLHPDVTEAMLYEKFSPAGPILSIRICRDLITSGSSNYAYVNFQ
HTKDAEHALDTMNFDVIKGKPVRIMWSQRDPSLRKSGVGNIFVKNLDKSINNKALYDTVS
AFGNILSCNVVCDENGSKGYGFVHFETHEAAERAIKKMNGMLLNGRKVFVGQFKSRKERE
AELGARAKEFPNVYIKNFGEDMDDERLKDLFGKFGPALSVKVMTDESGKSKGFGFVSFER
HEDAQKAVDEMNGKELNGKQIYVGRAQKKVERQTELKRTFEQMKQDRITRYQVVNLYVKN
LDDGIDDERLRKAFSPFGTITSAKVMMEGGRSKGFGFVCFSSPEEATKAVTEMNGRIVAT
KPLYVALAQRKEERQAYLTNEYMQRMASVRAVPNQRAPPSGYFMTAVPQTQNHAAYYPPS
QIARLRPSPRWTAQGARPHPFQNKPSAIRPGAPRVPFSTMRPASSQVPRVMSTQRVANTS
TQTVGPRPAAAAAAAATPAVRTVPRYKYAAGVRNPQQHRNAQPQVTMQQLAVHVQGQETL
TASRLASAPPQKQKQMLGERLFPLIQAMHPTLAGKITGMLLEIDNSELLYMLESPESLRS
KVDEAVAVLQAHQAKEATQKAVNSATGVPTV

>sp|Q4VXU2|PAP1L_HUMAN Polyadenylate-binding protein 1-like OS=Homo sapiens OX=9606 GN=PABPC1L PE=2 SV=1
MNASGSGYPLASLYVGDLHPDVTEAMLYEKFSPAGPILSIRVCRDVATRRSLGYAYINFQ
QPADAERALDTMNFEMLKGQPIRIMWSQRDPGLRKSGVGNIFIKNLEDSIDNKALYDTFS
TFGNILSCKVACDEHGSRGFGFVHFETHEAAQQAINTMNGMLLNDRKVFVGHFKSRRERE
AELGARALEFTNIYVKNLPVDVDEQGLQDLFSQFGKMLSVKVMRDNSGHSRCFGFVNFEK
HEEAQKAVVHMNGKEVSGRLLYAGRAQKRVERQNELKRRFEQMKQDRLRRYQGVNLYVKN
LDDSIDDDKLRKEFSPYGVITSAKVMTEGGHSKGFGFVCFSSPEEATKAVTEMNGRIVGT
KPLYVALAQRKEERKAILTNQYMQRLSTMRTLSNPLLGSFQQPSSYFLPAMPQPPAQAAY
YGCGPVTPTQPAPRWTSQPPRPSCASMVRPPVVPRRPPAHISSVRQASTQVPRTVPHTQR
VANIGTQTTGPSGVGCCTPGRPLLPCKCSSAAHSTYRVQEPAVHIPGQEPLTASMLAAAP
LHEQKQMIGERLYPLIHDVHTQLAGKITGMLLEIDNSELLLMLESPESLHAKIDEAVAVL
QAHQAMEQPKAYMH
>sp|Q5JQF8|PAP1M_HUMAN Polyadenylate-binding protein 1-like 2 OS=Homo sapiens OX=9606 GN=PABPC1L2B PE=2 SV=1
MASLYVGDLHPEVTEAMLYEKFSPAGPILSIRICRDKITRRSLGYAYVNYQQPVDAKRAL
ETLNFDVIKGRPVRIMWSQRDPSLRKSGVGNVFIKNLGKTIDNKALYNIFSAFGNILSCK
VACDEKGPKGYGFVHFQKQESAERAIDVMNGMFLNYRKIFVGRFKSHKEREAERGAWARQ
STSADVKDFEEDTDEEATLR
>sp|O75342|LX12B_HUMAN Arachidonate 12-lipoxygenase, 12R-type OS=Homo sapiens OX=9606 GN=ALOX12B PE=1 SV=1
MATYKVRVATGTDLLSGTRDSISLTIVGTQGESHKQLLNHFGRDFATGAVGQYTVQCPQD
LGELIIIRLHKERYAFFPKDPWYCNYVQICAPNGRIYHFPAYQWMDGYETLALREATGKT
TADDSLPVLLEHRKEEIRAKQDFYHWRVFLPGLPSYVHIPSYRPPVRRHRNPNRPEWNGY
IPGFPILINFKATKFLNLNLRYSFLKTASFFVRLGPMALAFKVRGLLDCKHSWKRLKDIR
KIFPGKKSVVSEYVAEHWAEDTFFGYQYLNGVNPGLIRRCTRIPDKFPVTDDMVAPFLGE
GTCLQAELEKGNIYLADYRIMEGIPTVELSGRKQHHCAPLCLLHFGPEGKMMPIAIQLSQ
TPGPDCPIFLPSDSEWDWLLAKTWVRYAEFYSHEAIAHLLETHLIAEAFCLALLRNLPMC
HPLYKLLIPHTRYTVQINSIGRAVLLNEGGLSAKGMSLGVEGFAGVMVRALSELTYDSLY
LPNDFVERGVQDLPGYYYRDDSLAVWNALEKYVTEIITYYYPSDAAVEGDPELQSWVQEI
FKECLLGRESSGFPRCLRTVPELIRYVTIVIYTCSAKHAAVNTGQMEFTAWMPNFPASMR
NPPIQTKGLTTLETFMDTLPDVKTTCITLLVLWTLSREPDDRRPLGHFPDIHFVEEAPRR
SIEAFRQRLNQISHDIRQRNKCLPIPYYYLDPVLIENSISI
>sp|P60660|MYL6_HUMAN Myosin light polypeptide 6 OS=Homo sapiens OX=9606 GN=MYL6 PE=1 SV=2
MCDFTEDQTAEFKEAFQLFDRTGDGKILYSQCGDVMRALGQNPTNAEVLKVLGNPKSDEM
NVKVLDFEHFLPMLQTVAKNKDQGTYEDYVEGLRVFDKEGNGTVMGAEIRHVLVTLGEKM
TEEEVEMLVAGHEDSNGCINYEAFVRHILSG
>sp|P14649|MYL6B_HUMAN Myosin light chain 6B OS=Homo sapiens OX=9606 GN=MYL6B PE=1 SV=1
MPPKKDVPVKKPAGPSISKPAAKPAAAGAPPAKTKAEPAVPQAPQKTQEPPVDLSKVVIE
FNKDQLEEFKEAFELFDRVGDGKILYSQCGDVMRALGQNPTNAEVLKVLGNPKSDELKSR
RVDFETFLPMLQAVAKNRGQGTYEDYLEGFRVFDKEGNGKVMGAELRHVLTTLGEKMTEE
EVETVLAGHEDSNGCINYEAFLKHILSV
>sp|P61978|HNRPK_HUMAN Heterogeneous nuclear ribonucleoprotein K OS=Homo sapiens OX=9606 GN=HNRNPK PE=1 SV=1
METEQPEETFPNTETNGEFGKRPAEDMEEEQAFKRSRNTDEMVELRILLQSKNAGAVIGK

GGKNIKALRTDYNASVSVPDSSGPERILSISADIETIGEILKKIIPTLEEGLQLPSPTAT
SQLPLESDAVECLNYQHYKGSDFDCELRLLIHQSLAGGIIGVKGAKIKELRENTQTTIKL
FQECCPHSTDRVVLIGGKPDRVVECIKIILDLISESPIKGRAQPYDPNFYDETYDYGGFT
MMFDDRRGRPVGFPMRGRGGFDRMPPGRGGRPMPPSRRDYDDMSPRRGPPPPPPGRGGRG
GSRARNLPLPPPPPPRGGDLMAYDRRGRPGDRYDGMVGFSADETWDSAIDTWSPSEWQMA
YEPQGGSGYDYSYAGGRGSYGDLGGPIITTQVTIPKDLAGSIIGKGGQRIKQIRHESGAS
IKIDEPLEGSEDRIITITGTQDQIQNAQYLLQNSVKQYSGKFF
>sp|P26641|EF1G_HUMAN Elongation factor 1-gamma OS=Homo sapiens OX=9606 GN=EEF1G PE=1 SV=3
MAAGTLYTYPENWRAFKALIAAQYSGAQVRVLSAPPHFHFGQTNRTPEFLRKFPAGKVPA
FEGDDGFCVFESNAIAYYVSNEELRGSTPEAAAQVVQWVSFADSDIVPPASTWVFPTLGI
MHHNKQATENAKEEVRRILGLLDAYLKTRTFLVGERVTLADITVVCTLLWLYKQVLEPSF
RQAFPNTNRWFLTCINQPQFRAVLGEVKLCEKMAQFDAKKFAETQPKKDTPRKEKGSREE
KQKPQAERKEEKKAAAPAPEEEMDECEQALAAEPKAKDPFAHLPKSTFVLDEFKRKYSNE
DTLSVALPYFWEHFDKDGWSLWYSEYRFPEELTQTFMSCNLITGMFQRLDKLRKNAFASV
ILFGTNNSSSISGVWVFRGQELAFPLSPDWQVDYESYTWRKLDPGSEETQTLVREYFSWE
GAFQHVGKAFNQGKIFK
>sp|P02647|APOA1_HUMAN Apolipoprotein A-I OS=Homo sapiens OX=9606 GN=APOA1 PE=1 SV=1
MKAAVLTLAVLFLTGSQARHFWQQDEPPQSPWDRVKDLATVYVDVLKDSGRDYVSQFEGS
ALGKQLNLKLLDNWDSVTSTFSKLREQLGPVTQEFWDNLEKETEGLRQEMSKDLEEVKAK
VQPYLDDFQKKWQEEMELYRQKVEPLRAELQEGARQKLHELQEKLSPLGEEMRDRARAHV
DALRTHLAPYSDELRQRLAARLEALKENGGARLAEYHAKATEHLSTLSEKAKPALEDLRQ
GLLPVLESFKVSFLSALEEYTKKLNTQ
>sp|P05496|AT5G1_HUMAN ATP synthase F(0) complex subunit C1, mitochondrial OS=Homo sapiens OX=9606 GN=ATP5MC1 PE=1 SV=2
MQTAGALFISPALIRCCTRGLIRPVSASFLNSPVNSSKQPSYSNFPLQVARREFQTSVVS
RDIDTAAKFIGAGAATVGVAGSGAGIGTVFGSLIIGYARNPSLKQQLFSYAILGFALSEA
MGLFCLMVAFLILFAM
>sp|Q06055|AT5G2_HUMAN ATP synthase F(0) complex subunit C2, mitochondrial OS=Homo sapiens OX=9606 GN=ATP5MC2 PE=1 SV=1
MFACSKFVSTPSLVKSTSQLLSRPLSAVVLKRPEILTDESLSSLAVSCPLTSLVSSRSFQ
TSAISRDIDTAAKFIGAGAATVGVAGSGAGIGTVFGSLIIGYARNPSLKQQLFSYAILGF
ALSEAMGLFCLMVAFLILFAM
>sp|P48201|AT5G3_HUMAN ATP synthase F(0) complex subunit C3, mitochondrial OS=Homo sapiens OX=9606 GN=ATP5MC3 PE=1 SV=1
MFACAKLACTPSLIRAGSRVAYRPISASVLSRPEASRTGEGSTVFNGAQNGVSQLIQREF
QTSAISRDIDTAAKFIGAGAATVGVAGSGAGIGTVFGSLIIGYARNPSLKQQLFSYAILG
FALSEAMGLFCLMVAFLILFAM
>sp|O00299|CLIC1_HUMAN Chloride intracellular channel protein 1 OS=Homo sapiens OX=9606 GN=CLIC1 PE=1 SV=4
MAEEQPQVELFVKAGSDGAKIGNCPFSQRLFMVLWLKGVTFNVTTVDTKRRTETVQKLCP
GGQLPFLLYGTEVHTDTNKIEEFLEAVLCPPRYPKLAALNPESNTAGLDIFAKFSAYIKN
SNPALNDNLEKGLLKALKVLDNYLTSPLPEEVDETSAEDEGVSQRKFLDGNELTLADCNL
LPKLHIVQVVCKKYRGFTIPEAFRGVHRYLSNAYAREEFASTCPDDEEIELAYEQVAKAL
K

>sp|P61626|LYSC_HUMAN Lysozyme C OS=Homo sapiens OX=9606 GN=LYZ PE=1 SV=1
MKALIVLGLVLLSVTVQGKVFERCELARTLKRLGMDGYRGISLANWMCLAKWESGYNTRA
TNYNAGDRSTDYGIFQINSRYWCNDGKTPGAVNACHLSCSALLQDNIADAVACAKRVVRD
PQGIRAWVAWRNRCQNRDVRQYVQGCGV
>sp|P51149|RAB7A_HUMAN Ras-related protein Rab-7a OS=Homo sapiens OX=9606 GN=RAB7A PE=1 SV=1
MTSRKKVLLKVIILGDSGVGKTSLMNQYVNKKFSNQYKATIGADFLTKEVMVDDRLVTMQ
IWDTAGQERFQSLGVAFYRGADCCVLVFDVTAPNTFKTLDSWRDEFLIQASPRDPENFPF
VVLGNKIDLENRQVATKRAQAWCYSKNNIPYFETSAKEAINVEQAFQTIARNALKQETEV
ELYNEFPEPIKLDKNDRAKASAESCSC
>sp|P62820|RAB1A_HUMAN Ras-related protein Rab-1A OS=Homo sapiens OX=9606 GN=RAB1A PE=1 SV=3
MSSMNPEYDYLFKLLLIGDSGVGKSCLLLRFADDTYTESYISTIGVDFKIRTIELDGKTI
KLQIWDTAGQERFRTITSSYYRGAHGIIVVYDVTDQESFNNVKQWLQEIDRYASENVNKL
LVGNKCDLTTKKVVDYTTAKEFADSLGIPFLETSAKNATNVEQSFMTMAAEIKKRMGPGA
TAGGAEKSNVKIQSTPVKQSGGGCC
>sp|Q9H0U4|RAB1B_HUMAN Ras-related protein Rab-1B OS=Homo sapiens OX=9606 GN=RAB1B PE=1 SV=1
MNPEYDYLFKLLLIGDSGVGKSCLLLRFADDTYTESYISTIGVDFKIRTIELDGKTIKLQ
IWDTAGQERFRTITSSYYRGAHGIIVVYDVTDQESYANVKQWLQEIDRYASENVNKLLVG
NKSDLTTKKVVDNTTAKEFADSLGIPFLETSAKNATNVEQAFMTMAAEIKKRMGPGAASG
GERPNLKIDSTPVKPAGGGCC
>sp|Q92928|RAB1C_HUMAN Putative Ras-related protein Rab-1C OS=Homo sapiens OX=9606 GN=RAB1C PE=5 SV=2
MNPGYDCLFKLLLIGDSGVGKSCLLLRFADDPYTESYISTIGVDFKIQTIELDGKTIKLQ
IWDTAGQERFWTITSSYYRGAHGFLVVYDVTDQESYANVKQWLQEIDRHASENVNKLLVG
NKSDLTTKKVVDNTTAKEFADSLGIPFLETSAKNATNVEQAFMTMAAEIKKQMGPGAASG
GERPNLKIDSTPVKPAGGGCC
>sp|Q9HCY8|S10AE_HUMAN Protein S100-A14 OS=Homo sapiens OX=9606 GN=S100A14 PE=1 SV=1
MGQCRSANAEDAQEFSDVERAIETLIKNFHQYSVEGGKETLTPSELRDLVTQQLPHLMPS
NCGLEEKIANLGSCNDSKLEFRSFWELIGEAAKSVKLERPVRGH
>sp|P36578|RL4_HUMAN 60S ribosomal protein L4 OS=Homo sapiens OX=9606 GN=RPL4 PE=1 SV=5
MACARPLISVYSEKGESSGKNVTLPAVFKAPIRPDIVNFVHTNLRKNNRQPYAVSELAGH
QTSAESWGTGRAVARIPRVRGGGTHRSGQGAFGNMCRGGRMFAPTKTWRRWHRRVNTTQK
RYAICSALAASALPALVMSKGHRIEEVPELPLVVEDKVEGYKKTKEAVLLLKKLKAWNDI
KKVYASQRMRAGKGKMRNRRRIQRRGPCIIYNEDNGIIKAFRNIPGITLLNVSKLNILKL
APGGHVGRFCIWTESAFRKLDELYGTWRKAASLKSNYNLPMHKMINTDLSRILKSPEIQR
ALRAPRKKIHRRVLKKNPLKNLRIMLKLNPYAKTMRRNTILRQARNHKLRVDKAAAAAAA
LQAKSDEKAAVAGKKPVVGKKGKKAAVGVKKQKKPLVGKKAAATKKPAPEKKPAEKKPTT
EEKKPAA
>sp|P50395|GDIB_HUMAN Rab GDP dissociation inhibitor beta OS=Homo sapiens OX=9606 GN=GDI2 PE=1 SV=2
MNEEYDVIVLGTGLTECILSGIMSVNGKKVLHMDRNPYYGGESASITPLEDLYKRFKIPG
SPPESMGRGRDWNVDLIPKFLMANGQLVKMLLYTEVTRYLDFKVTEGSFVYKGGKIYKVP

STEAEALASSLMGLFEKRRFRKFLVYVANFDEKDPRTFEGIDPKKTTMRDVYKKFDLGQD
VIDFTGHALALYRTDDYLDQPCYETINRIKLYSESLARYGKSPYLYPLYGLGELPQGFAR
LSAIYGGTYMLNKPIEEIIVQNGKVIGVKSEGEIARCKQLICDPSYVKDRVEKVGQVIRV
ICILSHPIKNTNDANSCQIIIPQNQVNRKSDIYVCMISFAHNVAAQGKYIAIVSTTVETK
EPEKEIRPALELLEPIEQKFVSISDLLVPKDLGTESQIFISRTYDATTHFETTCDDIKNI
YKRMTGSEFDFEEMKRKKNDIYGED
>sp|P31150|GDIA_HUMAN Rab GDP dissociation inhibitor alpha OS=Homo sapiens OX=9606 GN=GDI1 PE=1 SV=2
MDEEYDVIVLGTGLTECILSGIMSVNGKKVLHMDRNPYYGGESSSITPLEELYKRFQLLE
GPPESMGRGRDWNVDLIPKFLMANGQLVKMLLYTEVTRYLDFKVVEGSFVYKGGKIYKVP
STETEALASNLMGMFEKRRFRKFLVFVANFDENDPKTFEGVDPQTTSMRDVYRKFDLGQD
VIDFTGHALALYRTDDYLDQPCLETVNRIKLYSESLARYGKSPYLYPLYGLGELPQGFAR
LSAIYGGTYMLNKPVDDIIMENGKVVGVKSEGEVARCKQLICDPSYIPDRVRKAGQVIRI
ICILSHPIKNTNDANSCQIIIPQNQVNRKSDIYVCMISYAHNVAAQGKYIAIASTTVETT
DPEKEVEPALELLEPIDQKFVAISDLYEPIDDGCESQVFCSCSYDATTHFETTCNDIKDI
YKRMAGTAFDFENMKRKQNDVFGEAEQ
>sp|P23396|RS3_HUMAN 40S ribosomal protein S3 OS=Homo sapiens OX=9606 GN=RPS3 PE=1 SV=2
MAVQISKKRKFVADGIFKAELNEFLTRELAEDGYSGVEVRVTPTRTEIIILATRTQNVLG
EKGRRIRELTAVVQKRFGFPEGSVELYAEKVATRGLCAIAQAESLRYKLLGGLAVRRACY
GVLRFIMESGAKGCEVVVSGKLRGQRAKSMKFVDGLMIHSGDPVNYYVDTAVRHVLLRQG
VLGIKVKIMLPWDPTGKIGPKKPLPDHVSIVEPKDEILPTTPISEQKGGKPEPPAMPQPV
PTA
>sp|Q8IW75|SPA12_HUMAN Serpin A12 OS=Homo sapiens OX=9606 GN=SERPINA12 PE=1 SV=1
MNPTLGLAIFLAVLLTVKGLLKPSFSPRNYKALSEVQGWKQRMAAKELARQNMDLGFKLL
KKLAFYNPGRNIFLSPLSISTAFSMLCLGAQDSTLDEIKQGFNFRKMPEKDLHEGFHYII
HELTQKTQDLKLSIGNTLFIDQRLQPQRKFLEDAKNFYSAETILTNFQNLEMAQKQINDF
ISQKTHGKINNLIENIDPGTVMLLANYIFFRARWKHEFDPNVTKEEDFFLEKNSSVKVPM
MFRSGIYQVGYDDKLSCTILEIPYQKNITAIFILPDEGKLKHLEKGLQVDTFSRWKTLLS
RRVVDVSVPRLHMTGTFDLKKTLSYIGVSKIFEEHGDLTKIAPHRSLKVGEAVHKAELKM
DERGTEGAAGTGAQTLPMETPLVVKIDKPYLLLIYSEKIPSVLFLGKIVNPIGK
>sp|P15311|EZRI_HUMAN Ezrin OS=Homo sapiens OX=9606 GN=EZR PE=1 SV=4
MPKPINVRVTTMDAELEFAIQPNTTGKQLFDQVVKTIGLREVWYFGLHYVDNKGFPTWLK
LDKKVSAQEVRKENPLQFKFRAKFYPEDVAEELIQDITQKLFFLQVKEGILSDEIYCPPE
TAVLLGSYAVQAKFGDYNKEVHKSGYLSSERLIPQRVMDQHKLTRDQWEDRIQVWHAEHR
GMLKDNAMLEYLKIAQDLEMYGINYFEIKNKKGTDLWLGVDALGLNIYEKDDKLTPKIGF
PWSEIRNISFNDKKFVIKPIDKKAPDFVFYAPRLRINKRILQLCMGNHELYMRRRKPDTI
EVQQMKAQAREEKHQKQLERQQLETEKKRRETVEREKEQMMREKEELMLRLQDYEEKTKK
AERELSEQIQRALQLEEERKRAQEEAERLEADRMAALRAKEELERQAVDQIKSQEQLAAE
LAEYTAKIALLEEARRRKEDEVEEWQHRAKEAQDDLVKTKEELHLVMTAPPPPPPPVYEP
VSYHVQESLQDEGAEPTGYSAELSSEGIRDDRNEEKRITEAEKNERVQRQLLTLSSELSQ
ARDENKRTHNDIIHNENMRQGRDKYKTLRQIRQGNTKQRIDEFEAL
>sp|P12081|HARS1_HUMAN Histidine--tRNA ligase, cytoplasmic OS=Homo sapiens OX=9606 GN=HARS1 PE=1 SV=2
MAERAALEELVKLQGERVRGLKQQKASAELIEEEVAKLLKLKAQLGPDESKQKFVLKTPK

GTRDYSPRQMAVREKVFDVIIRCFKRHGAEVIDTPVFELKETLMGKYGEDSKLIYDLKDQ
GGELLSLRYDLTVPFARYLAMNKLTNIKRYHIAKVYRRDNPAMTRGRYREFYQCDFDIAG
NFDPMIPDAECLKIMCEILSSLQIGDFLVKVNDRRILDGMFAICGVSDSKFRTICSSVDK
LDKVSWEEVKNEMVGEKGLAPEVADRIGDYVQQHGGVSLVEQLLQDPKLSQNKQALEGLG
DLKLLFEYLTLFGIDDKISFDLSLARGLDYYTGVIYEAVLLQTPAQAGEEPLGVGSVAAG
GRYDGLVGMFDPKGRKVPCVGLSIGVERIFSIVEQRLEALEEKIRTTETQVLVASAQKKL
LEERLKLVSELWDAGIKAELLYKKNPKLLNQLQYCEEAGIPLVAIIGEQELKDGVIKLRS
VTSREEVDVRREDLVEEIKRRTGQPLCIC
>sp|Q99832|TCPH_HUMAN T-complex protein 1 subunit eta OS=Homo sapiens OX=9606 GN=CCT7 PE=1 SV=2
MMPTPVILLKEGTDSSQGIPQLVSNISACQVIAEAVRTTLGPRGMDKLIVDGRGKATISN
DGATILKLLDVVHPAAKTLVDIAKSQDAEVGDGTTSVTLLAAEFLKQVKPYVEEGLHPQI
IIRAFRTATQLAVNKIKEIAVTVKKADKVEQRKLLEKCAMTALSSKLISQQKAFFAKMVV
DAVMMLDDLLQLKMIGIKKVQGGALEDSQLVAGVAFKKTFSYAGFEMQPKKYHNPKIALL
NVELELKAEKDNAEIRVHTVEDYQAIVDAEWNILYDKLEKIHHSGAKVVLSKLPIGDVAT
QYFADRDMFCAGRVPEEDLKRTMMACGGSIQTSVNALSADVLGRCQVFEETQIGGERYNF
FTGCPKAKTCTFILRGGAEQFMEETERSLHDAIMIVRRAIKNDSVVAGGGAIEMELSKYL
RDYSRTIPGKQQLLIGAYAKALEIIPRQLCDNAGFDATNILNKLRARHAQGGTWYGVDIN
NEDIADNFEAFVWEPAMVRINALTAASEAACLIVSVDETIKNPRSTVDAPTAAGRGRGRG
RPH
>sp|O95147|DUS14_HUMAN Dual specificity protein phosphatase 14 OS=Homo sapiens OX=9606 GN=DUSP14 PE=1 SV=1
MSSRGHSTLPRTLMAPRMISEGDIGGIAQITSSLFLGRGSVASNRHLLQARGITCIVNAT
IEIPNFNWPQFEYVKVPLADMPHAPIGLYFDTVADKIHSVSRKHGATLVHCAAGVSRSAT
LCIAYLMKFHNVCLLEAYNWVKARRPVIRPNVGFWRQLIDYERQLFGKSTVKMVQTPYGI
VPDVYEKESRHLMPYWGI
>sp|Q99878|H2A1J_HUMAN Histone H2A type 1-J OS=Homo sapiens OX=9606 GN=H2AC14 PE=1 SV=3
MSGRGKQGGKARAKAKTRSSRAGLQFPVGRVHRLLRKGNYAERVGAGAPVYLAAVLEYLT
AEILELAGNAARDNKKTRIIPRHLQLAIRNDEELNKLLGKVTIAQGGVLPNIQAVLLPKK
TESHHKTK
>sp|Q16777|H2A2C_HUMAN Histone H2A type 2-C OS=Homo sapiens OX=9606 GN=H2AC20 PE=1 SV=4
MSGRGKQGGKARAKAKSRSSRAGLQFPVGRVHRLLRKGNYAERVGAGAPVYMAAVLEYLT
AEILELAGNAARDNKKTRIIPRHLQLAIRNDEELNKLLGKVTIAQGGVLPNIQAVLLPKK
TESHKAKSK
>sp|Q6FI13|H2A2A_HUMAN Histone H2A type 2-A OS=Homo sapiens OX=9606 GN=H2AC19 PE=1 SV=3
MSGRGKQGGKARAKAKSRSSRAGLQFPVGRVHRLLRKGNYAERVGAGAPVYMAAVLEYLT
AEILELAGNAARDNKKTRIIPRHLQLAIRNDEELNKLLGKVTIAQGGVLPNIQAVLLPKK
TESHHKAKGK
>sp|Q96KK5|H2A1H_HUMAN Histone H2A type 1-H OS=Homo sapiens OX=9606 GN=H2AC12 PE=1 SV=3
MSGRGKQGGKARAKAKTRSSRAGLQFPVGRVHRLLRKGNYAERVGAGAPVYLAAVLEYLT
AEILELAGNAARDNKKTRIIPRHLQLAIRNDEELNKLLGKVTIAQGGVLPNIQAVLLPKK
TESHHKAK

>sp|Q9BTM1|H2AJ_HUMAN Histone H2A.J OS=Homo sapiens OX=9606 GN=H2AJ PE=1 SV=1
MSGRGKQGGKVRAKAKSRSSRAGLQFPVGRVHRLLRKGNYAERVGAGAPVYLAAVLEYLT
AEILELAGNAARDNKKTRIIPRHLQLAIRNDEELNKLLGKVTIAQGGVLPNIQAVLLPKK
TESQKTKSK
>sp|P0C0S8|H2A1_HUMAN Histone H2A type 1 OS=Homo sapiens OX=9606 GN=H2AC17 PE=1 SV=2
MSGRGKQGGKARAKAKTRSSRAGLQFPVGRVHRLLRKGNYAERVGAGAPVYLAAVLEYLT
AEILELAGNAARDNKKTRIIPRHLQLAIRNDEELNKLLGKVTIAQGGVLPNIQAVLLPKK
TESHHKAKGK
>sp|P20671|H2A1D_HUMAN Histone H2A type 1-D OS=Homo sapiens OX=9606 GN=H2AC7 PE=1 SV=2
MSGRGKQGGKARAKAKTRSSRAGLQFPVGRVHRLLRKGNYSERVGAGAPVYLAAVLEYLT
AEILELAGNAARDNKKTRIIPRHLQLAIRNDEELNKLLGKVTIAQGGVLPNIQAVLLPKK
TESHHKAKGK
>sp|P16104|H2AX_HUMAN Histone H2AX OS=Homo sapiens OX=9606 GN=H2AX PE=1 SV=2
MSGRGKTGGKARAKAKSRSSRAGLQFPVGRVHRLLRKGHYAERVGAGAPVYLAAVLEYLT
AEILELAGNAARDNKKTRIIPRHLQLAIRNDEELNKLLGGVTIAQGGVLPNIQAVLLPKK
TSATVGPKAPSGGKKATQASQEY
>sp|Q96QV6|H2A1A_HUMAN Histone H2A type 1-A OS=Homo sapiens OX=9606 GN=H2AC1 PE=1 SV=3
MSGRGKQGGKARAKSKSRSSRAGLQFPVGRIHRLLRKGNYAERIGAGAPVYLAAVLEYLT
AEILELAGNASRDNKKTRIIPRHLQLAIRNDEELNKLLGGVTIAQGGVLPNIQAVLLPKK
TESHHHKAQSK
>sp|Q93077|H2A1C_HUMAN Histone H2A type 1-C OS=Homo sapiens OX=9606 GN=H2AC6 PE=1 SV=3
MSGRGKQGGKARAKAKSRSSRAGLQFPVGRVHRLLRKGNYAERVGAGAPVYLAAVLEYLT
AEILELAGNAARDNKKTRIIPRHLQLAIRNDEELNKLLGRVTIAQGGVLPNIQAVLLPKK
TESHHKAKGK
>sp|Q7L7L0|H2A3_HUMAN Histone H2A type 3 OS=Homo sapiens OX=9606 GN=H2AW PE=1 SV=3
MSGRGKQGGKARAKAKSRSSRAGLQFPVGRVHRLLRKGNYSERVGAGAPVYLAAVLEYLT
AEILELAGNAARDNKKTRIIPRHLQLAIRNDEELNKLLGRVTIAQGGVLPNIQAVLLPKK
TESHHKAKGK
>sp|P04908|H2A1B_HUMAN Histone H2A type 1-B/E OS=Homo sapiens OX=9606 GN=H2AC8 PE=1 SV=2
MSGRGKQGGKARAKAKTRSSRAGLQFPVGRVHRLLRKGNYSERVGAGAPVYLAAVLEYLT
AEILELAGNAARDNKKTRIIPRHLQLAIRNDEELNKLLGRVTIAQGGVLPNIQAVLLPKK
TESHHKAKGK
>sp|P09525|ANXA4_HUMAN Annexin A4 OS=Homo sapiens OX=9606 GN=ANXA4 PE=1 SV=4
MATKGGTVKAASGFNAMEDAQTLRKAMKGLGTDEDAIISVLAYRNTAQRQEIRTAYKSTI
GRDLIDDLKSELSGNFEQVIVGMMTPTVLYDVQELRRAMKGAGTDEGCLIEILASRTPEE
IRRISQTYQQQYGRSLEDDIRSDTSFMFQRVLVSLSAGGRDEGNYLDDALVRQDAQDLYE
AGEKKWGTDEVKFLTVLCSRNRNHLLHVFDEYKRISQKDIEQSIKSETSGSFEDALLAIV
KCMRNKSAYFAEKLYKSMKGLGTDDNTLIRVMVSRAEIDMLDIRAHFKRLYGKSLYSFIK

GDTSGDYRKVLLVLCGGDD
>sp|P61981|1433G_HUMAN 14-3-3 protein gamma OS=Homo sapiens OX=9606 GN=YWHAG PE=1 SV=2
MVDREQLVQKARLAEQAERYDDMAAAMKNVTELNEPLSNEERNLLSVAYKNVVGARRSSW
RVISSIEQKTSADGNEKKIEMVRAYREKIEKELEAVCQDVLSLLDNYLIKNCSETQYESK
VFYLKMKGDYYRYLAEVATGEKRATVVESSEKAYSEAHEISKEHMQPTHPIRLGLALNYS
VFYYEIQNAPEQACHLAKTAFDDAIAELDTLNEDSYKDSTLIMQLLRDNLTLWTSDQQDD
DGGEGNN
>sp|Q5VT79|AXA81_HUMAN Annexin A8-like protein 1 OS=Homo sapiens OX=9606 GN=ANXA8L1 PE=2 SV=2
MAWWKAWIEQEGVTVKSSSHFNPDPDAETLYKAMKGIGTNEQAIIDVLTKRSNTQRQQIA
KSFKAQFGKDLTETLKSELSGKFERLIVALMYPPYRYEAKELHDAMKGLGTKEGVIIEIL
ASRTKNQLREIMKAYEEDYGSSLEEDIQADTSGYLERILVCLLQGSRDDVSSFVDPALAL
QDAQDLYAAGEKIRGTDEMKFITILCTRSATHLLRVFEEYEKIANKSIEDSIKSETHGSL
EEAMLTVVKCTQNLHSYFAERLYYAMKGAGTRDGTLIRNIVSRSEIDLNLIKCHFKKMYG
KTLSSMIMEDTSGDYKNALLSLVGSDP
>sp|P13928|ANXA8_HUMAN Annexin A8 OS=Homo sapiens OX=9606 GN=ANXA8 PE=1 SV=3
MAWWKSWIEQEGVTVKSSSHFNPDPDAETLYKAMKGIGTNEQAIIDVLTKRSNTQRQQIA
KSFKAQFGKDLTETLKSELSGKFERLIVALMYPPYRYEAKELHDAMKGLGTKEGVIIEIL
ASRTKNQLREIMKAYEEDYGSSLEEDIQADTSGYLERILVCLLQGSRDDVSSFVDPGLAL
QDAQDLYAAGEKIRGTDEMKFITILCTRSATHLLRVFEEYEKIANKSIEDSIKSETHGSL
EEAMLTVVKCTQNLHSYFAERLYYAMKGAGTRDGTLIRNIVSRSEIDLNLIKCHFKKMYG
KTLSSMIMEDTSGDYKNALLSLVGSDP
>sp|Q3LI77|KR134_HUMAN Keratin-associated protein 13-4 OS=Homo sapiens OX=9606 GN=KRTAP13-4 PE=1 SV=1
MSYNCCSRNFSSRSFGGYLYYPGSYPSSLVYSTALCSPSTCQLRSSLYRDCQKTCWEPAS
CQKSCYRPRTSILCCPCQTTCSGSLGFRSSSCRSQGYGSRCCYSLGNGSSGFRFLKYGGC
GFPSLSYGSRFCYPNYLASGAWQSSCYRPICGSRFYQFTC
>sp|P18206|VINC_HUMAN Vinculin OS=Homo sapiens OX=9606 GN=VCL PE=1 SV=4
MPVFHTRTIESILEPVAQQISHLVIMHEEGEVDGKAIPDLTAPVAAVQAAVSNLVRVGKE
TVQTTEDQILKRDMPPAFIKVENACTKLVQAAQMLQSDPYSVPARDYLIDGSRGILSGTS
DLLLTFDEAEVRKIIRVCKGILEYLTVAEVVETMEDLVTYTKNLGPGMTKMAKMIDERQQ
ELTHQEHRVMLVNSMNTVKELLPVLISAMKIFVTTKNSKNQGIEEALKNRNFTVEKMSAE
INEIIRVLQLTSWDEDAWASKDTEAMKRALASIDSKLNQAKGWLRDPSASPGDAGEQAIR
QILDEAGKVGELCAGKERREILGTCKMLGQMTDQVADLRARGQGSSPVAMQKAQQVSQGL
DVLTAKVENAARKLEAMTNSKQSIAKKIDAAQNWLADPNGGPEGEEQIRGALAEARKIAE
LCDDPKERDDILRSLGEISALTSKLADLRRQGKGDSPEARALAKQVATALQNLQTKTNRA
VANSRPAKAAVHLEGKIEQAQRWIDNPTVDDRGVGQAAIRGLVAEGHRLANVMMGPYRQD
LLAKCDRVDQLTAQLADLAARGEGESPQARALASQLQDSLKDLKARMQEAMTQEVSDVFS
DTTTPIKLLAVAATAPPDAPNREEVFDERAANFENHSGKLGATAEKAAAVGTANKSTVEG
IQASVKTARELTPQVVSAARILLRNPGNQAAYEHFETMKNQWIDNVEKMTGLVDEAIDTK
SLLDASEEAIKKDLDKCKVAMANIQPQMLVAGATSIARRANRILLVAKREVENSEDPKFR
EAVKAASDELSKTISPMVMDAKAVAGNISDPGLQKSFLDSGYRILGAVAKVREAFQPQEP
DFPPPPPDLEQLRLTDELAPPKPPLPEGEVPPPRPPPPEEKDEEFPEQKAGEVINQPMMM
AARQLHDEARKWSSKPGIPAAEVGIGVVAEADAADAAGFPVPPDMEDDYEPELLLMPSNQ

PVNQPILAAAQSLHREATKWSSKGNDIIAAAKRMALLMAEMSRLVRGGSGTKRALIQCAK
DIAKASDEVTRLAKEVAKQCTDKRIRTNLLQVCERIPTISTQLKILSTVKATMLGRTNIS
DEESEQATEMLVHNAQNLMQSVKETVREAEAASIKIRTDAGFTLRWVRKTPWYQ
>sp|P78371|TCPB_HUMAN T-complex protein 1 subunit beta OS=Homo sapiens OX=9606 GN=CCT2 PE=1 SV=4
MASLSLAPVNIFKAGADEERAETARLTSFIGAIAIGDLVKSTLGPKGMDKILLSSGRDAS
LMVTNDGATILKNIGVDNPAAKVLVDMSRVQDDEVGDGTTSVTVLAAELLREAESLIAKK
IHPQTIIAGWREATKAAREALLSSAVDHGSDEVKFRQDLMNIAGTTLSSKLLTHHKDHFT
KLAVEAVLRLKGSGNLEAIHIIKKLGGSLADSYLDEGFLLDKKIGVNQPKRIENAKILIA
NTGMDTDKIKIFGSRVRVDSTAKVAEIEHAEKEKMKEKVERILKHGINCFINRQLIYNYP
EQLFGAAGVMAIEHADFAGVERLALVTGGEIASTFDHPELVKLGSCKLIEEVMIGEDKLI
HFSGVALGEACTIVLRGATQQILDEAERSLHDALCVLAQTVKDSRTVYGGGCSEMLMAHA
VTQLANRTPGKEAVAMESYAKALRMLPTIIADNAGYDSADLVAQLRAAHSEGNTTAGLDM
REGTIGDMAILGITESFQVKRQVLLSAAEAAEVILRVDNIIKAAPRKRVPDHHPC
>sp|P27348|1433T_HUMAN 14-3-3 protein theta OS=Homo sapiens OX=9606 GN=YWHAQ PE=1 SV=1
MEKTELIQKAKLAEQAERYDDMATCMKAVTEQGAELSNEERNLLSVAYKNVVGGRRSAWR
VISSIEQKTDTSDKKLQLIKDYREKVESELRSICTTVLELLDKYLIANATNPESKVFYLK
MKGDYFRYLAEVACGDDRKQTIDNSQGAYQEAFDISKKEMQPTHPIRLGLALNFSVFYYE
ILNNPELACTLAKTAFDEAIAELDTLNEDSYKDSTLIMQLLRDNLTLWTSDSAGEECDAA
EGAEN
>sp|P05109|S10A8_HUMAN Protein S100-A8 OS=Homo sapiens OX=9606 GN=S100A8 PE=1 SV=1
MLTELEKALNSIIDVYHKYSLIKGNFHAVYRDDLKKLLETECPQYIRKKGADVWFKELDI
NTDGAVNFQEFLILVIKMGVAAHKKSHEESHKE
>sp|P09651|ROA1_HUMAN Heterogeneous nuclear ribonucleoprotein A1 OS=Homo sapiens OX=9606 GN=HNRNPA1 PE=1 SV=5
MSKSESPKEPEQLRKLFIGGLSFETTDESLRSHFEQWGTLTDCVVMRDPNTKRSRGFGFV
TYATVEEVDAAMNARPHKVDGRVVEPKRAVSREDSQRPGAHLTVKKIFVGGIKEDTEEHH
LRDYFEQYGKIEVIEIMTDRGSGKKRGFAFVTFDDHDSVDKIVIQKYHTVNGHNCEVRKA
LSKQEMASASSSQRGRSGSGNFGGGRGGGFGGNDNFGRGGNFSGRGGFGGSRGGGGYGGS
GDGYNGFGNDGGYGGGGPGYSGGSRGYGSGGQGYGNQGSGYGGSGSYDSYNNGGGGGFGG
GSGSNFGGGGSYNDFGNYNNQSSNFGPMKGGNFGGRSSGPYGGGGQYFAKPRNQGGYGGS
SSSSSYGSGRRF
>sp|Q32P51|RA1L2_HUMAN Heterogeneous nuclear ribonucleoprotein A1-like 2 OS=Homo sapiens OX=9606 GN=HNRNPA1L2 PE=2 SV=2
MSKSASPKEPEQLRKLFIGGLSFETTDESLRSHFEQWGTLTDCVVMRDPNTKRSRGFGFV
TYATVEEVDAAMNTTPHKVDGRVVEPKRAVSREDSQRPGAHLTVKKIFVGGIKEDTEEHH
LRDYFEQYGKIEVIEIMTDRGSGKKRGFAFVTFDDHDSVDKIVIQKYHTVKGHNCEVRKA
LPKQEMASASSSQRGRRGSGNFGGGRGDGFGGNDNFGRGGNFSGRGGFGGSCGGGGYGGS
GDGYNGFGNDGSNFGGGGSYNDFGNYNNQSSNFGPMKGGNFGGRSSGPYGGGGQYFAKPQ
NQGGYGVSSSSSSYGSGRRF
>sp|P62081|RS7_HUMAN 40S ribosomal protein S7 OS=Homo sapiens OX=9606 GN=RPS7 PE=1 SV=1
MFSSSAKIVKPNGEKPDEFESGISQALLELEMNSDLKAQLRELNITAAKEIEVGGGRKAI
IIFVPVPQLKSFQKIQVRLVRELEKKFSGKHVVFIAQRRILPKPTRKSRTKNKQKRPRSR
TLTAVHDAILEDLVFPSEIVGKRIRVKLDGSRLIKVHLDKAQQNNVEHKVETFSGVYKKL

TGKDVNFEFPEFQL
>sp|P0DP24|CALM2_HUMAN Calmodulin-2 OS=Homo sapiens OX=9606 GN=CALM2 PE=1 SV=1
MADQLTEEQIAEFKEAFSLFDKDGDGTITTKELGTVMRSLGQNPTEAELQDMINEVDADG
NGTIDFPEFLTMMARKMKDTDSEEEIREAFRVFDKDGNGYISAAELRHVMTNLGEKLTDE
EVDEMIREADIDGDGQVNYEEFVQMMTAK
>sp|P0DP23|CALM1_HUMAN Calmodulin-1 OS=Homo sapiens OX=9606 GN=CALM1 PE=1 SV=1
MADQLTEEQIAEFKEAFSLFDKDGDGTITTKELGTVMRSLGQNPTEAELQDMINEVDADG
NGTIDFPEFLTMMARKMKDTDSEEEIREAFRVFDKDGNGYISAAELRHVMTNLGEKLTDE
EVDEMIREADIDGDGQVNYEEFVQMMTAK
>sp|P0DP25|CALM3_HUMAN Calmodulin-3 OS=Homo sapiens OX=9606 GN=CALM3 PE=1 SV=1
MADQLTEEQIAEFKEAFSLFDKDGDGTITTKELGTVMRSLGQNPTEAELQDMINEVDADG
NGTIDFPEFLTMMARKMKDTDSEEEIREAFRVFDKDGNGYISAAELRHVMTNLGEKLTDE
EVDEMIREADIDGDGQVNYEEFVQMMTAK
>sp|P63167|DYL1_HUMAN Dynein light chain 1, cytoplasmic OS=Homo sapiens OX=9606 GN=DYNLL1 PE=1 SV=1
MCDRKAVIKNADMSEEMQQDSVECATQALEKYNIEKDIAAHIKKEFDKKYNPTWHCIVGR
NFGSYVTHETKHFIYFYLGQVAILLFKSG
>sp|Q96FJ2|DYL2_HUMAN Dynein light chain 2, cytoplasmic OS=Homo sapiens OX=9606 GN=DYNLL2 PE=1 SV=1
MSDRKAVIKNADMSEDMQQDAVDCATQAMEKYNIEKDIAAYIKKEFDKKYNPTWHCIVGR
NFGSYVTHETKHFIYFYLGQVAILLFKSG
>sp|P40227|TCPZ_HUMAN T-complex protein 1 subunit zeta OS=Homo sapiens OX=9606 GN=CCT6A PE=1 SV=3
MAAVKTLNPKAEVARAQAALAVNISAARGLQDVLRTNLGPKGTMKMLVSGAGDIKLTKDG
NVLLHEMQIQHPTASLIAKVATAQDDITGDGTTSNVLIIGELLKQADLYISEGLHPRIIT
EGFEAAKEKALQFLEEVKVSREMDRETLIDVARTSLRTKVHAELADVLTEAVVDSILAIK
KQDEPIDLFMIEIMEMKHKSETDTSLIRGLVLDHGARHPDMKKRVEDAYILTCNVSLEYE
KTEVNSGFFYKSAEEREKLVKAERKFIEDRVKKIIELKRKVCGDSDKGFVVINQKGIDPF
SLDALSKEGIVALRRAKRRNMERLTLACGGVALNSFDDLSPDCLGHAGLVYEYTLGEEKF
TFIEKCNNPRSVTLLIKGPNKHTLTQIKDAVRDGLRAVKNAIDDGCVVPGAGAVEVAMAE
ALIKHKPSVKGRAQLGVQAFADALLIIPKVLAQNSGFDLQETLVKIQAEHSESGQLVGVD
LNTGEPMVAAEVGVWDNYCVKKQLLHSCTVIATNILLVDEIMRAGMSSLKG
>sp|O95833|CLIC3_HUMAN Chloride intracellular channel protein 3 OS=Homo sapiens OX=9606 GN=CLIC3 PE=1 SV=2
MAETKLQLFVKASEDGESVGHCPSCQRLFMVLLLKGVPFTLTTVDTRRSPDVLKDFAPGS
QLPILLYDSDAKTDTLQIEDFLEETLGPPDFPSLAPRYRESNTAGNDVFHKFSAFIKNPV
PAQDEALYQQLLRALARLDSYLRAPLEHELAGEPQLRESRRRFLDGDRLTLADCSLLPKL
HIVDTVCAHFRQAPIPAELRGVRRYLDSAMQEKEFKYTCPHSAEILAAYRPAVHPR
>sp|P60953|CDC42_HUMAN Cell division control protein 42 homolog OS=Homo sapiens OX=9606 GN=CDC42 PE=1 SV=2
MQTIKCVVVGDGAVGKTCLLISYTTNKFPSEYVPTVFDNYAVTVMIGGEPYTLGLFDTAG
QEDYDRLRPLSYPQTDVFLVCFSVVSPSSFENVKEKWVPEITHHCPKTPFLLVGTQIDLR
DDPSTIEKLAKNKQKPITPETAEKLARDLKAVKYVECSALTQKGLKNVFDEAILAALEPP
EPKKSRRCVLL

>sp|P06744|G6PI_HUMAN Glucose-6-phosphate isomerase OS=Homo sapiens OX=9606 GN=GPI PE=1 SV=4
MAALTRDPQFQKLQQWYREHRSELNLRRLFDANKDRFNHFSLTLNTNHGHILVDYSKNLV
TEDVMRMLVDLAKSRGVEAARERMFNGEKINYTEGRAVLHVALRNRSNTPILVDGKDVMP
EVNKVLDKMKSFCQRVRSGDWKGYTGKTITDVINIGIGGSDLGPLMVTEALKPYSSGGPR
VWYVSNIDGTHIAKTLAQLNPESSLFIIASKTFTTQETITNAETAKEWFLQAAKDPSAVA
KHFVALSTNTTKVKEFGIDPQNMFEFWDWVGGRYSLWSAIGLSIALHVGFDNFEQLLSGA
HWMDQHFRTTPLEKNAPVLLALLGIWYINCFGCETHAMLPYDQYLHRFAAYFQQGDMESN
GKYITKSGTRVDHQTGPIVWGEPGTNGQHAFYQLIHQGTKMIPCDFLIPVQTQHPIRKGL
HHKILLANFLAQTEALMRGKSTEEARKELQAAGKSPEDLERLLPHKVFEGNRPTNSIVFT
KLTPFMLGALVAMYEHKIFVQGIIWDINSFDQWGVELGKQLAKKIEPELDGSAQVTSHDA
STNGLINFIKQQREARVQ
>sp|Q5T750|XP32_HUMAN Skin-specific protein 32 OS=Homo sapiens OX=9606 GN=XP32 PE=1 SV=1
MCDQQKQPQFPPSCVKGSGLGAGQGSNGASVKCPVPCQTQTVCVTGPAPCPTQTYVKYQV
PCQTQTYVKCPAPCQRTYVKYPTPCQTYVKCPAPCQTTYVKCPTPCQTYVKCPAPCQMTY
IKSPAPCQTQTCYVQGASPCQSYYVQAPASGSTSQYCVTDPCSAPCSTSYCCLAPRTFGV
SPLRRWIQRPQNCNTGSSGCCENSGSSGCCGSGGCGCSCGCGSSGCCCLGIIPMRSRGPA
CCDHEDDCCC
>sp|P61163|ACTZ_HUMAN Alpha-centractin OS=Homo sapiens OX=9606 GN=ACTR1A PE=1 SV=1
MESYDVIANQPVVIDNGSGVIKAGFAGDQIPKYCFPNYVGRPKHVRVMAGALEGDIFIGP
KAEEHRGLLSIRYPMEHGIVKDWNDMERIWQYVYSKDQLQTFSEEHPVLLTEAPLNPRKN
RERAAEVFFETFNVPALFISMQAVLSLYATGRTTGVVLDSGDGVTHAVPIYEGFAMPHSI
MRIDIAGRDVSRFLRLYLRKEGYDFHSSSEFEIVKAIKERACYLSINPQKDETLETEKAQ
YYLPDGSTIEIGPSRFRAPELLFRPDLIGEESEGIHEVLVFAIQKSDMDLRRTLFSNIVL
SGGSTLFKGFGDRLLSEVKKLAPKDVKIRISAPQERLYSTWIGGSILASLDTFKKMWVSK
KEYEEDGARSIHRKTF
>sp|P42025|ACTY_HUMAN Beta-centractin OS=Homo sapiens OX=9606 GN=ACTR1B PE=1 SV=1
MESYDIIANQPVVIDNGSGVIKAGFAGDQIPKYCFPNYVGRPKHMRVMAGALEGDLFIGP
KAEEHRGLLTIRYPMEHGVVRDWNDMERIWQYVYSKDQLQTFSEEHPVLLTEAPLNPSKN
REKAAEVFFETFNVPALFISMQAVLSLYATGRTTGVVLDSGDGVTHAVPIYEGFAMPHSI
MRVDIAGRDVSRYLRLLLRKEGVDFHTSAEFEVVRTIKERACYLSINPQKDEALETEKVQ
YTLPDGSTLDVGPARFRAPELLFQPDLVGDESEGLHEVVAFAIHKSDMDLRRTLFANIVL
SGGSTLFKGFGDRLLSEVKKLAPKDIKIKISAPQERLYSTWIGGSILASLDTFKKMWVSK
KEYEEDGSRAIHRKTF
>sp|P07384|CAN1_HUMAN Calpain-1 catalytic subunit OS=Homo sapiens OX=9606 GN=CAPN1 PE=1 SV=1
MSEEIITPVYCTGVSAQVQKQRARELGLGRHENAIKYLGQDYEQLRVRCLQSGTLFRDEA
FPPVPQSLGYKDLGPNSSKTYGIKWKRPTELLSNPQFIVDGATRTDICQGALGDCWLLAA
IASLTLNDTLLHRVVPHGQSFQNGYAGIFHFQLWQFGEWVDVVVDDLLPIKDGKLVFVHS
AEGNEFWSALLEKAYAKVNGSYEALSGGSTSEGFEDFTGGVTEWYELRKAPSDLYQIILK
ALERGSLLGCSIDISSVLDMEAITFKKLVKGHAYSVTGAKQVNYRGQVVSLIRMRNPWGE
VEWTGAWSDSSSEWNNVDPYERDQLRVKMEDGEFWMSFRDFMREFTRLEICNLTPDALKS
RTIRKWNTTLYEGTWRRGSTAGGCRNYPATFWVNPQFKIRLDETDDPDDYGDRESGCSFV
LALMQKHRRRERRFGRDMETIGFAVYEVPPELVGQPAVHLKRDFFLANASRARSEQFINL

REVSTRFRLPPGEYVVVPSTFEPNKEGDFVLRFFSEKSAGTVELDDQIQANLPDEQVLSE
EEIDENFKALFRQLAGEDMEISVKELRTILNRIISKHKDLRTKGFSLESCRSMVNLMDRD
GNGKLGLVEFNILWNRIRNYLSIFRKFDLDKSGSMSAYEMRMAIESAGFKLNKKLYELII
TRYSEPDLAVDFDNFVCCLVRLETMFRFFKTLDTDLDGVVTFDLFKWLQLTMFA
>sp|P06576|ATPB_HUMAN ATP synthase subunit beta, mitochondrial OS=Homo sapiens OX=9606 GN=ATP5F1B PE=1 SV=3
MLGFVGRVAAAPASGALRRLTPSASLPPAQLLLRAAPTAVHPVRDYAAQTSPSPKAGAAT
GRIVAVIGAVVDVQFDEGLPPILNALEVQGRETRLVLEVAQHLGESTVRTIAMDGTEGLV
RGQKVLDSGAPIKIPVGPETLGRIMNVIGEPIDERGPIKTKQFAPIHAEAPEFMEMSVEQ
EILVTGIKVVDLLAPYAKGGKIGLFGGAGVGKTVLIMELINNVAKAHGGYSVFAGVGERT
REGNDLYHEMIESGVINLKDATSKVALVYGQMNEPPGARARVALTGLTVAEYFRDQEGQD
VLLFIDNIFRFTQAGSEVSALLGRIPSAVGYQPTLATDMGTMQERITTTKKGSITSVQAI
YVPADDLTDPAPATTFAHLDATTVLSRAIAELGIYPAVDPLDSTSRIMDPNIVGSEHYDV
ARGVQKILQDYKSLQDIIAILGMDELSEEDKLTVSRARKIQRFLSQPFQVAEVFTGHMGK
LVPLKETIKGFQQILAGEYDHLPEQAFYMVGPIEEAVAKADKLAEEHSS
>sp|P23490|LORI_HUMAN Loricrin OS=Homo sapiens OX=9606 GN=LORICRIN PE=1 SV=2
MSYQKKQPTPQPPVDCVKTSGGGGGGGGSGGGGCGFFGGGGSGGGSSGSGCGYSGGGGYS
GGGCGGGSSGGGGGGGIGGCGGGSGGSVKYSGGGGSSGGGSGCFSSGGGGSGCFSSGGGG
SSGGGSGCFSSGGGGSSGGGSGCFSSGGGGFSGQAVQCQSYGGVSSGGSSGGGSGCFSSG
GGGGSVCGYSGGGSGCGGGSSGGSGSGYVSSQQVTQTSCAPQPSYGGGSSGGGGSGGSGC
FSSGGGGGSSGCGGGSSGIGSGCIISGGGSVCGGGSSGGGGGGSSVGGSGSGKGVPICHQ
TQQKQAPTWPSK
>sp|P62244|RS15A_HUMAN 40S ribosomal protein S15a OS=Homo sapiens OX=9606 GN=RPS15A PE=1 SV=2
MVRMNVLADALKSINNAEKRGKRQVLIRPCSKVIVRFLTVMMKHGYIGEFEIIDDHRAGK
IVVNLTGRLNKCGVISPRFDVQLKDLEKWQNNLLPSRQFGFIVLTTSAGIMDHEEARRKH
TGGKILGFFF
>sp|P30101|PDIA3_HUMAN Protein disulfide-isomerase A3 OS=Homo sapiens OX=9606 GN=PDIA3 PE=1 SV=4
MRLRRLALFPGVALLLAAARLAAASDVLELTDDNFESRISDTGSAGLMLVEFFAPWCGHC
KRLAPEYEAAATRLKGIVPLAKVDCTANTNTCNKYGVSGYPTLKIFRDGEEAGAYDGPRT
ADGIVSHLKKQAGPASVPLRTEEEFKKFISDKDASIVGFFDDSFSEAHSEFLKAASNLRD
NYRFAHTNVESLVNEYDDNGEGIIILFRPSHLTNKFEDKTVAYTEQKMTSGKIKKFIQENI
FGICPHMTEDNKDLIQGKDLLIAYYDVDYEKNAKGSNYWRNRVMMVAKKFLDAGHKLNFA
VASRKTFSHELSDFGLESTAGEIPVVAIRTAKGEKFVMQEEFSRDGKALERFLQDYFDGN
LKRYLKSEPIPESNDGPVKVVVAENFDEIVNNENKDVLIEFYAPWCGHCKNLEPKYKELG
EKLSKDPNIVIAKMDATANDVPSPYEVRGFPTIYFSPANKKLNPKKYEGGRELSDFISYL
QREATNPPVIQEEKPKKKKKAQEDL
>sp|O76014|KRT37_HUMAN Keratin, type I cuticular Ha7 OS=Homo sapiens OX=9606 GN=KRT37 PE=1 SV=3
MTSFYSTSSCPLGCTMAPGARNVFVSPIDVGCQPVAEANAASMCLLANVAHANRVRVGST
PLGRPSLCLPPTSHTACPLPGTCHIPGNIGICGAYGKNTLNGHEKETMKFLNDRLANYLE
KVRQLEQENAELETTLLERSKCHESTVCPDYQSYFRTIEELQQKILCSKAENARLIVQID
NAKLAADDFRIKLESERSLHQLVEADKCGTQKLLDDATLAKADLEAQQESLKEEQLSLKS
NHEQEVKILRSQLGEKFRIELDIEPTIDLNRVLGEMRAQYEAMVETNHQDVEQWFQAQSE
GISLQAMSCSEELQCCQSEILELRCTVNALEVERQAQHTLKDCLQNSLCEAEDRYGTELA

QMQSLISNLEEQLSEIRADLERQNQEYQVLLDVKARLENEIATYRNLLESEDCKLPCNPC
STPASCTSCPSCGPVTGGSPSGHGASMGR
>sp|Q7Z406|MYH14_HUMAN Myosin-14 OS=Homo sapiens OX=9606 GN=MYH14 PE=1 SV=2
MAAVTMSVPGRKAPPRPGPVPEAAQPFLFTPRGPSAGGGPGSGTSPQVEWTARRLVWVPS
ELHGFEAAALRDEGEEEAEVELAESGRRLRLPRDQIQRMNPPKFSKAEDMAELTCLNEAS
VLHNLRERYYSGLIYTYSGLFCVVINPYKQLPIYTEAIVEMYRGKKRHEVPPHVYAVTEG
AYRSMLQDREDQSILCTGESGAGKTENTKKVIQYLAHVASSPKGRKEPGVPGELERQLLQ
ANPILEAFGNAKTVKNDNSSRFGKFIRINFDVAGYIVGANIETYLLEKSRAIRQAKDECS
FHIFYQLLGGAGEQLKADLLLEPCSHYRFLTNGPSSSPGQERELFQETLESLRVLGFSHE
EIISMLRMVSAVLQFGNIALKRERNTDQATMPDNTAAQKLCRLLGLGVTDFSRALLTPRI
KVGRDYVQKAQTKEQADFALEALAKATYERLFRWLVLRLNRALDRSPRQGASFLGILDIA
GFEIFQLNSFEQLCINYTNEKLQQLFNHTMFVLEQEEYQREGIPWTFLDFGLDLQPCIDL
IERPANPPGLLALLDEECWFPKATDKSFVEKVAQEQGGHPKFQRPRHLRDQADFSVLHYA
GKVDYKANEWLMKNMDPLNDNVAALLHQSTDRLTAEIWKDVEGIVGLEQVSSLGDGPPGG
RPRRGMFRTVGQLYKESLSRLMATLSNTNPSFVRCIVPNHEKRAGKLEPRLVLDQLRCNG
VLEGIRICRQGFPNRILFQEFRQRYEILTPNAIPKGFMDGKQACEKMIQALELDPNLYRV
GQSKIFFRAGVLAQLEEERDLKVTDIIVSFQAAARGYLARRAFQKRQQQQSALRVMQRNC
AAYLKLRHWQWWRLFTKVKPLLQVTRQDEVLQARAQELQKVQELQQQSAREVGELQGRVA
QLEEERARLAEQLRAEAELCAEAEETRGRLAARKQELELVVSELEARVGEEEECSRQMQT
EKKRLQQHIQELEAHLEAEEGARQKLQLEKVTTEAKMKKFEEDLLLLEDQNSKLSKERKL
LEDRLAEFSSQAAEEEEKVKSLNKLRLKYEATIADMEDRLRKEEKGRQELEKLKRRLDGE
SSELQEQMVEQQQRAEELRAQLGRKEEELQAALARAEDEGGARAQLLKSLREAQAALAEA
QEDLESERVARTKAEKQRRDLGEELEALRGELEDTLDSTNAQQELRSKREQEVTELKKTL
EEETRIHEAAVQELRQRHGQALGELAEQLEQARRGKGAWEKTRLALEAEVSELRAELSSL
QTARQEGEQRRRRLELQLQEVQGRAGDGERARAEAAEKLQRAQAELENVSGALNEAESKT
IRLSKELSSTEAQLHDAQELLQEETRAKLALGSRVRAMEAEAAGLREQLEEEAAARERAG
RELQTAQAQLSEWRRRQEEEAGALEAGEEARRRAAREAEALTQRLAEKTETVDRLERGRR
RLQQELDDATMDLEQQRQLVSTLEKKQRKFDQLLAEEKAAVLRAVEERERAEAEGREREA
RALSLTRALEEEQEAREELERQNRALRAELEALLSSKDDVGKSVHELERACRVAEQAAND
LRAQVTELEDELTAAEDAKLRLEVTVQALKTQHERDLQGRDEAGEERRRQLAKQLRDAEV
ERDEERKQRTLAVAARKKLEGELEELKAQMASAGQGKEEAVKQLRKMQAQMKELWREVEE
TRTSREEIFSQNRESEKRLKGLEAEVLRLQEELAASDRARRQAQQDRDEMADEVANGNLS
KAAILEEKRQLEGRLGQLEEELEEEQSNSELLNDRYRKLLLQVESLTTELSAERSFSAKA
ESGRQQLERQIQELRGRLGEEDAGARARHKMTIAALESKLAQAEEQLEQETRERILSGKL
VRRAEKRLKEVVLQVEEERRVADQLRDQLEKGNLRVKQLKRQLEEAEEEASRAQAGRRRL
QRELEDVTESAESMNREVTTLRNRLRRGPLTFTTRTVRQVFRLEEGVASDEEAEEAQPGS
GPSPEPEGSPPAHPQ
>sp|Q9BYR0|KRA47_HUMAN Keratin-associated protein 4-7 OS=Homo sapiens OX=9606 GN=KRTAP4-7 PE=1 SV=2
MVSSCCGSVCSDQGCSQDLCQETCCRPSCCQTTCCRTTCYRPSCCVSSCCRPQCCQSVCC
QPTCCRPTCCETTCCHPRCCISSCCRPSCCMSSCCKPQCCQSVCCQPTCCHPSCCISSCC
RPSCCVSRCCRPQCCQSVCCQPTCCRPSCCISSCCRPSCCESSCCRPSCCRPCCCLRPVC
GRVSCHTTCYRPTCVISTCPRPLCCASSCC
>sp|Q9BYQ5|KRA46_HUMAN Keratin-associated protein 4-6 OS=Homo sapiens OX=9606 GN=KRTAP4-6 PE=2 SV=4
MVSSCCGSVCSDQGCGLETCCRPSCCQTTCCRTTCCRPSCCVSSCCRPQCCQSVCCQPTC

CRPSCCPSCCQTTCCRTTCCRPSCCVSSCCRPQCCQSVCCQPTCCRPSCSISSCCRPSCC
VSRCCRSQCCQSVCCQPTCCRPSCCISSCCRPSCCESSCCRPCCCRPCCCLRPVCGRVSC
HTTCYRPTCVISTCPRPLCCASSCC
>sp|Q9BQ66|KR412_HUMAN Keratin-associated protein 4-12 OS=Homo sapiens OX=9606 GN=KRTAP4-12 PE=1 SV=1
MVNSCCGSVCSDQGCGLENCCRPSCCQTTCCRTTCCRPSCCVSSCCRPQCCQSVCCQPTC
CRPSCCQTTCCRTTCCRPSCCVSSCCRPQCCQSVCCQPTCCRPSCCQTTCCRTTCCRPSC
CVSSCCRPQCCQSVCCQPTCCRPSCCISSSCCPSCCESSCCRPCCCLRPVCGRVSCHTTC
YRPTCVISTCPRPLCCASSCC
>sp|Q9BYR2|KRA45_HUMAN Keratin-associated protein 4-5 OS=Homo sapiens OX=9606 GN=KRTAP4-5 PE=1 SV=4
MVSSCCGSVSSEQSCGLENCCRPSCCQTTCCRTTCCRPSCCKPQCCQSVCYQPTCCHPSC
CISSCCRPYCCESSCCRPCCCQTTCCRTTCCRTTCCCPSCCVSSCCRPQCCQSVCCQPTC
CRPSCCISSCCHPSCCESSCCRPCCCVRPVCGRVSCHTTCYRPTCVISTCPRPLCCASSC
C
>sp|P26038|MOES_HUMAN Moesin OS=Homo sapiens OX=9606 GN=MSN PE=1 SV=3
MPKTISVRVTTMDAELEFAIQPNTTGKQLFDQVVKTIGLREVWFFGLQYQDTKGFSTWLK
LNKKVTAQDVRKESPLLFKFRAKFYPEDVSEELIQDITQRLFFLQVKEGILNDDIYCPPE
TAVLLASYAVQSKYGDFNKEVHKSGYLAGDKLLPQRVLEQHKLNKDQWEERIQVWHEEHR
GMLREDAVLEYLKIAQDLEMYGVNYFSIKNKKGSELWLGVDALGLNIYEQNDRLTPKIGF
PWSEIRNISFNDKKFVIKPIDKKAPDFVFYAPRLRINKRILALCMGNHELYMRRRKPDTI
EVQQMKAQAREEKHQKQMERAMLENEKKKREMAEKEKEKIEREKEELMERLKQIEEQTKK
AQQELEEQTRRALELEQERKRAQSEAEKLAKERQEAEEAKEALLQASRDQKKTQEQLALE
MAELTARISQLEMARQKKESEAVEWQQKAQMVQEDLEKTRAELKTAMSTPHVAEPAENEQ
DEQDENGAEASADLRADAMAKDRSEEERTTEAEKNERVQKHLKALTSELANARDESKKTA
NDMIHAENMRLGRDKYKTLRQIRQGNTKQRIDEFESM
>sp|Q6ZVX7|FBX50_HUMAN F-box only protein 50 OS=Homo sapiens OX=9606 GN=NCCRP1 PE=1 SV=1
MEEVREGHALGGGMEADGPASLQELPPSPRSPSPPPSPPPLPSPPSLPSPAAPEAPELPE
PAQPSEAHARQLLLEEWGPLSGGLELPQRLTWKLLLLRRPLYRNLLRSPNPEGINIYEPA
PPTGPTQRPLETLGNFRGWYIRTEKLQQNQSWTVKQQCVDLLAEGLWEELLDDEQPAITV
MDWFEDSRLDACVYELHVWLLAADRRTVIAQHHVAPRTSGRGPPGRWVQVSHVFRHYGPG
VRFIHFLHKAKNRMEPGGLRRTRVTDSSVSVQLRE
>sp|P07814|SYEP_HUMAN Bifunctional glutamate/proline--tRNA ligase OS=Homo sapiens OX=9606 GN=EPRS1 PE=1 SV=5
MATLSLTVNSGDPPLGALLAVEHVKDDVSISVEEGKENILHVSENVIFTDVNSILRYLAR
VATTAGLYGSNLMEHTEIDHWLEFSATKLSSCDSFTSTINELNHCLSLRTYLVGNSLSLA
DLCVWATLKGNAAWQEQLKQKKAPVHVKRWFGFLEAQQAFQSVGTKWDVSTTKARVAPEK
KQDVGKFVELPGAEMGKVTVRFPPEASGYLHIGHAKAALLNQHYQVNFKGKLIMRFDDTN
PEKEKEDFEKVILEDVAMLHIKPDQFTYTSDHFETIMKYAEKLIQEGKAYVDDTPAEQMK
AEREQRIDSKHRKNPIEKNLQMWEEMKKGSQFGQSCCLRAKIDMSSNNGCMRDPTLYRCK
IQPHPRTGNKYNVYPTYDFACPIVDSIEGVTHALRTTEYHDRDEQFYWIIEALGIRKPYI
WEYSRLNLNNTVLSKRKLTWFVNEGLVDGWDDPRFPTVRGVLRRGMTVEGLKQFIAAQGS
SRSVVNMEWDKIWAFNKKVIDPVAPRYVALLKKEVIPVNVPEAQEEMKEVAKHPKNPEVG
LKPVWYSPKVFIEGADAETFSEGEMVTFINWGNLNITKIHKNADGKIISLDAKLNLENKD
YKKTTKVTWLAETTHALPIPVICVTYEHLITKPVLGKDEDFKQYVNKNSKHEELMLGDPC

LKDLKKGDIIQLQRRGFFICDQPYEPVSPYSCKEAPCVLIYIPDGHTKEMPTSGSKEKTK
VEATKNETSAPFKERPTPSLNNNCTTSEDSLVLYNRVAVQGDVVRELKAKKAPKEDVDAA
VKQLLSLKAEYKEKTGQEYKPGNPPAEIGQNISSNSSASILESKSLYDEVAAQGEVVRKL
KAEKSPKAKINEAVECLLSLKAQYKEKTGKEYIPGQPPLSQSSDSSPTRNSEPAGLETPE
AKVLFDKVASQGEVVRKLKTEKAPKDQVDIAVQELLQLKAQYKSLIGVEYKPVSATGAED
KDKKKKEKENKSEKQNKPQKQNDGQRKDPSKNQGGGLSSSGAGEGQGPKKQTRLGLEAKK
EENLADWYSQVITKSEMIEYHDISGCYILRPWAYAIWEAIKDFFDAEIKKLGVENCYFPM
FVSQSALEKEKTHVADFAPEVAWVTRSGKTELAEPIAIRPTSETVMYPAYAKWVQSHRDL
PIKLNQWCNVVRWEFKHPQPFLRTREFLWQEGHSAFATMEEAAEEVLQILDLYAQVYEEL
LAIPVVKGRKTEKEKFAGGDYTTTIEAFISASGRAIQGGTSHHLGQNFSKMFEIVFEDPK
IPGEKQFAYQNSWGLTTRTIGVMTMVHGDNMGLVLPPRVACVQVVIIPCGITNALSEEDK
EALIAKCNDYRRRLLSVNIRVRADLRDNYSPGWKFNHWELKGVPIRLEVGPRDMKSCQFV
AVRRDTGEKLTVAENEAETKLQAILEDIQVTLFTRASEDLKTHMVVANTMEDFQKILDSG
KIVQIPFCGEIDCEDWIKKTTARDQDLEPGAPSMGAKSLCIPFKPLCELQPGAKCVCGKN
PAKYYTLFGRSY
>sp|Q15181|IPYR_HUMAN Inorganic pyrophosphatase OS=Homo sapiens OX=9606 GN=PPA1 PE=1 SV=2
MSGFSTEERAAPFSLEYRVFLKNEKGQYISPFHDIPIYADKDVFHMVVEVPRWSNAKMEI
ATKDPLNPIKQDVKKGKLRYVANLFPYKGYIWNYGAIPQTWEDPGHNDKHTGCCGDNDPI
DVCEIGSKVCARGEIIGVKVLGILAMIDEGETDWKVIAINVDDPDAANYNDINDVKRLKP
GYLEATVDWFRRYKVPDGKPENEFAFNAEFKDKDFAIDIIKSTHDHWKALVTKKTNGKGI
SCMNTTLSESPFKCDPDAARAIVDALPPPCESACTVPTDVDKWFHHQKN
>sp|P61106|RAB14_HUMAN Ras-related protein Rab-14 OS=Homo sapiens OX=9606 GN=RAB14 PE=1 SV=4
MATAPYNYSYIFKYIIIGDMGVGKSCLLHQFTEKKFMADCPHTIGVEFGTRIIEVSGQKI
KLQIWDTAGQERFRAVTRSYYRGAAGALMVYDITRRSTYNHLSSWLTDARNLTNPNTVII
LIGNKADLEAQRDVTYEEAKQFAEENGLLFLEASAKTGENVEDAFLEAAKKIYQNIQDGS
LDLNAAESGVQHKPSAPQGGRLTSEPQPQREGCGC
>sp|Q99456|K1C12_HUMAN Keratin, type I cytoskeletal 12 OS=Homo sapiens OX=9606 GN=KRT12 PE=1 SV=1
MDLSNNTMSLSVRTPGLSRRLSSQSVIGRPRGMSASSVGSGYGGSAFGFGASCGGGFSAA
SMFGSSSGFGGGSGSSMAGGLGAGYGRALGGGSFGGLGMGFGGSPGGGSLGILSGNDGGL
LSGSEKETMQNLNDRLASYLDKVRALEEANTELENKIREWYETRGTGTADASQSDYSKYY
PLIEDLRNKIISASIGNAQLLLQIDNARLAAEDFRMKYENELALRQGVEADINGLRRVLD
ELTLTRTDLEMQIESLNEELAYMKKNHEDELQSFRVGGPGEVSVEMDAAPGVDLTRLLND
MRAQYETIAEQNRKDAEAWFIEKSGELRKEISTNTEQLQSSKSEVTDLRRAFQNLEIELQ
SQLAMKKSLEDSLAEAEGDYCAQLSQVQQLISNLEAQLLQVRADAERQNVDHQRLLNVKA
RLELEIETYRRLLDGEAQGDGLEESLFVTDSKSQAQSTDSSKDPTKTRKIKTVVQEMVNG
EVVSSQVQEIEELM
>sp|P60900|PSA6_HUMAN Proteasome subunit alpha type-6 OS=Homo sapiens OX=9606 GN=PSMA6 PE=1 SV=1
MSRGSSAGFDRHITIFSPEGRLYQVEYAFKAINQGGLTSVAVRGKDCAVIVTQKKVPDKL
LDSSTVTHLFKITENIGCVMTGMTADSRSQVQRARYEAANWKYKYGYEIPVDMLCKRIAD
ISQVYTQNAEMRPLGCCMILIGIDEEQGPQVYKCDPAGYYCGFKATAAGVKQTESTSFLE
KKVKKKFDWTFEQTVETAITCLSTVLSIDFKPSEIEVGVVTVENPKFRILTEAEIDAHLV
ALAERD

>sp|P15880|RS2_HUMAN 40S ribosomal protein S2 OS=Homo sapiens OX=9606 GN=RPS2 PE=1 SV=2
MADDAGAAGGPGGPGGPGMGNRGGFRGGFGSGIRGRGRGRGRGRGRGRGARGGKAEDKEW
MPVTKLGRLVKDMKIKSLEEIYLFSLPIKESEIIDFFLGASLKDEVLKIMPVQKQTRAGQ
RTRFKAFVAIGDYNGHVGLGVKCSKEVATAIRGAIILAKLSIVPVRRGYWGNKIGKPHTV
PCKVTGRCGSVLVRLIPAPRGTGIVSAPVPKKLLMMAGIDDCYTSARGCTATLGNFAKAT
FDAISKTYSYLTPDLWKETVFTKSPYQEFTDHLVKTHTRVSVQRTQAPAVATT
>sp|Q9BYR8|KRA31_HUMAN Keratin-associated protein 3-1 OS=Homo sapiens OX=9606 GN=KRTAP3-1 PE=1 SV=1
MYCCALRSCSVPTGPATTFCSFDKSCRCGVCLPSTCPHEISLLQPICCDTCPPPCCKPDT
YVPTCWLLNNCHPTPGLSGINLTTYVQPGCESPCEPRC
>sp|P34932|HSP74_HUMAN Heat shock 70 kDa protein 4 OS=Homo sapiens OX=9606 GN=HSPA4 PE=1 SV=4
MSVVGIDLGFQSCYVAVARAGGIETIANEYSDRCTPACISFGPKNRSIGAAAKSQVISNA
KNTVQGFKRFHGRAFSDPFVEAEKSNLAYDIVQLPTGLTGIKVTYMEEERNFTTEQVTAM
LLSKLKETAESVLKKPVVDCVVSVPCFYTDAERRSVMDATQIAGLNCLRLMNETTAVALA
YGIYKQDLPALEEKPRNVVFVDMGHSAYQVSVCAFNRGKLKVLATAFDTTLGGRKFDEVL
VNHFCEEFGKKYKLDIKSKIRALLRLSQECEKLKKLMSANASDLPLSIECFMNDVDVSGT
MNRGKFLEMCNDLLARVEPPLRSVLEQTKLKKEDIYAVEIVGGATRIPAVKEKISKFFGK
ELSTTLNADEAVTRGCALQCAILSPAFKVREFSITDVVPYPISLRWNSPAEEGSSDCEVF
SKNHAAPFSKVLTFYRKEPFTLEAYYSSPQDLPYPDPAIAQFSVQKVTPQSDGSSSKVKV
KVRVNVHGIFSVSSASLVEVHKSEENEEPMETDQNAKEEEKMQVDQEEPHVEEQQQQTPA
ENKAESEEMETSQAGSKDKKMDQPPQAKKAKVKTSTVDLPIENQLLWQIDREMLNLYIEN
EGKMIMQDKLEKERNDAKNAVEEYVYEMRDKLSGEYEKFVSEDDRNSFTLKLEDTENWLY
EDGEDQPKQVYVDKLAELKNLGQPIKIRFQESEERPKLFEELGKQIQQYMKIISSFKNKE
DQYDHLDAADMTKVEKSTNEAMEWMNNKLNLQNKQSLTMDPVVKSKEIEAKIKELTSTCS
PIISKPKPKVEPPKEEQKNAEQNGPVDGQGDNPGPQAAEQGTDTAVPSDSDKKLPEMDID
>sp|Q9UI42|CBPA4_HUMAN Carboxypeptidase A4 OS=Homo sapiens OX=9606 GN=CPA4 PE=1 SV=2
MRWILFIGALIGSSICGQEKFFGDQVLRINVRNGDEISKLSQLVNSNNLKLNFWKSPSSF
NRPVDVLVPSVSLQAFKSFLRSQGLEYAVTIEDLQALLDNEDDEMQHNEGQERSSNNFNY
GAYHSLEAIYHEMDNIAADFPDLARRVKIGHSFENRPMYVLKFSTGKGVRRPAVWLNAGI
HSREWISQATAIWTARKIVSDYQRDPAITSILEKMDIFLLPVANPDGYVYTQTQNRLWRK
TRSRNPGSSCIGADPNRNWNASFAGKGASDNPCSEVYHGPHANSEVEVKSVVDFIQKHGN
FKGFIDLHSYSQLLMYPYGYSVKKAPDAEELDKVARLAAKALASVSGTEYQVGPTCTTVY
PASGSSIDWAYDNGIKFAFTFELRDTGTYGFLLPANQIIPTAEETWLGLKTIMEHVRDNL
Y
>sp|P62241|RS8_HUMAN 40S ribosomal protein S8 OS=Homo sapiens OX=9606 GN=RPS8 PE=1 SV=2
MGISRDNWHKRRKTGGKRKPYHKKRKYELGRPAANTKIGPRRIHTVRVRGGNKKYRALRL
DVGNFSWGSECCTRKTRIIDVVYNASNNELVRTKTLVKNCIVLIDSTPYRQWYESHYALP
LGRKKGAKLTPEEEEILNKKRSKKIQKKYDERKKNAKISSLLEEQFQQGKLLACIASRPG
QCGRADGYVLEGKELEFYLRKIKARKGK
>sp|P31151|S10A7_HUMAN Protein S100-A7 OS=Homo sapiens OX=9606 GN=S100A7 PE=1 SV=4
MSNTQAERSIIGMIDMFHKYTRRDDKIEKPSLLTMMKENFPNFLSACDKKGTNYLADVFE
KKDKNEDKKIDFSEFLSLLGDIATDYHKQSHGAAPCSGGSQ

>sp|Q86SG5|S1A7A_HUMAN Protein S100-A7A OS=Homo sapiens OX=9606 GN=S100A7A PE=1 SV=3
MSNTQAERSIIGMIDMFHKYTGRDGKIEKPSLLTMMKENFPNFLSACDKKGIHYLATVFE
KKDKNEDKKIDFSEFLSLLGDIAADYHKQSHGAAPCSGGSQ
>sp|O14818|PSA7_HUMAN Proteasome subunit alpha type-7 OS=Homo sapiens OX=9606 GN=PSMA7 PE=1 SV=1
MSYDRAITVFSPDGHLFQVEYAQEAVKKGSTAVGVRGRDIVVLGVEKKSVAKLQDERTVR
KICALDDNVCMAFAGLTADARIVINRARVECQSHRLTVEDPVTVEYITRYIASLKQRYTQ
SNGRRPFGISALIVGFDFDGTPRLYQTDPSGTYHAWKANAIGRGAKSVREFLEKNYTDEA
IETDDLTIKLVIKALLEVVQSGGKNIELAVMRRDQSLKILNPEEIEKYVAEIEKEKEENE
KKKQKKAS
>sp|Q8TAA3|PSMA8_HUMAN Proteasome subunit alpha-type 8 OS=Homo sapiens OX=9606 GN=PSMA8 PE=2 SV=3
MASRYDRAITVFSPDGHLFQVEYAQEAVKKGSTAVGIRGTNIVVLGVEKKSVAKLQDERT
VRKICALDDHVCMAFAVLTIFIGLTADARVVINRARVECQSHKLTVEDPVTVEYITRFIA
TLKQKYTQSNGRRPFGISALIVGFDDDGISRLYQTDPSGTYHAWKANAIGRSAKTVREFL
EKNYTEDAIASDSEAIKLAIKALLEVVQSGGKNIELAIIRRNQPLKMFSAKEVELYVTEI
EKEKEEAEKKKSKKSV
>sp|P60981|DEST_HUMAN Destrin OS=Homo sapiens OX=9606 GN=DSTN PE=1 SV=3
MASGVQVADEVCRIFYDMKVRKCSTPEEIKKRKKAVIFCLSADKKCIIVEEGKEILVGDV
GVTITDPFKHFVGMLPEKDCRYALYDASFETKESRKEELMFFLWAPELAPLKSKMIYASS
KDAIKKKFQGIKHECQANGPEDLNRACIAEKLGGSLIVAFEGCPV
>sp|P20339|RAB5A_HUMAN Ras-related protein Rab-5A OS=Homo sapiens OX=9606 GN=RAB5A PE=1 SV=2
MASRGATRPNGPNTGNKICQFKLVLLGESAVGKSSLVLRFVKGQFHEFQESTIGAAFLTQ
TVCLDDTTVKFEIWDTAGQERYHSLAPMYYRGAQAAIVVYDITNEESFARAKNWVKELQR
QASPNIVIALSGNKADLANKRAVDFQEAQSYADDNSLLFMETSAKTSMNVNEIFMAIAKK
LPKNEPQNPGANSARGRGVDLTEPTQPTRNQCCSN
>sp|Q07020|RL18_HUMAN 60S ribosomal protein L18 OS=Homo sapiens OX=9606 GN=RPL18 PE=1 SV=2
MGVDIRHNKDRKVRRKEPKSQDIYLRLLVKLYRFLARRTNSTFNQVVLKRLFMSRTNRPP
LSLSRMIRKMKLPGRENKTAVVVGTITDDVRVQEVPKLKVCALRVTSRARSRILRAGGKI
LTFDQLALDSPKGCGTVLLSGPRKGREVYRHFGKAPGTPHSHTKPYVRSKGRKFERARGR
RASRGYKN
>sp|Q16610|ECM1_HUMAN Extracellular matrix protein 1 OS=Homo sapiens OX=9606 GN=ECM1 PE=1 SV=2
MGTTARAALVLTYLAVASAASEGGFTATGQRQLRPEHFQEVGYAAPPSPPLSRSLPMDHP
DSSQHGPPFEGQSQVQPPPSQEATPLQQEKLLPAQLPAEKEVGPPLPQEAVPLQKELPSL
QHPNEQKEGTPAPFGDQSHPEPESWNAAQHCQQDRSQGGWGHRLDGFPPGRPSPDNLNQI
CLPNRQHVVYGPWNLPQSSYSHLTRQGETLNFLEIGYSRCCHCRSHTNRLECAKLVWEEA
MSRFCEAEFSVKTRPHWCCTRQGEARFSCFQEEAPQPHYQLRACPSHQPDISSGLELPFP
PGVPTLDNIKNICHLRRFRSVPRNLPATDPLQRELLALIQLEREFQRCCRQGNNHTCTWK
AWEDTLDKYCDREYAVKTHHHLCCRHPPSPTRDECFARRAPYPNYDRDILTIDIGRVTPN
LMGHLCGNQRVLTKHKHIPGLIHNMTARCCDLPFPEQACCAEEEKLTFINDLCGPRRNIW
RDPALCCYLSPGDEQVNCFNINYLRNVALVSGDTENAKGQGEQGSTGGTNISSTSEPKEE

>sp|P59998|ARPC4_HUMAN Actin-related protein 2/3 complex subunit 4 OS=Homo sapiens OX=9606 GN=ARPC4 PE=1 SV=3
MTATLRPYLSAVRATLQAALCLENFSSQVVERHNKPEVEVRSSKELLLQPVTISRNEKEK
VLIEGSINSVRVSIAVKQADEIEKILCHKFMRFMMMRAENFFILRRKPVEGYDISFLITN
FHTEQMYKHKLVDFVIHFMEEIDKEISEMKLSVNARARIVAEEFLKNF
>sp|P61247|RS3A_HUMAN 40S ribosomal protein S3a OS=Homo sapiens OX=9606 GN=RPS3A PE=1 SV=2
MAVGKNKRLTKGGKKGAKKKVVDPFSKKDWYDVKAPAMFNIRNIGKTLVTRTQGTKIASD
GLKGRVFEVSLADLQNDEVAFRKFKLITEDVQGKNCLTNFHGMDLTRDKMCSMVKKWQTM
IEAHVDVKTTDGYLLRLFCVGFTKKRNNQIRKTSYAQHQQVRQIRKKMMEIMTREVQTND
LKEVVNKLIPDSIGKDIEKACQSIYPLHDVFVRKVKMLKKPKFELGKLMELHGEGSSSGK
ATGDETGAKVERADGYEPPVQESV
>sp|P0DOY3|IGLC3_HUMAN Immunoglobulin lambda constant 3 OS=Homo sapiens OX=9606 GN=IGLC3 PE=1 SV=1
GQPKAAPSVTLFPPSSEELQANKATLVCLISDFYPGAVTVAWKADSSPVKAGVETTTPSK
QSNNKYAASSYLSLTPEQWKSHKSYSCQVTHEGSTVEKTVAPTECS
>sp|P0DOY2|IGLC2_HUMAN Immunoglobulin lambda constant 2 OS=Homo sapiens OX=9606 GN=IGLC2 PE=1 SV=1
GQPKAAPSVTLFPPSSEELQANKATLVCLISDFYPGAVTVAWKADSSPVKAGVETTTPSK
QSNNKYAASSYLSLTPEQWKSHRSYSCQVTHEGSTVEKTVAPTECS
>sp|P0CF74|IGLC6_HUMAN Immunoglobulin lambda constant 6 OS=Homo sapiens OX=9606 GN=IGLC6 PE=1 SV=1
GQPKAAPSVTLFPPSSEELQANKATLVCLISDFYPGAVKVAWKADGSPVNTGVETTTPSK
QSNNKYAASSYLSLTPEQWKSHRSYSCQVTHEGSTVEKTVAPAECS
>sp|P0DOX8|IGL1_HUMAN Immunoglobulin lambda-1 light chain OS=Homo sapiens OX=9606 PE=1 SV=1
QSALTQPPSASGSLGQSVTISCTGTSSDVGGYNYVSWYQQHAGKAPKVIIYEVNKRPSGV
PDRFSGSKSGNTASLTVSGLQAEDEADYYCSSYEGSDNFVFGTGTKVTVLGQPKANPTVT
LFPPSSEELQANKATLVCLISDFYPGAVTVAWKADGSPVKAGVETTKPSKQSNNKYAASS
YLSLTPEQWKSHRSYSCQVTHEGSTVEKTVAPTECS
>sp|B9A064|IGLL5_HUMAN Immunoglobulin lambda-like polypeptide 5 OS=Homo sapiens OX=9606 GN=IGLL5 PE=2 SV=2
MRPKTGQVGCETPEELGPGPRQRWPLLLLGLAMVAHGLLRPMVAPQSGDPDPGASVGSSR
SSLRSLWGRLLLQPSPQRADPRCWPRGFWSEPQSLCYVFGTGTKVTVLGQPKANPTVTLF
PPSSEELQANKATLVCLISDFYPGAVTVAWKADGSPVKAGVETTKPSKQSNNKYAASSYL
SLTPEQWKSHRSYSCQVTHEGSTVEKTVAPTECS
>sp|A0M8Q6|IGLC7_HUMAN Immunoglobulin lambda constant 7 OS=Homo sapiens OX=9606 GN=IGLC7 PE=1 SV=3
GQPKAAPSVTLFPPSSEELQANKATLVCLVSDFNPGAVTVAWKADGSPVKVGVETTKPSK
QSNNKYAASSYLSLTPEQWKSHRSYSCRVTHEGSTVEKTVAPAECS
>sp|P0CG04|IGLC1_HUMAN Immunoglobulin lambda constant 1 OS=Homo sapiens OX=9606 GN=IGLC1 PE=1 SV=1
GQPKANPTVTLFPPSSEELQANKATLVCLISDFYPGAVTVAWKADGSPVKAGVETTKPSK
QSNNKYAASSYLSLTPEQWKSHRSYSCQVTHEGSTVEKTVAPTECS
>sp|P01037|CYTN_HUMAN Cystatin-SN OS=Homo sapiens OX=9606 GN=CST1 PE=1 SV=3
MAQYLSTLLLLLATLAVALAWSPKEEDRIIPGGIYNADLNDEWVQRALHFAISEYNKATK

DDYYRRPLRVLRARQQTVGGVNYFFDVEVGRTICTKSQPNLDTCAFHEQPELQKKQLCSF
EIYEVPWENRRSLVKSRCQES
>sp|P12236|ADT3_HUMAN ADP/ATP translocase 3 OS=Homo sapiens OX=9606 GN=SLC25A6 PE=1 SV=4
MTEQAISFAKDFLAGGIAAAISKTAVAPIERVKLLLQVQHASKQIAADKQYKGIVDCIVR
IPKEQGVLSFWRGNLANVIRYFPTQALNFAFKDKYKQIFLGGVDKHTQFWRYFAGNLASG
GAAGATSLCFVYPLDFARTRLAADVGKSGTEREFRGLGDCLVKITKSDGIRGLYQGFSVS
VQGIIIYRAAYFGVYDTAKGMLPDPKNTHIVVSWMIAQTVTAVAGVVSYPFDTVRRRMMM
QSGRKGADIMYTGTVDCWRKIFRDEGGKAFFKGAWSNVLRGMGGAFVLVLYDELKKVI
>sp|P12235|ADT1_HUMAN ADP/ATP translocase 1 OS=Homo sapiens OX=9606 GN=SLC25A4 PE=1 SV=4
MGDHAWSFLKDFLAGGVAAAVSKTAVAPIERVKLLLQVQHASKQISAEKQYKGIIDCVVR
IPKEQGFLSFWRGNLANVIRYFPTQALNFAFKDKYKQLFLGGVDRHKQFWRYFAGNLASG
GAAGATSLCFVYPLDFARTRLAADVGKGAAQREFHGLGDCIIKIFKSDGLRGLYQGFNVS
VQGIIIYRAAYFGVYDTAKGMLPDPKNVHIFVSWMIAQSVTAVAGLVSYPFDTVRRRMMM
QSGRKGADIMYTGTVDCWRKIAKDEGAKAFFKGAWSNVLRGMGGAFVLVLYDEIKKYV
>sp|P07741|APT_HUMAN Adenine phosphoribosyltransferase OS=Homo sapiens OX=9606 GN=APRT PE=1 SV=2
MADSELQLVEQRIRSFPDFPTPGVVFRDISPVLKDPASFRAAIGLLARHLKATHGGRIDY
IAGLDSRGFLFGPSLAQELGLGCVLIRKRGKLPGPTLWASYSLEYGKAELEIQKDALEPG
QRVVVVDDLLATGGTMNAACELLGRLQAEVLECVSLVELTSLKGREKLAPVPFFSLLQYE
>sp|O43175|SERA_HUMAN D-3-phosphoglycerate dehydrogenase OS=Homo sapiens OX=9606 GN=PHGDH PE=1 SV=4
MAFANLRKVLISDSLDPCCRKILQDGGLQVVEKQNLSKEELIAELQDCEGLIVRSATKVT
ADVINAAEKLQVVGRAGTGVDNVDLEAATRKGILVMNTPNGNSLSAAELTCGMIMCLARQ
IPQATASMKDGKWERKKFMGTELNGKTLGILGLGRIGREVATRMQSFGMKTIGYDPIISP
EVSASFGVQQLPLEEIWPLCDFITVHTPLLPSTTGLLNDNTFAQCKKGVRVVNCARGGIV
DEGALLRALQSGQCAGAALDVFTEEPPRDRALVDHENVISCPHLGASTKEAQSRCGEEIA
VQFVDMVKGKSLTGVVNAQALTSAFSPHTKPWIGLAEALGTLMRAWAGSPKGTIQVITQG
TSLKNAGNCLSPAVIVGLLKEASKQADVNLVNAKLLVKEAGLNVTTSHSPAAPGEQGFGE
CLLAVALAGAPYQAVGLVQGTTPVLQGLNGAVFRPEVPLRRDLPLLLFRTQTSDPAMLPT
MIGLLAEAGVRLLSYQTSLVSDGETWHVMGISSLLPSLEAWKQHVTEAFQFHF
>sp|P11279|LAMP1_HUMAN Lysosome-associated membrane glycoprotein 1 OS=Homo sapiens OX=9606 GN=LAMP1 PE=1 SV=3
MAAPGSARRPLLLLLLLLLLGLMHCASAAMFMVKNGNGTACIMANFSAAFSVNYDTKSGP
KNMTFDLPSDATVVLNRSSCGKENTSDPSLVIAFGRGHTLTLNFTRNATRYSVQLMSFVY
NLSDTHLFPNASSKEIKTVESITDIRADIDKKYRCVSGTQVHMNNVTVTLHDATIQAYLS
NSSFSRGETRCEQDRPSPTTAPPAPPSPSPSPVPKSPSVDKYNVSGTNGTCLLASMGLQL
NLTYERKDNTTVTRLLNINPNKTSASGSCGAHLVTLELHSEGTTVLLFQFGMNASSSRFF
LQGIQLNTILPDARDPAFKAANGSLRALQATVGNSYKCNAEEHVRVTKAFSVNIFKVWVQ
AFKVEGGQFGSVEECLLDENSMLIPIAVGGALAGLVLIVLIAYLVGRKRSHAGYQTI
>sp|P21281|VATB2_HUMAN V-type proton ATPase subunit B, brain isoform OS=Homo sapiens OX=9606 GN=ATP6V1B2 PE=1 SV=3
MALRAMRGIVNGAAPELPVPTGGPAVGAREQALAVSRNYLSQPRLTYKTVSGVNGPLVIL
DHVKFPRYAEIVHLTLPDGTKRSGQVLEVSGSKAVVQVFEGTSGIDAKKTSCEFTGDILR
TPVSEDMLGRVFNGSGKPIDRGPVVLAEDFLDIMGQPINPQCRIYPEEMIQTGISAIDGM
NSIARGQKIPIFSAAGLPHNEIAAQICRQAGLVKKSKDVVDYSEENFAIVFAAMGVNMET

ARFFKSDFEENGSMDNVCLFLNLANDPTIERIITPRLALTTAEFLAYQCEKHVLVILTDM
SSYAEALREVSAAREEVPGRRGFPGYMYTDLATIYERAGRVEGRNGSITQIPILTMPNDD
ITHPIPDLTGYITEGQIYVDRQLHNRQIYPPINVLPSLSRLMKSAIGEGMTRKDHADVSN
QLYACYAIGKDVQAMKAVVGEEALTSDDLLYLEFLQKFERNFIAQGPYENRTVFETLDIG
WQLLRIFPKEMLKRIPQSTLSEFYPRDSAKH
>sp|P15313|VATB1_HUMAN V-type proton ATPase subunit B, kidney isoform OS=Homo sapiens OX=9606 GN=ATP6V1B1 PE=1 SV=3
MAMEIDSRPGGLPGSSCNLGAAREHMQAVTRNYITHPRVTYRTVCSVNGPLVVLDRVKFA
QYAEIVHFTLPDGTQRSGQVLEVAGTKAIVQVFEGTSGIDARKTTCEFTGDILRTPVSED
MLGRVFNGSGKPIDKGPVVMAEDFLDINGQPINPHSRIYPEEMIQTGISPIDVMNSIARG
QKIPIFSAAGLPHNEIAAQICRQAGLVKKSKAVLDYHDDNFAIVFAAMGVNMETARFFKS
DFEQNGTMGNVCLFLNLANDPTIERIITPRLALTTAEFLAYQCEKHVLVILTDMSSYAEA
LREVSAAREEVPGRRGFPGYMYTDLATIYERAGRVEGRGGSITQIPILTMPNDDITHPIP
DLTGFITEGQIYVDRQLHNRQIYPPINVLPSLSRLMKSAIGEGMTRKDHGDVSNQLYACY
AIGKDVQAMKAVVGEEALTSEDLLYLEFLQKFEKNFINQGPYENRSVFESLDLGWKLLRI
FPKEMLKRIPQAVIDEFYSREGALQDLAPDTAL
>sp|P26639|SYTC_HUMAN Threonine--tRNA ligase 1, cytoplasmic OS=Homo sapiens OX=9606 GN=TARS1 PE=1 SV=3
MFEEKASSPSGKMGGEEKPIGAGEEKQKEGGKKKNKEGSGDGGRAELNPWPEYIYTRLEM
YNILKAEHDSILAEKAEKDSKPIKVTLPDGKQVDAESWKTTPYQIACGISQGLADNTVIA
KVNNVVWDLDRPLEEDCTLELLKFEDEEAQAVYWHSSAHIMGEAMERVYGGCLCYGPPIE
NGFYYDMYLEEGGVSSNDFSSLEALCKKIIKEKQAFERLEVKKETLLAMFKYNKFKCRIL
NEKVNTPTTTVYRCGPLIDLCRGPHVRHTGKIKALKIHKNSSTYWEGKADMETLQRIYGI
SFPDPKMLKEWEKFQEEAKNRDHRKIGRDQELYFFHELSPGSCFFLPKGAYIYNALIEFI
RSEYRKRGFQEVVTPNIFNSRLWMTSGHWQHYSENMFSFEVEKELFALKPMNCPGHCLMF
DHRPRSWRELPLRLADFGVLHRNELSGALTGLTRVRRFQQDDAHIFCAMEQIEDEIKGCL
DFLRTVYSVFGFSFKLNLSTRPEKFLGDIEVWDQAEKQLENSLNEFGEKWELNSGDGAFY
GPKIDIQIKDAIGRYHQCATIQLDFQLPIRFNLTYVSHDGDDKKRPVIVHRAILGSVERM
IAILTENYGGKWPFWLSPRQVMVVPVGPTCDEYAQKVRQQFHDAKFMADIDLDPGCTLNK
KIRNAQLAQYNFILVVGEKEKISGTVNIRTRDNKVHGERTISETIERLQQLKEFRSKQAE
EEF
>sp|P22392|NDKB_HUMAN Nucleoside diphosphate kinase B OS=Homo sapiens OX=9606 GN=NME2 PE=1 SV=1
MANLERTFIAIKPDGVQRGLVGEIIKRFEQKGFRLVAMKFLRASEEHLKQHYIDLKDRPF
FPGLVKYMNSGPVVAMVWEGLNVVKTGRVMLGETNPADSKPGTIRGDFCIQVGRNIIHGS
DSVKSAEKEISLWFKPEELVDYKSCAHDWVYE
>sp|O60361|NDK8_HUMAN Putative nucleoside diphosphate kinase OS=Homo sapiens OX=9606 GN=NME2P1 PE=5 SV=1
MQCGLVGKIIKRFEQKGFRLVAMKFLPASEEHLKQHYIDLKDRPFFPGLVKYMNSGPVVA
MVWEGLNVVKTGRVMLGETNPADSKPGTIRGDFCIQVGRNIIHGSDSVKSAEKEISLRFK
PEELVDYKSCAHDWVYE
>sp|P30050|RL12_HUMAN 60S ribosomal protein L12 OS=Homo sapiens OX=9606 GN=RPL12 PE=1 SV=1
MPPKFDPNEIKVVYLRCTGGEVGATSALAPKIGPLGLSPKKVGDDIAKATGDWKGLRITV
KLTIQNRQAQIEVVPSASALIIKALKEPPRDRKKQKNIKHSGNITFDEIVNIARQMRHRS
LARELSGTIKEILGTAQSVGCNVDGRHPHDIIDDINSGAVECPAS

>sp|P61158|ARP3_HUMAN Actin-related protein 3 OS=Homo sapiens OX=9606 GN=ACTR3 PE=1 SV=3
MAGRLPACVVDCGTGYTKLGYAGNTEPQFIIPSCIAIKESAKVGDQAQRRVMKGVDDLDF
FIGDEAIEKPTYATKWPIRHGIVEDWDLMERFMEQVIFKYLRAEPEDHYFLLTEPPLNTP
ENREYTAEIMFESFNVPGLYIAVQAVLALAASWTSRQVGERTLTGTVIDSGDGVTHVIPV
AEGYVIGSCIKHIPIAGRDITYFIQQLLRDREVGIPPEQSLETAKAVKERYSYVCPDLVK
EFNKYDTDGSKWIKQYTGINAISKKEFSIDVGYERFLGPEIFFHPEFANPDFTQPISEVV
DEVIQNCPIDVRRPLYKNIVLSGGSTMFRDFGRRLQRDLKRTVDARLKLSEELSGGRLKP
KPIDVQVITHHMQRYAVWFGGSMLASTPEFYQVCHTKKDYEEIGPSICRHNPVFGVMS
>sp|Q9P1U1|ARP3B_HUMAN Actin-related protein 3B OS=Homo sapiens OX=9606 GN=ACTR3B PE=2 SV=1
MAGSLPPCVVDCGTGYTKLGYAGNTEPQFIIPSCIAIRESAKVVDQAQRRVLRGVDDLDF
FIGDEAIDKPTYATKWPIRHGIIEDWDLMERFMEQVVFKYLRAEPEDHYFLMTEPPLNTP
ENREYLAEIMFESFNVPGLYIAVQAVLALAASWTSRQVGERTLTGIVIDSGDGVTHVIPV
AEGYVIGSCIKHIPIAGRDITYFIQQLLREREVGIPPEQSLETAKAIKEKYCYICPDIVK
EFAKYDVDPRKWIKQYTGINAINQKKFVIDVGYERFLGPEIFFHPEFANPDFMESISDVV
DEVIQNCPIDVRRPLYKNVVLSGGSTMFRDFGRRLQRDLKRVVDARLRLSEELSGGRIKP
KPVEVQVVTHHMQRYAVWFGGSMLASTPEFFQVCHTKKDYEEYGPSICRHNPVFGVMS
>sp|Q9C0K3|ARP3C_HUMAN Actin-related protein 3C OS=Homo sapiens OX=9606 GN=ACTR3C PE=2 SV=1
MFESFNVPGLYIAVQAVLALAASWTSRQVGERTLTGIVIDSGDGVTHVIPVAEGYVIGSC
IKHIPIAGRDITYFIQQLLREREVGIPPEQSLETAKAIKEKYCYICPDIVKEFAKYDVDP
QKWIKQYTGINAINQKKFVIDVGYERFLGPEIFFHPEFANPDSMESISDVVDEVIQNCPI
DVRRPLYKMEQIPLSYPQGHGFHPLSPPFH
>sp|P01024|CO3_HUMAN Complement C3 OS=Homo sapiens OX=9606 GN=C3 PE=1 SV=2
MGPTSGPSLLLLLLTHLPLALGSPMYSIITPNILRLESEETMVLEAHDAQGDVPVTVTVH
DFPGKKLVLSSEKTVLTPATNHMGNVTFTIPANREFKSEKGRNKFVTVQATFGTQVVEKV
VLVSLQSGYLFIQTDKTIYTPGSTVLYRIFTVNHKLLPVGRTVMVNIENPEGIPVKQDSL
SSQNQLGVLPLSWDIPELVNMGQWKIRAYYENSPQQVFSTEFEVKEYVLPSFEVIVEPTE
KFYYIYNEKGLEVTITARFLYGKKVEGTAFVIFGIQDGEQRISLPESLKRIPIEDGSGEV
VLSRKVLLDGVQNPRAEDLVGKSLYVSATVILHSGSDMVQAERSGIPIVTSPYQIHFTKT
PKYFKPGMPFDLMVFVTNPDGSPAYRVPVAVQGEDTVQSLTQGDGVAKLSINTHPSQKPL
SITVRTKKQELSEAEQATRTMQALPYSTVGNSNNYLHLSVLRTELRPGETLNVNFLLRMD
RAHEAKIRYYTYLIMNKGRLLKAGRQVREPGQDLVVLPLSITTDFIPSFRLVAYYTLIGA
SGQREVVADSVWVDVKDSCVGSLVVKSGQSEDRQPVPGQQMTLKIEGDHGARVVLVAVDK
GVFVLNKKNKLTQSKIWDVVEKADIGCTPGSGKDYAGVFSDAGLTFTSSSGQQTAQRAEL
QCPQPAARRRRSVQLTEKRMDKVGKYPKELRKCCEDGMRENPMRFSCQRRTRFISLGEAC
KKVFLDCCNYITELRRQHARASHLGLARSNLDEDIIAEENIVSRSEFPESWLWNVEDLKE
PPKNGISTKLMNIFLKDSITTWEILAVSMSDKKGICVADPFEVTVMQDFFIDLRLPYSVV
RNEQVEIRAVLYNYRQNQELKVRVELLHNPAFCSLATTKRRHQQTVTIPPKSSLSVPYVI
VPLKTGLQEVEVKAAVYHHFISDGVRKSLKVVPEGIRMNKTVAVRTLDPERLGREGVQKE
DIPPADLSDQVPDTESETRILLQGTPVAQMTEDAVDAERLKHLIVTPSGCGEQNMIGMTP
TVIAVHYLDETEQWEKFGLEKRQGALELIKKGYTQQLAFRQPSSAFAAFVKRAPSTWLTA
YVVKVFSLAVNLIAIDSQVLCGAVKWLILEKQKPDGVFQEDAPVIHQEMIGGLRNNNEKD
MALTAFVLISLQEAKDICEEQVNSLPGSITKAGDFLEANYMNLQRSYTVAIAGYALAQMG
RLKGPLLNKFLTTAKDKNRWEDPGKQLYNVEATSYALLALLQLKDFDFVPPVVRWLNEQR

YYGGGYGSTQATFMVFQALAQYQKDAPDHQELNLDVSLQLPSRSSKITHRIHWESASLLR
SEETKENEGFTVTAEGKGQGTLSVVTMYHAKAKDQLTCNKFDLKVTIKPAPETEKRPQDA
KNTMILEICTRYRGDQDATMSILDISMMTGFAPDTDDLKQLANGVDRYISKYELDKAFSD
RNTLIIYLDKVSHSEDDCLAFKVHQYFNVELIQPGAVKVYAYYNLEESCTRFYHPEKEDG
KLNKLCRDELCRCAEENCFIQKSDDKVTLEERLDKACEPGVDYVYKTRLVKVQLSNDFDE
YIMAIEQTIKSGSDEVQVGQQRTFISPIKCREALKLEEKKHYLMWGLSSDFWGEKPNLSY
IIGKDTWVEHWPEEDECQDEENQKQCQDLGAFTESMVVFGCPN
>sp|P28070|PSB4_HUMAN Proteasome subunit beta type-4 OS=Homo sapiens OX=9606 GN=PSMB4 PE=1 SV=4
MEAFLGSRSGLWAGGPAPGQFYRIPSTPDSFMDPASALYRGPITRTQNPMVTGTSVLGVK
FEGGVVIAADMLGSYGSLARFRNISRIMRVNNSTMLGASGDYADFQYLKQVLGQMVIDEE
LLGDGHSYSPRAIHSWLTRAMYSRRSKMNPLWNTMVIGGYADGESFLGYVDMLGVAYEAP
SLATGYGAYLAQPLLREVLEKQPVLSQTEARDLVERCMRVLYYRDARSYNRFQIATVTEK
GVEIEGPLSTETNWDIAHMISGFE
>sp|O00204|ST2B1_HUMAN Sulfotransferase 2B1 OS=Homo sapiens OX=9606 GN=SULT2B1 PE=1 SV=2
MDGPAEPQIPGLWDTYEDDISEISQKLPGEYFRYKGVPFPVGLYSLESISLAENTQDVRD
DDIFIITYPKSGTTWMIEIICLILKEGDPSWIRSVPIWERAPWCETIVGAFSLPDQYSPR
LMSSHLPIQIFTKAFFSSKAKVIYMGRNPRDVVVSLYHYSKIAGQLKDPGTPDQFLRDFL
KGEVQFGSWFDHIKGWLRMKGKDNFLFITYEELQQDLQGSVERICGFLGRPLGKEALGSV
VAHSTFSAMKANTMSNYTLLPPSLLDHRRGAFLRKGVCGDWKNHFTVAQSEAFDRAYRKQ
MRGMPTFPWDEDPEEDGSPDPEPSPEPEPKPSLEPNTSLEREPRPNSSPSPSPGQASETP
HPRPS
>sp|O95865|DDAH2_HUMAN N(G),N(G)-dimethylarginine dimethylaminohydrolase 2 OS=Homo sapiens OX=9606 GN=DDAH2 PE=1 SV=1
MGTPGEGLGRCSHALIRGVPESLASGEGAGAGLPALDLAKAQREHGVLGGKLRQRLGLQL
LELPPEESLPLGPLLGDTAVIQGDTALITRPWSPARRPEVDGVRKALQDLGLRIVEIGDE
NATLDGTDVLFTGREFFVGLSKWTNHRGAEIVADTFRDFAVSTVPVSGPSHLRGLCGMGG
PRTVVAGSSDAAQKAVRAMAVLTDHPYASLTLPDDAAADCLFLRPGLPGVPPFLLHRGGG
DLPNSQEALQKLSDVTLVPVSCSELEKAGAGLSSLCLVLSTRPHS
>sp|P62826|RAN_HUMAN GTP-binding nuclear protein Ran OS=Homo sapiens OX=9606 GN=RAN PE=1 SV=3
MAAQGEPQVQFKLVLVGDGGTGKTTFVKRHLTGEFEKKYVATLGVEVHPLVFHTNRGPIK
FNVWDTAGQEKFGGLRDGYYIQAQCAIIMFDVTSRVTYKNVPNWHRDLVRVCENIPIVLC
GNKVDIKDRKVKAKSIVFHRKKNLQYYDISAKSNYNFEKPFLWLARKLIGDPNLEFVAMP
ALAPPEVVMDPALAAQYEHDLEVAQTTALPDEDDDL
>sp|P07195|LDHB_HUMAN L-lactate dehydrogenase B chain OS=Homo sapiens OX=9606 GN=LDHB PE=1 SV=2
MATLKEKLIAPVAEEEATVPNNKITVVGVGQVGMACAISILGKSLADELALVDVLEDKLK
GEMMDLQHGSLFLQTPKIVADKDYSVTANSKIVVVTAGVRQQEGESRLNLVQRNVNVFKF
IIPQIVKYSPDCIIIVVSNPVDILTYVTWKLSGLPKHRVIGSGCNLDSARFRYLMAEKLG
IHPSSCHGWILGEHGDSSVAVWSGVNVAGVSLQELNPEMGTDNDSENWKEVHKMVVESAY
EVIKLKGYTNWAIGLSVADLIESMLKNLSRIHPVSTMVKGMYGIENEVFLSLPCILNARG
LTSVINQKLKDDEVAQLKKSADTLWDIQKDLKDL
>sp|P62701|RS4X_HUMAN 40S ribosomal protein S4, X isoform OS=Homo sapiens OX=9606 GN=RPS4X PE=1 SV=2

MARGPKKHLKRVAAPKHWMLDKLTGVFAPRPSTGPHKLRECLPLIIFLRNRLKYALTGDE
VKKICMQRFIKIDGKVRTDITYPAGFMDVISIDKTGENFRLIYDTKGRFAVHRITPEEAK
YKLCKVRKIFVGTKGIPHLVTHDARTIRYPDPLIKVNDTIQIDLETGKITDFIKFDTGNL
CMVTGGANLGRIGVITNRERHPGSFDVVHVKDANGNSFATRLSNIFVIGKGNKPWISLPR
GKGIRLTIAEERDKRLAAKQSSG
>sp|Q8TD47|RS4Y2_HUMAN 40S ribosomal protein S4, Y isoform 2 OS=Homo sapiens OX=9606 GN=RPS4Y2 PE=2 SV=3
MARGPKKHLKRVAAPKHWMLDKLTGVFAPRPSTGPHKLRECLPLIVFLRNRLKYALTGDE
VKKICMQHFLKIDGKVRVDITYPAGFIDVISIEKTGEHFRLVYNTKGCFAVHRITVEEAK
YKLCKVRKITVGTKGIPHLVTHDARTIRYPDPLIKVNDTVQIDLGTGKITSFIKFDTGNV
CMVIAGANLGRVGVITNRERHPGSCDVVHVKDANGNSFATRISNIFVIGNGNKPWISLPR
GKGIRLTIAEERDKRLAAKQSSG
>sp|P22090|RS4Y1_HUMAN 40S ribosomal protein S4, Y isoform 1 OS=Homo sapiens OX=9606 GN=RPS4Y1 PE=1 SV=2
MARGPKKHLKRVAAPKHWMLDKLTGVFAPRPSTGPHKLRECLPLIVFLRNRLKYALTGDE
VKKICMQRFIKIDGKVRVDVTYPAGFMDVISIEKTGEHFRLVYDTKGRFAVHRITVEEAK
YKLCKVRKITVGVKGIPHLVTHDARTIRYPDPVIKVNDTVQIDLGTGKIINFIKFDTGNL
CMVIGGANLGRVGVITNRERHPGSFDVVHVKDANGNSFATRLSNIFVIGNGNKPWISLPR
GKGIRLTVAEERDKRLATKQSSG
>sp|Q8N1K5|THMS1_HUMAN Protein THEMIS OS=Homo sapiens OX=9606 GN=THEMIS PE=1 SV=3
MALSLEEFVHSLDLRTLPRVLEIQAGIYLEGSIYEMFGNECCFSTGEVIKITGLKVKKII
AEICEQIEGCESLQPFELPMNFPGLFKIVADKTPYLTMEEITRTIHIGPSRLGHPCFYHQ
KDIKLENLIIKQGEQIMLNSVEEIDGEIMVSCAVARNHQTHSFNLPLSQEGEFYECEDER
IYTLKEIVEWKIPKNRTRTVNLTDFSNKWDSTNPFPKDFYGTLILKPVYEIQGVMKFRKD
IIRILPSLDVEVKDITDSYDANWFLQLLSTEDLFEMTSKEFPIVTEVIEAPEGNHLPQSI
LQPGKTIVIHKKYQASRILASEIRSNFPKRHFLIPTSYKGKFKRRPREFPTAYDLEIAKS
EKEPLHVVATKAFHSPHDKLSSVSVGDQFLVHQSETTEVLCEGIKKVVNVLACEKILKKS
YEAALLPLYMEGGFVEVIHDKKQYPISELCKQFRLPFNVKVSVRDLSIEEDVLAATPGLQ
LEEDITDSYLLISDFANPTECWEIPVGRLNMTVQLVSNFSRDAEPFLVRTLVEEITEEQY
YMMRRYESSASHPPPRPPKHPSVEETKLTLLTLAEERTVDLPKSPKRHHVDITKKLHPNQ
AGLDSKVLIGSQNDLVDEEKERSNRGATAIAETFKNEKHQK
>sp|Q96G03|PGM2_HUMAN Phosphopentomutase OS=Homo sapiens OX=9606 GN=PGM2 PE=1 SV=4
MAAPEGSGLGEDARLDQETAQWLRWDKNSLTLEAVKRLIAEGNKEELRKCFGARMEFGTA
GLRAAMGPGISRMNDLTIIQTTQGFCRYLEKQFSDLKQKGIVISFDARAHPSSGGSSRRF
ARLAATTFISQGIPVYLFSDITPTPFVPFTVSHLKLCAGIMITASHNPKQDNGYKVYWDN
GAQIISPHDKGISQAIEENLEPWPQAWDDSLIDSSPLLHNPSASINNDYFEDLKKYCFHR
SVNRETKVKFVHTSVHGVGHSFVQSAFKAFDLVPPEAVPEQKDPDPEFPTVKYPNPEEGK
GVLTLSFALADKTKARIVLANDPDADRLAVAEKQDSGEWRVFSGNELGALLGWWLFTSWK
EKNQDRSALKDTYMLSSTVSSKILRAIALKEGFHFEETLTGFKWMGNRAKQLIDQGKTVL
FAFEEAIGYMCCPFVLDKDGVSAAVISAELASFLATKNLSLSQQLKAIYVEYGYHITKAS
YFICHDQETIKKLFENLRNYDGKNNYPKACGKFEISAIRDLTTGYDDSQPDKKAVLPTSK
SSQMITFTFANGGVATMRTSGTEPKIKYYAELCAPPGNSDPEQLKKELNELVSAIEEHFF
QPQKYNLQPKAD
>sp|Q8N3Y7|RDHE2_HUMAN Epidermal retinol dehydrogenase 2 OS=Homo sapiens OX=9606 GN=SDR16C5 PE=1 SV=2

MSFNLQSSKKLFIFLGKSLFSLLEAMIFALLPKPRKNVAGEIVLITGAGSGLGRLLALQF
ARLGSVLVLWDINKEGNEETCKMAREAGATRVHAYTCDCSQKEGVYRVADQVKKEVGDVS
ILINNAGIVTGKKFLDCPDELMEKSFDVNFKAHLWTYKAFLPAMIANDHGHLVCISSSAG
LSGVNGLADYCASKFAAFGFAESVFVETFVQKQKGIKTTIVCPFFIKTGMFEGCTTGCPS
LLPILEPKYAVEKIVEAILQEKMYLYMPKLLYFMMFLKSFLPLKTGLLIADYLGILHAMD
GFVDQKKKL
>sp|P61160|ARP2_HUMAN Actin-related protein 2 OS=Homo sapiens OX=9606 GN=ACTR2 PE=1 SV=1
MDSQGRKVVVCDNGTGFVKCGYAGSNFPEHIFPALVGRPIIRSTTKVGNIEIKDLMVGDE
ASELRSMLEVNYPMENGIVRNWDDMKHLWDYTFGPEKLNIDTRNCKILLTEPPMNPTKNR
EKIVEVMFETYQFSGVYVAIQAVLTLYAQGLLTGVVVDSGDGVTHICPVYEGFSLPHLTR
RLDIAGRDITRYLIKLLLLRGYAFNHSADFETVRMIKEKLCYVGYNIEQEQKLALETTVL
VESYTLPDGRIIKVGGERFEAPEALFQPHLINVEGVGVAELLFNTIQAADIDTRSEFYKH
IVLSGGSTMYPGLPSRLERELKQLYLERVLKGDVEKLSKFKIRIEDPPRRKHMVFLGGAV
LADIMKDKDNFWMTRQEYQEKGVRVLEKLGVTVR
>sp|P51674|GPM6A_HUMAN Neuronal membrane glycoprotein M6-a OS=Homo sapiens OX=9606 GN=GPM6A PE=1 SV=2
MEENMEEGQTQKGCFECCIKCLGGIPYASLIATILLYAGVALFCGCGHEALSGTVNILQT
YFEMARTAGDTLDVFTMIDIFKYVIYGIAAAFFVYGILLMVEGFFTTGAIKDLYGDFKIT
TCGRCVSAWFIMLTYLFMLAWLGVTAFTSLPVYMYFNLWTICRNTTLVEGANLCLDLRQF
GIVTIGEEKKICTVSENFLRMCESTELNMTFHLFIVALAGAGAAVIAMVHYLMVLSANWA
YVKDACRMQKYEDIKSKEEQELHDIHSTRSKERLNAYT
>sp|P41250|GARS_HUMAN Glycine--tRNA ligase OS=Homo sapiens OX=9606 GN=GARS1 PE=1 SV=3
MPSPRPVLLRGARAALLLLLPPRLLARPSLLLRRSLSAASCPPISLPAAASRSSMDGAGA
EEVLAPLRLAVRQQGDLVRKLKEDKAPQVDVDKAVAELKARKRVLEAKELALQPKDDIVD
RAKMEDTLKRRFFYDQAFAIYGGVSGLYDFGPVGCALKNNIIQTWRQHFIQEEQILEIDC
TMLTPEPVLKTSGHVDKFADFMVKDVKNGECFRADHLLKAHLQKLMSDKKCSVEKKSEME
SVLAQLDNYGQQELADLFVNYNVKSPITGNDLSPPVSFNLMFKTFIGPGGNMPGYLRPET
AQGIFLNFKRLLEFNQGKLPFAAAQIGNSFRNEISPRSGLIRVREFTMAEIEHFVDPSEK
DHPKFQNVADLHLYLYSAKAQVSGQSARKMRLGDAVEQGVINNTVLGYFIGRIYLYLTKV
GISPDKLRFRQHMENEMAHYACDCWDAESKTSYGWIEIVGCADRSCYDLSCHARATKVPL
VAEKPLKEPKTVNVVQFEPSKGAIGKAYKKDAKLVMEYLAICDECYITEMEMLLNEKGEF
TIETEGKTFQLTKDMINVKRFQKTLYVEEVVPNVIEPSFGLGRIMYTVFEHTFHVREGDE
QRTFFSFPAVVAPFKCSVLPLSQNQEFMPFVKELSEALTRHGVSHKVDDSSGSIGRRYAR
TDEIGVAFGVTIDFDTVNKTPHTATLRDRDSMRQIRAEISELPSIVQDLANGNITWADVE
ARYPLFEGQETGKKETIEE
>sp|P48643|TCPE_HUMAN T-complex protein 1 subunit epsilon OS=Homo sapiens OX=9606 GN=CCT5 PE=1 SV=1
MASMGTLAFDEYGRPFLIIKDQDRKSRLMGLEALKSHIMAAKAVANTMRTSLGPNGLDKM
MVDKDGDVTVTNDGATILSMMDVDHQIAKLMVELSKSQDDEIGDGTTGVVVLAGALLEEA
EQLLDRGIHPIRIADGYEQAARVAIEHLDKISDSVLVDIKDTEPLIQTAKTTLGSKVVNS
CHRQMAEIAVNAVLTVADMERRDVDFELIKVEGKVGGRLEDTKLIKGVIVDKDFSHPQMP
KKVEDAKIAILTCPFEPPKPKTKHKLDVTSVEDYKALQKYEKEKFEEMIQQIKETGANLA
ICQWGFDDEANHLLLQNNLPAVRWVGGPEIELIAIATGGRIVPRFSELTAEKLGFAGLVQ
EISFGTTKDKMLVIEQCKNSRAVTIFIRGGNKMIIEEAKRSLHDALCVIRNLIRDNRVVY
GGGAAEISCALAVSQEADKCPTLEQYAMRAFADALEVIPMALSENSGMNPIQTMTEVRAR

QVKEMNPALGIDCLHKGTNDMKQQHVIETLIGKKQQISLATQMVRMILKIDDIRKPGESE
E
>sp|P08758|ANXA5_HUMAN Annexin A5 OS=Homo sapiens OX=9606 GN=ANXA5 PE=1 SV=2
MAQVLRGTVTDFPGFDERADAETLRKAMKGLGTDEESILTLLTSRSNAQRQEISAAFKTL
FGRDLLDDLKSELTGKFEKLIVALMKPSRLYDAYELKHALKGAGTNEKVLTEIIASRTPE
ELRAIKQVYEEEYGSSLEDDVVGDTSGYYQRMLVVLLQANRDPDAGIDEAQVEQDAQALF
QAGELKWGTDEEKFITIFGTRSVSHLRKVFDKYMTISGFQIEETIDRETSGNLEQLLLAV
VKSIRSIPAYLAETLYYAMKGAGTDDHTLIRVMVSRSEIDLFNIRKEFRKNFATSLYSMI
KGDTSGDYKKALLLLCGEDD
>sp|Q8IUE6|H2A2B_HUMAN Histone H2A type 2-B OS=Homo sapiens OX=9606 GN=H2AC21 PE=1 SV=3
MSGRGKQGGKARAKAKSRSSRAGLQFPVGRVHRLLRKGNYAERVGAGAPVYLAAVLEYLT
AEILELAGNAARDNKKTRIIPRHLQLAVRNDEELNKLLGGVTIAQGGVLPNIQAVLLPKK
TESHKPGKNK
>sp|P23526|SAHH_HUMAN Adenosylhomocysteinase OS=Homo sapiens OX=9606 GN=AHCY PE=1 SV=4
MSDKLPYKVADIGLAAWGRKALDIAENEMPGLMRMRERYSASKPLKGARIAGCLHMTVET
AVLIETLVTLGAEVQWSSCNIFSTQDHAAAAIAKAGIPVYAWKGETDEEYLWCIEQTLYF
KDGPLNMILDDGGDLTNLIHTKYPQLLPGIRGISEETTTGVHNLYKMMANGILKVPAINV
NDSVTKSKFDNLYGCRESLIDGIKRATDVMIAGKVAVVAGYGDVGKGCAQALRGFGARVI
ITEIDPINALQAAMEGYEVTTMDEACQEGNIFVTTTGCIDIILGRHFEQMKDDAIVCNIG
HFDVEIDVKWLNENAVEKVNIKPQVDRYRLKNGRRIILLAEGRLVNLGCAMGHPSFVMSN
SFTNQVMAQIELWTHPDKYPVGVHFLPKKLDEAVAEAHLGKLNVKLTKLTEKQAQYLGMS
CDGPFKPDHYRY
>sp|Q15836|VAMP3_HUMAN Vesicle-associated membrane protein 3 OS=Homo sapiens OX=9606 GN=VAMP3 PE=1 SV=3
MSTGPTAATGSNRRLQQTQNQVDEVVDIMRVNVDKVLERDQKLSELDDRADALQAGASQF
ETSAAKLKRKYWWKNCKMWAIGITVLVIFIIIIIVWVVSS
>sp|P63027|VAMP2_HUMAN Vesicle-associated membrane protein 2 OS=Homo sapiens OX=9606 GN=VAMP2 PE=1 SV=3
MSATAATAPPAAPAGEGGPPAPPPNLTSNRRLQQTQAQVDEVVDIMRVNVDKVLERDQKL
SELDDRADALQAGASQFETSAAKLKRKYWWKNLKMMIILGVICAIILIIIIVYFST
>sp|P23763|VAMP1_HUMAN Vesicle-associated membrane protein 1 OS=Homo sapiens OX=9606 GN=VAMP1 PE=1 SV=1
MSAPAQPPAEGTEGTAPGGGPPGPPPNMTSNRRLQQTQAQVEEVVDIIRVNVDKVLERDQ
KLSELDDRADALQAGASQFESSAAKLKRKYWWKNCKMMIMLGAICAIIVVVIVIYFFT
>sp|P61026|RAB10_HUMAN Ras-related protein Rab-10 OS=Homo sapiens OX=9606 GN=RAB10 PE=1 SV=1
MAKKTYDLLFKLLLIGDSGVGKTCVLFRFSDDAFNTTFISTIGIDFKIKTVELQGKKIKL
QIWDTAGQERFHTITTSYYRGAMGIMLVYDITNGKSFENISKWLRNIDEHANEDVERMLL
GNKCDMDDKRVVPKGKGEQIAREHGIRFFETSAKANINIEKAFLTLAEDILRKTPVKEPN
SENVDISSGGGVTGWKSKCC
>sp|P32926|DSG3_HUMAN Desmoglein-3 OS=Homo sapiens OX=9606 GN=DSG3 PE=1 SV=2
MMGLFPRTTGALAIFVVVILVHGELRIETKGQYDEEEMTMQQAKRRQKREWVKFAKPCRE
GEDNSKRNPIAKITSDYQATQKITYRISGVGIDQPPFGIFVVDKNTGDINITAIVDREET

PSFLITCRALNAQGLDVEKPLILTVKILDINDNPPVFSQQIFMGEIEENSASNSLVMILN
ATDADEPNHLNSKIAFKIVSQEPAGTPMFLLSRNTGEVRTLTNSLDREQASSYRLVVSGA
DKDGEGLSTQCECNIKVKDVNDNFPMFRDSQYSARIEENILSSELLRFQVTDLDEEYTDN
WLAVYFFTSGNEGNWFEIQTDPRTNEGILKVVKALDYEQLQSVKLSIAVKNKAEFHQSVI
SRYRVQSTPVTIQVINVREGIAFRPASKTFTVQKGISSKKLVDYILGTYQAIDEDTNKAA
SNVKYVMGRNDGGYLMIDSKTAEIKFVKNMNRDSTFIVNKTITAEVLAIDEYTGKTSTGT
VYVRVPDFNDNCPTAVLEKDAVCSSSPSVVVSARTLNNRYTGPYTFALEDQPVKLPAVWS
ITTLNATSALLRAQEQIPPGVYHISLVLTDSQNNRCEMPRSLTLEVCQCDNRGICGTSYP
TTSPGTRYGRPHSGRLGPAAIGLLLLGLLLLLLAPLLLLTCDCGAGSTGGVTGGFIPVPD
GSEGTIHQWGIEGAHPEDKEITNICVPPVTANGADFMESSEVCTNTYARGTAVEGTSGME
MTTKLGAATESGGAAGFATGTVSGAASGFGAATGVGICSSGQSGTMRTRHSTGGTNKDYA
DGAISMNFLDSYFSQKAFACAEEDDGQEANDCLLIYDNEGADATGSPVGSVGCCSFIADD
LDDSFLDSLGPKFKKLAEISLGVDGEGKEVQPPSKDSGYGIESCGHPIEVQQTGFVKCQT
LSGSQGASALSTSGSVQPAVSIPDPLQHGNYLVTETYSASGSLVQPSTAGFDPLLTQNVI
VTERVICPISSVPGNLAGPTQLRGSHTMLCTEDPCSRLI
>sp|P32320|CDD_HUMAN Cytidine deaminase OS=Homo sapiens OX=9606 GN=CDA PE=1 SV=2
MAQKRPACTLKPECVQQLLVCSQEAKKSAYCPYSHFPVGAALLTQEGRIFKGCNIENACY
PLGICAERTAIQKAVSEGYKDFRAIAIASDMQDDFISPCGACRQVMREFGTNWPVYMTKP
DGTYIVMTVQELLPSSFGPEDLQKTQ
>sp|P24534|EF1B_HUMAN Elongation factor 1-beta OS=Homo sapiens OX=9606 GN=EEF1B2 PE=1 SV=3
MGFGDLKSPAGLQVLNDYLADKSYIEGYVPSQADVAVFEAVSSPPPADLCHALRWYNHIK
SYEKEKASLPGVKKALGKYGPADVEDTTGSGATDSKDDDDIDLFGSDDEEESEEAKRLRE
ERLAQYESKKAKKPALVAKSSILLDVKPWDDETDMAKLEECVRSIQADGLVWGSSKLVPV
GYGIKKLQIQCVVEDDKVGTDMLEEQITAFEDYVQSMDVAAFNKI
>sp|P05386|RLA1_HUMAN 60S acidic ribosomal protein P1 OS=Homo sapiens OX=9606 GN=RPLP1 PE=1 SV=1
MASVSELACIYSALILHDDEVTVTEDKINALIKAAGVNVEPFWPGLFAKALANVNIGSLI
CNVGAGGPAPAAGAAPAGGPAPSTAAAPAEEKKVEAKKEESEESDDDMGFGLFD
>sp|P25787|PSA2_HUMAN Proteasome subunit alpha type-2 OS=Homo sapiens OX=9606 GN=PSMA2 PE=1 SV=2
MAERGYSFSLTTFSPSGKLVQIEYALAAVAGGAPSVGIKAANGVVLATEKKQKSILYDER
SVHKVEPITKHIGLVYSGMGPDYRVLVHRARKLAQQYYLVYQEPIPTAQLVQRVASVMQE
YTQSGGVRPFGVSLLICGWNEGRPYLFQSDPSGAYFAWKATAMGKNYVNGKTFLEKRYNE
DLELEDAIHTAILTLKESFEGQMTEDNIEVGICNEAGFRRLTPTEVKDYLAAIA
>sp|P54920|SNAA_HUMAN Alpha-soluble NSF attachment protein OS=Homo sapiens OX=9606 GN=NAPA PE=1 SV=3
MDNSGKEAEAMALLAEAERKVKNSQSFFSGLFGGSSKIEEACEIYARAANMFKMAKNWSA
AGNAFCQAAQLHLQLQSKHDAATCFVDAGNAFKKADPQEAINCLMRAIEIYTDMGRFTIA
AKHHISIAEIYETELVDIEKAIAHYEQSADYYKGEESNSSANKCLLKVAGYAALLEQYQK
AIDIYEQVGTNAMDSPLLKYSAKDYFFKAALCHFCIDMLNAKLAVQKYEELFPAFSDSRE
CKLMKKLLEAHEEQNVDSYTESVKEYDSISRLDQWLTTMLLRIKKTIQGDEEDLR
>sp|Q9H115|SNAB_HUMAN Beta-soluble NSF attachment protein OS=Homo sapiens OX=9606 GN=NAPB PE=1 SV=2
MDNAGKEREAVQLMAEAEKRVKASHSFLRGLFGGNTRIEEACEMYTRAANMFKMAKNWSA
AGNAFCQAAKLHMQLQSKHDSATSFVDAGNAYKKADPQEAINCLNAAIDIYTDMGRFTIA

AKHHITIAEIYETELVDIEKAIAHYEQSADYYKGEESNSSANKCLLKVAAYAAQLEQYQK
AIEIYEQVGANTMDNPLLKYSAKDYFFKAALCHFIVDELNAKLALEKYEEMFPAFTDSRE
CKLLKKLLEAHEEQNSEAYTEAVKEFDSISRLDQWLTTMLLRIKKSIQGDGEGDGDLK
>sp|P61313|RL15_HUMAN 60S ribosomal protein L15 OS=Homo sapiens OX=9606 GN=RPL15 PE=1 SV=2
MGAYKYIQELWRKKQSDVMRFLLRVRCWQYRQLSALHRAPRPTRPDKARRLGYKAKQGYV
IYRIRVRRGGRKRPVPKGATYGKPVHHGVNQLKFARSLQSVAEERAGRHCGALRVLNSYW
VGEDSTYKFFEVILIDPFHKAIRRNPDTQWITKPVHKHREMRGLTSAGRKSRGLGKGHKF
HHTIGGSRRAAWRRRNTLQLHRYR
>sp|Q9H1E1|RNAS7_HUMAN Ribonuclease 7 OS=Homo sapiens OX=9606 GN=RNASE7 PE=1 SV=2
MAPARAGFCPLLLLLLLGLWVAEIPVSAKPKGMTSSQWFKIQHMQPSPQACNSAMKNINK
HTKRCKDLNTFLHEPFSSVAATCQTPKIACKNGDKNCHQSHGAVSLTMCKLTSGKHPNCR
YKEKRQNKSYVVACKPPQKKDSQQFHLVPVHLDRVL
>sp|P49720|PSB3_HUMAN Proteasome subunit beta type-3 OS=Homo sapiens OX=9606 GN=PSMB3 PE=1 SV=2
MSIMSYNGGAVMAMKGKNCVAIAADRRFGIQAQMVTTDFQKIFPMGDRLYIGLAGLATDV
QTVAQRLKFRLNLYELKEGRQIKPYTLMSMVANLLYEKRFGPYYTEPVIAGLDPKTFKPF
ICSLDLIGCPMVTDDFVVSGTCAEQMYGMCESLWEPNMDPDHLFETISQAMLNAVDRDAV
SGMGVIVHIIEKDKITTRTLKARMD
>sp|Q15907|RB11B_HUMAN Ras-related protein Rab-11B OS=Homo sapiens OX=9606 GN=RAB11B PE=1 SV=4
MGTRDDEYDYLFKVVLIGDSGVGKSNLLSRFTRNEFNLESKSTIGVEFATRSIQVDGKTI
KAQIWDTAGQERYRAITSAYYRGAVGALLVYDIAKHLTYENVERWLKELRDHADSNIVIM
LVGNKSDLRHLRAVPTDEARAFAEKNNLSFIETSALDSTNVEEAFKNILTEIYRIVSQKQ
IADRAAHDESPGNNVVDISVPPTTDGQKPNKLQCCQNL
>sp|P62491|RB11A_HUMAN Ras-related protein Rab-11A OS=Homo sapiens OX=9606 GN=RAB11A PE=1 SV=3
MGTRDDEYDYLFKVVLIGDSGVGKSNLLSRFTRNEFNLESKSTIGVEFATRSIQVDGKTI
KAQIWDTAGQERYRAITSAYYRGAVGALLVYDIAKHLTYENVERWLKELRDHADSNIVIM
LVGNKSDLRHLRAVPTDEARAFAEKNGLSFIETSALDSTNVEAAFQTILTEIYRIVSQKQ
MSDRRENDMSPSNNVVPIHVPPTTENKPKVQCCQNI
>sp|P33764|S10A3_HUMAN Protein S100-A3 OS=Homo sapiens OX=9606 GN=S100A3 PE=1 SV=1
MARPLEQAVAAIVCTFQEYAGRCGDKYKLCQAELKELLQKELATWTPTEFRECDYNKFMS
VLDTNKDCEVDFVEYVRSLACLCLYCHEYFKDCPSEPPCSQ
>sp|A0A0C4DH25|KVD20_HUMAN Immunoglobulin kappa variable 3D-20 OS=Homo sapiens OX=9606 GN=IGKV3D-20 PE=3 SV=1
METPAQLLFLLLLWLPDTTGEIVLTQSPATLSLSPGERATLSCGASQSVSSSYLAWYQQK
PGLAPRLLIYDASSRATGIPDRFSGSGSGTDFTLTISRLEPEDFAVYYCQQYGSSP
>sp|A0A0A0MRZ8|KVD11_HUMAN Immunoglobulin kappa variable 3D-11 OS=Homo sapiens OX=9606 GN=IGKV3D-11 PE=3 SV=6
MEAPAQLLFLLLLWLPDTTGEIVLTQSPATLSLSPGERATLSCRASQSVSSYLAWYQQKP
GQAPRLLIYDASNRATGIPARFSGSGPGTDFTLTISSLEPEDFAVYYCQQRSNWH
>sp|P04433|KV311_HUMAN Immunoglobulin kappa variable 3-11 OS=Homo sapiens OX=9606 GN=IGKV3-11 PE=1 SV=1
MEAPAQLLFLLLLWLPDTTGEIVLTQSPATLSLSPGERATLSCRASQSVSSYLAWYQQKP

GQAPRLLIYDASNRATGIPARFSGSGSGTDFTLTISSLEPEDFAVYYCQQRSNWP
>sp|P35754|GLRX1_HUMAN Glutaredoxin-1 OS=Homo sapiens OX=9606 GN=GLRX PE=1 SV=2
MAQEFVNCKIQPGKVVVFIKPTCPYCRRAQEILSQLPIKQGLLEFVDITATNHTNEIQDY
LQQLTGARTVPRVFIGKDCIGGCSDLVSLQQSGELLTRLKQIGALQ
>sp|P26640|SYVC_HUMAN Valine--tRNA ligase OS=Homo sapiens OX=9606 GN=VARS1 PE=1 SV=4
MSTLYVSPHPDAFPSLRALIAARYGEAGEGPGWGGAHPRICLQPPPTSRTPFPPPRLPAL
EQGPGGLWVWGATAVAQLLWPAGLGGPGGSRAAVLVQQWVSYADTELIPAACGATLPALG
LRSSAQDPQAVLGALGRALSPLEEWLRLHTYLAGEAPTLADLAAVTALLLPFRYVLDPPA
RRIWNNVTRWFVTCVRQPEFRAVLGEVVLYSGARPLSHQPGPEAPALPKTAAQLKKEAKK
REKLEKFQQKQKIQQQQPPPGEKKPKPEKREKRDPGVITYDLPTPPGEKKDVSGPMPDSY
SPRYVEAAWYPWWEQQGFFKPEYGRPNVSAANPRGVFMMCIPPPNVTGSLHLGHALTNAI
QDSLTRWHRMRGETTLWNPGCDHAGIATQVVVEKKLWREQGLSRHQLGREAFLQEVWKWK
EEKGDRIYHQLKKLGSSLDWDRACFTMDPKLSAAVTEAFVRLHEEGIIYRSTRLVNWSCT
LNSAISDIEVDKKELTGRTLLSVPGYKEKVEFGVLVSFAYKVQGSDSDEEVVVATTRIET
MLGDVAVAVHPKDTRYQHLKGKNVIHPFLSRSLPIVFDEFVDMDFGTGAVKITPAHDQND
YEVGQRHGLEAISIMDSRGALINVPPPFLGLPRFEARKAVLVALKERGLFRGIEDNPMVV
PLCNRSKDVVEPLLRPQWYVRCGEMAQAASAAVTRGDLRILPEAHQRTWHAWMDNIREWC
ISRQLWWGHRIPAYFVTVSDPAVPPGEDPDGRYWVSGRNEAEAREKAAKEFGVSPDKISL
QQDEDVLDTWFSSGLFPLSILGWPNQSEDLSVFYPGTLLETGHDILFFWVARMVMLGLKL
TGRLPFREVYLHAIVRDAHGRKMSKSLGNVIDPLDVIYGISLQGLHNQLLNSNLDPSEVE
KAKEGQKADFPAGIPECGTDALRFGLCAYMSQGRDINLDVNRILGYRHFCNKLWNATKFA
LRGLGKGFVPSPTSQPGGHESLVDRWIRSRLTEAVRLSNQGFQAYDFPAVTTAQYSFWLY
ELCDVYLECLKPVLNGVDQVAAECARQTLYTCLDVGLRLLSPFMPFVTEELFQRLPRRMP
QAPPSLCVTPYPEPSECSWKDPEAEAALELALSITRAVRSLRADYNLTRIRPDCFLEVAD
EATGALASAVSGYVQALASAGVVAVLALGAPAPQGCAVALASDRCSIHLQLQGLVDPARE
LGKLQAKRVEAQRQAQRLRERRAASGYPVKVPLEVQEADEAKLQQTEAELRKVDEAIALF
QKML
>sp|Q07627|KRA11_HUMAN Keratin-associated protein 1-1 OS=Homo sapiens OX=9606 GN=KRTAP1-1 PE=1 SV=1
MACCQTSFCGFPSCSTSGTCGSSCCQPSCCETSSCQPRCCETSCCQPSCCQTSFCGFPSF
STGGTCDSSCCQPSCCETSCCQPSCYQTSSCGTGCGIGGGIGYGQEGSSGAVSTRIRWCR
PDCRVEGTCLPPCCVVSCTPPSCCQLHHAEASCCRPSYCGQSCCRPVCCCYCSEPTC
>sp|Q8IUG1|KRA13_HUMAN Keratin-associated protein 1-3 OS=Homo sapiens OX=9606 GN=KRTAP1-3 PE=1 SV=2
MTCCQTSFCGYPSCSTSGTCGSSCCQPSCCETSCCQPSCCQTSFCGFPSFSTSGTCSSSC
CQPSCCETSCCQPSCCQTSSCGTGCGIGGGIGYGQEGSSGAVSTRIRWCRPDCRVEGTCL
PPCCVVSCTPPTCCQLHHAEASCCRPSYCGQSCCRPVCCCYSCEPTC
>sp|P05141|ADT2_HUMAN ADP/ATP translocase 2 OS=Homo sapiens OX=9606 GN=SLC25A5 PE=1 SV=7
MTDAAVSFAKDFLAGGVAAAISKTAVAPIERVKLLLQVQHASKQITADKQYKGIIDCVVR
IPKEQGVLSFWRGNLANVIRYFPTQALNFAFKDKYKQIFLGGVDKRTQFWLYFAGNLASG
GAAGATSLCFVYPLDFARTRLAADVGKAGAEREFRGLGDCLVKIYKSDGIKGLYQGFNVS
VQGIIIYRAAYFGIYDTAKGMLPDPKNTHIVISWMIAQTVTAVAGLTSYPFDTVRRRMMM
QSGRKGTDIMYTGTLDCWRKIARDEGGKAFFKGAWSNVLRGMGGAFVLVLYDEIKKYT

>sp|Q9BYR6|KRA33_HUMAN Keratin-associated protein 3-3 OS=Homo sapiens OX=9606 GN=KRTAP3-3 PE=1 SV=1
MDCCASRGCSVPTGPATTICSSDKSCRCGVCLPSTCPHTVWLLEPTCCDNCPPPCHIPQP
CVPTCFLLNSCQPTPGLETLNLTTFTQPCCEPCLPRGC
>sp|P68871|HBB_HUMAN Hemoglobin subunit beta OS=Homo sapiens OX=9606 GN=HBB PE=1 SV=2
MVHLTPEEKSAVTALWGKVNVDEVGGEALGRLLVVYPWTQRFFESFGDLSTPDAVMGNPK
VKAHGKKVLGAFSDGLAHLDNLKGTFATLSELHCDKLHVDPENFRLLGNVLVCVLAHHFG
KEFTPPVQAAYQKVVAGVANALAHKYH
>sp|P46777|RL5_HUMAN 60S ribosomal protein L5 OS=Homo sapiens OX=9606 GN=RPL5 PE=1 SV=3
MGFVKVVKNKAYFKRYQVKFRRRREGKTDYYARKRLVIQDKNKYNTPKYRMIVRVTNRDI
ICQIAYARIEGDMIVCAAYAHELPKYGVKVGLTNYAAAYCTGLLLARRLLNRFGMDKIYE
GQVEVTGDEYNVESIDGQPGAFTCYLDAGLARTTTGNKVFGALKGAVDGGLSIPHSTKRF
PGYDSESKEFNAEVHRKHIMGQNVADYMRYLMEEDEDAYKKQFSQYIKNSVTPDMMEEMY
KKAHAAIRENPVYEKKPKKEVKKKRWNRPKMSLAQKKDRVAQKKASFLRAQERAAES
>sp|P02788|TRFL_HUMAN Lactotransferrin OS=Homo sapiens OX=9606 GN=LTF PE=1 SV=6
MKLVFLVLLFLGALGLCLAGRRRSVQWCAVSQPEATKCFQWQRNMRKVRGPPVSCIKRDS
PIQCIQAIAENRADAVTLDGGFIYEAGLAPYKLRPVAAEVYGTERQPRTHYYAVAVVKKG
GSFQLNELQGLKSCHTGLRRTAGWNVPIGTLRPFLNWTGPPEPIEAAVARFFSASCVPGA
DKGQFPNLCRLCAGTGENKCAFSSQEPYFSYSGAFKCLRDGAGDVAFIRESTVFEDLSDE
AERDEYELLCPDNTRKPVDKFKDCHLARVPSHAVVARSVNGKEDAIWNLLRQAQEKFGKD
KSPKFQLFGSPSGQKDLLFKDSAIGFSRVPPRIDSGLYLGSGYFTAIQNLRKSEEEVAAR
RARVVWCAVGEQELRKCNQWSGLSEGSVTCSSASTTEDCIALVLKGEADAMSLDGGYVYT
AGKCGLVPVLAENYKSQQSSDPDPNCVDRPVEGYLAVAVVRRSDTSLTWNSVKGKKSCHT
AVDRTAGWNIPMGLLFNQTGSCKFDEYFSQSCAPGSDPRSNLCALCIGDEQGENKCVPNS
NERYYGYTGAFRCLAENAGDVAFVKDVTVLQNTDGNNNEAWAKDLKLADFALLCLDGKRK
PVTEARSCHLAMAPNHAVVSRMDKVERLKQVLLHQQAKFGRNGSDCPDKFCLFQSETKNL
LFNDNTECLARLHGKTTYEKYLGPQYVAGITNLKKCSTSPLLEACEFLRK
>sp|Q8IUC1|KR111_HUMAN Keratin-associated protein 11-1 OS=Homo sapiens OX=9606 GN=KRTAP11-1 PE=1 SV=1
MSFNCSTRNCSSRPIGGRCIVPVAQVTTTSTTDADCLGGICLPSSFQTGSWLLDHCQETC
CEPTACQPTCYRRTSCVSNPCQVTCSRQTTCISNPCSTTYSRPLTFVSSGCQPLGGISSV
CQPVGGISTVCQPVGGVSTVCQPACGVSRTYQQSCVSSCRRTC
>sp|P31943|HNRH1_HUMAN Heterogeneous nuclear ribonucleoprotein H OS=Homo sapiens OX=9606 GN=HNRNPH1 PE=1 SV=4
MMLGTEGGEGFVVKVRGLPWSCSADEVQRFFSDCKIQNGAQGIRFIYTREGRPSGEAFVE
LESEDEVKLALKKDRETMGHRYVEVFKSNNVEMDWVLKHTGPNSPDTANDGFVRLRGLPF
GCSKEEIVQFFSGLEIVPNGITLPVDFQGRSTGEAFVQFASQEIAEKALKKHKERIGHRY
IEIFKSSRAEVRTHYDPPRKLMAMQRPGPYDRPGAGRGYNSIGRGAGFERMRRGAYGGGY
GGYDDYNGYNDGYGFGSDRFGRDLNYCFSGMSDHRYGDGGSTFQSTTGHCVHMRGLPYRA
TENDIYNFFSPLNPVRVHIEIGPDGRVTGEADVEFATHEDAVAAMSKDKANMQHRYVELF
LNSTAGASGGAYEHRYVELFLNSTAGASGGAYGSQMMGGMGLSNQSSYGGPASQQLSGGY
GGGYGGQSSMSGYDQVLQENSSDFQSNIA
>sp|P55795|HNRH2_HUMAN Heterogeneous nuclear ribonucleoprotein H2 OS=Homo sapiens OX=9606 GN=HNRNPH2 PE=1 SV=1

MMLSTEGREGFVVKVRGLPWSCSADEVMRFFSDCKIQNGTSGIRFIYTREGRPSGEAFVE
LESEEEVKLALKKDRETMGHRYVEVFKSNSVEMDWVLKHTGPNSPDTANDGFVRLRGLPF
GCSKEEIVQFFSGLEIVPNGMTLPVDFQGRSTGEAFVQFASQEIAEKALKKHKERIGHRY
IEIFKSSRAEVRTHYDPPRKLMAMQRPGPYDRPGAGRGYNSIGRGAGFERMRRGAYGGGY
GGYDDYGGYNDGYGFGSDRFGRDLNYCFSGMSDHRYGDGGSSFQSTTGHCVHMRGLPYRA
TENDIYNFFSPLNPMRVHIEIGPDGRVTGEADVEFATHEDAVAAMAKDKANMQHRYVELF
LNSTAGTSGGAYDHSYVELFLNSTAGASGGAYGSQMMGGMGLSNQSSYGGPASQQLSGGY
GGGYGGQSSMSGYDQVLQENSSDYQSNLA
>sp|P84085|ARF5_HUMAN ADP-ribosylation factor 5 OS=Homo sapiens OX=9606 GN=ARF5 PE=1 SV=2
MGLTVSALFSRIFGKKQMRILMVGLDAAGKTTILYKLKLGEIVTTIPTIGFNVETVEYKN
ICFTVWDVGGQDKIRPLWRHYFQNTQGLIFVVDSNDRERVQESADELQKMLQEDELRDAV
LLVFANKQDMPNAMPVSELTDKLGLQHLRSRTWYVQATCATQGTGLYDGLDWLSHELSKR
>sp|P61204|ARF3_HUMAN ADP-ribosylation factor 3 OS=Homo sapiens OX=9606 GN=ARF3 PE=1 SV=2
MGNIFGNLLKSLIGKKEMRILMVGLDAAGKTTILYKLKLGEIVTTIPTIGFNVETVEYKN
ISFTVWDVGGQDKIRPLWRHYFQNTQGLIFVVDSNDRERVNEAREELMRMLAEDELRDAV
LLVFANKQDLPNAMNAAEITDKLGLHSLRHRNWYIQATCATSGDGLYEGLDWLANQLKNK
K
>sp|P84077|ARF1_HUMAN ADP-ribosylation factor 1 OS=Homo sapiens OX=9606 GN=ARF1 PE=1 SV=2
MGNIFANLFKGLFGKKEMRILMVGLDAAGKTTILYKLKLGEIVTTIPTIGFNVETVEYKN
ISFTVWDVGGQDKIRPLWRHYFQNTQGLIFVVDSNDRERVNEAREELMRMLAEDELRDAV
LLVFANKQDLPNAMNAAEITDKLGLHSLRHRNWYIQATCATSGDGLYEGLDWLSNQLRNQ
K
>sp|P18085|ARF4_HUMAN ADP-ribosylation factor 4 OS=Homo sapiens OX=9606 GN=ARF4 PE=1 SV=3
MGLTISSLFSRLFGKKQMRILMVGLDAAGKTTILYKLKLGEIVTTIPTIGFNVETVEYKN
ICFTVWDVGGQDRIRPLWKHYFQNTQGLIFVVDSNDRERIQEVADELQKMLLVDELRDAV
LLLFANKQDLPNAMAISEMTDKLGLQSLRNRTWYVQATCATQGTGLYEGLDWLSNELSKR
>sp|P11413|G6PD_HUMAN Glucose-6-phosphate 1-dehydrogenase OS=Homo sapiens OX=9606 GN=G6PD PE=1 SV=4
MAEQVALSRTQVCGILREELFQGDAFHQSDTHIFIIMGASGDLAKKKIYPTIWWLFRDGL
LPENTFIVGYARSRLTVADIRKQSEPFFKATPEEKLKLEDFFARNSYVAGQYDDAASYQR
LNSHMNALHLGSQANRLFYLALPPTVYEAVTKNIHESCMSQIGWNRIIVEKPFGRDLQSS
DRLSNHISSLFREDQIYRIDHYLGKEMVQNLMVLRFANRIFGPIWNRDNIACVILTFKEP
FGTEGRGGYFDEFGIIRDVMQNHLLQMLCLVAMEKPASTNSDDVRDEKVKVLKCISEVQA
NNVVLGQYVGNPDGEGEATKGYLDDPTVPRGSTTATFAAVVLYVENERWDGVPFILRCGK
ALNERKAEVRLQFHDVAGDIFHQQCKRNELVIRVQPNEAVYTKMMTKKPGMFFNPEESEL
DLTYGNRYKNVKLPDAYERLILDVFCGSQMHFVRSDELREAWRIFTPLLHQIELEKPKPI
PYIYGSRGPTEADELMKRVGFQYEGTYKWVNPHKL
>sp|P40925|MDHC_HUMAN Malate dehydrogenase, cytoplasmic OS=Homo sapiens OX=9606 GN=MDH1 PE=1 SV=4
MSEPIRVLVTGAAGQIAYSLLYSIGNGSVFGKDQPIILVLLDITPMMGVLDGVLMELQDC
ALPLLKDVIATDKEDVAFKDLDVAILVGSMPRREGMERKDLLKANVKIFKSQGAALDKYA
KKSVKVIVVGNPANTNCLTASKSAPSIPKENFSCLTRLDHNRAKAQIALKLGVTANDVKN
VIIWGNHSSTQYPDVNHAKVKLQGKEVGVYEALKDDSWLKGEFVTTVQQRGAAVIKARKL

SSAMSAAKAICDHVRDIWFGTPEGEFVSMGVISDGNSYGVPDDLLYSFPVVIKNKTWKFV
EGLPINDFSREKMDLTAKELTEEKESAFEFLSSA
>sp|P01009|A1AT_HUMAN Alpha-1-antitrypsin OS=Homo sapiens OX=9606 GN=SERPINA1 PE=1 SV=3
MPSSVSWGILLLAGLCCLVPVSLAEDPQGDAAQKTDTSHHDQDHPTFNKITPNLAEFAFS
LYRQLAHQSNSTNIFFSPVSIATAFAMLSLGTKADTHDEILEGLNFNLTEIPEAQIHEGF
QELLRTLNQPDSQLQLTTGNGLFLSEGLKLVDKFLEDVKKLYHSEAFTVNFGDTEEAKKQ
INDYVEKGTQGKIVDLVKELDRDTVFALVNYIFFKGKWERPFEVKDTEEEDFHVDQVTTV
KVPMMKRLGMFNIQHCKKLSSWVLLMKYLGNATAIFFLPDEGKLQHLENELTHDIITKFL
ENEDRRSASLHLPKLSITGTYDLKSVLGQLGITKVFSNGADLSGVTEEAPLKLSKAVHKA
VLTIDEKGTEAAGAMFLEAIPMSIPPEVKFNKPFVFLMIEQNTKSPLFMGKVVNPTQK
>sp|P39023|RL3_HUMAN 60S ribosomal protein L3 OS=Homo sapiens OX=9606 GN=RPL3 PE=1 SV=2
MSHRKFSAPRHGSLGFLPRKRSSRHRGKVKSFPKDDPSKPVHLTAFLGYKAGMTHIVREV
DRPGSKVNKKEVVEAVTIVETPPMVVVGIVGYVETPRGLRTFKTVFAEHISDECKRRFYK
NWHKSKKKAFTKYCKKWQDEDGKKQLEKDFSSMKKYCQVIRVIAHTQMRLLPLRQKKAHL
MEIQVNGGTVAEKLDWARERLEQQVPVNQVFGQDEMIDVIGVTKGKGYKGVTSRWHTKKL
PRKTHRGLRKVACIGAWHPARVAFSVARAGQKGYHHRTEINKKIYKIGQGYLIKDGKLIK
NNASTDYDLSDKSINPLGGFVHYGEVTNDFVMLKGCVVGTKKRVLTLRKSLLVQTKRRAL
EKIDLKFIDTTSKFGHGRFQTMEEKKAFMGPLKKDRIAKEEGA
>sp|P04080|CYTB_HUMAN Cystatin-B OS=Homo sapiens OX=9606 GN=CSTB PE=1 SV=2
MMCGAPSATQPATAETQHIADQVRSQLEEKENKKFPVFKAVSFKSQVVAGTNYFIKVHVG
DEDFVHLRVFQSLPHENKPLTLSNYQTNKAKHDELTYF
>sp|Q9BYR7|KRA32_HUMAN Keratin-associated protein 3-2 OS=Homo sapiens OX=9606 GN=KRTAP3-2 PE=1 SV=1
MDCCASRSCSVPTGPATTICSSDKSCRCGVCLPSTCPHTVWLLEPICCDNCPPPCHIPQP
CVPTCFLLNSCQPTPGLETLNLTTFTQPCCEPCLPRGC
>sp|O60506|HNRPQ_HUMAN Heterogeneous nuclear ribonucleoprotein Q OS=Homo sapiens OX=9606 GN=SYNCRIP PE=1 SV=2
MATEHVNGNGTEEPMDTTSAVIHSENFQTLLDAGLPQKVAEKLDEIYVAGLVAHSDLDER
AIEALKEFNEDGALAVLQQFKDSDLSHVQNKSAFLCGVMKTYRQREKQGTKVADSSKGPD
EAKIKALLERTGYTLDVTTGQRKYGGPPPDSVYSGQQPSVGTEIFVGKIPRDLFEDELVP
LFEKAGPIWDLRLMMDPLTGLNRGYAFVTFCTKEAAQEAVKLYNNHEIRSGKHIGVCISV
ANNRLFVGSIPKSKTKEQILEEFSKVTEGLTDVILYHQPDDKKKNRGFCFLEYEDHKTAA
QARRRLMSGKVKVWGNVGTVEWADPIEDPDPEVMAKVKVLFVRNLANTVTEEILEKAFSQ
FGKLERVKKLKDYAFIHFDERDGAVKAMEEMNGKDLEGENIEIVFAKPPDQKRKERKAQR
QAAKNQMYDDYYYYGPPHMPPPTRGRGRGGRGGYGYPPDYYGYEDYYDYYGYDYHNYRGG
YEDPYYGYEDFQVGARGRGGRGARGAAPSRGRGAAPPRGRAGYSQRGGPGSARGVRGARG
GAQQQRGRGVRGARGGRGGNVGGKRKADGYNQPDSKRRQTNNQNWGSQPIAQQPLQGGDH
SGNYGYKSENQEFYQDTFGQQWK
>sp|O43390|HNRPR_HUMAN Heterogeneous nuclear ribonucleoprotein R OS=Homo sapiens OX=9606 GN=HNRNPR PE=1 SV=1
MANQVNGNAVQLKEEEEPMDTSSVTHTEHYKTLIEAGLPQKVAERLDEIFQTGLVAYVDL
DERAIDALREFNEEGALSVLQQFKESDLSHVQNKSAFLCGVMKTYRQREKQGSKVQESTK
GPDEAKIKALLERTGYTLDVTTGQRKYGGPPPDSVYSGVQPGIGTEVFVGKIPRDLYEDE
LVPLFEKAGPIWDLRLMMDPLSGQNRGYAFITFCGKEAAQEAVKLCDSYEIRPGKHLGVC

ISVANNRLFVGSIPKNKTKENILEEFSKVTEGLVDVILYHQPDDKKKNRGFCFLEYEDHK
SAAQARRRLMSGKVKVWGNVVTVEWADPVEEPDPEVMAKVKVLFVRNLATTVTEEILEKS
FSEFGKLERVKKLKDYAFVHFEDRGAAVKAMDEMNGKEIEGEEIEIVLAKPPDKKRKERQ
AARQASRSTAYEDYYYHPPPRMPPPIRGRGRGGGRGGYGYPPDYYGYEDYYDDYYGYDYH
DYRGGYEDPYYGYDDGYAVRGRGGGRGGRGAPPPPRGRGAPPPRGRAGYSQRGAPLGPPR
GSRGGRGGPAQQQRGRGSRGSRGNRGGNVGGKRKADGYNQPDSKRRQTNNQQNWGSQPIA
QQPLQQGGDYSGNYGYNNDNQEFYQDTYGQQWK
>sp|Q14974|IMB1_HUMAN Importin subunit beta-1 OS=Homo sapiens OX=9606 GN=KPNB1 PE=1 SV=2
MELITILEKTVSPDRLELEAAQKFLERAAVENLPTFLVELSRVLANPGNSQVARVAAGLQ
IKNSLTSKDPDIKAQYQQRWLAIDANARREVKNYVLQTLGTETYRPSSASQCVAGIACAE
IPVNQWPELIPQLVANVTNPNSTEHMKESTLEAIGYICQDIDPEQLQDKSNEILTAIIQG
MRKEEPSNNVKLAATNALLNSLEFTKANFDKESERHFIMQVVCEATQCPDTRVRVAALQN
LVKIMSLYYQYMETYMGPALFAITIEAMKSDIDEVALQGIEFWSNVCDEEMDLAIEASEA
AEQGRPPEHTSKFYAKGALQYLVPILTQTLTKQDENDDDDDWNPCKAAGVCLMLLATCCE
DDIVPHVLPFIKEHIKNPDWRYRDAAVMAFGCILEGPEPSQLKPLVIQAMPTLIELMKDP
SVVVRDTAAWTVGRICELLPEAAINDVYLAPLLQCLIEGLSAEPRVASNVCWAFSSLAEA
AYEAADVADDQEEPATYCLSSSFELIVQKLLETTDRPDGHQNNLRSSAYESLMEIVKNSA
KDCYPAVQKTTLVIMERLQQVLQMESHIQSTSDRIQFNDLQSLLCATLQNVLRKVQHQDA
LQISDVVMASLLRMFQSTAGSGGVQEDALMAVSTLVEVLGGEFLKYMEAFKPFLGIGLKN
YAEYQVCLAAVGLVGDLCRALQSNIIPFCDEVMQLLLENLGNENVHRSVKPQILSVFGDI
ALAIGGEFKKYLEVVLNTLQQASQAQVDKSDYDMVDYLNELRESCLEAYTGIVQGLKGDQ
ENVHPDVMLVQPRVEFILSFIDHIAGDEDHTDGVVACAAGLIGDLCTAFGKDVLKLVEAR
PMIHELLTEGRRSKTNKAKTLATWATKELRKLKNQA
>sp|P50990|TCPQ_HUMAN T-complex protein 1 subunit theta OS=Homo sapiens OX=9606 GN=CCT8 PE=1 SV=4
MALHVPKAPGFAQMLKEGAKHFSGLEEAVYRNIQACKELAQTTRTAYGPNGMNKMVINHL
EKLFVTNDAATILRELEVQHPAAKMIVMASHMQEQEVGDGTNFVLVFAGALLELAEELLR
IGLSVSEVIEGYEIACRKAHEILPNLVCCSAKNLRDIDEVSSLLRTSIMSKQYGNEVFLA
KLIAQACVSIFPDSGHFNVDNIRVCKILGSGISSSSVLHGMVFKKETEGDVTSVKDAKIA
VYSCPFDGMITETKGTVLIKTAEELMNFSKGEENLMDAQVKAIADTGANVVVTGGKVADM
ALHYANKYNIMLVRLNSKWDLRRLCKTVGATALPRLTPPVLEEMGHCDSVYLSEVGDTQV
VVFKHEKEDGAISTIVLRGSTDNLMDDIERAVDDGVNTFKVLTRDKRLVPGGGATEIELA
KQITSYGETCPGLEQYAIKKFAEAFEAIPRALAENSGVKANEVISKLYAVHQEGNKNVGL
DIEAEVPAVKDMLEAGILDTYLGKYWAIKLATNAAVTVLRVDQIIMAKPAGGPKPPSGKK
DWDDDQND
>sp|P62263|RS14_HUMAN 40S ribosomal protein S14 OS=Homo sapiens OX=9606 GN=RPS14 PE=1 SV=3
MAPRKGKEKKEEQVISLGPQVAEGENVFGVCHIFASFNDTFVHVTDLSGKETICRVTGGM
KVKADRDESSPYAAMLAAQDVAQRCKELGITALHIKLRATGGNRTKTPGPGAQSALRALA
RSGMKIGRIEDVTPIPSDSTRRKGGRRGRRL
>sp|P01619|KV320_HUMAN Immunoglobulin kappa variable 3-20 OS=Homo sapiens OX=9606 GN=IGKV3-20 PE=1 SV=2
METPAQLLFLLLLWLPDTTGEIVLTQSPGTLSLSPGERATLSCRASQSVSSSYLAWYQQK
PGQAPRLLIYGASSRATGIPDRFSGSGSGTDFTLTISRLEPEDFAVYYCQQYGSSP
>sp|P50452|SPB8_HUMAN Serpin B8 OS=Homo sapiens OX=9606 GN=SERPINB8 PE=1 SV=2

MDDLCEANGTFAISLFKILGEEDNSRNVFFSPMSISSALAMVFMGAKGSTAAQMSQALCL
YKDGDIHRGFQSLLSEVNRTGTQYLLRTANRLFGEKTCDFLPDFKEYCQKFYQAELEELS
FAEDTEECRKHINDWVAEKTEGKISEVLDAGTVDPLTKLVLVNAIYFKGKWNEQFDRKYT
RGMLFKTNEEKKTVQMMFKEAKFKMGYADEVHTQVLELPYVEEELSMVILLPDDNTDLAV
VEKALTYEKFKAWTNSEKLTKSKVQVFLPRLKLEESYDLEPFLRRLGMIDAFDEAKADFS
GMSTEKNVPLSKVAHKCFVEVNEEGTEAAAATAVVRNSRCSRMEPRFCADHPFLFFIRHH
KTNCILFCGRFSSP
>sp|P60903|S10AA_HUMAN Protein S100-A10 OS=Homo sapiens OX=9606 GN=S100A10 PE=1 SV=2
MPSQMEHAMETMMFTFHKFAGDKGYLTKEDLRVLMEKEFPGFLENQKDPLAVDKIMKDLD
QCRDGKVGFQSFFSLIAGLTIACNDYFVVHMKQKGKK
>sp|P84243|H33_HUMAN Histone H3.3 OS=Homo sapiens OX=9606 GN=H3-3B PE=1 SV=2
MARTKQTARKSTGGKAPRKQLATKAARKSAPSTGGVKKPHRYRPGTVALREIRRYQKSTE
LLIRKLPFQRLVREIAQDFKTDLRFQSAAIGALQEASEAYLVGLFEDTNLCAIHAKRVTI
MPKDIQLARRIRGERA
>sp|Q16695|H31T_HUMAN Histone H3.1t OS=Homo sapiens OX=9606 GN=H3-4 PE=1 SV=3
MARTKQTARKSTGGKAPRKQLATKVARKSAPATGGVKKPHRYRPGTVALREIRRYQKSTE
LLIRKLPFQRLMREIAQDFKTDLRFQSSAVMALQEACESYLVGLFEDTNLCVIHAKRVTI
MPKDIQLARRIRGERA
>sp|Q71DI3|H32_HUMAN Histone H3.2 OS=Homo sapiens OX=9606 GN=H3C13 PE=1 SV=3
MARTKQTARKSTGGKAPRKQLATKAARKSAPATGGVKKPHRYRPGTVALREIRRYQKSTE
LLIRKLPFQRLVREIAQDFKTDLRFQSSAVMALQEASEAYLVGLFEDTNLCAIHAKRVTI
MPKDIQLARRIRGERA
>sp|P68431|H31_HUMAN Histone H3.1 OS=Homo sapiens OX=9606 GN=H3C12 PE=1 SV=2
MARTKQTARKSTGGKAPRKQLATKAARKSAPATGGVKKPHRYRPGTVALREIRRYQKSTE
LLIRKLPFQRLVREIAQDFKTDLRFQSSAVMALQEACEAYLVGLFEDTNLCAIHAKRVTI
MPKDIQLARRIRGERA
>sp|Q6NXT2|H3C_HUMAN Histone H3.3C OS=Homo sapiens OX=9606 GN=H3-5 PE=1 SV=3
MARTKQTARKSTGGKAPRKQLATKAARKSTPSTCGVKPHRYRPGTVALREIRRYQKSTEL
LIRKLPFQRLVREIAQDFNTDLRFQSAAVGALQEASEAYLVGLLEDTNLCAIHAKRVTIM
PKDIQLARRIRGERA
>sp|P05091|ALDH2_HUMAN Aldehyde dehydrogenase, mitochondrial OS=Homo sapiens OX=9606 GN=ALDH2 PE=1 SV=2
MLRAAARFGPRLGRRLLSAAATQAVPAPNQQPEVFCNQIFINNEWHDAVSRKTFPTVNPS
TGEVICQVAEGDKEDVDKAVKAARAAFQLGSPWRRMDASHRGRLLNRLADLIERDRTYLA
ALETLDNGKPYVISYLVDLDMVLKCLRYYAGWADKYHGKTIPIDGDFFSYTRHEPVGVCG
QIIPWNFPLLMQAWKLGPALATGNVVVMKVAEQTPLTALYVANLIKEAGFPPGVVNIVPG
FGPTAGAAIASHEDVDKVAFTGSTEIGRVIQVAAGSSNLKRVTLELGGKSPNIIMSDADM
DWAVEQAHFALFFNQGQCCCAGSRTFVQEDIYDEFVERSVARAKSRVVGNPFDSKTEQGP
QVDETQFKKILGYINTGKQEGAKLLCGGGIAADRGYFIQPTVFGDVQDGMTIAKEEIFGP
VMQILKFKTIEEVVGRANNSTYGLAAAVFTKDLDKANYLSQALQAGTVWVNCYDVFGAQS
PFGGYKMSGSGRELGEYGLQAYTEVKTVTVKVPQKNS

>sp|P13693|TCTP_HUMAN Translationally-controlled tumor protein OS=Homo sapiens OX=9606 GN=TPT1 PE=1 SV=1
MIIYRDLISHDEMFSDIYKIREIADGLCLEVEGKMVSRTEGNIDDSLIGGNASAEGPEGE
GTESTVITGVDIVMNHHLQETSFTKEAYKKYIKDYMKSIKGKLEEQRPERVKPFMTGAAE
QIKHILANFKNYQFFIGENMNPDGMVALLDYREDGVTPYMIFFKDGLEMEKC
>sp|Q56UQ5|TPT1L_HUMAN TPT1-like protein OS=Homo sapiens OX=9606 PE=2 SV=2
METVIMITYWDLISHSEMFSDSYMSQEIADGLRLEVEGKIVSRTEGNIFDSLIGGNASAE
GPEGKGTESTVITGVDSVMNHHLQETSFTKEAYNKCIKDYMKSIKGKLEEQRPKRVKPFM
TGAAEQIKHILANFKNYQKT
>sp|P00738|HPT_HUMAN Haptoglobin OS=Homo sapiens OX=9606 GN=HP PE=1 SV=1
MSALGAVIALLLWGQLFAVDSGNDVTDIADDGCPKPPEIAHGYVEHSVRYQCKNYYKLRT
EGDGVYTLNDKKQWINKAVGDKLPECEADDGCPKPPEIAHGYVEHSVRYQCKNYYKLRTE
GDGVYTLNNEKQWINKAVGDKLPECEAVCGKPKNPANPVQRILGGHLDAKGSFPWQAKMV
SHHNLTTGATLINEQWLLTTAKNLFLNHSENATAKDIAPTLTLYVGKKQLVEIEKVVLHP
NYSQVDIGLIKLKQKVSVNERVMPICLPSKDYAEVGRVGYVSGWGRNANFKFTDHLKYVM
LPVADQDQCIRHYEGSTVPEKKTPKSPVGVQPILNEHTFCAGMSKYQEDTCYGDAGSAFA
VHDLEEDTWYATGILSFDKSCAVAEYGVYVKVTSIQDWVQKTIAEN
>sp|P00739|HPTR_HUMAN Haptoglobin-related protein OS=Homo sapiens OX=9606 GN=HPR PE=2 SV=2
MSDLGAVISLLLWGRQLFALYSGNDVTDISDDRFPKPPEIANGYVEHLFRYQCKNYYRLR
TEGDGVYTLNDKKQWINKAVGDKLPECEAVCGKPKNPANPVQRILGGHLDAKGSFPWQAK
MVSHHNLTTGATLINEQWLLTTAKNLFLNHSENATAKDIAPTLTLYVGKKQLVEIEKVVL
HPNYHQVDIGLIKLKQKVLVNERVMPICLPSKNYAEVGRVGYVSGWGQSDNFKLTDHLKY
VMLPVADQYDCITHYEGSTCPKWKAPKSPVGVQPILNEHTFCVGMSKYQEDTCYGDAGSA
FAVHDLEEDTWYAAGILSFDKSCAVAEYGVYVKVTSIQHWVQKTIAEN
>sp|Q9NZH6|IL37_HUMAN Interleukin-37 OS=Homo sapiens OX=9606 GN=IL37 PE=1 SV=1
MSFVGENSGVKMGSEDWEKDEPQCCLEDPAGSPLEPGPSLPTMNFVHTSPKVKNLNPKKF
SIHDQDHKVLVLDSGNLIAVPDKNYIRPEIFFALASSLSSASAEKGSPILLGVSKGEFCL
YCDKDKGQSHPSLQLKKEKLMKLAAQKESARRPFIFYRAQVGSWNMLESAAHPGWFICTS
CNCNEPVGVTDKFENRKHIEFSFQPVCKAEMSPSEVSD
>sp|O75608|LYPA1_HUMAN Acyl-protein thioesterase 1 OS=Homo sapiens OX=9606 GN=LYPLA1 PE=1 SV=1
MCGNNMSTPLPAIVPAARKATAAVIFLHGLGDTGHGWAEAFAGIRSSHIKYICPHAPVRP
VTLNMNVAMPSWFDIIGLSPDSQEDESGIKQAAENIKALIDQEVKNGIPSNRIILGGFSQ
GGALSLYTALTTQQKLAGVTALSCWLPLRASFPQGPIGGANRDISILQCHGDCDPLVPLM
FGSLTVEKLKTLVNPANVTFKTYEGMMHSSCQQEMMDVKQFIDKLLPPID
>sp|P37837|TALDO_HUMAN Transaldolase OS=Homo sapiens OX=9606 GN=TALDO1 PE=1 SV=2
MSSSPVKRQRMESALDQLKQFTTVVADTGDFHAIDEYKPQDATTNPSLILAAAQMPAYQE
LVEEAIAYGRKLGGSQEDQIKNAIDKLFVLFGAEILKKIPGRVSTEVDARLSFDKDAMVA
RARRLIELYKEAGISKDRILIKLSSTWEGIQAGKELEEQHGIHCNMTLLFSFAQAVACAE
AGVTLISPFVGRILDWHVANTDKKSYEPLEDPGVKSVTKIYNYYKKFSYKTIVMGASFRN
TGEIKALAGCDFLTISPKLLGELLQDNAKLVPVLSAKAAQASDLEKIHLDEKSFRWLHNE
DQMAVEKLSDGIRKFAADAVKLERMLTERMFNAENGK

>sp|P53621|COPA_HUMAN Coatomer subunit alpha OS=Homo sapiens OX=9606 GN=COPA PE=1 SV=2
MLTKFETKSARVKGLSFHPKRPWILTSLHNGVIQLWDYRMCTLIDKFDEHDGPVRGIDFH
KQQPLFVSGGDDYKIKVWNYKLRRCLFTLLGHLDYIRTTFFHHEYPWILSASDDQTIRVW
NWQSRTCVCVLTGHNHYVMCAQFHPTEDLVVSASLDQTVRVWDISGLRKKNLSPGAVESD
VRGITGVDLFGTTDAVVKHVLEGHDRGVNWAAFHPTMPLIVSGADDRQVKIWRMNESKAW
EVDTCRGHYNNVSCAVFHPRQELILSNSEDKSIRVWDMSKRTGVQTFRRDHDRFWVLAAH
PNLNLFAAGHDGGMIVFKLERERPAYAVHGNMLHYVKDRFLRQLDFNSSKDVAVMQLRSG
SKFPVFNMSYNPAENAVLLCTRASNLENSTYDLYTIPKDADSQNPDAPEGKRSSGLTAVW
VARNRFAVLDRMHSLLIKNLKNEITKKVQVPNCDEIFYAGTGNLLLRDADSITLFDVQQK
RTLASVKISKVKYVIWSADMSHVALLAKHAIVICNRKLDALCNIHENIRVKSGAWDESGV
FIYTTSNHIKYAVTTGDHGIIRTLDLPIYVTRVKGNNVYCLDRECRPRVLTIDPTEFKFK
LALINRKYDEVLHMVRNAKLVGQSIIAYLQKKGYPEVALHFVKDEKTRFSLALECGNIEI
ALEAAKALDDKNCWEKLGEVALLQGNHQIVEMCYQRTKNFDKLSFLYLITGNLEKLRKMM
KIAEIRKDMSGHYQNALYLGDVSERVRILKNCGQKSLAYLTAATHGLDEEAESLKETFDP
EKETIPDIDPNAKLLQPPAPIMPLDTNWPLLTVSKGFFEGTIASKGKGGALAADIDIDTV
GTEGWGEDAELQLDEDGFVEATEGLGDDALGKGQEEGGGWDVEEDLELPPELDISPGAAG
GAEDGFFVPPTKGTSPTQIWCNNSQLPVDHILAGSFETAMRLLHDQVGVIQFGPYKQLFL
QTYARGRTTYQALPCLPSMYGYPNRNWKDAGLKNGVPAVGLKLNDLIQRLQLCYQLTTVG
KFEEAVEKFRSILLSVPLLVVDNKQEIAEAQQLITICREYIVGLSVETERKKLPKETLEQ
QKRICEMAAYFTHSNLQPVHMILVLRTALNLFFKLKNFKTAATFARRLLELGPKPEVAQQ
TRKILSACEKNPTDAYQLNYDMHNPFDICAASYRPIYRGKPVEKCPLSGACYSPEFKGQI
CRVTTVTEIGKDVIGLRISPLQFR
>sp|P62249|RS16_HUMAN 40S ribosomal protein S16 OS=Homo sapiens OX=9606 GN=RPS16 PE=1 SV=2
MPSKGPLQSVQVFGRKKTATAVAHCKRGNGLIKVNGRPLEMIEPRTLQYKLLEPVLLLGK
ERFAGVDIRVRVKGGGHVAQIYAIRQSISKALVAYYQKYVDEASKKEIKDILIQYDRTLL
VADPRRCESKKFGGPGARARYQKSYR
>sp|E9PAV3|NACAM_HUMAN Nascent polypeptide-associated complex subunit alpha, muscle-specific form OS=Homo sapiens OX=9606 GN=NACA PE=1 SV=1
MPGEATETVPATEQELPQPQAETAVLPMSSALSVTAALGQPGPTLPPPCSPAPQQCPLSA
ANQASPFPSPSTIASTPLEVPFPQSSSGTALPLGTAPEAPTFLPNLIGPPISPAALALAS
PMIAPTLKGTPSSSAPLALVALAPHSVQKSSAFPPNLLTSPPSVAVAESGSVITLSAPIA
PSEPKTNLNKVPSEVVPNPKGTPSPPCIVSTVPYHCVTPMASIQSGVASLPQTTPTTTLA
IASPQVKDTTISSVLISPQNPGSLSLKGPVSPPAALSLSTQSLPVVTSSQKTAGPNTPPD
FPISLGSHLAPLHQSSFGSVQLLGQTGPSALSDPTVKTISVDHSSTGASYPSQRSVIPPL
PSRNEVVPATVAAFPVVAPSVDKGPSTISSITCSPSGSLNVATSFSLSPTTSLILKSSPN
ATYHYPLVAQMPVSSVGTTPLVVTNPCTIAAAPTTTFEVATCVSPPMSSGPISNIEPTSP
AALVMAPVAPKEPSTQVATTLRIPVSPPLPDPEDLKNLPSSVLVKFPTQKDLQTVPASLE
GAPFSPAQAGLTTKKDPTVLPLVQAAPKNSPSFQSTSSSPEIPLSPEATLAKKSLGEPLP
IGKPASSMTSPLGVNSSASVIKTDSYAGPDSAGPLLKSSLITPTVAAFPLESADPAGVAP
TTAKGTSTYTTTASPFLEGTVSLAPKNHPVKEGTLTTLPLVPTASENCPVAPSPQNTCAP
LATLVLAPEIPKSVPSPSLPPAGTPPGTKKVDGISHTSALAPVASSPKECPTEDSGASAT
ASSKGTLTYLADSPSPLGVSVSPQTKRPPTKKGSAGPDTPIGNLSSPVSPVEASFLPENS
LSFQGSKDSPATTHSPTPPSPKGAPTPSAVTPLSPKGVTLPPKETPTPSVVNLPFPKEGP
ATPAPKQAPALSMTSSSPKKARATPAPKGIPASPSPKGAPTPPAATPPSPKGGPATPSPK

WAPTPPAATPPSPKGGPATPSPKGAPTPPAATPPSPKGGPATPSPKGAPTPPAVTPPSPK
GSPAATPFPKGASTPPAATPPSPKGSPAATPLPKGAPTTPAATLPSPKGGPATPSLKGAP
TPPAATPPSPKGGPATPSPKGAPMPPAATPPSPKGGLATPPHKGAPTTPAATPPSPKGGL
ATPPPKGAPTTPAATPPSPKGGLATPPPKGAPTTPAATPPSPKGGLATPSPKGAPTTPAA
TPPSPKGGLATPSPKGAPTTPAATPPSPKGGLATPSPKGAPTTPAATPPSPKGGPATPPP
KGAPTPPAATPPSLKGGLATPPHKGAPNPAVVTPPSPKGGPATSPPKGAPTPPAATPPSP
KGSPGTPPPKGAPTPPAVTPPSPKGTPTLPATTPSSKGGPTTPSSKEGPTPPAATPSHKG
GPAMTPPSPKRGPAIPSPKGDPTSPAVIPLSPKKAPATPVTREGAATPSKGDLTPPAVTP
VSLKKAPATSAPKGGPATPSSKGDPTLPAVTPPSPKEPPAPKQVATSSSPKKAPATPAPM
GAPTLPAVIPSSPKEVPATPSSRRDPIAPTATLLSKKTPATLAPKEALIPPAMTVPSPKK
TPAIPTPKEAPATPSSKEASSPPAVTPSTYKGAPSPKELLIPPAVTSPSPKEAPTPPAVT
PPSPEKGPATPAPKGTPTSPPVTPSSLKDSPTSPASVTCKMGATVPQASKGLPAKKGPTA
LKEVLVAPAPESTPIITAPTRKGPQTKKSSATSPPICPDPSAKNGSKGPLSTVAPAPLLP
VQKDSSKTAKGKDASHSPKGPLAPPESKASTPLTAAAFEKVLPKPESASVSAAPSPPVSL
PLAPSPVPTLPPKQQFLPSSPGLVLESPSKPLAPADEDELLPLIPPEPISGGVPFQSVLV
NMPTPKSAGIPVPTPSAKQPVTKNNKGSGTESDSDESVPELEEQDSTQATTQQAQLAAAA
EIDEEPVSKAKQSRSEKKARKAMSKLGLRQVTGVTRVTIRKSKNILFVITKPDVYKSPAS
DTYIVFGEAKIEDLSQQAQLAAAEKFKVQGEAVSNIQENTQTPTVQEESEEEEVDETGVE
VKDIELVMSQANVSRAKAVRALKNNSNDIVNAIMELTM
>sp|Q13765|NACA_HUMAN Nascent polypeptide-associated complex subunit alpha OS=Homo sapiens OX=9606 GN=NACA PE=1 SV=1
MPGEATETVPATEQELPQPQAETGSGTESDSDESVPELEEQDSTQATTQQAQLAAAAEID
EEPVSKAKQSRSEKKARKAMSKLGLRQVTGVTRVTIRKSKNILFVITKPDVYKSPASDTY
IVFGEAKIEDLSQQAQLAAAEKFKVQGEAVSNIQENTQTPTVQEESEEEEVDETGVEVKD
IELVMSQANVSRAKAVRALKNNSNDIVNAIMELTM
>sp|Q92874|DNSL2_HUMAN Deoxyribonuclease-1-like 2 OS=Homo sapiens OX=9606 GN=DNASE1L2 PE=1 SV=1
MGGPRALLAALWALEAAGTAALRIGAFNIQSFGDSKVSDPACGSIIAKILAGYDLALVQE
VRDPDLSAVSALMEQINSVSEHEYSFVSSQPLGRDQYKEMYLFVYRKDAVSVVDTYLYPD
PEDVFSREPFVVKFSAPGTGERAPPLPSRRALTPPPLPAAAQNLVLIPLHAAPHQAVAEI
DALYDVYLDVIDKWGTDDMLFLGDFNADCSYVRAQDWAAIRLRSSEVFKWLIPDSADTTV
GNSDCAYDRIVACGARLRRSLKPQSATVHDFQEEFGLDQTQALAISDHFPVEVTLKFHR
>sp|P62888|RL30_HUMAN 60S ribosomal protein L30 OS=Homo sapiens OX=9606 GN=RPL30 PE=1 SV=2
MVAAKKTKKSLESINSRLQLVMKSGKYVLGYKQTLKMIRQGKAKLVILANNCPALRKSEI
EYYAMLAKTGVHHYSGNNIELGTACGKYYRVCTLAIIDPGDSDIIRSMPEQTGEK
>sp|P26373|RL13_HUMAN 60S ribosomal protein L13 OS=Homo sapiens OX=9606 GN=RPL13 PE=1 SV=4
MAPSRNGMVLKPHFHKDWQRRVATWFNQPARKIRRRKARQAKARRIAPRPASGPIRPIVR
CPTVRYHTKVRAGRGFSLEELRVAGIHKKVARTIGISVDPRRRNKSTESLQANVQRLKEY
RSKLILFPRKPSAPKKGDSSAEELKLATQLTGPVMPVRNVYKKEKARVITEEEKNFKAFA
SLRMARANARLFGIRAKRAKEAAEQDVEKKK
>sp|Q96DA0|ZG16B_HUMAN Zymogen granule protein 16 homolog B OS=Homo sapiens OX=9606 GN=ZG16B PE=1 SV=3
MGAQGAQESIKAMWRVPGTTRRPVTGESPGMHRPEAMLLLLTLALLGGPTWAGKMYGPGG
GKYFSTTEDYDHEITGLRVSVGLLLVKSVQVKLGDSWDVKLGALGGNTQEVTLQPGEYIT
KVFVAFQAFLRGMVMYTSKDRYFYFGKLDGQISSAYPSQEGQVLVGIYGQYQLLGIKSIG

FEWNYPLEEPTTEPPVNLTYSANSPVGR
>sp|Q9ULZ3|ASC_HUMAN Apoptosis-associated speck-like protein containing a CARD OS=Homo sapiens OX=9606 GN=PYCARD PE=1 SV=2
MGRARDAILDALENLTAEELKKFKLKLLSVPLREGYGRIPRGALLSMDALDLTDKLVSFY
LETYGAELTANVLRDMGLQEMAGQLQAATHQGSGAAPAGIQAPPQSAAKPGLHFIDQHRA
ALIARVTNVEWLLDALYGKVLTDEQYQAVRAEPTNPSKMRKLFSFTPAWNWTCKDLLLQA
LRESQSYLVEDLERS
>sp|P52565|GDIR1_HUMAN Rho GDP-dissociation inhibitor 1 OS=Homo sapiens OX=9606 GN=ARHGDIA PE=1 SV=3
MAEQEPTAEQLAQIAAENEEDEHSVNYKPPAQKSIQEIQELDKDDESLRKYKEALLGRVA
VSADPNVPNVVVTGLTLVCSSAPGPLELDLTGDLESFKKQSFVLKEGVEYRIKISFRVNR
EIVSGMKYIQHTYRKGVKIDKTDYMVGSYGPRAEEYEFLTPVEEAPKGMLARGSYSIKSR
FTDDDKTDHLSWEWNLTIKKDWKD
>sp|Q9Y2T3|GUAD_HUMAN Guanine deaminase OS=Homo sapiens OX=9606 GN=GDA PE=1 SV=1
MCAAQMPPLAHIFRGTFVHSTWTCPMEVLRDHLLGVSDSGKIVFLEEASQQEKLAKEWCF
KPCEIRELSHHEFFMPGLVDTHIHASQYSFAGSSIDLPLLEWLTKYTFPAEHRFQNIDFA
EEVYTRVVRRTLKNGTTTACYFATIHTDSSLLLADITDKFGQRAFVGKVCMDLNDTFPEY
KETTEESIKETERFVSEMLQKNYSRVKPIVTPRFSLSCSETLMGELGNIAKTRDLHIQSH
ISENRDEVEAVKNLYPSYKNYTSVYDKNNLLTNKTVMAHGCYLSAEELNVFHERGASIAH
CPNSNLSLSSGFLNVLEVLKHEVKIGLGTDVAGGYSYSMLDAIRRAVMVSNILLINKVNE
KSLTLKEVFRLATLGGSQALGLDGEIGNFEVGKEFDAILINPKASDSPIDLFYGDFFGDI
SEAVIQKFLYLGDDRNIEEVYVGGKQVVPFSSSV
>sp|Q14247|SRC8_HUMAN Src substrate cortactin OS=Homo sapiens OX=9606 GN=CTTN PE=1 SV=2
MWKASAGHAVSIAQDDAGADDWETDPDFVNDVSEKEQRWGAKTVQGSGHQEHINIHKLRE
NVFQEHQTLKEKELETGPKASHGYGGKFGVEQDRMDKSAVGHEYQSKLSKHCSQVDSVRG
FGGKFGVQMDRVDQSAVGFEYQGKTEKHASQKDYSSGFGGKYGVQADRVDKSAVGFDYQG
KTEKHESQRDYSKGFGGKYGIDKDKVDKSAVGFEYQGKTEKHESQKDYVKGFGGKFGVQT
DRQDKCALGWDHQEKLQLHESQKDYKTGFGGKFGVQSERQDSAAVGFDYKEKLAKHESQQ
DYSKGFGGKYGVQKDRMDKNASTFEDVTQVSSAYQKTVPVEAVTSKTSNIRANFENLAKE
KEQEDRRKAEAERAQRMAKERQEQEEARRKLEEQARAKTQTPPVSPAPQPTEERLPSSPV
YEDAASFKAELSYRGPVSGTEPEPVYSMEAADYREASSQQGLAYATEAVYESAEAPGHYP
AEDSTYDEYENDLGITAVALYDYQAAGDDEISFDPDDIITNIEMIDDGWWRGVCKGRYGL
FPANYVELRQ
>sp|P15531|NDKA_HUMAN Nucleoside diphosphate kinase A OS=Homo sapiens OX=9606 GN=NME1 PE=1 SV=1
MANCERTFIAIKPDGVQRGLVGEIIKRFEQKGFRLVGLKFMQASEDLLKEHYVDLKDRPF
FAGLVKYMHSGPVVAMVWEGLNVVKTGRVMLGETNPADSKPGTIRGDFCIQVGRNIIHGS
DSVESAEKEIGLWFHPEELVDYTSCAQNWIYE
>sp|Q6ZMR3|LDH6A_HUMAN L-lactate dehydrogenase A-like 6A OS=Homo sapiens OX=9606 GN=LDHAL6A PE=1 SV=1
MATIKSELIKNFAEEEAIHHNKISIVGTGSVGVACAISILLKGLSDELVLVDVDEGKLKG
ETMDLQHGSPFMKMPNIVSSKDYLVTANSNLVIITAGARQKKGETRLDLVQRNVSIFKLM
IPNITQYSPHCKLLIVTNPVDILTYVAWKLSGFPKNRVIGSGCNLDSARFRYFIGQRLGI
HSESCHGLILGEHGDSSVPVWSGVNIAGVPLKDLNPDIGTDKDPEQWENVHKKVISSGYE
MVKMKGYTSWGISLSVADLTESILKNLRRVHPVSTLSKGLYGINEDIFLSVPCILGENGI

TDLIKVKLTLEEEACLQKSAETLWEIQKELKL
>sp|P50914|RL14_HUMAN 60S ribosomal protein L14 OS=Homo sapiens OX=9606 GN=RPL14 PE=1 SV=4
MVFRRFVEVGRVAYVSFGPHAGKLVAIVDVIDQNRALVDGPCTQVRRQAMPFKCMQLTDF
ILKFPHSAHQKYVRQAWQKADINTKWAATRWAKKIEARERKAKMTDFDRFKVMKAKKMRN
RIIKNEVKKLQKAALLKASPKKAPGTKGTAAAAAAAAAAKVPAKKITAASKKAPAQKVPA
QKATGQKAAPAPKAQKGQKAPAQKAPAPKASGKKA
>sp|Q9NQ38|ISK5_HUMAN Serine protease inhibitor Kazal-type 5 OS=Homo sapiens OX=9606 GN=SPINK5 PE=1 SV=2
MKIATVSVLLPLALCLIQDAASKNEDQEMCHEFQAFMKNGKLFCPQDKKFFQSLDGIMFI
NKCATCKMILEKEAKSQKRARHLARAPKATAPTELNCDDFKKGERDGDFICPDYYEAVCG
TDGKTYDNRCALCAENAKTGSQIGVKSEGECKSSNPEQDVCSAFRPFVRDGRLGCTREND
PVLGPDGKTHGNKCAMCAELFLKEAENAKREGETRIRRNAEKDFCKEYEKQVRNGRLFCT
RESDPVRGPDGRMHGNKCALCAEIFKQRFSEENSKTDQNLGKAEEKTKVKREIVKLCSQY
QNQAKNGILFCTRENDPIRGPDGKMHGNLCSMCQAYFQAENEEKKKAEARARNKRESGKA
TSYAELCSEYRKLVRNGKLACTRENDPIQGPDGKVHGNTCSMCEVFFQAEEEEKKKKEGK
SRNKRQSKSTASFEELCSEYRKSRKNGRLFCTRENDPIQGPDGKMHGNTCSMCEAFFQQE
ERARAKAKREAAKEICSEFRDQVRNGTLICTREHNPVRGPDGKMHGNKCAMCASVFKLEE
EEKKNDKEEKGKVEAEKVKREAVQELCSEYRHYVRNGRLPCTRENDPIEGLDGKIHGNTC
SMCEAFFQQEAKEKERAEPRAKVKREAEKETCDEFRRLLQNGKLFCTRENDPVRGPDGKT
HGNKCAMCKAVFQKENEERKRKEEEDQRNAAGHGSSGGGGGNTQDECAEYREQMKNGRLS
CTRESDPVRDADGKSYNNQCTMCKAKLEREAERKNEYSRSRSNGTGSESGKDTCDEFRSQ
MKNGKLICTRESDPVRGPDGKTHGNKCTMCKEKLEREAAEKKKKEDEDRSNTGERSNTGE
RSNDKEDLCREFRSMQRNGKLICTRENNPVRGPYGKMHINKCAMCQSIFDREANERKKKD
EEKSSSKPSNNAKDECSEFRNYIRNNELICPRENDPVHGADGKFYTNKCYMCRAVFLTEA
LERAKLQEKPSHVRASQEEDSPDSFSSLDSEMCKDYRVLPRIGYLCPKDLKPVCGDDGQT
YNNPCMLCHENLIRQTNTHIRSTGKCEESSTPGTTAASMPPSDE
>sp|P13637|AT1A3_HUMAN Sodium/potassium-transporting ATPase subunit alpha-3 OS=Homo sapiens OX=9606 GN=ATP1A3 PE=1 SV=3
MGDKKDDKDSPKKNKGKERRDLDDLKKEVAMTEHKMSVEEVCRKYNTDCVQGLTHSKAQE
ILARDGPNALTPPPTTPEWVKFCRQLFGGFSILLWIGAILCFLAYGIQAGTEDDPSGDNL
YLGIVLAAVVIITGCFSYYQEAKSSKIMESFKNMVPQQALVIREGEKMQVNAEEVVVGDL
VEIKGGDRVPADLRIISAHGCKVDNSSLTGESEPQTRSPDCTHDNPLETRNITFFSTNCV
EGTARGVVVATGDRTVMGRIATLASGLEVGKTPIAIEIEHFIQLITGVAVFLGVSFFILS
LILGYTWLEAVIFLIGIIVANVPEGLLATVTVCLTLTAKRMARKNCLVKNLEAVETLGST
STICSDKTGTLTQNRMTVAHMWFDNQIHEADTTEDQSGTSFDKSSHTWVALSHIAGLCNR
AVFKGGQDNIPVLKRDVAGDASESALLKCIELSSGSVKLMRERNKKVAEIPFNSTNKYQL
SIHETEDPNDNRYLLVMKGAPERILDRCSTILLQGKEQPLDEEMKEAFQNAYLELGGLGE
RVLGFCHYYLPEEQFPKGFAFDCDDVNFTTDNLCFVGLMSMIDPPRAAVPDAVGKCRSAG
IKVIMVTGDHPITAKAIAKGVGIISEGNETVEDIAARLNIPVSQVNPRDAKACVIHGTDL
KDFTSEQIDEILQNHTEIVFARTSPQQKLIIVEGCQRQGAIVAVTGDGVNDSPALKKADI
GVAMGIAGSDVSKQAADMILLDDNFASIVTGVEEGRLIFDNLKKSIAYTLTSNIPEITPF
LLFIMANIPLPLGTITILCIDLGTDMVPAISLAYEAAESDIMKRQPRNPRTDKLVNERLI
SMAYGQIGMIQALGGFFSYFVILAENGFLPGNLVGIRLNWDDRTVNDLEDSYGQQWTYEQ
RKVVEFTCHTAFFVSIVVVQWADLIICKTRRNSVFQQGMKNKILIFGLFEETALAAFLSY
CPGMDVALRMYPLKPSWWFCAFPYSFLIFVYDEIRKLILRRNPGGWVEKETYY

>sp|P13798|ACPH_HUMAN Acylamino-acid-releasing enzyme OS=Homo sapiens OX=9606 GN=APEH PE=1 SV=4
MERQVLLSEPEEAAALYRGLSRQPALSAACLGPEVTTQYGGQYRTVHTEWTQRDLERMEN
IRFCRQYLVFHDGDSVVFAGPAGNSVETRGELLSRESPSGTMKAVLRKAGGTGPGEEKQF
LEVWEKNRKLKSFNLSALEKHGPVYEDDCFGCLSWSHSETHLLYVAEKKRPKAESFFQTK
ALDVSASDDEIARLKKPDQAIKGDQFVFYEDWGENMVSKSIPVLCVLDVESGNISVLEGV
PENVSPGQAFWAPGDAGVVFVGWWHEPFRLGIRFCTNRRSALYYVDLIGGKCELLSDDSL
AVSSPRLSPDQCRIVYLQYPSLIPHHQCSQLCLYDWYTKVTSVVVDVVPRQLGENFSGIY
CSLLPLGCWSADSQRVVFDSAQRSRQDLFAVDTQVGTVTSLTAGGSGGSWKLLTIDQDLM
VAQFSTPSLPPTLKVGFLPSAGKEQSVLWVSLEEAEPIPDIHWGIRVLQPPPEQENVQYA
GLDFEAILLQPGSPPDKTQVPMVVMPHGGPHSSFVTAWMLFPAMLCKMGFAVLLVNYRGS
TGFGQDSILSLPGNVGHQDVKDVQFAVEQVLQEEHFDASHVALMGGSHGGFISCHLIGQY
PETYRACVARNPVINIASMLGSTDIPDWCVVEAGFPFSSDCLPDLSVWAEMLDKSPIRYI
PQVKTPLLLMLGQEDRRVPFKQGMEYYRALKTRNVPVRLLLYPKSTHALSEVEVESDSFM
NAVLWLRTHLGS
>sp|P52209|6PGD_HUMAN 6-phosphogluconate dehydrogenase, decarboxylating OS=Homo sapiens OX=9606 GN=PGD PE=1 SV=3
MAQADIALIGLAVMGQNLILNMNDHGFVVCAFNRTVSKVDDFLANEAKGTKVVGAQSLKE
MVSKLKKPRRIILLVKAGQAVDDFIEKLVPLLDTGDIIIDGGNSEYRDTTRRCRDLKAKG
ILFVGSGVSGGEEGARYGPSLMPGGNKEAWPHIKTIFQGIAAKVGTGEPCCDWVGDEGAG
HFVKMVHNGIEYGDMQLICEAYHLMKDVLGMAQDEMAQAFEDWNKTELDSFLIEITANIL
KFQDTDGKHLLPKIRDSAGQKGTGKWTAISALEYGVPVTLIGEAVFARCLSSLKDERIQA
SKKLKGPQKFQFDGDKKSFLEDIRKALYASKIISYAQGFMLLRQAATEFGWTLNYGGIAL
MWRGGCIIRSVFLGKIKDAFDRNPELQNLLLDDFFKSAVENCQDSWRRAVSTGVQAGIPM
PCFTTALSFYDGYRHEMLPASLIQAQRDYFGAHTYELLAKPGQFIHTNWTGHGGTVSSSS
YNA
>sp|P05783|K1C18_HUMAN Keratin, type I cytoskeletal 18 OS=Homo sapiens OX=9606 GN=KRT18 PE=1 SV=2
MSFTTRSTFSTNYRSLGSVQAPSYGARPVSSAASVYAGAGGSGSRISVSRSTSFRGGMGS
GGLATGIAGGLAGMGGIQNEKETMQSLNDRLASYLDRVRSLETENRRLESKIREHLEKKG
PQVRDWSHYFKIIEDLRAQIFANTVDNARIVLQIDNARLAADDFRVKYETELAMRQSVEN
DIHGLRKVIDDTNITRLQLETEIEALKEELLFMKKNHEEEVKGLQAQIASSGLTVEVDAP
KSQDLAKIMADIRAQYDELARKNREELDKYWSQQIEESTTVVTTQSAEVGAAETTLTELR
RTVQSLEIDLDSMRNLKASLENSLREVEARYALQMEQLNGILLHLESELAQTRAEGQRQA
QEYEALLNIKVKLEAEIATYRRLLEDGEDFNLGDALDSSNSMQTIQKTTTRRIVDGKVVS
ETNDTKVLRH
>sp|Q9NQC3|RTN4_HUMAN Reticulon-4 OS=Homo sapiens OX=9606 GN=RTN4 PE=1 SV=2
MEDLDQSPLVSSSDSPPRPQPAFKYQFVREPEDEEEEEEEEEDEDEDLEELEVLERKPA
AGLSAAPVPTAPAAGAPLMDFGNDFVPPAPRGPLPAAPPVAPERQPSWDPSPVSSTVPAP
SPLSAAAVSPSKLPEDDEPPARPPPPPPASVSPQAEPVWTPPAPAPAAPPSTPAAPKRRG
SSGSVDETLFALPAASEPVIRSSAENMDLKEQPGNTISAGQEDFPSVLLETAASLPSLSP
LSAASFKEHEYLGNLSTVLPTEGTLQENVSEASKEVSEKAKTLLIDRDLTEFSELEYSEM
GSSFSVSPKAESAVIVANPREEIIVKNKDEEEKLVSNNILHNQQELPTALTKLVKEDEVV
SSEKAKDSFNEKRVAVEAPMREEYADFKPFERVWEVKDSKEDSDMLAAGGKIESNLESKV
DKKCFADSLEQTNHEKDSESSNDDTSFPSTPEGIKDRSGAYITCAPFNPAATESIATNIF
PLLGDPTSENKTDEKKIEEKKAQIVTEKNTSTKTSNPFLVAAQDSETDYVTTDNLTKVTE

EVVANMPEGLTPDLVQEACESELNEVTGTKIAYETKMDLVQTSEVMQESLYPAAQLCPSF
EESEATPSPVLPDIVMEAPLNSAVPSAGASVIQPSSSPLEASSVNYESIKHEPENPPPYE
EAMSVSLKKVSGIKEEIKEPENINAALQETEAPYISIACDLIKETKLSAEPAPDFSDYSE
MAKVEQPVPDHSELVEDSSPDSEPVDLFSDDSIPDVPQKQDETVMLVKESLTETSFESMI
EYENKEKLSALPPEGGKPYLESFKLSLDNTKDTLLPDEVSTLSKKEKIPLQMEELSTAVY
SNDDLFISKEAQIRETETFSDSSPIEIIDEFPTLISSKTDSFSKLAREYTDLEVSHKSEI
ANAPDGAGSLPCTELPHDLSLKNIQPKVEEKISFSDDFSKNGSATSKVLLLPPDVSALAT
QAEIESIVKPKVLVKEAEKKLPSDTEKEDRSPSAIFSAELSKTSVVDLLYWRDIKKTGVV
FGASLFLLLSLTVFSIVSVTAYIALALLSVTISFRIYKGVIQAIQKSDEGHPFRAYLESE
VAISEELVQKYSNSALGHVNCTIKELRRLFLVDDLVDSLKFAVLMWVFTYVGALFNGLTL
LILALISLFSVPVIYERHQAQIDHYLGLANKNVKDAMAKIQAKIPGLKRKAE
>sp|P28066|PSA5_HUMAN Proteasome subunit alpha type-5 OS=Homo sapiens OX=9606 GN=PSMA5 PE=1 SV=3
MFLTRSEYDRGVNTFSPEGRLFQVEYAIEAIKLGSTAIGIQTSEGVCLAVEKRITSPLME
PSSIEKIVEIDAHIGCAMSGLIADAKTLIDKARVETQNHWFTYNETMTVESVTQAVSNLA
LQFGEEDADPGAMSRPFGVALLFGGVDEKGPQLFHMDPSGTFVQCDARAIGSASEGAQSS
LQEVYHKSMTLKEAIKSSLIILKQVMEEKLNATNIELATVQPGQNFHMFTKEELEEVIKD
I
>sp|P47755|CAZA2_HUMAN F-actin-capping protein subunit alpha-2 OS=Homo sapiens OX=9606 GN=CAPZA2 PE=1 SV=3
MADLEEQLSDEEKVRIAAKFIIHAPPGEFNEVFNDVRLLLNNDNLLREGAAHAFAQYNLD
QFTPVKIEGYEDQVLITEHGDLGNGKFLDPKNRICFKFDHLRKEATDPRPCEVENAVESW
RTSVETALRAYVKEHYPNGVCTVYGKKIDGQQTIIACIESHQFQAKNFWNGRWRSEWKFT
ITPSTTQVVGILKIQVHYYEDGNVQLVSHKDIQDSLTVSNEVQTAKEFIKIVEAAENEYQ
TAISENYQTMSDTTFKALRRQLPVTRTKIDWNKILSYKIGKEMQNA
>sp|P25705|ATPA_HUMAN ATP synthase subunit alpha, mitochondrial OS=Homo sapiens OX=9606 GN=ATP5F1A PE=1 SV=1
MLSVRVAAAVVRALPRRAGLVSRNALGSSFIAARNFHASNTHLQKTGTAEMSSILEERIL
GADTSVDLEETGRVLSIGDGIARVHGLRNVQAEEMVEFSSGLKGMSLNLEPDNVGVVVFG
NDKLIKEGDIVKRTGAIVDVPVGEELLGRVVDALGNAIDGKGPIGSKTRRRVGLKAPGII
PRISVREPMQTGIKAVDSLVPIGRGQRELIIGDRQTGKTSIAIDTIINQKRFNDGSDEKK
KLYCIYVAIGQKRSTVAQLVKRLTDADAMKYTIVVSATASDAAPLQYLAPYSGCSMGEYF
RDNGKHALIIYDDLSKQAVAYRQMSLLLRRPPGREAYPGDVFYLHSRLLERAAKMNDAFG
GGSLTALPVIETQAGDVSAYIPTNVISITDGQIFLETELFYKGIRPAINVGLSVSRVGSA
AQTRAMKQVAGTMKLELAQYREVAAFAQFGSDLDAATQQLLSRGVRLTELLKQGQYSPMA
IEEQVAVIYAGVRGYLDKLEPSKITKFENAFLSHVVSQHQALLGTIRADGKISEQSDAKL
KEIVTNFLAGFEA
>sp|Q15019|SEPT2_HUMAN Septin-2 OS=Homo sapiens OX=9606 GN=SEPTIN2 PE=1 SV=1
MSKQQPTQFINPETPGYVGFANLPNQVHRKSVKKGFEFTLMVVGESGLGKSTLINSLFLT
DLYPERVIPGAAEKIERTVQIEASTVEIEERGVKLRLTVVDTPGYGDAINCRDCFKTIIS
YIDEQFERYLHDESGLNRRHIIDNRVHCCFYFISPFGHGLKPLDVAFMKAIHNKVNIVPV
IAKADTLTLKERERLKKRILDEIEEHNIKIYHLPDAESDEDEDFKEQTRLLKASIPFSVV
GSNQLIEAKGKKVRGRLYPWGVVEVENPEHNDFLKLRTMLITHMQDLQEVTQDLHYENFR
SERLKRGGRKVENEDMNKDQILLEKEAELRRMQEMIARMQAQMQMQMQGGDGDGGALGHH
V

>sp|P08123|CO1A2_HUMAN Collagen alpha-2(I) chain OS=Homo sapiens OX=9606 GN=COL1A2 PE=1 SV=7
MLSFVDTRTLLLLAVTLCLATCQSLQEETVRKGPAGDRGPRGERGPPGPPGRDGEDGPTG
PPGPPGPPGPPGLGGNFAAQYDGKGVGLGPGPMGLMGPRGPPGAAGAPGPQGFQGPAGEP
GEPGQTGPAGARGPAGPPGKAGEDGHPGKPGRPGERGVVGPQGARGFPGTPGLPGFKGIR
GHNGLDGLKGQPGAPGVKGEPGAPGENGTPGQTGARGLPGERGRVGAPGPAGARGSDGSV
GPVGPAGPIGSAGPPGFPGAPGPKGEIGAVGNAGPAGPAGPRGEVGLPGLSGPVGPPGNP
GANGLTGAKGAAGLPGVAGAPGLPGPRGIPGPVGAAGATGARGLVGEPGPAGSKGESGNK
GEPGSAGPQGPPGPSGEEGKRGPNGEAGSAGPPGPPGLRGSPGSRGLPGADGRAGVMGPP
GSRGASGPAGVRGPNGDAGRPGEPGLMGPRGLPGSPGNIGPAGKEGPVGLPGIDGRPGPI
GPAGARGEPGNIGFPGPKGPTGDPGKNGDKGHAGLAGARGAPGPDGNNGAQGPPGPQGVQ
GGKGEQGPPGPPGFQGLPGPSGPAGEVGKPGERGLHGEFGLPGPAGPRGERGPPGESGAA
GPTGPIGSRGPSGPPGPDGNKGEPGVVGAVGTAGPSGPSGLPGERGAAGIPGGKGEKGEP
GLRGEIGNPGRDGARGAPGAVGAPGPAGATGDRGEAGAAGPAGPAGPRGSPGERGEVGPA
GPNGFAGPAGAAGQPGAKGERGAKGPKGENGVVGPTGPVGAAGPAGPNGPPGPAGSRGDG
GPPGMTGFPGAAGRTGPPGPSGISGPPGPPGPAGKEGLRGPRGDQGPVGRTGEVGAVGPP
GFAGEKGPSGEAGTAGPPGTPGPQGLLGAPGILGLPGSRGERGLPGVAGAVGEPGPLGIA
GPPGARGPPGAVGSPGVNGAPGEAGRDGNPGNDGPPGRDGQPGHKGERGYPGNIGPVGAA
GAPGPHGPVGPAGKHGNRGETGPSGPVGPAGAVGPRGPSGPQGIRGDKGEPGEKGPRGLP
GLKGHNGLQGLPGIAGHHGDQGAPGSVGPAGPRGPAGPSGPAGKDGRTGHPGTVGPAGIR
GPQGHQGPAGPPGPPGPPGPPGVSGGGYDFGYDGDFYRADQPRSAPSLRPKDYEVDATLK
SLNNQIETLLTPEGSRKNPARTCRDLRLSHPEWSSGYYWIDPNQGCTMDAIKVYCDFSTG
ETCIRAQPENIPAKNWYRSSKDKKHVWLGETINAGSQFEYNVEGVTSKEMATQLAFMRLL
ANYASQNITYHCKNSIAYMDEETGNLKKAVILQGSNDVELVAEGNSRFTYTVLVDGCSKK
TNEWGKTIIEYKTNKPSRLPFLDIAPLDIGGADQEFFVDIGPVCFK
>sp|Q9NZD2|GLTP_HUMAN Glycolipid transfer protein OS=Homo sapiens OX=9606 GN=GLTP PE=1 SV=3
MALLAEHLLKPLPADKQIETGPFLEAVSHLPPFFDCLGSPVFTPIKADISGNITKIKAVY
DTNPAKFRTLQNILEVEKEMYGAEWPKVGATLALMWLKRGLRFIQVFLQSICDGERDENH
PNLIRVNATKAYEMALKKYHGWIVQKIFQAALYAAPYKSDFLKALSKGQNVTEEECLEKI
RLFLVNYTATIDVIYEMYTQMNAELNYKV
>sp|P63151|2ABA_HUMAN Serine/threonine-protein phosphatase 2A 55 kDa regulatory subunit B alpha isoform OS=Homo sapiens OX=9606 GN=PPP2R2A PE=1 SV=1
MAGAGGGNDIQWCFSQVKGAVDDDVAEADIISTVEFNHSGELLATGDKGGRVVIFQQEQE
NKIQSHSRGEYNVYSTFQSHEPEFDYLKSLEIEEKINKIRWLPQKNAAQFLLSTNDKTIK
LWKISERDKRPEGYNLKEEDGRYRDPTTVTTLRVPVFRPMDLMVEASPRRIFANAHTYHI
NSISINSDYETYLSADDLRINLWHLEITDRSFNIVDIKPANMEELTEVITAAEFHPNSCN
TFVYSSSKGTIRLCDMRASALCDRHSKLFEEPEDPSNRSFFSEIISSISDVKFSHSGRYM
MTRDYLSVKIWDLNMENRPVETYQVHEYLRSKLCSLYENDCIFDKFECCWNGSDSVVMTG
SYNNFFRMFDRNTKRDITLEASRENNKPRTVLKPRKVCASGKRKKDEISVDSLDFNKKIL
HTAWHPKENIIAVATTNNLYIFQDKVN
>sp|P62829|RL23_HUMAN 60S ribosomal protein L23 OS=Homo sapiens OX=9606 GN=RPL23 PE=1 SV=1
MSKRGRGGSSGAKFRISLGLPVGAVINCADNTGAKNLYIISVKGIKGRLNRLPAAGVGDM
VMATVKKGKPELRKKVHPAVVIRQRKSYRRKDGVFLYFEDNAGVIVNNKGEMKGSAITGP
VAKECADLWPRIASNAGSIA

>sp|P62269|RS18_HUMAN 40S ribosomal protein S18 OS=Homo sapiens OX=9606 GN=RPS18 PE=1 SV=3
MSLVIPEKFQHILRVLNTNIDGRRKIAFAITAIKGVGRRYAHVVLRKADIDLTKRAGELT
EDEVERVITIMQNPRQYKIPDWFLNRQKDVKDGKYSQVLANGLDNKLREDLERLKKIRAH
RGLRHFWGLRVRGQHTKTTGRRGRTVGVSKKK
>sp|Q9H299|SH3L3_HUMAN SH3 domain-binding glutamic acid-rich-like protein 3 OS=Homo sapiens OX=9606 GN=SH3BGRL3 PE=1 SV=1
MSGLRVYSTSVTGSREIKSQQSEVTRILDGKRIQYQLVDISQDNALRDEMRALAGNPKAT
PPQIVNGDQYCGDYELFVEAVEQNTLQEFLKLA
>sp|Q9GZP4|PITH1_HUMAN PITH domain-containing protein 1 OS=Homo sapiens OX=9606 GN=PITHD1 PE=1 SV=1
MSHGHSHGGGGCRCAAEREEPPEQRGLAYGLYLRIDLERLQCLNESREGSGRGVFKPWEE
RTDRSKFVESDADEELLFNIPFTGNVKLKGIIIMGEDDDSHPSEMRLYKNIPQMSFDDTE
REPDQTFSLNRDLTGELEYATKISRFSNVYHLSIHISKNFGADTTKVFYIGLRGEWTELR
RHEVTICNYEASANPADHRVHQVTPQTHFIS
>sp|Q13561|DCTN2_HUMAN Dynactin subunit 2 OS=Homo sapiens OX=9606 GN=DCTN2 PE=1 SV=4
MADPKYADLPGIARNEPDVYETSDLPEDDQAEFDAEELTSTSVEHIIVNPNAAYDKFKDK
RVGTKGLDFSDRIGKTKRTGYESGEYEMLGEGLGVKETPQQKYQRLLHEVQELTTEVEKI
KTTVKESATEEKLTPVLLAKQLAALKQQLVASHLEKLLGPDAAINLTDPDGALAKRLLLQ
LEATKNSKGGSGGKTTGTPPDSSLVTYELHSRPEQDKFSQAAKVAELEKRLTELETAVRC
DQDAQNPLSAGLQGACLMETVELLQAKVSALDLAVLDQVEARLQSVLGKVNEIAKHKASV
EDADTQSKVHQLYETIQRWSPIASTLPELVQRLVTIKQLHEQAMQFGQLLTHLDTTQQMI
ANSLKDNTTLLTQVQTTMRENLATVEGNFASIDERMKKLGK
>sp|Q9BQ50|TREX2_HUMAN Three prime repair exonuclease 2 OS=Homo sapiens OX=9606 GN=TREX2 PE=1 SV=2
MSEAPRAETFVFLDLEATGLPSVEPEIAELSLFAVHRSSLENPEHDESGALVLPRVLDKL
TLCMCPERPFTAKASEITGLSSEGLARCRKAGFDGAVVRTLQAFLSRQAGPICLVAHNGF
DYDFPLLCAELRRLGARLPRDTVCLDTLPALRGLDRAHSHGTRARGRQGYSLGSLFHRYF
RAEPSAAHSAEGDVHTLLLIFLHRAAELLAWADEQARGWAHIEPMYLPPDDPSLEA
>sp|P10619|PPGB_HUMAN Lysosomal protective protein OS=Homo sapiens OX=9606 GN=CTSA PE=1 SV=2
MIRAAPPPLFLLLLLLLLLVSWASRGEAAPDQDEIQRLPGLAKQPSFRQYSGYLKGSGSK
HLHYWFVESQKDPENSPVVLWLNGGPGCSSLDGLLTEHGPFLVQPDGVTLEYNPYSWNLI
ANVLYLESPAGVGFSYSDDKFYATNDTEVAQSNFEALQDFFRLFPEYKNNKLFLTGESYA
GIYIPTLAVLVMQDPSMNLQGLAVGNGLSSYEQNDNSLVYFAYYHGLLGNRLWSSLQTHC
CSQNKCNFYDNKDLECVTNLQEVARIVGNSGLNIYNLYAPCAGGVPSHFRYEKDTVVVQD
LGNIFTRLPLKRMWHQALLRSGDKVRMDPPCTNTTAASTYLNNPYVRKALNIPEQLPQWD
MCNFLVNLQYRRLYRSMNSQYLKLLSSQKYQILLYNGDVDMACNFMGDEWFVDSLNQKME
VQRRPWLVKYGDSGEQIAGFVKEFSHIAFLTIKGAGHMVPTDKPLAAFTMFSRFLNKQPY
>sp|Q6ZRV2|FA83H_HUMAN Protein FAM83H OS=Homo sapiens OX=9606 GN=FAM83H PE=1 SV=3
MARRSQSSSQGDNPLAPGYLPPHYKEYYRLAVDALAEGGSEAYSRFLATEGAPDFLCPEE
LEHVSRHLRPPQYVTREPPEGSLLDVDMDGSSGTYWPVNSDQAVPELDLGWPLTFGFQGT
EVTTLVQPPPPDSPSIKDEARRMIRSAQQVVAVVMDMFTDVDLLSEVLEAAARRVPVYIL
LDEMNAQHFLDMADKCRVNLQHVDFLRVRTVAGPTYYCRTGKSFKGHVKEKFLLVDCAVV
MSGSYSFMWSFEKIHRSLAHVFQGELVSSFDEEFRILFAQSEPLVPSAAALARMDAYALA

PYAGAGPLVGVPGVGAPTPFSFPKRAHLLFPPPREEGLGFPSFLDPDRHFLSAFRREEPP
RMPGGALEPHAGLRPLSRRLEAEAGPAGELAGARGFFQARHLEMDAFKRHSFATEGAGAV
ENFAAARQVSRQTFLSHGDDFRFQTSHFHRDQLYQQQYQWDPQLTPARPQGLFEKLRGGR
AGFADPDDFTLGAGPRFPELGPDGHQRLDYVPSSASREVRHGSDPAFAPGPRGLEPSGAP
RPNLTQRFPCQAAARPGPDPAPEAEPERRGGPEGRAGLRRWRLASYLSGCHGEDGGDDGL
PAPMEAEAYEDDVLAPGGRAPAGDLLPSAFRVPAAFPTKVPVPGPGSGGNGPEREGPEEP
GLAKQDSFRSRLNPLVQRSSRLRSSLIFSTSQAEGAAGAAAATEKVQLLHKEQTVSETLG
PGGEAVRSAASTKVAELLEKYKGPARDPGGGAGAITVASHSKAVVSQAWREEVAAPGAVG
GERRSLESCLLDLRDSFAQQLHQEAERQPGAASLTAAQLLDTLGRSGSDRLPSRFLSAQS
HSTSPQGLDSPLPLEGSGAHQVLHNESKGSPTSAYPERKGSPTPGFSTRRGSPTTGFIEQ
KGSPTSAYPERRGSPVPPVPERRSSPVPPVPERRGSLTLTISGESPKAGPAEEGPSGPME
VLRKGSLRLRQLLSPKGERRMEDEGGFPVPQENGQPESPRRLSLGQGDSTEAATEERGPR
ARLSSATANALYSSNLRDDTKAILEQISAHGQKHRAVPAPSPGPTHNSPELGRPPAAGVL
APDMSDKDKCSAIFRSDSLGTQGRLSRTLPASAEERDRLLRRMESMRKEKRVYSRFEVFC
KKEEASSPGAGEGPAEEGTRDSKVGKFVPKILGTFKSKK
>sp|P62333|PRS10_HUMAN 26S proteasome regulatory subunit 10B OS=Homo sapiens OX=9606 GN=PSMC6 PE=1 SV=1
MADPRDKALQDYRKKLLEHKEIDGRLKELREQLKELTKQYEKSENDLKALQSVGQIVGEV
LKQLTEEKFIVKATNGPRYVVGCRRQLDKSKLKPGTRVALDMTTLTIMRYLPREVDPLVY
NMSHEDPGNVSYSEIGGLSEQIRELREVIELPLTNPELFQRVGIIPPKGCLLYGPPGTGK
TLLARAVASQLDCNFLKVVSSSIVDKYIGESARLIREMFNYARDHQPCIIFMDEIDAIGG
RRFSEGTSADREIQRTLMELLNQMDGFDTLHRVKMIMATNRPDTLDPALLRPGRLDRKIH
IDLPNEQARLDILKIHAGPITKHGEIDYEAIVKLSDGFNGADLRNVCTEAGMFAIRADHD
FVVQEDFMKAVRKVADSKKLESKLDYKPV
>sp|P53634|CATC_HUMAN Dipeptidyl peptidase 1 OS=Homo sapiens OX=9606 GN=CTSC PE=1 SV=2
MGAGPSLLLAALLLLLSGDGAVRCDTPANCTYLDLLGTWVFQVGSSGSQRDVNCSVMGPQ
EKKVVVYLQKLDTAYDDLGNSGHFTIIYNQGFEIVLNDYKWFAFFKYKEEGSKVTTYCNE
TMTGWVHDVLGRNWACFTGKKVGTASENVYVNIAHLKNSQEKYSNRLYKYDHNFVKAINA
IQKSWTATTYMEYETLTLGDMIRRSGGHSRKIPRPKPAPLTAEIQQKILHLPTSWDWRNV
HGINFVSPVRNQASCGSCYSFASMGMLEARIRILTNNSQTPILSPQEVVSCSQYAQGCEG
GFPYLIAGKYAQDFGLVEEACFPYTGTDSPCKMKEDCFRYYSSEYHYVGGFYGGCNEALM
KLELVHHGPMAVAFEVYDDFLHYKKGIYHHTGLRDPFNPFELTNHAVLLVGYGTDSASGM
DYWIVKNSWGTGWGENGYFRIRRGTDECAIESIAVAATPIPKL
>sp|O75367|H2AY_HUMAN Core histone macro-H2A.1 OS=Homo sapiens OX=9606 GN=MACROH2A1 PE=1 SV=4
MSSRGGKKKSTKTSRSAKAGVIFPVGRMLRYIKKGHPKYRIGVGAPVYMAAVLEYLTAEI
LELAGNAARDNKKGRVTPRHILLAVANDEELNQLLKGVTIASGGVLPNIHPELLAKKRGS
KGKLEAIITPPPAKKAKSPSQKKPVSKKAGGKKGARKSKKKQGEVSKAASADSTTEGTPA
DGFTVLSTKSLFLGQKLNLIHSEISNLAGFEVEAIINPTNADIDLKDDLGNTLEKKGGKE
FVEAVLELRKKNGPLEVAGAAVSAGHGLPAKFVIHCNSPVWGADKCEELLEKTVKNCLAL
ADDKKLKSIAFPSIGSGRNGFPKQTAAQLILKAISSYFVSTMSSSIKTVYFVLFDSESIG
IYVQEMAKLDAN
>sp|P16402|H13_HUMAN Histone H1.3 OS=Homo sapiens OX=9606 GN=H1-3 PE=1 SV=2
MSETAPLAPTIPAPAEKTPVKKKAKKAGATAGKRKASGPPVSELITKAVAASKERSGVSL
AALKKALAAAGYDVEKNNSRIKLGLKSLVSKGTLVQTKGTGASGSFKLNKKAASGEGKPK

AKKAGAAKPRKPAGAAKKPKKVAGAATPKKSIKKTPKKVKKPATAAGTKKVAKSAKKVKT
PQPKKAAKSPAKAKAPKPKAAKPKSGKPKVTKAKKAAPKKK
>sp|P16403|H12_HUMAN Histone H1.2 OS=Homo sapiens OX=9606 GN=H1-2 PE=1 SV=2
MSETAPAAPAAAPPAEKAPVKKKAAKKAGGTPRKASGPPVSELITKAVAASKERSGVSLA
ALKKALAAAGYDVEKNNSRIKLGLKSLVSKGTLVQTKGTGASGSFKLNKKAASGEAKPKV
KKAGGTKPKKPVGAAKKPKKAAGGATPKKSAKKTPKKAKKPAAATVTKKVAKSPKKAKVA
KPKKAAKSAAKAVKPKAAKPKVVKPKKAAPKKK
>sp|P10412|H14_HUMAN Histone H1.4 OS=Homo sapiens OX=9606 GN=H1-4 PE=1 SV=2
MSETAPAAPAAPAPAEKTPVKKKARKSAGAAKRKASGPPVSELITKAVAASKERSGVSLA
ALKKALAAAGYDVEKNNSRIKLGLKSLVSKGTLVQTKGTGASGSFKLNKKAASGEAKPKA
KKAGAAKAKKPAGAAKKPKKATGAATPKKSAKKTPKKAKKPAAAAGAKKAKSPKKAKAAK
PKKAPKSPAKAKAVKPKAAKPKTAKPKAAKPKKAAAKKK
>sp|Q02539|H11_HUMAN Histone H1.1 OS=Homo sapiens OX=9606 GN=H1-1 PE=1 SV=3
MSETVPPAPAASAAPEKPLAGKKAKKPAKAAAASKKKPAGPSVSELIVQAASSSKERGGV
SLAALKKALAAAGYDVEKNNSRIKLGIKSLVSKGTLVQTKGTGASGSFKLNKKASSVETK
PGASKVATKTKATGASKKLKKATGASKKSVKTPKKAKKPAATRKSSKNPKKPKTVKPKKV
AKSPAKAKAVKPKAAKARVTKPKTAKPKKAAPKKK
>sp|P22492|H1T_HUMAN Histone H1t OS=Homo sapiens OX=9606 GN=H1-6 PE=2 SV=4
MSETVPAASASAGVAAMEKLPTKKRGRKPAGLISASRKVPNLSVSKLITEALSVSQERVG
MSLVALKKALAAAGYDVEKNNSRIKLSLKSLVNKGILVQTRGTGASGSFKLSKKVIPKST
RSKAKKSVSAKTKKLVLSRDSKSPKTAKTNKRAKKPRATTPKTVRSGRKAKGAKGKQQQK
SPVKARASKSKLTQHHEVNVRKATSKK
>sp|P62913|RL11_HUMAN 60S ribosomal protein L11 OS=Homo sapiens OX=9606 GN=RPL11 PE=1 SV=2
MAQDQGEKENPMRELRIRKLCLNICVGESGDRLTRAAKVLEQLTGQTPVFSKARYTVRSF
GIRRNEKIAVHCTVRGAKAEEILEKGLKVREYELRKNNFSDTGNFGFGIQEHIDLGIKYD
PSIGIYGLDFYVVLGRPGFSIADKKRRTGCIGAKHRISKEEAMRWFQQKYDGIILPGK
>sp|Q9BYS1|KRA15_HUMAN Keratin-associated protein 1-5 OS=Homo sapiens OX=9606 GN=KRTAP1-5 PE=1 SV=1
MTCCQTSFCGYPSFSISGTCGSSCCQPSCCETSCCQPRSCQTSFCGFPSFSTSGTCSSSC
CQPSCCETSCCQPSCCETSCCQPSCCQISSCGTGCGIGGGISYGQEGSSGAVSTRIRWCR
PDSRVEGTYLPPCCVVSCTPPSCCQLHHAQASCCRPSYCGQSCCRPVCCCEPTC
>sp|P20290|BTF3_HUMAN Transcription factor BTF3 OS=Homo sapiens OX=9606 GN=BTF3 PE=1 SV=1
MRRTGAPAQADSRGRGRARGGCPGGEATLSQPPPRGGTRGQEPQMKETIMNQEKLAKLQA
QVRIGGKGTARRKKKVVHRTATADDKKLQFSLKKLGVNNISGIEEVNMFTNQGTVIHFNN
PKVQASLAANTFTITGHAETKQLTEMLPSILNQLGADSLTSLRRLAEALPKQSVDGKAPL
ATGEDDDDEVPDLVENFDEASKNEAN
>sp|P25788|PSA3_HUMAN Proteasome subunit alpha type-3 OS=Homo sapiens OX=9606 GN=PSMA3 PE=1 SV=2
MSSIGTGYDLSASTFSPDGRVFQVEYAMKAVENSSTAIGIRCKDGVVFGVEKLVLSKLYE
EGSNKRLFNVDRHVGMAVAGLLADARSLADIAREEASNFRSNFGYNIPLKHLADRVAMYV
HAYTLYSAVRPFGCSFMLGSYSVNDGAQLYMIDPSGVSYGYWGCAIGKARQAAKTEIEKL

QMKEMTCRDIVKEVAKIIYIVHDEVKDKAFELELSWVGELTNGRHEIVPKDIREEAEKYA
KESLKEEDESDDDNM
>sp|Q9UQ80|PA2G4_HUMAN Proliferation-associated protein 2G4 OS=Homo sapiens OX=9606 GN=PA2G4 PE=1 SV=3
MSGEDEQQEQTIAEDLVVTKYKMGGDIANRVLRSLVEASSSGVSVLSLCEKGDAMIMEET
GKIFKKEKEMKKGIAFPTSISVNNCVCHFSPLKSDQDYILKEGDLVKIDLGVHVDGFIAN
VAHTFVVDVAQGTQVTGRKADVIKAAHLCAEAALRLVKPGNQNTQVTEAWNKVAHSFNCT
PIEGMLSHQLKQHVIDGEKTIIQNPTDQQKKDHEKAEFEVHEVYAVDVLVSSGEGKAKDA
GQRTTIYKRDPSKQYGLKMKTSRAFFSEVERRFDAMPFTLRAFEDEKKARMGVVECAKHE
LLQPFNVLYEKEGEFVAQFKFTVLLMPNGPMRITSGPFEPDLYKSEMEVQDAELKALLQS
SASRKTQKKKKKKASKTAENATSGETLEENEAGD
>sp|Q14152|EIF3A_HUMAN Eukaryotic translation initiation factor 3 subunit A OS=Homo sapiens OX=9606 GN=EIF3A PE=1 SV=1
MPAYFQRPENALKRANEFLEVGKKQPALDVLYDVMKSKKHRTWQKIHEPIMLKYLELCVD
LRKSHLAKEGLYQYKNICQQVNIKSLEDVVRAYLKMAEEKTEAAKEESQQMVLDIEDLDN
IQTPESVLLSAVSGEDTQDRTDRLLLTPWVKFLWESYRQCLDLLRNNSRVERLYHDIAQQ
AFKFCLQYTRKAEFRKLCDNLRMHLSQIQRHHNQSTAINLNNPESQSMHLETRLVQLDSA
ISMELWQEAFKAVEDIHGLFSLSKKPPKPQLMANYYNKVSTVFWKSGNALFHASTLHRLY
HLSREMRKNLTQDEMQRMSTRVLLATLSIPITPERTDIARLLDMDGIIVEKQRRLATLLG
LQAPPTRIGLINDMVRFNVLQYVVPEVKDLYNWLEVEFNPLKLCERVTKVLNWVREQPEK
EPELQQYVPQLQNNTILRLLQQVSQIYQSIEFSRLTSLVPFVDAFQLERAIVDAARHCDL
QVRIDHTSRTLSFGSDLNYATREDAPIGPHLQSMPSEQIRNQLTAMSSVLAKALEVIKPA
HILQEKEEQHQLAVTAYLKNSRKEHQRILARRQTIEERKERLESLNIQREKEELEQREAE
LQKVRKAEEERLRQEAKEREKERILQEHEQIKKKTVRERLEQIKKTELGAKAFKDIDIED
LEELDPDFIMAKQVEQLEKEKKELQERLKNQEKKIDYFERAKRLEEIPLIKSAYEEQRIK
DMDLWEQQEEERITTMQLEREKALEHKNRMSRMLEDRDLFVMRLKAARQSVYEEKLKQFE
ERLAEERHNRLEERKRQRKEERRITYYREKEEEEQRRAEEQMLKEREEERERAERAKREEE
LREYQERVKKLEEVERKKRQRELEIEERERRREEERRLGDSSLSRKDSRWGDRDSEGTWR
KGPEADSEWRRGPPEKEWRRGEGRDEDRSHRRDEERPRRLGDDEDREPSLRPDDDRVPRR
GMDDDRGPRRGPEEDRFSRRGADDDRPSWRNTDDDRPPRRIADEDRGNWRHADDDRPPRR
GLDEDRGSWRTADEDRGPRRGMDDDRGPRRGGADDERSSWRNADDDRGPRRGLDDDRGPR
RGMDDDRGPRRGMDDDRGPRRGMDDDRGPRRGLDDDRGPWRNADDDRIPRRGAEDDRGPW
RNMDDDRLSRRADDDRFPRRGDDSRPGPWRPLVKPGGWREKEKAREESWGPPRESRPSEE
REWDREKERDRDNQDREENDKDPERERDRERDVDREDRFRRPRDEGGWRRGPAEESSSWR
DSSRRDDRDRDDRRRERDDRRDLRERRDLRDDRDRRGPPLRSEREEVSSWRRADDRKDDR
VEERDPPRRVPPPALSRDRERDRDREREGEKEKASWRAEKDRESLRRTKNETDEDGWTTV
RR
>sp|P51659|DHB4_HUMAN Peroxisomal multifunctional enzyme type 2 OS=Homo sapiens OX=9606 GN=HSD17B4 PE=1 SV=3
MGSPLRFDGRVVLVTGAGAGLGRAYALAFAERGALVVVNDLGGDFKGVGKGSLAADKVVE
EIRRRGGKAVANYDSVEEGEKVVKTALDAFGRIDVVVNNAGILRDRSFARISDEDWDIIH
RVHLRGSFQVTRAAWEHMKKQKYGRIIMTSSASGIYGNFGQANYSAAKLGLLGLANSLAI
EGRKSNIHCNTIAPNAGSRMTQTVMPEDLVEALKPEYVAPLVLWLCHESCEENGGLFEVG
AGWIGKLRWERTLGAIVRQKNHPMTPEAVKANWKKICDFENASKPQSIQESTGSIIEVLS
KIDSEGGVSANHTSRATSTATSGFAGAIGQKLPPFSYAYTELEAIMYALGVGASIKDPKD
LKFIYEGSSDFSCLPTFGVIIGQKSMMGGGLAEIPGLSINFAKVLHGEQYLELYKPLPRA
GKLKCEAVVADVLDKGSGVVIIMDVYSYSEKELICHNQFSLFLVGSGGFGGKRTSDKVKV

AVAIPNRPPDAVLTDTTSLNQAALYRLSGDWNPLHIDPNFASLAGFDKPILHGLCTFGFS
ARRVLQQFADNDVSRFKAIKARFAKPVYPGQTLQTEMWKEGNRIHFQTKVQETGDIVISN
AYVDLAPTSGTSAKTPSEGGKLQSTFVFEEIGRRLKDIGPEVVKKVNAVFEWHITKGGNI
GAKWTIDLKSGSGKVYQGPAKGAADTTIILSDEDFMEVVLGKLDPQKAFFSGRLKARGNI
MLSQKLQMILKDYAKL
>sp|P40429|RL13A_HUMAN 60S ribosomal protein L13a OS=Homo sapiens OX=9606 GN=RPL13A PE=1 SV=2
MAEVQVLVLDGRGHLLGRLAAIVAKQVLLGRKVVVVRCEGINISGNFYRNKLKYLAFLRK
RMNTNPSRGPYHFRAPSRIFWRTVRGMLPHKTKRGQAALDRLKVFDGIPPPYDKKKRMVV
PAALKVVRLKPTRKFAYLGRLAHEVGWKYQAVTATLEEKRKEKAKIHYRKKKQLMRLRKQ
AEKNVEKKIDKYTEVLKTHGLLV
>sp|Q96FQ6|S10AG_HUMAN Protein S100-A16 OS=Homo sapiens OX=9606 GN=S100A16 PE=1 SV=1
MSDCYTELEKAVIVLVENFYKYVSKYSLVKNKISKSSFREMLQKELNHMLSDTGNRKAAD
KLIQNLDANHDGRISFDEYWTLIGGITGPIAKLIHEQEQQSSS
>sp|Q15828|CYTM_HUMAN Cystatin-M OS=Homo sapiens OX=9606 GN=CST6 PE=1 SV=1
MARSNLPLALGLALVAFCLLALPRDARARPQERMVGELRDLSPDDPQVQKAAQAAVASYN
MGSNSIYYFRDTHIIKAQSQLVAGIKYFLTMEMGSTDCRKTRVTGDHVDLTTCPLAAGAQ
QEKLRCDFEVLVVPWQNSSQLLKHNCVQM
>sp|Q13200|PSMD2_HUMAN 26S proteasome non-ATPase regulatory subunit 2 OS=Homo sapiens OX=9606 GN=PSMD2 PE=1 SV=3
MEEGGRDKAPVQPQQSPAAAPGGTDEKPSGKERRDAGDKDKEQELSEEDKQLQDELEMLV
ERLGEKDTSLYRPALEELRRQIRSSTTSMTSVPKPLKFLRPHYGKLKEIYENMAPGENKR
FAADIISVLAMTMSGERECLKYRLVGSQEELASWGHEYVRHLAGEVAKEWQELDDAEKVQ
REPLLTLVKEIVPYNMAHNAEHEACDLLMEIEQVDMLEKDIDENAYAKVCLYLTSCVNYV
PEPENSALLRCALGVFRKFSRFPEALRLALMLNDMELVEDIFTSCKDVVVQKQMAFMLGR
HGVFLELSEDVEEYEDLTEIMSNVQLNSNFLALARELDIMEPKVPDDIYKTHLENNRFGG
SGSQVDSARMNLASSFVNGFVNAAFGQDKLLTDDGNKWLYKNKDHGMLSAAASLGMILLW
DVDGGLTQIDKYLYSSEDYIKSGALLACGIVNSGVRNECDPALALLSDYVLHNSNTMRLG
SIFGLGLAYAGSNREDVLTLLLPVMGDSKSSMEVAGVTALACGMIAVGSCNGDVTSTILQ
TIMEKSETELKDTYARWLPLGLGLNHLGKGEAIEAILAALEVVSEPFRSFANTLVDVCAY
AGSGNVLKVQQLLHICSEHFDSKEKEEDKDKKEKKDKDKKEAPADMGAHQGVAVLGIALI
AMGEEIGAEMALRTFGHLLRYGEPTLRRAVPLALALISVSNPRLNILDTLSKFSHDADPE
VSYNSIFAMGMVGSGTNNARLAAMLRQLAQYHAKDPNNLFMVRLAQGLTHLGKGTLTLCP
YHSDRQLMSQVAVAGLLTVLVSFLDVRNIILGKSHYVLYGLVAAMQPRMLVTFDEELRPL
PVSVRVGQAVDVVGQAGKPKTITGFQTHTTPVLLAHGERAELATEEFLPVTPILEGFVIL
RKNPNYDL
>sp|P48147|PPCE_HUMAN Prolyl endopeptidase OS=Homo sapiens OX=9606 GN=PREP PE=1 SV=2
MLSLQYPDVYRDETAVQDYHGHKICDPYAWLEDPDSEQTKAFVEAQNKITVPFLEQCPIR
GLYKERMTELYDYPKYSCHFKKGKRYFYFYNTGLQNQRVLYVQDSLEGEARVFLDPNILS
DDGTVALRGYAFSEDGEYFAYGLSASGSDWVTIKFMKVDGAKELPDVLERVKFSCMAWTH
DGKGMFYNSYPQQDGKSDGTETSTNLHQKLYYHVLGTDQSEDILCAEFPDEPKWMGGAEL
SDDGRYVLLSIREGCDPVNRLWYCDLQQESSGIAGILKWVKLIDNFEGEYDYVTNEGTVF
TFKTNRQSPNYRVINIDFRDPEESKWKVLVPEHEKDVLEWIACVRSNFLVLCYLHDVKNI
LQLHDLTTGALLKTFPLDVGSIVGYSGQKKDTEIFYQFTSFLSPGIIYHCDLTKEELEPR

VFREVTVKGIDASDYQTVQIFYPSKDGTKIPMFIVHKKGIKLDGSHPAFLYGYGGFNISI
TPNYSVSRLIFVRHMGGILAVANIRGGGEYGETWHKGGILANKQNCFDDFQCAAEYLIKE
GYTSPKRLTINGGSNGGLLVAACANQRPDLFGCVIAQVGVMDMLKFHKYTIGHAWTTDYG
CSDSKQHFEWLVKYSPLHNVKLPEADDIQYPSMLLLTADHDDRVVPLHSLKFIATLQYIV
GRSRKQSNPLLIHVDTKAGHGAGKPTAKVIEEVSDMFAFIARCLNVDWIP
>sp|Q9BYJ1|LOXE3_HUMAN Hydroperoxide isomerase ALOXE3 OS=Homo sapiens OX=9606 GN=ALOXE3 PE=1 SV=1
MAVYRLCVTTGPYLRAGTLDNISVTLVGTCGESPKQRLDRMGRDFAPGSVQKYKVRCTAE
LGELLLLRVHKERYAFFRKDSWYCSRICVTEPDGSVSHFPCYQWIEGYCTVELRPGTART
ICQDSLPLLLDHRTRELRARQECYRWKIYAPGFPCMVDVNSFQEMESDKKFALTKTTTCV
DQGDSSGNRYLPGFPMKIDIPSLMYMEPNVRYSATKTISLLFNAIPASLGMKLRGLLDRK
GSWKKLDDMQNIFWCHKTFTTKYVTEHWCEDHFFGYQYLNGVNPVMLHCISSLPSKLPVT
NDMVAPLLGQDTCLQTELERGNIFLADYWILAEAPTHCLNGRQQYVAAPLCLLWLSPQGA
LVPLAIQLSQTPGPDSPIFLPTDSEWDWLLAKTWVRNSEFLVHENNTHFLCTHLLCEAFA
MATLRQLPLCHPIYKLLLPHTRYTLQVNTIARATLLNPEGLVDQVTSIGRQGLIYLMSTG
LAHFTYTNFCLPDSLRARGVLAIPNYHYRDDGLKIWAAIESFVSEIVGYYYPSDASVQQD
SELQAWTGEIFAQAFLGRESSGFPSRLCTPGEMVKFLTAIIFNCSAQHAAVNSGQHDFGA
WMPNAPSSMRQPPPQTKGTTTLKTYLDTLPEVNISCNNLLLFWLVSQEPKDQRPLGTYPD
EHFTEEAPRRSIAAFQSRLAQISRDIQERNQGLALPYTYLDPPLIENSVSI
>sp|O75083|WDR1_HUMAN WD repeat-containing protein 1 OS=Homo sapiens OX=9606 GN=WDR1 PE=1 SV=4
MPYEIKKVFASLPQVERGVSKIIGGDPKGNNFLYTNGKCVILRNIDNPALADIYTEHAHQ
VVVAKYAPSGFYIASGDVSGKLRIWDTTQKEHLLKYEYQPFAGKIKDIAWTEDSKRIAVV
GEGREKFGAVFLWDSGSSVGEITGHNKVINSVDIKQSRPYRLATGSDDNCAAFFEGPPFK
FKFTIGDHSRFVNCVRFSPDGNRFATASADGQIYIYDGKTGEKVCALGGSKAHDGGIYAI
SWSPDSTHLLSASGDKTSKIWDVSVNSVVSTFPMGSTVLDQQLGCLWQKDHLLSVSLSGY
INYLDRNNPSKPLHVIKGHSKSIQCLTVHKNGGKSYIYSGSHDGHINYWDSETGENDSFA
GKGHTNQVSRMTVDESGQLISCSMDDTVRYTSLMLRDYSGQGVVKLDVQPKCVAVGPGGY
AVVVCIGQIVLLKDQRKCFSIDNPGYEPEVVAVHPGGDTVAIGGVDGNVRLYSILGTTLK
DEGKLLEAKGPVTDVAYSHDGAFLAVCDASKVVTVFSVADGYSENNVFYGHHAKIVCLAW
SPDNEHFASGGMDMMVYVWTLSDPETRVKIQDAHRLHHVSSLAWLDEHTLVTTSHDASVK
EWTITY
>sp|Q15646|OASL_HUMAN 2'-5'-oligoadenylate synthase-like protein OS=Homo sapiens OX=9606 GN=OASL PE=1 SV=2
MALMQELYSTPASRLDSFVAQWLQPHREWKEEVLDAVRTVEEFLRQEHFQGKRGLDQDVR
VLKVVKVGSFGNGTVLRSTREVELVAFLSCFHSFQEAAKHHKDVLRLIWKTMWQSQDLLD
LGLEDLRMEQRVPDALVFTIQTRGTAEPITVTIVPAYRALGPSLPNSQPPPEVYVSLIKA
CGGPGNFCPSFSELQRNFVKHRPTKLKSLLRLVKHWYQQYVKARSPRANLPPLYALELLT
IYAWEMGTEEDENFMLDEGFTTVMDLLLEYEVICIYWTKYYTLHNAIIEDCVRKQLKKER
PIILDPADPTLNVAEGYRWDIVAQRASQCLKQDCCYDNRENPISSWNVKRARDIHLTVEQ
RGYPDFNLIVNPYEPIRKVKEKIRRTRGYSGLQRLSFQVPGSERQLLSSRCSLAKYGIFS
HTHIYLLETIPSEIQVFVKNPDGGSYAYAINPNSFILGLKQQIEDQQGLPKKQQQLEFQG
QVLQDWLGLGIYGIQDSDTLILSKKKGEALFPAS
>sp|P43004|EAA2_HUMAN Excitatory amino acid transporter 2 OS=Homo sapiens OX=9606 GN=SLC1A2 PE=1 SV=2
MASTEGANNMPKQVEVRMHDSHLGSEEPKHRHLGLRLCDKLGKNLLLTLTVFGVILGAVC
GGLLRLASPIHPDVVMLIAFPGDILMRMLKMLILPLIISSLITGLSGLDAKASGRLGTRA

MVYYMSTTIIAAVLGVILVLAIHPGNPKLKKQLGPGKKNDEVSSLDAFLDLIRNLFPENL
VQACFQQIQTVTKKVLVAPPPDEEANATSAVVSLLNETVTEVPEETKMVIKKGLEFKDGM
NVLGLIGFFIAFGIAMGKMGDQAKLMVDFFNILNEIVMKLVIMIMWYSPLGIACLICGKI
IAIKDLEVVARQLGMYMVTVIIGLIIHGGIFLPLIYFVVTRKNPFSFFAGIFQAWITALG
TASSAGTLPVTFRCLEENLGIDKRVTRFVLPVGATINMDGTALYEAVAAIFIAQMNGVVL
DGGQIVTVSLTATLASVGAASIPSAGLVTMLLILTAVGLPTEDISLLVAVDWLLDRMRTS
VNVVGDSFGAGIVYHLSKSELDTIDSQHRVHEDIEMTKTQSIYDDMKNHRESNSNQCVYA
AHNSVIVDECKVTLAANGKSADCSVEEEPWKREK
>sp|Q9NTK5|OLA1_HUMAN Obg-like ATPase 1 OS=Homo sapiens OX=9606 GN=OLA1 PE=1 SV=2
MPPKKGGDGIKPPPIIGRFGTSLKIGIVGLPNVGKSTFFNVLTNSQASAENFPFCTIDPN
ESRVPVPDERFDFLCQYHKPASKIPAFLNVVDIAGLVKGAHNGQGLGNAFLSHISACDGI
FHLTRAFEDDDITHVEGSVDPIRDIEIIHEELQLKDEEMIGPIIDKLEKVAVRGGDKKLK
PEYDIMCKVKSWVIDQKKPVRFYHDWNDKEIEVLNKHLFLTSKPMVYLVNLSEKDYIRKK
NKWLIKIKEWVDKYDPGALVIPFSGALELKLQELSAEERQKYLEANMTQSALPKIIKAGF
AALQLEYFFTAGPDEVRAWTIRKGTKAPQAAGKIHTDFEKGFIMAEVMKYEDFKEEGSEN
AVKAAGKYRQQGRNYIVEDGDIIFFKFNTPQQPKKK
>sp|Q9UKX2|MYH2_HUMAN Myosin-2 OS=Homo sapiens OX=9606 GN=MYH2 PE=1 SV=1
MSSDSELAVFGEAAPFLRKSERERIEAQNRPFDAKTSVFVAEPKESFVKGTIQSREGGKV
TVKTEGGATLTVKDDQVFPMNPPKYDKIEDMAMMTHLHEPAVLYNLKERYAAWMIYTYSG
LFCVTVNPYKWLPVYKPEVVTAYRGKKRQEAPPHIFSISDNAYQFMLTDRENQSILITGE
SGAGKTVNTKRVIQYFATIAVTGEKKKEEITSGKIQGTLEDQIISANPLLEAFGNAKTVR
NDNSSRFGKFIRIHFGTTGKLASADIETYLLEKSRVVFQLKAERSYHIFYQITSNKKPEL
IEMLLITTNPYDYPFVSQGEISVASIDDQEELMATDSAIDILGFTNEEKVSIYKLTGAVM
HYGNLKFKQKQREEQAEPDGTEVADKAAYLQSLNSADLLKALCYPRVKVGNEYVTKGQTV
EQVSNAVGALAKAVYEKMFLWMVARINQQLDTKQPRQYFIGVLDIAGFEIFDFNSLEQLC
INFTNEKLQQFFNHHMFVLEQEEYKKEGIEWTFIDFGMDLAACIELIEKPMGIFSILEEE
CMFPKATDTSFKNKLYDQHLGKSANFQKPKVVKGKAEAHFALIHYAGVVDYNITGWLEKN
KDPLNETVVGLYQKSAMKTLAQLFSGAQTAEGEGAGGGAKKGGKKKGSSFQTVSALFREN
LNKLMTNLRSTHPHFVRCIIPNETKTPGAMEHELVLHQLRCNGVLEGIRICRKGFPSRIL
YADFKQRYKVLNASAIPEGQFIDSKKASEKLLASIDIDHTQYKFGHTKVFFKAGLLGLLE
EMRDDKLAQLITRTQARCRGFLARVEYQRMVERREAIFCIQYNIRSFMNVKHWPWMKLFF
KIKPLLKSAETEKEMATMKEEFQKIKDELAKSEAKRKELEEKMVTLLKEKNDLQLQVQAE
AEGLADAEERCDQLIKTKIQLEAKIKEVTERAEDEEEINAELTAKKRKLEDECSELKKDI
DDLELTLAKVEKEKHATENKVKNLTEEMAGLDETIAKLTKEKKALQEAHQQTLDDLQAEE
DKVNTLTKAKIKLEQQVDDLEGSLEQEKKLRMDLERAKRKLEGDLKLAQESIMDIENEKQ
QLDEKLKKKEFEISNLQSKIEDEQALGIQLQKKIKELQARIEELEEEIEAERASRAKAEK
QRSDLSRELEEISERLEEAGGATSAQIEMNKKREAEFQKMRRDLEEATLQHEATAATLRK
KHADSVAELGEQIDNLQRVKQKLEKEKSEMKMEIDDLASNVETVSKAKGNLEKMCRTLED
QLSELKSKEEEQQRLINDLTAQRGRLQTESGEFSRQLDEKEALVSQLSRGKQAFTQQIEE
LKRQLEEEIKAKNALAHALQSSRHDCDLLREQYEEEQESKAELQRALSKANTEVAQWRTK
YETDAIQRTEELEEAKKKLAQRLQAAEEHVEAVNAKCASLEKTKQRLQNEVEDLMLDVER
TNAACAALDKKQRNFDKILAEWKQKCEETHAELEASQKEARSLGTELFKIKNAYEESLDQ
LETLKRENKNLQQEISDLTEQIAEGGKRIHELEKIKKQVEQEKCELQAALEEAEASLEHE
EGKILRIQLELNQVKSEVDRKIAEKDEEIDQLKRNHIRIVESMQSTLDAEIRSRNDAIRL
KKKMEGDLNEMEIQLNHANRMAAEALRNYRNTQGILKDTQIHLDDALRSQEDLKEQLAMV

ERRANLLQAEIEELRATLEQTERSRKIAEQELLDASERVQLLHTQNTSLINTKKKLETDI
SQMQGEMEDILQEARNAEEKAKKAITDAAMMAEELKKEQDTSAHLERMKKNMEQTVKDLQ
LRLDEAEQLALKGGKKQIQKLEARVRELEGEVESEQKRNAEAVKGLRKHERRVKELTYQT
EEDRKNILRLQDLVDKLQAKVKSYKRQAEEAEEQSNTNLAKFRKLQHELEEAEERADIAE
SQVNKLRVKSREVHTKVISEE
>sp|P12882|MYH1_HUMAN Myosin-1 OS=Homo sapiens OX=9606 GN=MYH1 PE=1 SV=3
MSSDSEMAIFGEAAPFLRKSERERIEAQNKPFDAKTSVFVVDPKESFVKATVQSREGGKV
TAKTEAGATVTVKDDQVFPMNPPKYDKIEDMAMMTHLHEPAVLYNLKERYAAWMIYTYSG
LFCVTVNPYKWLPVYNAEVVTAYRGKKRQEAPPHIFSISDNAYQFMLTDRENQSILITGE
SGAGKTVNTKRVIQYFATIAVTGEKKKEEVTSGKMQGTLEDQIISANPLLEAFGNAKTVR
NDNSSRFGKFIRIHFGTTGKLASADIETYLLEKSRVTFQLKAERSYHIFYQIMSNKKPDL
IEMLLITTNPYDYAFVSQGEITVPSIDDQEELMATDSAIEILGFTSDERVSIYKLTGAVM
HYGNMKFKQKQREEQAEPDGTEVADKAAYLQNLNSADLLKALCYPRVKVGNEYVTKGQTV
QQVYNAVGALAKAVYDKMFLWMVTRINQQLDTKQPRQYFIGVLDIAGFEIFDFNSLEQLC
INFTNEKLQQFFNHHMFVLEQEEYKKEGIEWTFIDFGMDLAACIELIEKPMGIFSILEEE
CMFPKATDTSFKNKLYEQHLGKSNNFQKPKPAKGKPEAHFSLIHYAGTVDYNIAGWLDKN
KDPLNETVVGLYQKSAMKTLALLFVGATGAEAEAGGGKKGGKKKGSSFQTVSALFRENLN
KLMTNLRSTHPHFVRCIIPNETKTPGAMEHELVLHQLRCNGVLEGIRICRKGFPSRILYA
DFKQRYKVLNASAIPEGQFIDSKKASEKLLGSIDIDHTQYKFGHTKVFFKAGLLGLLEEM
RDEKLAQLITRTQAMCRGFLARVEYQKMVERRESIFCIQYNVRAFMNVKHWPWMKLYFKI
KPLLKSAETEKEMANMKEEFEKTKEELAKTEAKRKELEEKMVTLMQEKNDLQLQVQAEAD
SLADAEERCDQLIKTKIQLEAKIKEVTERAEDEEEINAELTAKKRKLEDECSELKKDIDD
LELTLAKVEKEKHATENKVKNLTEEMAGLDETIAKLTKEKKALQEAHQQTLDDLQAEEDK
VNTLTKAKIKLEQQVDDLEGSLEQEKKIRMDLERAKRKLEGDLKLAQESTMDIENDKQQL
DEKLKKKEFEMSGLQSKIEDEQALGMQLQKKIKELQARIEELEEEIEAERASRAKAEKQR
SDLSRELEEISERLEEAGGATSAQIEMNKKREAEFQKMRRDLEEATLQHEATAATLRKKH
ADSVAELGEQIDNLQRVKQKLEKEKSEMKMEIDDLASNMETVSKAKGNLEKMCRALEDQL
SEIKTKEEEQQRLINDLTAQRARLQTESGEYSRQLDEKDTLVSQLSRGKQAFTQQIEELK
RQLEEEIKAKSALAHALQSSRHDCDLLREQYEEEQEAKAELQRAMSKANSEVAQWRTKYE
TDAIQRTEELEEAKKKLAQRLQDAEEHVEAVNAKCASLEKTKQRLQNEVEDLMIDVERTN
AACAALDKKQRNFDKILAEWKQKCEETHAELEASQKESRSLSTELFKIKNAYEESLDQLE
TLKRENKNLQQEISDLTEQIAEGGKRIHELEKIKKQVEQEKSELQAALEEAEASLEHEEG
KILRIQLELNQVKSEVDRKIAEKDEEIDQMKRNHIRIVESMQSTLDAEIRSRNDAIRLKK
KMEGDLNEMEIQLNHANRMAAEALRNYRNTQAILKDTQLHLDDALRSQEDLKEQLAMVER
RANLLQAEIEELRATLEQTERSRKIAEQELLDASERVQLLHTQNTSLINTKKKLETDISQ
IQGEMEDIIQEARNAEEKAKKAITDAAMMAEELKKEQDTSAHLERMKKNLEQTVKDLQHR
LDEAEQLALKGGKKQIQKLEARVRELEGEVESEQKRNVEAVKGLRKHERKVKELTYQTEE
DRKNILRLQDLVDKLQAKVKSYKRQAEEAEEQSNVNLSKFRRIQHELEEAEERADIAESQ
VNKLRVKSREVHTKIISEE
>sp|P48163|MAOX_HUMAN NADP-dependent malic enzyme OS=Homo sapiens OX=9606 GN=ME1 PE=1 SV=1
MEPEAPRRRHTHQRGYLLTRNPHLNKDLAFTLEERQQLNIHGLLPPSFNSQEIQVLRVVK
NFEHLNSDFDRYLLLMDLQDRNEKLFYRVLTSDIEKFMPIVYTPTVGLACQQYSLVFRKP
RGLFITIHDRGHIASVLNAWPEDVIKAIVVTDGERILGLGDLGCNGMGIPVGKLALYTAC
GGMNPQECLPVILDVGTENEELLKDPLYIGLRQRRVRGSEYDDFLDEFMEAVSSKYGMNC
LIQFEDFANVNAFRLLNKYRNQYCTFNDDIQGTASVAVAGLLAALRITKNKLSDQTILFQ

GAGEAALGIAHLIVMALEKEGLPKEKAIKKIWLVDSKGLIVKGRASLTQEKEKFAHEHEE
MKNLEAIVQEIKPTALIGVAAIGGAFSEQILKDMAAFNERPIIFALSNPTSKAECSAEQC
YKITKGRAIFASGSPFDPVTLPNGQTLYPGQGNNSYVFPGVALGVVACGLRQITDNIFLT
TAEVIAQQVSDKHLEEGRLYPPLNTIRDVSLKIAEKIVKDAYQEKTATVYPEPQNKEAFV
RSQMYSTDYDQILPDCYSWPEEVQKIQTKVDQ
>sp|P50993|AT1A2_HUMAN Sodium/potassium-transporting ATPase subunit alpha-2 OS=Homo sapiens OX=9606 GN=ATP1A2 PE=1 SV=1
MGRGAGREYSPAATTAENGGGKKKQKEKELDELKKEVAMDDHKLSLDELGRKYQVDLSKG
LTNQRAQDVLARDGPNALTPPPTTPEWVKFCRQLFGGFSILLWIGAILCFLAYGIQAAME
DEPSNDNLYLGVVLAAVVIVTGCFSYYQEAKSSKIMDSFKNMVPQQALVIREGEKMQINA
EEVVVGDLVEVKGGDRVPADLRIISSHGCKVDNSSLTGESEPQTRSPEFTHENPLETRNI
CFFSTNCVEGTARGIVIATGDRTVMGRIATLASGLEVGRTPIAMEIEHFIQLITGVAVFL
GVSFFVLSLILGYSWLEAVIFLIGIIVANVPEGLLATVTVCLTLTAKRMARKNCLVKNLE
AVETLGSTSTICSDKTGTLTQNRMTVAHMWFDNQIHEADTTEDQSGATFDKRSPTWTALS
RIAGLCNRAVFKAGQENISVSKRDTAGDASESALLKCIELSCGSVRKMRDRNPKVAEIPF
NSTNKYQLSIHEREDSPQSHVLVMKGAPERILDRCSTILVQGKEIPLDKEMQDAFQNAYM
ELGGLGERVLGFCQLNLPSGKFPRGFKFDTDELNFPTEKLCFVGLMSMIDPPRAAVPDAV
GKCRSAGIKVIMVTGDHPITAKAIAKGVGIISEGNETVEDIAARLNIPMSQVNPREAKAC
VVHGSDLKDMTSEQLDEILKNHTEIVFARTSPQQKLIIVEGCQRQGAIVAVTGDGVNDSP
ALKKADIGIAMGISGSDVSKQAADMILLDDNFASIVTGVEEGRLIFDNLKKSIAYTLTSN
IPEITPFLLFIIANIPLPLGTVTILCIDLGTDMVPAISLAYEAAESDIMKRQPRNSQTDK
LVNERLISMAYGQIGMIQALGGFFTYFVILAENGFLPSRLLGIRLDWDDRTMNDLEDSYG
QEWTYEQRKVVEFTCHTAFFASIVVVQWADLIICKTRRNSVFQQGMKNKILIFGLLEETA
LAAFLSYCPGMGVALRMYPLKVTWWFCAFPYSLLIFIYDEVRKLILRRYPGGWVEKETYY
>sp|P62280|RS11_HUMAN 40S ribosomal protein S11 OS=Homo sapiens OX=9606 GN=RPS11 PE=1 SV=3
MADIQTERAYQKQPTIFQNKKRVLLGETGKEKLPRYYKNIGLGFKTPKEAIEGTYIDKKC
PFTGNVSIRGRILSGVVTKMKMQRTIVIRRDYLHYIRKYNRFEKRHKNMSVHLSPCFRDV
QIGDIVTVGECRPLSKTVRFNVLKVTKAAGTKKQFQKF
>sp|Q9BYQ8|KRA49_HUMAN Keratin-associated protein 4-9 OS=Homo sapiens OX=9606 GN=KRTAP4-9 PE=2 SV=2
MVSSCCGSVCSDQGCGQDLCQETCCRPSCCETTCCRTTCCRPSCCVSSCCRPQCCQSVCC
QPTCSRPSCCQTTCCRTTCYRPSCCVSSCCRPQCCQPACCQPTCCRPSCCETTCCHPRCC
ISSCCRPSCCVSSCCKPQCCQSVCCQPNCCRPSCSISSCCRPSCCESSCCRPCCCVRPVC
GRVSCHTTCYRPTCVISSCPRPLCCASSCC
>sp|P02787|TRFE_HUMAN Serotransferrin OS=Homo sapiens OX=9606 GN=TF PE=1 SV=3
MRLAVGALLVCAVLGLCLAVPDKTVRWCAVSEHEATKCQSFRDHMKSVIPSDGPSVACVK
KASYLDCIRAIAANEADAVTLDAGLVYDAYLAPNNLKPVVAEFYGSKEDPQTFYYAVAVV
KKDSGFQMNQLRGKKSCHTGLGRSAGWNIPIGLLYCDLPEPRKPLEKAVANFFSGSCAPC
ADGTDFPQLCQLCPGCGCSTLNQYFGYSGAFKCLKDGAGDVAFVKHSTIFENLANKADRD
QYELLCLDNTRKPVDEYKDCHLAQVPSHTVVARSMGGKEDLIWELLNQAQEHFGKDKSKE
FQLFSSPHGKDLLFKDSAHGFLKVPPRMDAKMYLGYEYVTAIRNLREGTCPEAPTDECKP
VKWCALSHHERLKCDEWSVNSVGKIECVSAETTEDCIAKIMNGEADAMSLDGGFVYIAGK
CGLVPVLAENYNKSDNCEDTPEAGYFAIAVVKKSASDLTWDNLKGKKSCHTAVGRTAGWN
IPMGLLYNKINHCRFDEFFSEGCAPGSKKDSSLCKLCMGSGLNLCEPNNKEGYYGYTGAF
RCLVEKGDVAFVKHQTVPQNTGGKNPDPWAKNLNEKDYELLCLDGTRKPVEEYANCHLAR

APNHAVVTRKDKEACVHKILRQQQHLFGSNVTDCSGNFCLFRSETKDLLFRDDTVCLAKL
HDRNTYEKYLGEEYVKAVGNLRKCSTSSLLEACTFRRP
>sp|O15145|ARPC3_HUMAN Actin-related protein 2/3 complex subunit 3 OS=Homo sapiens OX=9606 GN=ARPC3 PE=1 SV=3
MPAYHSSLMDPDTKLIGNMALLPIRSQFKGPAPRETKDTDIVDEAIYYFKANVFFKNYEI
KNEADRTLIYITLYISECLKKLQKCNSKSQGEKEMYTLGITNFPIPGEPGFPLNAIYAKP
ANKQEDEVMRAYLQQLRQETGLRLCEKVFDPQNDKPSKWWTCFVKRQFMNKSLSGPGQ
>sp|O95197|RTN3_HUMAN Reticulon-3 OS=Homo sapiens OX=9606 GN=RTN3 PE=1 SV=2
MAEPSAATQSHSISSSSFGAEPSAPGGGGSPGACPALGTKSCSSSCADSFVSSSSSQPVS
LFSTSQEGLSSLCSDEPSSEIMTSSFLSSSEIHNTGLTILHGEKSHVLGSQPILAKEGKD
HLDLLDMKKMEKPQGTSNNVSDSSVSLAAGVHCDRPSIPASFPEHPAFLSKKIGQVEEQI
DKETKNPNGVSSREAKTALDADDRFTLLTAQKPPTEYSKVEGIYTYSLSPSKVSGDDVIE
KDSPESPFEVIIDKAAFDKEFKDSYKESTDDFGSWSVHTDKESSEDISETNDKLFPLRNK
EAGRYPMSALLSRQFSHTNAALEEVSRCVNDMHNFTNEILTWDLVPQVKQQTDKSSDCIT
KTTGLDMSEYNSEIPVVNLKTSTHQKTPVCSIDGSTPITKSTGDWAEASLQQENAITGKP
VPDSLNSTKEFSIKGVQGNMQKQDDTLAELPGSPPEKCDSLGSGVATVKVVLPDDHLKDE
MDWQSSALGEITEADSSGESDDTVIEDITADTSFENNKIQAEKPVSIPSAVVKTGEREIK
EIPSCEREEKTSKNFEELVSDSELHQDQPDILGRSPASEAACSKVPDTNVSLEDVSEVAP
EKPITTENPKLPSTVSPNVFNETEFSLNVTTSAYLESLHGKNVKHIDDSSPEDLIAAFTE
TRDKGIVDSERNAFKAISEKMTDFKTTPPVEVLHENESGGSEIKDIGSKYSEQSKETNGS
EPLGVFPTQGTPVASLDLEQEQLTIKALKELGERQVEKSTSAQRDAELPSEEVLKQTFTF
APESWPQRSYDILERNVKNGSDLGISQKPITIRETTRVDAVSSLSKTELVKKHVLARLLT
DFSVHDLIFWRDVKKTGFVFGTTLIMLLSLAAFSVISVVSYLILALLSVTISFRIYKSVI
QAVQKSEEGHPFKAYLDVDITLSSEAFHNYMNAAMVHINRALKLIIRLFLVEDLVDSLKL
AVFMWLMTYVGAVFNGITLLILAELLIFSVPIVYEKYKTQIDHYVGIARDQTKSIVEKIQ
AKLPGIAKKKAE
>sp|P46783|RS10_HUMAN 40S ribosomal protein S10 OS=Homo sapiens OX=9606 GN=RPS10 PE=1 SV=1
MLMPKKNRIAIYELLFKEGVMVAKKDVHMPKHPELADKNVPNLHVMKAMQSLKSRGYVKE
QFAWRHFYWYLTNEGIQYLRDYLHLPPEIVPATLRRSRPETGRPRPKGLEGERPARLTRG
EADRDTYRRSAVPPGADKKAEAGAGSATEFQFRGGFGRGRGQPPQ
>sp|Q9UHG3|PCYOX_HUMAN Prenylcysteine oxidase 1 OS=Homo sapiens OX=9606 GN=PCYOX1 PE=1 SV=3
MGRVVAELVSSLLGLWLLLCSCGCPEGAELRAPPDKIAIIGAGIGGTSAAYYLRQKFGKD
VKIDLFEREEVGGRLATMMVQGQEYEAGGSVIHPLNLHMKRFVKDLGLSAVQASGGLLGI
YNGETLVFEESNWFIINVIKLVWRYGFQSLRMHMWVEDVLDKFMRIYRYQSHDYAFSSVE
KLLHALGGDDFLGMLNRTLLETLQKAGFSEKFLNEMIAPVMRVNYGQSTDINAFVGAVSL
SCSDSGLWAVEGGNKLVCSGLLQASKSNLISGSVMYIEEKTKTKYTGNPTKMYEVVYQIG
TETRSDFYDIVLVATPLNRKMSNITFLNFDPPIEEFHQYYQHIVTTLVKGELNTSIFSSR
PIDKFGLNTVLTTDNSDLFINSIGIVPSVREKEDPEPSTDGTYVWKIFSQETLTKAQILK
LFLSYDYAVKKPWLAYPHYKPPEKCPSIILHDRLYYLNGIECAASAMEMSAIAAHNAALL
AYHRWNGHTDMIDQDGLYEKLKTEL
>sp|P63220|RS21_HUMAN 40S ribosomal protein S21 OS=Homo sapiens OX=9606 GN=RPS21 PE=1 SV=1
MQNDAGEFVDLYVPRKCSASNRIIGAKDHASIQMNVAEVDKVTGRFNGQFKTYAICGAIR
RMGESDDSILRLAKADGIVSKNF

>sp|P35268|RL22_HUMAN 60S ribosomal protein L22 OS=Homo sapiens OX=9606 GN=RPL22 PE=1 SV=2
MAPVKKLVVKGGKKKKQVLKFTLDCTHPVEDGIMDAANFEQFLQERIKVNGKAGNLGGGV
VTIERSKSKITVTSEVPFSKRYLKYLTKKYLKKNNLRDWLRVVANSKESYELRYFQINQD
EEEEEDED
>sp|P43307|SSRA_HUMAN Translocon-associated protein subunit alpha OS=Homo sapiens OX=9606 GN=SSR1 PE=1 SV=3
MRLLPRLLLLLLLVFPATVLFRGGPRGLLAVAQDLTEDEETVEDSIIEDEDDEAEVEEDE
PTDLVEDKEEEDVSGEPEASPSADTTILFVKGEDFPANNIVKFLVGFTNKGTEDFIVESL
DASFRYPQDYQFYIQNFTALPLNTVVPPQRQATFEYSFIPAEPMGGRPFGLVINLNYKDL
NGNVFQDAVFNQTVTVIEREDGLDGETIFMYMFLAGLGLLVIVGLHQLLESRKRKRPIQK
VEMGTSSQNDVDMSWIPQETLNQINKASPRRLPRKRAQKRSVGSDE
>sp|P01036|CYTS_HUMAN Cystatin-S OS=Homo sapiens OX=9606 GN=CST4 PE=1 SV=3
MARPLCTLLLLMATLAGALASSSKEENRIIPGGIYDADLNDEWVQRALHFAISEYNKATE
DEYYRRPLQVLRAREQTFGGVNYFFDVEVGRTICTKSQPNLDTCAFHEQPELQKKQLCSF
EIYEVPWEDRMSLVNSRCQEA
>sp|P50991|TCPD_HUMAN T-complex protein 1 subunit delta OS=Homo sapiens OX=9606 GN=CCT4 PE=1 SV=4
MPENVAPRSGATAGAAGGRGKGAYQDRDKPAQIRFSNISAAKAVADAIRTSLGPKGMDKM
IQDGKGDVTITNDGATILKQMQVLHPAARMLVELSKAQDIEAGDGTTSVVIIAGSLLDSC
TKLLQKGIHPTIISESFQKALEKGIEILTDMSRPVELSDRETLLNSATTSLNSKVVSQYS
SLLSPMSVNAVMKVIDPATATSVDLRDIKIVKKLGGTIDDCELVEGLVLTQKVSNSGITR
VEKAKIGLIQFCLSAPKTDMDNQIVVSDYAQMDRVLREERAYILNLVKQIKKTGCNVLLI
QKSILRDALSDLALHFLNKMKIMVIKDIEREDIEFICKTIGTKPVAHIDQFTADMLGSAE
LAEEVNLNGSGKLLKITGCASPGKTVTIVVRGSNKLVIEEAERSIHDALCVIRCLVKKRA
LIAGGGAPEIELALRLTEYSRTLSGMESYCVRAFADAMEVIPSTLAENAGLNPISTVTEL
RNRHAQGEKTAGINVRKGGISNILEELVVQPLLVSVSALTLATETVRSILKIDDVVNTR
>sp|P59666|DEF3_HUMAN Neutrophil defensin 3 OS=Homo sapiens OX=9606 GN=DEFA3 PE=1 SV=1
MRTLAILAAILLVALQAQAEPLQARADEVAAAPEQIAADIPEVVVSLAWDESLAPKHPGS
RKNMDCYCRIPACIAGERRYGTCIYQGRLWAFCC
>sp|P59665|DEF1_HUMAN Neutrophil defensin 1 OS=Homo sapiens OX=9606 GN=DEFA1B PE=1 SV=1
MRTLAILAAILLVALQAQAEPLQARADEVAAAPEQIAADIPEVVVSLAWDESLAPKHPGS
RKNMACYCRIPACIAGERRYGTCIYQGRLWAFCC
>sp|P35998|PRS7_HUMAN 26S proteasome regulatory subunit 7 OS=Homo sapiens OX=9606 GN=PSMC2 PE=1 SV=3
MPDYLGADQRKTKEDEKDDKPIRALDEGDIALLKTYGQSTYSRQIKQVEDDIQQLLKKIN
ELTGIKESDTGLAPPALWDLAADKQTLQSEQPLQVARCTKIINADSEDPKYIINVKQFAK
FVVDLSDQVAPTDIEEGMRVGVDRNKYQIHIPLPPKIDPTVTMMQVEEKPDVTYSDVGGC
KEQIEKLREVVETPLLHPERFVNLGIEPPKGVLLFGPPGTGKTLCARAVANRTDACFIRV
IGSELVQKYVGEGARMVRELFEMARTKKACLIFFDEIDAIGGARFDDGAGGDNEVQRTML
ELINQLDGFDPRGNIKVLMATNRPDTLDPALMRPGRLDRKIEFSLPDLEGRTHIFKIHAR
SMSVERDIRFELLARLCPNSTGAEIRSVCTEAGMFAIRARRKIATEKDFLEAVNKVIKSY
AKFSATPRYMTYN

>sp|P47756|CAPZB_HUMAN F-actin-capping protein subunit beta OS=Homo sapiens OX=9606 GN=CAPZB PE=1 SV=4
MSDQQLDCALDLMRRLPPQQIEKNLSDLIDLVPSLCEDLLSSVDQPLKIARDKVVGKDYL
LCDYNRDGDSYRSPWSNKYDPPLEDGAMPSARLRKLEVEANNAFDQYRDLYFEGGVSSVY
LWDLDHGFAGVILIKKAGDGSKKIKGCWDSIHVVEVQEKSSGRTAHYKLTSTVMLWLQTN
KSGSGTMNLGGSLTRQMEKDETVSDCSPHIANIGRLVEDMENKIRSTLNEIYFGKTKDIV
NGLRSIDAIPDNQKFKQLQRELSQVLTQRQIYIQPDN
>sp|P15374|UCHL3_HUMAN Ubiquitin carboxyl-terminal hydrolase isozyme L3 OS=Homo sapiens OX=9606 GN=UCHL3 PE=1 SV=1
MEGQRWLPLEANPEVTNQFLKQLGLHPNWQFVDVYGMDPELLSMVPRPVCAVLLLFPITE
KYEVFRTEEEEKIKSQGQDVTSSVYFMKQTISNACGTIGLIHAIANNKDKMHFESGSTLK
KFLEESVSMSPEERARYLENYDAIRVTHETSAHEGQTEAPSIDEKVDLHFIALVHVDGHL
YELDGRKPFPINHGETSDETLLEDAIEVCKKFMERDPDELRFNAIALSAA
>sp|Q9H2S5|RNF39_HUMAN RING finger protein 39 OS=Homo sapiens OX=9606 GN=RNF39 PE=1 SV=2
MWWRDLTRLRLWLKREAIPGEGRKAAKVNAGVGEKGIYTASSRGGPPSARSKAVTVVAEG
AASRSWLSMDAPELGPGLVERLEQLATCPLCGGSFEDPVLLACEHSFCRACLARRWGTPP
ATGTEASPTACPCCGLPCPRRSLRSNVRLAVEVRISRELREKLAEPGARAGRRRGGRIPT
MGCLDLPGEDMRKTWRRFEVPTSKSSNSEDDLPEDYPVVKKMLHRLTADLTLDPGTAHRR
LLISADRRSVQLAPPGTPAPPDGPKRFDQLPAVLGAQGFGAGRHCWEVETADAASCRDSS
GEDADDEESHYAVGAAGESVQRKGCVRLCPAGAVWAVEGRGGRLWALTAPEPTLLGGVEP
PPRRIRVDLDWERGRVAFYDGRSLDLLYAFQAPGPLGERIFPLFCTCDPRAPLRIVPAES
>sp|Q6P4A8|PLBL1_HUMAN Phospholipase B-like 1 OS=Homo sapiens OX=9606 GN=PLBD1 PE=1 SV=2
MTRGGPGGRPGLPQPPPLLLLLLLLPLLLVTAEPPKPAGVYYATAYWMPAEKTVQVKNVM
DKNGDAYGFYNNSVKTTGWGILEIRAGYGSQTLSNEIIMFVAGFLEGYLTAPHMNDHYTN
LYPQLITKPSIMDKVQDFMEKQDKWTRKNIKEYKTDSFWRHTGYVMAQIDGLYVGAKKRA
ILEGTKPMTLFQIQFLNSVGDLLDLIPSLSPTKNGSLKVFKRWDMGHCSALIKVLPGFEN
ILFAHSSWYTYAAMLRIYKHWDFNVIDKDTSSSRLSFSSYPGFLESLDDFYILSSGLILL
QTTNSVFNKTLLKQVIPETLLSWQRVRVANMMADSGKRWADIFSKYNSGTYNNQYMVLDL
KKVKLNHSLDKGTLYIVEQIPTYVEYSEQTDVLRKGYWPSYNVPFHEKIYNWSGYPLLVQ
KLGLDYSYDLAPRAKIFRRDQGKVTDTASMKYIMRYNNYKKDPYSRGDPCNTICCREDLN
SPNPSPGGCYDTKVADIYLASQYTSYAISGPTVQGGLPVFRWDRFNKTLHQGMPEVYNFD
FITMKPILKLDIK
>sp|Q9ULV4|COR1C_HUMAN Coronin-1C OS=Homo sapiens OX=9606 GN=CORO1C PE=1 SV=1
MRRVVRQSKFRHVFGQAVKNDQCYDDIRVSRVTWDSSFCAVNPRFVAIIIEASGGGAFLV
LPLHKTGRIDKSYPTVCGHTGPVLDIDWCPHNDQVIASGSEDCTVMVWQIPENGLTLSLT
EPVVILEGHSKRVGIVAWHPTARNVLLSAGCDNAIIIWNVGTGEALINLDDMHSDMIYNV
SWNRNGSLICTASKDKKVRVIDPRKQEIVAEKEKAHEGARPMRAIFLADGNVFTTGFSRM
SERQLALWNPKNMQEPIALHEMDTSNGVLLPFYDPDTSIIYLCGKGDSSIRYFEITDESP
YVHYLNTFSSKEPQRGMGYMPKRGLDVNKCEIARFFKLHERKCEPIIMTVPRKSDLFQDD
LYPDTAGPEAALEAEEWFEGKNADPILISLKHGYIPGKNRDLKVVKKNILDSKPTANKKC
DLISIPKKTTDTASVQNEAKLDEILKEIKSIKDTICNQDERISKLEQQMAKIAA
>sp|P61020|RAB5B_HUMAN Ras-related protein Rab-5B OS=Homo sapiens OX=9606 GN=RAB5B PE=1 SV=1
MTSRSTARPNGQPQASKICQFKLVLLGESAVGKSSLVLRFVKGQFHEYQESTIGAAFLTQ

SVCLDDTTVKFEIWDTAGQERYHSLAPMYYRGAQAAIVVYDITNQETFARAKTWVKELQR
QASPSIVIALAGNKADLANKRMVEYEEAQAYADDNSLLFMETSAKTAMNVNDLFLAIAKK
LPKSEPQNLGGAAGRSRGVDLHEQSQQNKSQCCSN
>sp|P62857|RS28_HUMAN 40S ribosomal protein S28 OS=Homo sapiens OX=9606 GN=RPS28 PE=1 SV=1
MDTSRVQPIKLARVTKVLGRTGSQGQCTQVRVEFMDDTSRSIIRNVKGPVREGDVLTLLE
SEREARRLR
>sp|O43548|TGM5_HUMAN Protein-glutamine gamma-glutamyltransferase 5 OS=Homo sapiens OX=9606 GN=TGM5 PE=1 SV=4
MAQGLEVALTDLQSSRNNVRHHTEEITVDHLLVRRGQAFNLTLYFRNRSFQPGLDNIIFV
VETGPLPDLALGTRAVFSLARHHSPSPWIAWLETNGATSTEVSLCAPPTAAVGRYLLKIH
IDSFQGSVTAYQLGEFILLFNPWCPEDAVYLDSEPQRQEYVMNDYGFIYQGSKNWIRPCP
WNYGQFEDKIIDICLKLLDKSLHFQTDPATDCALRGSPVYVSRVVCAMINSNDDNGVLNG
NWSENYTDGANPAEWTGSVAILKQWNATGCQPVRYGQCWVFAAVMCTVMRCLGIPTRVIT
NFDSGHDTDGNLIIDEYYDNTGRILGNKKKDTIWNFHVWNECWMARKDLPPAYGGWQVLD
ATPQEMSNGVYCCGPASVRAIKEGEVDLNYDTPFVFSMVNADCMSWLVQGGKEQKLHQDT
SSVGNFISTKSIQSDERDDITENYKYEEGSLQERQVFLKALQKLKARSFHGSQRGAELQP
SRPTSLSQDSPRSLHTPSLRPSDVVQVSLKFKLLDPPNMGQDICFVLLALNMSSQFKDLK
VNLSAQSLLHDGSPLSPFWQDTAFITLSPKEAKTYPCKISYSQYSQYLSTDKLIRISALG
EEKSSPEKILVNKIITLSYPSITINVLGAAVVNQPLSIQVIFSNPLSEQVEDCVLTVEGS
GLFKKQQKVFLGVLKPQHQASIILETVPFKSGQRQIQANMRSNKFKDIKGYRNVYVDFAL
>sp|P0C0L5|CO4B_HUMAN Complement C4-B OS=Homo sapiens OX=9606 GN=C4B_2 PE=1 SV=2
MRLLWGLIWASSFFTLSLQKPRLLLFSPSVVHLGVPLSVGVQLQDVPRGQVVKGSVFLRN
PSRNNVPCSPKVDFTLSSERDFALLSLQVPLKDAKSCGLHQLLRGPEVQLVAHSPWLKDS
LSRTTNIQGINLLFSSRRGHLFLQTDQPIYNPGQRVRYRVFALDQKMRPSTDTITVMVEN
SHGLRVRKKEVYMPSSIFQDDFVIPDISEPGTWKISARFSDGLESNSSTQFEVKKYVLPN
FEVKITPGKPYILTVPGHLDEMQLDIQARYIYGKPVQGVAYVRFGLLDEDGKKTFFRGLE
SQTKLVNGQSHISLSKAEFQDALEKLNMGITDLQGLRLYVAAAIIESPGGEMEEAELTSW
YFVSSPFSLDLSKTKRHLVPGAPFLLQALVREMSGSPASGIPVKVSATVSSPGSVPEVQD
IQQNTDGSGQVSIPIIIPQTISELQLSVSAGSPHPAIARLTVAAPPSGGPGFLSIERPDS
RPPRVGDTLNLNLRAVGSGATFSHYYYMILSRGQIVFMNREPKRTLTSVSVFVDHHLAPS
FYFVAFYYHGDHPVANSLRVDVQAGACEGKLELSVDGAKQYRNGESVKLHLETDSLALVA
LGALDTALYAAGSKSHKPLNMGKVFEAMNSYDLGCGPGGGDSALQVFQAAGLAFSDGDQW
TLSRKRLSCPKEKTTRKKRNVNFQKAINEKLGQYASPTAKRCCQDGVTRLPMMRSCEQRA
ARVQQPDCREPFLSCCQFAESLRKKSRDKGQAGLQRALEILQEEDLIDEDDIPVRSFFPE
NWLWRVETVDRFQILTLWLPDSLTTWEIHGLSLSKTKGLCVATPVQLRVFREFHLHLRLP
MSVRRFEQLELRPVLYNYLDKNLTVSVHVSPVEGLCLAGGGGLAQQVLVPAGSARPVAFS
VVPTAATAVSLKVVARGSFEFPVGDAVSKVLQIEKEGAIHREELVYELNPLDHRGRTLEI
PGNSDPNMIPDGDFNSYVRVTASDPLDTLGSEGALSPGGVASLLRLPRGCGEQTMIYLAP
TLAASRYLDKTEQWSTLPPETKDHAVDLIQKGYMRIQQFRKADGSYAAWLSRGSSTWLTA
FVLKVLSLAQEQVGGSPEKLQETSNWLLSQQQADGSFQDLSPVIHRSMQGGLVGNDETVA
LTAFVTIALHHGLAVFQDEGAEPLKQRVEASISKASSFLGEKASAGLLGAHAAAITAYAL
TLTKAPADLRGVAHNNLMAMAQETGDNLYWGSVTGSQSNAVSPTPAPRNPSDPMPQAPAL
WIETTAYALLHLLLHEGKAEMADQAAAWLTRQGSFQGGFRSTQDTVIALDALSAYWIASH
TTEERGLNVTLSSTGRNGFKSHALQLNNRQIRGLEEELQFSLGSKINVKVGGNSKGTLKV
LRTYNVLDMKNTTCQDLQIEVTVKGHVEYTMEANEDYEDYEYDELPAKDDPDAPLQPVTP

LQLFEGRRNRRRREAPKVVEEQESRVHYTVCIWRNGKVGLSGMAIADVTLLSGFHALRAD
LEKLTSLSDRYVSHFETEGPHVLLYFDSVPTSRECVGFEAVQEVPVGLVQPASATLYDYY
NPERRCSVFYGAPSKSRLLATLCSAEVCQCAEGKCPRQRRALERGLQDEDGYRMKFACYY
PRVEYGFQVKVLREDSRAAFRLFETKITQVLHFTKDVKAAANQMRNFLVRASCRLRLEPG
KEYLIMGLDGATYDLEGHPQYLLDSNSWIEEMPSERLCRSTRQRAACAQLNDFLQEYGTQ
GCQV
>sp|P0C0L4|CO4A_HUMAN Complement C4-A OS=Homo sapiens OX=9606 GN=C4A PE=1 SV=2
MRLLWGLIWASSFFTLSLQKPRLLLFSPSVVHLGVPLSVGVQLQDVPRGQVVKGSVFLRN
PSRNNVPCSPKVDFTLSSERDFALLSLQVPLKDAKSCGLHQLLRGPEVQLVAHSPWLKDS
LSRTTNIQGINLLFSSRRGHLFLQTDQPIYNPGQRVRYRVFALDQKMRPSTDTITVMVEN
SHGLRVRKKEVYMPSSIFQDDFVIPDISEPGTWKISARFSDGLESNSSTQFEVKKYVLPN
FEVKITPGKPYILTVPGHLDEMQLDIQARYIYGKPVQGVAYVRFGLLDEDGKKTFFRGLE
SQTKLVNGQSHISLSKAEFQDALEKLNMGITDLQGLRLYVAAAIIESPGGEMEEAELTSW
YFVSSPFSLDLSKTKRHLVPGAPFLLQALVREMSGSPASGIPVKVSATVSSPGSVPEVQD
IQQNTDGSGQVSIPIIIPQTISELQLSVSAGSPHPAIARLTVAAPPSGGPGFLSIERPDS
RPPRVGDTLNLNLRAVGSGATFSHYYYMILSRGQIVFMNREPKRTLTSVSVFVDHHLAPS
FYFVAFYYHGDHPVANSLRVDVQAGACEGKLELSVDGAKQYRNGESVKLHLETDSLALVA
LGALDTALYAAGSKSHKPLNMGKVFEAMNSYDLGCGPGGGDSALQVFQAAGLAFSDGDQW
TLSRKRLSCPKEKTTRKKRNVNFQKAINEKLGQYASPTAKRCCQDGVTRLPMMRSCEQRA
ARVQQPDCREPFLSCCQFAESLRKKSRDKGQAGLQRALEILQEEDLIDEDDIPVRSFFPE
NWLWRVETVDRFQILTLWLPDSLTTWEIHGLSLSKTKGLCVATPVQLRVFREFHLHLRLP
MSVRRFEQLELRPVLYNYLDKNLTVSVHVSPVEGLCLAGGGGLAQQVLVPAGSARPVAFS
VVPTAAAAVSLKVVARGSFEFPVGDAVSKVLQIEKEGAIHREELVYELNPLDHRGRTLEI
PGNSDPNMIPDGDFNSYVRVTASDPLDTLGSEGALSPGGVASLLRLPRGCGEQTMIYLAP
TLAASRYLDKTEQWSTLPPETKDHAVDLIQKGYMRIQQFRKADGSYAAWLSRDSSTWLTA
FVLKVLSLAQEQVGGSPEKLQETSNWLLSQQQADGSFQDPCPVLDRSMQGGLVGNDETVA
LTAFVTIALHHGLAVFQDEGAEPLKQRVEASISKANSFLGEKASAGLLGAHAAAITAYAL
TLTKAPVDLLGVAHNNLMAMAQETGDNLYWGSVTGSQSNAVSPTPAPRNPSDPMPQAPAL
WIETTAYALLHLLLHEGKAEMADQASAWLTRQGSFQGGFRSTQDTVIALDALSAYWIASH
TTEERGLNVTLSSTGRNGFKSHALQLNNRQIRGLEEELQFSLGSKINVKVGGNSKGTLKV
LRTYNVLDMKNTTCQDLQIEVTVKGHVEYTMEANEDYEDYEYDELPAKDDPDAPLQPVTP
LQLFEGRRNRRRREAPKVVEEQESRVHYTVCIWRNGKVGLSGMAIADVTLLSGFHALRAD
LEKLTSLSDRYVSHFETEGPHVLLYFDSVPTSRECVGFEAVQEVPVGLVQPASATLYDYY
NPERRCSVFYGAPSKSRLLATLCSAEVCQCAEGKCPRQRRALERGLQDEDGYRMKFACYY
PRVEYGFQVKVLREDSRAAFRLFETKITQVLHFTKDVKAAANQMRNFLVRASCRLRLEPG
KEYLIMGLDGATYDLEGHPQYLLDSNSWIEEMPSERLCRSTRQRAACAQLNDFLQEYGTQ
GCQV
>sp|P11169|GTR3_HUMAN Solute carrier family 2, facilitated glucose transporter member 3 OS=Homo sapiens OX=9606 GN=SLC2A3 PE=1 SV=1
MGTQKVTPALIFAITVATIGSFQFGYNTGVINAPEKIIKEFINKTLTDKGNAPPSEVLLT
SLWSLSVAIFSVGGMIGSFSVGLFVNRFGRRNSMLIVNLLAVTGGCFMGLCKVAKSVEML
ILGRLVIGLFCGLCTGFVPMYIGEISPTALRGAFGTLNQLGIVVGILVAQIFGLEFILGS
EELWPLLLGFTILPAILQSAALPFCPESPRFLLINRKEEENAKQILQRLWGTQDVSQDIQ
EMKDESARMSQEKQVTVLELFRVSSYRQPIIISIVLQLSQQLSGINAVFYYSTGIFKDAG
VQEPIYATIGAGVVNTIFTVVSLFLVERAGRRTLHMIGLGGMAFCSTLMTVSLLLKDNYN
GMSFVCIGAILVFVAFFEIGPGPIPWFIVAELFSQGPRPAAMAVAGCSNWTSNFLVGLLF

PSAAHYLGAYVFIIFTGFLITFLAFTFFKVPETRGRTFEDITRAFEGQAHGADRSGKDGV
MEMNSIEPAKETTTNV
>sp|Q8TDB8|GTR14_HUMAN Solute carrier family 2, facilitated glucose transporter member 14 OS=Homo sapiens OX=9606 GN=SLC2A14 PE=2 SV=1
MEFHNGGHVSGIGGFLVSLTSRMKPHTLAVTPALIFAITVATIGSFQFGYNTGVINAPET
IIKEFINKTLTDKANAPPSEVLLTNLWSLSVAIFSVGGMIGSFSVGLFVNRFGRRNSMLI
VNLLAATGGCLMGLCKIAESVEMLILGRLVIGLFCGLCTGFVPMYIGEISPTALRGAFGT
LNQLGIVIGILVAQIFGLELILGSEELWPVLLGFTILPAILQSAALPCCPESPRFLLINR
KKEENATRILQRLWGTQDVSQDIQEMKDESARMSQEKQVTVLELFRVSSYRQPIIISIVL
QLSQQLSGINAVFYYSTGIFKDAGVQQPIYATISAGVVNTIFTLLSLFLVERAGRRTLHM
IGLGGMAFCSTLMTVSLLLKNHYNGMSFVCIGAILVFVACFEIGPGPIPWFIVAELFSQG
PRPAAMAVAGCSNWTSNFLVGLLFPSAAYYLGAYVFIIFTGFLITFLAFTFFKVPETRGR
TFEDITRAFEGQAHGADRSGKDGVMGMNSIEPAKETTTNV
>sp|Q8WWI5|CTL1_HUMAN Choline transporter-like protein 1 OS=Homo sapiens OX=9606 GN=SLC44A1 PE=1 SV=1
MGCCSSASSAAQSSKREWKPLEDRSCTDIPWLLLFILFCIGMGFICGFSIATGAAARLVS
GYDSYGNICGQKNTKLEAIPNSGMDHTQRKYVFFLDPCNLDLINRKIKSVALCVAACPRQ
ELKTLSDVQKFAEINGSALCSYNLKPSEYTTSPKSSVLCPKLPVPASAPIPFFHRCAPVN
ISCYAKFAEALITFVSDNSVLHRLISGVMTSKEIILGLCLLSLVLSMILMVIIRYISRVL
VWILTILVILGSLGGTGVLWWLYAKQRRSPKETVTPEQLQIAEDNLRALLIYAISATVFT
VILFLIMLVMRKRVALTIALFHVAGKVFIHLPLLVFQPFWTFFALVLFWVYWIMTLLFLG
TTGSPVQNEQGFVEFKISGPLQYMWWYHVVGLIWISEFILACQQMTVAGAVVTYYFTRDK
RNLPFTPILASVNRLIRYHLGTVAKGSFIITLVKIPRMILMYIHSQLKGKENACARCVLK
SCICCLWCLEKCLNYLNQNAYTATAINSTNFCTSAKDAFVILVENALRVATINTVGDFML
FLGKVLIVCSTGLAGIMLLNYQQDYTVWVLPLIIVCLFAFLVAHCFLSIYEMVVDVLFLC
FAIDTKYNDGSPGREFYMDKVLMEFVENSRKAMKEAGKGGVADSRELKPMASGASSA
>sp|P0C0S5|H2AZ_HUMAN Histone H2A.Z OS=Homo sapiens OX=9606 GN=H2AZ1 PE=1 SV=2
MAGGKAGKDSGKAKTKAVSRSQRAGLQFPVGRIHRHLKSRTTSHGRVGATAAVYSAAILE
YLTAEVLELAGNASKDLKVKRITPRHLQLAIRGDEELDSLIKATIAGGGVIPHIHKSLIG
KKGQQKTV
>sp|Q71UI9|H2AV_HUMAN Histone H2A.V OS=Homo sapiens OX=9606 GN=H2AZ2 PE=1 SV=3
MAGGKAGKDSGKAKAKAVSRSQRAGLQFPVGRIHRHLKTRTTSHGRVGATAAVYSAAILE
YLTAEVLELAGNASKDLKVKRITPRHLQLAIRGDEELDSLIKATIAGGGVIPHIHKSLIG
KKGQQKTA
>sp|Q15084|PDIA6_HUMAN Protein disulfide-isomerase A6 OS=Homo sapiens OX=9606 GN=PDIA6 PE=1 SV=1
MALLVLGLVSCTFFLAVNGLYSSSDDVIELTPSNFNREVIQSDSLWLVEFYAPWCGHCQR
LTPEWKKAATALKDVVKVGAVDADKHHSLGGQYGVQGFPTIKIFGSNKNRPEDYQGGRTG
EAIVDAALSALRQLVKDRLGGRSGGYSSGKQGRSDSSSKKDVIELTDDSFDKNVLDSEDV
WMVEFYAPWCGHCKNLEPEWAAAASEVKEQTKGKVKLAAVDATVNQVLASRYGIRGFPTI
KIFQKGESPVDYDGGRTRSDIVSRALDLFSDNAPPPELLEIINEDIAKRTCEEHQLCVVA
VLPHILDTGAAGRNSYLEVLLKLADKYKKKMWGWLWTEAGAQSELETALGIGGFGYPAMA
AINARKMKFALLKGSFSEQGINEFLRELSFGRGSTAPVGGGAFPTIVEREPWDGRDGELP
VEDDIDLSDVELDDLGKDEL

>sp|Q99460|PSMD1_HUMAN 26S proteasome non-ATPase regulatory subunit 1 OS=Homo sapiens OX=9606 GN=PSMD1 PE=1 SV=2
MITSAAGIISLLDEDEPQLKEFALHKLNAVVNDFWAEISESVDKIEVLYEDEGFRSRQFA
ALVASKVFYHLGAFEESLNYALGAGDLFNVNDNSEYVETIIAKCIDHYTKQCVENADLPE
GEKKPIDQRLEGIVNKMFQRCLDDHKYKQAIGIALETRRLDVFEKTILESNDVPGMLAYS
LKLCMSLMQNKQFRNKVLRVLVKIYMNLEKPDFINVCQCLIFLDDPQAVSDILEKLVKED
NLLMAYQICFDLYESASQQFLSSVIQNLRTVGTPIASVPGSTNTGTVPGSEKDSDSMETE
EKTSSAFVGKTPEASPEPKDQTLKMIKILSGEMAIELHLQFLIRNNNTDLMILKNTKDAV
RNSVCHTATVIANSFMHCGTTSDQFLRDNLEWLARATNWAKFTATASLGVIHKGHEKEAL
QLMATYLPKDTSPGSAYQEGGGLYALGLIHANHGGDIIDYLLNQLKNASNDIVRHGGSLG
LGLAAMGTARQDVYDLLKTNLYQDDAVTGEAAGLALGLVMLGSKNAQAIEDMVGYAQETQ
HEKILRGLAVGIALVMYGRMEEADALIESLCRDKDPILRRSGMYTVAMAYCGSGNNKAIR
RLLHVAVSDVNDDVRRAAVESLGFILFRTPEQCPSVVSLLSESYNPHVRYGAAMALGICC
AGTGNKEAINLLEPMTNDPVNYVRQGALIASALIMIQQTEITCPKVNQFRQLYSKVINDK
HDDVMAKFGAILAQGILDAGGHNVTISLQSRTGHTHMPSVVGVLVFTQFWFWFPLSHFLS
LAYTPTCVIGLNKDLKMPKVQYKSNCKPSTFAYPAPLEVPKEKEKEKVSTAVLSITAKAK
KKEKEKEKKEEEKMEVDEAEKKEEKEKKKEPEPNFQLLDNPARVMPAQLKVLTMPETCRY
QPFKPLSIGGIIILKDTSEDIEELVEPVAAHGPKIEEEEQEPEPPEPFEYIDD
>sp|Q7L5L3|GDPD3_HUMAN Lysophospholipase D GDPD3 OS=Homo sapiens OX=9606 GN=GDPD3 PE=1 SV=3
MSLLLYYALPALGSYAMLSIFFLRRPHLLHTPRAPTFRIRLGAHRGGSGELLENTMEAME
NSMAQRSDLLELDCQLTRDRVVVVSHDENLCRQSGLNRDVGSLDFEDLPLYKEKLEVYFS
PGHFAHGSDRRMVRLEDLFQRFPRTPMSVEIKGKNEELIREIAGLVRRYDRNEITIWASE
KSSVMKKCKAANPEMPLSFTISRGFWVLLSYYLGLLPFIPIPEKFFFCFLPNIINRTYFP
FSCSCLNQLLAVVSKWLIMRKSLIRHLEERGVQVVFWCLNEESDFEAAFSVGATGVITDY
PTALRHYLDNHGPAARTS
>sp|P01766|HV313_HUMAN Immunoglobulin heavy variable 3-13 OS=Homo sapiens OX=9606 GN=IGHV3-13 PE=1 SV=2
MELGLSWVFLVAILEGVQCEVQLVESGGGLVQPGGSLRLSCAASGFTFSSYDMHWVRQAT
GKGLEWVSAIGTAGDPYYPGSVKGRFTISRENAKNSLYLQMNSLRAGDTAVYYCAR
>sp|P01763|HV348_HUMAN Immunoglobulin heavy variable 3-48 OS=Homo sapiens OX=9606 GN=IGHV3-48 PE=1 SV=2
MELGLCWVFLVAILEGVQCEVQLVESGGGLVQPGGSLRLSCAASGFTFSSYEMNWVRQAP
GKGLEWVSYISSSGSTIYYADSVKGRFTISRDNAKNSLYLQMNSLRAEDTAVYYCAR
>sp|A0A0B4J1X5|HV374_HUMAN Immunoglobulin heavy variable 3-74 OS=Homo sapiens OX=9606 GN=IGHV3-74 PE=3 SV=1
MEFGLSWVFLVAILKGVQCEVQLVESGGGLVQPGGSLRLSCAASGFTFSSYWMHWVRQAP
GKGLVWVSRINSDGSSTSYADSVKGRFTISRDNAKNTLYLQMNSLRAEDTAVYYCAR
>sp|A0A0B4J1Y9|HV372_HUMAN Immunoglobulin heavy variable 3-72 OS=Homo sapiens OX=9606 GN=IGHV3-72 PE=3 SV=1
MEFGLSWVFLVVILQGVQCEVQLVESGGGLVQPGGSLRLSCAASGFTFSDHYMDWVRQAP
GKGLEWVGRTRNKANSYTTEYAASVKGRFTISRDDSKNSLYLQMNSLKTEDTAVYYCAR
>sp|P01780|HV307_HUMAN Immunoglobulin heavy variable 3-7 OS=Homo sapiens OX=9606 GN=IGHV3-7 PE=1 SV=2
MELGLSWVFLVAILEGVQCEVQLVESGGGLVQPGGSLRLSCAASGFTFSSYWMSWVRQAP
GKGLEWVANIKQDGSEKYYVDSVKGRFTISRDNAKNSLYLQMNSLRAEDTAVYYCAR

>sp|P01764|HV323_HUMAN Immunoglobulin heavy variable 3-23 OS=Homo sapiens OX=9606 GN=IGHV3-23 PE=1 SV=2
MEFGLSWLFLVAILKGVQCEVQLVESGGGLVQPGGSLRLSCAASGFTFSSYAMSWVRQAP
GKGLEWVSAISGSGGSTYYADSVKGRFTISRDNSKNTLYLQMNSLRAEDTAVYYCAK
>sp|P0DP57|SLUR2_HUMAN Secreted Ly-6/uPAR domain-containing protein 2 OS=Homo sapiens OX=9606 GN=SLURP2 PE=1 SV=1
MQLGTGLLLAAVLSLQLAAAEAIWCHQCTGFGGCSHGSRCLRDSTHCVTTATRVLSNTED
LPLVTKMCHIGCPDIPSLGLGPYVSIACCQTSLCNHD
>sp|P12429|ANXA3_HUMAN Annexin A3 OS=Homo sapiens OX=9606 GN=ANXA3 PE=1 SV=3
MASIWVGHRGTVRDYPDFSPSVDAEAIQKAIRGIGTDEKMLISILTERSNAQRQLIVKEY
QAAYGKELKDDLKGDLSGHFEHLMVALVTPPAVFDAKQLKKSMKGAGTNEDALIEILTTR
TSRQMKDISQAYYTVYKKSLGDDISSETSGDFRKALLTLADGRRDESLKVDEHLAKQDAQ
ILYKAGENRWGTDEDKFTEILCLRSFPQLKLTFDEYRNISQKDIVDSIKGELSGHFEDLL
LAIVNCVRNTPAFLAERLHRALKGIGTDEFTLNRIMVSRSEIDLLDIRTEFKKHYGYSLY
SAIKSDTSGDYEITLLKICGGDD
>sp|Q14210|LY6D_HUMAN Lymphocyte antigen 6D OS=Homo sapiens OX=9606 GN=LY6D PE=1 SV=1
MRTALLLLAALAVATGPALTLRCHVCTSSSNCKHSVVCPASSRFCKTTNTVEPLRGNLVK
KDCAESCTPSYTLQGQVSSGTSSTQCCQEDLCNEKLHNAAPTRTALAHSALSLGLALSLL
AVILAPSL
>sp|P09110|THIK_HUMAN 3-ketoacyl-CoA thiolase, peroxisomal OS=Homo sapiens OX=9606 GN=ACAA1 PE=1 SV=2
MQRLQVVLGHLRGPADSGWMPQAAPCLSGAPQASAADVVVVHGRRTAICRAGRGGFKDTT
PDELLSAVMTAVLKDVNLRPEQLGDICVGNVLQPGAGAIMARIAQFLSDIPETVPLSTVN
RQCSSGLQAVASIAGGIRNGSYDIGMACGVESMSLADRGNPGNITSRLMEKEKARDCLIP
MGITSENVAERFGISREKQDTFALASQQKAARAQSKGCFQAEIVPVTTTVHDDKGTKRSI
TVTQDEGIRPSTTMEGLAKLKPAFKKDGSTTAGNSSQVSDGAAAILLARRSKAEELGLPI
LGVLRSYAVVGVPPDIMGIGPAYAIPVALQKAGLTVSDVDIFEINEAFASQAAYCVEKLR
LPPEKVNPLGGAVALGHPLGCTGARQVITLLNELKRRGKRAYGVVSMCIGTGMGAAAVFE
YPGN
>sp|Q9HC38|GLOD4_HUMAN Glyoxalase domain-containing protein 4 OS=Homo sapiens OX=9606 GN=GLOD4 PE=1 SV=1
MAARRALHFVFKVGNRFQTARFYRDVLGMKVESCSVARLECSGAISAHCSDYTRITEDSF
SKPYDGKWSKTMVGFGPEDDHFVAELTYNYGVGDYKLGNDFMGITLASSQAVSNARKLEW
PLTEVAEGVFETEAPGGYKFYLQNRSLPQSDPVLKVTLAVSDLQKSLNYWCNLLGMKIYE
KDEEKQRALLGYADNQCKLELQGVKGGVDHAAAFGRIAFSCPQKELPDLEDLMKRENQKI
LTPLVSLDTPGKATVQVVILADPDGHEICFVGDEAFRELSKMDPEGSKLLDDAMAADKSD
EWFAKHNKPKASG
>sp|O00154|BACH_HUMAN Cytosolic acyl coenzyme A thioester hydrolase OS=Homo sapiens OX=9606 GN=ACOT7 PE=1 SV=3
MKLLARALRLCEFGRQASSRRLVAGQGCVGPRRGCCAPVQVVGPRADLPPCGACITGRIM
RPDDANVAGNVHGGTILKMIEEAGAIISTRHCNSQNGERCVAALARVERTDFLSPMCIGE
VAHVSAEITYTSKHSVEVQVNVMSENILTGAKKLTNKATLWYVPLSLKNVDKVLEVPPVV
YSRQEQEEEGRKRYEAQKLERMETKWRNGDIVQPVLNPEPNTVSYSQSSLIHLVGPSDCT
LHGFVHGGVTMKLMDEVAGIVAARHCKTNIVTASVDAINFHDKIRKGCVITISGRMTFTS
NKSMEIEVLVDADPVVDSSQKRYRAASAFFTYVSLSQEGRSLPVPQLVPETEDEKKRFEE

GKGRYLQMKAKRQGHAEPQP
>sp|P63241|IF5A1_HUMAN Eukaryotic translation initiation factor 5A-1 OS=Homo sapiens OX=9606 GN=EIF5A PE=1 SV=2
MADDLDFETGDAGASATFPMQCSALRKNGFVVLKGRPCKIVEMSTSKTGKHGHAKVHLVG
IDIFTGKKYEDICPSTHNMDVPNIKRNDFQLIGIQDGYLSLLQDSGEVREDLRLPEGDLG
KEIEQKYDCGEEILITVLSAMTEEAAVAIKAMAK
>sp|Q6IS14|IF5AL_HUMAN Eukaryotic translation initiation factor 5A-1-like OS=Homo sapiens OX=9606 GN=EIF5AL1 PE=2 SV=2
MADDLDFETGDAGASATFPMQCSALRKNGFVVLKGWPCKIVEMSASKTGKHGHAKVHLVG
IDIFTGKKYEDICPSTHNMDVPNIKRNDFQLIGIQDGYLSLLQDSGEVPEDLRLPEGDLG
KEIEQKYDCGEEILITVLSAMTEEAAVAIKAMAK
>sp|Q9GZV4|IF5A2_HUMAN Eukaryotic translation initiation factor 5A-2 OS=Homo sapiens OX=9606 GN=EIF5A2 PE=1 SV=3
MADEIDFTTGDAGASSTYPMQCSALRKNGFVVLKGRPCKIVEMSTSKTGKHGHAKVHLVG
IDIFTGKKYEDICPSTHNMDVPNIKRNDYQLICIQDGYLSLLTETGEVREDLKLPEGELG
KEIEGKYNAGEDVQVSVMCAMSEEYAVAIKPCK
>sp|P30044|PRDX5_HUMAN Peroxiredoxin-5, mitochondrial OS=Homo sapiens OX=9606 GN=PRDX5 PE=1 SV=4
MGLAGVCALRRSAGYILVGGAGGQSAAAAARRYSEGEWASGGVRSFSRAAAAMAPIKVGD
AIPAVEVFEGEPGNKVNLAELFKGKKGVLFGVPGAFTPGCSKTHLPGFVEQAEALKAKGV
QVVACLSVNDAFVTGEWGRAHKAEGKVRLLADPTGAFGKETDLLLDDSLVSIFGNRRLKR
FSMVVQDGIVKALNVEPDGTGLTCSLAPNIISQL
>sp|Q69YW2|STUM_HUMAN Protein stum homolog OS=Homo sapiens OX=9606 GN=STUM PE=1 SV=1
MEPSHKDAETAAAAAAVAAADPRGASSSSGVVVQVREKKGPLRAAIPYMPFPVAVICLFL
NTFVPGLGTFVSAFTVLCGARTDLPDRHVCCVFWLNIAAALIQILTAIVMVGWIMSIFWG
MDMVILAISQGYKEQGIPQQL
>sp|Q02978|M2OM_HUMAN Mitochondrial 2-oxoglutarate/malate carrier protein OS=Homo sapiens OX=9606 GN=SLC25A11 PE=1 SV=3
MAATASAGAGGIDGKPRTSPKSVKFLFGGLAGMGATVFVQPLDLVKNRMQLSGEGAKTRE
YKTSFHALTSILKAEGLRGIYTGLSAGLLRQATYTTTRLGIYTVLFERLTGADGTPPGFL
LKAVIGMTAGATGAFVGTPAEVALIRMTADGRLPADQRRGYKNVFNALIRITREEGVLTL
WRGCIPTMARAVVVNAAQLASYSQSKQFLLDSGYFSDNILCHFCASMISGLVTTAASMPV
DIAKTRIQNMRMIDGKPEYKNGLDVLFKVVRYEGFFSLWKGFTPYYARLGPHTVLTFIFL
EQMNKAYKRLFLSG
>sp|Q16881|TRXR1_HUMAN Thioredoxin reductase 1, cytoplasmic OS=Homo sapiens OX=9606 GN=TXNRD1 PE=1 SV=3
MGCAEGKAVAAAAPTELQTKGKNGDGRRRSAKDHHPGKTLPENPAGFTSTATADSRALLQ
AYIDGHSVVIFSRSTCTRCTEVKKLFKSLCVPYFVLELDQTEDGRALEGTLSELAAETDL
PVVFVKQRKIGGHGPTLKAYQEGRLQKLLKMNGPEDLPKSYDYDLIIIGGGSGGLAAAKE
AAQYGKKVMVLDFVTPTPLGTRWGLGGTCVNVGCIPKKLMHQAALLGQALQDSRNYGWKV
EETVKHDWDRMIEAVQNHIGSLNWGYRVALREKKVVYENAYGQFIGPHRIKATNNKGKEK
IYSAERFLIATGERPRYLGIPGDKEYCISSDDLFSLPYCPGKTLVVGASYVALECAGFLA
GIGLDVTVMVRSILLRGFDQDMANKIGEHMEEHGIKFIRQFVPIKVEQIEAGTPGRLRVV
AQSTNSEEIIEGEYNTVMLAIGRDACTRKIGLETVGVKINEKTGKIPVTDEEQTNVPYIY
AIGDILEDKVELTPVAIQAGRLLAQRLYAGSTVKCDYENVPTTVFTPLEYGACGLSEEKA
VEKFGEENIEVYHSYFWPLEWTIPSRDNNKCYAKIICNTKDNERVVGFHVLGPNAGEVTQ

GFAAALKCGLTKKQLDSTIGIHPVCAEVFTTLSVTKRSGASILQAGCUG
>sp|P46776|RL27A_HUMAN 60S ribosomal protein L27a OS=Homo sapiens OX=9606 GN=RPL27A PE=1 SV=2
MPSRLRKTRKLRGHVSHGHGRIGKHRKHPGGRGNAGGLHHHRINFDKYHPGYFGKVGMKH
YHLKRNQSFCPTVNLDKLWTLVSEQTRVNAAKNKTGAAPIIDVVRSGYYKVLGKGKLPKQ
PVIVKAKFFSRRAEEKIKSVGGACVLVA
>sp|Q9Y639|NPTN_HUMAN Neuroplastin OS=Homo sapiens OX=9606 GN=NPTN PE=1 SV=2
MSGSSLPSALALSLLLVSGSLLPGPGAAQNAGFVKSPMSETKLTGDAFELYCDVVGSPTP
EIQWWYAEVNRAESFRQLWDGARKRRVTVNTAYGSNGVSVLRITRLTLEDSGTYECRASN
DPKRNDLRQNPSITWIRAQATISVLQKPRIVTSEEVIIRDSPVLPVTLQCNLTSSSHTLT
YSYWTKNGVELSATRKNASNMEYRINKPRAEDSGEYHCVYHFVSAPKANATIEVKAAPDI
TGHKRSENKNEGQDATMYCKSVGYPHPDWIWRKKENGMPMDIVNTSGRFFIINKENYTEL
NIVNLQITEDPGEYECNATNAIGSASVVTVLRVRSHLAPLWPFLGILAEIIILVVIIVVY
EKRKRPDEVPDDDEPAGPMKTNSTNNHKDKNLRQRNTN
>sp|P22626|ROA2_HUMAN Heterogeneous nuclear ribonucleoproteins A2/B1 OS=Homo sapiens OX=9606 GN=HNRNPA2B1 PE=1 SV=2
MEKTLETVPLERKKREKEQFRKLFIGGLSFETTEESLRNYYEQWGKLTDCVVMRDPASKR
SRGFGFVTFSSMAEVDAAMAARPHSIDGRVVEPKRAVAREESGKPGAHVTVKKLFVGGIK
EDTEEHHLRDYFEEYGKIDTIEIITDRQSGKKRGFGFVTFDDHDPVDKIVLQKYHTINGH
NAEVRKALSRQEMQEVQSSRSGRGGNFGFGDSRGGGGNFGPGPGSNFRGGSDGYGSGRGF
GDGYNGYGGGPGGGNFGGSPGYGGGRGGYGGGGPGYGNQGGGYGGGYDNYGGGNYGSGNY
NDFGNYNQQPSNYGPMKSGNFGGSRNMGGPYGGGNYGPGGSGGSGGYGGRSRY
>sp|O94919|ENDD1_HUMAN Endonuclease domain-containing 1 protein OS=Homo sapiens OX=9606 GN=ENDOD1 PE=1 SV=2
MGTARWLALGSLFALAGLLEGRLVGEEEAGFGECDKFFYAGTPPAGLAADSHVKICQRAE
GAERFATLYSTRDRIPVYSAFRAPRPAPGGAEQRWLVEPQIDDPNSNLEEAINEAEAITS
VNSLGSKQALNTDYLDSDYQRGQLYPFSLSSDVQVATFTLTNSAPMTQSFQERWYVNLHS
LMDRALTPQCGSGEDLYILTGTVPSDYRVKDKVAVPEFVWLAACCAVPGGGWAMGFVKHT
RDSDIIEDVMVKDLQKLLPFNPQLFQNNCGETEQDTEKMKKILEVVNQIQDEERMVQSQK
SSSPLSSTRSKRSTLLPPEASEGSSSFLGKLMGFIATPFIKLFQLIYYLVVAILKNIVYF
LWCVTKQVINGIESCLYRLGSATISYFMAIGEELVSIPWKVLKVVAKVIRALLRILCCLL
KAICRVLSIPVRVLVDVATFPVYTMGAIPIVCKDIALGLGGTVSLLFDTAFGTLGGLFQV
VFSVCKRIGYKVTFDNSGEL
>sp|P55884|EIF3B_HUMAN Eukaryotic translation initiation factor 3 subunit B OS=Homo sapiens OX=9606 GN=EIF3B PE=1 SV=3
MQDAENVAVPEAAEERAEPGQQQPAAEPPPAEGLLRPAGPGAPEAAGTEASSEEVGIAEA
GPESEVRTEPAAEAEAASGPSESPSPPAAEELPGSHAEPPVPAQGEAPGEQARDERSDSR
AQAVSEDAGGNEGRAAEAEPRALENGDADEPSFSDPEDFVDDVSEEELLGDVLKDRPQEA
DGIDSVIVVDNVPQVGPDRLEKLKNVIHKIFSKFGKITNDFYPEEDGKTKGYIFLEYASP
AHAVDAVKNADGYKLDKQHTFRVNLFTDFDKYMTISDEWDIPEKQPFKDLGNLRYWLEEA
ECRDQYSVIFESGDRTSIFWNDVKDPVSIEERARWTETYVRWSPKGTYLATFHQRGIALW
GGEKFKQIQRFSHQGVQLIDFSPCERYLVTFSPLMDTQDDPQAIIIWDILTGHKKRGFHC
ESSAHWPIFKWSHDGKFFARMTLDTLSIYETPSMGLLDKKSLKISGIKDFSWSPGGNIIA
FWVPEDKDIPARVTLMQLPTRQEIRVRNLFNVVDCKLHWQKNGDYLCVKVDRTPKGTQGV
VTNFEIFRMREKQVPVDVVEMKETIIAFAWEPNGSKFAVLHGEAPRISVSFYHVKNNGKI
ELIKMFDKQQANTIFWSPQGQFVVLAGLRSMNGALAFVDTSDCTVMNIAEHYMASDVEWD

PTGRYVVTSVSWWSHKVDNAYWLWTFQGRLLQKNNKDRFCQLLWRPRPPTLLSQEQIKQI
KKDLKKYSKIFEQKDRLSQSKASKELVERRRTMMEDFRKYRKMAQELYMEQKNERLELRG
GVDTDELDSNVDDWEEETIEFFVTEEIIPLGNQE
>sp|Q5VSP4|LC1L1_HUMAN Putative lipocalin 1-like protein 1 OS=Homo sapiens OX=9606 GN=LCN1P1 PE=5 SV=1
MKPLLLAISLSLIAALQAHHLLASDEEIQDVSGTWYLKAMTVDRELPEMNLESVTPMTLT
ILEGGNLEAKATMLISGQCQEVKVILEKTDEPGKYTANRGKHVAYIIRSHMKDHYIFYCE
GRDPENNLEALEDFEKAAGARGLSTESILIPRQSETCSPGSD
>sp|P31025|LCN1_HUMAN Lipocalin-1 OS=Homo sapiens OX=9606 GN=LCN1 PE=1 SV=1
MKPLLLAVSLGLIAALQAHHLLASDEEIQDVSGTWYLKAMTVDREFPEMNLESVTPMTLT
TLEGGNLEAKVTMLISGRCQEVKAVLEKTDEPGKYTADGGKHVAYIIRSHVKDHYIFYCE
GELHGKPVRGVKLVGRDPKNNLEALEDFEKAAGARGLSTESILIPRQSETCSPGSD
>sp|Q9NZ01|TECR_HUMAN Very-long-chain enoyl-CoA reductase OS=Homo sapiens OX=9606 GN=TECR PE=1 SV=1
MKHYEVEILDAKTREKLCFLDKVEPHATIAEIKNLFTKTHPQWYPARQSLRLDPKGKSLK
DEDVLQKLPVGTTATLYFRDLGAQISWVTVFLTEYAGPLFIYLLFYFRVPFIYGHKYDFT
SSRHTVVHLACICHSFHYIKRLLETLFVHRFSHGTMPLRNIFKNCTYYWGFAAWMAYYIN
HPLYTPPTYGAQQVKLALAIFVICQLGNFSIHMALRDLRPAGSKTRKIPYPTKNPFTWLF
LLVSCPNYTYEVGSWIGFAIMTQCLPVALFSLVGFTQMTIWAKGKHRSYLKEFRDYPPLR
MPIIPFLL
>sp|P14174|MIF_HUMAN Macrophage migration inhibitory factor OS=Homo sapiens OX=9606 GN=MIF PE=1 SV=4
MPMFIVNTNVPRASVPDGFLSELTQQLAQATGKPPQYIAVHVVPDQLMAFGGSSEPCALC
SLHSIGKIGGAQNRSYSKLLCGLLAERLRISPDRVYINYYDMNAANVGWNNSTFA
>sp|P62191|PRS4_HUMAN 26S proteasome regulatory subunit 4 OS=Homo sapiens OX=9606 GN=PSMC1 PE=1 SV=1
MGQSQSGGHGPGGGKKDDKDKKKKYEPPVPTRVGKKKKKTKGPDAASKLPLVTPHTQCRL
KLLKLERIKDYLLMEEEFIRNQEQMKPLEEKQEEERSKVDDLRGTPMSVGTLEEIIDDNH
AIVSTSVGSEHYVSILSFVDKDLLEPGCSVLLNHKVHAVIGVLMDDTDPLVTVMKVEKAP
QETYADIGGLDNQIQEIKESVELPLTHPEYYEEMGIKPPKGVILYGPPGTGKTLLAKAVA
NQTSATFLRVVGSELIQKYLGDGPKLVRELFRVAEEHAPSIVFIDEIDAIGTKRYDSNSG
GEREIQRTMLELLNQLDGFDSRGDVKVIMATNRIETLDPALIRPGRIDRKIEFPLPDEKT
KKRIFQIHTSRMTLADDVTLDDLIMAKDDLSGADIKAICTEAGLMALRERRMKVTNEDFK
KSKENVLYKKQEGTPEGLYL
>sp|P62195|PRS8_HUMAN 26S proteasome regulatory subunit 8 OS=Homo sapiens OX=9606 GN=PSMC5 PE=1 SV=1
MALDGPEQMELEEGKAGSGLRQYYLSKIEELQLIVNDKSQNLRRLQAQRNELNAKVRLLR
EELQLLQEQGSYVGEVVRAMDKKKVLVKVHPEGKFVVDVDKNIDINDVTPNCRVALRNDS
YTLHKILPNKVDPLVSLMMVEKVPDSTYEMIGGLDKQIKEIKEVIELPVKHPELFEALGI
AQPKGVLLYGPPGTGKTLLARAVAHHTDCTFIRVSGSELVQKFIGEGARMVRELFVMARE
HAPSIIFMDEIDSIGSSRLEGGSGGDSEVQRTMLELLNQLDGFEATKNIKVIMATNRIDI
LDSALLRPGRIDRKIEFPPPNEEARLDILKIHSRKMNLTRGINLRKIAELMPGASGAEVK
GVCTEAGMYALRERRVHVTQEDFEMAVAKVMQKDSEKNMSIKKLWK
>sp|Q99536|VAT1_HUMAN Synaptic vesicle membrane protein VAT-1 homolog OS=Homo sapiens OX=9606 GN=VAT1 PE=1 SV=2
MSDEREVAEAATGEDASSPPPKTEAASDPQHPAASEGAAAAAASPPLLRCLVLTGFGGYD

KVKLQSRPAAPPAPGPGQLTLRLRACGLNFADLMARQGLYDRLPPLPVTPGMEGAGVVIA
VGEGVSDRKAGDRVMVLNRSGMWQEEVTVPSVQTFLIPEAMTFEEAAALLVNYITAYMVL
FDFGNLQPGHSVLVHMAAGGVGMAAVQLCRTVENVTVFGTASASKHEALKENGVTHPIDY
HTTDYVDEIKKISPKGVDIVMDPLGGSDTAKGYNLLKPMGKVVTYGMANLLTGPKRNLMA
LARTWWNQFSVTALQLLQANRAVCGFHLGYLDGEVELVSGVVARLLALYNQGHIKPHIDS
VWPFEKVADAMKQMQEKKNVGKVLLVPGPEKEN
>sp|P16520|GBB3_HUMAN Guanine nucleotide-binding protein G(I)/G(S)/G(T) subunit beta-3 OS=Homo sapiens OX=9606 GN=GNB3 PE=1 SV=1
MGEMEQLRQEAEQLKKQIADARKACADVTLAELVSGLEVVGRVQMRTRRTLRGHLAKIYA
MHWATDSKLLVSASQDGKLIVWDSYTTNKVHAIPLRSSWVMTCAYAPSGNFVACGGLDNM
CSIYNLKSREGNVKVSRELSAHTGYLSCCRFLDDNNIVTSSGDTTCALWDIETGQQKTVF
VGHTGDCMSLAVSPDFNLFISGACDASAKLWDVREGTCRQTFTGHESDINAICFFPNGEA
ICTGSDDASCRLFDLRADQELICFSHESIICGITSVAFSLSGRLLFAGYDDFNCNVWDSM
KSERVGILSGHDNRVSCLGVTADGMAVATGSWDSFLKIWN
>sp|P62873|GBB1_HUMAN Guanine nucleotide-binding protein G(I)/G(S)/G(T) subunit beta-1 OS=Homo sapiens OX=9606 GN=GNB1 PE=1 SV=3
MSELDQLRQEAEQLKNQIRDARKACADATLSQITNNIDPVGRIQMRTRRTLRGHLAKIYA
MHWGTDSRLLVSASQDGKLIIWDSYTTNKVHAIPLRSSWVMTCAYAPSGNYVACGGLDNI
CSIYNLKTREGNVRVSRELAGHTGYLSCCRFLDDNQIVTSSGDTTCALWDIETGQQTTTF
TGHTGDVMSLSLAPDTRLFVSGACDASAKLWDVREGMCRQTFTGHESDINAICFFPNGNA
FATGSDDATCRLFDLRADQELMTYSHDNIICGITSVSFSKSGRLLLAGYDDFNCNVWDAL
KADRAGVLAGHDNRVSCLGVTDDGMAVATGSWDSFLKIWN
>sp|P62879|GBB2_HUMAN Guanine nucleotide-binding protein G(I)/G(S)/G(T) subunit beta-2 OS=Homo sapiens OX=9606 GN=GNB2 PE=1 SV=3
MSELEQLRQEAEQLRNQIRDARKACGDSTLTQITAGLDPVGRIQMRTRRTLRGHLAKIYA
MHWGTDSRLLVSASQDGKLIIWDSYTTNKVHAIPLRSSWVMTCAYAPSGNFVACGGLDNI
CSIYSLKTREGNVRVSRELPGHTGYLSCCRFLDDNQIITSSGDTTCALWDIETGQQTVGF
AGHSGDVMSLSLAPDGRTFVSGACDASIKLWDVRDSMCRQTFIGHESDINAVAFFPNGYA
FTTGSDDATCRLFDLRADQELLMYSHDNIICGITSVAFSRSGRLLLAGYDDFNCNIWDAM
KGDRAGVLAGHDNRVSCLGVTDDGMAVATGSWDSFLKIWN
>sp|Q9HAV0|GBB4_HUMAN Guanine nucleotide-binding protein subunit beta-4 OS=Homo sapiens OX=9606 GN=GNB4 PE=1 SV=3
MSELEQLRQEAEQLRNQIQDARKACNDATLVQITSNMDSVGRIQMRTRRTLRGHLAKIYA
MHWGYDSRLLVSASQDGKLIIWDSYTTNKMHAIPLRSSWVMTCAYAPSGNYVACGGLDNI
CSIYNLKTREGNVRVSRELPGHTGYLSCCRFLDDSQIVTSSGDTTCALWDIETAQQTTTF
TGHSGDVMSLSLSPDMRTFVSGACDASSKLWDIRDGMCRQSFTGHVSDINAVSFFPNGYA
FATGSDDATCRLFDLRADQELLLYSHDNIICGITSVAFSKSGRLLLAGYDDFNCNVWDTL
KGDRAGVLAGHDNRVSCLGVTDDGMAVATGSWDSFLRIWN
>sp|P61019|RAB2A_HUMAN Ras-related protein Rab-2A OS=Homo sapiens OX=9606 GN=RAB2A PE=1 SV=1
MAYAYLFKYIIIGDTGVGKSCLLLQFTDKRFQPVHDLTIGVEFGARMITIDGKQIKLQIW
DTAGQESFRSITRSYYRGAAGALLVYDITRRDTFNHLTTWLEDARQHSNSNMVIMLIGNK
SDLESRREVKKEEGEAFAREHGLIFMETSAKTASNVEEAFINTAKEIYEKIQEGVFDINN
EANGIKIGPQHAATNATHAGNQGGQQAGGGCC

>sp|Q8WUD1|RAB2B_HUMAN Ras-related protein Rab-2B OS=Homo sapiens OX=9606 GN=RAB2B PE=1 SV=1
MTYAYLFKYIIIGDTGVGKSCLLLQFTDKRFQPVHDLTIGVEFGARMVNIDGKQIKLQIW
DTAGQESFRSITRSYYRGAAGALLVYDITRRETFNHLTSWLEDARQHSSSNMVIMLIGNK
SDLESRRDVKREEGEAFAREHGLIFMETSAKTACNVEEAFINTAKEIYRKIQQGLFDVHN
EANGIKIGPQQSISTSVGPSASQRNSRDIGSNSGCC
>sp|Q12905|ILF2_HUMAN Interleukin enhancer-binding factor 2 OS=Homo sapiens OX=9606 GN=ILF2 PE=1 SV=2
MRGDRGRGRGGRFGSRGGPGGGFRPFVPHIPFDFYLCEMAFPRVKPAPDETSFSEALLKR
NQDLAPNSAEQASILSLVTKINNVIDNLIVAPGTFEVQIEEVRQVGSYKKGTMTTGHNVA
DLVVILKILPTLEAVAALGNKVVESLRAQDPSEVLTMLTNETGFEISSSDATVKILITTV
PPNLRKLDPELHLDIKVLQSALAAIRHARWFEENASQSTVKVLIRLLKDLRIRFPGFEPL
TPWILDLLGHYAVMNNPTRQPLALNVAYRRCLQILAAGLFLPGSVGITDPCESGNFRVHT
VMTLEQQDMVCYTAQTLVRILSHGGFRKILGQEGDASYLASEISTWDGVIVTPSEKAYEK
PPEKKEGEEEEENTEEPPQGEEEESMETQE
>sp|Q9Y285|SYFA_HUMAN Phenylalanine--tRNA ligase alpha subunit OS=Homo sapiens OX=9606 GN=FARSA PE=1 SV=3
MADGQVAELLLRRLEASDGGLDSAELAAELGMEHQAVVGAVKSLQALGEVIEAELRSTKH
WELTAEGEEIAREGSHEARVFRSIPPEGLAQSELMRLPSGKVGFSKAMSNKWIRVDKSAA
DGPRVFRVVDSMEDEVQRRLQLVRGGQAEKLGEKERSELRKRKLLAEVTLKTYWVSKGSA
FSTSISKQETELSPEMISSGSWRDRPFKPYNFLAHGVLPDSGHLHPLLKVRSQFRQIFLE
MGFTEMPTDNFIESSFWNFDALFQPQQHPARDQHDTFFLRDPAEALQLPMDYVQRVKRTH
SQGGYGSQGYKYNWKLDEARKNLLRTHTTSASARALYRLAQKKPFTPVKYFSIDRVFRNE
TLDATHLAEFHQIEGVVADHGLTLGHLMGVLREFFTKLGITQLRFKPAYNPYTEPSMEVF
SYHQGLKKWVEVGNSGVFRPEMLLPMGLPENVSVIAWGLSLERPTMIKYGINNIRELVGH
KVNLQMVYDSPLCRLDAEPRPPPTQEAA
>sp|Q9Y4K1|CRBG1_HUMAN Beta/gamma crystallin domain-containing protein 1 OS=Homo sapiens OX=9606 GN=CRYBG1 PE=1 SV=3
MEKRSSGRRSGRRRGSQKSTDSPGADAELPESAARDDAVFDDEVAPNAASDNASAEKKVK
SPRAALDGGVASAASPESKPSPGTKGQLRGESDRSKQPPPASSPTKRKGRSRALEAVPAP
PASGPRAPAKESPPKRVPDPSPVTKGTAAESGEEAARAIPRELPVKSSSLLPEIKPEHKR
GPLPNHFNGRAEGGRSRELGRAAGAPGASDADGLKPRNHFGVGRSTVTTKVTLPAKPKHV
ELNLKTPKNLDSLGNEHNPFSQPVHKGNTATKISLFENKRTNSSPRHTDIRGQRNTPASS
KTFVGRAKLNLAKKAKEMEQPEKKVMPNSPQNGVLVKETAIETKVTVSEEEILPATRGMN
GDSSENQALGPQPNQDDKADVQTDAGCLSEPVASALIPVKDHKLLEKEDSEAADSKSLVL
ENVTDTAQDIPTTVDTKDLPPTAMPKPQHTFSDSQSPAESSPGPSLSLSAPAPGDVPKDT
CVQSPISSFPCTDLKVSENHKGCVLPVSRQNNEKMPLLELGGETTPPLSTERSPEAVGSE
CPSRVLVQVRSFVLPVESTQDVSSQVIPESSEVREVQLPTCHSNEPEVVSVASCAPPQEE
VLGNEHSHCTAELAAKSGPQVIPPASEKTLPIQAQSQGSRTPLMAESSPTNSPSSGNHLA
TPQRPDQTVTNGQDSPASLLNISAGSDDSVFDSSSDMEKFTEIIKQMDSAVCMPMKRKKA
RMPNSPAPHFAMPPIHEDHLEKVFDPKVFTFGLGKKKESQPEMSPALHLMQNLDTKSKLR
PKRASAEQSVLFKSLHTNTNGNSEPLVMPEINDKENRDVTNGGIKRSRLEKSALFSSLLS
SLPQDKIFSPSVTSVNTMTTAFSTSQNGSLSQSSVSQPTTEGAPPCGLNKEQSNLLPDNS
LKVFNFNSSSTSHSSLKSPSHMEKYPQKEKTKEDLDSRSNLHLPETKFSELSKLKNDDME
KANHIESVIKSNLPNCANSDTDFMGLFKSSRYDPSISFSGMSLSDTMTLRGSVQNKLNPR
PGKVVIYSEPDVSEKCIEVFSDIQDCSSWSLSPVILIKVVRGCWILYEQPNFEGHSIPLE
EGELELSGLWGIEDILERHEEAESDKPVVIGSIRHVVQDYRVSHIDLFTEPEGLGILSSY

FDDTEEMQGFGVMQKTCSMKVHWGTWLIYEEPGFQGVPFILEPGEYPDLSFWDTEEAYIG
SMRPLKMGGRKVEFPTDPKVVVYEKPFFEGKCVELETGMCSFVMEGGETEEATGDDHLPF
TSVGSMKVLRGIWVAYEKPGFTGHQYLLEEGEYRDWKAWGGYNGELQSLRPILGDFSNAH
MIMYSEKNFGSKGSSIDVLGIVANLKETGYGVKTQSINVLSGVWVAYENPDFTGEQYILD
KGFYTSFEDWGGKNCKISSVQPICLDSFTGPRRRNQIHLFSEPQFQGHSQSFEETTSQID
DSFSTKSCRVSGGSWVVYDGENFTGNQYVLEEGHYPCLSAMGCPPGATFKSLRFIDVEFS
EPTIILFEREDFKGKKIELNAETVNLRSLGFNTQIRSVQVIGGIWVTYEYGSYRGRQFLL
SPAEVPNWYEFSGCRQIGSLRPFVQKRIYFRLRNKATGLFMSTNGNLEDLKLLRIQVMED
VGADDQIWIYQEGCIKCRIAEDCCLTIVGSLVTSGSKLGLALDQNADSQFWSLKSDGRIY
SKLKPNLVLDIKGGTQYDQNHIILNTVSKEKFTQVWEAMVLYT
>sp|Q9H0P0|5NT3A_HUMAN Cytosolic 5'-nucleotidase 3A OS=Homo sapiens OX=9606 GN=NT5C3A PE=1 SV=3
MRAPSMDRAAVARVGAVASASVCALVAGVVLAQYIFTLKRKTGRKTKIIEMMPEFQKSSV
RIKNPTRVEEIICGLIKGGAAKLQIITDFDMTLSRFSYKGKRCPTCHNIIDNCKLVTDEC
RKKLLQLKEKYYAIEVDPVLTVEEKYPYMVEWYTKSHGLLVQQALPKAKLKEIVAESDVM
LKEGYENFFDKLQQHSIPVFIFSAGIGDVLEEVIRQAGVYHPNVKVVSNFMDFDETGVLK
GFKGELIHVFNKHDGALRNTEYFNQLKDNSNIILLGDSQGDLRMADGVANVEHILKIGYL
NDRVDELLEKYMDSYDIVLVQDESLEVANSILQKIL
>sp|P30153|2AAA_HUMAN Serine/threonine-protein phosphatase 2A 65 kDa regulatory subunit A alpha isoform OS=Homo sapiens OX=9606 GN=PPP2R1A PE=1 SV=4
MAAADGDDSLYPIAVLIDELRNEDVQLRLNSIKKLSTIALALGVERTRSELLPFLTDTIY
DEDEVLLALAEQLGTFTTLVGGPEYVHCLLPPLESLATVEETVVRDKAVESLRAISHEHS
PSDLEAHFVPLVKRLAGGDWFTSRTSACGLFSVCYPRVSSAVKAELRQYFRNLCSDDTPM
VRRAAASKLGEFAKVLELDNVKSEIIPMFSNLASDEQDSVRLLAVEACVNIAQLLPQEDL
EALVMPTLRQAAEDKSWRVRYMVADKFTELQKAVGPEITKTDLVPAFQNLMKDCEAEVRA
AASHKVKEFCENLSADCRENVIMSQILPCIKELVSDANQHVKSALASVIMGLSPILGKDN
TIEHLLPLFLAQLKDECPEVRLNIISNLDCVNEVIGIRQLSQSLLPAIVELAEDAKWRVR
LAIIEYMPLLAGQLGVEFFDEKLNSLCMAWLVDHVYAIREAATSNLKKLVEKFGKEWAHA
TIIPKVLAMSGDPNYLHRMTTLFCINVLSEVCGQDITTKHMLPTVLRMAGDPVANVRFNV
AKSLQKIGPILDNSTLQSEVKPILEKLTQDQDVDVKYFAQEALTVLSLA
>sp|P0DOX6|IGM_HUMAN Immunoglobulin mu heavy chain OS=Homo sapiens OX=9606 PE=1 SV=2
QVTLTESGPALVKPKQPLTLTCTFSGFSLSTSRMRVSWIRRPPGKALEWLARIDDDDKFY
WSTSLRTRLSISKNDSKNQVVLIMINVNPVDTATYYCARVVNSVMAGYYYYYMDVWGKGT
TVTVSSGSASAPTLFPLVSCENSNPSSTVAVGCLAQDFLPDSITFSWKYNQSQKISSTRG
FPSVLRGGKYAATSQVLLPSKDVMQGTDEHVCKWVQHPNGNKQKNVPLPVIAELPPKVSV
FVPPRDGFFGNPRKSKLICQATGFSPRQVWSLREGKQVGSGVTTDQVQAEAKESGPTTYK
VTSTLTIKESDWLGESMFTCRVDHRGLTFQQNASSMCVPDQDTAIRVFAIPPSFASIFLT
KSTKLTCLVTDLTTYDSVTISWTREENGAVKTHTNISESHPNATFSAVGEASICEDDDWS
GERFTCTVTHTDLPSPLKQTISRPKGVALHRPDVYLLPPAREQLNLRESATITCLVTGFS
PADVFVQWMQRGEPLSPEKYVTSAPMPEPQAPGRYFAHSILTVSEEEWNTGQTYTCVVAH
EALPNRVTERTVDKSTGKPTLYNVSLVMSDTAGTCY
>sp|Q9UI12|VATH_HUMAN V-type proton ATPase subunit H OS=Homo sapiens OX=9606 GN=ATP6V1H PE=1 SV=1
MTKMDIRGAVDAAVPTNIIAAKAAEVRANKVNWQSYLQGQMISAEDCEFIQRFEMKRSPE
EKQEMLQTEGSQCAKTFINLMTHICKEQTVQYILTMVDDMLQENHQRVSIFFDYARCSKN

TAWPYFLPMLNRQDPFTVHMAARIIAKLAAWGKELMEGSDLNYYFNWIKTQLSSQKLRGS
GVAVETGTVSSSDSSQYVQCVAGCLQLMLRVNEYRFAWVEADGVNCIMGVLSNKCGFQLQ
YQMIFSIWLLAFSPQMCEHLRRYNIIPVLSDILQESVKEKVTRIILAAFRNFLEKSTERE
TRQEYALAMIQCKVLKQLENLEQQKYDDEDISEDIKFLLEKLGESVQDLSSFDEYSSELK
SGRLEWSPVHKSEKFWRENAVRLNEKNYELLKILTKLLEVSDDPQVLAVAAHDVGEYVRH
YPRGKRVIEQLGGKQLVMNHMHHEDQQVRYNALLAVQKLMVHNWEYLGKQLQSEQPQTAA
ARS
>sp|Q9BSJ8|ESYT1_HUMAN Extended synaptotagmin-1 OS=Homo sapiens OX=9606 GN=ESYT1 PE=1 SV=1
MERSPGEGPSPSPMDQPSAPSDPTDQPPAAHAKPDPGSGGQPAGPGAAGEALAVLTSFGR
RLLVLIPVYLAGAVGLSVGFVLFGLALYLGWRRVRDEKERSLRAARQLLDDEEQLTAKTL
YMSHRELPAWVSFPDVEKAEWLNKIVAQVWPFLGQYMEKLLAETVAPAVRGSNPHLQTFT
FTRVELGEKPLRIIGVKVHPGQRKEQILLDLNISYVGDVQIDVEVKKYFCKAGVKGMQLH
GVLRVILEPLIGDLPFVGAVSMFFIRRPTLDINWTGMTNLLDIPGLSSLSDTMIMDSIAA
FLVLPNRLLVPLVPDLQDVAQLRSPLPRGIIRIHLLAARGLSSKDKYVKGLIEGKSDPYA
LVRLGTQTFCSRVIDEELNPQWGETYEVMVHEVPGQEIEVEVFDKDPDKDDFLGRMKLDV
GKVLQASVLDDWFPLQGGQGQVHLRLEWLSLLSDAEKLEQVLQWNWGVSSRPDPPSAAIL
VVYLDRAQDLPLKKGNKEPNPMVQLSIQDVTQESKAVYSTNCPVWEEAFRFFLQDPQSQE
LDVQVKDDSRALTLGALTLPLARLLTAPELILDQWFQLSSSGPNSRLYMKLVMRILYLDS
SEICFPTVPGCPGAWDVDSENPQRGSSVDAPPRPCHTTPDSQFGTEHVLRIHVLEAQDLI
AKDRFLGGLVKGKSDPYVKLKLAGRSFRSHVVREDLNPRWNEVFEVIVTSVPGQELEVEV
FDKDLDKDDFLGRCKVRLTTVLNSGFLDEWLTLEDVPSGRLHLRLERLTPRPTAAELEEV
LQVNSLIQTQKSAELAAALLSIYMERAEDLPLRKGTKHLSPYATLTVGDSSHKTKTISQT
SAPVWDESASFLIRKPHTESLELQVRGEGTGVLGSLSLPLSELLVADQLCLDRWFTLSSG
QGQVLLRAQLGILVSQHSGVEAHSHSYSHSSSSLSEEPELSGGPPHITSSAPELRQRLTH
VDSPLEAPAGPLGQVKLTLWYYSEERKLVSIVHGCRSLRQNGRDPPDPYVSLLLLPDKNR
GTKRRTSQKKRTLSPEFNERFEWELPLDEAQRRKLDVSVKSNSSFMSRERELLGKVQLDL
AETDLSQGVARWYDLMDNKDKGSS
>sp|A0A075B6P5|KV228_HUMAN Immunoglobulin kappa variable 2-28 OS=Homo sapiens OX=9606 GN=IGKV2-28 PE=3 SV=1
MRLPAQLLGLLMLWVSGSSGDIVMTQSPLSLPVTPGEPASISCRSSQSLLHSNGYNYLDW
YLQKPGQSPQLLIYLGSNRASGVPDRFSGSGSGTDFTLKISRVEAEDVGVYYCMQALQTP
>sp|P01615|KVD28_HUMAN Immunoglobulin kappa variable 2D-28 OS=Homo sapiens OX=9606 GN=IGKV2D-28 PE=1 SV=2
MRLPAQLLGLLMLWVSGSSGDIVMTQSPLSLPVTPGEPASISCRSSQSLLHSNGYNYLDW
YLQKPGQSPQLLIYLGSNRASGVPDRFSGSGSGTDFTLKISRVEAEDVGVYYCMQALQTP
>sp|A0A075B6S2|KVD29_HUMAN Immunoglobulin kappa variable 2D-29 OS=Homo sapiens OX=9606 GN=IGKV2D-29 PE=3 SV=1
MRLPAQLLGLLMLWIPGSSADIVMTQTPLSLSVTPGQPASISCKSSQSLLHSDGKTYLYW
YLQKPGQPPQLLIYEVSNRFSGVPDRFSGSGSGTDFTLKISRVEAEDVGVYYCMQSIQLP
>sp|A2NJV5|KV229_HUMAN Immunoglobulin kappa variable 2-29 OS=Homo sapiens OX=9606 GN=IGKV2-29 PE=3 SV=2
MRLPAQLLGLLMLWIPGSSADIVMTQTPLSLSVTPGQPASISCKSSQSLLHSDGKTYLYW
YLQKPGQSPQLLIYEVSSRFSGVPDRFSGSGSGTDFTLKISRVEAEDVGVYYCMQGIHLP
>sp|A0A0A0MRZ7|KVD26_HUMAN Immunoglobulin kappa variable 2D-26 OS=Homo sapiens OX=9606 GN=IGKV2D-26 PE=3 SV=1
MRLPAQLLGLLMLWVPGSSAEIVMTQTPLSLSITPGEQASMSCRSSQSLLHSDGYTYLYW

FLQKARPVSTLLIYEVSNRFSGVPDRFSGSGSGTDFTLKISRVEAEDFGVYYCMQDAQDP
>sp|P06310|KV230_HUMAN Immunoglobulin kappa variable 2-30 OS=Homo sapiens OX=9606 GN=IGKV2-30 PE=3 SV=2
MRLPAQLLGLLMLWVPGSSGDVVMTQSPLSLPVTLGQPASISCRSSQSLVYSDGNTYLNW
FQQRPGQSPRRLIYKVSNRDSGVPDRFSGSGSGTDFTLKISRVEAEDVGVYYCMQGTHWP
>sp|A0A075B6S6|KVD30_HUMAN Immunoglobulin kappa variable 2D-30 OS=Homo sapiens OX=9606 GN=IGKV2D-30 PE=3 SV=1
MRLPAQLLGLLMLWVPGSSGDVVMTQSPLSLPVTLGQPASISCRSSQSLVYSDGNTYLNW
FQQRPGQSPRRLIYKVSNWDSGVPDRFSGSGSGTDFTLKISRVEAEDVGVYYCMQGTHWP
>sp|P01614|KVD40_HUMAN Immunoglobulin kappa variable 2D-40 OS=Homo sapiens OX=9606 GN=IGKV2D-40 PE=1 SV=2
MRLPAQLLGLLMLWVPGSSEDIVMTQTPLSLPVTPGEPASISCRSSQSLLDSDDGNTYLD
WYLQKPGQSPQLLIYTLSYRASGVPDRFSGSGSGTDFTLKISRVEAEDVGVYYCMQRIEF
P
>sp|A0A087WW87|KV240_HUMAN Immunoglobulin kappa variable 2-40 OS=Homo sapiens OX=9606 GN=IGKV2-40 PE=3 SV=2
MRLPAQLLGLLMLWVPGSSEDIVMTQTPLSLPVTPGEPASISCRSSQSLLDSDDGNTYLD
WYLQKPGQSPQLLIYTLSYRASGVPDRFSGSGSGTDFTLKISRVEAEDVGVYYCMQRIEF
P
>sp|P61586|RHOA_HUMAN Transforming protein RhoA OS=Homo sapiens OX=9606 GN=RHOA PE=1 SV=1
MAAIRKKLVIVGDGACGKTCLLIVFSKDQFPEVYVPTVFENYVADIEVDGKQVELALWDT
AGQEDYDRLRPLSYPDTDVILMCFSIDSPDSLENIPEKWTPEVKHFCPNVPIILVGNKKD
LRNDEHTRRELAKMKQEPVKPEEGRDMANRIGAFGYMECSAKTKDGVREVFEMATRAALQ
ARRGKKKSGCLVL
>sp|P08134|RHOC_HUMAN Rho-related GTP-binding protein RhoC OS=Homo sapiens OX=9606 GN=RHOC PE=1 SV=1
MAAIRKKLVIVGDGACGKTCLLIVFSKDQFPEVYVPTVFENYIADIEVDGKQVELALWDT
AGQEDYDRLRPLSYPDTDVILMCFSIDSPDSLENIPEKWTPEVKHFCPNVPIILVGNKKD
LRQDEHTRRELAKMKQEPVRSEEGRDMANRISAFGYLECSAKTKEGVREVFEMATRAGLQ
VRKNKRRRGCPIL
>sp|O00194|RB27B_HUMAN Ras-related protein Rab-27B OS=Homo sapiens OX=9606 GN=RAB27B PE=1 SV=4
MTDGDYDYLIKLLALGDSGVGKTTFLYRYTDNKFNPKFITTVGIDFREKRVVYNAQGPNG
SSGKAFKVHLQLWDTAGQERFRSLTTAFFRDAMGFLLMFDLTSQQSFLNVRNWMSQLQAN
AYCENPDIVLIGNKADLPDQREVNERQARELADKYGIPYFETSAATGQNVEKAVETLLDL
IMKRMEQCVEKTQIPDTVNGGNSGNLDGEKPPEKKCIC
>sp|O95747|OXSR1_HUMAN Serine/threonine-protein kinase OSR1 OS=Homo sapiens OX=9606 GN=OXSR1 PE=1 SV=1
MSEDSSALPWSINRDDYELQEVIGSGATAVVQAAYCAPKKEKVAIKRINLEKCQTSMDEL
LKEIQAMSQCHHPNIVSYYTSFVVKDELWLVMKLLSGGSVLDIIKHIVAKGEHKSGVLDE
STIATILREVLEGLEYLHKNGQIHRDVKAGNILLGEDGSVQIADFGVSAFLATGGDITRN
KVRKTFVGTPCWMAPEVMEQVRGYDFKADIWSFGITAIELATGAAPYHKYPPMKVLMLTL
QNDPPSLETGVQDKEMLKKYGKSFRKMISLCLQKDPEKRPTAAELLRHKFFQKAKNKEFL
QEKTLQRAPTISERAKKVRRVPGSSGRLHKTEDGGWEWSDDEFDEESEEGKAAISQLRSP
RVKESISNSELFPTTDPVGTLLQVPEQISAHLPQPAGQIATQPTQVSLPPTAEPAKTAQA
LSSGSGSQETKIPISLVLRLRNSKKELNDIRFEFTPGRDTAEGVSQELISAGLVDGRDLV

IVAANLQKIVEEPQSNRSVTFKLASGVEGSDIPDDGKLIGFAQLSIS
>sp|P01772|HV333_HUMAN Immunoglobulin heavy variable 3-33 OS=Homo sapiens OX=9606 GN=IGHV3-33 PE=1 SV=2
MEFGLSWVFLVALLRGVQCQVQLVESGGGVVQPGRSLRLSCAASGFTFSSYGMHWVRQAP
GKGLEWVAVIWYDGSNKYYADSVKGRFTISRDNSKNTLYLQMNSLRAEDTAVYYCAR
>sp|P0DP03|HVC05_HUMAN Immunoglobulin heavy variable 3-30-5 OS=Homo sapiens OX=9606 GN=IGHV3-30-5 PE=3 SV=1
MEFGLSWVFLVALLRGVQCQVQLVESGGGVVQPGRSLRLSCAASGFTFSSYGMHWVRQAP
GKGLEWVAVISYDGSNKYYADSVKGRFTISRDNSKNTLYLQMNSLRAEDTAVYYCAK
>sp|P01768|HV330_HUMAN Immunoglobulin heavy variable 3-30 OS=Homo sapiens OX=9606 GN=IGHV3-30 PE=1 SV=2
MEFGLSWVFLVALLRGVQCQVQLVESGGGVVQPGRSLRLSCAASGFTFSSYGMHWVRQAP
GKGLEWVAVISYDGSNKYYADSVKGRFTISRDNSKNTLYLQMNSLRAEDTAVYYCAK
>sp|P0DP02|HVC33_HUMAN Immunoglobulin heavy variable 3-30-3 OS=Homo sapiens OX=9606 GN=IGHV3-30-3 PE=1 SV=1
MEFGLSWVFLVALLRGVQCQVQLVESGGGVVQPGRSLRLSCAASGFTFSSYAMHWVRQAP
GKGLEWVAVISYDGSNKYYADSVKGRFTISRDNSKNTLYLQMNSLRAEDTAVYYCAR
>sp|Q13813|SPTN1_HUMAN Spectrin alpha chain, non-erythrocytic 1 OS=Homo sapiens OX=9606 GN=SPTAN1 PE=1 SV=3
MDPSGVKVLETAEDIQERRQQVLDRYHRFKELSTLRRQKLEDSYRFQFFQRDAEELEKWI
QEKLQIASDENYKDPTNLQGKLQKHQAFEAEVQANSGAIVKLDETGNLMISEGHFASETI
RTRLMELHRQWELLLEKMREKGIKLLQAQKLVQYLRECEDVMDWINDKEAIVTSEELGQD
LEHVEVLQKKFEEFQTDMAAHEERVNEVNQFAAKLIQEQHPEEELIKTKQDEVNAAWQRL
KGLALQRQGKLFGAAEVQRFNRDVDETISWIKEKEQLMASDDFGRDLASVQALLRKHEGL
ERDLAALEDKVKALCAEADRLQQSHPLSATQIQVKREELITNWEQIRTLAAERHARLNDS
YRLQRFLADFRDLTSWVTEMKALINADELASDVAGAEALLDRHQEHKGEIDAHEDSFKSA
DESGQALLAAGHYASDEVREKLTVLSEERAALLELWELRRQQYEQCMDLQLFYRDTEQVD
NWMSKQEAFLLNEDLGDSLDSVEALLKKHEDFEKSLSAQEEKITALDEFATKLIQNNHYA
MEDVATRRDALLSRRNALHERAMRRRAQLADSFHLQQFFRDSDELKSWVNEKMKTATDEA
YKDPSNLQGKVQKHQAFEAELSANQSRIDALEKAGQKLIDVNHYAKDEVAARMNEVISLW
KKLLEATELKGIKLREANQQQQFNRNVEDIELWLYEVEGHLASDDYGKDLTNVQNLQKKH
ALLEADVAAHQDRIDGITIQARQFQDAGHFDAENIKKKQEALVARYEALKEPMVARKQKL
ADSLRLQQLFRDVEDEETWIREKEPIAASTNRGKDLIGVQNLLKKHQALQAEIAGHEPRI
KAVTQKGNAMVEEGHFAAEDVKAKLHELNQKWEALKAKASQRRQDLEDSLQAQQYFADAN
EAESWMREKEPIVGSTDYGKDEDSAEALLKKHEALMSDLSAYGSSIQALREQAQSCRQQV
APTDDETGKELVLALYDYQEKSPREVTMKKGDILTLLNSTNKDWWKVEVNDRQGFVPAAY
VKKLDPAQSASRENLLEEQGSIALRQEQIDNQTRITKEAGSVSLRMKQVEELYHSLLELG
EKRKGMLEKSCKKFMLFREANELQQWINEKEAALTSEEVGADLEQVEVLQKKFDDFQKDL
KANESRLKDINKVAEDLESEGLMAEEVQAVQQQEVYGMMPRDETDSKTASPWKSARLMVH
TVATFNSIKELNERWRSLQQLAEERSQLLGSAHEVQRFHRDADETKEWIEEKNQALNTDN
YGHDLASVQALQRKHEGFERDLAALGDKVNSLGETAERLIQSHPESAEDLQEKCTELNQA
WSSLGKRADQRKAKLGDSHDLQRFLSDFRDLMSWINGIRGLVSSDELAKDVTGAEALLER
HQEHRTEIDARAGTFQAFEQFGQQLLAHGHYASPEIKQKLDILDQERADLEKAWVQRRMM
LDQCLELQLFHRDCEQAENWMAAREAFLNTEDKGDSLDSVEALIKKHEDFDKAINVQEEK
IAALQAFADQLIAAGHYAKGDISSRRNEVLDRWRRLKAQMIEKRSKLGESQTLQQFSRDV
DEIEAWISEKLQTASDESYKDPTNIQSKHQKHQAFEAELHANADRIRGVIDMGNSLIERG
ACAGSEDAVKARLAALADQWQFLVQKSAEKSQKLKEANKQQNFNTGIKDFDFWLSEVEAL

LASEDYGKDLASVNNLLKKHQLLEADISAHEDRLKDLNSQADSLMTSSAFDTSQVKDKRD
TINGRFQKIKSMAASRRAKLNESHRLHQFFRDMDDEESWIKEKKLLVGSEDYGRDLTGVQ
NLRKKHKRLEAELAAHEPAIQGVLDTGKKLSDDNTIGKEEIQQRLAQFVEHWKELKQLAA
ARGQRLEESLEYQQFVANVEEEEAWINEKMTLVASEDYGDTLAAIQGLLKKHEAFETDFT
VHKDRVNDVCTNGQDLIKKNNHHEENISSKMKGLNGKVSDLEKAAAQRKAKLDENSAFLQ
FNWKADVVESWIGEKENSLKTDDYGRDLSSVQTLLTKQETFDAGLQAFQQEGIANITALK
DQLLAAKHVQSKAIEARHASLMKRWSQLLANSAARKKKLLEAQSHFRKVEDLFLTFAKKA
SAFNSWFENAEEDLTDPVRCNSLEEIKALREAHDAFRSSLSSAQADFNQLAELDRQIKSF
RVASNPYTWFTMEALEETWRNLQKIIKERELELQKEQRRQEENDKLRQEFAQHANAFHQW
IQETRTYLLDGSCMVEESGTLESQLEATKRKHQEIRAMRSQLKKIEDLGAAMEEALILDN
KYTEHSTVGLAQQWDQLDQLGMRMQHNLEQQIQARNTTGVTEEALKEFSMMFKHFDKDKS
GRLNHQEFKSCLRSLGYDLPMVEEGEPDPEFEAILDTVDPNRDGHVSLQEYMAFMISRET
ENVKSSEEIESAFRALSSEGKPYVTKEELYQNLTREQADYCVSHMKPYVDGKGRELPTAF
DYVEFTRSLFVN
>sp|P62750|RL23A_HUMAN 60S ribosomal protein L23a OS=Homo sapiens OX=9606 GN=RPL23A PE=1 SV=1
MAPKAKKEAPAPPKAEAKAKALKAKKAVLKGVHSHKKKKIRTSPTFRRPKTLRLRRQPKY
PRKSAPRRNKLDHYAIIKFPLTTESAMKKIEDNNTLVFIVDVKANKHQIKQAVKKLYDID
VAKVNTLIRPDGEKKAYVRLAPDYDALDVANKIGII
>sp|Q9H6N6|MYH16_HUMAN Putative uncharacterized protein MYH16 OS=Homo sapiens OX=9606 GN=MYH16 PE=1 SV=2
MGLKVIQQNVHKFLQLRFWGWWKLYNKVKPLLNVARQEEEMKAKEEELRKAMAQTQELVN
KVKELEEKTATLSQEKNDLTIQLQAEQENLMDAEERLTWMMKTKMDLESQISDMRERLEE
EEGMAASLSAAKRKLEGELSDLKRDLEGLETTLAKTEKEKQALDHKVRTLTGDLSLREDS
ITKLQKEKRALEELHQKTLDDLQAEEDKVNHLTKNNSKLSTQIHELEDNWEQEKKIRAEV
EKARRKAESDLKMTIDNLNEMERSKLDLEEVVKKRDLEINSVNSKYEDEQSLNSTLQRKL
KEHQDRIEELEEELEAERAMRAKIEQNRKREAELLKLRRELEEAALQSEATASTLRKKHV
DSMAELTEHVESLQRVKSKLEKDKQVMKAEIDDLNASMETIQKSKMNAEAHVRKLEDSLS
EANAKVAELERNQAEINAIRTRLQAENSELSREYEESQSRLNQILRIKTSLTSQVDDYKR
QLDEESKSRSTAVVSLANTKHDLDLVKEQLEEEQGGKSELQRLVSKLNTEVTTWRTKYET
DAIQRTEELEETKRKLAARLQEAEEAAETAQARAASLEKNKQRLQAEVEDLTIDLEKANA
AAAALDKKQRLFDKMLAEWQQKCEELQVEVDSSQKECRMYMTESFKIKTAYEESLEHLES
VKKENKTLQEEIKDLIDQLGEGGRSVHELQKLKKKLEMEKEELQVALEEAESSLEVEESK
VIRIQLELAQVKADIDRRIHEKEEEFEATRKNHQRAIESLQASLEAEAKGRAEALRLKKK
METDLNEMEIQLDHANKNNSELVKTLKRLQQQIKDLQVQMDEDARQHEELRKQYNLQERR
LSLLQTELEEVRSALEGSERSRKLLEQEVVEITEWHNEINIQNQSLLVVKRKLESDVQRI
SNEHEELISEFRLTEERAKKAMMDAARMAEELRQEQDHCMHLEKIKKNYEVTIKDLQAKM
EEAEQLALKGGKRTIMKLEARIKELETELDGEQKQHVETVKTLCKNERRLKELVFQTEED
HKTNQRMQALVEKLQNKLKVYKRQIEEAEDQANQTLARYRKTVHELDDAEDRAGMAETAL
NKLRTRHRVAGKGITSV
>sp|Q9Y5Z4|HEBP2_HUMAN Heme-binding protein 2 OS=Homo sapiens OX=9606 GN=HEBP2 PE=1 SV=1
MAEPLQPDPGAAEDAAAQAVETPGWKAPEDAGPQPGSYEIRHYGPAKWVSTSVESMDWDS
AIQTGFTKLNSYIQGKNEKEMKIKMTAPVTSYVEPGSGPFSESTITISLYIPSEQQFDPP
RPLESDVFIEDRAEMTVFVRSFDGFSSAQKNQEQLLTLASILREDGKVFDEKVYYTAGYN
SPVKLLNRNNEVWLIQKNEPTKENE

>sp|P08247|SYPH_HUMAN Synaptophysin OS=Homo sapiens OX=9606 GN=SYP PE=1 SV=3
MLLLADMDVVNQLVAGGQFRVVKEPLGFVKVLQWVFAIFAFATCGSYSGELQLSVDCANK
TESDLSIEVEFEYPFRLHQVYFDAPTCRGGTTKVFLVGDYSSSAEFFVTVAVFAFLYSMG
ALATYIFLQNKYRENNKGPMLDFLATAVFAFMWLVSSSAWAKGLSDVKMATDPENIIKEM
PVCRQTGNTCKELRDPVTSGLNTSVVFGFLNLVLWVGNLWFVFKETGWAAPFLRAPPGAP
EKQPAPGDAYGDAGYGQGPGGYGPQDSYGPQGGYQPDYGQPAGSGGSGYGPQGDYGQQGY
GPQGAPTSFSNQM
>sp|O95782|AP2A1_HUMAN AP-2 complex subunit alpha-1 OS=Homo sapiens OX=9606 GN=AP2A1 PE=1 SV=3
MPAVSKGDGMRGLAVFISDIRNCKSKEAEIKRINKELANIRSKFKGDKALDGYSKKKYVC
KLLFIFLLGHDIDFGHMEAVNLLSSNKYTEKQIGYLFISVLVNSNSELIRLINNAIKNDL
ASRNPTFMCLALHCIANVGSREMGEAFAADIPRILVAGDSMDSVKQSAALCLLRLYKASP
DLVPMGEWTARVVHLLNDQHMGVVTAAVSLITCLCKKNPDDFKTCVSLAVSRLSRIVSSA
STDLQDYTYYFVPAPWLSVKLLRLLQCYPPPEDAAVKGRLVECLETVLNKAQEPPKSKKV
QHSNAKNAILFETISLIIHYDSEPNLLVRACNQLGQFLQHRETNLRYLALESMCTLASSE
FSHEAVKTHIDTVINALKTERDVSVRQRAADLLYAMCDRSNAKQIVSEMLRYLETADYAI
REEIVLKVAILAEKYAVDYSWYVDTILNLIRIAGDYVSEEVWYRVLQIVTNRDDVQGYAA
KTVFEALQAPACHENMVKVGGYILGEFGNLIAGDPRSSPPVQFSLLHSKFHLCSVATRAL
LLSTYIKFINLFPETKATIQGVLRAGSQLRNADVELQQRAVEYLTLSSVASTDVLATVLE
EMPPFPERESSILAKLKRKKGPGAGSALDDGRRDPSSNDINGGMEPTPSTVSTPSPSADL
LGLRAAPPPAAPPASAGAGNLLVDVFDGPAAQPSLGPTPEEAFLSELEPPAPESPMALLA
DPAPAADPGPEDIGPPIPEADELLNKFVCKNNGVLFENQLLQIGVKSEFRQNLGRMYLFY
GNKTSVQFQNFSPTVVHPGDLQTQLAVQTKRVAAQVDGGAQVQQVLNIECLRDFLTPPLL
SVRFRYGGAPQALTLKLPVTINKFFQPTEMAAQDFFQRWKQLSLPQQEAQKIFKANHPMD
AEVTKAKLLGFGSALLDNVDPNPENFVGAGIIQTKALQVGCLLRLEPNAQAQMYRLTLRT
SKEPVSRHLCELLAQQF
>sp|O94973|AP2A2_HUMAN AP-2 complex subunit alpha-2 OS=Homo sapiens OX=9606 GN=AP2A2 PE=1 SV=2
MPAVSKGDGMRGLAVFISDIRNCKSKEAEIKRINKELANIRSKFKGDKALDGYSKKKYVC
KLLFIFLLGHDIDFGHMEAVNLLSSNRYTEKQIGYLFISVLVNSNSELIRLINNAIKNDL
ASRNPTFMGLALHCIASVGSREMAEAFAGEIPKVLVAGDTMDSVKQSAALCLLRLYRTSP
DLVPMGDWTSRVVHLLNDQHLGVVTAATSLITTLAQKNPEEFKTSVSLAVSRLSRIVTSA
STDLQDYTYYFVPAPWLSVKLLRLLQCYPPPDPAVRGRLTECLETILNKAQEPPKSKKVQ
HSNAKNAVLFEAISLIIHHDSEPNLLVRACNQLGQFLQHRETNLRYLALESMCTLASSEF
SHEAVKTHIETVINALKTERDVSVRQRAVDLLYAMCDRSNAPQIVAEMLSYLETADYSIR
EEIVLKVAILAEKYAVDYTWYVDTILNLIRIAGDYVSEEVWYRVIQIVINRDDVQGYAAK
TVFEALQAPACHENLVKVGGYILGEFGNLIAGDPRSSPLIQFHLLHSKFHLCSVPTRALL
LSTYIKFVNLFPEVKPTIQDVLRSDSQLRNADVELQQRAVEYLRLSTVASTDILATVLEE
MPPFPERESSILAKLKKKKGPSTVTDLEDTKRDRSVDVNGGPEPAPASTSAVSTPSPSAD
LLGLGAAPPAPAGPPPSSGGSGLLVDVFSDSASVVAPLAPGSEDNFARFVCKNNGVLFEN
QLLQIGLKSEFRQNLGRMFIFYGNKTSTQFLNFTPTLICSDDLQPNLNLQTKPVDPTVEG
GAQVQQVVNIECVSDFTEAPVLNIQFRYGGTFQNVSVQLPITLNKFFQPTEMASQDFFQR
WKQLSNPQQEVQNIFKAKHPMDTEVTKAKIIGFGSALLEEVDPNPANFVGAGIIHTKTTQ
IGCLLRLEPNLQAQMYRLTLRTSKEAVSQRLCELLSAQF
>sp|Q16555|DPYL2_HUMAN Dihydropyrimidinase-related protein 2 OS=Homo sapiens OX=9606 GN=DPYSL2 PE=1 SV=1

MSYQGKKNIPRITSDRLLIKGGKIVNDDQSFYADIYMEDGLIKQIGENLIVPGGVKTIEA
HSRMVIPGGIDVHTRFQMPDQGMTSADDFFQGTKAALAGGTTMIIDHVVPEPGTSLLAAF
DQWREWADSKSCCDYSLHVDISEWHKGIQEEMEALVKDHGVNSFLVYMAFKDRFQLTDCQ
IYEVLSVIRDIGAIAQVHAENGDIIAEEQQRILDLGITGPEGHVLSRPEEVEAEAVNRAI
TIANQTNCPLYITKVMSKSSAEVIAQARKKGTVVYGEPITASLGTDGSHYWSKNWAKAAA
FVTSPPLSPDPTTPDFLNSLLSCGDLQVTGSAHCTFNTAQKAVGKDNFTLIPEGTNGTEE
RMSVIWDKAVVTGKMDENQFVAVTSTNAAKVFNLYPRKGRIAVGSDADLVIWDPDSVKTI
SAKTHNSSLEYNIFEGMECRGSPLVVISQGKIVLEDGTLHVTEGSGRYIPRKPFPDFVYK
RIKARSRLAELRGVPRGLYDGPVCEVSVTPKTVTPASSAKTSPAKQQAPPVRNLHQSGFS
LSGAQIDDNIPRRTTQRIVAPPGGRANITSLG
>sp|Q14194|DPYL1_HUMAN Dihydropyrimidinase-related protein 1 OS=Homo sapiens OX=9606 GN=CRMP1 PE=1 SV=1
MSYQGKKSIPHITSDRLLIKGGRIINDDQSLYADVYLEDGLIKQIGENLIVPGGVKTIEA
NGRMVIPGGIDVNTYLQKPSQGMTAADDFFQGTRAALVGGTTMIIDHVVPEPGSSLLTSF
EKWHEAADTKSCCDYSLHVDITSWYDGVREELEVLVQDKGVNSFQVYMAYKDVYQMSDSQ
LYEAFTFLKGLGAVILVHAENGDLIAQEQKRILEMGITGPEGHALSRPEELEAEAVFRAI
TIAGRINCPVYITKVMSKSAADIIALARKKGPLVFGEPIAASLGTDGTHYWSKNWAKAAA
FVTSPPLSPDPTTPDYLTSLLACGDLQVTGSGHCPYSTAQKAVGKDNFTLIPEGVNGIEE
RMTVVWDKAVATGKMDENQFVAVTSTNAAKIFNLYPRKGRIAVGSDADVVIWDPDKLKTI
TAKSHKSAVEYNIFEGMECHGSPLVVISQGKIVFEDGNINVNKGMGRFIPRKAFPEHLYQ
RVKIRNKVFGLQGVSRGMYDGPVYEVPATPKYATPAPSAKSSPSKHQPPPIRNLHQSNFS
LSGAQIDDNNPRRTGHRIVAPPGGRSNITSLG
>sp|P62851|RS25_HUMAN 40S ribosomal protein S25 OS=Homo sapiens OX=9606 GN=RPS25 PE=1 SV=1
MPPKDDKKKKDAGKSAKKDKDPVNKSGGKAKKKKWSKGKVRDKLNNLVLFDKATYDKLCK
EVPNYKLITPAVVSERLKIRGSLARAALQELLSKGLIKLVSKHRAQVIYTRNTKGGDAPA
AGEDA
>sp|Q9H0E2|TOLIP_HUMAN Toll-interacting protein OS=Homo sapiens OX=9606 GN=TOLLIP PE=1 SV=1
MATTVSTQRGPVYIGELPQDFLRITPTQQQRQVQLDAQAAQQLQYGGAVGTVGRLNITVV
QAKLAKNYGMTRMDPYCRLRLGYAVYETPTAHNGAKNPRWNKVIHCTVPPGVDSFYLEIF
DERAFSMDDRIAWTHITIPESLRQGKVEDKWYSLSGRQGDDKEGMINLVMSYALLPAAMV
MPPQPVVLMPTVYQQGVGYVPITGMPAVCSPGMVPVALPPAAVNAQPRCSEEDLKAIQDM
FPNMDQEVIRSVLEAQRGNKDAAINSLLQMGEEP
>sp|P29034|S10A2_HUMAN Protein S100-A2 OS=Homo sapiens OX=9606 GN=S100A2 PE=1 SV=3
MMCSSLEQALAVLVTTFHKYSCQEGDKFKLSKGEMKELLHKELPSFVGEKVDEEGLKKLM
GSLDENSDQQVDFQEYAVFLALITVMCNDFFQGCPDRP
>sp|Q9H4A4|AMPB_HUMAN Aminopeptidase B OS=Homo sapiens OX=9606 GN=RNPEP PE=1 SV=2
MASGEHSPGSGAARRPLHSAQAVDVASASNFRAFELLHLHLDLRAEFGPPGPGAGSRGLS
GTAVLDLRCLEPEGAAELRLDSHPCLEVTAAALRRERPGSEEPPAEPVSFYTQPFSHYGQ
ALCVSFPQPCRAAERLQVLLTYRVGEGPGVCWLAPEQTAGKKKPFVYTQGQAVLNRAFFP
CFDTPAVKYKYSALIEVPDGFTAVMSASTWEKRGPNKFFFQMCQPIPSYLIALAIGDLVS
AEVGPRSRVWAEPCLIDAAKEEYNGVIEEFLATGEKLFGPYVWGRYDLLFMPPSFPFGGM
ENPCLTFVTPCLLAGDRSLADVIIHEISHSWFGNLVTNANWGEFWLNEGFTMYAQRRIST
ILFGAAYTCLEAATGRALLRQHMDITGEENPLNKLRVKIEPGVDPDDTYNETPYEKGFCF

VSYLAHLVGDQDQFDSFLKAYVHEFKFRSILADDFLDFYLEYFPELKKKRVDIIPGFEFD
RWLNTPGWPPYLPDLSPGDSLMKPAEELAQLWAAEELDMKAIEAVAISPWKTYQLVYFLD
KILQKSPLPPGNVKKLGDTYPSISNARNAELRLRWGQIVLKNDHQEDFWKVKEFLHNQGK
QKYTLPLYHAMMGGSEVAQTLAKETFASTASQLHSNVVNYVQQIVAPKGS
>sp|P17980|PRS6A_HUMAN 26S proteasome regulatory subunit 6A OS=Homo sapiens OX=9606 GN=PSMC3 PE=1 SV=3
MNLLPNIESPVTRQEKMATVWDEAEQDGIGEEVLKMSTEEIIQRTRLLDSEIKIMKSEVL
RVTHELQAMKDKIKENSEKIKVNKTLPYLVSNVIELLDVDPNDQEEDGANIDLDSQRKGK
CAVIKTSTRQTYFLPVIGLVDAEKLKPGDLVGVNKDSYLILETLPTEYDSRVKAMEVDER
PTEQYSDIGGLDKQIQELVEAIVLPMNHKEKFENLGIQPPKGVLMYGPPGTGKTLLARAC
AAQTKATFLKLAGPQLVQMFIGDGAKLVRDAFALAKEKAPSIIFIDELDAIGTKRFDSEK
AGDREVQRTMLELLNQLDGFQPNTQVKVIAATNRVDILDPALLRSGRLDRKIEFPMPNEE
ARARIMQIHSRKMNVSPDVNYEELARCTDDFNGAQCKAVCVEAGMIALRRGATELTHEDY
MEGILEVQAKKKANLQYYA
>sp|Q9NZD1|GPC5D_HUMAN G-protein coupled receptor family C group 5 member D OS=Homo sapiens OX=9606 GN=GPRC5D PE=1 SV=1
MYKDCIESTGDYFLLCDAEGPWGIILESLAILGIVVTILLLLAFLFLMRKIQDCSQWNVL
PTQLLFLLSVLGLFGLAFAFIIELNQQTAPVRYFLFGVLFALCFSCLLAHASNLVKLVRG
CVSFSWTTILCIAIGCSLLQIIIATEYVTLIMTRGMMFVNMTPCQLNVDFVVLLVYVLFL
MALTFFVSKATFCGPCENWKQHGRLIFITVLFSIIIWVVWISMLLRGNPQFQRQPQWDDP
VVCIALVTNAWVFLLLYIVPELCILYRSCRQECPLQGNACPVTAYQHSFQVENQELSRAR
DSDGAEEDVALTSYGTPIQPQTVDPTQECFIPQAKLSPQQDAGGV
>sp|Q86VP6|CAND1_HUMAN Cullin-associated NEDD8-dissociated protein 1 OS=Homo sapiens OX=9606 GN=CAND1 PE=1 SV=2
MASASYHISNLLEKMTSSDKDFRFMATNDLMTELQKDSIKLDDDSERKVVKMILKLLEDK
NGEVQNLAVKCLGPLVSKVKEYQVETIVDTLCTNMLSDKEQLRDISSIGLKTVIGELPPA
SSGSALAANVCKKITGRLTSAIAKQEDVSVQLEALDIMADMLSRQGGLLVNFHPSILTCL
LPQLTSPRLAVRKRTIIALGHLVMSCGNIVFVDLIEHLLSELSKNDSMSTTRTYIQCIAA
ISRQAGHRIGEYLEKIIPLVVKFCNVDDDELREYCIQAFESFVRRCPKEVYPHVSTIINI
CLKYLTYDPNYNYDDEDEDENAMDADGGDDDDQGSDDEYSDDDDMSWKVRRAAAKCLDAV
VSTRHEMLPEFYKTVSPALISRFKEREENVKADVFHAYLSLLKQTRPVQSWLCDPDAMEQ
GETPLTMLQSQVPNIVKALHKQMKEKSVKTRQCCFNMLTELVNVLPGALTQHIPVLVPGI
IFSLNDKSSSSNLKIDALSCLYVILCNHSPQVFHPHVQALVPPVVACVGDPFYKITSEAL
LVTQQLVKVIRPLDQPSSFDATPYIKDLFTCTIKRLKAADIDQEVKERAISCMGQIICNL
GDNLGSDLPNTLQIFLERLKNEITRLTTVKALTLIAGSPLKIDLRPVLGEGVPILASFLR
KNQRALKLGTLSALDILIKNYSDSLTAAMIDAVLDELPPLISESDMHVSQMAISFLTTLA
KVYPSSLSKISGSILNELIGLVRSPLLQGGALSAMLDFFQALVVTGTNNLGYMDLLRMLT
GPVYSQSTALTHKQSYYSIAKCVAALTRACPKEGPAVVGQFIQDVKNSRSTDSIRLLALL
SLGEVGHHIDLSGQLELKSVILEAFSSPSEEVKSAASYALGSISVGNLPEYLPFVLQEIT
SQPKRQYLLLHSLKEIISSASVVGLKPYVENIWALLLKHCECAEEGTRNVVAECLGKLTL
IDPETLLPRLKGYLISGSSYARSSVVTAVKFTISDHPQPIDPLLKNCIGDFLKTLEDPDL
NVRRVALVTFNSAAHNKPSLIRDLLDTVLPHLYNETKVRKELIREVEMGPFKHTVDDGLD
IRKAAFECMYTLLDSCLDRLDIFEFLNHVEDGLKDHYDIKMLTFLMLVRLSTLCPSAVLQ
RLDRLVEPLRATCTTKVKANSVKQEFEKQDELKRSAMRAVAALLTIPEAEKSPLMSEFQS
QISSNPELAAIFESIQKDSSSTNLESMDTS
>sp|Q96S97|MYADM_HUMAN Myeloid-associated differentiation marker OS=Homo sapiens OX=9606 GN=MYADM PE=1 SV=2

MPVTVTRTTITTTTTSSSGLGSPMIVGSPRALTQPLGLLRLLQLVSTCVAFSLVASVGAW
TGSMGNWSMFTWCFCFSVTLIILIVELCGLQARFPLSWRNFPITFACYAALFCLSASIIY
PTTYVQFLSHGRSRDHAIAATFFSCIACVAYATEVAWTRARPGEITGYMATVPGLLKVLE
TFVACIIFAFISDPNLYQHQPALEWCVAVYAICFILAAIAILLNLGECTNVLPIPFPSFL
SGLALLSVLLYATALVLWPLYQFDEKYGGQPRRSRDVSCSRSHAYYVCAWDRRLAVAILT
AINLLAYVADLVHSAHLVFVKV
>sp|P04632|CPNS1_HUMAN Calpain small subunit 1 OS=Homo sapiens OX=9606 GN=CAPNS1 PE=1 SV=1
MFLVNSFLKGGGGGGGGGGGLGGGLGNVLGGLISGAGGGGGGGGGGGGGGGGGGGTAMR
ILGGVISAISEAAAQYNPEPPPPRTHYSNIEANESEEVRQFRRLFAQLAGDDMEVSATEL
MNILNKVVTRHPDLKTDGFGIDTCRSMVAVMDSDTTGKLGFEEFKYLWNNIKRWQAIYKQ
FDTDRSGTICSSELPGAFEAAGFHLNEHLYNMIIRRYSDESGNMDFDNFISCLVRLDAMF
RAFKSLDKDGTGQIQVNIQEWLQLTMYS
>sp|Q8TF09|DLRB2_HUMAN Dynein light chain roadblock-type 2 OS=Homo sapiens OX=9606 GN=DYNLRB2 PE=1 SV=1
MAEVEETLKRIQSHKGVIGTMVVNAEGIPIRTTLDNSTTVQYAGLLHHLTMKAKSTVRDI
DPQNDLTFLRIRSKKHEIMVAPDKEYLLIVIQNPCE
>sp|Q9NP97|DLRB1_HUMAN Dynein light chain roadblock-type 1 OS=Homo sapiens OX=9606 GN=DYNLRB1 PE=1 SV=3
MAEVEETLKRLQSQKGVQGIIVVNTEGIPIKSTMDNPTTTQYASLMHSFILKARSTVRDI
DPQNDLTFLRIRSKKNEIMVAPDKDYFLIVIQNPTE
>sp|P52272|HNRPM_HUMAN Heterogeneous nuclear ribonucleoprotein M OS=Homo sapiens OX=9606 GN=HNRNPM PE=1 SV=3
MAAGVEAAAEVAATEIKMEEESGAPGVPSGNGAPGPKGEGERPAQNEKRKEKNIKRGGNR
FEPYANPTKRYRAFITNIPFDVKWQSLKDLVKEKVGEVTYVELLMDAEGKSRGCAVVEFK
MEESMKKAAEVLNKHSLSGRPLKVKEDPDGEHARRAMQKVMATTGGMGMGPGGPGMITIP
PSILNNPNIPNEIIHALQAGRLGSTVFVANLDYKVGWKKLKEVFSMAGVVVRADILEDKD
GKSRGIGTVTFEQSIEAVQAISMFNGQLLFDRPMHVKMDERALPKGDFFPPERPQQLPHG
LGGIGMGLGPGGQPIDANHLNKGIGMGNIGPAGMGMEGIGFGINKMGGMEGPFGGGMENM
GRFGSGMNMGRINEILSNALKRGEIIAKQGGGGGGGSVPGIERMGPGIDRLGGAGMERMG
AGLGHGMDRVGSEIERMGLVMDRMGSVERMGSGIERMGPLGLDHMASSIERMGQTMERIG
SGVERMGAGMGFGLERMAAPIDRVGQTIERMGSGVERMGPAIERMGLSMERMVPAGMGAG
LERMGPVMDRMATGLERMGANNLERMGLERMGANSLERMGLERMGANSLERMGPAMGPAL
GAGIERMGLAMGGGGGASFDRAIEMERGNFGGSFAGSFGGAGGHAPGVARKACQIFVRNL
PFDFTWKMLKDKFNECGHVLYADIKMENGKSKGCGVVKFESPEVAERACRMMNGMKLSGR
EIDVRIDRNA
>sp|P41091|IF2G_HUMAN Eukaryotic translation initiation factor 2 subunit 3 OS=Homo sapiens OX=9606 GN=EIF2S3 PE=1 SV=3
MAGGEAGVTLGQPHLSRQDLTTLDVTKLTPLSHEVISRQATINIGTIGHVAHGKSTVVKA
ISGVHTVRFKNELERNITIKLGYANAKIYKLDDPSCPRPECYRSCGSSTPDEFPTDIPGT
KGNFKLVRHVSFVDCPGHDILMATMLNGAAVMDAALLLIAGNESCPQPQTSEHLAAIEIM
KLKHILILQNKIDLVKESQAKEQYEQILAFVQGTVAEGAPIIPISAQLKYNIEVVCEYIV
KKIPVPPRDFTSEPRLIVIRSFDVNKPGCEVDDLKGGVAGGSILKGVLKVGQEIEVRPGI
VSKDSEGKLMCKPIFSKIVSLFAEHNDLQYAAPGGLIGVGTKIDPTLCRADRMVGQVLGA
VGALPEIFTELEISYFLLRRLLGVRTEGDKKAAKVQKLSKNEVLMVNIGSLSTGGRVSAV
KADLGKIVLTNPVCTEVGEKIALSRRVEKHWRLIGWGQIRRGVTIKPTVDDD

>sp|P61764|STXB1_HUMAN Syntaxin-binding protein 1 OS=Homo sapiens OX=9606 GN=STXBP1 PE=1 SV=1
MAPIGLKAVVGEKIMHDVIKKVKKKGEWKVLVVDQLSMRMLSSCCKMTDIMTEGITIVED
INKRREPLPSLEAVYLITPSEKSVHSLISDFKDPPTAKYRAAHVFFTDSCPDALFNELVK
SRAAKVIKTLTEINIAFLPYESQVYSLDSADSFQSFYSPHKAQMKNPILERLAEQIATLC
ATLKEYPAVRYRGEYKDNALLAQLIQDKLDAYKADDPTMGEGPDKARSQLLILDRGFDPS
SPVLHELTFQAMSYDLLPIENDVYKYETSGIGEARVKEVLLDEDDDLWIALRHKHIAEVS
QEVTRSLKDFSSSKRMNTGEKTTMRDLSQMLKKMPQYQKELSKYSTHLHLAEDCMKHYQG
TVDKLCRVEQDLAMGTDAEGEKIKDPMRAIVPILLDANVSTYDKIRIILLYIFLKNGITE
ENLNKLIQHAQIPPEDSEIITNMAHLGVPIVTDSTLRRRSKPERKERISEQTYQLSRWTP
IIKDIMEDTIEDKLDTKHYPYISTRSSASFSTTAVSARYGHWHKNKAPGEYRSGPRLIIF
ILGGVSLNEMRCAYEVTQANGKWEVLIGSTHILTPQKLLDTLKKLNKTDEEISS
>sp|P62854|RS26_HUMAN 40S ribosomal protein S26 OS=Homo sapiens OX=9606 GN=RPS26 PE=1 SV=3
MTKKRRNNGRAKKGRGHVQPIRCTNCARCVPKDKAIKKFVIRNIVEAAAVRDISEASVFD
AYVLPKLYVKLHYCVSCAIHSKVVRNRSREARKDRTPPPRFRPAGAAPRPPPKPM
>sp|Q5JNZ5|RS26L_HUMAN Putative 40S ribosomal protein S26-like 1 OS=Homo sapiens OX=9606 GN=RPS26P11 PE=5 SV=1
MTKKRRNNSHAKKGRGHVQPIRCTNCVRCVPTDKAIKKFVIRNIVEAAAVRDISEVSVFD
AYVLPKLYVKLHYCVSCAIHSKVVRNRSREACKDRTPPPRFRPAGAAPRPPPKPM
>sp|P31942|HNRH3_HUMAN Heterogeneous nuclear ribonucleoprotein H3 OS=Homo sapiens OX=9606 GN=HNRNPH3 PE=1 SV=2
MDWVMKHNGPNDASDGTVRLRGLPFGCSKEEIVQFFQGLEIVPNGITLTMDYQGRSTGEA
FVQFASKEIAENALGKHKERIGHRYIEIFRSSRSEIKGFYDPPRRLLGQRPGPYDRPIGG
RGGYYGAGRGSMYDRMRRGGDGYDGGYGGFDDYGGYNNYGYGNDGFDDRMRDGRGMGGHG
YGGAGDASSGFHGGHFVHMRGLPFRATENDIANFFSPLNPIRVHIDIGADGRATGEADVE
FVTHEDAVAAMSKDKNNMQHRYIELFLNSTPGGGSGMGGSGMGGYGRDGMDNQGGYGSVG
RMGMGNNYSGGYGTPDGLGGYGRGGGGSGGYYGQGGMSGGGWRGMY
>sp|P29692|EF1D_HUMAN Elongation factor 1-delta OS=Homo sapiens OX=9606 GN=EEF1D PE=1 SV=5
MATNFLAHEKIWFDKFKYDDAERRFYEQMNGPVAGASRQENGASVILRDIARARENIQKS
LAGSSGPGASSGTSGDHGELVVRIASLEVENQSLRGVVQELQQAISKLEARLNVLEKSSP
GHRATAPQTQHVSPMRQVEPPAKKPATPAEDDEDDDIDLFGSDNEEEDKEAAQLREERLR
QYAEKKAKKPALVAKSSILLDVKPWDDETDMAQLEACVRSIQLDGLVWGASKLVPVGYGI
RKLQIQCVVEDDKVGTDLLEEEITKFEEHVQSVDIAAFNKI
>sp|Q3ZCW2|LEGL_HUMAN Galectin-related protein OS=Homo sapiens OX=9606 GN=LGALSL PE=1 SV=2
MAGSVADSDAVVKLDDGHLNNSLSSPVQADVYFPRLIVPFCGHIKGGMRPGKKVLVMGIV
DLNPESFAISLTCGDSEDPPADVAIELKAVFTDRQLLRNSCISGERGEEQSAIPYFPFIP
DQPFRVEILCEHPRFRVFVDGHQLFDFYHRIQTLSAIDTIKINGDLQITKLG
>sp|P53396|ACLY_HUMAN ATP-citrate synthase OS=Homo sapiens OX=9606 GN=ACLY PE=1 SV=3
MSAKAISEQTGKELLYKFICTTSAIQNRFKYARVTPDTDWARLLQDHPWLLSQNLVVKPD
QLIKRRGKLGLVGVNLTLDGVKSWLKPRLGQEATVGKATGFLKNFLIEPFVPHSQAEEFY
VCIYATREGDYVLFHHEGGVDVGDVDAKAQKLLVGVDEKLNPEDIKKHLLVHAPEDKKEI
LASFISGLFNFYEDLYFTYLEINPLVVTKDGVYVLDLAAKVDATADYICKVKWGDIEFPP
PFGREAYPEEAYIADLDAKSGASLKLTLLNPKGRIWTMVAGGGASVVYSDTICDLGGVNE

LANYGEYSGAPSEQQTYDYAKTILSLMTREKHPDGKILIIGGSIANFTNVAATFKGIVRA
IRDYQGPLKEHEVTIFVRRGGPNYQEGLRVMGEVGKTTGIPIHVFGTETHMTAIVGMALG
HRPIPNQPPTAAHTANFLLNASGSTSTPAPSRTASFSESRADEVAPAKKAKPAMPQDSVP
SPRSLQGKSTTLFSRHTKAIVWGMQTRAVQGMLDFDYVCSRDEPSVAAMVYPFTGDHKQK
FYWGHKEILIPVFKNMADAMRKHPEVDVLINFASLRSAYDSTMETMNYAQIRTIAIIAEG
IPEALTRKLIKKADQKGVTIIGPATVGGIKPGCFKIGNTGGMLDNILASKLYRPGSVAYV
SRSGGMSNELNNIISRTTDGVYEGVAIGGDRYPGSTFMDHVLRYQDTPGVKMIVVLGEIG
GTEEYKICRGIKEGRLTKPIVCWCIGTCATMFSSEVQFGHAGACANQASETAVAKNQALK
EAGVFVPRSFDELGEIIQSVYEDLVANGVIVPAQEVPPPTVPMDYSWARELGLIRKPASF
MTSICDERGQELIYAGMPITEVFKEEMGIGGVLGLLWFQKRLPKYSCQFIEMCLMVTADH
GPAVSGAHNTIICARAGKDLVSSLTSGLLTIGDRFGGALDAAAKMFSKAFDSGIIPMEFV
NKMKKEGKLIMGIGHRVKSINNPDMRVQILKDYVRQHFPATPLLDYALEVEKITTSKKPN
LILNVDGLIGVAFVDMLRNCGSFTREEADEYIDIGALNGIFVLGRSMGFIGHYLDQKRLK
QGLYRHPWDDISYVLPEHMSM
>sp|P46976|GLYG_HUMAN Glycogenin-1 OS=Homo sapiens OX=9606 GN=GYG1 PE=1 SV=4
MTDQAFVTLTTNDAYAKGALVLGSSLKQHRTTRRLVVLATPQVSDSMRKVLETVFDEVIM
VDVLDSGDSAHLTLMKRPELGVTLTKLHCWSLTQYSKCVFMDADTLVLANIDDLFDREEL
SAAPDPGWPDCFNSGVFVYQPSVETYNQLLHLASEQGSFDGGDQGILNTFFSSWATTDIR
KHLPFIYNLSSISIYSYLPAFKVFGASAKVVHFLGRVKPWNYTYDPKTKSVKSEAHDPNM
THPEFLILWWNIFTTNVLPLLQQFGLVKDTCSYVNVLSDLVYTLAFSCGFCRKEDVSGAI
SHLSLGEIPAMAQPFVSSEERKERWEQGQADYMGADSFDNIKRKLDTYLQ
>sp|P30838|AL3A1_HUMAN Aldehyde dehydrogenase, dimeric NADP-preferring OS=Homo sapiens OX=9606 GN=ALDH3A1 PE=1 SV=3
MSKISEAVKRARAAFSSGRTRPLQFRIQQLEALQRLIQEQEQELVGALAADLHKNEWNAY
YEEVVYVLEEIEYMIQKLPEWAADEPVEKTPQTQQDELYIHSEPLGVVLVIGTWNYPFNL
TIQPMVGAIAAGNSVVLKPSELSENMASLLATIIPQYLDKDLYPVINGGVPETTELLKER
FDHILYTGSTGVGKIIMTAAAKHLTPVTLELGGKSPCYVDKNCDLDVACRRIAWGKFMNS
GQTCVAPDYILCDPSIQNQIVEKLKKSLKEFYGEDAKKSRDYGRIISARHFQRVMGLIEG
QKVAYGGTGDAATRYIAPTILTDVDPQSPVMQEEIFGPVLPIVCVRSLEEAIQFINQREK
PLALYMFSSNDKVIKKMIAETSSGGVAANDVIVHITLHSLPFGGVGNSGMGSYHGKKSFE
TFSHRRSCLVRPLMNDEGLKVRYPPSPAKMTQH
>sp|P62847|RS24_HUMAN 40S ribosomal protein S24 OS=Homo sapiens OX=9606 GN=RPS24 PE=1 SV=1
MNDTVTIRTRKFMTNRLLQRKQMVIDVLHPGKATVPKTEIREKLAKMYKTTPDVIFVFGF
RTHFGGGKTTGFGMIYDSLDYAKKNEPKHRLARHGLYEKKKTSRKQRKERKNRMKKVRGT
AKANVGAGKKPKE
>sp|P53618|COPB_HUMAN Coatomer subunit beta OS=Homo sapiens OX=9606 GN=COPB1 PE=1 SV=3
MTAAENVCYTLINVPMDSEPPSEISLKNDLEKGDVKSKTEALKKVIIMILNGEKLPGLLM
TIIRFVLPLQDHTIKKLLLVFWEIVPKTTPDGRLLHEMILVCDAYRKDLQHPNEFIRGST
LRFLCKLKEAELLEPLMPAIRACLEHRHSYVRRNAVLAIYTIYRNFEHLIPDAPELIHDF
LVNEKDASCKRNAFMMLIHADQDRALDYLSTCIDQVQTFGDILQLVIVELIYKVCHANPS
ERARFIRCIYNLLQSSSPAVKYEAAGTLVTLSSAPTAIKAAAQCYIDLIIKESDNNVKLI
VLDRLIELKEHPAHERVLQDLVMDILRVLSTPDLEVRKKTLQLALDLVSSRNVEELVIVL
KKEVIKTNNVSEHEDTDKYRQLLVRTLHSCSVRFPDMAANVIPVLMEFLSDNNEAAAADV
LEFVREAIQRFDNLRMLIVEKMLEVFHAIKSVKIYRGALWILGEYCSTKEDIQSVMTEIR

RSLGEIPIVESEIKKEAGELKPEEEITVGPVQKLVTEMGTYATQSALSSSRPTKKEEDRP
PLRGFLLDGDFFVAASLATTLTKIALRYVALVQEKKKQNSFVAEAMLLMATILHLGKSSL
PKKPITDDDVDRISLCLKVLSECSPLMNDIFNKECRQSLSHMLSAKLEEEKLSQKKESEK
RNVTVQPDDPISFMQLTAKNEMNCKEDQFQLSLLAAMGNTQRKEAADPLASKLNKVTQLT
GFSDPVYAEAYVHVNQYDIVLDVLVVNQTSDTLQNCTLELATLGDLKLVEKPSPLTLAPH
DFANIKANVKVASTENGIIFGNIVYDVSGAASDRNCVVLSDIHIDIMDYIQPATCTDAEF
RQMWAEFEWENKVTVNTNMVDLNDYLQHILKSTNMKCLTPEKALSGYCGFMAANLYARSI
FGEDALANVSIEKPIHQGPDAAVTGHIRIRAKSQGMALSLGDKINLSQKKTSI
>sp|O00231|PSD11_HUMAN 26S proteasome non-ATPase regulatory subunit 11 OS=Homo sapiens OX=9606 GN=PSMD11 PE=1 SV=3
MAAAAVVEFQRAQSLLSTDREASIDILHSIVKRDIQENDEEAVQVKEQSILELGSLLAKT
GQAAELGGLLKYVRPFLNSISKAKAARLVRSLLDLFLDMEAATGQEVELCLECIEWAKSE
KRTFLRQALEARLVSLYFDTKRYQEALHLGSQLLRELKKMDDKALLVEVQLLESKTYHAL
SNLPKARAALTSARTTANAIYCPPKLQATLDMQSGIIHAAEEKDWKTAYSYFYEAFEGYD
SIDSPKAITSLKYMLLCKIMLNTPEDVQALVSGKLALRYAGRQTEALKCVAQASKNRSLA
DFEKALTDYRAELRDDPIISTHLAKLYDNLLEQNLIRVIEPFSRVQIEHISSLIKLSKAD
VERKLSQMILDKKFHGILDQGEGVLIIFDEPPVDKTYEAALETIQNMSKVVDSLYNKAKK
LT
>sp|Q8NEX9|DR9C7_HUMAN Short-chain dehydrogenase/reductase family 9C member 7 OS=Homo sapiens OX=9606 GN=SDR9C7 PE=1 SV=1
MAALTDLSFMYRWFKNCNLVGNLSEKYVFITGCDSGFGNLLAKQLVDRGMQVLAACFTEE
GSQKLQRDTSYRLQTTLLDVTKSESIKAAAQWVRDKVGEQGLWALVNNAGVGLPSGPNEW
LTKDDFVKVINVNLVGLIEVTLHMLPMVKRARGRVVNMSSSGGRVAVIGGGYCVSKFGVE
AFSDSIRRELYYFGVKVCIIEPGNYRTAILGKENLESRMRKLWERLPQETRDSYGEDYFR
IYTDKLKNIMQVAEPRVRDVINSMEHAIVSRSPRIRYNPGLDAKLLYIPLAKLPTPVTDF
ILSRYLPRPADSV
>sp|Q15365|PCBP1_HUMAN Poly(rC)-binding protein 1 OS=Homo sapiens OX=9606 GN=PCBP1 PE=1 SV=2
MDAGVTESGLNVTLTIRLLMHGKEVGSIIGKKGESVKRIREESGARINISEGNCPERIIT
LTGPTNAIFKAFAMIIDKLEEDINSSMTNSTAASRPPVTLRLVVPATQCGSLIGKGGCKI
KEIRESTGAQVQVAGDMLPNSTERAITIAGVPQSVTECVKQICLVMLETLSQSPQGRVMT
IPYQPMPASSPVICAGGQDRCSDAAGYPHATHDLEGPPLDAYSIQGQHTISPLDLAKLNQ
VARQQSHFAMMHGGTGFAGIDSSSPEVKGYWASLDASTQTTHELTIPNNLIGCIIGRQGA
NINEIRQMSGAQIKIANPVEGSSGRQVTITGSAASISLAQYLINARLSSEKGMGCS
>sp|Q01813|PFKAP_HUMAN ATP-dependent 6-phosphofructokinase, platelet type OS=Homo sapiens OX=9606 GN=PFKP PE=1 SV=2
MDADDSRAPKGSLRKFLEHLSGAGKAIGVLTSGGDAQGMNAAVRAVVRMGIYVGAKVYFI
YEGYQGMVDGGSNIAEADWESVSSILQVGGTIIGSARCQAFRTREGRLKAACNLLQRGIT
NLCVIGGDGSLTGANLFRKEWSGLLEELARNGQIDKEAVQKYAYLNVVGMVGSIDNDFCG
TDMTIGTDSALHRIIEVVDAIMTTAQSHQRTFVLEVMGRHCGYLALVSALACGADWVFLP
ESPPEEGWEEQMCVKLSENRARKKRLNIIIVAEGAIDTQNKPITSEKIKELVVTQLGYDT
RVTILGHVQRGGTPSAFDRILASRMGVEAVIALLEATPDTPACVVSLNGNHAVRLPLMEC
VQMTQDVQKAMDERRFQDAVRLRGRSFAGNLNTYKRLAIKLPDDQIPKTNCNVAVINVGA
PAAGMNAAVRSAVRVGIADGHRMLAIYDGFDGFAKGQIKEIGWTDVGGWTGQGGSILGTK
RVLPGKYLEEIATQMRTHSINALLIIGGFEAYLGLLELSAAREKHEEFCVPMVMVPATVS
NNVPGSDFSIGADTALNTITDTCDRIKQSASGTKRRVFIIETMGGYCGYLANMGGLAAGA
DAAYIFEEPFDIRDLQSNVEHLTEKMKTTIQRGLVLRNESCSENYTTDFIYQLYSEEGKG

VFDCRKNVLGHMQQGGAPSPFDRNFGTKISARAMEWITAKLKEARGRGKKFTTDDSICVL
GISKRNVIFQPVAELKKQTDFEHRIPKEQWWLKLRPLMKILAKYKASYDVSDSGQLEHVQ
PWSV
>sp|Q9BRX8|PXL2A_HUMAN Peroxiredoxin-like 2A OS=Homo sapiens OX=9606 GN=PRXL2A PE=1 SV=3
MSFLQDPSFFTMGMWSIGAGALGAAALALLLANTDVFLSKPQKAALEYLEDIDLKTLEKE
PRTFKAKELWEKNGAVIMAVRRPGCFLCREEAADLSSLKSMLDQLGVPLYAVVKEHIRTE
VKDFQPYFKGEIFLDEKKKFYGPQRRKMMFMGFIRLGVWYNFFRAWNGGFSGNLEGEGFI
LGGVFVVGSGKQGILLEHREKEFGDKVNLLSVLEAAKMIKPQTLASEKK
>sp|P00367|DHE3_HUMAN Glutamate dehydrogenase 1, mitochondrial OS=Homo sapiens OX=9606 GN=GLUD1 PE=1 SV=2
MYRYLGEALLLSRAGPAALGSASADSAALLGWARGQPAAAPQPGLALAARRHYSEAVADR
EDDPNFFKMVEGFFDRGASIVEDKLVEDLRTRESEEQKRNRVRGILRIIKPCNHVLSLSF
PIRRDDGSWEVIEGYRAQHSQHRTPCKGGIRYSTDVSVDEVKALASLMTYKCAVVDVPFG
GAKAGVKINPKNYTDNELEKITRRFTMELAKKGFIGPGIDVPAPDMSTGEREMSWIADTY
ASTIGHYDINAHACVTGKPISQGGIHGRISATGRGVFHGIENFINEASYMSILGMTPGFG
DKTFVVQGFGNVGLHSMRYLHRFGAKCIAVGESDGSIWNPDGIDPKELEDFKLQHGSILG
FPKAKPYEGSILEADCDILIPAASEKQLTKSNAPRVKAKIIAEGANGPTTPEADKIFLER
NIMVIPDLYLNAGGVTVSYFEWLKNLNHVSYGRLTFKYERDSNYHLLMSVQESLERKFGK
HGGTIPIVPTAEFQDRISGASEKDIVHSGLAYTMERSARQIMRTAMKYNLGLDLRTAAYV
NAIEKVFKVYNEAGVTFT
>sp|P49448|DHE4_HUMAN Glutamate dehydrogenase 2, mitochondrial OS=Homo sapiens OX=9606 GN=GLUD2 PE=1 SV=2
MYRYLAKALLPSRAGPAALGSAANHSAALLGRGRGQPAAASQPGLALAARRHYSELVADR
EDDPNFFKMVEGFFDRGASIVEDKLVKDLRTQESEEQKRNRVRGILRIIKPCNHVLSLSF
PIRRDDGSWEVIEGYRAQHSQHRTPCKGGIRYSTDVSVDEVKALASLMTYKCAVVDVPFG
GAKAGVKINPKNYTENELEKITRRFTMELAKKGFIGPGVDVPAPDMNTGEREMSWIADTY
ASTIGHYDINAHACVTGKPISQGGIHGRISATGRGVFHGIENFINEASYMSILGMTPGFR
DKTFVVQGFGNVGLHSMRYLHRFGAKCIAVGESDGSIWNPDGIDPKELEDFKLQHGSILG
FPKAKPYEGSILEVDCDILIPAATEKQLTKSNAPRVKAKIIAEGANGPTTPEADKIFLER
NILVIPDLYLNAGGVTVSYFEWLKNLNHVSYGRLTFKYERDSNYHLLLSVQESLERKFGK
HGGTIPIVPTAEFQDSISGASEKDIVHSALAYTMERSARQIMHTAMKYNLGLDLRTAAYV
NAIEKVFKVYSEAGVTFT
>sp|Q12965|MYO1E_HUMAN Unconventional myosin-Ie OS=Homo sapiens OX=9606 GN=MYO1E PE=1 SV=2
MGSKGVYQYHWQSHNVKHSGVDDMVLLSKITENSIVENLKKRYMDDYIFTYIGSVLISVN
PFKQMPYFGEKEIEMYQGAAQYENPPHIYALADNMYRNMIIDRENQCVIISGESGAGKTV
AAKYIMSYISRVSGGGTKVQHVKDIILQSNPLLEAFGNAKTVRNNNSSRFGKYFEIQFSP
GGEPDGGKISNFLLEKSRVVMRNPGERSFHIFYQLIEGASAEQKHSLGITSMDYYYYLSL
SGSYKVDDIDDRREFQETLHAMNVIGIFAEEQTLVLQIVAGILHLGNISFKEVGNYAAVE
SEEFLAFPAYLLGINQDRLKEKLTSRQMDSKWGGKSESIHVTLNVEQACYTRDALAKALH
ARVFDFLVDSINKAMEKDHEEYNIGVLDIYGFEIFQKNGFEQFCINFVNEKLQQIFIELT
LKAEQEEYVQEGIRWTPIEYFNNKIVCDLIENKVNPPGIMSILDDVCATMHAVGEGADQT
LLQKLQMQIGSHEHFNSWNQGFIIHHYAGKVSYDMDGFCERNRDVLFMDLIELMQSSELP
FIKSLFPENLQADKKGRPTTAGSKIKKQANDLVSTLMKCTPHYIRCIKPNETKKPRDWEE
SRVKHQVEYLGLKENIRVRRAGYAYRRIFQKFLQRYAILTKATWPSWQGEEKQGVLHLLQ
SVNMDSDQFQLGRSKVFIKAPESLFLLEEMRERKYDGYARVIQKSWRKFVARKKYVQMRE

EASDLLLNKKERRRNSINRNFIGDYIGMEEHPELQQFVGKREKIDFADTVTKYDRRFKGV
KRDLLLTPKCLYLIGREKVKQGPDKGLVKEVLKRKIEIERILSVSLSTMQDDIFILHEQE
YDSLLESVFKTEFLSLLAKRYEEKTQKQLPLKFSNTLELKLKKENWGPWSAGGSRQVQFH
QGFGDLAVLKPSNKVLQVSIGPGLPKNSRPTRRNTTQNTGYSSGTQNANYPVRAAPPPPG
YHQNGVIRNQYVPYPHAPGSQRSNQKSLYTSMARPPLPRQQSTSSDRVSQTPESLDFLKV
PDQGAAGVRRQTTSRPPPAGGRPKPQPKPKPQVPQCKALYAYDAQDTDELSFNANDIIDI
IKEDPSGWWTGRLRGKQGLFPNNYVTKI
>sp|O00160|MYO1F_HUMAN Unconventional myosin-If OS=Homo sapiens OX=9606 GN=MYO1F PE=1 SV=3
MGSKERFHWQSHNVKQSGVDDMVLLPQITEDAIAANLRKRFMDDYIFTYIGSVLISVNPF
KQMPYFTDREIDLYQGAAQYENPPHIYALTDNMYRNMLIDCENQCVIISGESGAGKTVAA
KYIMGYISKVSGGGEKVQHVKDIILQSNPLLEAFGNAKTVRNNNSSRFGKYFEIQFSRGG
EPDGGKISNFLLEKSRVVMQNENERNFHIYYQLLEGASQEQRQNLGLMTPDYYYYLNQSD
TYQVDGTDDRSDFGETLSAMQVIGIPPSIQQLVLQLVAGILHLGNISFCEDGNYARVESV
DLLAFPAYLLGIDSGRLQEKLTSRKMDSRWGGRSESINVTLNVEQAAYTRDALAKGLYAR
LFDFLVEAINRAMQKPQEEYSIGVLDIYGFEIFQKNGFEQFCINFVNEKLQQIFIELTLK
AEQEEYVQEGIRWTPIQYFNNKVVCDLIENKLSPPGIMSVLDDVCATMHATGGGADQTLL
QKLQAAVGTHEHFNSWSAGFVIHHYAGKVSYDVSGFCERNRDVLFSDLIELMQTSEQAFL
RMLFPEKLDGDKKGRPSTAGSKIKKQANDLVATLMRCTPHYIRCIKPNETKRPRDWEENR
VKHQVEYLGLKENIRVRRAGFAYRRQFAKFLQRYAILTPETWPRWRGDERQGVQHLLRAV
NMEPDQYQMGSTKVFVKNPESLFLLEEVRERKFDGFARTIQKAWRRHVAVRKYEEMREEA
SNILLNKKERRRNSINRNFVGDYLGLEERPELRQFLGKRERVDFADSVTKYDRRFKPIKR
DLILTPKCVYVIGREKVKKGPEKGQVCEVLKKKVDIQALRGVSLSTRQDDFFILQEDAAD
SFLESVFKTEFVSLLCKRFEEATRRPLPLTFSDTLQFRVKKEGWGGGGTRSVTFSRGFGD
LAVLKVGGRTLTVSVGDGLPKSSKPTRKGMAKGKPRRSSQAPTRAAPAPPRGMDRNGVPP
SARGGPLPLEIMSGGGTHRPPRGPPSTSLGASRRPRARPPSEHNTEFLNVPDQGMAGMQR
KRSVGQRPVPGVGRPKPQPRTHGPRCRALYQYVGQDVDELSFNVNEVIEILMEDPSGWWK
GRLHGQEGLFPGNYVEKI
>sp|P0DUB6|AMY1A_HUMAN Alpha-amylase 1A OS=Homo sapiens OX=9606 GN=AMY1A PE=1 SV=1
MKLFWLLFTIGFCWAQYSSNTQQGRTSIVHLFEWRWVDIALECERYLAPKGFGGVQVSPP
NENVAIHNPFRPWWERYQPVSYKLCTRSGNEDEFRNMVTRCNNVGVRIYVDAVINHMCGN
AVSAGTSSTCGSYFNPGSRDFPAVPYSGWDFNDGKCKTGSGDIENYNDATQVRDCRLSGL
LDLALGKDYVRSKIAEYMNHLIDIGVAGFRIDASKHMWPGDIKAILDKLHNLNSNWFPEG
SKPFIYQEVIDLGGEPIKSSDYFGNGRVTEFKYGAKLGTVIRKWNGEKMSYLKNWGEGWG
FMPSDRALVFVDNHDNQRGHGAGGASILTFWDARLYKMAVGFMLAHPYGFTRVMSSYRWP
RYFENGKDVNDWVGPPNDNGVTKEVTINPDTTCGNDWVCEHRWRQIRNMVNFRNVVDGQP
FTNWYDNGSNQVAFGRGNRGFIVFNNDDWTFSLTLQTGLPAGTYCDVISGDKINGNCTGI
KIYVSDDGKAHFSISNSAEDPFIAIHAESKL
>sp|P0DTE7|AMY1B_HUMAN Alpha-amylase 1B OS=Homo sapiens OX=9606 GN=AMY1B PE=1 SV=1
MKLFWLLFTIGFCWAQYSSNTQQGRTSIVHLFEWRWVDIALECERYLAPKGFGGVQVSPP
NENVAIHNPFRPWWERYQPVSYKLCTRSGNEDEFRNMVTRCNNVGVRIYVDAVINHMCGN
AVSAGTSSTCGSYFNPGSRDFPAVPYSGWDFNDGKCKTGSGDIENYNDATQVRDCRLSGL
LDLALGKDYVRSKIAEYMNHLIDIGVAGFRIDASKHMWPGDIKAILDKLHNLNSNWFPEG
SKPFIYQEVIDLGGEPIKSSDYFGNGRVTEFKYGAKLGTVIRKWNGEKMSYLKNWGEGWG
FMPSDRALVFVDNHDNQRGHGAGGASILTFWDARLYKMAVGFMLAHPYGFTRVMSSYRWP

RYFENGKDVNDWVGPPNDNGVTKEVTINPDTTCGNDWVCEHRWRQIRNMVNFRNVVDGQP
FTNWYDNGSNQVAFGRGNRGFIVFNNDDWTFSLTLQTGLPAGTYCDVISGDKINGNCTGI
KIYVSDDGKAHFSISNSAEDPFIAIHAESKL
>sp|P0DTE8|AMY1C_HUMAN Alpha-amylase 1C OS=Homo sapiens OX=9606 GN=AMY1C PE=1 SV=1
MKLFWLLFTIGFCWAQYSSNTQQGRTSIVHLFEWRWVDIALECERYLAPKGFGGVQVSPP
NENVAIHNPFRPWWERYQPVSYKLCTRSGNEDEFRNMVTRCNNVGVRIYVDAVINHMCGN
AVSAGTSSTCGSYFNPGSRDFPAVPYSGWDFNDGKCKTGSGDIENYNDATQVRDCRLSGL
LDLALGKDYVRSKIAEYMNHLIDIGVAGFRIDASKHMWPGDIKAILDKLHNLNSNWFPEG
SKPFIYQEVIDLGGEPIKSSDYFGNGRVTEFKYGAKLGTVIRKWNGEKMSYLKNWGEGWG
FMPSDRALVFVDNHDNQRGHGAGGASILTFWDARLYKMAVGFMLAHPYGFTRVMSSYRWP
RYFENGKDVNDWVGPPNDNGVTKEVTINPDTTCGNDWVCEHRWRQIRNMVNFRNVVDGQP
FTNWYDNGSNQVAFGRGNRGFIVFNNDDWTFSLTLQTGLPAGTYCDVISGDKINGNCTGI
KIYVSDDGKAHFSISNSAEDPFIAIHAESKL
>sp|O15254|ACOX3_HUMAN Peroxisomal acyl-coenzyme A oxidase 3 OS=Homo sapiens OX=9606 GN=ACOX3 PE=1 SV=2
MASTVEGGDTALLPEFPRGPLDAYRARASFSWKELALFTEGEGMLRFKKTIFSALENDPL
FARSPGADLSLEKYRELNFLRCKRIFEYDFLSVEDMFKSPLKVPALIQCLGMYDSSLAAK
YLLHSLVFGSAVYSSGSERHLTYIQKIFRMEIFGCFALTELSHGSNTKAIRTTAHYDPAT
EEFIIHSPDFEAAKFWVGNMGKTATHAVVFAKLCVPGDQCHGLHPFIVQIRDPKTLLPMP
GVMVGDIGKKLGQNGLDNGFAMFHKVRVPRQSLLNRMGDVTPEGTYVSPFKDVRQRFGAS
LGSLSSGRVSIVSLAILNLKLAVAIALRFSATRRQFGPTEEEEIPVLEYPMQQWRLLPYL
AAVYALDHFSKSLFLDLVELQRGLASGDRSARQAELGREIHALASASKPLASWTTQQGIQ
ECREACGGHGYLAMNRLGVLRDDNDPNCTYEGDNNILLQQTSNYLLGLLAHQVHDGACFR
SPLKSVDFLDAYPGILDQKFEVSSVADCLDSAVALAAYKWLVCYLLRETYQKLNQEKRSG
SSDFEARNKCQVSHGRPLALAFVELTVVQRFHEHVHQPSVPPSLRAVLGRLSALYALWSL
SRHAALLYRGGYFSGEQAGEVLESAVLALCSQLKDDAVALVDVIAPPDFVLDSPIGRADG
ELYKNLWGAVLQESKVLERASWWPEFSVNKPVIGSLKSKL
>sp|Q9UBG3|CRNN_HUMAN Cornulin OS=Homo sapiens OX=9606 GN=CRNN PE=1 SV=1
MPQLLQNINGIIEAFRRYARTEGNCTALTRGELKRLLEQEFADVIVKPHDPATVDEVLRL
LDEDHTGTVEFKEFLVLVFKVAQACFKTLSESAEGACGSQESGSLHSGASQELGEGQRSG
TEVGRAGKGQHYEGSSHRQSQQGSRGQNRPGVQTQGQATGSAWVSSYDRQAESQSQERIS
PQIQLSGQTEQTQKAGEGKRNQTTEMRPERQPQTREQDRAHQTGETVTGSGTQTQAGATQ
TVEQDSSHQTGRTSKQTQEATNDQNRGTETHGQGRSQTSQAVTGGHAQIQAGTHTQTPTQ
TVEQDSSHQTGSTSTQTQESTNGQNRGTEIHGQGRSQTSQAVTGGHTQIQAGSHTETVEQ
DRSQTVSHGGAREQGQTQTQPGSGQRWMQVSNPEAGETVPGGQAQTGASTESGRQEWSST
HPRRCVTEGQGDRQPTVVGEEWVDDHSRETVILRLDQGNLHTSVSSAQGQDAAQSEEKRG
ITARELYSYLRSTKP
>sp|Q13515|BFSP2_HUMAN Phakinin OS=Homo sapiens OX=9606 GN=BFSP2 PE=1 SV=1
MSERRVVVDLPTSASSSMPLQRRRASFRGPRSSSSLESPPASRTNAMSGLVRAPGVYVGT
APSGCIGGLGARVTRRALGISSVFLQGLRSSGLATVPAPGLERDHGAVEDLGGCLVEYMA
KVHALEQVSQELETQLRMHLESKATRSGNWGALRASWASSCQQVGEAVLENARLMLQTET
IQAGADDFKERYENEQPFRKAAEEEINSLYKVIDEANLTKMDLESQIESLKEELGSLSRN
YEEDVKLLHKQLAGCELEQMDAPIGTGLDDILETIRIQWERDVEKNRVEAGALLQAKQQA
EVAHMSQTQEEKLAAALRVELHNTSCQVQSLQAETESLRALKRGLENTLHDAKHWHDMEL

QNLGAVVGRLEAELREIRAEAEQQQQERAHLLARKCQLQKDVASYHALLDREESG
>sp|P05023|AT1A1_HUMAN Sodium/potassium-transporting ATPase subunit alpha-1 OS=Homo sapiens OX=9606 GN=ATP1A1 PE=1 SV=1
MGKGVGRDKYEPAAVSEQGDKKGKKGKKDRDMDELKKEVSMDDHKLSLDELHRKYGTDLS
RGLTSARAAEILARDGPNALTPPPTTPEWIKFCRQLFGGFSMLLWIGAILCFLAYSIQAA
TEEEPQNDNLYLGVVLSAVVIITGCFSYYQEAKSSKIMESFKNMVPQQALVIRNGEKMSI
NAEEVVVGDLVEVKGGDRIPADLRIISANGCKVDNSSLTGESEPQTRSPDFTNENPLETR
NIAFFSTNCVEGTARGIVVYTGDRTVMGRIATLASGLEGGQTPIAAEIEHFIHIITGVAV
FLGVSFFILSLILEYTWLEAVIFLIGIIVANVPEGLLATVTVCLTLTAKRMARKNCLVKN
LEAVETLGSTSTICSDKTGTLTQNRMTVAHMWFDNQIHEADTTENQSGVSFDKTSATWLA
LSRIAGLCNRAVFQANQENLPILKRAVAGDASESALLKCIELCCGSVKEMRERYAKIVEI
PFNSTNKYQLSIHKNPNTSEPQHLLVMKGAPERILDRCSSILLHGKEQPLDEELKDAFQN
AYLELGGLGERVLGFCHLFLPDEQFPEGFQFDTDDVNFPIDNLCFVGLISMIDPPRAAVP
DAVGKCRSAGIKVIMVTGDHPITAKAIAKGVGIISEGNETVEDIAARLNIPVSQVNPRDA
KACVVHGSDLKDMTSEQLDDILKYHTEIVFARTSPQQKLIIVEGCQRQGAIVAVTGDGVN
DSPALKKADIGVAMGIAGSDVSKQAADMILLDDNFASIVTGVEEGRLIFDNLKKSIAYTL
TSNIPEITPFLIFIIANIPLPLGTVTILCIDLGTDMVPAISLAYEQAESDIMKRQPRNPK
TDKLVNERLISMAYGQIGMIQALGGFFTYFVILAENGFLPIHLLGLRVDWDDRWINDVED
SYGQQWTYEQRKIVEFTCHTAFFVSIVVVQWADLVICKTRRNSVFQQGMKNKILIFGLFE
ETALAAFLSYCPGMGVALRMYPLKPTWWFCAFPYSLLIFVYDEVRKLIIRRRPGGWVEKE
TYY
>sp|P27797|CALR_HUMAN Calreticulin OS=Homo sapiens OX=9606 GN=CALR PE=1 SV=1
MLLSVPLLLGLLGLAVAEPAVYFKEQFLDGDGWTSRWIESKHKSDFGKFVLSSGKFYGDE
EKDKGLQTSQDARFYALSASFEPFSNKGQTLVVQFTVKHEQNIDCGGGYVKLFPNSLDQT
DMHGDSEYNIMFGPDICGPGTKKVHVIFNYKGKNVLINKDIRCKDDEFTHLYTLIVRPDN
TYEVKIDNSQVESGSLEDDWDFLPPKKIKDPDASKPEDWDERAKIDDPTDSKPEDWDKPE
HIPDPDAKKPEDWDEEMDGEWEPPVIQNPEYKGEWKPRQIDNPDYKGTWIHPEIDNPEYS
PDPSIYAYDNFGVLGLDLWQVKSGTIFDNFLITNDEAYAEEFGNETWGVTKAAEKQMKDK
QDEEQRLKEEEEDKKRKEEEEAEDKEDDEDKDEDEEDEEDKEEDEEEDVPGQAKDEL
>sp|Q9Y4I1|MYO5A_HUMAN Unconventional myosin-Va OS=Homo sapiens OX=9606 GN=MYO5A PE=1 SV=2
MAASELYTKFARVWIPDPEEVWKSAELLKDYKPGDKVLLLHLEEGKDLEYHLDPKTKELP
HLRNPDILVGENDLTALSYLHEPAVLHNLRVRFIDSKLIYTYCGIVLVAINPYEQLPIYG
EDIINAYSGQNMGDMDPHIFAVAEEAYKQMARDERNQSIIVSGESGAGKTVSAKYAMRYF
ATVSGSASEANVEEKVLASNPIMESIGNAKTTRNDNSSRFGKYIEIGFDKRYRIIGANMR
TYLLEKSRVVFQAEEERNYHIFYQLCASAKLPEFKMLRLGNADNFNYTKQGGSPVIEGVD
DAKEMAHTRQACTLLGISESHQMGIFRILAGILHLGNVGFTSRDADSCTIPPKHEPLCIF
CELMGVDYEEMCHWLCHRKLATATETYIKPISKLQATNARDALAKHIYAKLFNWIVDNVN
QALHSAVKQHSFIGVLDIYGFETFEINSFEQFCINYANEKLQQQFNMHVFKLEQEEYMKE
QIPWTLIDFYDNQPCINLIESKLGILDLLDEECKMPKGTDDTWAQKLYNTHLNKCALFEK
PRLSNKAFIIQHFADKVEYQCEGFLEKNKDTVFEEQIKVLKSSKFKMLPELFQDDEKAIS
PTSATSSGRTPLTRTPAKPTKGRPGQMAKEHKKTVGHQFRNSLHLLMETLNATTPHYVRC
IKPNDFKFPFTFDEKRAVQQLRACGVLETIRISAAGFPSRWTYQEFFSRYRVLMKQKDVL
SDRKQTCKNVLEKLILDKDKYQFGKTKIFFRAGQVAYLEKLRADKLRAACIRIQKTIRGW
LLRKKYLRMRKAAITMQRYVRGYQARCYAKFLRRTKAATIIQKYWRMYVVRRRYKIRRAA
TIVLQSYLRGFLARNRYRKILREHKAVIIQKRVRGWLARTHYKRSMHAIIYLQCCFRRMM

AKRELKKLKIEARSVERYKKLHIGMENKIMQLQRKVDEQNKDYKCLVEKLTNLEGIYNSE
TEKLRSDLERLQLSEEEAKVATGRVLSLQEEIAKLRKDLEQTRSEKKCIEEHADRYKQET
EQLVSNLKEENTLLKQEKEALNHRIVQQAKEMTETMEKKLVEETKQLELDLNDERLRYQN
LLNEFSRLEERYDDLKEEMTLMVHVPKPGHKRTDSTHSSNESEYIFSSEIAEMEDIPSRT
EEPSEKKVPLDMSLFLKLQKRVTELEQEKQVMQDELDRKEEQVLRSKAKEEERPQIRGAE
LEYESLKRQELESENKKLKNELNELRKALSEKSAPEVTAPGAPAYRVLMEQLTSVSEELD
VRKEEVLILRSQLVSQKEAIQPKDDKNTMTDSTILLEDVQKMKDKGEIAQAYIGLKETNR
SSALDYHELNEDGELWLVYEGLKQANRLLESQLQSQKRSHENEAEALRGEIQSLKEENNR
QQQLLAQNLQLPPEARIEASLQHEITRLTNENLDLMEQLEKQDKTVRKLKKQLKVFAKKI
GELEVGQMENISPGQIIDEPIRPVNIPRKEKDFQGMLEYKKEDEQKLVKNLILELKPRGV
AVNLIPGLPAYILFMCVRHADYLNDDQKVRSLLTSTINSIKKVLKKRGDDFETVSFWLSN
TCRFLHCLKQYSGEEGFMKHNTSRQNEHCLTNFDLAEYRQVLSDLAIQIYQQLVRVLENI
LQPMIVSGMLEHETIQGVSGVKPTGLRKRTSSIADEGTYTLDSILRQLNSFHSVMCQHGM
DPELIKQVVKQMFYIIGAITLNNLLLRKDMCSWSKGMQIRYNVSQLEEWLRDKNLMNSGA
KETLEPLIQAAQLLQVKKKTDDDAEAICSMCNALTTAQIVKVLNLYTPVNEFEERVSVSF
IRTIQMRLRDRKDSPQLLMDAKHIFPVTFPFNPSSLALETIQIPASLGLGFISRV
>sp|P14625|ENPL_HUMAN Endoplasmin OS=Homo sapiens OX=9606 GN=HSP90B1 PE=1 SV=1
MRALWVLGLCCVLLTFGSVRADDEVDVDGTVEEDLGKSREGSRTDDEVVQREEEAIQLDG
LNASQIRELREKSEKFAFQAEVNRMMKLIINSLYKNKEIFLRELISNASDALDKIRLISL
TDENALSGNEELTVKIKCDKEKNLLHVTDTGVGMTREELVKNLGTIAKSGTSEFLNKMTE
AQEDGQSTSELIGQFGVGFYSAFLVADKVIVTSKHNNDTQHIWESDSNEFSVIADPRGNT
LGRGTTITLVLKEEASDYLELDTIKNLVKKYSQFINFPIYVWSSKTETVEEPMEEEEAAK
EEKEESDDEAAVEEEEEEKKPKTKKVEKTVWDWELMNDIKPIWQRPSKEVEEDEYKAFYK
SFSKESDDPMAYIHFTAEGEVTFKSILFVPTSAPRGLFDEYGSKKSDYIKLYVRRVFITD
DFHDMMPKYLNFVKGVVDSDDLPLNVSRETLQQHKLLKVIRKKLVRKTLDMIKKIADDKY
NDTFWKEFGTNIKLGVIEDHSNRTRLAKLLRFQSSHHPTDITSLDQYVERMKEKQDKIYF
MAGSSRKEAESSPFVERLLKKGYEVIYLTEPVDEYCIQALPEFDGKRFQNVAKEGVKFDE
SEKTKESREAVEKEFEPLLNWMKDKALKDKIEKAVVSQRLTESPCALVASQYGWSGNMER
IMKAQAYQTGKDISTNYYASQKKTFEINPRHPLIRDMLRRIKEDEDDKTVLDLAVVLFET
ATLRSGYLLPDTKAYGDRIERMLRLSLNIDPDAKVEEEPEEEPEETAEDTTEDTEQDEDE
EMDVGTDEEEETAKESTAEKDEL
>sp|Q01082|SPTB2_HUMAN Spectrin beta chain, non-erythrocytic 1 OS=Homo sapiens OX=9606 GN=SPTBN1 PE=1 SV=2
MTTTVATDYDNIEIQQQYSDVNNRWDVDDWDNENSSARLFERSRIKALADEREAVQKKTF
TKWVNSHLARVSCRITDLYTDLRDGRMLIKLLEVLSGERLPKPTKGRMRIHCLENVDKAL
QFLKEQRVHLENMGSHDIVDGNHRLTLGLIWTIILRFQIQDISVETEDNKEKKSAKDALL
LWCQMKTAGYPNVNIHNFTTSWRDGMAFNALIHKHRPDLIDFDKLKKSNAHYNLQNAFNL
AEQHLGLTKLLDPEDISVDHPDEKSIITYVVTYYHYFSKMKALAVEGKRIGKVLDNAIET
EKMIEKYESLASDLLEWIEQTIIILNNRKFANSLVGVQQQLQAFNTYRTVEKPPKFTEKG
NLEVLLFTIQSKMRANNQKVYMPREGKLISDINKAWERLEKAEHERELALRNELIRQEKL
EQLARRFDRKAAMRETWLSENQRLVSQDNFGFDLPAVEAATKKHEAIETDIAAYEERVQA
VVAVARELEAENYHDIKRITARKDNVIRLWEYLLELLRARRQRLEMNLGLQKIFQEMLYI
MDWMDEMKVLVLSQDYGKHLLGVEDLLQKHTLVEADIGIQAERVRGVNASAQKFATDGEG
YKPCDPQVIRDRVAHMEFCYQELCQLAAERRARLEESRRLWKFFWEMAEEEGWIREKEKI
LSSDDYGKDLTSVMRLLSKHRAFEDEMSGRSGHFEQAIKEGEDMIAEEHFGSEKIRERII
YIREQWANLEQLSAIRKKRLEEASLLHQFQADADDIDAWMLDILKIVSSSDVGHDEYSTQ

SLVKKHKDVAEEIANYRPTLDTLHEQASALPQEHAESPDVRGRLSGIEERYKEVAELTRL
RKQALQDTLALYKMFSEADACELWIDEKEQWLNNMQIPEKLEDLEVIQHRFESLEPEMNN
QASRVAVVNQIARQLMHSGHPSEKEIKAQQDKLNTRWSQFRELVDRKKDALLSALSIQNY
HLECNETKSWIREKTKVIESTQDLGNDLAGVMALQRKLTGMERDLVAIEAKLSDLQKEAE
KLESEHPDQAQAILSRLAEISDVWEEMKTTLKNREASLGEASKLQQFLRDLDDFQSWLSR
TQTAIASEDMPNTLTEAEKLLTQHENIKNEIDNYEEDYQKMRDMGEMVTQGQTDAQYMFL
RQRLQALDTGWNELHKMWENRQNLLSQSHAYQQFLRDTKQAEAFLNNQEYVLAHTEMPTT
LEGAEAAIKKQEDFMTTMDANEEKINAVVETGRRLVSDGNINSDRIQEKVDSIDDRHRKN
RETASELLMRLKDNRDLQKFLQDCQELSLWINEKMLTAQDMSYDEARNLHSKWLKHQAFM
AELASNKEWLDKIEKEGMQLISEKPETEAVVKEKLTGLHKMWEVLESTTQTKAQRLFDAN
KAELFTQSCADLDKWLHGLESQIQSDDYGKDLTSVNILLKKQQMLENQMEVRKKEIEELQ
SQAQALSQEGKSTDEVDSKRLTVQTKFMELLEPLNERKHNLLASKEIHQFNRDVEDEILW
VGERMPLATSTDHGHNLQTVQLLIKKNQTLQKEIQGHQPRIDDIFERSQNIVTDSSSLSA
EAIRQRLADLKQLWGLLIEETEKRHRRLEEAHRAQQYYFDAAEAEAWMSEQELYMMSEEK
AKDEQSAVSMLKKHQILEQAVEDYAETVHQLSKTSRALVADSHPESERISMRQSKVDKLY
AGLKDLAEERRGKLDERHRLFQLNREVDDLEQWIAEREVVAGSHELGQDYEHVTMLQERF
REFARDTGNIGQERVDTVNHLADELINSGHSDAATIAEWKDGLNEAWADLLELIDTRTQI
LAASYELHKFYHDAKEIFGRIQDKHKKLPEELGRDQNTVETLQRMHTTFEHDIQALGTQV
RQLQEDAARLQAAYAGDKADDIQKRENEVLEAWKSLLDACESRRVRLVDTGDKFRFFSMV
RDLMLWMEDVIRQIEAQEKPRDVSSVELLMNNHQGIKAEIDARNDSFTTCIELGKSLLAR
KHYASEEIKEKLLQLTEKRKEMIDKWEDRWEWLRLILEVHQFSRDASVAEAWLLGQEPYL
SSREIGQSVDEVEKLIKRHEAFEKSAATWDERFSALERLTTLELLEVRRQQEEEERKRRP
PSPEPSTKVSEEAESQQQWDTSKGEQVSQNGLPAEQGSPRMAETVDTSEMVNGATEQRTS
SKESSPIPSPTSDRKAKTALPAQSAATLPARTQETPSAQMEGFLNRKHEWEAHNKKASSR
SWHNVYCVINNQEMGFYKDAKTAASGIPYHSEVPVSLKEAVCEVALDYKKKKHVFKLRLN
DGNEYLFQAKDDEEMNTWIQAISSAISSDKHEVSASTQSTPASSRAQTLPTSVVTITSES
SPGKREKDKEKDKEKRFSLFGKKK
>sp|Q6P1J6|PLB1_HUMAN Phospholipase B1, membrane-associated OS=Homo sapiens OX=9606 GN=PLB1 PE=1 SV=3
MGLRPGIFLLELLLLLGQGTPQIHTSPRKSTLEGQLWPETLKNSPFPCNPNKLGVNMPSK
SVHSLKPSDIKFVAAIGNLEIPPDPGTGDLEKQDWTERPQQVCMGVMTVLSDIIRYFSPS
VPMPVCHTGKRVIPHDGAEDLWIQAQELVRNMKENLQLDFQFDWKLINVFFSNASQCYLC
PSAQQNGLAAGGVDELMGVLDYLQQEVPRAFVNLVDLSEVAEVSRQYHGTWLSPAPEPCN
CSEETTRLAKVVMQWSYQEAWNSLLASSRYSEQESFTVVFQPFFYETTPSLHSEDPRLQD
STTLAWHLWNRMMEPAGEKDEPLSVKHGRPMKCPSQESPYLFSYRNSNYLTRLQKPQDKL
EVREGAEIRCPDKDPSDTVPTSVHRLKPADINVIGALGDSLTAGNGAGSTPGNVLDVLTQ
YRGLSWSVGGDENIGTVTTLANILREFNPSLKGFSVGTGKETSPNAFLNQAVAGGRAEDL
PVQARRLVDLMKNDTRIHFQEDWKIITLFIGGNDLCDFCNDLVHYSPQNFTDNIGKALDI
LHAEVPRAFVNLVTVLEIVNLRELYQEKKVYCPRMILRSLCPCVLKFDDNSTELATLIEF
NKKFQEKTHQLIESGRYDTREDFTVVVQPFFENVDMPKTSEGLPDNSFFAPDCFHFSSKS
HSRAASALWNNMLEPVGQKTTRHKFENKINITCPNQVQPFLRTYKNSMQGHGTWLPCRDR
APSALHPTSVHALRPADIQVVAALGDSLTAGNGIGSKPDDLPDVTTQYRGLSYSAGGDGS
LENVTTLPNILREFNRNLTGYAVGTGDANDTNAFLNQAVPGAKAEDLMSQVQTLMQKMKD
DHRVNFHEDWKVITVLIGGSDLCDYCTDSNLYSAANFVHHLRNALDVLHREVPRVLVNLV
DFLNPTIMRQVFLGNPDKCPVQQASVLCNCVLTLRENSQELARLEAFSRAYRSSMRELVG
SGRYDTQEDFSVVLQPFFQNIQLPVLADGLPDTSFFAPDCIHPNQKFHSQLARALWTNML
EPLGSKTETLDLRAEMPITCPTQNEPFLRTPRNSNYTYPIKPAIENWGSDFLCTEWKASN

SVPTSVHQLRPADIKVVAALGDSLTTAVGARPNNSSDLPTSWRGLSWSIGGDGNLETHTT
LPNILKKFNPYLLGFSTSTWEGTAGLNVAAEGARARDMPAQAWDLVERMKNSPDINLEKD
WKLVTLFIGVNDLCHYCENPEAHLATEYVQHIQQALDILSEELPRAFVNVVEVMELASLY
QGQGGKCAMLAAQNNCTCLRHSQSSLEKQELKKVNWNLQHGISSFSYWHQYTQREDFAVV
VQPFFQNTLTPLNERGDTDLTFFSEDCFHFSDRGHAEMAIALWNNMLEPVGRKTTSNNFT
HSRAKLKCPSPESPYLYTLRNSRLLPDQAEEAPEVLYWAVPVAAGVGLVVGIIGTVVWRC
RRGGRREDPPMSLRTVAL
>sp|Q9BYP9|KRA99_HUMAN Keratin-associated protein 9-9 OS=Homo sapiens OX=9606 GN=KRTAP9-9 PE=2 SV=1
MTHCCSPCCQPTCCRTTCCRTTCWKPTTVTTCSSTPCCQPSCCVSSCCQPCCRPACCQNT
CCRTTCCQPTCLSSCCGQTSCGSSCGQSSSCAPVYCRRTCYYPTTVCLPGCLNQSCGSSC
CQPCCRPACCETTCCRTTCFQPTCVSSCCQPSCC
>sp|Q9BYQ4|KRA92_HUMAN Keratin-associated protein 9-2 OS=Homo sapiens OX=9606 GN=KRTAP9-2 PE=1 SV=2
MTHCCSPCCQPTCCRTTCCRTTCWKPTTVTTCSSTPCCQPACCVSSCCQPCCRPTCCQNT
CCRTTCCQPTCVTSCCQPSCCSTPCCQPTCCGSSCCGQTSCGSSCGQSSSCAPVYCRRTC
YYPTTVCLPGCLNQSCGSNCCQPCCRPACCETTCCRTTCFQPTCVSSCCQPSCC
>sp|Q9BYQ0|KRA98_HUMAN Keratin-associated protein 9-8 OS=Homo sapiens OX=9606 GN=KRTAP9-8 PE=1 SV=2
MTHCCSPCCQPTCCRTTCWKPTTVTTCSSTPCCQPSCCVSSCCQPCCRPTCCQNTCCQPI
CVTSCCQPSCCSTPCCQPTCCGQTSCGSSCGQSSSCAPVYCRRTCYHPTTVCLPGCLNQS
CGSNCCQPCCRPACCETTCCRTTCFQPTCVSSCCQPSCC
>sp|A8MTY7|KRA97_HUMAN Keratin-associated protein 9-7 OS=Homo sapiens OX=9606 GN=KRTAP9-7 PE=3 SV=1
MTHCCSPCCQPTCCRTTCWKPTTVTTCSSTPCCQPSCCVSSCCQPCCHPTCCQNTCCRTT
CCQPTCVTSCCQPSCCSTPCCQPICCGSSCCGQTSCGSSCCQPSSCAPIYCRRTCYHPTS
VYLPGCLNQSCGSSCCQPCCRPACCETTCCRTTCFQPTCVTSCCQPACC
>sp|Q9BYQ3|KRA93_HUMAN Keratin-associated protein 9-3 OS=Homo sapiens OX=9606 GN=KRTAP9-3 PE=1 SV=1
MTHCCSPCCQPTCCRTTCWQPTTVTTCSSTPCCQPSCCVSSCCQPCCHPTCCQNTCCRTT
CCQPICVTSCCQPSCCSTPCCQPTCCGSSCGQSSSCAPVYCRRTCYHPTSVCLPGCLNQS
CGSNCCQPCCRPACCETTCCRTTCFQPTCVYSCCQPSCC
>sp|A8MXZ3|KRA91_HUMAN Keratin-associated protein 9-1 OS=Homo sapiens OX=9606 GN=KRTAP9-1 PE=3 SV=1
MTHCCSPCCQPTCCRTTCCRTTCWKPTTVTTCSSTPCCQPSCCVPSCCQPCCHPTCCQNT
CCRTTCCQPTCVASCCQPSCCSTPCCQPTCCGSSCCGQTSCGSSCCQPICGSSCCQPCCH
PTCYQTICFRTTCCQPTCCQPTCCRNTSCQPTCCGSSCCQPCCHPTCCQTICRSTCCQPS
CVTRCCSTPCCQPTCGGSSCCSQTCNESSYCLPCCRPTCCQTTCYRTTCCRPSCCCSPCC
VSSCCQPSCC
>sp|Q9BYQ2|KRA94_HUMAN Keratin-associated protein 9-4 OS=Homo sapiens OX=9606 GN=KRTAP9-4 PE=1 SV=2
MTHCCSPCCQPTCCRTTCCRTTCWKPTTVTTCSSTPCCQPSCCVSSCCQPCCRPTCCQNT
CCQPTCVTSCCQPSCCSTPCCQPTCCGSSCDQSSSCAPVYCRRTCYYPTTVCLPGCLNQS
CGSNCCQPCCRPACCETTCFQPTCVSSCCQPFCC
>sp|Q8N442|GUF1_HUMAN Translation factor GUF1, mitochondrial OS=Homo sapiens OX=9606 GN=GUF1 PE=1 SV=1
MWTLVGRGWGCARALAPRATGAALLVAPGPRSAPTLGAAPESWATDRLYSSAEFKEKLDM

SRFPVENIRNFSIVAHVDHGKSTLADRLLELTGTIDKTKNNKQVLDKLQVERERGITVKA
QTASLFYNCEGKQYLLNLIDTPGHVDFSYEVSRSLSACQGVLLVVDANEGIQAQTVANFF
LAFEAQLSVIPVINKIDLKNADPERVENQIEKVFDIPSDECIKISAKLGTNVESVLQAII
ERIPPPKVHRKNPLRALVFDSTFDQYRGVIANVALFDGVVSKGDKIVSAHTQKTYEVNEV
GVLNPNEQPTHKLYAGQVGYLIAGMKDVTEAQIGDTLCLHKQPVEPLPGFKSAKPMVFAG
MYPLDQSEYNNLKSAIEKLTLNDSSVTVHRDSSLALGAGWRLGFLGLLHMEVFNQRLEQE
YNASVILTTPTVPYKAVLSSSKLIKEHREKEITIINPAQFPDKSKVTEYLEPVVLGTIIT
PDEYTGKIMMLCEARRAVQKNMIFIDQNRVMLKYLFPLNEIVVDFYDSLKSLSSGYASFD
YEDAGYQTAELVKMDILLNGNTVEELVTVVHKDKAHSIGKAICERLKDSLPRQLFEIAIQ
AAIGSKIIARETVKAYRKNVLAKCYGGDITRKMKLLKRQAEGKKKLRKIGNVEVPKDAFI
KVLKTQSSK
>sp|P22234|PUR6_HUMAN Bifunctional phosphoribosylaminoimidazole carboxylase/phosphoribosylaminoimidazole succinocarboxamide synthetase OS=Homo sapiens OX=9606 GN=PAICS PE=1 SV=3
MATAEVLNIGKKLYEGKTKEVYELLDSPGKVLLQSKDQITAGNAARKNHLEGKAAISNKI
TSCIFQLLQEAGIKTAFTRKCGETAFIAPQCEMIPIEWVCRRIATGSFLKRNPGVKEGYK
FYPPKVELFFKDDANNDPQWSEEQLIAAKFCFAGLLIGQTEVDIMSHATQAIFEILEKSW
LPQNCTLVDMKIEFGVDVTTKEIVLADVIDNDSWRLWPSGDRSQQKDKQSYRDLKEVTPE
GLQMVKKNFEWVAERVELLLKSESQCRVVVLMGSTSDLGHCEKIKKACGNFGIPCELRVT
SAHKGPDETLRIKAEYEGDGIPTVFVAVAGRSNGLGPVMSGNTAYPVISCPPLTPDWGVQ
DVWSSLRLPSGLGCSTVLSPEGSAQFAAQIFGLSNHLVWSKLRASILNTWISLKQADKKI
RECNL
>sp|P28072|PSB6_HUMAN Proteasome subunit beta type-6 OS=Homo sapiens OX=9606 GN=PSMB6 PE=1 SV=4
MAATLLAARGAGPAPAWGPEAFTPDWESREVSTGTTIMAVQFDGGVVLGADSRTTTGSYI
ANRVTDKLTPIHDRIFCCRSGSAADTQAVADAVTYQLGFHSIELNEPPLVHTAASLFKEM
CYRYREDLMAGIIIAGWDPQEGGQVYSVPMGGMMVRQSFAIGGSGSSYIYGYVDATYREG
MTKEECLQFTANALALAMERDGSSGGVIRLAAIAESGVERQVLLGDQIPKFAVATLPPA
>sp|P04844|RPN2_HUMAN Dolichyl-diphosphooligosaccharide--protein glycosyltransferase subunit 2 OS=Homo sapiens OX=9606 GN=RPN2 PE=1 SV=3
MAPPGSSTVFLLALTIIASTWALTPTHYLTKHDVERLKASLDRPFTNLESAFYSIVGLSS
LGAQVPDAKKACTYIRSNLDPSNVDSLFYAAQASQALSGCEISISNETKDLLLAAVSEDS
SVTQIYHAVAALSGFGLPLASQEALSALTARLSKEETVLATVQALQTASHLSQQADLRSI
VEEIEDLVARLDELGGVYLQFEEGLETTALFVAATYKLMDHVGTEPSIKEDQVIQLMNAI
FSKKNFESLSEAFSVASAAAVLSHNRYHVPVVVVPEGSASDTHEQAILRLQVTNVLSQPL
TQATVKLEHAKSVASRATVLQKTSFTPVGDVFELNFMNVKFSSGYYDFLVEVEGDNRYIA
NTVELRVKISTEVGITNVDLSTVDKDQSIAPKTTRVTYPAKAKGTFIADSHQNFALFFQL
VDVNTGAELTPHQTFVRLHNQKTGQEVVFVAEPDNKNVYKFELDTSERKIEFDSASGTYT
LYLIIGDATLKNPILWNVADVVIKFPEEEAPSTVLSQNLFTPKQEIQHLFREPEKRPPTV
VSNTFTALILSPLLLLFALWIRIGANVSNFTFAPSTIIFHLGHAAMLGLMYVYWTQLNMF
QTLKYLAILGSVTFLAGNRMLAQQAVKRTAH
>sp|P62266|RS23_HUMAN 40S ribosomal protein S23 OS=Homo sapiens OX=9606 GN=RPS23 PE=1 SV=3
MGKCRGLRTARKLRSHRRDQKWHDKQYKKAHLGTALKANPFGGASHAKGIVLEKVGVEAK
QPNSAIRKCVRVQLIKNGKKITAFVPNDGCLNFIEENDEVLVAGFGRKGHAVGDIPGVRF
KVVKVANVSLLALYKGKKERPRS

>sp|P17900|SAP3_HUMAN Ganglioside GM2 activator OS=Homo sapiens OX=9606 GN=GM2A PE=1 SV=4
MQSLMQAPLLIALGLLLAAPAQAHLKKPSQLSSFSWDNCDEGKDPAVIRSLTLEPDPIIV
PGNVTLSVMGSTSVPLSSPLKVDLVLEKEVAGLWIKIPCTDYIGSCTFEHFCDVLDMLIP
TGEPCPEPLRTYGLPCHCPFKEGTYSLPKSEFVVPDLELPSWLTTGNYRIESVLSSSGKR
LGCIKIAASLKGI
>sp|P63000|RAC1_HUMAN Ras-related C3 botulinum toxin substrate 1 OS=Homo sapiens OX=9606 GN=RAC1 PE=1 SV=1
MQAIKCVVVGDGAVGKTCLLISYTTNAFPGEYIPTVFDNYSANVMVDGKPVNLGLWDTAG
QEDYDRLRPLSYPQTDVFLICFSLVSPASFENVRAKWYPEVRHHCPNTPIILVGTKLDLR
DDKDTIEKLKEKKLTPITYPQGLAMAKEIGAVKYLECSALTQRGLKTVFDEAIRAVLCPP
PVKKRKRKCLLL
>sp|P30408|T4S1_HUMAN Transmembrane 4 L6 family member 1 OS=Homo sapiens OX=9606 GN=TM4SF1 PE=1 SV=1
MCYGKCARCIGHSLVGLALLCIAANILLYFPNGETKYASENHLSRFVWFFSGIVGGGLLM
LLPAFVFIGLEQDDCCGCCGHENCGKRCAMLSSVLAALIGIAGSGYCVIVAALGLAEGPL
CLDSLGQWNYTFASTEGQYLLDTSTWSECTEPKHIVEWNVSLFSILLALGGIEFILCLIQ
VINGVLGGICGFCCSHQQQYDC
>sp|P61077|UB2D3_HUMAN Ubiquitin-conjugating enzyme E2 D3 OS=Homo sapiens OX=9606 GN=UBE2D3 PE=1 SV=1
MALKRINKELSDLARDPPAQCSAGPVGDDMFHWQATIMGPNDSPYQGGVFFLTIHFPTDY
PFKPPKVAFTTRIYHPNINSNGSICLDILRSQWSPALTISKVLLSICSLLCDPNPDDPLV
PEIARIYKTDRDKYNRISREWTQKYAM
>sp|P62837|UB2D2_HUMAN Ubiquitin-conjugating enzyme E2 D2 OS=Homo sapiens OX=9606 GN=UBE2D2 PE=1 SV=1
MALKRIHKELNDLARDPPAQCSAGPVGDDMFHWQATIMGPNDSPYQGGVFFLTIHFPTDY
PFKPPKVAFTTRIYHPNINSNGSICLDILRSQWSPALTISKVLLSICSLLCDPNPDDPLV
PEIARIYKTDREKYNRIAREWTQKYAM
>sp|A8MTJ3|GNAT3_HUMAN Guanine nucleotide-binding protein G(t) subunit alpha-3 OS=Homo sapiens OX=9606 GN=GNAT3 PE=2 SV=2
MGSGISSESKESAKRSKELEKKLQEDAERDARTVKLLLLGAGESGKSTIVKQMKIIHKNG
YSEQECMEFKAVIYSNTLQSILAIVKAMTTLGIDYVNPRSAEDQRQLYAMANTLEDGGMT
PQLAEVIKRLWRDPGIQACFERASEYQLNDSAAYYLNDLDRITASGYVPNEQDVLHSRVK
TTGIIETQFSFKDLHFRMFDVGGQRSERKKWIHCFEGVTCIIFCAALSAYDMVLVEDEEV
NRMHESLHLFNSICNHKYFSTTSIVLFLNKKDIFQEKVTKVHLSICFPEYTGPNTFEDAG
NYIKNQFLDLNLKKEDKEIYSHMTCATDTQNVKFVFDAVTDIIIKENLKDCGLF
>sp|P19087|GNAT2_HUMAN Guanine nucleotide-binding protein G(t) subunit alpha-2 OS=Homo sapiens OX=9606 GN=GNAT2 PE=1 SV=4
MGSGASAEDKELAKRSKELEKKLQEDADKEAKTVKLLLLGAGESGKSTIVKQMKIIHQDG
YSPEECLEFKAIIYGNVLQSILAIIRAMTTLGIDYAEPSCADDGRQLNNLADSIEEGTMP
PELVEVIRRLWKDGGVQACFERAAEYQLNDSASYYLNQLERITDPEYLPSEQDVLRSRVK
TTGIIETKFSVKDLNFRMFDVGGQRSERKKWIHCFEGVTCIIFCAALSAYDMVLVEDDEV
NRMHESLHLFNSICNHKFFAATSIVLFLNKKDLFEEKIKKVHLSICFPEYDGNNSYDDAG
NYIKSQFLDLNMRKDVKEIYSHMTCATDTQNVKFVFDAVTDIIIKENLKDCGLF
>sp|P11488|GNAT1_HUMAN Guanine nucleotide-binding protein G(t) subunit alpha-1 OS=Homo sapiens OX=9606 GN=GNAT1 PE=1 SV=5
MGAGASAEEKHSRELEKKLKEDAEKDARTVKLLLLGAGESGKSTIVKQMKIIHQDGYSLE

ECLEFIAIIYGNTLQSILAIVRAMTTLNIQYGDSARQDDARKLMHMADTIEEGTMPKEMS
DIIQRLWKDSGIQACFERASEYQLNDSAGYYLSDLERLVTPGYVPTEQDVLRSRVKTTGI
IETQFSFKDLNFRMFDVGGQRSERKKWIHCFEGVTCIIFIAALSAYDMVLVEDDEVNRMH
ESLHLFNSICNHRYFATTSIVLFLNKKDVFFEKIKKAHLSICFPDYDGPNTYEDAGNYIK
VQFLELNMRRDVKEIYSHMTCATDTQNVKFVFDAVTDIIIKENLKDCGLF
>sp|P09471|GNAO_HUMAN Guanine nucleotide-binding protein G(o) subunit alpha OS=Homo sapiens OX=9606 GN=GNAO1 PE=1 SV=4
MGCTLSAEERAALERSKAIEKNLKEDGISAAKDVKLLLLGAGESGKSTIVKQMKIIHEDG
FSGEDVKQYKPVVYSNTIQSLAAIVRAMDTLGIEYGDKERKADAKMVCDVVSRMEDTEPF
SAELLSAMMRLWGDSGIQECFNRSREYQLNDSAKYYLDSLDRIGAADYQPTEQDILRTRV
KTTGIVETHFTFKNLHFRLFDVGGQRSERKKWIHCFEDVTAIIFCVALSGYDQVLHEDET
TNRMHESLMLFDSICNNKFFIDTSIILFLNKKDLFGEKIKKSPLTICFPEYTGPNTYEDA
AAYIQAQFESKNRSPNKEIYCHMTCATDTNNIQVVFDAVTDIIIANNLRGCGLY
>sp|Q5JWF2|GNAS1_HUMAN Guanine nucleotide-binding protein G(s) subunit alpha isoforms XLas OS=Homo sapiens OX=9606 GN=GNAS PE=1 SV=2
MGVRNCLYGNNMSGQRDIPPEIGEQPEQPPLEAPGAAAPGAGPSPAEEMETEPPHNEPIP
VENDGEACGPPEVSRPNFQVLNPAFREAGAHGSYSPPPEEAMPFEAEQPSLGGFWPTLEQ
PGFPSGVHAGLEAFGPALMEPGAFSGARPGLGGYSPPPEEAMPFEFDQPAQRGCSQLLLQ
VPDLAPGGPGAAGVPGAPPEEPQALRPAKAGSRGGYSPPPEETMPFELDGEGFGDDSPPP
GLSRVIAQVDGSSQFAAVAASSAVRLTPAANAPPLWVPGAIGSPSQEAVRPPSNFTGSSP
WMEISGPPFEIGSAPAGVDDTPVNMDSPPIALDGPPIKVSGAPDKRERAERPPVEEEAAE
MEGAADAAEGGKVPSPGYGSPAAGAASADTAARAAPAAPADPDSGATPEDPDSGTAPADP
DSGAFAADPDSGAAPAAPADPDSGAAPDAPADPDSGAAPDAPADPDAGAAPEAPAAPAAA
ETRAAHVAPAAPDAGAPTAPAASATRAAQVRRAASAAPASGARRKIHLRPPSPEIQAADP
PTPRPTRASAWRGKSESSRGRRVYYDEGVASSDDDSSGDESDDGTSGCLRWFQHRRNRRR
RKPQRNLLRNFLVQAFGGCFGRSESPQPKASRSLKVKKVPLAEKRRQMRKEALEKRAQKR
AEKKRSKLIDKQLQDEKMGYMCTHRLLLLGAGESGKSTIVKQMRILHVNGFNGEGGEEDP
QAARSNSDGEKATKVQDIKNNLKEAIETIVAAMSNLVPPVELANPENQFRVDYILSVMNV
PDFDFPPEFYEHAKALWEDEGVRACYERSNEYQLIDCAQYFLDKIDVIKQADYVPSDQDL
LRCRVLTSGIFETKFQVDKVNFHMFDVGGQRDERRKWIQCFNDVTAIIFVVASSSYNMVI
REDNQTNRLQEALNLFKSIWNNRWLRTISVILFLNKQDLLAEKVLAGKSKIEDYFPEFAR
YTTPEDATPEPGEDPRVTRAKYFIRDEFLRISTASGDGRHYCYPHFTCAVDTENIRRVFN
DCRDIIQRMHLRQYELL
>sp|P63092|GNAS2_HUMAN Guanine nucleotide-binding protein G(s) subunit alpha isoforms short OS=Homo sapiens OX=9606 GN=GNAS PE=1 SV=1
MGCLGNSKTEDQRNEEKAQREANKKIEKQLQKDKQVYRATHRLLLLGAGESGKSTIVKQM
RILHVNGFNGEGGEEDPQAARSNSDGEKATKVQDIKNNLKEAIETIVAAMSNLVPPVELA
NPENQFRVDYILSVMNVPDFDFPPEFYEHAKALWEDEGVRACYERSNEYQLIDCAQYFLD
KIDVIKQADYVPSDQDLLRCRVLTSGIFETKFQVDKVNFHMFDVGGQRDERRKWIQCFND
VTAIIFVVASSSYNMVIREDNQTNRLQEALNLFKSIWNNRWLRTISVILFLNKQDLLAEK
VLAGKSKIEDYFPEFARYTTPEDATPEPGEDPRVTRAKYFIRDEFLRISTASGDGRHYCY
PHFTCAVDTENIRRVFNDCRDIIQRMHLRQYELL
>sp|P38405|GNAL_HUMAN Guanine nucleotide-binding protein G(olf) subunit alpha OS=Homo sapiens OX=9606 GN=GNAL PE=1 SV=1
MGCLGGNSKTTEDQGVDEKERREANKKIEKQLQKERLAYKATHRLLLLGAGESGKSTIVK

QMRILHVNGFNPEEKKQKILDIRKNVKDAIVTIVSAMSTIIPPVPLANPENQFRSDYIKS
IAPITDFEYSQEFFDHVKKLWDDEGVKACFERSNEYQLIDCAQYFLERIDSVSLVDYTPT
DQDLLRCRVLTSGIFETRFQVDKVNFHMFDVGGQRDERRKWIQCFNDVTAIIYVAACSSY
NMVIREDNNTNRLRESLDLFESIWNNRWLRTISIILFLNKQDMLAEKVLAGKSKIEDYFP
EYANYTVPEDATPDAGEDPKVTRAKFFIRDLFLRISTATGDGKHYCYPHFTCAVDTENIR
RVFNDCRDIIQRMHLKQYELL
>sp|P04899|GNAI2_HUMAN Guanine nucleotide-binding protein G(i) subunit alpha-2 OS=Homo sapiens OX=9606 GN=GNAI2 PE=1 SV=3
MGCTVSAEDKAAAERSKMIDKNLREDGEKAAREVKLLLLGAGESGKSTIVKQMKIIHEDG
YSEEECRQYRAVVYSNTIQSIMAIVKAMGNLQIDFADPSRADDARQLFALSCTAEEQGVL
PDDLSGVIRRLWADHGVQACFGRSREYQLNDSAAYYLNDLERIAQSDYIPTQQDVLRTRV
KTTGIVETHFTFKDLHFKMFDVGGQRSERKKWIHCFEGVTAIIFCVALSAYDLVLAEDEE
MNRMHESMKLFDSICNNKWFTDTSIILFLNKKDLFEEKITHSPLTICFPEYTGANKYDEA
ASYIQSKFEDLNKRKDTKEIYTHFTCATDTKNVQFVFDAVTDVIIKNNLKDCGLF
>sp|P63096|GNAI1_HUMAN Guanine nucleotide-binding protein G(i) subunit alpha-1 OS=Homo sapiens OX=9606 GN=GNAI1 PE=1 SV=2
MGCTLSAEDKAAVERSKMIDRNLREDGEKAAREVKLLLLGAGESGKSTIVKQMKIIHEAG
YSEEECKQYKAVVYSNTIQSIIAIIRAMGRLKIDFGDSARADDARQLFVLAGAAEEGFMT
AELAGVIKRLWKDSGVQACFNRSREYQLNDSAAYYLNDLDRIAQPNYIPTQQDVLRTRVK
TTGIVETHFTFKDLHFKMFDVGGQRSERKKWIHCFEGVTAIIFCVALSDYDLVLAEDEEM
NRMHESMKLFDSICNNKWFTDTSIILFLNKKDLFEEKIKKSPLTICYPEYAGSNTYEEAA
AYIQCQFEDLNKRKDTKEIYTHFTCATDTKNVQFVFDAVTDVIIKNNLKDCGLF
>sp|P08754|GNAI3_HUMAN Guanine nucleotide-binding protein G(i) subunit alpha-3 OS=Homo sapiens OX=9606 GN=GNAI3 PE=1 SV=3
MGCTLSAEDKAAVERSKMIDRNLREDGEKAAKEVKLLLLGAGESGKSTIVKQMKIIHEDG
YSEDECKQYKVVVYSNTIQSIIAIIRAMGRLKIDFGEAARADDARQLFVLAGSAEEGVMT
PELAGVIKRLWRDGGVQACFSRSREYQLNDSASYYLNDLDRISQSNYIPTQQDVLRTRVK
TTGIVETHFTFKDLYFKMFDVGGQRSERKKWIHCFEGVTAIIFCVALSDYDLVLAEDEEM
NRMHESMKLFDSICNNKWFTETSIILFLNKKDLFEEKIKRSPLTICYPEYTGSNTYEEAA
AYIQCQFEDLNRRKDTKEIYTHFTCATDTKNVQFVFDAVTDVIIKNNLKECGLY
>sp|Q03113|GNA12_HUMAN Guanine nucleotide-binding protein subunit alpha-12 OS=Homo sapiens OX=9606 GN=GNA12 PE=1 SV=4
MSGVVRTLSRCLLPAEAGGARERRAGSGARDAEREARRRSRDIDALLARERRAVRRLVKI
LLLGAGESGKSTFLKQMRIIHGREFDQKALLEFRDTIFDNILKGSRVLVDARDKLGIPWQ
YSENEKHGMFLMAFENKAGLPVEPATFQLYVPALSALWRDSGIREAFSRRSEFQLGESVK
YFLDNLDRIGQLNYFPSKQDILLARKATKGIVEHDFVIKKIPFKMVDVGGQRSQRQKWFQ
CFDGITSILFMVSSSEYDQVLMEDRRTNRLVESMNIFETIVNNKLFFNVSIILFLNKMDL
LVEKVKTVSIKKHFPDFRGDPHRLEDVQRYLVQCFDRKRRNRSKPLFHHFTTAIDTENVR
FVFHAVKDTILQENLKDIMLQ
>sp|Q14344|GNA13_HUMAN Guanine nucleotide-binding protein subunit alpha-13 OS=Homo sapiens OX=9606 GN=GNA13 PE=1 SV=2
MADFLPSRSVLSVCFPGCLLTSGEAEQQRKSKEIDKCLSREKTYVKRLVKILLLGAGESG
KSTFLKQMRIIHGQDFDQRAREEFRPTIYSNVIKGMRVLVDAREKLHIPWGDNSNQQHGD
KMMSFDTRAPMAAQGMVETRVFLQYLPAIRALWADSGIQNAYDRRREFQLGESVKYFLDN
LDKLGEPDYIPSQQDILLARRPTKGIHEYDFEIKNVPFKMVDVGGQRSERKRWFECFDSV
TSILFLVSSSEFDQVLMEDRLTNRLTESLNIFETIVNNRVFSNVSIILFLNKTDLLEEKV
QIVSIKDYFLEFEGDPHCLRDVQKFLVECFRNKRRDQQQKPLYHHFTTAINTENIRLVFR

DVKDTILHDNLKQLMLQ
>sp|P48444|COPD_HUMAN Coatomer subunit delta OS=Homo sapiens OX=9606 GN=ARCN1 PE=1 SV=1
MVLLAAAVCTKAGKAIVSRQFVEMTRTRIEGLLAAFPKLMNTGKQHTFVETESVRYVYQP
MEKLYMVLITTKNSNILEDLETLRLFSRVIPEYCRALEENEISEHCFDLIFAFDEIVALG
YRENVNLAQIRTFTEMDSHEEKVFRAVRETQEREAKAEMRRKAKELQQARRDAERQGKKA
PGFGGFGSSAVSGGSTAAMITETIIETDKPKVAPAPARPSGPSKALKLGAKGKEVDNFVD
KLKSEGETIMSSSMGKRTSEATKMHAPPINMESVHMKIEEKITLTCGRDGGLQNMELHGM
IMLRISDDKYGRIRLHVENEDKKGVQLQTHPNVDKKLFTAESLIGLKNPEKSFPVNSDVG
VLKWRLQTTEESFIPLTINCWPSESGNGCDVNIEYELQEDNLELNDVVITIPLPSGVGAP
VIGEIDGEYRHDSRRNTLEWCLPVIDAKNKSGSLEFSIAGQPNDFFPVQVSFVSKKNYCN
IQVTKVTQVDGNSPVRFSTETTFLVDKYEIL
>sp|P48735|IDHP_HUMAN Isocitrate dehydrogenase [NADP], mitochondrial OS=Homo sapiens OX=9606 GN=IDH2 PE=1 SV=2
MAGYLRVVRSLCRASGSRPAWAPAALTAPTSQEQPRRHYADKRIKVAKPVVEMDGDEMTR
IIWQFIKEKLILPHVDIQLKYFDLGLPNRDQTDDQVTIDSALATQKYSVAVKCATITPDE
ARVEEFKLKKMWKSPNGTIRNILGGTVFREPIICKNIPRLVPGWTKPITIGRHAHGDQYK
ATDFVADRAGTFKMVFTPKDGSGVKEWEVYNFPAGGVGMGMYNTDESISGFAHSCFQYAI
QKKWPLYMSTKNTILKAYDGRFKDIFQEIFDKHYKTDFDKNKIWYEHRLIDDMVAQVLKS
SGGFVWACKNYDGDVQSDILAQGFGSLGLMTSVLVCPDGKTIEAEAAHGTVTRHYREHQK
GRPTSTNPIASIFAWTRGLEHRGKLDGNQDLIRFAQMLEKVCVETVESGAMTKDLAGCIH
GLSNVKLNEHFLNTTDFLDTIKSNLDRALGRQ
>sp|Q8NCW5|NNRE_HUMAN NAD(P)H-hydrate epimerase OS=Homo sapiens OX=9606 GN=NAXE PE=1 SV=2
MSRLRALLGLGLLVAGSRVPRIKSQTIACRSGPTWWGPQRLNSGGRWDSEVMASTVVKYL
SQEEAQAVDQELFNEYQFSVDQLMELAGLSCATAIAKAYPPTSMSRSPPTVLVICGPGNN
GGDGLVCARHLKLFGYEPTIYYPKRPNKPLFTALVTQCQKMDIPFLGEMPAEPMTIDELY
ELVVDAIFGFSFKGDVREPFHSILSVLKGLTVPIASIDIPSGWDVEKGNAGGIQPDLLIS
LTAPKKSATQFTGRYHYLGGRFVPPALEKKYQLNLPPYPDTECVYRLQ
>sp|A0A0C4DH29|HV103_HUMAN Immunoglobulin heavy variable 1-3 OS=Homo sapiens OX=9606 GN=IGHV1-3 PE=3 SV=1
MDWTWRILFLVAAATGAHSQVQLVQSGAEVKKPGASVKVSCKASGYTFTSYAMHWVRQAP
GQRLEWMGWINAGNGNTKYSQKFQGRVTITRDTSASTAYMELSSLRSEDTAVYYCAR
>sp|A0A0C4DH33|HV124_HUMAN Immunoglobulin heavy variable 1-24 OS=Homo sapiens OX=9606 GN=IGHV1-24 PE=3 SV=1
MDCTWRILFLVAAATGTHAQVQLVQSGAEVKKPGASVKVSCKVSGYTLTELSMHWVRQAP
GKGLEWMGGFDPEDGETIYAQKFQGRVTMTEDTSTDTAYMELSSLRSEDTAVYYCAT
>sp|P23083|HV102_HUMAN Immunoglobulin heavy variable 1-2 OS=Homo sapiens OX=9606 GN=IGHV1-2 PE=1 SV=2
MDWTWRILFLVAAATGAHSQVQLVQSGAEVKKPGASVKVSCKASGYTFTGYYMHWVRQAP
GQGLEWMGWINPNSGGTNYAQKFQGWVTMTRDTSISTAYMELSRLRSDDTAVYYCAR
>sp|A0A0B4J2H0|HV69D_HUMAN Immunoglobulin heavy variable 1-69D OS=Homo sapiens OX=9606 GN=IGHV1-69D PE=1 SV=1
MDWTWRFLFVVAAATGVQSQVQLVQSGAEVKKPGSSVKVSCKASGGTFSSYAISWVRQAP
GQGLEWMGGIIPIFGTANYAQKFQGRVTITADESTSTAYMELSSLRSEDTAVYYCAR
>sp|P01742|HV169_HUMAN Immunoglobulin heavy variable 1-69 OS=Homo sapiens OX=9606 GN=IGHV1-69 PE=1 SV=2

MDWTWRFLFVVAAATGVQSQVQLVQSGAEVKKPGSSVKVSCKASGGTFSSYAISWVRQAP
GQGLEWMGGIIPIFGTANYAQKFQGRVTITADKSTSTAYMELSSLRSEDTAVYYCAR
>sp|P01743|HV146_HUMAN Immunoglobulin heavy variable 1-46 OS=Homo sapiens OX=9606 GN=IGHV1-46 PE=1 SV=2
MDWTWRVFCLLAVAPGAHSQVQLVQSGAEVKKPGASVKVSCKASGYTFTSYYMHWVRQAP
GQGLEWMGIINPSGGSTSYAQKFQGRVTMTRDTSTSTVYMELSSLRSEDTAVYYCAR
>sp|A0A0C4DH31|HV118_HUMAN Immunoglobulin heavy variable 1-18 OS=Homo sapiens OX=9606 GN=IGHV1-18 PE=3 SV=1
MDWTWSILFLVAAATGAHSQVQLVQSGAEVKKPGASVKVSCKASGYTFTSYGISWVRQAP
GQGLEWMGWISAYNGNTNYAQKLQGRVTMTTDTSTSTAYMELRSLRSDDTAVYYCAR
>sp|P0DP01|HV108_HUMAN Immunoglobulin heavy variable 1-8 OS=Homo sapiens OX=9606 GN=IGHV1-8 PE=1 SV=1
MDWTWRILFLVAAATSAHSQVQLVQSGAEVKKPGASVKVSCKASGYTFTSYDINWVRQAT
GQGLEWMGWMNPNSGNTGYAQKFQGRVTMTRNTSISTAYMELSSLRSEDTAVYYCAR
>sp|O14880|MGST3_HUMAN Microsomal glutathione S-transferase 3 OS=Homo sapiens OX=9606 GN=MGST3 PE=1 SV=1
MAVLSKEYGFVLLTGAASFIMVAHLAINVSKARKKYKVEYPIMYSTDPENGHIFNCIQRA
HQNTLEVYPPFLFFLAVGGVYHPRIASGLGLAWIVGRVLYAYGYYTGEPSKRSRGALGSI
ALLGLVGTTVCSAFQHLGWVKSGLGSGPKCCH
>sp|Q13275|SEM3F_HUMAN Semaphorin-3F OS=Homo sapiens OX=9606 GN=SEMA3F PE=2 SV=2
MLVAGLLLWASLLTGAWPSFPTQDHLPATPRVRLSFKELKATGTAHFFNFLLNTTDYRIL
LKDEDHDRMYVGSKDYVLSLDLHDINREPLIIHWAASPQRIEECVLSGKDVNGECGNFVR
LIQPWNRTHLYVCGTGAYNPMCTYVNRGRRAQATPWTQTQAVRGRGSRATDGALRPMPTA
PRQDYIFYLEPERLESGKGKCPYDPKLDTASALINEELYAGVYIDFMGTDAAIFRTLGKQ
TAMRTDQYNSRWLNDPSFIHAELIPDSAERNDDKLYFFFRERSAEAPQSPAVYARIGRIC
LNDDGGHCCLVNKWSTFLKARLVCSVPGEDGIETHFDELQDVFVQQTQDVRNPVIYAVFT
SSGSVFRGSAVCVYSMADIRMVFNGPFAHKEGPNYQWMPFSGKMPYPRPGTCPGGTFTPS
MKSTKDYPDEVINFMRSHPLMYQAVYPLQRRPLVVRTGAPYRLTTIAVDQVDAADGRYEV
LFLGTDRGTVQKVIVLPKDDQELEELMLEEVEVFKDPAPVKTMTISSKRQQLYVASAVGV
THLSLHRCQAYGAACADCCLARDPYCAWDGQACSRYTASSKRRSRRQDVRHGNPIRQCRG
FNSNANKNAVESVQYGVAGSAAFLECQPRSPQATVKWLFQRDPGDRRREIRAEDRFLRTE
QGLLLRALQLSDRGLYSCTATENNFKHVVTRVQLHVLGRDAVHAALFPPLSMSAPPPPGA
GPPTPPYQELAQLLAQPEVGLIHQYCQGYWRHVPPSPREAPGAPRSPEPQDQKKPRNRRH
HPPDT
>sp|O60763|USO1_HUMAN General vesicular transport factor p115 OS=Homo sapiens OX=9606 GN=USO1 PE=1 SV=2
MNFLRGVMGGQSAGPQHTEAETIQKLCDRVASSTLLDDRRNAVRALKSLSKKYRLEVGIQ
AMEHLIHVLQTDRSDSEIIGYALDTLYNIISNEEEEEVEENSTRQSEDLGSQFTEIFIKQ
QENVTLLLSLLEEFDFHVRWPGVKLLTSLLKQLGPQVQQIILVSPMGVSRLMDLLADSRE
VIRNDGVLLLQALTRSNGAIQKIVAFENAFERLLDIISEEGNSDGGIVVEDCLILLQNLL
KNNNSNQNFFKEGSYIQRMKPWFEVGDENSGWSAQKVTNLHLMLQLVRVLVSPTNPPGAT
SSCQKAMFQCGLLQQLCTILMATGVPADILTETINTVSEVIRGCQVNQDYFASVNAPSNP
PRPAIVVLLMSMVNERQPFVLRCAVLYCFQCFLYKNQKGQGEIVSTLLPSTIDATGNSVS
AGQLLCGGLFSTDSLSNWCAAVALAHALQENATQKEQLLRVQLATSIGNPPVSLLQQCTN
ILSQGSKIQTRVGLLMLLCTWLSNCPIAVTHFLHNSANVPFLTGQIAENLGEEEQLVQGL
CALLLGISIYFNDNSLESYMKEKLKQLIEKRIGKENFIEKLGFISKHELYSRASQKPQPN

FPSPEYMIFDHEFTKLVKELEGVITKAIYKSSEEDKKEEEVKKTLEQHDNIVTHYKNMIR
EQDLQLEELRQQVSTLKCQNEQLQTAVTQQVSQIQQHKDQYNLLKIQLGKDNQHQGSYSE
GAQMNGIQPEEIGRLREEIEELKRNQELLQSQLTEKDSMIENMKSSQTSGTNEQSSAIVS
ARDSEQVAELKQELATLKSQLNSQSVEITKLQTEKQELLQKTEAFAKSVEVQGETETIIA
TKTTDVEGRLSALLQETKELKNEIKALSEERTAIKEQLDSSNSTIAILQTEKDKLELEIT
DSKKEQDDLLVLLADQDQKILSLKNKLKDLGHPVEEEDELESGDQEDEDDESEDPGKDLD
HI
>sp|O00232|PSD12_HUMAN 26S proteasome non-ATPase regulatory subunit 12 OS=Homo sapiens OX=9606 GN=PSMD12 PE=1 SV=3
MADGGSERADGRIVKMEVDYSATVDQRLPECAKLAKEGRLQEVIETLLSLEKQTRTASDM
VSTSRILVAVVKMCYEAKEWDLLNENIMLLSKRRSQLKQAVAKMVQQCCTYVEEITDLPI
KLRLIDTLRMVTEGKIYVEIERARLTKTLATIKEQNGDVKEAASILQELQVETYGSMEKK
ERVEFILEQMRLCLAVKDYIRTQIISKKINTKFFQEENTEKLKLKYYNLMIQLDQHEGSY
LSICKHYRAIYDTPCIQAESEKWQQALKSVVLYVILAPFDNEQSDLVHRISGDKKLEEIP
KYKDLLKLFTTMELMRWSTLVEDYGMELRKGSLESPATDVFGSTEEGEKRWKDLKNRVVE
HNIRIMAKYYTRITMKRMAQLLDLSVDESEAFLSNLVVNKTIFAKVDRLAGIINFQRPKD
PNNLLNDWSQKLNSLMSLVNKTTHLIAKEEMIHNLQ
>sp|P12111|CO6A3_HUMAN Collagen alpha-3(VI) chain OS=Homo sapiens OX=9606 GN=COL6A3 PE=1 SV=5
MRKHRHLPLVAVFCLFLSGFPTTHAQQQQADVKNGAAADIIFLVDSSWTIGEEHFQLVRE
FLYDVVKSLAVGENDFHFALVQFNGNPHTEFLLNTYRTKQEVLSHISNMSYIGGTNQTGK
GLEYIMQSHLTKAAGSRAGDGVPQVIVVLTDGHSKDGLALPSAELKSADVNVFAIGVEDA
DEGALKEIASEPLNMHMFNLENFTSLHDIVGNLVSCVHSSVSPERAGDTETLKDITAQDS
ADIIFLIDGSNNTGSVNFAVILDFLVNLLEKLPIGTQQIRVGVVQFSDEPRTMFSLDTYS
TKAQVLGAVKALGFAGGELANIGLALDFVVENHFTRAGGSRVEEGVPQVLVLISAGPSSD
EIRYGVVALKQASVFSFGLGAQAASRAELQHIATDDNLVFTVPEFRSFGDLQEKLLPYIV
GVAQRHIVLKPPTIVTQVIEVNKRDIVFLVDGSSALGLANFNAIRDFIAKVIQRLEIGQD
LIQVAVAQYADTVRPEFYFNTHPTKREVITAVRKMKPLDGSALYTGSALDFVRNNLFTSS
AGYRAAEGIPKLLVLITGGKSLDEISQPAQELKRSSIMAFAIGNKGADQAELEEIAFDSS
LVFIPAEFRAAPLQGMLPGLLAPLRTLSGTPEVHSNKRDIIFLLDGSANVGKTNFPYVRD
FVMNLVNSLDIGNDNIRVGLVQFSDTPVTEFSLNTYQTKSDILGHLRQLQLQGGSGLNTG
SALSYVYANHFTEAGGSRIREHVPQLLLLLTAGQSEDSYLQAANALTRAGILTFCVGASQ
ANKAELEQIAFNPSLVYLMDDFSSLPALPQQLIQPLTTYVSGGVEEVPLAQPESKRDILF
LFDGSANLVGQFPVVRDFLYKIIDELNVKPEGTRIAVAQYSDDVKVESRFDEHQSKPEIL
NLVKRMKIKTGKALNLGYALDYAQRYIFVKSAGSRIEDGVLQFLVLLVAGRSSDRVDGPA
SNLKQSGVVPFIFQAKNADPAELEQIVLSPAFILAAESLPKIGDLHPQIVNLLKSVHNGA
PAPVSGEKDVVFLLDGSEGVRSGFPLLKEFVQRVVESLDVGQDRVRVAVVQYSDRTRPEF
YLNSYMNKQDVVNAVRQLTLLGGPTPNTGAALEFVLRNILVSSAGSRITEGVPQLLIVLT
ADRSGDDVRNPSVVVKRGGAVPIGIGIGNADITEMQTISFIPDFAVAIPTFRQLGTVQQV
ISERVTQLTREELSRLQPVLQPLPSPGVGGKRDVVFLIDGSQSAGPEFQYVRTLIERLVD
YLDVGFDTTRVAVIQFSDDPKVEFLLNAHSSKDEVQNAVQRLRPKGGRQINVGNALEYVS
RNIFKRPLGSRIEEGVPQFLVLISSGKSDDEVDDPAVELKQFGVAPFTIARNADQEELVK
ISLSPEYVFSVSTFRELPSLEQKLLTPITTLTSEQIQKLLASTRYPPPAVESDAADIVFL
IDSSEGVRPDGFAHIRDFVSRIVRRLNIGPSKVRVGVVQFSNDVFPEFYLKTYRSQAPVL
DAIRRLRLRGGSPLNTGKALEFVARNLFVKSAGSRIEDGVPQHLVLVLGGKSQDDVSRFA
QVIRSSGIVSLGVGDRNIDRTELQTITNDPRLVFTVREFRELPNIEERIMNSFGPSAATP
APPGVDTPPPSRPEKKKADIVFLLDGSINFRRDSFQEVLRFVSEIVDTVYEDGDSIQVGL

VQYNSDPTDEFFLKDFSTKRQIIDAINKVVYKGGRHANTKVGLEHLRVNHFVPEAGSRLD
QRVPQIAFVITGGKSVEDAQDVSLALTQRGVKVFAVGVRNIDSEEVGKIASNSATAFRVG
NVQELSELSEQVLETLHDAMHETLCPGVTDAAKACNLDVILGFDGSRDQNVFVAQKGFES
KVDAILNRISQMHRVSCSGGRSPTVRVSVVANTPSGPVEAFDFDEYQPEMLEKFRNMRSQ
HPYVLTEDTLKVYLNKFRQSSPDSVKVVIHFTDGADGDLADLHRASENLRQEGVRALILV
GLERVVNLERLMHLEFGRGFMYDRPLRLNLLDLDYELAEQLDNIAEKACCGVPCKCSGQR
GDRGPIGSIGPKGIPGEDGYRGYPGDEGGPGERGPPGVNGTQGFQGCPGQRGVKGSRGFP
GEKGEVGEIGLDGLDGEDGDKGLPGSSGEKGNPGRRGDKGPRGEKGERGDVGIRGDPGNP
GQDSQERGPKGETGDLGPMGVPGRDGVPGGPGETGKNGGFGRRGPPGAKGNKGGPGQPGF
EGEQGTRGAQGPAGPAGPPGLIGEQGISGPRGSGGAAGAPGERGRTGPLGRKGEPGEPGP
KGGIGNRGPRGETGDDGRDGVGSEGRRGKKGERGFPGYPGPKGNPGEPGLNGTTGPKGIR
GRRGNSGPPGIVGQKGDPGYPGPAGPKGNRGDSIDQCALIQSIKDKCPCCYGPLECPVFP
TELAFALDTSEGVNQDTFGRMRDVVLSIVNDLTIAESNCPRGARVAVVTYNNEVTTEIRF
ADSKRKSVLLDKIKNLQVALTSKQQSLETAMSFVARNTFKRVRNGFLMRKVAVFFSNTPT
RASPQLREAVLKLSDAGITPLFLTRQEDRQLINALQINNTAVGHALVLPAGRDLTDFLEN
VLTCHVCLDICNIDPSCGFGSWRPSFRDRRAAGSDVDIDMAFILDSAETTTLFQFNEMKK
YIAYLVRQLDMSPDPKASQHFARVAVVQHAPSESVDNASMPPVKVEFSLTDYGSKEKLVD
FLSRGMTQLQGTRALGSAIEYTIENVFESAPNPRDLKIVVLMLTGEVPEQQLEEAQRVIL
QAKCKGYFFVVLGIGRKVNIKEVYTFASEPNDVFFKLVDKSTELNEEPLMRFGRLLPSFV
SSENAFYLSPDIRKQCDWFQGDQPTKNLVKFGHKQVNVPNNVTSSPTSNPVTTTKPVTTT
KPVTTTTKPVTTTTKPVTIINQPSVKPAAAKPAPAKPVAAKPVATKMATVRPPVAVKPAT
AAKPVAAKPAAVRPPAAAAAKPVATKPEVPRPQAAKPAATKPATTKPMVKMSREVQVFEI
TENSAKLHWERAEPPGPYFYDLTVTSAHDQSLVLKQNLTVTDRVIGGLLAGQTYHVAVVC
YLRSQVRATYHGSFSTKKSQPPPPQPARSASSSTINLMVSTEPLALTETDICKLPKDEGT
CRDFILKWYYDPNTKSCARFWYGGCGGNENKFGSQKECEKVCAPVLAKPGVISVMGT
>sp|P09543|CN37_HUMAN 2',3'-cyclic-nucleotide 3'-phosphodiesterase OS=Homo sapiens OX=9606 GN=CNP PE=1 SV=2
MNRGFSRKSHTFLPKIFFRKMSSSGAKDKPELQFPFLQDEDTVATLLECKTLFILRGLPG
SGKSTLARVIVDKYRDGTKMVSADAYKITPGARGAFSEEYKRLDEDLAAYCRRRDIRILV
LDDTNHERERLEQLFEMADQYQYQVVLVEPKTAWRLDCAQLKEKNQWQLSADDLKKLKPG
LEKDFLPLYFGWFLTKKSSETLRKAGQVFLEELGNHKAFKKELRQFVPGDEPREKMDLVT
YFGKRPPGVLHCTTKFCDYGKAPGAEEYAQQDVLKKSYSKAFTLTISALFVTPKTTGARV
ELSEQQLQLWPSDVDKLSPTDNLPRGSRAHITLGCAADVEAVQTGLDLLEILRQEKGGSR
GEEVGELSRGKLYSLGNGRWMLTLAKNMEVRAIFTGYYGKGKPVPTQGSRKGGALQSCTI
I
>sp|Q15366|PCBP2_HUMAN Poly(rC)-binding protein 2 OS=Homo sapiens OX=9606 GN=PCBP2 PE=1 SV=1
MDTGVIEGGLNVTLTIRLLMHGKEVGSIIGKKGESVKKMREESGARINISEGNCPERIIT
LAGPTNAIFKAFAMIIDKLEEDISSSMTNSTAASRPPVTLRLVVPASQCGSLIGKGGCKI
KEIRESTGAQVQVAGDMLPNSTERAITIAGIPQSIIECVKQICVVMLETLSQSPPKGVTI
PYRPKPSSSPVIFAGGQDRYSTGSDSASFPHTTPSMCLNPDLEGPPLEAYTIQGQYAIPQ
PDLTKLHQLAMQQSHFPMTHGNTGFSGIESSSPEVKGYWGLDASAQTTSHELTIPNDLIG
CIIGRQGAKINEIRQMSGAQIKIANPVEGSTDRQVTITGSAASISLAQYLINVRLSSETG
GMGSS
>sp|P61254|RL26_HUMAN 60S ribosomal protein L26 OS=Homo sapiens OX=9606 GN=RPL26 PE=1 SV=1
MKFNPFVTSDRSKNRKRHFNAPSHIRRKIMSSPLSKELRQKYNVRSMPIRKDDEVQVVRG

HYKGQQIGKVVQVYRKKYVIYIERVQREKANGTTVHVGIHPSKVVITRLKLDKDRKKILE
RKAKSRQVGKEKGKYKEETIEKMQE
>sp|Q9UNX3|RL26L_HUMAN 60S ribosomal protein L26-like 1 OS=Homo sapiens OX=9606 GN=RPL26L1 PE=1 SV=1
MKFNPFVTSDRSKNRKRHFNAPSHVRRKIMSSPLSKELRQKYNVRSMPIRKDDEVQVVRG
HYKGQQIGKVVQVYRKKYVIYIERVQREKANGTTVHVGIHPSKVVITRLKLDKDRKKILE
RKAKSRQVGKEKGKYKEELIEKMQE
>sp|P36406|TRI23_HUMAN E3 ubiquitin-protein ligase TRIM23 OS=Homo sapiens OX=9606 GN=TRIM23 PE=1 SV=1
MATLVVNKLGAGVDSGRQGSRGTAVVKVLECGVCEDVFSLQGDKVPRLLLCGHTVCHDCL
TRLPLHGRAIRCPFDRQVTDLGDSGVWGLKKNFALLELLERLQNGPIGQYGAAEESIGIS
GESIIRCDEDEAHLASVYCTVCATHLCSECSQVTHSTKTLAKHRRVPLADKPHEKTMCSQ
HQVHAIEFVCLEEGCQTSPLMCCVCKEYGKHQGHKHSVLEPEANQIRASILDMAHCIRTF
TEEISDYSRKLVGIVQHIEGGEQIVEDGIGMAHTEHVPGTAENARSCIRAYFYDLHETLC
RQEEMALSVVDAHVREKLIWLRQQQEDMTILLSEVSAACLHCEKTLQQDDCRVVLAKQEI
TRLLETLQKQQQQFTEVADHIQLDASIPVTFTKDNRVHIGPKMEIRVVTLGLDGAGKTTI
LFKLKQDEFMQPIPTIGFNVETVEYKNLKFTIWDVGGKHKLRPLWKHYYLNTQAVVFVVD
SSHRDRISEAHSELAKLLTEKELRDALLLIFANKQDVAGALSVEEITELLSLHKLCCGRS
WYIQGCDARSGMGLYEGLDWLSRQLVAAGVLDVA
>sp|P62330|ARF6_HUMAN ADP-ribosylation factor 6 OS=Homo sapiens OX=9606 GN=ARF6 PE=1 SV=2
MGKVLSKIFGNKEMRILMLGLDAAGKTTILYKLKLGQSVTTIPTVGFNVETVTYKNVKFN
VWDVGGQDKIRPLWRHYYTGTQGLIFVVDCADRDRIDEARQELHRIINDREMRDAIILIF
ANKQDLPDAMKPHEIQEKLGLTRIRDRNWYVQPSCATSGDGLYEGLTWLTSNYKS
>sp|P13473|LAMP2_HUMAN Lysosome-associated membrane glycoprotein 2 OS=Homo sapiens OX=9606 GN=LAMP2 PE=1 SV=2
MVCFRLFPVPGSGLVLVCLVLGAVRSYALELNLTDSENATCLYAKWQMNFTVRYETTNKT
YKTVTISDHGTVTYNGSICGDDQNGPKIAVQFGPGFSWIANFTKAASTYSIDSVSFSYNT
GDNTTFPDAEDKGILTVDELLAIRIPLNDLFRCNSLSTLEKNDVVQHYWDVLVQAFVQNG
TVSTNEFLCDKDKTSTVAPTIHTTVPSPTTTPTPKEKPEAGTYSVNNGNDTCLLATMGLQ
LNITQDKVASVININPNTTHSTGSCRSHTALLRLNSSTIKYLDFVFAVKNENRFYLKEVN
ISMYLVNGSVFSIANNNLSYWDAPLGSSYMCNKEQTVSVSGAFQINTFDLRVQPFNVTQG
KYSTAQDCSADDDNFLVPIAVGAALAGVLILVLLAYFIGLKHHHAGYEQF
>sp|P02766|TTHY_HUMAN Transthyretin OS=Homo sapiens OX=9606 GN=TTR PE=1 SV=1
MASHRLLLLCLAGLVFVSEAGPTGTGESKCPLMVKVLDAVRGSPAINVAVHVFRKAADDT
WEPFASGKTSESGELHGLTTEEEFVEGIYKVEIDTKSYWKALGISPFHEHAEVVFTANDS
GPRRYTIAALLSPYSYSTTAVVTNPKE
>sp|P23284|PPIB_HUMAN Peptidyl-prolyl cis-trans isomerase B OS=Homo sapiens OX=9606 GN=PPIB PE=1 SV=2
MLRLSERNMKVLLAAALIAGSVFFLLLPGPSAADEKKKGPKVTVKVYFDLRIGDEDVGRV
IFGLFGKTVPKTVDNFVALATGEKGFGYKNSKFHRVIKDFMIQGGDFTRGDGTGGKSIYG
ERFPDENFKLKHYGPGWVSMANAGKDTNGSQFFITTVKTAWLDGKHVVFGKVLEGMEVVR
KVESTKTDSRDKPLKDVIIADCGKIEVEKPFAIAKE
>sp|Q9H2U1|DHX36_HUMAN ATP-dependent DNA/RNA helicase DHX36 OS=Homo sapiens OX=9606 GN=DHX36 PE=1 SV=2
MSYDYHQNWGRDGGPRSSGGGYGGGPAGGHGGNRGSGGGGGGGGGGRGGRGRHPGHLKGR

EIGMWYAKKQGQKNKEAERQERAVVHMDERREEQIVQLLNSVQAKNDKESEAQISWFAPE
DHGYGTEVSTKNTPCSENKLDIQEKKLINQEKKMFRIRNRSYIDRDSEYLLQENEPDGTL
DQKLLEDLQKKKNDLRYIEMQHFREKLPSYGMQKELVNLIDNHQVTVISGETGCGKTTQV
TQFILDNYIERGKGSACRIVCTQPRRISAISVAERVAAERAESCGSGNSTGYQIRLQSRL
PRKQGSILYCTTGIILQWLQSDPYLSSVSHIVLDEIHERNLQSDVLMTVVKDLLNFRSDL
KVILMSATLNAEKFSEYFGNCPMIHIPGFTFPVVEYLLEDVIEKIRYVPEQKEHRSQFKR
GFMQGHVNRQEKEEKEAIYKERWPDYVRELRRRYSASTVDVIEMMEDDKVDLNLIVALIR
YIVLEEEDGAILVFLPGWDNISTLHDLLMSQVMFKSDKFLIIPLHSLMPTVNQTQVFKRT
PPGVRKIVIATNIAETSITIDDVVYVIDGGKIKETHFDTQNNISTMSAEWVSKANAKQRK
GRAGRVQPGHCYHLYNGLRASLLDDYQLPEILRTPLEELCLQIKILRLGGIAYFLSRLMD
PPSNEAVLLSIRHLMELNALDKQEELTPLGVHLARLPVEPHIGKMILFGALFCCLDPVLT
IAASLSFKDPFVIPLGKEKIADARRKELAKDTRSDHLTVVNAFEGWEEARRRGFRYEKDY
CWEYFLSSNTLQMLHNMKGQFAEHLLGAGFVSSRNPKDPESNINSDNEKIIKAVICAGLY
PKVAKIRLNLGKKRKMVKVYTKTDGLVAVHPKSVNVEQTDFHYNWLIYHLKMRTSSIYLY
DCTEVSPYCLLFFGGDISIQKDNDQETIAVDEWIVFQSPARIAHLVKELRKELDILLQEK
IESPHPVDWNDTKSRDCAVLSAIIDLIKTQEKATPRNFPPRFQDGYYS
>sp|Q6P158|DHX57_HUMAN Putative ATP-dependent RNA helicase DHX57 OS=Homo sapiens OX=9606 GN=DHX57 PE=1 SV=2
MSSSVRRKGKPGKGGGKGSSRGGRGGRSHASKSHGSGGGGGGGGGGGGGNRKASSRIWDD
GDDFCIFSESRRPSRPSNSNISKGESRPKWKPKAKVPLQTLHMTSENQEKVKALLRDLQE
QDADAGSERGLSGEEEDDEPDCCNDERYWPAGQEPSLVPDLDPLEYAGLASVEPYVPEFT
VSPFAVQKLSRYGFNTERCQAVLRMCDGDVGASLEHLLTQCFSETFGERMKISEAVNQIS
LDECMEQRQEEAFALKSICGEKFIERIQNRVWTIGLELEYLTSRFRKSKPKESTKNVQEN
SLEICKFYLKGNCKFGSKCRFKHEVPPNQIVGRIERSVDDSHLNAIEDASFLYELEIRFS
KDHKYPYQAPLVAFYSTNENLPLACRLHISEFLYDKALTFAETSEPVVYSLITLLEEESE
IVKLLTNTHHKYSDPPVNFLPVPSRTRINNPACHKTVIPNNSFVSNQIPEVEKASESEES
DEDDGPAPVIVENESYVNLKKKISKRYDWQAKSVHAENGKICKQFRMKQASRQFQSILQE
RQSLPAWEERETILNLLRKHQVVVISGMTGCGKTTQIPQFILDDSLNGPPEKVANIICTQ
PRRISAISVAERVAKERAERVGLTVGYQIRLESVKSSATRLLYCTTGVLLRRLEGDTALQ
GVSHIIVDEVHERTEESDFLLLVLKDIVSQRPGLQVILMSATLNAELFSDYFNSCPVITI
PGRTFPVDQFFLEDAIAVTRYVLQDGSPYMRSMKQISKEKLKARRNRTAFEEVEEDLRLS
LHLQDQDSVKDAVPDQQLDFKQLLARYKGVSKSVIKTMSIMDFEKVNLELIEALLEWIVD
GKHSYPPGAILVFLPGLAEIKMLYEQLQSNSLFNNRRSNRCVIHPLHSSLSSEEQQAVFV
KPPAGVTKIIISTNIAETSITIDDVVYVIDSGKMKEKRYDASKGMESLEDTFVSQANALQ
RKGRAGRVASGVCFHLFTSHHYNHQLLKQQLPEIQRVPLEQLCLRIKILEMFSAHNLQSV
FSRLIEPPHTDSLRASKIRLRDLGALTPDERLTPLGYHLASLPVDVRIGKLMLFGSIFRC
LDPALTIAASLAFKSPFVSPWDKKEEANQKKLEFAFANSDYLALLQAYKGWQLSTKEGVR
ASYNYCRQNFLSGRVLQEMASLKRQFTELLSDIGFAREGLRAREIEKRAQGGDGVLDATG
EEANSNAENPKLISAMLCAALYPNVVQVKSPEGKFQKTSTGAVRMQPKSAELKFVTKNDG
YVHIHPSSVNYQVRHFDSPYLLYHEKIKTSRVFIRDCSMVSVYPLVLFGGGQVNVQLQRG
EFVVSLDDGWIRFVAASHQVAELVKELRCELDQLLQDKIKNPSIDLCTCPRGSRIISTIV
KLVTTQ
>sp|P19957|ELAF_HUMAN Elafin OS=Homo sapiens OX=9606 GN=PI3 PE=1 SV=3
MRASSFLIVVVFLIAGTLVLEAAVTGVPVKGQDTVKGRVPFNGQDPVKGQVSVKGQDKVK
AQEPVKGPVSTKPGSCPIILIRCAMLNPPNRCLKDTDCPGIKKCCEGSCGMACFVPQ

>sp|P46779|RL28_HUMAN 60S ribosomal protein L28 OS=Homo sapiens OX=9606 GN=RPL28 PE=1 SV=3
MSAHLQWMVVRNCSSFLIKRNKQTYSTEPNNLKARNSFRYNGLIHRKTVGVEPAADGKGV
VVVIKRRSGQRKPATSYVRTTINKNARATLSSIRHMIRKNKYRPDLRMAAIRRASAILRS
QKPVMVKRKRTRPTKSS
>sp|P25786|PSA1_HUMAN Proteasome subunit alpha type-1 OS=Homo sapiens OX=9606 GN=PSMA1 PE=1 SV=1
MFRNQYDNDVTVWSPQGRIHQIEYAMEAVKQGSATVGLKSKTHAVLVALKRAQSELAAHQ
KKILHVDNHIGISIAGLTADARLLCNFMRQECLDSRFVFDRPLPVSRLVSLIGSKTQIPT
QRYGRRPYGVGLLIAGYDDMGPHIFQTCPSANYFDCRAMSIGARSQSARTYLERHMSEFM
ECNLNELVKHGLRALRETLPAEQDLTTKNVSIGIVGKDLEFTIYDDDDVSPFLEGLEERP
QRKAQPAQPADEPAEKADEPMEH
>sp|P07602|SAP_HUMAN Prosaposin OS=Homo sapiens OX=9606 GN=PSAP PE=1 SV=2
MYALFLLASLLGAALAGPVLGLKECTRGSAVWCQNVKTASDCGAVKHCLQTVWNKPTVKS
LPCDICKDVVTAAGDMLKDNATEEEILVYLEKTCDWLPKPNMSASCKEIVDSYLPVILDI
IKGEMSRPGEVCSALNLCESLQKHLAELNHQKQLESNKIPELDMTEVVAPFMANIPLLLY
PQDGPRSKPQPKDNGDVCQDCIQMVTDIQTAVRTNSTFVQALVEHVKEECDRLGPGMADI
CKNYISQYSEIAIQMMMHMQPKEICALVGFCDEVKEMPMQTLVPAKVASKNVIPALELVE
PIKKHEVPAKSDVYCEVCEFLVKEVTKLIDNNKTEKEILDAFDKMCSKLPKSLSEECQEV
VDTYGSSILSILLEEVSPELVCSMLHLCSGTRLPALTVHVTQPKDGGFCEVCKKLVGYLD
RNLEKNSTKQEILAALEKGCSFLPDPYQKQCDQFVAEYEPVLIEILVEVMDPSFVCLKIG
ACPSAHKPLLGTEKCIWGPSYWCQNTETAAQCNAVEHCKRHVWN
>sp|O95208|EPN2_HUMAN Epsin-2 OS=Homo sapiens OX=9606 GN=EPN2 PE=1 SV=3
MTTSSIRRQMKNIVNNYSEAEIKVREATSNDPWGPSSSLMTEIADLTYNVVAFSEIMSMV
WKRLNDHGKNWRHVYKALTLLDYLIKTGSERVAQQCRENIFAIQTLKDFQYIDRDGKDQG
INVREKSKQLVALLKDEERLKAERAQALKTKERMAQVATGMGSNQITFGRGSSQPNLSTS
HSEQEYGKAGGSPASYHGSPEASLCPQHRTGAPLGQSEELQPLSQRHPFLPHLGLASRPN
GDWSQPCLTCDRAARATSPRVSSELEQARPQTSGEEELQLQLALAMSREVAEQEERLRRG
DDLRLQMALEESRRDTVKIPKKKEHGSLPQQTTLLDLMDALPSSGPAAQKAEPWGPSAST
NQTNPWGGPAAPASTSDPWPSFGTKPAASIDPWGVPTGATVQSVPKNSDPWAASQQPASS
AGKRASDAWGAVSTTKPVSVSGSFELFSNLNGTIKDDFSEFDNLRTSKKTAESVTSLPSQ
NNGTTSPDPFESQPLTVASSKPSSARKTPESFLGPNAALVNLDSLVTRPAPPAQSLNPFL
APGAPATSAPVNPFQVNQPQPLTLNQLRGSPVLGTSTSFGPGPGVESMAVASMTSAAPQP
ALGATGSSLTPLGPAMMNMVGSVGIPPSAAQATGTTNPFLL
>sp|Q9H201|EPN3_HUMAN Epsin-3 OS=Homo sapiens OX=9606 GN=EPN3 PE=2 SV=1
MTTSALRRQVKNIVHNYSEAEIKVREATSNDPWGPPSSLMSEIADLTFNTVAFTEVMGML
WRRLNDSGKNWRHVYKALTLLDYLLKTGSERVAHQCRENLYTIQTLKDFQYIDRDGKDQG
VNVREKVKQVMALLKDEERLRQERTHALKTKERMALEGIGIGSGQLGFSRRYGEDYSRSR
GSPSSYNSSSSSPRYTSDLEQARPQTSGEEELQLQLALAMSREEAEKPVPPASHRDEDLQ
LQLALRLSRQEHEKEVRSWQGDGSPMANGAGAVVHHQRDREPEREERKEEEKLKTSQSSI
LDLADIFVPALAPPSTHCSADPWDIPGFRPNTEASGSSWGPSADPWSPIPSGTVLSRSQP
WDLTPMLSSSEPWGRTPVLPAGPPTTDPWALNSPHHKLPSTGADPWGASLETSDTPGGAS
TFDPFAKPPESTETKEGLEQALPSGKPSSPVELDLFGDPSPSSKQNGTKEPDALDLGILG
EALTQPSKEARACRTPESFLGPSASSLVNLDSLVKAPQVAKTRNPFLTGLSAPSPTNPFG

AGEPGRPTLNQMRTGSPALGLAGGPVGAPLGSMTYSASLPLPLSSVPAGLTLPASVSVFP
QAGAFAPQPLLPTPSSAGPRPPPPQTGTNPFL
>sp|P57088|TMM33_HUMAN Transmembrane protein 33 OS=Homo sapiens OX=9606 GN=TMEM33 PE=1 SV=2
MADTTPNGPQGAGAVQFMMTNKLDTAMWLSRLFTVYCSALFVLPLLGLHEAASFYQRALL
ANALTSALRLHQRLPHFQLSRAFLAQALLEDSCHYLLYSLIFVNSYPVTMSIFPVLLFSL
LHAATYTKKVLDARGSNSLPLLRSVLDKLSANQQNILKFIACNEIFLMPATVFMLFSGQG
SLLQPFIYYRFLTLRYSSRRNPYCRTLFNELRIVVEHIIMKPACPLFVRRLCLQSIAFIS
RLAPTVP
>sp|P49454|CENPF_HUMAN Centromere protein F OS=Homo sapiens OX=9606 GN=CENPF PE=1 SV=3
MSWALEEWKEGLPTRALQKIQELEGQLDKLKKEKQQRQFQLDSLEAALQKQKQKVENEKT
EGTNLKRENQRLMEICESLEKTKQKISHELQVKESQVNFQEGQLNSGKKQIEKLEQELKR
CKSELERSQQAAQSADVSLNPCNTPQKIFTTPLTPSQYYSGSKYEDLKEKYNKEVEERKR
LEAEVKALQAKKASQTLPQATMNHRDIARHQASSSVFSWQQEKTPSHLSSNSQRTPIRRD
FSASYFSGEQEVTPSRSTLQIGKRDANSSFFDNSSSPHLLDQLKAQNQELRNKINELELR
LQGHEKEMKGQVNKFQELQLQLEKAKVELIEKEKVLNKCRDELVRTTAQYDQASTKYTAL
EQKLKKLTEDLSCQRQNAESARCSLEQKIKEKEKEFQEELSRQQRSFQTLDQECIQMKAR
LTQELQQAKNMHNVLQAELDKLTSVKQQLENNLEEFKQKLCRAEQAFQASQIKENELRRS
MEEMKKENNLLKSHSEQKAREVCHLEAELKNIKQCLNQSQNFAEEMKAKNTSQETMLRDL
QEKINQQENSLTLEKLKLAVADLEKQRDCSQDLLKKREHHIEQLNDKLSKTEKESKALLS
ALELKKKEYEELKEEKTLFSCWKSENEKLLTQMESEKENLQSKINHLETCLKTQQIKSHE
YNERVRTLEMDRENLSVEIRNLHNVLDSKSVEVETQKLAYMELQQKAEFSDQKHQKEIEN
MCLKTSQLTGQVEDLEHKLQLLSNEIMDKDRCYQDLHAEYESLRDLLKSKDASLVTNEDH
QRSLLAFDQQPAMHHSFANIIGEQGSMPSERSECRLEADQSPKNSAILQNRVDSLEFSLE
SQKQMNSDLQKQCEELVQIKGEIEENLMKAEQMHQSFVAETSQRISKLQEDTSAHQNVVA
ETLSALENKEKELQLLNDKVETEQAEIQELKKSNHLLEDSLKELQLLSETLSLEKKEMSS
IISLNKREIEELTQENGTLKEINASLNQEKMNLIQKSESFANYIDEREKSISELSDQYKQ
EKLILLQRCEETGNAYEDLSQKYKAAQEKNSKLECLLNECTSLCENRKNELEQLKEAFAK
EHQEFLTKLAFAEERNQNLMLELETVQQALRSEMTDNQNNSKSEAGGLKQEIMTLKEEQN
KMQKEVNDLLQENEQLMKVMKTKHECQNLESEPIRNSVKERESERNQCNFKPQMDLEVKE
ISLDSYNAQLVQLEAMLRNKELKLQESEKEKECLQHELQTIRGDLETSNLQDMQSQEISG
LKDCEIDAEEKYISGPHELSTSQNDNAHLQCSLQTTMNKLNELEKICEILQAEKYELVTE
LNDSRSECITATRKMAEEVGKLLNEVKILNDDSGLLHGELVEDIPGGEFGEQPNEQHPVS
LAPLDESNSYEHLTLSDKEVQMHFAELQEKFLSLQSEHKILHDQHCQMSSKMSELQTYVD
SLKAENLVLSTNLRNFQGDLVKEMQLGLEEGLVPSLSSSCVPDSSSLSSLGDSSFYRALL
EQTGDMSLLSNLEGAVSANQCSVDEVFCSSLQEENLTRKETPSAPAKGVEELESLCEVYR
QSLEKLEEKMESQGIMKNKEIQELEQLLSSERQELDCLRKQYLSENEQWQQKLTSVTLEM
ESKLAAEKKQTEQLSLELEVARLQLQGLDLSSRSLLGIDTEDAIQGRNESCDISKEHTSE
TTERTPKHDVHQICDKDAQQDLNLDIEKITETGAVKPTGECSGEQSPDTNYEPPGEDKTQ
GSSECISELSFSGPNALVPMDFLGNQEDIHNLQLRVKETSNENLRLLHVIEDRDRKVESL
LNEMKELDSKLHLQEVQLMTKIEACIELEKIVGELKKENSDLSEKLEYFSCDHQELLQRV
ETSEGLNSDLEMHADKSSREDIGDNVAKVNDSWKERFLDVENELSRIRSEKASIEHEALY
LEADLEVVQTEKLCLEKDNENKQKVIVCLEEELSVVTSERNQLRGELDTMSKKTTALDQL
SEKMKEKTQELESHQSECLHCIQVAEAEVKEKTELLQTLSSDVSELLKDKTHLQEKLQSL
EKDSQALSLTKCELENQIAQLNKEKELLVKESESLQARLSESDYEKLNVSKALEAALVEK
GEFALRLSSTQEEVHQLRRGIEKLRVRIEADEKKQLHIAEKLKERERENDSLKDKVENLE

RELQMSEENQELVILDAENSKAEVETLKTQIEEMARSLKVFELDLVTLRSEKENLTKQIQ
EKQGQLSELDKLLSSFKSLLEEKEQAEIQIKEESKTAVEMLQNQLKELNEAVAALCGDQE
IMKATEQSLDPPIEEEHQLRNSIEKLRARLEADEKKQLCVLQQLKESEHHADLLKGRVEN
LERELEIARTNQEHAALEAENSKGEVETLKAKIEGMTQSLRGLELDVVTIRSEKENLTNE
LQKEQERISELEIINSSFENILQEKEQEKVQMKEKSSTAMEMLQTQLKELNERVAALHND
QEACKAKEQNLSSQVECLELEKAQLLQGLDEAKNNYIVLQSSVNGLIQEVEDGKQKLEKK
DEEISRLKNQIQDQEQLVSKLSQVEGEHQLWKEQNLELRNLTVELEQKIQVLQSKNASLQ
DTLEVLQSSYKNLENELELTKMDKMSFVEKVNKMTAKETELQREMHEMAQKTAELQEELS
GEKNRLAGELQLLLEEIKSSKDQLKELTLENSELKKSLDCMHKDQVEKEGKVREEIAEYQ
LRLHEAEKKHQALLLDTNKQYEVEIQTYREKLTSKEECLSSQKLEIDLLKSSKEELNNSL
KATTQILEELKKTKMDNLKYVNQLKKENERAQGKMKLLIKSCKQLEEEKEILQKELSQLQ
AAQEKQKTGTVMDTKVDELTTEIKELKETLEEKTKEADEYLDKYCSLLISHEKLEKAKEM
LETQVAHLCSQQSKQDSRGSPLLGPVVPGPSPIPSVTEKRLSSGQNKASGKRQRSSGIWE
NGRGPTPATPESFSKKSKKAVMSGIHPAEDTEGTEFEPEGLPEVVKKGFADIPTGKTSPY
ILRRTTMATRTSPRLAAQKLALSPLSLGKENLAESSKPTAGGSRSQKVKVAQRSPVDSGT
ILREPTTKSVPVNNLPERSPTDSPREGLRVKRGRLVPSPKAGLESNGSENCKVQ
>sp|P62917|RL8_HUMAN 60S ribosomal protein L8 OS=Homo sapiens OX=9606 GN=RPL8 PE=1 SV=2
MGRVIRGQRKGAGSVFRAHVKHRKGAARLRAVDFAERHGYIKGIVKDIIHDPGRGAPLAK
VVFRDPYRFKKRTELFIAAEGIHTGQFVYCGKKAQLNIGNVLPVGTMPEGTIVCCLEEKP
GDRGKLARASGNYATVISHNPETKKTRVKLPSGSKKVISSANRAVVGVVAGGGRIDKPIL
KAGRAYHKYKAKRNCWPRVRGVAMNPVEHPFGGGNHQHIGKPSTIRRDAPAGRKVGLIAA
RRTGRLRGTKTVQEKEN
>sp|Q969L2|MAL2_HUMAN Protein MAL2 OS=Homo sapiens OX=9606 GN=MAL2 PE=1 SV=1
MSAGGASVPPPPNPAVSFPPPRVTLPAGPDILRTYSGAFVCLEILFGGLVWILVASSNVP
LPLLQGWVMFVSVTAFFFSLLFLGMFLSGMVAQIDANWNFLDFAYHFTVFVFYFGAFLLE
AAATSLHDLHCNTTITGQPLLSDNQYNINVAASIFAFMTTACYGCSLGLALRRWRP
>sp|Q96DD7|SHSA4_HUMAN Protein shisa-4 OS=Homo sapiens OX=9606 GN=SHISA4 PE=1 SV=3
MPPAGLRRAAPLTAIALLVLGAPLVLAGEDCLWYLDRNGSWHPGFNCEFFTFCCGTCYHR
YCCRDLTLLITERQQKHCLAFSPKTIAGIASAVILFVAVVATTICCFLCSCCYLYRRRQQ
LQSPFEGQEIPMTGIPVQPVYPYPQDPKAGPAPPQPGFIYPPSGPAPQYPLYPAGPPVYN
PAAPPPYMPPQPSYPGA
>sp|P02652|APOA2_HUMAN Apolipoprotein A-II OS=Homo sapiens OX=9606 GN=APOA2 PE=1 SV=1
MKLLAATVLLLTICSLEGALVRRQAKEPCVESLVSQYFQTVTDYGKDLMEKVKSPELQAE
AKSYFEKSKEQLTPLIKKAGTELVNFLSYFVELGTQPATQ
>sp|P17302|CXA1_HUMAN Gap junction alpha-1 protein OS=Homo sapiens OX=9606 GN=GJA1 PE=1 SV=2
MGDWSALGKLLDKVQAYSTAGGKVWLSVLFIFRILLLGTAVESAWGDEQSAFRCNTQQPG
CENVCYDKSFPISHVRFWVLQIIFVSVPTLLYLAHVFYVMRKEEKLNKKEEELKVAQTDG
VNVDMHLKQIEIKKFKYGIEEHGKVKMRGGLLRTYIISILFKSIFEVAFLLIQWYIYGFS
LSAVYTCKRDPCPHQVDCFLSRPTEKTIFIIFMLVVSLVSLALNIIELFYVFFKGVKDRV
KGKSDPYHATSGALSPAKDCGSQKYAYFNGCSSPTAPLSPMSPPGYKLVTGDRNNSSCRN
YNKQASEQNWANYSAEQNRMGQAGSTISNSHAQPFDFPDDNQNSKKLAAGHELQPLAIVD
QRPSSRASSRASSRPRPDDLEI

>sp|Q9NTQ9|CXB4_HUMAN Gap junction beta-4 protein OS=Homo sapiens OX=9606 GN=GJB4 PE=1 SV=1
MNWAFLQGLLSGVNKYSTVLSRIWLSVVFIFRVLVYVVAAEEVWDDEQKDFVCNTKQPGC
PNVCYDEFFPVSHVRLWALQLILVTCPSLLVVMHVAYREERERKHHLKHGPNAPSLYDNL
SKKRGGLWWTYLLSLIFKAAVDAGFLYIFHRLYKDYDMPRVVACSVEPCPHTVDCYISRP
TEKKVFTYFMVTTAAICILLNLSEVFYLVGKRCMEIFGPRHRRPRCRECLPDTCPPYVLS
QGGHPEDGNSVLMKAGSAPVDAGGYP
>sp|Q8TF72|SHRM3_HUMAN Protein Shroom3 OS=Homo sapiens OX=9606 GN=SHROOM3 PE=1 SV=2
MMRTTEDFHKPSATLNSNTATKGRYIYLEAFLEGGAPWGFTLKGGLEHGEPLIISKVEEG
GKADTLSSKLQAGDEVVHINEVTLSSSRKEAVSLVKGSYKTLRLVVRRDVCTDPGHADTG
ASNFVSPEHLTSGPQHRKAAWSGGVKLRLKHRRSEPAGRPHSWHTTKSGEKQPDASMMQI
SQGMIGPPWHQSYHSSSSTSDLSNYDHAYLRRSPDQCSSQGSMESLEPSGAYPPCHLSPA
KSTGSIDQLSHFHNKRDSAYSSFSTSSSILEYPHPGISGRERSGSMDNTSARGGLLEGMR
QADIRYVKTVYDTRRGVSAEYEVNSSALLLQGREARASANGQGYDKWSNIPRGKGVPPPS
WSQQCPSSLETATDNLPPKVGAPLPPARSDSYAAFRHRERPSSWSSLDQKRLCRPQANSL
GSLKSPFIEEQLHTVLEKSPENSPPVKPKHNYTQKAQPGQPLLPTSIYPVPSLEPHFAQV
PQPSVSSNGMLYPALAKESGYIAPQGACNKMATIDENGNQNGSGRPGFAFCQPLEHDLLS
PVEKKPEATAKYVPSKVHFCSVPENEEDASLKRHLTPPQGNSPHSNERKSTHSNKPSSHP
HSLKCPQAQAWQAGEDKRSSRLSEPWEGDFQEDHNANLWRRLEREGLGQSLSGNFGKTKS
AFSSLQNIPESLRRHSSLELGRGTQEGYPGGRPTCAVNTKAEDPGRKAAPDLGSHLDRQV
SYPRPEGRTGASASFNSTDPSPEEPPAPSHPHTSSLGRRGPGPGSASALQGFQYGKPHCS
VLEKVSKFEQREQGSQRPSVGGSGFGHNYRPHRTVSTSSTSGNDFEETKAHIRFSESAEP
LGNGEQHFKNGELKLEEASRQPCGQQLSGGASDSGRGPQRPDARLLRSQSTFQLSSEPER
EPEWRDRPGSPESPLLDAPFSRAYRNSIKDAQSRVLGATSFRRRDLELGAPVASRSWRPR
PSSAHVGLRSPEASASASPHTPRERHSVTPAEGDLARPVPPAARRGARRRLTPEQKKRSY
SEPEKMNEVGIVEEAEPAPLGPQRNGMRFPESSVADRRRLFERDGKACSTLSLSGPELKQ
FQQSALADYIQRKTGKRPTSAAGCSLQEPGPLRERAQSAYLQPGPAALEGSGLASASSLS
SLREPSLQPRREATLLPATVAETQQAPRDRSSSFAGGRRLGERRRGDLLSGANGGTRGTQ
RGDETPREPSSWGARAGKSMSAEDLLERSDVLAGPVHVRSRSSPATADKRQDVLLGQDSG
FGLVKDPCYLAGPGSRSLSCSERGQEEMLPLFHHLTPRWGGSGCKAIGDSSVPSECPGTL
DHQRQASRTPCPRPPLAGTQGLVTDTRAAPLTPIGTPLPSAIPSGYCSQDGQTGRQPLPP
YTPAMMHRSNGHTLTQPPGPRGCEGDGPEHGVEEGTRKRVSLPQWPPPSRAKWAHAARED
SLPEESSAPDFANLKHYQKQQSLPSLCSTSDPDTPLGAPSTPGRISLRISESVLRDSPPP
HEDYEDEVFVRDPHPKATSSPTFEPLPPPPPPPPSQETPVYSMDDFPPPPPHTVCEAQLD
SEDPEGPRPSFNKLSKVTIARERHMPGAAHVVGSQTLASRLQTSIKGSEAESTPPSFMSV
HAQLAGSLGGQPAPIQTQSLSHDPVSGTQGLEKKVSPDPQKSSEDIRTEALAKEIVHQDK
SLADILDPDSRLKTTMDLMEGLFPRDVNLLKENSVKRKAIQRTVSSSGCEGKRNEDKEAV
SMLVNCPAYYSVSAPKAELLNKIKEMPAEVNEEEEQADVNEKKAELIGSLTHKLETLQEA
KGSLLTDIKLNNALGEEVEALISELCKPNEFDKYRMFIGDLDKVVNLLLSLSGRLARVEN
VLSGLGEDASNEERSSLYEKRKILAGQHEDARELKENLDRRERVVLGILANYLSEEQLQD
YQHFVKMKSTLLIEQRKLDDKIKLGQEQVKCLLESLPSDFIPKAGALALPPNLTSEPIPA
GGCTFSGIFPTLTSPL
>sp|Q16378|PROL4_HUMAN Proline-rich protein 4 OS=Homo sapiens OX=9606 GN=PRR4 PE=1 SV=3
MLLVLLSVVLLALSSAQSTDNDVNYEDFTFTIPDVEDSSQRPDQGPQRPPPEGLLPRPPG
DSGNQDDGPQQRPPKPGGHHRHPPPPPFQNQQRPPRRGHRQLSLPRFPSVSLQEASSFFQ

RDRPARHPQEQPLW
>sp|P45877|PPIC_HUMAN Peptidyl-prolyl cis-trans isomerase C OS=Homo sapiens OX=9606 GN=PPIC PE=1 SV=1
MGPGPRLLLPLVLCVGLGALVFSSGAEGFRKRGPSVTAKVFFDVRIGDKDVGRIVIGLFG
KVVPKTVENFVALATGEKGYGYKGSKFHRVIKDFMIQGGDITTGDGTGGVSIYGETFPDE
NFKLKHYGIGWVSMANAGPDTNGSQFFITLTKPTWLDGKHVVFGKVIDGMTVVHSIELQA
TDGHDRPLTNCSIINSGKIDVKTPFVVEIADW
>sp|P50995|ANX11_HUMAN Annexin A11 OS=Homo sapiens OX=9606 GN=ANXA11 PE=1 SV=1
MSYPGYPPPPGGYPPAAPGGGPWGGAAYPPPPSMPPIGLDNVATYAGQFNQDYLSGMAAN
MSGTFGGANMPNLYPGAPGAGYPPVPPGGFGQPPSAQQPVPPYGMYPPPGGNPPSRMPSY
PPYPGAPVPGQPMPPPGQQPPGAYPGQPPVTYPGQPPVPLPGQQQPVPSYPGYPGSGTVT
PAVPPTQFGSRGTITDAPGFDPLRDAEVLRKAMKGFGTDEQAIIDCLGSRSNKQRQQILL
SFKTAYGKDLIKDLKSELSGNFEKTILALMKTPVLFDIYEIKEAIKGVGTDEACLIEILA
SRSNEHIRELNRAYKAEFKKTLEEAIRSDTSGHFQRLLISLSQGNRDESTNVDMSLAQRD
AQELYAAGENRLGTDESKFNAVLCSRSRAHLVAVFNEYQRMTGRDIEKSICREMSGDLEE
GMLAVVKCLKNTPAFFAERLNKAMRGAGTKDRTLIRIMVSRSETDLLDIRSEYKRMYGKS
LYHDISGDTSGDYRKILLKICGGND
>sp|O75508|CLD11_HUMAN Claudin-11 OS=Homo sapiens OX=9606 GN=CLDN11 PE=1 SV=2
MVATCLQVVGFVTSFVGWIGVIVTTSTNDWVVTCGYTIPTCRKLDELGSKGLWADCVMAT
GLYHCKPLVDILILPGYVQACRALMIAASVLGLPAILLLLTVLPCIRMGQEPGVAKYRRA
QLAGVLLILLALCALVATIWFPVCAHRETTIVSFGYSLYAGWIGAVLCLVGGCVILCCAG
DAQAFGENRFYYTAGSSSPTHAKSAHV
>sp|P0DME0|SETLP_HUMAN Protein SETSIP OS=Homo sapiens OX=9606 GN=SETSIP PE=1 SV=1
MVWFLDFPNSMAPKRQSPLPLQKKKPRPPPALGLEETSASAGLPKKGEKEQQEAIEHIDE
VQNEIDRLNEQDSEEILKVEQKYNKLRQPFFQKRSELIAKIPNFGVTTFVNHPQVSSLLG
EEDEEALHYLTKVEVTEFEDIKSGYRIDFYFDENPYFENKVFSKEFHLNESGDPSSKSTK
IKWKSGKDVTKRSSQTQNKASRKRQHEEPESFFTWFTDHSDAGADELEEVIKDDIWPNPL
QYYLVPDMDDEEGGEDDDDDDDDGDEGEEELEDIDEGDEDEGEEDEDDDEGEEGEEDEGE
DD
>sp|Q01105|SET_HUMAN Protein SET OS=Homo sapiens OX=9606 GN=SET PE=1 SV=3
MAPKRQSPLPPQKKKPRPPPALGPEETSASAGLPKKGEKEQQEAIEHIDEVQNEIDRLNE
QASEEILKVEQKYNKLRQPFFQKRSELIAKIPNFWVTTFVNHPQVSALLGEEDEEALHYL
TRVEVTEFEDIKSGYRIDFYFDENPYFENKVLSKEFHLNESGDPSSKSTEIKWKSGKDLT
KRSSQTQNKASRKRQHEEPESFFTWFTDHSDAGADELGEVIKDDIWPNPLQYYLVPDMDD
EEGEGEEDDDDDEEEEGLEDIDEEGDEDEGEEDEDDDEGEEGEEDEGEDD
>sp|O43426|SYNJ1_HUMAN Synaptojanin-1 OS=Homo sapiens OX=9606 GN=SYNJ1 PE=1 SV=2
MAFSKGFRIYHKLDPPPFSLIVETRHKEECLMFESGAVAVLSSAEKEAIKGTYSKVLDAY
GLLGVLRLNLGDTMLHYLVLVTGCMSVGKIQESEVFRVTSTEFISLRIDSSDEDRISEVR
KVLNSGNFYFAWSASGISLDLSLNAHRSMQEQTTDNRFFWNQSLHLHLKHYGVNCDDWLL
RLMCGGVEIRTIYAAHKQAKACLISRLSCERAGTRFNVRGTNDDGHVANFVETEQVVYLD
DSVSSFIQIRGSVPLFWEQPGLQVGSHRVRMSRGFEANAPAFDRHFRTLKNLYGKQIIVN
LLGSKEGEHMLSKAFQSHLKASEHAADIQMVNFDYHQMVKGGKAEKLHSVLKPQVQKFLD

YGFFYFNGSEVQRCQSGTVRTNCLDCLDRTNSVQAFLGLEMLAKQLEALGLAEKPQLVTR
FQEVFRSMWSVNGDSISKIYAGTGALEGKAKLKDGARSVTRTIQNNFFDSSKQEAIDVLL
LGNTLNSDLADKARALLTTGSLRVSEQTLQSASSKVLKSMCENFYKYSKPKKIRVCVGTW
NVNGGKQFRSIAFKNQTLTDWLLDAPKLAGIQEFQDKRSKPTDIFAIGFEEMVELNAGNI
VSASTTNQKLWAVELQKTISRDNKYVLLASEQLVGVCLFVFIRPQHAPFIRDVAVDTVKT
GMGGATGNKGAVAIRMLFHTTSLCFVCSHFAAGQSQVKERNEDFIEIARKLSFPMGRMLF
SHDYVFWCGDFNYRIDLPNEEVKELIRQQNWDSLIAGDQLINQKNAGQVFRGFLEGKVTF
APTYKYDLFSDDYDTSEKCRTPAWTDRVLWRRRKWPFDRSAEDLDLLNASFQDESKILYT
WTPGTLLHYGRAELKTSDHRPVVALIDIDIFEVEAEERQNIYKEVIAVQGPPDGTVLVSI
KSSLPENNFFDDALIDELLQQFASFGEVILIRFVEDKMWVTFLEGSSALNVLSLNGKELL
NRTITIALKSPDWIKNLEEEMSLEKISIALPSSTSSTLLGEDAEVAADFDMEGDVDDYSA
EVEELLPQHLQPSSSSGLGTSPSSSPRTSPCQSPTISEGPVPSLPIRPSRAPSRTPGPPS
AQSSPIDAQPATPLPQKDPAQPLEPKRPPPPRPVAPPTRPAPPQRPPPPSGARSPAPTRK
EFGGIGAPPSPGVARREMEAPKSPGTTRKDNIGRSQPSPQAGLAGPGPAGYSTARPTIPP
RAGVISAPQSHARASAGRLTPESQSKTSETSKGSTFLPEPLKPQAAFPPQSSLPPPAQRL
QEPLVPVAAPMPQSGPQPNLETPPQPPPRSRSSHSLPSEASSQPQVKTNGISDGKRESPL
KIDPFEDLSFNLLAVSKAQLSVQTSPVPTPDPKRLIQLPSATQSNVLSSVSCMPTMPPIP
ARSQSQENMRSSPNPFITGLTRTNPFSDRTAAPGNPFRAKSEESEATSWFSKEEPVTISP
FPSLQPLGHNKSRASSSLDGFKDSFDLQGQSTLKISNPKGWVTFEEEEDFGVKGKSKSAC
SDLLGNQPSSFSGSNLTLNDDWNKGTNVSFCVLPSRRPPPPPVPLLPPGTSPPVDPFTTL
ASKASPTLDFTER
>sp|P16401|H15_HUMAN Histone H1.5 OS=Homo sapiens OX=9606 GN=H1-5 PE=1 SV=3
MSETAPAETATPAPVEKSPAKKKATKKAAGAGAAKRKATGPPVSELITKAVAASKERNGL
SLAALKKALAAGGYDVEKNNSRIKLGLKSLVSKGTLVQTKGTGASGSFKLNKKAASGEAK
PKAKKAGAAKAKKPAGATPKKAKKAAGAKKAVKKTPKKAKKPAAAGVKKVAKSPKKAKAA
AKPKKATKSPAKPKAVKPKAAKPKAAKPKAAKPKAAKAKKAAAKKK
>sp|Q96JG9|ZN469_HUMAN Zinc finger protein 469 OS=Homo sapiens OX=9606 GN=ZNF469 PE=1 SV=3
MPGERPRGAPPPTMTGDLQPRQVASSPGHPSQPPLEDNTPATRTTKGAREAGGQAQAMEL
PEAQPRQARDGELKPPSLRGQAPSSTPGKRGSPQTPPGRSPLQAPSRLAGRAEGSPPQRY
ILGIASSRTKPTLDETPENPQLEAAQLPEVDTPQGPGTGAPLRPGLPRTEAQPAAEELGF
HRCFQEPPSSFTSTNYTSPSATPRPPAPGPPQSRGTSPLQPGSYPEYQASGADSWPPAAE
NSFPGANFGVPPAEPEPIPKGSRPGGSPRGVSFQFPFPALHGASTKPFPADVAGHAFTNG
PLVFAFHQPQGAWPEEAVGTGPAYPLPTQPAPSPLPCYQGQPGGLNRHSDLSGALSSPGA
AHSAPRPFSDSLHKSLTKILPERPPSAQDGLGSTRGPPSSLPQRHFPGQAYRASGVDTSP
GPPDTELAAPGPPPARLPQLWDPTAAPYPTPPGGPLAATRSMFFNGQPSPGQRLCLPQSA
PLPWPQVLPTARPSPHGMEMLSRLPFPAGGPEWQGGSQGALGTAGKTPGPREKLPAVRSS
QGGSPALFTYNGMTDPGAQPLFFGVAQPQVSPHGTPSLPPPRVVGASPSESPLPSPATNT
AGSTCSSLSPMSSSPANPSSEESQLPGPLGPSAFFHPPTHPQETGSPFPSPEPPHSLPTH
YQPEPAKAFPFPADGLGAEGAFQCLEETPFPHEGPEVGRGGLQGFPRAPPPYPTHHFSLS
SASLDQLDVLLTCRQCDRNYSSLAAFLAHRQFCGLLLARAKDGHQRSPGPPGLPSPPAAP
RVPADAHAGLLSHAKTFLLAGDAQAEGKDDPLRTGFLPSLAATPFPLPASDLDMEDDAKL
DSLITEALNGMEYQSDNPEIDSSFIDVFADEEPSGPRGPSSGHPLKSKAGVTPESKAPPP
LPAATPDPQTPRPGDRGCPARGRPKTRSLGLAPTEADAPSQGRQQRRGKQLKLFRKDLDS
GGAAEGSGSGGGGRASGLRPRRNDGLGERPPPRPRRPRTQAPGSRADPAPRVPRAAALPE
ETRSSRRRRLPPRKDPRKRKARGGAWGKELILKIVQQKNRLREYDFASESEEDEQPPPRG

PGFRGRRGRGEKRKEVELTQGPREDEPQKPRKAARQEAGGDGAPANPEEPGGSRPGPGRS
PQARGPSRSLETGAAAREGGPKCADRPSVAPKDPLQVPTNTETSEETRPSLDFPQEAKEP
ETAEESAPDSTEFTEALRSPPAACAGEMGASPGLLIPEQPPPSRHDTGTPKPSGSLANTA
PHGSSPTPGVGSLLGGPGGTQAPVSHNSKDPPARQPGEFLAPVANPSSTACPKPSVLSSK
ISSFGCDPAGFNRDPLGVPVAKKGPQPYSSPHSELFLGPKDLAGCFLEELHPKPSARDAP
PASSSCLCQDGEDAGSLEPQLPRSPPGTAETEPGRAASPPTLESSSLFPDLPVDRFDPPL
YGSLSANRDSGLPFACADPPQKTVPSDPPYPSFLLLEEVSPMLPSHFPDLSGGKVLSKTC
PPERTVVPGAAPSLPGKGSGCSVALMSHLSEDELEIQKLVTELESQLQRSKDTRGAPREL
AEAESVGRVELGTGTEPPSQRRTCQATVPHEDTFSAADLTRVGESTAHREGAESAVATVE
AVQGRPGGTWPCPASFHPGHAALLPCAQEDLVSGAPFSPRGANFHFQPVQKAGASKTGLC
QAEGDSRPPQDVCLPEPSKQPGPQLDAGSLAKCSPDQELSFPKNKEAASSQESEDSLRLL
PCEQRGGFLPEPGTADQPHRGAPAPEAFGSPAVHLAPDLAFQGDGAPPLDATWPFGASPS
HAAQGHSAGRAGGHLHPTAGRPGFEGNEFAPAGASSLTAPRGREAWLVPVPSPACVSNTH
PSRRSQDPALSPPIRQLQLPGPGVAKSKDGILGLQELTPAAQSPPRVNPSGLEGGTVEGG
KVACGPAQGSPGGVQVTTLPAVAGHQLGLEADGHWGLLGQAEKTQGQGTANQLQPENGVS
PGGTDNHASVNASPKTALTGPTEGAVLLEKCKGSRAAMSLQEEAEPTPSPPSPNRESLAL
ALTAAHSRSGSEGRTPERASSPGLNKPLLATGDSPAPSVGDLAACAPSPTSAAHMPCSLG
PLPREDPLTSPSRAQGGLGGQLPASPSCRDPPGPQQLLACSPAWAPLEEADGVQATTDTG
AEDSPVAPPSLTTSPCDPKEALAGCLLQGEGSPLEDPSSWPPGSVSAVTCTHSGDTPKDS
TLRIPEDSRKEKLWESPGRATSPPLAGAVSPSVAVRATGLSSTPTGDEAQAGRGLPGPDP
QSRGAPPHTNPDRMPRGHSSYSPSNTARLGHREGQAVTAVPTEPPTLQGAGPDSPACLEG
EMGTSSKEPEDPGTPETGRSGATKMPRVTCPSTGLGLGRTTAPSSTASDFQSDSPQSHRN
ASHQTPQGDPLGPQDLKQRSRGYKKKPASTENGQWKGQAPHGPVTCEVCAASFRSGPGLS
RHKARKHRPHPGAPAEPSPAALPAQQPLEPLAQKCQPPRKKSHRVSGKERPNHSRGDPSH
VTQPPPAQGSKEVLRAPGSPHSQQLHPPSPTEHEVDVKTPASKPRPDQAREDELHPKQAE
KREGRRWRREPTVDSPSHSEGKSNKKRGKLRGRRLREESILPVSADVISDGRGSRPSPAM
ASYAASPSHCLSVEGGPEADGEQPPRLATLGPGVMEGAAETDQEALCAGETGAQKPPGDR
MLCPGRMDGAALGEQPTGQKGASARGFWGPRETKALGVCKESGSEPAEDSSRAHSRSEEG
VWEENTPPLGPLGFPETSSSPADSTTSSCLQGLPDNPDTQGGVQGPEGPTPDASGSSAKD
PPSLFDDEVSFSQLFPPGGRLTRKRNPHVYGKRCEKPVLPLPTQPSFEEGGDPTLGPARL
PTDLSDSSSLCLCHEDPWEDEDPAGLPESFLLDGFLNSRVPGIDPWAPGLSLWALEPSRE
AGAEKLPSHCPEDDRPEAIPELHMVPAAWRGLEMPAPADDSSSSLGDVSPEPPSLERERC
DGGLPGNTHLLPLRATDFEVLSTKFEMQDLCFLGPFEDPVGLPGPSFLDFEGTASSQGPQ
SRRTEEAAGAGRAQGRGRPAKGRRASYKCKVCFQRFRSLGELDLHKLAHTPAPPPTCYMC
VERRFGSRELLRGHLQERHAQSKAGPWACGMCLKEVADVWMYNEHLREHAVRFARRGQAR
RSLGDLPGGLEGSSAVAHLLNSITEPAPKHHRGKRSAGKAAGSPGDPWGQEGEAKKDSPG
ERAKPRARSTPSNPDGAATPDSASATALADAGSPGPPRTTPSPSPDPWAGGEPLLQATPV
HEACKDPSRDCHHCGKRFPKPFKLQRHLAVHSPQRVYLCPRCPRVYPEHGELLAHLGGAH
GLLERPELQHTPLYACELCATVMRIIKKSFACSSCNYTFAKKEQFDRHMNKHLRGGRQPF
AFRGVRRPGAPGQKARALEGTLPSKRRRVAMPGSAPGPGEDRPPPRGSSPILSEGSLPAL
LHLCSEVAPSTTKGWPETLERPVDPVTHPIRGCELPSNHQECPPPSLSPFPAALADGRGD
CALDGALERPENEASPGSPGPLLQQALPLGASLPRPGARGQDAEGKRAPLVFSGKRRAPG
ARGRCAPDHFQEDHLLQKEKEVSSSHMVSEGGPRGTFHKGSATKPAGCQSSSKDRSAAST
PSKALKFPVHPRKAVGSLAPGELARGTENGMKPATPKAKPGPSSQGSGSPRPGTKTGGGS
QPQPASGQLQSETATTPAKPSFPSRSPAPERLPARAQAKSCTKGPREAGEQGPHGSLGPK
EKGESSTKRKKGQVPGPARSESVGSFGRAPSAPDKPPRTPRKQATPSRVLPTKPKPNSQN
KPRPPPSEQRKAEPGHTQRKDRLGKAFPQGRPLLRPPKRGTAVHGAEPAEPHTHRTAEAQ

SDLLSQLFGQRLTGFKIPLKKDASE
>sp|Q99417|MYCBP_HUMAN c-Myc-binding protein OS=Homo sapiens OX=9606 GN=MYCBP PE=1 SV=3
MAHYKAADSKREQFRRYLEKSGVLDTLTKVLVALYEEPEKPNSALDFLKHHLGAATPENP
EIELLRLELAEMKEKYEAIVEENKKLKAKLAQYEPPQEEKRAE
>sp|Q14677|EPN4_HUMAN Clathrin interactor 1 OS=Homo sapiens OX=9606 GN=CLINT1 PE=1 SV=1
MLNMWKVRELVDKATNVVMNYSEIESKVREATNDDPWGPSGQLMGEIAKATFMYEQFPEL
MNMLWSRMLKDNKKNWRRVYKSLLLLAYLIRNGSERVVTSAREHIYDLRSLENYHFVDEH
GKDQGINIRQKVKELVEFAQDDDRLREERKKAKKNKDKYVGVSSDSVGGFRYSERYDPEP
KSKWDEEWDKNKSAFPFSDKLGELSDKIGSTIDDTISKFRRKDREDSPERCSDSDEEKKA
RRGRSPKGEFKDEEETVTTKHIHITQATETTTTRHKRTANPSKTIDLGAAAHYTGDKASP
DQNASTHTPQSSVKTSVPSSKSSGDLVDLFDGTSQSTGGSADLFGGFADFGSAAASGSFP
SQVTATSGNGDFGDWSAFNQAPSGPVASSGEFFGSASQPAVELVSGSQSALGPPPAASNS
SDLFDLMGSSQATMTSSQSMNFSMMSTNTVGLGLPMSRSQNTDMVQKSVSKTLPSTWSDP
SVNISLDNLLPGMQPSKPQQPSLNTMIQQQNMQQPMNVMTQSFGAVNLSSPSNMLPVRPQ
TNALIGGPMPMSMPNVMTGTMGMAPLGNTPMMNQSMMGMNMNIGMSAAGMGLTGTMGMGM
PNIAMTSGTVQPKQDAFANFANFSK
>sp|Q99996|AKAP9_HUMAN A-kinase anchor protein 9 OS=Homo sapiens OX=9606 GN=AKAP9 PE=1 SV=4
MEDEERQKKLEAGKAKLAQFRQRKAQSDGQSPSKKQKKKRKTSSSKHDVSAHHDLNIDQS
QCNEMYINSSQRVESTVIPESTIMRTLHSGEITSHEQGFSVELESEISTTADDCSSEVNG
CSFVMRTGKPTNLLREEEFGVDDSYSEQGAQDSPTHLEMMESELAGKQHEIEELNRELEE
MRVTYGTEGLQQLQEFEAAIKQRDGIITQLTANLQQARREKDETMREFLELTEQSQKLQI
QFQQLQASETLRNSTHSSTAADLLQAKQQILTHQQQLEEQDHLLEDYQKKKEDFTMQISF
LQEKIKVYEMEQDKKVENSNKEEIQEKETIIEELNTKIIEEEKKTLELKDKLTTADKLLG
ELQEQIVQKNQEIKNMKLELTNSKQKERQSSEEIKQLMGTVEELQKRNHKDSQFETDIVQ
RMEQETQRKLEQLRAELDEMYGQQIVQMKQELIRQHMAQMEEMKTRHKGEMENALRSYSN
ITVNEDQIKLMNVAINELNIKLQDTNSQKEKLKEELGLILEEKCALQRQLEDLVEELSFS
REQIQRARQTIAEQESKLNEAHKSLSTVEDLKAEIVSASESRKELELKHEAEVTNYKIKL
EMLEKEKNAVLDRMAESQEAELERLRTQLLFSHEEELSKLKEDLEIEHRINIEKLKDNLG
IHYKQQIDGLQNEMSQKIETMQFEKDNLITKQNQLILEISKLKDLQQSLVNSKSEEMTLQ
INELQKEIEILRQEEKEKGTLEQEVQELQLKTELLEKQMKEKENDLQEKFAQLEAENSIL
KDEKKTLEDMLKIHTPVSQEERLIFLDSIKSKSKDSVWEKEIEILIEENEDLKQQCIQLN
EEIEKQRNTFSFAEKNFEVNYQELQEEYACLLKVKDDLEDSKNKQELEYKSKLKALNEEL
HLQRINPTTVKMKSSVFDEDKTFVAETLEMGEVVEKDTTELMEKLEVTKREKLELSQRLS
DLSEQLKQKHGEISFLNEEVKSLKQEKEQVSLRCRELEIIINHNRAENVQSCDTQVSSLL
DGVVTMTSRGAEGSVSKVNKSFGEESKIMVEDKVSFENMTVGEESKQEQLILDHLPSVTK
ESSLRATQPSENDKLQKELNVLKSEQNDLRLQMEAQRICLSLVYSTHVDQVREYMENEKD
KALCSLKEELIFAQEEKIKELQKIHQLELQTMKTQETGDEGKPLHLLIGKLQKAVSEECS
YFLQTLCSVLGEYYTPALKCEVNAEDKENSGDYISENEDPELQDYRYEVQDFQENMHTLL
NKVTEEYNKLLVLQTRLSKIWGQQTDGMKLEFGEENLPKEETEFLSIHSQMTNLEDIDVN
HKSKLSSLQDLEKTKLEEQVQELESLISSLQQQLKETEQNYEAEIHCLQKRLQAVSESTV
PPSLPVDSVVITESDAQRTMYPGSCVKKNIDGTIEFSGEFGVKEETNIVKLLEKQYQEQL
EEEVAKVIVSMSIAFAQQTELSRISGGKENTASSKQAHAVCQQEQHYFNEMKLSQDQIGF
QTFETVDVKFKEEFKPLSKELGEHGKEILLSNSDPHDIPESKDCVLTISEEMFSKDKTFI
VRQSIHDEISVSSMDASRQLMLNEEQLEDMRQELVRQYQEHQQATELLRQAHMRQMERQR

EDQEQLQEEIKRLNRQLAQRSSIDNENLVSERERVLLEELEALKQLSLAGREKLCCELRN
SSTQTQNGNENQGEVEEQTFKEKELDRKPEDVPPEILSNERYALQKANNRLLKILLEVVK
TTAAVEETIGRHVLGILDRSSKSQSSASLIWRSEAEASVKSCVHEEHTRVTDESIPSYSG
SDMPRNDINMWSKVTEEGTELSQRLVRSGFAGTEIDPENEELMLNISSRLQAAVEKLLEA
ISETSSQLEHAKVTQTELMRESFRQKQEATESLKCQEELRERLHEESRAREQLAVELSKA
EGVIDGYADEKTLFERQIQEKTDIIDRLEQELLCASNRLQELEAEQQQIQEERELLSRQK
EAMKAEAGPVEQQLLQETEKLMKEKLEVQCQAEKVRDDLQKQVKALEIDVEEQVSRFIEL
EQEKNTELMDLRQQNQALEKQLEKMRKFLDEQAIDREHERDVFQQEIQKLEQQLKVVPRF
QPISEHQTREVEQLANHLKEKTDKCSELLLSKEQLQRDIQERNEEIEKLEFRVRELEQAL
LVSADTFQKVEDRKHFGAVEAKPELSLEVQLQAERDAIDRKEKEITNLEEQLEQFREELE
NKNEEVQQLHMQLEIQKKESTTRLQELEQENKLFKDDMEKLGLAIKESDAMSTQDQHVLF
GKFAQIIQEKEVEIDQLNEQVTKLQQQLKITTDNKVIEEKNELIRDLETQIECLMSDQEC
VKRNREEEIEQLNEVIEKLQQELANIGQKTSMNAHSLSEEADSLKHQLDVVIAEKLALEQ
QVETANEEMTFMKNVLKETNFKMNQLTQELFSLKRERESVEKIQSIPENSVNVAIDHLSK
DKPELEVVLTEDALKSLENQTYFKSFEENGKGSIINLETRLLQLESTVSAKDLELTQCYK
QIKDMQEQGQFETEMLQKKIVNLQKIVEEKVAAALVSQIQLEAVQEYAKFCQDNQTISSE
PERTNIQNLNQLREDELGSDISALTLRISELESQVVEMHTSLILEKEQVEIAEKNVLEKE
KKLLELQKLLEGNEKKQREKEKKRSPQDVEVLKTTTELFHSNEESGFFNELEALRAESVA
TKAELASYKEKAEKLQEELLVKETNMTSLQKDLSQVRDHLAEAKEKLSILEKEDETEVQE
SKKACMFEPLPIKLSKSIASQTDGTLKISSSNQTPQILVKNAGIQINLQSECSSEEVTEI
ISQFTEKIEKMQELHAAEILDMESRHISETETLKREHYVAVQLLKEECGTLKAVIQCLRS
KEGSSIPELAHSDAYQTREICSSDSGSDWGQGIYLTHSQGFDIASEGRGEESESATDSFP
KKIKGLLRAVHNEGMQVLSLTESPYSDGEDHSIQQVSEPWLEERKAYINTISSLKDLITK
MQLQREAEVYDSSQSHESFSDWRGELLLALQQVFLEERSVLLAAFRTELTALGTTDAVGL
LNCLEQRIQEQGVEYQAAMECLQKADRRSLLSEIQALHAQMNGRKITLKREQESEKPSQE
LLEYNIQQKQSQMLEMQVELSSMKDRATELQEQLSSEKMVVAELKSELAQTKLELETTLK
AQHKHLKELEAFRLEVKDKTDEVHLLNDTLASEQKKSRELQWALEKEKAKLGRSEERDKE
ELEDLKFSLESQKQRNLQLNLLLEQQKQLLNESQQKIESQRMLYDAQLSEEQGRNLELQV
LLESEKVRIREMSSTLDRERELHAQLQSSDGTGQSRPPLPSEDLLKELQKQLEEKHSRIV
ELLNETEKYKLDSLQTRQQMEKDRQVHRKTLQTEQEANTEGQKKMHELQSKVEDLQRQLE
EKRQQVYKLDLEGQRLQGIMQEFQKQELEREEKRESRRILYQNLNEPTTWSLTSDRTRNW
VLQQKIEGETKESNYAKLIEMNGGGTGCNHELEMIRQKLQCVASKLQVLPQKASERLQFE
TADDEDFIWVQENIDEIILQLQKLTGQQGEEPSLVSPSTSCGSLTERLLRQNAELTGHIS
QLTEEKNDLRNMVMKLEEQIRWYRQTGAGRDNSSRFSLNGGANIEAIIASEKEVWNREKL
TLQKSLKRAEAEVYKLKAELRNDSLLQTLSPDSEHVTLKRIYGKYLRAESFRKALIYQKK
YLLLLLGGFQECEDATLALLARMGGQPAFTDLEVITNRPKGFTRFRSAVRVSIAISRMKF
LVRRWHRVTGSVSININRDGFGLNQGAEKTDSFYHSSGGLELYGEPRHTTYRSRSDLDYI
RSPLPFQNRYPGTPADFNPGSLACSQLQNYDPDRALTDYITRLEALQRRLGTIQSGSTTQ
FHAGMRR
>sp|Q7Z2Z2|EFL1_HUMAN Elongation factor-like GTPase 1 OS=Homo sapiens OX=9606 GN=EFL1 PE=1 SV=2
MVLNSLDKMIQLQKNTANIRNICVLAHVDHGKTTLADCLISSNGIISSRLAGKLRYMDSR
EDEQIRGITMKSSAISLHYATGNEEYLINLIDSPGHVDFSSEVSTAVRICDGCIIVVDAV
EGVCPQTQAVLRQAWLENIRPVLVINKIDRLIVELKFTPQEAYSHLKNILEQINALTGTL
FTSKVLEERAERETESQVNPNSEQGEQVYDWSTGLEDTDDSHLYFSPEQGNVVFTSAIDG
WGFGIEHFARIYSQKIGIKKEVLMKTLWGDYYINMKAKKIMKGDQAKGKKPLFVQLILEN
IWSLYDAVLKKDKDKIDKIVTSLGLKIGAREARHSDPKVQINAICSQWLPISHAVLAMVC

QKLPSPLDITAERVERLMCTGSQTFDSFPPETQALKAAFMKCGSEDTAPVIIFVSKMFAV
DAKALPQNKPRPLTQEEIAQRRERARQRHAEKLAAAQGQAPLEPTQDGSAIETCPKGEEP
RGDEQQVESMTPKPVLQEENNQESFIAFARVFSGVARRGKKIFVLGPKYSPLEFLRRVPL
GFSAPPDGLPQVPHMAYCALENLYLLMGRELEYLEEVPPGNVLGIGGLQDFVLKSATLCS
LPSCPPFIPLNFEATPIVRVAVEPKHPSEMPQLVKGMKLLNQADPCVQILIQETGEHVLV
TAGEVHLQRCLDDLKERFAKIHISVSEPIIPFRETITKPPKVDMVNEEIGKQQKVAVIHQ
MKEDQSKIPEGIQVDSDGLITITTPNKLATLSVRAMPLPEEVTQILEENSDLIRSMEQLT
SSLNEGENTHMIHQKTQEKIWEFKGKLEQHLTGRRWRNIVDQIWSFGPRKCGPNILVNKS
EDFQNSVWTGPADKASKEASRYRDLGNSIVSGFQLATLSGPMCEEPLMGVCFVLEKWDLS
KFEEQGASDLAKEGQEENETCSGGNENQELQDGCSEAFEKRTSQKGESPLTDCYGPFSGQ
LIATMKEACRYALQVKPQRLMAAMYTCDIMATGDVLGRVYAVLSKREGRVLQEEMKEGTD
MFIIKAVLPVAESFGFADEIRKRTSGLASPQLVFSHWEIIPSDPFWVPTTEEEYLHFGEK
ADSENQARKYMNAVRKRKGLYVEEKIVEHAEKQRTLSKNK
>sp|B3EWG5|FM25C_HUMAN Protein FAM25C OS=Homo sapiens OX=9606 GN=FAM25C PE=1 SV=1
MLGGLGKLAAEGLAHRTEKATEGAIHAVEEVVKEVVGHAKETGEKAIAEAIKKAQESGDK
KMKEITETVTNTVTNAITHAAESLDKLGQ
>sp|B3EWG6|FM25G_HUMAN Protein FAM25G OS=Homo sapiens OX=9606 GN=FAM25G PE=3 SV=1
MLGGLGKLAAEGLAHRTEKATEGAIHAVEEVVKEVVGHAKETGEKAIAEAIKKAQESGDK
KMKEITETVTNTVTNAITHAAESLDKLGQ
>sp|B3EWG3|FM25A_HUMAN Protein FAM25A OS=Homo sapiens OX=9606 GN=FAM25A PE=3 SV=1
MLGGLGKLAAEGLAHRTEKATEGAIHAVEEVVKEVVGHAKETGEKAIAEAIKKAQESGDK
KMKEITETVTNTVTNAITHAAESLDKLGQ
>sp|P07196|NFL_HUMAN Neurofilament light polypeptide OS=Homo sapiens OX=9606 GN=NEFL PE=1 SV=3
MSSFSYEPYYSTSYKRRYVETPRVHISSVRSGYSTARSAYSSYSAPVSSSLSVRRSYSSS
SGSLMPSLENLDLSQVAAISNDLKSIRTQEKAQLQDLNDRFASFIERVHELEQQNKVLEA
ELLVLRQKHSEPSRFRALYEQEIRDLRLAAEDATNEKQALQGEREGLEETLRNLQARYEE
EVLSREDAEGRLMEARKGADEAALARAELEKRIDSLMDEISFLKKVHEEEIAELQAQIQY
AQISVEMDVTKPDLSAALKDIRAQYEKLAAKNMQNAEEWFKSRFTVLTESAAKNTDAVRA
AKDEVSESRRLLKAKTLEIEACRGMNEALEKQLQELEDKQNADISAMQDTINKLENELRT
TKSEMARYLKEYQDLLNVKMALDIEIAAYRKLLEGEETRLSFTSVGSITSGYSQSSQVFG
RSAYGGLQTSSYLMSTRSFPSYYTSHVQEEQIEVEETIEAAKAEEAKDEPPSEGEAEEEE
KDKEEAEEEEAAEEEEAAKEESEEAKEEEEGGEGEEGEETKEAEEEEKKVEGAGEEQAAK
KKD
>sp|Q9Y295|DRG1_HUMAN Developmentally-regulated GTP-binding protein 1 OS=Homo sapiens OX=9606 GN=DRG1 PE=1 SV=1
MSSTLAKIAEIEAEMARTQKNKATAHHLGLLKARLAKLRRELITPKGGGGGGPGEGFDVA
KTGDARIGFVGFPSVGKSTLLSNLAGVYSEVAAYEFTTLTTVPGVIRYKGAKIQLLDLPG
IIEGAKDGKGRGRQVIAVARTCNLILIVLDVLKPLGHKKIIENELEGFGIRLNSKPPNIG
FKKKDKGGINLTATCPQSELDAETVKSILAEYKIHNADVTLRSDATADDLIDVVEGNRVY
IPCIYVLNKIDQISIEELDIIYKVPHCVPISAHHRWNFDDLLEKIWDYLKLVRIYTKPKG
QLPDYTSPVVLPYSRTTVEDFCMKIHKNLIKEFKYALVWGLSVKHNPQKVGKDHTLEDED
VIQIVKK

>sp|O15078|CE290_HUMAN Centrosomal protein of 290 kDa OS=Homo sapiens OX=9606 GN=CEP290 PE=1 SV=2
MPPNINWKEIMKVDPDDLPRQEELADNLLISLSKVEVNELKSEKQENVIHLFRITQSLMK
MKAQEVELALEEVEKAGEEQAKFENQLKTKVMKLENELEMAQQSAGGRDTRFLRNEICQL
EKQLEQKDRELEDMEKELEKEKKVNEQLALRNEEAENENSKLRRENKRLKKKNEQLCQDI
IDYQKQIDSQKETLLSRRGEDSDYRSQLSKKNYELIQYLDEIQTLTEANEKIEVQNQEMR
KNLEESVQEMEKMTDEYNRMKAIVHQTDNVIDQLKKENDHYQLQVQELTDLLKSKNEEDD
PIMVAVNAKVEEWKLILSSKDDEIIEYQQMLHNLREKLKNAQLDADKSNVMALQQGIQER
DSQIKMLTEQVEQYTKEMEKNTCIIEDLKNELQRNKGASTLSQQTHMKIQSTLDILKEKT
KEAERTAELAEADAREKDKELVEALKRLKDYESGVYGLEDAVVEIKNCKNQIKIRDREIE
ILTKEINKLELKISDFLDENEALRERVGLEPKTMIDLTEFRNSKHLKQQQYRAENQILLK
EIESLEEERLDLKKKIRQMAQERGKRSATSGLTTEDLNLTENISQGDRISERKLDLLSLK
NMSEAQSKNEFLSRELIEKERDLERSRTVIAKFQNKLKELVEENKQLEEGMKEILQAIKE
MQKDPDVKGGETSLIIPSLERLVNAIESKNAEGIFDASLHLKAQVDQLTGRNEELRQELR
ESRKEAINYSQQLAKANLKIDHLEKETSLLRQSEGSNVVFKGIDLPDGIAPSSASIINSQ
NEYLIHLLQELENKEKKLKNLEDSLEDYNRKFAVIRHQQSLLYKEYLSEKETWKTESKTI
KEEKRKLEDQVQQDAIKVKEYNNLLNALQMDSDEMKKILAENSRKITVLQVNEKSLIRQY
TTLVELERQLRKENEKQKNELLSMEAEVCEKIGCLQRFKEMAIFKIAALQKVVDNSVSLS
ELELANKQYNELTAKYRDILQKDNMLVQRTSNLEHLECENISLKEQVESINKELEITKEK
LHTIEQAWEQETKLGNESSMDKAKKSITNSDIVSISKKITMLEMKELNERQRAEHCQKMY
EHLRTSLKQMEERNFELETKFAELTKINLDAQKVEQMLRDELADSVSKAVSDADRQRILE
LEKNEMELKVEVSKLREISDIARRQVEILNAQQQSRDKEVESLRMQLLDYQAQSDEKSLI
AKLHQHNVSLQLSEATALGKLESITSKLQKMEAYNLRLEQKLDEKEQALYYARLEGRNRA
KHLRQTIQSLRRQFSGALPLAQQEKFSKTMIQLQNDKLKIMQEMKNSQQEHRNMENKTLE
MELKLKGLEELISTLKDTKGAQKVINWHMKIEELRLQELKLNRELVKDKEEIKYLNNIIS
EYERTISSLEEEIVQQNKFHEERQMAWDQREVDLERQLDIFDRQQNEILNAAQKFEEATG
SIPDPSLPLPNQLEIALRKIKENIRIILETRATCKSLEEKLKEKESALRLAEQNILSRDK
VINELRLRLPATAEREKLIAELGRKEMEPKSHHTLKIAHQTIANMQARLNQKEEVLKKYQ
RLLEKAREEQREIVKKHEEDLHILHHRLELQADSSLNKFKQTAWDLMKQSPTPVPTNKHF
IRLAEMEQTVAEQDDSLSSLLVKLKKVSQDLERQREITELKVKEFENIKLQLQENHEDEV
KKVKAEVEDLKYLLDQSQKESQCLKSELQAQKEANSRAPTTTMRNLVERLKSQLALKEKQ
QKALSRALLELRAEMTAAAEERIISATSQKEAHLNVQQIVDRHTRELKTQVEDLNENLLK
LKEALKTSKNRENSLTDNLNDLNNELQKKQKAYNKILREKEEIDQENDELKRQIKRLTSG
LQGKPLTDNKQSLIEELQRKVKKLENQLEGKVEEVDLKPMKEKNAKEELIRWEEGKKWQA
KIEGIRNKLKEKEGEVFTLTKQLNTLKDLFAKADKEKLTLQRKLKTTGMTVDQVLGIRAL
ESEKELEELKKRNLDLENDILYMRAHQALPRDSVVEDLHLQNRYLQEKLHALEKQFSKDT
YSKPSISGIESDDHCQREQELQKENLKLSSENIELKFQLEQANKDLPRLKNQVRDLKEMC
EFLKKEKAEVQRKLGHVRGSGRSGKTIPELEKTIGLMKKVVEKVQRENEQLKKASGILTS
EKMANIEQENEKLKAELEKLKAHLGHQLSMHYESKTKGTEKIIAENERLRKELKKETDAA
EKLRIAKNNLEILNEKMTVQLEETGKRLQFAESRGPQLEGADSKSWKSIVVTRMYETKLK
ELETDIAKKNQSITDLKQLVKEATEREQKVNKYNEDLEQQIKILKHVPEGAETEQGLKRE
LQVLRLANHQLDKEKAELIHQIEANKDQSGAESTIPDADQLKEKIKDLETQLKMSDLEKQ
HLKEEIKKLKKELENFDPSFFEEIEDLKYNYKEEVKKNILLEEKVKKLSEQLGVELTSPV
AASEEFEDEEESPVNFPIY
>sp|O00487|PSDE_HUMAN 26S proteasome non-ATPase regulatory subunit 14 OS=Homo sapiens OX=9606 GN=PSMD14 PE=1 SV=1
MDRLLRLGGGMPGLGQGPPTDAPAVDTAEQVYISSLALLKMLKHGRAGVPMEVMGLMLGE

FVDDYTVRVIDVFAMPQSGTGVSVEAVDPVFQAKMLDMLKQTGRPEMVVGWYHSHPGFGC
WLSGVDINTQQSFEALSERAVAVVVDPIQSVKGKVVIDAFRLINANMMVLGHEPRQTTSN
LGHLNKPSIQALIHGLNRHYYSITINYRKNELEQKMLLNLHKKSWMEGLTLQDYSEHCKH
NESVVKEMLELAKNYNKAVEEEDKMTPEQLAIKNVGKQDPKRHLEEHVDVLMTSNIVQCL
AAMLDTVVFK
>sp|P29322|EPHA8_HUMAN Ephrin type-A receptor 8 OS=Homo sapiens OX=9606 GN=EPHA8 PE=1 SV=2
MAPARGRLPPALWVVTAAAAAATCVSAARGEVNLLDTSTIHGDWGWLTYPAHGWDSINEV
DESFQPIHTYQVCNVMSPNQNNWLRTSWVPRDGARRVYAEIKFTLRDCNSMPGVLGTCKE
TFNLYYLESDRDLGASTQESQFLKIDTIAADESFTGADLGVRRLKLNTEVRSVGPLSKRG
FYLAFQDIGACLAILSLRIYYKKCPAMVRNLAAFSEAVTGADSSSLVEVRGQCVRHSEER
DTPKMYCSAEGEWLVPIGKCVCSAGYEERRDACVACELGFYKSAPGDQLCARCPPHSHSA
APAAQACHCDLSYYRAALDPPSSACTRPPSAPVNLISSVNGTSVTLEWAPPLDPGGRSDI
TYNAVCRRCPWALSRCEACGSGTRFVPQQTSLVQASLLVANLLAHMNYSFWIEAVNGVSD
LSPEPRRAAVVNITTNQAAPSQVVVIRQERAGQTSVSLLWQEPEQPNGIILEYEIKYYEK
DKEMQSYSTLKAVTTRATVSGLKPGTRYVFQVRARTSAGCGRFSQAMEVETGKPRPRYDT
RTIVWICLTLITGLVVLLLLLICKKRHCGYSKAFQDSDEEKMHYQNGQAPPPVFLPLHHP
PGKLPEPQFYAEPHTYEEPGRAGRSFTREIEASRIHIEKIIGSGDSGEVCYGRLRVPGQR
DVPVAIKALKAGYTERQRRDFLSEASIMGQFDHPNIIRLEGVVTRGRLAMIVTEYMENGS
LDTFLRTHDGQFTIMQLVGMLRGVGAGMRYLSDLGYVHRDLAARNVLVDSNLVCKVSDFG
LSRVLEDDPDAAYTTTGGKIPIRWTAPEAIAFRTFSSASDVWSFGVVMWEVLAYGERPYW
NMTNRDVISSVEEGYRLPAPMGCPHALHQLMLDCWHKDRAQRPRFSQIVSVLDALIRSPE
SLRATATVSRCPPPAFVRSCFDLRGGSGGGGGLTVGDWLDSIRMGRYRDHFAAGGYSSLG
MVLRMNAQDVRALGITLMGHQKKILGSIQTMRAQLTSTQGPRRHL
>sp|Q9UL51|HCN2_HUMAN Potassium/sodium hyperpolarization-activated cyclic nucleotide-gated channel 2 OS=Homo sapiens OX=9606 GN=HCN2 PE=1 SV=3
MDARGGGGRPGESPGATPAPGPPPPPPPAPPQQQPPPPPPPAPPPGPGPAPPQHPPRAEA
LPPEAADEGGPRGRLRSRDSSCGRPGTPGAASTAKGSPNGECGRGEPQCSPAGPEGPARG
PKVSFSCRGAASGPAPGPGPAEEAGSEEAGPAGEPRGSQASFMQRQFGALLQPGVNKFSL
RMFGSQKAVEREQERVKSAGAWIIHPYSDFRFYWDFTMLLFMVGNLIIIPVGITFFKDET
TAPWIVFNVVSDTFFLMDLVLNFRTGIVIEDNTEIILDPEKIKKKYLRTWFVVDFVSSIP
VDYIFLIVEKGIDSEVYKTARALRIVRFTKILSLLRLLRLSRLIRYIHQWEEIFHMTYDL
ASAVMRICNLISMMLLLCHWDGCLQFLVPMLQDFPRNCWVSINGMVNHSWSELYSFALFK
AMSHMLCIGYGRQAPESMTDIWLTMLSMIVGATCYAMFIGHATALIQSLDSSRRQYQEKY
KQVEQYMSFHKLPADFRQKIHDYYEHRYQGKMFDEDSILGELNGPLREEIVNFNCRKLVA
SMPLFANADPNFVTAMLTKLKFEVFQPGDYIIREGTIGKKMYFIQHGVVSVLTKGNKEMK
LSDGSYFGEICLLTRGRRTASVRADTYCRLYSLSVDNFNEVLEEYPMMRRAFETVAIDRL
DRIGKKNSILLHKVQHDLNSGVFNNQENAIIQEIVKYDREMVQQAELGQRVGLFPPPPPP
PQVTSAIATLQQAAAMSFCPQVARPLVGPLALGSPRLVRRPPPGPAPAAASPGPPPPASP
PGAPASPRAPRTSPYGGLPAAPLAGPALPARRLSRASRPLSASQPSLPHGAPGPAASTRP
ASSSTPRLGPTPAARAAAPSPDRRDSASPGAAGGLDPQDSARSRLSSNL
>sp|P0C869|PA24B_HUMAN Cytosolic phospholipase A2 beta OS=Homo sapiens OX=9606 GN=PLA2G4B PE=1 SV=2
MAVAEVSRTCLLTVRVLQAHRLPSKDLVTPSDCYVTLWLPTACSHRLQTRTVKNSSSPVW
NQSFHFRIHRQLKNVMELKVFDQDLVTGDDPVLSVLFDAGTLRAGEFRRESFSLSPQGEG
RLEVEFRLQSLADRGEWLVSNGVLVARELSCLHVQLEETGDQKSSEHRVQLVVPGSCEGP

QEASVGTGTFRFHCPACWEQELSIRLQDAPEEQLKAPLSALPSGQVVRLVFPTSQEPLMR
VELKKEAGLRELAVRLGFGPCAEEQAFLSRRKQVVAAALRQALQLDGDLQEDEIPVVAIM
ATGGGIRAMTSLYGQLAGLKELGLLDCVSYITGASGSTWALANLYEDPEWSQKDLAGPTE
LLKTQVTKNKLGVLAPSQLQRYRQELAERARLGYPSCFTNLWALINEALLHDEPHDHKLS
DQREALSHGQNPLPIYCALNTKGQSLTTFEFGEWCEFSPYEVGFPKYGAFIPSELFGSEF
FMGQLMKRLPESRICFLEGIWSNLYAANLQDSLYWASEPSQFWDRWVRNQANLDKEQVPL
LKIEEPPSTAGRIAEFFTDLLTWRPLAQATHNFLRGLHFHKDYFQHPHFSTWKATTLDGL
PNQLTPSEPHLCLLDVGYLINTSCLPLLQPTRDVDLILSLDYNLHGAFQQLQLLGRFCQE
QGIPFPPISPSPEEQLQPRECHTFSDPTCPGAPAVLHFPLVSDSFREYSAPGVRRTPEEA
AAGEVNLSSSDSPYHYTKVTYSQEDVDKLLHLTHYNVCNNQEQLLEALRQAVQRRRQRRP
H
>sp|Q9UNS2|CSN3_HUMAN COP9 signalosome complex subunit 3 OS=Homo sapiens OX=9606 GN=COPS3 PE=1 SV=3
MASALEQFVNSVRQLSAQGQMTQLCELINKSGELLAKNLSHLDTVLGALDVQEHSLGVLA
VLFVKFSMPSVPDFETLFSQVQLFISTCNGEHIRYATDTFAGLCHQLTNALVERKQPLRG
IGILKQAIDKMQMNTNQLTSIHADLCQLCLLAKCFKPALPYLDVDMMDICKENGAYDAKH
FLCYYYYGGMIYTGLKNFERALYFYEQAITTPAMAVSHIMLESYKKYILVSLILLGKVQQ
LPKYTSQIVGRFIKPLSNAYHELAQVYSTNNPSELRNLVNKHSETFTRDNNMGLVKQCLS
SLYKKNIQRLTKTFLTLSLQDMASRVQLSGPQEAEKYVLHMIEDGEIFASINQKDGMVSF
HDNPEKYNNPAMLHNIDQEMLKCIELDERLKAMDQEITVNPQFVQKSMGSQEDDSGNKPS
SYS
>sp|Q96D53|COQ8B_HUMAN Atypical kinase COQ8B, mitochondrial OS=Homo sapiens OX=9606 GN=COQ8B PE=1 SV=2
MWLKVGGLLRGTGGQLGQTVGWPCGALGPGPHRWGPCGGSWAQKFYQDGPGRGLGEEDIR
RAREARPRKTPRPQLSDRSRERKVPASRISRLANFGGLAVGLGLGVLAEMAKKSMPGGRL
QSEGGSGLDSSPFLSEANAERIVQTLCTVRGAALKVGQMLSIQDNSFISPQLQHIFERVR
QSADFMPRWQMLRVLEEELGRDWQAKVASLEEVPFAAASIGQVHQGLLRDGTEVAVKIQY
PGIAQSIQSDVQNLLAVLKMSAALPAGLFAEQSLQALQQELAWECDYRREAACAQNFRQL
LANDPFFRVPAVVKELCTTRVLGMELAGGVPLDQCQGLSQDLRNQICFQLLTLCLRELFE
FRFMQTDPNWANFLYDASSHQVTLLDFGASREFGTEFTDHYIEVVKAAADGDRDCVLQKS
RDLKFLTGFETKAFSDAHVEAVMILGEPFATQGPYDFGSGETARRIQDLIPVLLRHRLCP
PPEETYALHRKLAGAFLACAHLRAHIACRDLFQDTYHRYWASRQPDAATAGSLPTKGDSW
VDPS
>sp|Q5HYK9|ZN667_HUMAN Zinc finger protein 667 OS=Homo sapiens OX=9606 GN=ZNF667 PE=1 SV=2
MPSARGKSKSKAPITFGDLAIYFSQEEWEWLSPIQKDLYEDVMLENYRNLVSLGLSFRRP
NVITLLEKGKAPWMVEPVRRRRAPDSGSKCETKKLPPNQCNKSGQSICQKLVSAQQKAPT
RKSGCNKNSVLVKPKKGHSGKKPLKCNDCGKTFSRSFSLKLHQNIHTGEKPFECSNCRKA
FRQISSILLHQRIHSGKKSHECNKCGESFNQRTTLILHMRIHDGKEILDCGKALSQCQSF
NIHQKIHVVGNVCQCRKCGKAFNQMSSLLLHKKIHNGKKTHKYNKCGRGFKKKSVFVVHK
RIHAGEKIPENAKALSQSLQQRSHHLENPFKCRKCGKLFNRISPLMLHQRIHTSEKPYKC
DKCDKFFRRLSTLILHLRIHNGEKLYRCNKCEKVCNRHSSLIQHQKVHTKKKKLFECKEC
GKMFSGTANLKIHQNIHSEEKPFKCNKCSKVFGRQSFLIEHQRIHTGEKPYQCEECGKAF
SHRISLTRHKRIHTEDRPYECDQCGKAFSQSAHLAQHERIHTGEKPYTCKTCGKAFSQRT
SLILHERSHTGEKPYECNECGKAFSSGSDLIRHQRSHSSEKPYECSKCGKAYSRSSSLIR
HQNTHSEEKA

>sp|Q8IWA5|CTL2_HUMAN Choline transporter-like protein 2 OS=Homo sapiens OX=9606 GN=SLC44A2 PE=1 SV=3
MGDERPHYYGKHGTPQKYDPTFKGPIYNRGCTDIICCVFLLLAIVGYVAVGIIAWTHGDP
RKVIYPTDSRGEFCGQKGTKNENKPYLFYFNIVKCASPLVLLEFQCPTPQICVEKCPDRY
LTYLNARSSRDFEYYKQFCVPGFKNNKGVAEVLQDGDCPAVLIPSKPLARRCFPAIHAYK
GVLMVGNETTYEDGHGSRKNITDLVEGAKKANGVLEARQLAMRIFEDYTVSWYWIIIGLV
IAMAMSLLFIILLRFLAGIMVWVMIIMVILVLGYGIFHCYMEYSRLRGEAGSDVSLVDLG
FQTDFRVYLHLRQTWLAFMIILSILEVIIILLLIFLRKRILIAIALIKEASRAVGYVMCS
LLYPLVTFFLLCLCIAYWASTAVFLSTSNEAVYKIFDDSPCPFTAKTCNPETFPSSNESR
QCPNARCQFAFYGGESGYHRALLGLQIFNAFMFFWLANFVLALGQVTLAGAFASYYWALR
KPDDLPAFPLFSAFGRALRYHTGSLAFGALILAIVQIIRVILEYLDQRLKAAENKFAKCL
MTCLKCCFWCLEKFIKFLNRNAYIMIAIYGTNFCTSARNAFFLLMRNIIRVAVLDKVTDF
LFLLGKLLIVGSVGILAFFFFTHRIRIVQDTAPPLNYYWVPILTVIVGSYLIAHGFFSVY
GMCVDTLFLCFLEDLERNDGSAERPYFMSSTLKKLLNKTNKKAAES
>sp|Q709C8|VP13C_HUMAN Intermembrane lipid transfer protein VPS13C OS=Homo sapiens OX=9606 GN=VPS13C PE=1 SV=1
MVLESVVADLLNRFLGDYVENLNKSQLKLGIWGGNVALDNLQIKENALSELDVPFKVKAG
QIDKLTLKIPWKNLYGEAVVATLEGLYLLVVPGASIKYDAVKEEKSLQDVKQKELSRIEE
ALQKAAEKGTHSGEFIYGLENFVYKDIKPGRKRKKHKKHFKKPFKGLDRSKDKPKEAKKD
TFVEKLATQVIKNVQVKITDIHIKYEDDVTDPKRPLSFGVTLGELSLLTANEHWTPCILN
EADKIIYKLIRLDSLSAYWNVNCSMSYQRSREQILDQLKNEILTSGNIPPNYQYIFQPIS
ASAKLYMNPYAESELKTPKLDCNIEIQNIAIELTKPQYLSMIDLLESVDYMVRNAPYRKY
KPYLPLHTNGRRWWKYAIDSVLEVHIRRYTQMWSWSNIKKHRQLLKSYKIAYKNKLTQSK
VSEEIQKEIQDLEKTLDVFNIILARQQAQVEVIRSGQKLRKKSADTGEKRGGWFSGLWGK
KESKKKDEESLIPETIDDLMTPEEKDKLFTAIGYSESTHNLTLPKQYVAHIMTLKLVSTS
VTIRENKNIPEILKIQIIGLGTQVSQRPGAQALKVEAKLEHWYITGLRQQDIVPSLVASI
GDTTSSLLKIKFETNPEDSPADQTLIVQSQPVEVIYDAKTVNAVVEFFQSNKGLDLEQIT
SATLMKLEEIKERTATGLTHIIETRKVLDLRINLKPSYLVVPQTGFHHEKSDLLILDFGT
FQLNSKDQGLQKTTNSSLEEIMDKAYDKFDVEIKNVQLLFARAEETWKKCRFQHPSTMHI
LQPMDIHVELAKAMVEKDIRMARFKVSGGLPLMHVRISDQKMKDVLYLMNSIPLPQKSSA
QSPERQVSSIPIISGGTKGLLGTSLLLDTVESESDDEYFDAEDGEPQTCKSMKGSELKKA
AEVPNEELINLLLKFEIKEVILEFTKQQKEEDTILVFNVTQLGTEATMRTFDLTVVSYLK
KISLDYHEIEGSKRKPLHLISSSDKPGLDLLKVEYIKADKNGPSFQTAFGKTEQTVKVAF
SSLNLLLQTQALVASINYLTTIIPSDDQSISVAKEVQISTEKQQKNSTLPKAIVSSRDSD
IIDFRLFAKLNAFCVIVCNEKNNIAEIKIQGLDSSLSLQSRKQSLFARLENIIVTDVDPK
TVHKKAVSIMGNEVFRFNLDLYPDATEGDLYTDMSKVDGVLSLNVGCIQIVYLHKFLMSL
LNFLNNFQTAKESLSAATAQAAERAATSVKDLAQRSFRVSINIDLKAPVIVIPQSSISTN
AVVVDLGLIRVHNQFSLVSDEDYLNPPVIDRMDVQLTKLTLYRTVIQPGIYHPDIQLLHP
INLEFLVNRNLAASWYHKVPVVEIKGHLDSMNVSLNQEDLNLLFRILTENLCEGTEDLDK
VKPRVQETGEIKEPLEISISQDVHDSKNTLTTGVEEIRSVDIINMLLNFEIKEVVVTLMK
KSEKKGRPLHELNVLQLGMEAKVKTYDMTAKAYLKKISMQCFDFTDSKGEPLHIINSSNV
TDEPLLKMLLTKADSDGPEFKTIHDSTKQRLKVSFASLDLVLHLEALLSFMDFLSSAAPF
SEPSSSEKESELKPLVGESRSIAVKAVSSNISQKDVFDLKITAELNAFNVFVCDQKCNIA
DIKIHGMDASISVKPKQTDVFARLKDIIVMNVDLQSIHKKAVSILGDEVFRFQLTLYPDA
TEGEAYADMSKVDGKLSFKVGCIQIVYVHKFFMSLLNFLNNFQTAKEALSTATVQAAERA
ASSMKDLAQKSFRLLMDINLKAPVIIIPQSSVSPNAVIADLGLIRVENKFSLVPMEHYSL
PPVIDKMNIELTQLKLSRTILQASLPQNDIEILKPVNMLLSIQRNLAAAWYVQIPGMEIK

GKLKPMQVALSEDDLTVLMKILLENLGEASSQPSPTQSVQETVRVRKVDVSSVPDHLKEQ
EDWTDSKLSMNQIVSLQFDFHFESLSIILYNNDINQESGVAFHNDSFQLGELRLHLMASS
GKMFKDGSMNVSVKLKTCTLDDLREGIERATSRMIDRKNDQDNNSSMIDISYKQDKNGSQ
IDAVLDKLYVCASVEFLMTVADFFIKAVPQSPENVAKETQILPRQTATGKVKIEKDDSVR
PNMTLKAMITDPEVVFVASLTKADAPALTASFQCNLSLSTSKLEQMMEASVRDLKVLACP
FLREKRGKNITTVLQPCSLFMEKCTWASGKQNINIMVKEFIIKISPIILNTVLTIMAALS
PKTKEDGSKDTSKEMENLWGIKSINDYNTWFLGVDTATEITESFKGIEHSLIEENCGVVV
ESIQVTLECGLGHRTVPLLLAESKFSGNIKNWTSLMAAVADVTLQVHYYNEIHAVWEPLI
ERVEGKRQWNLRLDVKKNPVQDKSLLPGDDFIPEPQMAIHISSGNTMNITISKSCLNVFN
NLAKGFSEGTASTFDYSLKDRAPFTVKNAVGVPIKVKPNCNLRVMGFPEKSDIFDVDAGQ
NLELEYASMVPSSQGNLSILSRQESSFFTLTIVPHGYTEVANIPVARPGRRLYNVRNPNA
SHSDSVLVQIDATEGNKVITLRSPLQIKNHFSIAFIIYKFVKNVKLLERIGIARPEEEFH
VPLDSYRCQLFIQPAGILEHQYKESTTYISWKEELHRSREVRCMLQCPSVEVSFLPLIVN
TVALPDELSYICTHGEDWDVAYIIHLYPSLTLRNLLPYSLRYLLEGTAETHELAEGSTAD
VLHSRISGEIMELVLVKYQGKNWNGHFRIRDTLPEFFPVCFSSDSTEVTTVDLSVHVRRI
GSRMVLSVFSPYWLINKTTRVLQYRSEDIHVKHPADFRDIILFSFKKKNIFTKNKVQLKI
STSAWSSSFSLDTVGSYGCVKCPANNMEYLVGVSIKMSSFNLSRIVTLTPFCTIANKSSL
ELEVGEIASDGSMPTNKWNYIASSECLPFWPESLSGKLCVRVVGCEGSSKPFFYNRQDNG
TLLSLEDLNGGILVDVNTAEHSTVITFSDYHEGSAPALIMNHTPWDILTYKQSGSPEEMV
LLPRQARLFAWADPTGTRKLTWTYAANVGEHDLLKDGCGQFPYDANIQIHWVSFLDGRQR
VLLFTDDVALVSKALQAEEMEQADYEITLSLHSLGLSLVNNESKQEVSYIGITSSGVVWE
VKPKQKWKPFSQKQIILLEQSYQKHQISRDHGWIKLDNNFEVNFDKDPMEMRLPIRSPIK
RDFLSGIQIEFKQSSHQRSLRARLYWLQVDNQLPGAMFPVVFHPVAPPKSIALDSEPKPF
IDVSVITRFNEYSKVLQFKYFMVLIQEMALKIDQGFLGAIIALFTPTTDPEAERRRTKLI
QQDIDALNAELMETSMTDMSILSFFEHFHISPVKLHLSLSLGSGGEESDKEKQEMFAVHS
VNLLLKSIGATLTDVDDLIFKLAYYEIRYQFYKRDQLIWSVVRHYSEQFLKQMYVLVLGL
DVLGNPFGLIRGLSEGVEALFYEPFQGAVQGPEEFAEGLVIGVRSLFGHTVGGAAGVVSR
ITGSVGKGLAAITMDKEYQQKRREELSRQPRDFGDSLARGGKGFLRGVVGGVTGIITKPV
EGAKKEGAAGFFKGIGKGLVGAVARPTGGIVDMASSTFQGIQRAAESTEEVSSLRPPRLI
HEDGIIRPYDRQESEGSDLLENHIKKLEGETYRYHCAIPGSKKTILMVTNRRVLCIKEVE
ILGLMCVDWQCPFEDFVFPPSVSENVLKISVKEQGLFHKKDSANQGCVRKVYLKDTATAE
RACNAIEDAQSTRQQQKLMKQSSVRLLRPQLPS
>sp|P49757|NUMB_HUMAN Protein numb homolog OS=Homo sapiens OX=9606 GN=NUMB PE=1 SV=2
MNKLRQSFRRKKDVYVPEASRPHQWQTDEEGVRTGKCSFPVKYLGHVEVDESRGMHICED
AVKRLKAERKFFKGFFGKTGKKAVKAVLWVSADGLRVVDEKTKDLIVDQTIEKVSFCAPD
RNFDRAFSYICRDGTTRRWICHCFMAVKDTGERLSHAVGCAFAACLERKQKREKECGVTA
TFDASRTTFTREGSFRVTTATEQAEREEIMKQMQDAKKAETDKIVVGSSVAPGNTAPSPS
SPTSPTSDATTSLEMNNPHAIPRRHAPIEQLARQGSFRGFPALSQKMSPFKRQLSLRINE
LPSTMQRKTDFPIKNAVPEVEGEAESISSLCSQITNAFSTPEDPFSSAPMTKPVTVVAPQ
SPTFQANGTDSAFHVLAKPAHTALAPVAMPVRETNPWAHAPDAANKEIAATCSGTEWGQS
SGAASPGLFQAGHRRTPSEADRWLEEVSKSVRAQQPQASAAPLQPVLQPPPPTAISQPAS
PFQGNAFLTSQPVPVGVVPALQPAFVPAQSYPVANGMPYPAPNVPVVGITPSQMVANVFG
TAGHPQAAHPHQSPSLVRQQTFPHYEASSATTSPFFKPPAQHLNGSAAFNGVDDGRLASA
DRHTEVPTGTCPVDPFEAQWAALENKSKQRTNPSPTNPFSSDLQKTFEIEL
>sp|A0A0B4J2D9|KVD13_HUMAN Immunoglobulin kappa variable 1D-13 OS=Homo sapiens OX=9606 GN=IGKV1D-13 PE=3 SV=1

MDMRVPAQLLGLLLLWLPGARCAIQLTQSPSSLSASVGDRVTITCRASQGISSALAWYQQ
KPGKAPKLLIYDASSLESGVPSRFSGSGSGTDFTLTISSLQPEDFATYYCQQFNSYP
>sp|P0DP09|KV113_HUMAN Immunoglobulin kappa variable 1-13 OS=Homo sapiens OX=9606 GN=IGKV1-13 PE=3 SV=1
MDMRVPAQLLGLLLLWLPGARCAIQLTQSPSSLSASVGDRVTITCRASQGISSALAWYQQ
KPGKAPKLLIYDASSLESGVPSRFSGSGSGTDFTLTISSLQPEDFATYYCQQFNSYP
>sp|P21128|ENDOU_HUMAN Uridylate-specific endoribonuclease OS=Homo sapiens OX=9606 GN=ENDOU PE=1 SV=2
MRACISLVLAVLCGLAWAGKIESCASRCNEKFNRDAACQCDRRCLWHGNCCEDYEHLCTE
DHKESEPLPQLEEETEEALASNLYSAPTSCQGRCYEAFDKHHQCHCNARCQEFGNCCKDF
ESLCSDHEVSHSSDAITKEEIQSISEKIYRADTNKAQKEDIVLNSQNCISPSETRNQVDR
CPKPLFTYVNEKLFSKPTYAAFINLLNNYQRATGHGEHFSAQELAEQDAFLREIMKTAVM
KELYSFLHHQNRYGSEQEFVDDLKNMWFGLYSRGNEEGDSSGFEHVFSGEVKKGKVTGFH
NWIRFYLEEKEGLVDYYSHIYDGPWDSYPDVLAMQFNWDGYYKEVGSAFIGSSPEFEFAL
YSLCFIARPGKVCQLSLGGYPLAVRTYTWDKSTYGNGKKYIATAYIVSST
>sp|P27449|VATL_HUMAN V-type proton ATPase 16 kDa proteolipid subunit c OS=Homo sapiens OX=9606 GN=ATP6V0C PE=1 SV=1
MSESKSGPEYASFFAVMGASAAMVFSALGAAYGTAKSGTGIAAMSVMRPEQIMKSIIPVV
MAGIIAIYGLVVAVLIANSLNDDISLYKSFLQLGAGLSVGLSGLAAGFAIGIVGDAGVRG
TAQQPRLFVGMILILIFAEVLGLYGLIVALILSTK
>sp|Q14643|ITPR1_HUMAN Inositol 1,4,5-trisphosphate receptor type 1 OS=Homo sapiens OX=9606 GN=ITPR1 PE=1 SV=3
MSDKMSSFLHIGDICSLYAEGSTNGFISTLGLVDDRCVVQPETGDLNNPPKKFRDCLFKL
CPMNRYSAQKQFWKAAKPGANSTTDAVLLNKLHHAADLEKKQNETENRKLLGTVIQYGNV
IQLLHLKSNKYLTVNKRLPALLEKNAMRVTLDEAGNEGSWFYIQPFYKLRSIGDSVVIGD
KVVLNPVNAGQPLHASSHQLVDNPGCNEVNSVNCNTSWKIVLFMKWSDNKDDILKGGDVV
RLFHAEQEKFLTCDEHRKKQHVFLRTTGRQSATSATSSKALWEVEVVQHDPCRGGAGYWN
SLFRFKHLATGHYLAAEVDPDFEEECLEFQPSVDPDQDASRSRLRNAQEKMVYSLVSVPE
GNDISSIFELDPTTLRGGDSLVPRNSYVRLRHLCTNTWVHSTNIPIDKEEEKPVMLKIGT
SPVKEDKEAFAIVPVSPAEVRDLDFANDASKVLGSIAGKLEKGTITQNERRSVTKLLEDL
VYFVTGGTNSGQDVLEVVFSKPNRERQKLMREQNILKQIFKLLQAPFTDCGDGPMLRLEE
LGDQRHAPFRHICRLCYRVLRHSQQDYRKNQEYIAKQFGFMQKQIGYDVLAEDTITALLH
NNRKLLEKHITAAEIDTFVSLVRKNREPRFLDYLSDLCVSMNKSIPVTQELICKAVLNPT
NADILIETKLVLSRFEFEGVSSTGENALEAGEDEEEVWLFWRDSNKEIRSKSVRELAQDA
KEGQKEDRDVLSYYRYQLNLFARMCLDRQYLAINEISGQLDVDLILRCMSDENLPYDLRA
SFCRLMLHMHVDRDPQEQVTPVKYARLWSEIPSEIAIDDYDSSGASKDEIKERFAQTMEF
VEEYLRDVVCQRFPFSDKEKNKLTFEVVNLARNLIYFGFYNFSDLLRLTKILLAILDCVH
VTTIFPISKMAKGEENKGNNDVEKLKSSNVMRSIHGVGELMTQVVLRGGGFLPMTPMAAA
PEGNVKQAEPEKEDIMVMDTKLKIIEILQFILNVRLDYRISCLLCIFKREFDESNSQTSE
TSSGNSSQEGPSNVPGALDFEHIEEQAEGIFGGSEENTPLDLDDHGGRTFLRVLLHLTMH
DYPPLVSGALQLLFRHFSQRQEVLQAFKQVQLLVTSQDVDNYKQIKQDLDQLRSIVEKSE
LWVYKGQGPDETMDGASGENEHKKTEEGNNKPQKHESTSSYNYRVVKEILIRLSKLCVQE
SASVRKSRKQQQRLLRNMGAHAVVLELLQIPYEKAEDTKMQEIMRLAHEFLQNFCAGNQQ
NQALLHKHINLFLNPGILEAVTMQHIFMNNFQLCSEINERVVQHFVHCIETHGRNVQYIK
FLQTIVKAEGKFIKKCQDMVMAELVNSGEDVLVFYNDRASFQTLIQMMRSERDRMDENSP
LMYHIHLVELLAVCTEGKNVYTEIKCNSLLPLDDIVRVVTHEDCIPEVKIAYINFLNHCY
VDTEVEMKEIYTSNHMWKLFENFLVDICRACNNTSDRKHADSILEKYVTEIVMSIVTTFF

SSPFSDQSTTLQTRQPVFVQLLQGVFRVYHCNWLMPSQKASVESCIRVLSDVAKSRAIAI
PVDLDSQVNNLFLKSHSIVQKTAMNWRLSARNAARRDSVLAASRDYRNIIERLQDIVSAL
EDRLRPLVQAELSVLVDVLHRPELLFPENTDARRKCESGGFICKLIKHTKQLLEENEEKL
CIKVLQTLREMMTKDRGYGEKLISIDELDNAELPPAPDSENATEELEPSPPLRQLEDHKR
GEALRQVLVNRYYGNVRPSGRRESLTSFGNGPLSAGGPGKPGGGGGGSGSSSMSRGEMSL
AEVQCHLDKEGASNLVIDLIMNASSDRVFHESILLAIALLEGGNTTIQHSFFCRLTEDKK
SEKFFKVFYDRMKVAQQEIKATVTVNTSDLGNKKKDDEVDRDAPSRKKAKEPTTQITEEV
RDQLLEASAATRKAFTTFRREADPDDHYQPGEGTQATADKAKDDLEMSAVITIMQPILRF
LQLLCENHNRDLQNFLRCQNNKTNYNLVCETLQFLDCICGSTTGGLGLLGLYINEKNVAL
INQTLESLTEYCQGPCHENQNCIATHESNGIDIITALILNDINPLGKKRMDLVLELKNNA
SKLLLAIMESRHDSENAERILYNMRPKELVEVIKKAYMQGEVEFEDGENGEDGAASPRNV
GHNIYILAHQLARHNKELQSMLKPGGQVDGDEALEFYAKHTAQIEIVRLDRTMEQIVFPV
PSICEFLTKESKLRIYYTTERDEQGSKINDFFLRSEDLFNEMNWQKKLRAQPVLYWCARN
MSFWSSISFNLAVLMNLLVAFFYPFKGVRGGTLEPHWSGLLWTAMLISLAIVIALPKPHG
IRALIASTILRLIFSVGLQPTLFLLGAFNVCNKIIFLMSFVGNCGTFTRGYRAMVLDVEF
LYHLLYLVICAMGLFVHEFFYSLLLFDLVYREETLLNVIKSVTRNGRSIILTAVLALILV
YLFSIVGYLFFKDDFILEVDRLPNETAVPETGESLASEFLFSDVCRVESGENCSSPAPRE
ELVPAEETEQDKEHTCETLLMCIVTVLSHGLRSGGGVGDVLRKPSKEEPLFAARVIYDLL
FFFMVIIIVLNLIFGVIIDTFADLRSEKQKKEEILKTTCFICGLERDKFDNKTVTFEEHI
KEEHNMWHYLCFIVLVKVKDSTEYTGPESYVAEMIKERNLDWFPRMRAMSLVSSDSEGEQ
NELRNLQEKLESTMKLVTNLSGQLSELKDQMTEQRKQKQRIGLLGHPPHMNVNPQQPA
>sp|P04053|TDT_HUMAN DNA nucleotidylexotransferase OS=Homo sapiens OX=9606 GN=DNTT PE=1 SV=3
MDPPRASHLSPRKKRPRQTGALMASSPQDIKFQDLVVFILEKKMGTTRRAFLMELARRKG
FRVENELSDSVTHIVAENNSGSDVLEWLQAQKVQVSSQPELLDVSWLIECIRAGKPVEMT
GKHQLVVRRDYSDSTNPGPPKTPPIAVQKISQYACQRRTTLNNCNQIFTDAFDILAENCE
FRENEDSCVTFMRAASVLKSLPFTIISMKDTEGIPCLGSKVKGIIEEIIEDGESSEVKAV
LNDERYQSFKLFTSVFGVGLKTSEKWFRMGFRTLSKVRSDKSLKFTRMQKAGFLYYEDLV
SCVTRAEAEAVSVLVKEAVWAFLPDAFVTMTGGFRRGKKMGHDVDFLITSPGSTEDEEQL
LQKVMNLWEKKGLLLYYDLVESTFEKLRLPSRKVDALDHFQKCFLIFKLPRQRVDSDQSS
WQEGKTWKAIRVDLVLCPYERRAFALLGWTGSRQFERDLRRYATHERKMILDNHALYDKT
KRIFLKAESEEEIFAHLGLDYIEPWERNA
>sp|O94760|DDAH1_HUMAN N(G),N(G)-dimethylarginine dimethylaminohydrolase 1 OS=Homo sapiens OX=9606 GN=DDAH1 PE=1 SV=3
MAGLGHPAAFGRATHAVVRALPESLGQHALRSAKGEEVDVARAERQHQLYVGVLGSKLGL
QVVELPADESLPDCVFVEDVAVVCEETALITRPGAPSRRKEVDMMKEALEKLQLNIVEMK
DENATLDGGDVLFTGREFFVGLSKRTNQRGAEILADTFKDYAVSTVPVADGLHLKSFCSM
AGPNLIAIGSSESAQKALKIMQQMSDHRYDKLTVPDDIAANCIYLNIPNKGHVLLHRTPE
EYPESAKVYEKLKDHMLIPVSMSELEKVDGLLTCCSVLINKKVDS
>sp|A6NGW2|STRCL_HUMAN Putative stereocilin-like protein OS=Homo sapiens OX=9606 GN=STRCP1 PE=5 SV=1
MALSLWPLLLLLLLLLLLSFAVTLAPTGPHSLDPGLSFLKSLLSTLDQAPQGSLSRSRFF
TFLANISSSFEPGRMGEGPVGEPPPLQPPALRLHDFLVTLRGSPDWEPMLGLLGDMLALL
GQEQTPRDFLVHQAGVLGGLVEVLLGALVPGGPPTPTQPPCTRDGPSDCVLAADWLPSLL
LLLEGTRWQALVQVQPSVDPTNATGLDGREAAPHFLQGLLGLLTPTGELGSKEALWGGLL
RTVGAPLYAAFQEGLLRVTHSLQDEVFSILGQPEPDTNGQCQGGNLQQLLLWGVRHNLSW

DVQALGFLSGSPPPPPALLHCLSTGVPLPRASQPSAHISPRQRRAITVEALCENHLGPAP
PYSISNFSIHLLCQHTKPATPQPHPSTTAICQTAVWYAVSWAPGAQGWLQACHDQFPDEF
LDAICSNLSFSALSGSNRRLVKRLCAGLLPPPTSCPEGLPPVPLTPDIFWGCFLENETLW
AERLCGEASLQAVPPSNQAWVQHVCQGPTPDVTASPPCHIGPCGERCPDGGSFLVMVCAN
DTMYEVLVPFWPWLAGQCRISRGGNDTCFLEGLLGPLLPSLPPLGPSPLCLTPGPFLLGM
LSQLPRCQSSVPALAHPTRLHYLLRLLTFLLGPGAGGAEAQGMLGRALLLSSLPDNCSFW
DAFRPEGRRSVLRTIGEYLEQDEEQPTPSGFEPTVNPSSGISKMELLACFSPVLWDLLQR
EKSVWALQILVQAYLHMPPENLQQLVLSAEREAAQGFLTLMLQGKLQGKLQVPPSEEQAL
GRLTALLLQRYPRLTSQLFIDLSPLIPFLAVSDLMRFPPSLLANDSVLAAIRDYSPGMRP
EQKEALAKRLLAPELFGEVPAWPQELLWAVLPLLPHLPLENFLQLSPHQIQALEDSWPAA
GLGPGHARHVLRSLVNQSVQDGEEQVRRLGPLACFLSPEELQSLVPLSDPTGPVERGLLE
CAANGTLSPEGRVAYELLGVLRSSGGAVLSPRELRVWAPLFSQLGLRFLQELSEPQLRAM
LPVLQGTSVTPAQAVLLLGRLLPRHDLSLEELCSLHLLLPGLSPQTLQAIPRRVLVGACS
CLAPELSRLSACQTAALLQTFRVKDGVKNMGTTGAGPAVCIPGQQPIPTTWPDCLLPLLP
LKLLQLDSLALLANRRRYWELPWSEQQAQFLWKKMQVPTNLTLRNLQALGTLAGGMSCEF
LQQINSMVDFLEVVHMIYQLPTRVRGSLRACIWAELQRRMAMPEPEWTTVGPELNGLDSK
LLLDLPIQLMDRLSNESIMLVVELVQRAPEQLLALTPLHQAALAERALQNLAPKETPVSG
EVLETLGPLVGFLGTESTRQIPLQILLSHLSFCLGETFATELGWLLLQESVLGKPELWSQ
DEVEQAGRLVFTLSTEAISLIPREALGPETLERLLEKQQSWEQSRVGQLCRGPQLAAKKA
ALVAGVVRPAAEDLPEPVPNCADVRGTFPAACSATQIAEMELSDFKDCLTLFAGDPGLGP
EEPRAAMGKAKWLWGPPRGFGPEQILQLGRLLIGLGDQELQELILVDWGVLSTLGQIDGW
SSTQLRIVVSSFLRQSGRHVSHLDFVHLTALGYTLCGLRPEELQHISSWEFSQAALFLGT
LHLQCSEEQLEFLAHLFVLPGGFGPISNWGPEIFTEIGTIAAGIPDLALSALLRGQIQGV
TPLAISVIPPPKFAVVFSPIQLSSLASAQAVAVTPEQMAFLSPEQRRAVAWAQHEGKESP
EQQGRSTAWGLQDWSRPSWSLVLTISFLGHLL
>sp|Q7RTU9|STRC_HUMAN Stereocilin OS=Homo sapiens OX=9606 GN=STRC PE=2 SV=1
MALSLWPLLLLLLLLLLLSFAVTLAPTGPHSLDPGLSFLKSLLSTLDQAPQGSLSRSRFF
TFLANISSSFEPGRMGEGPVGEPPPLQPPALRLHDFLVTLRGSPDWEPMLGLLGDMLALL
GQEQTPRDFLVHQAGVLGGLVEVLLGALVPGGPPTPTRPPCTRDGPSDCVLAADWLPSLL
LLLEGTRWQALVQVQPSVDPTNATGLDGREAAPHFLQGLLGLLTPTGELGSKEALWGGLL
RTVGAPLYAAFQEGLLRVTHSLQDEVFSILGQPEPDTNGQCQGGNLQQLLLWGVRHNLSW
DVQALGFLSGSPPPPPALLHCLSTGVPLPRASQPSAHISPRQRRAITVEALCENHLGPAP
PYSISNFSIHLLCQHTKPATPQPHPSTTAICQTAVWYAVSWAPGAQGWLQACHDQFPDEF
LDAICSNLSFSALSGSNRRLVKRLCAGLLPPPTSCPEGLPPVPLTPDIFWGCFLENETLW
AERLCGEASLQAVPPSNQAWVQHVCQGPTPDVTASPPCHIGPCGERCPDGGSFLVMVCAN
DTMYEVLVPFWPWLAGQCRISRGGNDTCFLEGLLGPLLPSLPPLGPSPLCLTPGPFLLGM
LSQLPRCQSSVPALAHPTRLHYLLRLLTFLLGPGAGGAEAQGMLGRALLLSSLPDNCSFW
DAFRPEGRRSVLRTIGEYLEQDEEQPTPSGFEPTVNPSSGISKMELLACFSPVLWDLLQR
EKSVWALQILVQAYLHMPPENLQQLVLSAEREAAQGFLTLMLQGKLQGKLQVPPSEEQAL
GRLTALLLQRYPRLTSQLFIDLSPLIPFLAVSDLMRFPPSLLANDSVLAAIRDYSPGMRP
EQKEALAKRLLAPELFGEVPAWPQELLWAVLPLLPHLPLENFLQLSPHQIQALEDSWPAA
GLGPGHARHVLRSLVNQSVQDGEEQVRRLGPLACFLSPEELQSLVPLSDPTGPVERGLLE
CAANGTLSPEGRVAYELLGVLRSSGGAVLSPRELRVWAPLFSQLGLRFLQELSEPQLRAM
LPVLQGTSVTPAQAVLLLGRLLPRHDLSLEELCSLHLLLPGLSPQTLQAIPRRVLVGACS
CLAPELSRLSACQTAALLQTFRVKDGVKNMGTTGAGPAVCIPGQPIPTTWPDCLLPLLPL
KLLQLDSLALLANRRRYWELPWSEQQAQFLWKKMQVPTNLTLRNLQALGTLAGGMSCEFL

QQINSMVDFLEVVHMIYQLPTRVRGSLRACIWAELQRRMAMPEPEWTTVGPELNGLDSKL
LLDLPIQLMDRLSNESIMLVVELVQRAPEQLLALTPLHQAALAERALQNLAPKETPVSGE
VLETLGPLVGFLGTESTRQIPLQILLSHLSQLQGFCLGETFATELGWLLLQESVLGKPEL
WSQDEVEQAGRLVFTLSTEAISLIPREALGPETLERLLEKQQSWEQSRVGQLCREPQLAA
KKAALVAGVVRPAAEDLPEPVPNCADVRGTFPAAWSATQIAEMELSDFEDCLTLFAGDPG
LGPEELRAAMGKAKQLWGPPRGFRPEQILQLGRLLIGLGDRELQELILVDWGVLSTLGQI
DGWSTTQLRIVVSSFLRQSGRHVSHLDFVHLTALGYTLCGLRPEELQHISSWEFSQAALF
LGTLHLQCSEEQLEVLAHLLVLPGGFGPISNWGPEIFTEIGTIAAGIPDLALSALLRGQI
QGVTPLAISVIPPPKFAVVFSPIQLSSLTSAQAVAVTPEQMAFLSPEQRRAVAWAQHEGK
ESPEQQGRSTAWGLQDWSRPSWSLVLTISFLGHLL
>sp|Q03164|KMT2A_HUMAN Histone-lysine N-methyltransferase 2A OS=Homo sapiens OX=9606 GN=KMT2A PE=1 SV=5
MAHSCRWRFPARPGTTGGGGGGGRRGLGGAPRQRVPALLLPPGPPVGGGGPGAPPSPPAV
AAAAAAAGSSGAGVPGGAAAASAASSSSASSSSSSSSSASSGPALLRVGPGFDAALQVSA
AIGTNLRRFRAVFGESGGGGGSGEDEQFLGFGSDEEVRVRSPTRSPSVKTSPRKPRGRPR
SGSDRNSAILSDPSVFSPLNKSETKSGDKIKKKDSKSIEKKRGRPPTFPGVKIKITHGKD
ISELPKGNKEDSLKKIKRTPSATFQQATKIKKLRAGKLSPLKSKFKTGKLQIGRKGVQIV
RRRGRPPSTERIKTPSGLLINSELEKPQKVRKDKEGTPPLTKEDKTVVRQSPRRIKPVRI
IPSSKRTDATIAKQLLQRAKKGAQKKIEKEAAQLQGRKVKTQVKNIRQFIMPVVSAISSR
IIKTPRRFIEDEDYDPPIKIARLESTPNSRFSAPSCGSSEKSSAASQHSSQMSSDSSRSS
SPSVDTSTDSQASEEIQVLPEERSDTPEVHPPLPISQSPENESNDRRSRRYSVSERSFGS
RTTKKLSTLQSAPQQQTSSSPPPPLLTPPPPLQPASSISDHTPWLMPPTIPLASPFLPAS
TAPMQGKRKSILREPTFRWTSLKHSRSEPQYFSSAKYAKEGLIRKPIFDNFRPPPLTPED
VGFASGFSASGTAASARLFSPLHSGTRFDMHKRSPLLRAPRFTPSEAHSRIFESVTLPSN
RTSAGTSSSGVSNRKRKRKVFSPIRSEPRSPSHSMRTRSGRLSSSELSPLTPPSSVSSSL
SISVSPLATSALNPTFTFPSHSLTQSGESAEKNQRPRKQTSAPAEPFSSSSPTPLFPWFT
PGSQTERGRNKDKAPEELSKDRDADKSVEKDKSRERDREREKENKRESRKEKRKKGSEIQ
SSSALYPVGRVSKEKVVGEDVATSSSAKKATGRKKSSSHDSGTDITSVTLGDTTAVKTKI
LIKKGRGNLEKTNLDLGPTAPSLEKEKTLCLSTPSSSTVKHSTSSIGSMLAQADKLPMTD
KRVASLLKKAKAQLCKIEKSKSLKQTDQPKAQGQESDSSETSVRGPRIKHVCRRAAVALG
RKRAVFPDDMPTLSALPWEEREKILSSMGNDDKSSIAGSEDAEPLAPPIKPIKPVTRNKA
PQEPPVKKGRRSRRCGQCPGCQVPEDCGVCTNCLDKPKFGGRNIKKQCCKMRKCQNLQWM
PSKAYLQKQAKAVKKKEKKSKTSEKKDSKESSVVKNVVDSSQKPTPSAREDPAPKKSSSE
PPPRKPVEEKSEEGNVSAPGPESKQATTPASRKSSKQVSQPALVIPPQPPTTGPPRKEVP
KTTPSEPKKKQPPPPESGPEQSKQKKVAPRPSIPVKQKPKEKEKPPPVNKQENAGTLNIL
STLSNGNSSKQKIPADGVHRIRVDFKEDCEAENVWEMGGLGILTSVPITPRVVCFLCASS
GHVEFVYCQVCCEPFHKFCLEENERPLEDQLENWCCRRCKFCHVCGRQHQATKQLLECNK
CRNSYHPECLGPNYPTKPTKKKKVWICTKCVRCKSCGSTTPGKGWDAQWSHDFSLCHDCA
KLFAKGNFCPLCDKCYDDDDYESKMMQCGKCDRWVHSKCENLSDEMYEILSNLPESVAYT
CVNCTERHPAEWRLALEKELQISLKQVLTALLNSRTTSHLLRYRQAAKPPDLNPETEESI
PSRSSPEGPDPPVLTEVSKQDDQQPLDLEGVKRKMDQGNYTSVLEFSDDIVKIIQAAINS
DGGQPEIKKANSMVKSFFIRQMERVFPWFSVKKSRFWEPNKVSSNSGMLPNAVLPPSLDH
NYAQWQEREENSHTEQPPLMKKIIPAPKPKGPGEPDSPTPLHPPTPPILSTDRSREDSPE
LNPPPGIEDNRQCALCLTYGDDSANDAGRLLYIGQNEWTHVNCALWSAEVFEDDDGSLKN
VHMAVIRGKQLRCEFCQKPGATVGCCLTSCTSNYHFMCSRAKNCVFLDDKKVYCQRHRDL
IKGEVVPENGFEVFRRVFVDFEGISLRRKFLNGLEPENIHMMIGSMTIDCLGILNDLSDC
EDKLFPIGYQCSRVYWSTTDARKRCVYTCKIVECRPPVVEPDINSTVEHDENRTIAHSPT

SFTESSSKESQNTAEIISPPSPDRPPHSQTSGSCYYHVISKVPRIRTPSYSPTQRSPGCR
PLPSAGSPTPTTHEIVTVGDPLLSSGLRSIGSRRHSTSSLSPQRSKLRIMSPMRTGNTYS
RNNVSSVSTTGTATDLESSAKVVDHVLGPLNSSTSLGQNTSTSSNLQRTVVTVGNKNSHL
DGSSSSEMKQSSASDLVSKSSSLKGEKTKVLSSKSSEGSAHNVAYPGIPKLAPQVHNTTS
RELNVSKIGSFAEPSSVSFSSKEALSFPHLHLRGQRNDRDQHTDSTQSANSSPDEDTEVK
TLKLSGMSNRSSIINEHMGSSSRDRRQKGKKSCKETFKEKHSSKSFLEPGQVTTGEEGNL
KPEFMDEVLTPEYMGQRPCNNVSSDKIGDKGLSMPGVPKAPPMQVEGSAKELQAPRKRTV
KVTLTPLKMENESQSKNALKESSPASPLQIESTSPTEPISASENPGDGPVAQPSPNNTSC
QDSQSNNYQNLPVQDRNLMLPDGPKPQEDGSFKRRYPRRSARARSNMFFGLTPLYGVRSY
GEEDIPFYSSSTGKKRGKRSAEGQVDGADDLSTSDEDDLYYYNFTRTVISSGGEERLASH
NLFREEEQCDLPKISQLDGVDDGTESDTSVTATTRKSSQIPKRNGKENGTENLKIDRPED
AGEKEHVTKSSVGHKNEPKMDNCHSVSRVKTQGQDSLEAQLSSLESSRRVHTSTPSDKNL
LDTYNTELLKSDSDNNNSDDCGNILPSDIMDFVLKNTPSMQALGESPESSSSELLNLGEG
LGLDSNREKDMGLFEVFSQQLPTTEPVDSSVSSSISAEEQFELPLELPSDLSVLTTRSPT
VPSQNPSRLAVISDSGEKRVTITEKSVASSESDPALLSPGVDPTPEGHMTPDHFIQGHMD
ADHISSPPCGSVEQGHGNNQDLTRNSSTPGLQVPVSPTVPIQNQKYVPNSTDSPGPSQIS
NAAVQTTPPHLKPATEKLIVVNQNMQPLYVLQTLPNGVTQKIQLTSSVSSTPSVMETNTS
VLGPMGGGLTLTTGLNPSLPTSQSLFPSASKGLLPMSHHQHLHSFPAATQSSFPPNISNP
PSGLLIGVQPPPDPQLLVSESSQRTDLSTTVATPSSGLKKRPISRLQTRKNKKLAPSSTP
SNIAPSDVVSNMTLINFTPSQLPNHPSLLDLGSLNTSSHRTVPNIIKRSKSSIMYFEPAP
LLPQSVGGTAATAAGTSTISQDTSHLTSGSVSGLASSSSVLNVVSMQTTTTPTSSASVPG
HVTLTNPRLLGTPDIGSISNLLIKASQQSLGIQDQPVALPPSSGMFPQLGTSQTPSTAAI
TAASSICVLPSTQTTGITAASPSGEADEHYQLQHVNQLLASKTGIHSSQRDLDSASGPQV
SNFTQTVDAPNSMGLEQNKALSSAVQASPTSPGGSPSSPSSGQRSASPSVPGPTKPKPKT
KRFQLPLDKGNGKKHKVSHLRTSSSEAHIPDQETTSLTSGTGTPGAEAEQQDTASVEQSS
QKECGQPAGQVAVLPEVQVTQNPANEQESAEPKTVEEEESNFSSPLMLWLQQEQKRKESI
TEKKPKKGLVFEISSDDGFQICAESIEDAWKSLTDKVQEARSNARLKQLSFAGVNGLRML
GILHDAVVFLIEQLSGAKHCRNYKFRFHKPEEANEPPLNPHGSARAEVHLRKSAFDMFNF
LASKHRQPPEYNPNDEEEEEVQLKSARRATSMDLPMPMRFRHLKKTSKEAVGVYRSPIHG
RGLFCKRNIDAGEMVIEYAGNVIRSIQTDKREKYYDSKGIGCYMFRIDDSEVVDATMHGN
AARFINHSCEPNCYSRVINIDGQKHIVIFAMRKIYRGEELTYDYKFPIEDASNKLPCNCG
AKKCRKFLN
>sp|Q92616|GCN1_HUMAN eIF-2-alpha kinase activator GCN1 OS=Homo sapiens OX=9606 GN=GCN1 PE=1 SV=7
MAADTQVSETLKRFAGKVTTASVKERREILSELGKCVAGKDLPEGAVKGLCKLFCLTLHR
YRDAASRRALQAAIQQLAEAQPEATAKNLLHSLQSSGIGSKAGVPSKSSGSAALLALTWT
CLLVRIVFPSRAKRQGDIWNKLVEVQCLLLLEVLGGSHKHAVDGAVKKLTKLWKENPGLV
EQYLSAILSLEPNQNYAGMLGLLVQFCTSHKEMDVVSQHKSALLDFYMKNILMSKVKPPK
YLLDSCAPLLRYLSHSEFKDLILPTIQKSLLRSPENVIETISSLLASVTLDLSQYAMDIV
KGLAGHLKSNSPRLMDEAVLALRNLARQCSDSSAMESLTKHLFAILGGSEGKLTVVAQKM
SVLSGIGSVSHHVVSGPSSQVLNGIVAELFIPFLQQEVHEGTLVHAVSVLALWCNRFTME
VPKKLTEWFKKAFSLKTSTSAVRHAYLQCMLASYRGDTLLQALDLLPLLIQTVEKAASQS
TQVPTITEGVAAALLLLKLSVADSQAEAKLSSFWQLIVDEKKQVFTSEKFLVMASEDALC
TVLHLTERLFLDHPHRLTGNKVQQYHRALVAVLLSRTWHVRRQAQQTVRKLLSSLGGFKL
AHGLLEELKTVLSSHKVLPLEALVTDAGEVTEAGKAYVPPRVLQEALCVISGVPGLKGDV
TDTEQLAQEMLIISHHPSLVAVQSGLWPALLARMKIDPEAFITRHLDQIIPRMTTQSPLN
QSSMNAMGSLSVLSPDRVLPQLISTITASVQNPALRLVTREEFAIMQTPAGELYDKSIIQ

SAQQDSIKKANMKRENKAYSFKEQIIELELKEEIKKKKGIKEEVQLTSKQKEMLQAQLDR
EAQVRRRLQELDGELEAALGLLDIILAKNPSGLTQYIPVLVDSFLPLLKSPLAAPRIKNP
FLSLAACVMPSRLKALGTLVSHVTLRLLKPECVLDKSWCQEELSVAVKRAVMLLHTHTIT
SRVGKGEPGAAPLSAPAFSLVFPFLKMVLTEMPHHSEEEEEWMAQILQILTVQAQLRASP
NTPPGRVDENGPELLPRVAMLRLLTWVIGTGSPRLQVLASDTLTTLCASSSGDDGCAFAE
QEEVDVLLCALQSPCASVRETVLRGLMELHMVLPAPDTDEKNGLNLLRRLWVVKFDKEEE
IRKLAERLWSMMGLDLQPDLCSLLIDDVIYHEAAVRQAGAEALSQAVARYQRQAAEVMGR
LMEIYQEKLYRPPPVLDALGRVISESPPDQWEARCGLALALNKLSQYLDSSQVKPLFQFF
VPDALNDRHPDVRKCMLDAALATLNTHGKENVNSLLPVFEEFLKNAPNDASYDAVRQSVV
VLMGSLAKHLDKSDPKVKPIVAKLIAALSTPSQQVQESVASCLPPLVPAIKEDAGGMIQR
LMQQLLESDKYAERKGAAYGLAGLVKGLGILSLKQQEMMAALTDAIQDKKNFRRREGALF
AFEMLCTMLGKLFEPYVVHVLPHLLLCFGDGNQYVREAADDCAKAVMSNLSAHGVKLVLP
SLLAALEEESWRTKAGSVELLGAMAYCAPKQLSSCLPNIVPKLTEVLTDSHVKVQKAGQQ
ALRQIGSVIRNPEILAIAPVLLDALTDPSRKTQKCLQTLLDTKFVHFIDAPSLALIMPIV
QRAFQDRSTDTRKMAAQIIGNMYSLTDQKDLAPYLPSVTPGLKASLLDPVPEVRTVSAKA
LGAMVKGMGESCFEDLLPWLMETLTYEQSSVDRSGAAQGLAEVMAGLGVEKLEKLMPEIV
ATASKVDIAPHVRDGYIMMFNYLPITFGDKFTPYVGPIIPCILKALADENEFVRDTALRA
GQRVISMYAETAIALLLPQLEQGLFDDLWRIRFSSVQLLGDLLFHISGVTGKMTTETASE
DDNFGTAQSNKAIITALGVERRNRVLAGLYMGRSDTQLVVRQASLHVWKIVVSNTPRTLR
EILPTLFGLLLGFLASTCADKRTIAARTLGDLVRKLGEKILPEIIPILEEGLRSQKSDER
QGVCIGLSEIMKSTSRDAVLYFSESLVPTARKALCDPLEEVREAAAKTFEQLHSTIGHQA
LEDILPFLLKQLDDEEVSEFALDGLKQVMAIKSRVVLPYLVPKLTTPPVNTRVLAFLSSV
AGDALTRHLGVILPAVMLALKEKLGTPDEQLEMANCQAVILSVEDDTGHRIIIEDLLEAT
RSPEVGMRQAAAIILNIYCSRSKADYTSHLRSLVSGLIRLFNDSSPVVLEESWDALNAIT
KKLDAGNQLALIEELHKEIRLIGNESKGEHVPGFCLPKKGVTSILPVLREGVLTGSPEQK
EEAAKALGLVIRLTSADALRPSVVSITGPLIRILGDRFSWNVKAALLETLSLLLAKVGIA
LKPFLPQLQTTFTKALQDSNRGVRLKAADALGKLISIHIKVDPLFTELLNGIRAMEDPGV
RDTMLQALRFVIQGAGAKVDAVIRKNIVSLLLSMLGHDEDNTRISSAGCLGELCAFLTEE
ELSAVLQQCLLADVSGIDWMVRHGRSLALSVAVNVAPGRLCAGRYSSDVQEMILSSATAD
RIPIAVSGVRGMGFLMRHHIETGGGQLPAKLSSLFVKCLQNPSSDIRLVAEKMIWWANKD
PLPPLDPQAIKPILKALLDNTKDKNTVVRAYSDQAIVNLLKMRQGEEVFQSLSKILDVAS
LEVLNEVNRRSLKKLASQADSTEQVDDTILT
>sp|Q12802|AKP13_HUMAN A-kinase anchor protein 13 OS=Homo sapiens
OX=9606 GN=AKAP13 PE=1 SV=2
MKLNPQQAPLYGDCVVTVLLAEEDKAEDDVVFYLVFLGSTLRHCTSTRKVSSDTLETIAP
GHDCCETVKVQLCASKEGLPVFVVAEEDFHFVQDEAYDAAQFLATSAGNQQALNFTRFLD
QSGPPSGDVNSLDKKLVLAFRHLKLPTEWNVLGTDQSLHDAGPRETLMHFAVRLGLLRLT
WFLLQKPGGRGALSIHNQEGATPVSLALERGYHKLHQLLTEENAGEPDSWSSLSYEIPYG
DCSVRHHRELDIYTLTSESDSHHEHPFPGDGCTGPIFKLMNIQQQLMKTNLKQMDSLMPL
MMTAQDPSSAPETDGQFLPCAPEPTDPQRLSSSEETESTQCCPGSPVAQTESPCDLSSIV
EEENTDRSCRKKNKGVERKGEEVEPAPIVDSGTVSDQDSCLQSLPDCGVKGTEGLSSCGN
RNEETGTKSSGMPTDQESLSSGDAVLQRDLVMEPGTAQYSSGGELGGISTTNVSTPDTAG
EMEHGLMNPDATVWKNVLQGGESTKERFENSNIGTAGASDVHVTSKPVDKISVPNCAPAA
SSLDGNKPAESSLAFSNEETSTEKTAETETSRSREESADAPVDQNSVVIPAAAKDKISDG
LEPYTLLAAGIGEAMSPSDLALLGLEEDVMPHQNSETNSSHAQSQKGKSSPICSTTGDDK
LCADSACQQNTVTSSGDLVAKLCDNIVSESESTTARQPSSQDPPDASHCEDPQAHTVTSD
PVRDTQERADFCPFKVVDNKGQRKDVKLDKPLTNMLEVVSHPHPVVPKMEKELVPDQAVI

SDSTFSLANSPGSESVTKDDALSFVPSQKEKGTATPELHTATDYRDGPDGNSNEPDTRPL
EDRAVGLSTSSTAAELQHGMGNTSLTGLGGEHEGPAPPAIPEALNIKGNTDSSLQSVGKA
TLALDSVLTEEGKLLVVSESSAAQEQDKDKAVTCSSIKENALSSGTLQEEQRTPPPGQDT
QQFHEKSISADCAKDKALQLSNSPGASSAFLKAETEHNKEVAPQVSLLTQGGAAQSLVPP
GASLATESRQEALGAEHNSSALLPCLLPDGSDGSDALNCSQPSPLDVGVKNTQSQGKTSA
CEVSGDVTVDVTGVNALQGMAEPRRENISHNTQDILIPNVLLSQEKNAVLGLPVALQDKA
VTDPQGVGTPEMIPLDWEKGKLEGADHSCTMGDAEEAQIDDEAHPVLLQPVAKELPTDME
LSAHDDGAPAGVREVMRAPPSGRERSTPSLPCMVSAQDAPLPKGADLIEEAASRIVDAVI
EQVKAAGALLTEGEACHMSLSSPELGPLTKGLESAFTEKVSTFPPGESLPMGSTPEEATG
SLAGCFAGREEPEKIILPVQGPEPAAEMPDVKAEDEVDFRASSISEEVAVGSIAATLKMK
QGPMTQAINRENWCTIEPCPDAASLLASKQSPECENFLDVGLGRECTSKQGVLKRESGSD
SDLFHSPSDDMDSIIFPKPEEEHLACDITGSSSSTDDTASLDRHSSHGSDVSLSQILKPN
RSRDRQSLDGFYSHGMGAEGRESESEPADPGDVEEEEMDSITEVPANCSVLRSSMRSLSP
FRRHSWGPGKNAASDAEMNHRSSMRVLGDVVRRPPIHRRSFSLEGLTGGAGVGNKPSSSL
EVSSANAEELRHPFSGEERVDSLVSLSEEDLESDQREHRMFDQQICHRSKQQGFNYCTSA
ISSPLTKSISLMTISHPGLDNSRPFHSTFHNTSANLTESITEENYNFLPHSPSKKDSEWK
SGTKVSRTFSYIKNKMSSSKKSKEKEKEKDKIKEKEKDSKDKEKDKKTVNGHTFSSIPVV
GPISCSQCMKPFTNKDAYTCANCSAFVHKGCRESLASCAKVKMKQPKGSLQAHDTSSLPT
VIMRNKPSQPKERPRSAVLLVDETATTPIFANRRSQQSVSLSKSVSIQNITGVGNDENMS
NTWKFLSHSTDSLNKISKVNESTESLTDEGVGTDMNEGQLLGDFEIESKQLEAESWSRII
DSKFLKQQKKDVVKRQEVIYELMQTEFHHVRTLKIMSGVYSQGMMADLLFEQQMVEKLFP
CLDELISIHSQFFQRILERKKESLVDKSEKNFLIKRIGDVLVNQFSGENAERLKKTYGKF
CGQHNQSVNYFKDLYAKDKRFQAFVKKKMSSSVVRRLGIPECILLVTQRITKYPVLFQRI
LQCTKDNEVEQEDLAQSLSLVKDVIGAVDSKVASYEKKVRLNEIYTKTDSKSIMRMKSGQ
MFAKEDLKRKKLVRDGSVFLKNAAGRLKEVQAVLLTDILVFLQEKDQKYIFASLDQKSTV
ISLKKLIVREVAHEEKGLFLISMGMTDPEMVEVHASSKEERNSWIQIIQDTINTLNRDED
EGIPSENEEEKKMLDTRARELKEQLHQKDQKILLLLEEKEMIFRDMAECSTPLPEDCSPT
HSPRVLFRSNTEEALKGGPLMKSAINEVEILQGLVSGNLGGTLGPTVSSPIEQDVVGPVS
LPRRAETFGGFDSHQMNASKGGEKEEGDDGQDLRRTESDSGLKKGGNANLVFMLKRNSEQ
VVQSVVHLYELLSALQGVVLQQDSYIEDQKLVLSERALTRSLSRPSSLIEQEKQRSLEKQ
RQDLANLQKQQAQYLEEKRRREREWEARERELREREALLAQREEEVQQGQQDLEKEREEL
QQKKGTYQYDLERLRAAQKQLEREQEQLRREAERLSQRQTERDLCQVSHPHTKLMRIPSF
FPSPEEPPSPSAPSIAKSGSLDSELSVSPKRNSISRTHKDKGPFHILSSTSQTNKGPEGQ
SQAPASTSASTRLFGLTKPKEKKEKKKKNKTSRSQPGDGPASEVSAEGEEIFC
>sp|Q96BZ9|TBC20_HUMAN TBC1 domain family member 20 OS=Homo sapiens OX=9606 GN=TBC1D20 PE=1 SV=1
MALRSAQGDGPTSGHWDGGAEKADFNAKRKKKVAEIHQALNSDPTDVAALRRMAISEGGL
LTDEIRRKVWPKLLNVNANDPPPISGKNLRQMSKDYQQVLLDVRRSLRRFPPGMPEEQRE
GLQEELIDIILLILERNPQLHYYQGYHDIVVTFLLVVGERLATSLVEKLSTHHLRDFMDP
TMDNTKHILNYLMPIIDQVNPELHDFMQSAEVGTIFALSWLITWFGHVLSDFRHVVRLYD
FFLACHPLMPIYFAAVIVLYREQEVLDCDCDMASVHHLLSQIPQDLPYETLISRAGDLFV
QFPPSELAREAAAQQQAERTAASTFKDFELASAQQRPDMVLRQRFRGLLRPEDRTKDVLT
KPRTNRFVKLAVMGLTVALGAAALAVVKSALEWAPKFQLQLFP
>sp|P29590|PML_HUMAN Protein PML OS=Homo sapiens OX=9606 GN=PML PE=1 SV=3
MEPAPARSPRPQQDPARPQEPTMPPPETPSEGRQPSPSPSPTERAPASEEEFQFLRCQQC
QAEAKCPKLLPCLHTLCSGCLEASGMQCPICQAPWPLGADTPALDNVFFESLQRRLSVYR

QIVDAQAVCTRCKESADFWCFECEQLLCAKCFEAHQWFLKHEARPLAELRNQSVREFLDG
TRKTNNIFCSNPNHRTPTLTSIYCRGCSKPLCCSCALLDSSHSELKCDISAEIQQRQEEL
DAMTQALQEQDSAFGAVHAQMHAAVGQLGRARAETEELIRERVRQVVAHVRAQERELLEA
VDARYQRDYEEMASRLGRLDAVLQRIRTGSALVQRMKCYASDQEVLDMHGFLRQALCRLR
QEEPQSLQAAVRTDGFDEFKVRLQDLSSCITQGKDAAVSKKASPEAASTPRDPIDVDLPE
EAERVKAQVQALGLAEAQPMAVVQSVPGAHPVPVYAFSIKGPSYGEDVSNTTTAQKRKCS
QTQCPRKVIKMESEEGKEARLARSSPEQPRPSTSKAVSPPHLDGPPSPRSPVIGSEVFLP
NSNHVASGAGEAEERVVVISSSEDSDAENSSSRELDDSSSESSDLQLEGPSTLRVLDENL
ADPQAEDRPLVFFDLKIDNETQKISQLAAVNRESKFRVVIQPEAFFSIYSKAVSLEVGLQ
HFLSFLSSMRRPILACYKLWGPGLPNFFRALEDINRLWEFQEAISGFLAALPLIRERVPG
ASSFKLKNLAQTYLARNMSERSAMAAVLAMRDLCRLLEVSPGPQLAQHVYPFSSLQCFAS
LQPLVQAAVLPRAEARLLALHNVSFMELLSAHRRDRQGGLKKYSRYLSLQTTTLPPAQPA
FNLQALGTYFEGLLEGPALARAEGVSTPLAGRGLAERASQQS
>sp|Q9Y487|VPP2_HUMAN V-type proton ATPase 116 kDa subunit a 2 OS=Homo sapiens OX=9606 GN=ATP6V0A2 PE=1 SV=2
MGSLFRSETMCLAQLFLQSGTAYECLSALGEKGLVQFRDLNQNVSSFQRKFVGEVKRCEE
LERILVYLVQEINRADIPLPEGEASPPAPPLKQVLEMQEQLQKLEVELREVTKNKEKLRK
NLLELIEYTHMLRVTKTFVKRNVEFEPTYEEFPSLESDSLLDYSCMQRLGAKLGFVSGLI
NQGKVEAFEKMLWRVCKGYTIVSYAELDESLEDPETGEVIKWYVFLISFWGEQIGHKVKK
ICDCYHCHVYPYPNTAEERREIQEGLNTRIQDLYTVLHKTEDYLRQVLCKAAESVYSRVI
QVKKMKAIYHMLNMCSFDVTNKCLIAEVWCPEADLQDLRRALEEGSRESGATIPSFMNII
PTKETPPTRIRTNKFTEGFQNIVDAYGVGSYREVNPALFTIITFPFLFAVMFGDFGHGFV
MFLFALLLVLNENHPRLNQSQEIMRMFFNGRYILLLMGLFSVYTGLIYNDCFSKSVNLFG
SGWNVSAMYSSSHPPAEHKKMVLWNDSVVRHNSILQLDPSIPGVFRGPYPLGIDPIWNLA
TNRLTFLNSFKMKMSVILGIIHMTFGVILGIFNHLHFRKKFNIYLVSIPELLFMLCIFGY
LIFMIFYKWLVFSAETSRVAPSILIEFINMFLFPASKTSGLYTGQEYVQRVLLVVTALSV
PVLFLGKPLFLLWLHNGRSCFGVNRSGYTLIRKDSEEEVSLLGSQDIEEGNHQVEDGCRE
MACEEFNFGEILMTQVIHSIEYCLGCISNTASYLRLWALSLAHAQLSDVLWAMLMRVGLR
VDTTYGVLLLLPVIALFAVLTIFILLIMEGLSAFLHAIRLHWVEFQNKFYVGAGTKFVPF
SFSLLSSKFNNDDSVA
>sp|Q9Y570|PPME1_HUMAN Protein phosphatase methylesterase 1 OS=Homo sapiens OX=9606 GN=PPME1 PE=1 SV=3
MSALEKSMHLGRLPSRPPLPGSGGSQSGAKMRMGPGRKRDFSPVPWSQYFESMEDVEVEN
ETGKDTFRVYKSGSEGPVLLLLHGGGHSALSWAVFTAAIISRVQCRIVALDLRSHGETKV
KNPEDLSAETMAKDVGNVVEAMYGDLPPPIMLIGHSMGGAIAVHTASSNLVPSLLGLCMI
DVVEGTAMDALNSMQNFLRGRPKTFKSLENAIEWSVKSGQIRNLESARVSMVGQVKQCEG
ITSPEGSKSIVEGIIEEEEEDEEGSESISKRKKEDDMETKKDHPYTWRIELAKTEKYWDG
WFRGLSNLFLSCPIPKLLLLAGVDRLDKDLTIGQMQGKFQMQVLPQCGHAVHEDAPDKVA
EAVATFLIRHRFAEPIGGFQCVFPGC
>sp|Q07954|LRP1_HUMAN Prolow-density lipoprotein receptor-related protein 1 OS=Homo sapiens OX=9606 GN=LRP1 PE=1 SV=2
MLTPPLLLLLPLLSALVAAAIDAPKTCSPKQFACRDQITCISKGWRCDGERDCPDGSDEA
PEICPQSKAQRCQPNEHNCLGTELCVPMSRLCNGVQDCMDGSDEGPHCRELQGNCSRLGC
QHHCVPTLDGPTCYCNSSFQLQADGKTCKDFDECSVYGTCSQLCTNTDGSFICGCVEGYL
LQPDNRSCKAKNEPVDRPPVLLIANSQNILATYLSGAQVSTITPTSTRQTTAMDFSYANE
TVCWVHVGDSAAQTQLKCARMPGLKGFVDEHTINISLSLHHVEQMAIDWLTGNFYFVDDI
DDRIFVCNRNGDTCVTLLDLELYNPKGIALDPAMGKVFFTDYGQIPKVERCDMDGQNRTK

LVDSKIVFPHGITLDLVSRLVYWADAYLDYIEVVDYEGKGRQTIIQGILIEHLYGLTVFE
NYLYATNSDNANAQQKTSVIRVNRFNSTEYQVVTRVDKGGALHIYHQRRQPRVRSHACEN
DQYGKPGGCSDICLLANSHKARTCRCRSGFSLGSDGKSCKKPEHELFLVYGKGRPGIIRG
MDMGAKVPDEHMIPIENLMNPRALDFHAETGFIYFADTTSYLIGRQKIDGTERETILKDG
IHNVEGVAVDWMGDNLYWTDDGPKKTISVARLEKAAQTRKTLIEGKMTHPRAIVVDPLNG
WMYWTDWEEDPKDSRRGRLERAWMDGSHRDIFVTSKTVLWPNGLSLDIPAGRLYWVDAFY
DRIETILLNGTDRKIVYEGPELNHAFGLCHHGNYLFWTEYRSGSVYRLERGVGGAPPTVT
LLRSERPPIFEIRMYDAQQQQVGTNKCRVNNGGCSSLCLATPGSRQCACAEDQVLDADGV
TCLANPSYVPPPQCQPGEFACANSRCIQERWKCDGDNDCLDNSDEAPALCHQHTCPSDRF
KCENNRCIPNRWLCDGDNDCGNSEDESNATCSARTCPPNQFSCASGRCIPISWTCDLDDD
CGDRSDESASCAYPTCFPLTQFTCNNGRCININWRCDNDNDCGDNSDEAGCSHSCSSTQF
KCNSGRCIPEHWTCDGDNDCGDYSDETHANCTNQATRPPGGCHTDEFQCRLDGLCIPLRW
RCDGDTDCMDSSDEKSCEGVTHVCDPSVKFGCKDSARCISKAWVCDGDNDCEDNSDEENC
ESLACRPPSHPCANNTSVCLPPDKLCDGNDDCGDGSDEGELCDQCSLNNGGCSHNCSVAP
GEGIVCSCPLGMELGPDNHTCQIQSYCAKHLKCSQKCDQNKFSVKCSCYEGWVLEPDGES
CRSLDPFKPFIIFSNRHEIRRIDLHKGDYSVLVPGLRNTIALDFHLSQSALYWTDVVEDK
IYRGKLLDNGALTSFEVVIQYGLATPEGLAVDWIAGNIYWVESNLDQIEVAKLDGTLRTT
LLAGDIEHPRAIALDPRDGILFWTDWDASLPRIEAASMSGAGRRTVHRETGSGGWPNGLT
VDYLEKRILWIDARSDAIYSARYDGSGHMEVLRGHEFLSHPFAVTLYGGEVYWTDWRTNT
LAKANKWTGHNVTVVQRTNTQPFDLQVYHPSRQPMAPNPCEANGGQGPCSHLCLINYNRT
VSCACPHLMKLHKDNTTCYEFKKFLLYARQMEIRGVDLDAPYYNYIISFTVPDIDNVTVL
DYDAREQRVYWSDVRTQAIKRAFINGTGVETVVSADLPNAHGLAVDWVSRNLFWTSYDTN
KKQINVARLDGSFKNAVVQGLEQPHGLVVHPLRGKLYWTDGDNISMANMDGSNRTLLFSG
QKGPVGLAIDFPESKLYWISSGNHTINRCNLDGSGLEVIDAMRSQLGKATALAIMGDKLW
WADQVSEKMGTCSKADGSGSVVLRNSTTLVMHMKVYDESIQLDHKGTNPCSVNNGDCSQL
CLPTSETTRSCMCTAGYSLRSGQQACEGVGSFLLYSVHEGIRGIPLDPNDKSDALVPVSG
TSLAVGIDFHAENDTIYWVDMGLSTISRAKRDQTWREDVVTNGIGRVEGIAVDWIAGNIY
WTDQGFDVIEVARLNGSFRYVVISQGLDKPRAITVHPEKGYLFWTEWGQYPRIERSRLDG
TERVVLVNVSISWPNGISVDYQDGKLYWCDARTDKIERIDLETGENREVVLSSNNMDMFS
VSVFEDFIYWSDRTHANGSIKRGSKDNATDSVPLRTGIGVQLKDIKVFNRDRQKGTNVCA
VANGGCQQLCLYRGRGQRACACAHGMLAEDGASCREYAGYLLYSERTILKSIHLSDERNL
NAPVQPFEDPEHMKNVIALAFDYRAGTSPGTPNRIFFSDIHFGNIQQINDDGSRRITIVE
NVGSVEGLAYHRGWDTLYWTSYTTSTITRHTVDQTRPGAFERETVITMSGDDHPRAFVLD
ECQNLMFWTNWNEQHPSIMRAALSGANVLTLIEKDIRTPNGLAIDHRAEKLYFSDATLDK
IERCEYDGSHRYVILKSEPVHPFGLAVYGEHIFWTDWVRRAVQRANKHVGSNMKLLRVDI
PQQPMGIIAVANDTNSCELSPCRINNGGCQDLCLLTHQGHVNCSCRGGRILQDDLTCRAV
NSSCRAQDEFECANGECINFSLTCDGVPHCKDKSDEKPSYCNSRRCKKTFRQCSNGRCVS
NMLWCNGADDCGDGSDEIPCNKTACGVGEFRCRDGTCIGNSSRCNQFVDCEDASDEMNCS
ATDCSSYFRLGVKGVLFQPCERTSLCYAPSWVCDGANDCGDYSDERDCPGVKRPRCPLNY
FACPSGRCIPMSWTCDKEDDCEHGEDETHCNKFCSEAQFECQNHRCISKQWLCDGSDDCG
DGSDEAAHCEGKTCGPSSFSCPGTHVCVPERWLCDGDKDCADGADESIAAGCLYNSTCDD
REFMCQNRQCIPKHFVCDHDRDCADGSDESPECEYPTCGPSEFRCANGRCLSSRQWECDG
ENDCHDQSDEAPKNPHCTSQEHKCNASSQFLCSSGRCVAEALLCNGQDDCGDSSDERGCH
INECLSRKLSGCSQDCEDLKIGFKCRCRPGFRLKDDGRTCADVDECSTTFPCSQRCINTH
GSYKCLCVEGYAPRGGDPHSCKAVTDEEPFLIFANRYYLRKLNLDGSNYTLLKQGLNNAV
ALDFDYREQMIYWTDVTTQGSMIRRMHLNGSNVQVLHRTGLSNPDGLAVDWVGGNLYWCD
KGRDTIEVSKLNGAYRTVLVSSGLREPRALVVDVQNGYLYWTDWGDHSLIGRIGMDGSSR

SVIVDTKITWPNGLTLDYVTERIYWADAREDYIEFASLDGSNRHVVLSQDIPHIFALTLF
EDYVYWTDWETKSINRAHKTTGTNKTLLISTLHRPMDLHVFHALRQPDVPNHPCKVNNGG
CSNLCLLSPGGGHKCACPTNFYLGSDGRTCVSNCTASQFVCKNDKCIPFWWKCDTEDDCG
DHSDEPPDCPEFKCRPGQFQCSTGICTNPAFICDGDNDCQDNSDEANCDIHVCLPSQFKC
TNTNRCIPGIFRCNGQDNCGDGEDERDCPEVTCAPNQFQCSITKRCIPRVWVCDRDNDCV
DGSDEPANCTQMTCGVDEFRCKDSGRCIPARWKCDGEDDCGDGSDEPKEECDERTCEPYQ
FRCKNNRCVPGRWQCDYDNDCGDNSDEESCTPRPCSESEFSCANGRCIAGRWKCDGDHDC
ADGSDEKDCTPRCDMDQFQCKSGHCIPLRWRCDADADCMDGSDEEACGTGVRTCPLDEFQ
CNNTLCKPLAWKCDGEDDCGDNSDENPEECARFVCPPNRPFRCKNDRVCLWIGRQCDGTD
NCGDGTDEEDCEPPTAHTTHCKDKKEFLCRNQRCLSSSLRCNMFDDCGDGSDEEDCSIDP
KLTSCATNASICGDEARCVRTEKAAYCACRSGFHTVPGQPGCQDINECLRFGTCSQLCNN
TKGGHLCSCARNFMKTHNTCKAEGSEYQVLYIADDNEIRSLFPGHPHSAYEQAFQGDESV
RIDAMDVHVKAGRVYWTNWHTGTISYRSLPPAAPPTTSNRHRRQIDRGVTHLNISGLKMP
RGIAIDWVAGNVYWTDSGRDVIEVAQMKGENRKTLISGMIDEPHAIVVDPLRGTMYWSDW
GNHPKIETAAMDGTLRETLVQDNIQWPTGLAVDYHNERLYWADAKLSVIGSIRLNGTDPI
VAADSKRGLSHPFSIDVFEDYIYGVTYINNRVFKIHKFGHSPLVNLTGGLSHASDVVLYH
QHKQPEVTNPCDRKKCEWLCLLSPSGPVCTCPNGKRLDNGTCVPVPSPTPPPDAPRPGTC
NLQCFNGGSCFLNARRQPKCRCQPRYTGDKCELDQCWEHCRNGGTCAASPSGMPTCRCPT
GFTGPKCTQQVCAGYCANNSTCTVNQGNQPQCRCLPGFLGDRCQYRQCSGYCENFGTCQM
AADGSRQCRCTAYFEGSRCEVNKCSRCLEGACVVNKQSGDVTCNCTDGRVAPSCLTCVGH
CSNGGSCTMNSKMMPECQCPPHMTGPRCEEHVFSQQQPGHIASILIPLLLLLLLVLVAGV
VFWYKRRVQGAKGFQHQRMTNGAMNVEIGNPTYKMYEGGEPDDVGGLLDADFALDPDKPT
NFTNPVYATLYMGGHGSRHSLASTDEKRELLGRGPEDEIGDPLA
>sp|A4UGR9|XIRP2_HUMAN Xin actin-binding repeat-containing protein 2 OS=Homo sapiens OX=9606 GN=XIRP2 PE=1 SV=2
MSPESGHSRIFEATAGPNKPESGFAEDSAARGEGVSDLHEVVSLKERMARYQAAVSRGDC
RSFSANMMEESEMCAVPGGLAKVKKQFEDEITSSRNTFAQYQYQHQNRSEQEAIHSSQVG
TSRSSQEMARNEQEGSKVQKIDVHGTEMVSHLEKHTEEVNQASQFHQYVQETVIDTPEDE
EIPKVSTKLLKEQFEKSAQEKILYSDKEMTTPAKQIKTESEYEETFKPSSVVSTSSTSCV
STSQRKETSTTRYSDHSVTSSTLAQINATSSGMTEEFPPPPPDVLQTSVDVTAFSQSPEL
PSPPRRLPVPKDVYSKQRNLYELNRLYKHIHPELRKNLEKDYISEVSEIVSSQMNSGSSV
SADVQQARYVFENTNDSSQKDLNSEREYLEWDEILKGEVQSIRWIFENQPLDSINNGSPD
EGDISRGIADQEIIAGGDVKYTTWMFETQPIDTLGAYSSDTVENAEKIPELARGDVCTAR
WMFETRPLDSMNKMHQSQEESAVTISKDITGGDVKTVRYMFETQHLDQLGQLHSVDEVHL
LQLRSELKEIKGNVKRSIKCFETQPLYVIRDGSGQMLEIKTVHREDVEKGDVRTARWMFE
TQPLDTINKDITEIKVVRGISMEENVKGGVSKAKWLFETQPLEKIKESEEVIIEKEKIIG
TDVSRKCWMFETQPLDILKEVPDADSLQREEIIGGDVQTTKHLFETLPIEALKDSPDIGK
LQKITASEEEKGDVRHQKWIFETQPLEDIRKDKKEYTRTVKLEEVDRGDVKNYTHIFESN
NLIKFDASHKIEVEGVTRGAVELNKSLFETTPLYAIQDPLGKYHQVKTVQQEEIVRGDVR
SCRWLFETRPIDQFDESIHKFQIIRGISAQEIQTGNVKSAKWLFETQPLDSIKYFSDVEE
TESKTEQTRDIVKGDVKTCKWLFETQPMESLYEKVSLMTSSEEIHKGDVKTCTWLFETQP
LDTIKDDSETAVKLQTVKQEEIQGGDVRTACFLFETENLDSIQGEEVKEIKPVEMDIQAG
DVSSMRYKFENQSLDSISSSSEEVLKKIKTLKTEDIQKGNVLNCRWLFENQPIDKIKESQ
EGDECVKTVTDIQGGDVRKGCFIFETFSLDEIKEESDYISTKKTITEEVIQGDVKSYRML
FETQPLYAIQDREGSYHEVTTVKKEEVIHGDVRGTRWLFETKPLDSINKSETVYVIKSVT
QEDIQKGDVSSVRYRFETQPLDQISEESHNIMPSIDHIQGGNVKTSRQFFESENFDKNNY
IRTVSVNEIQKGNVKTSTWLFETHTMDELRGEGLEYENIKTVTQEDVQKGDVKQAVWLFE

NRTFDSIMEAHKGITKMTKEEIPPSDVKTTTWLFETTPLHEFNETRVEKIEIIGKSIKET
LEDLYSQKVIQAPGIIIEADEIGDVRMAKYKLMNQASPEIQKEEIIRADLRNIMVNLLSK
RDCTEREILISEEEKGNVNLTKTQLLNRSTEFHAEKEEIVKGDVQQAIKNLFSEERSVKK
GILIQEDEKGDINMTIYCLLHENDGDTIEREEVIGGDVKRTIHNLLSSTSNNKISERAKI
DASERGNVQFFTTCIEAGALDYLKQLHTESNETLTAKKQEGEKEIIGGDVEGTKLLLKKR
QSLVERTVSETDIIPGDVHNTVKVFMTEPQSTFGKIPKEEIIKGDLTSTLNSLSQAVNQK
TVTKTEEIIKGNMLATLKSLKESSHRWKESKQPDAIPGDIEKAIECLEKATNTKTEILKK
ELLKDDLETSLRSLKEAQRSFKEVHKEGVIKKDAKAVMAGSSGEQKTDIHQVAVQRNKNS
LLQPKPGPFEPAAKWQGGADTLSQTMGKSCHGNLVEERTEVNLPKAPKGTVKIVIDREQN
NDALEKSLRRLSNSHHKSNVLESGDKTGVWTDTTGEQHLRDEYMSRQLTSTVSVKNNLTT
KESDRAVRELKKDDVFNSIQSAGKTVGKQQTYELRNDHQKMEGFHIKSPKKTKNIKILTD
TQSSKPSPTQHPVSMPVGGTYDLSGDFQKQTLLKQETKYSNKDIKKKNINLQPMWQLLPV
EQDTSNVTEMKVSEKSHNTFKATNKKRETDVHLKSQDFLMKTNTSTGLKMAMERSLNPIN
FNPENNVKESECPLPPPSPPPPPPSNASSEIEFPLPPPPPLMMFPEKNGFLPSLSTEKIK
AEFESFPGLPLPPPPVDEKSERESSSMFLPPPPPPTPSQKPAHLLSSSAPEKHSGDFMQQ
YSQKEASNSQNSQAKIITGKTGVLPPPTLPKPKLPKHIKDNKNDFSPKVELATSLSDMEC
KITTSKDQKKVMVMTSSEHTETKQNVISKSLDERKQLSIDSANCLSHTVPGTSAPRKKQI
APLIKSHSFPESSGQQNPKPYMRKFKTPLMIAEEKYRQQKEEIEKQKQESSYYNIVKTQS
QNQHITEVEKEMPLQKTNEEVSLSGIDSECTVVQPSPGSQSNARILGVCSDNQLSTTSPE
TVAAKRLHHVLAASEDKDKMKKEVLQSSRDIMQSKSACEIKQSHQECSTQQTQQKKYLEQ
LHLPQSKPISPNFKVKTIKLPTLDHTLNETDHSYESHKQQSEIDVQTFTKKQYLKTKKTE
ASTECSHKQSLAERHYQLPKKEKRVTVQLPTESIQKNQEDKLKMVPRKQREFSGSDRGKL
PGSEEKNQGPSMIGRKEERLITERKHEHLKNKSAPKVVKQKVIDAHLDSQTQNFQQTQIQ
TAESKAEHKKLPQPYNSLQEEKCLEVKGIQEKQVFSNTKDSKQEITQNKSFFSSVKESQR
DDGKGALNIVEFLRKREELQQILSRVKQFEAEPNKSGLKTFQTLLNTIPGWLISEDKREY
AVHIAMENNLEKVKEEITHIKTQAEDMLVSYENIIQTAMMSSKTGKPGNKPTSLDETSSK
VSNVHVSNNKNSEQKENKIAKEKTVQHQVAAHHEATVRSHVKTHQEIKLDDSNIPPPSLK
TRPPSPTFITIESTARRTENPTKNELSQSPKKDSYVEPPPRRPMSQKSEIHRANTSPSPP
RSRSEQLVRLKDTTAKLSKGAIPCPAATPVPIVEKRSEIIMSPATLRRQIKIETRGRDSP
PTITIPVNINHAASGSFRESVDAQEEIRKVEKRATYVHKDGLNSTDHMVPDTESYDAVEI
IRKVAVPPRLSEHTQRYEAANRTVQMAENFVNDPENEINRWFREFEHGPVSEAKSNRRVY
AKGETNHNIQQESRTFCKEEFGLTSLGNTSFTDFSCKHPRELREKIPVKQPRICSETRSL
SEHFSGMDAFESQIVESKMKTSSSHSSEAGKSGCDFKHAPPTYEDVIAGHILDISDSPKE
VRKNFQKTWQESGRVFKGLGYATADASATEMRTTFQEESAFISEAAAPRQGNMYTLSKDS
LSNGVPSGRQAEFS
>sp|P29374|ARI4A_HUMAN AT-rich interactive domain-containing protein 4A OS=Homo sapiens OX=9606 GN=ARID4A PE=1 SV=3
MKAADEPAYLTVGTDVSAKYRGAFCEAKIKTVKRLVKVKVLLKQDNTTQLVQDDQVKGPL
RVGAIVETRTSDGSFQEAIISKLTDASWYTVVFDDGDERTLRRTSLCLKGERHFAESETL
DQLPLTNPEHFGTPVIAKKTNRGRRSSLPVTEDEKEEESSEEEDEDKRRLNDELLGKVVS
VVSATERTEWYPALVISPSCNDDITVKKDQCLVRSFIDSKFYSIARKDIKEVDILNLPES
ELSTKPGLQKASIFLKTRVVPDNWKMDISEILESSSSDDEDGPAEENDEEKEKEAKKTEE
EVPEEELDPEERDNFLQQLYKFMEDRGTPINKPPVLGYKDLNLFKLFRLVYHQGGCDNID
SGAVWKQIYMDLGIPILNSAASYNVKTAYRKYLYGFEEYCRSANIQFRTVHHHEPKVKEE
KKDLEESMEEALKLDQEMPLTEVKSEPEENIDSNSESEREEIELKSPRGRRRIARDVNSI
KKEIEEEKTEDKLKDNDTENKDVDDDYETAEKKENELLLGRKNTPKQKEKKIKKQEDSDK
DSDEEEEKSQEREETESKCDSEGEEDEEDMEPCLTGTKVKVKYGRGKTQKIYEASIKSTE

IDDGEVLYLVHYYGWNVRYDEWVKADRIIWPLDKGGPKKKQKKKAKNKEDSEKDEKRDEE
RQKSKRGRPPLKSTLSSNMPYGLSKTANSEGKSDSCSSDSETEDALEKNLINEELSLKDE
LEKNENLNDDKLDEENPKISAHILKENDRTQMQPLETLKLEVGENEQIVQIFGNKMEKTE
EVKKEAEKSPKGKGRRSKTKDLSLEIIKISSFGQNEAGSEPHIEAHSLELSSLDNKNFSS
ATEDEIDQCVKEKKLKRKILGQSSPEKKIRIENGMEMTNTVSQERTSDCIGSEGMKNLNF
EQHFERENEGMPSLIAESNQCIQQLTSERFDSPAEETVNIPLKEDEDAMPLIGPETLVCH
EVDLDDLDEKDKTSIEDVAVESSESNSLVSIPPALPPVVQHNFSVASPLTLSQDESRSVK
SESDITIEVDSIAEESQEGLCERESANGFETNVASGTCSIIVQERESREKGQKRPSDGNS
GLMAKKQKRTPKRTSAAAKNEKNGTGQSSDSEDLPVLDNSSKCTPVKHLNVSKPQKLARS
PARISPHIKDGEKDKHREKHPNSSPRTYKWSFQLNELDNMNSTERISFLQEKLQEIRKYY
MSLKSEVATIDRRRKRLKKKDREVSHAGASMSSASSDTGMSPSSSSPPQNVLAVECR
>sp|P0DOX4|IGE_HUMAN Immunoglobulin epsilon heavy chain OS=Homo sapiens OX=9606 PE=1 SV=1
QVQLVQSGAEVRKPGASVRVSCKASGYTFIDSYVGWIRQAPGHGLEWIHWINPNSGGTNY
APRFQGRVTMTRDASFSTAYMDLRSLRSDDSAVFYCAKSDPFWSDYNFDYSSSEEGTEVT
YTVSGAWTLPSVFPLTRCCKNIPSNATSVTLGCLATGYFPEPVMVTWDTGSLNGTTLPAT
TLTLSGHYATISLLTVSGAWAKQMFTCRVAHTPSSTVDNKTFSVCSRDFTPPTVKILQSS
CDGLGHFPPTIQLCLVSGYTPGTINITWLEDGQVMDVDLSTASTESQGELASTESQLTLS
QKHWLSDRTYTCQVTYQGHTFQDSTKKCADSNPRGVSAYLSRPSPFDLFIRKSPTITCLV
VDLAPSKGTVNLTWSRASGKPVNHSTRKEEKQRNGTLTVTSTLPVGTRDWIEGETYQCRV
THPHLPRALMRSTTKTSGPRAAPEVYAFATPEWPGSRDKRTLACLIQNFMPEDISVQWLH
NEVQLPDARHSTTQPRKTKGSGFFVFSRLEVTRAEWQEKDEFICRAVHEAASPSQTVQRA
VSVNPGK
>sp|Q86UP3|ZFHX4_HUMAN Zinc finger homeobox protein 4 OS=Homo sapiens OX=9606 GN=ZFHX4 PE=1 SV=1
METCDSPPISRQENGQSTSKLCGTTQLDNEVPEKVAGMEPDRENSSTDDNLKTDERKSEA
LLGFSVENAAATQVTSAKEIPCNECATSFPSLQKYMEHHCPNARLPVLKDDNESEISELE
DSDVENLTGEIVYQPDGSAYIIEDSKESGQNAQTGANSKLFSTAMFLDSLASAGEKSDQS
ASAPMSFYPQIINTFHIASSLGKPFTADQAFPNTSALAGVGPVLHSFRVYDLRHKREKDY
LTSDGSAKNSCVSKDVPNNVDLSKFDGCVSDGKRKPVLMCFLCKLSFGYIRSFVTHAVHD
HRMTLNDEEQKLLSNKCVSAIIQGIGKDKEPLISFLEPKKSTSVYPHFSTTNLIGPDPTF
RGLWSAFHVENGDSLPAGFAFLKGSASTSSSAEQPLGITQMPKAEVNLGGLSSLVVNTPI
TSVSLSHSSSESSKMSESKDQENNCERPKESNVLHPNGECPVKSEPTEPGDEDEEDAYSN
ELDDEEVLGELTDSIGNKDFPLLNQSISPLSSSVLKFIEKGTSSSSATVSDDTEKKKQTA
AVRASGSVASNYGISGKDFADASASKDSATAAHPSEIARGDEDSSATPHQHGFTPSTPGT
PGPGGDGSPGSGIECPKCDTVLGSSRSLGGHMTMMHSRNSCKTLKCPKCNWHYKYQQTLE
AHMKEKHPEPGGSCVYCKTGQPHPRLARGESYTCGYKPFRCEVCNYSTTTKGNLSIHMQS
DKHLNNVQNLQNGNGEQVFGHSAPAPNTSLSGCGTPSPSKPKQKPTWRCEVCDYETNVAR
NLRIHMTSEKHMHNMMLLQQNMKQIQHNLHLGLAPAEAELYQYYLAQNIGLTGMKLENPA
DPQLMINPFQLDPATAAALAPGLGELSPYISDPALKLFQCAVCNKFTSDSLEALSVHVSS
ERSLPEEEWRAVIGDIYQCKLCNYNTQLKANFQLHCKTDKHMQKYQLVAHIKEGGKSNEW
RLKCIAIGNPVHLKCNACDYYTNSVDKLRLHTTNHRHEAALKLYKHLQKQEGAVNPESCY
YYCAVCDYTTKVKLNLVQHVRSVKHQQTEGLRKLQLHQQGLAPEEDNLSEIFFVKDCPPN
ELETASLGARTCDDDLTEQHEEAEGAIKPTAVAEDDEKDTSERDNSEGKNSNKDSVSVAG
GTQPLLLAKEEDVATKRSKPTEDNKFCHEQFYQCPYCNYNSRDQSRIQMHVLSQHSVQPV
ICCPLCQDVLSNKMHLQLHLTHLHSVSPDCVEKLLMTVPVPDVMMPNSMLLPAAASEKSE
RDTPAAVTAEGSGKYSGESPMDDKSMAGLEDSKANVEVKNEEQKPTKEPLEVSEWNKNSS

KDVKIPDTLQDQLNEQQKRQPLSVSDRHVYKYRCNHCSLAFKTMQKLQIHSQYHAIRAAT
MCNLCQRSFRTFQALKKHLEAGHPELSEAELQQLYASLPVNGELWAESETMSQDDHGLEQ
EMEREYEVDHEGKASPVGSDSSSIPDDMGSEPKRTLPFRKGPNFTMEKFLDPSRPYKCTV
CKESFTQKNILLVHYNSVSHLHKLKKVLQEASSPVPQETNSNTDNKPYKCSICNVAYSQS
STLEIHMRSVLHQTKARAAKLEPSGHVAGGHSIAANVNSPGQGMLDSMSLAAVNSKDTHL
DAKELNKKQTPDLISAQPAHHPPQSPAQIQMQLQHELQQQAAFFQPQFLNPAFLPHFPMT
PEALLQFQQPQFLFPFYIPGTEFSLGPDLGLPGSATFGMPGMTGMAGSLLEDLKQQIQTQ
HHVGQTQLQILQQQAQQYQATQPQLQPQKQQQQPPPPQQQQQQQASKLLKQEQSNIVSAD
CQIMKDVPSYKEAEDISEKPEKPKQEFISEGEGLKEGKDTKKQKSLEPSIPPPRIASGAR
GNAAKALLENFGFELVIQYNENRQKVQKKGKSGEGENTDKLECGTCGKLFSNVLILKSHQ
EHVHGQFFPYAALEKFARQYREAYDKLYPISPSSPETPPPPPPPPPLPPAPPQPSSMGPV
KIPNTVSTPLQAPPPTPPPPPPPPPPPPPPPPPPPPPSAPPQVQLPVSLDLPLFPSIMMQP
VQHPALPPQLALQLPQMDALSADLTQLCQQQLGLDPNFLRHSQFKRPRTRITDDQLKILR
AYFDINNSPSEEQIQEMAEKSGLSQKVIKHWFRNTLFKERQRNKDSPYNFSNPPITVLED
IRIDPQPTSLEHYKSDASFSKRSSRTRFTDYQLRVLQDFFDTNAYPKDDEIEQLSTVLNL
PTRVIVVWFQNARQKARKSYENQAETKDNEKRELTNERYIRTSNMQYQCKKCNVVFPRIF
DLITHQKKQCYKDEDDDAQDESQTEDSMDATDQVVYKHCTVSGQTDAAKNAAAPAASSGS
GTSTPLIPSPKPEPEKTSPKPEYPAEKPKQSDPSPPSQGTKPALPLASTSSDPPQASTAQ
PQPQPQPPKQPQLIGRPPSASQTPVPSSPLQISMTSLQNSLPPQLLQYQCDQCTVAFPTL
ELWQEHQHMHFLAAQNQFLHSPFLERPMDMPYMIFDPNNPLMTGQLLGSSLTQMPPQASS
SHTTAPTTVAASLKRKLDDKEDNNCSEKEGGNSGEDQHRDKRLRTTITPEQLEILYEKYL
LDSNPTRKMLDHIAREVGLKKRVVQVWFQNTRARERKGQFRAVGPAQSHKRCPFCRALFK
AKSALESHIRSRHWNEGKQAGYSLPPSPLISTEDGGESPQKYIYFDYPSLPLTKIDLSSE
NELASTVSTPVSKTAELSPKNLLSPSSFKAECSEDVENLNAPPAEAGYDQNKTDFDETSS
INTAISDATTGDEGNTEMESTTGSSGDVKPALSPKEPKTLDTLPKPATTPTTEVCDDKFL
FSLTSPSIHFNDKDGDHDQSFYITDDPDDNADRSETSSIADPSSPNPFGSSNPFKSKSND
RPGHKRFRTQMSNLQLKVLKACFSDYRTPTMQECEMLGNEIGLPKRVVQVWFQNARAKEK
KFKINIGKPFMINQGGTEGTKPECTLCGVKYSARLSIRDHIFSKQHISKVRETVGSQLDR
EKDYLAPTTVRQLMAQQELDRIKKASDVLGLTVQQPGMMDSSSLHGISLPTAYPGLPGLP
PVLLPGMNGPSSLPGFPQNSNISAGMLGFPTSATSSPALSLSSAPTKPLLQTPPPPPPPP
PPPPSSSLSGQQTEQQNKESEKKQTKPNKVKKIKEEELEATKPEKHPKKEEKISSALSVL
GKVVGETHVDPIQLQALQNAIAGDPASFIGGQFLPYFIPGFASYFTPQLPGTVQGGYFPP
VCGMESLFPYGPTMPQTLAGLSPGALLQQYQQYQQNLQESLQKQQKQQQEQQQKPVQAKT
SKVESDQPQNSNDASETKEDKSTATESTKEEPQLESKSADFSDTYVVPFVKYEFICRKCQ
MMFTDEDAAVNHQKSFCYFGQPLIDPQETVLRVPVSKYQCLACDVAISGNEALSQHLQSS
LHKEKTIKQAMRNAKEHVRLLPHSVCSPNPNTTSTSQSAASSNNTYPHLSCFSMKSWPNI
LFQASARRAASPPSSPPSLSLPSTVTSSLCSTSGVQTSLPTESCSDESDSELSQKLEDLD
NSLEVKAKPASGLDGNFNSIRMDMFSV
>sp|Q8IZT6|ASPM_HUMAN Abnormal spindle-like microcephaly-associated protein OS=Homo sapiens OX=9606 GN=ASPM PE=1 SV=2
MANRRVGRGCWEVSPTERRPPAGLRGPAAEEEASSPPVLSLSHFCRSPFLCFGDVLLGAS
RTLSLALDNPNEEVAEVKISHFPAADLGFSVSQRCFVLQPKEKIVISVNWTPLKEGRVRE
IMTFLVNDVLKHQAILLGNAEEQKKKKRSLWDTIKKKKISASTSHNRRVSNIQNVNKTFS
VSQKVDRVRSPLQACENLAMNEGGPPTENNSLILEENKIPISPISPAFNECHGATCLPLS
VRRSTTYSSLHASENRELLNVHSANVSKVSFNEKAVTETSFNSVNVNGQRGENSKLSLTP
NCSSTLNITQSQIHFLSPDSFVNNSHGANNELELVTCLSSDMFMKDNSQPVHLESTIAHE
IYQKILSPDSFIKDNYGLNQDLESESVNPILSPNQFLKDNMAYMCTSQQTCKVPLSNENS

QVPQSPEDWRKSEVSPRIPECQGSKSPKAIFEELVEMKSNYYSFIKQNNPKFSAVQDISS
HSHNKQPKRRPILSATVTKRKATCTRENQTEINKPKAKRCLNSAVGEHEKVINNQKEKED
FHSYLPIIDPILSKSKSYKNEVTPSSTTASVARKRKSDGSMEDANVRVAITEHTEVREIK
RIHFSPSEPKTSAVKKTKNVTTPISKRISNREKLNLKKKTDLSIFRTPISKTNKRTKPII
AVAQSSLTFIKPLKTDIPRHPMPFAAKNMFYDERWKEKQEQGFTWWLNFILTPDDFTVKT
NISEVNAATLLLGIENQHKISVPRAPTKEEMSLRAYTARCRLNRLRRAACRLFTSEKMVK
AIKKLEIEIEARRLIVRKDRHLWKDVGERQKVLNWLLSYNPLWLRIGLETTYGELISLED
NSDVTGLAMFILNRLLWNPDIAAEYRHPTVPHLYRDGHEEALSKFTLKKLLLLVCFLDYA
KISRLIDHDPCLFCKDAEFKASKEILLAFSRDFLSGEGDLSRHLGLLGLPVNHVQTPFDE
FDFAVTNLAVDLQCGVRLVRTMELLTQNWDLSKKLRIPAISRLQKMHNVDIVLQVLKSRG
IELSDEHGNTILSKDIVDRHREKTLRLLWKIAFAFQVDISLNLDQLKEEIAFLKHTKSIK
KTISLLSCHSDDLINKKKGKRDSGSFEQYSENIKLLMDWVNAVCAFYNKKVENFTVSFSD
GRVLCYLIHHYHPCYVPFDAICQRTTQTVECTQTGSVVLNSSSESDDSSLDMSLKAFDHE
NTSELYKELLENEKKNFHLVRSAVRDLGGIPAMINHSDMSNTIPDEKVVITYLSFLCARL
LDLRKEIRAARLIQTTWRKYKLKTDLKRHQEREKAARIIQLAVINFLAKQRLRKRVNAAL
VIQKYWRRVLAQRKLLMLKKEKLEKVQNKAASLIQGYWRRYSTRQRFLKLKYYSIILQSR
IRMIIAVTSYKRYLWATVTIQRHWRAYLRRKQDQQRYEMLKSSTLIIQSMFRKWKQRKMQ
SQVKATVILQRAFREWHLRKQAKEENSAIIIQSWYRMHKELRKYIYIRSCVVIIQKRFRC
FQAQKLYKRRKESILTIQKYYKAYLKGKIERTNYLQKRAAAIQLQAAFRRLKAHNLCRQI
RAACVIQSYWRMRQDRVRFLNLKKTIIKFQAHVRKHQQRQKYKKMKKAAVIIQTHFRAYI
FAMKVLASYQKTRSAVIVLQSAYRGMQARKMYIHILTSVIKIQSYYRAYVSKKEFLSLKN
ATIKLQSTVKMKQTRKQYLHLRAAALFIQQCYRSKKIAAQKREEYMQMRESCIKLQAFVR
GYLVRKQMRLQRKAVISLQSYFRMRKARQYYLKMYKAIIVIQNYYHAYKAQVNQRKNFLQ
VKKAATCLQAAYRGYKVRQLIKQQSIAALKIQSAFRGYNKRVKYQSVLQSIIKIQRWYRA
YKTLHDTRTHFLKTKAAVISLQSAYRGWKVRKQIRREHQAALKIQSAFRMAKAQKQFRLF
KTAALVIQQNFRAWTAGRKQCMEYIELRHAVLVLQSMWKGKTLRRQLQRQHKCAIIIQSY
YRMHVQQKKWKIMKKAALLIQKYYRAYSIGREQNHLYLKTKAAVVTLQSAYRGMKVRKRI
KDCNKAAVTIQSKYRAYKTKKKYATYRASAIIIQRWYRGIKITNHQHKEYLNLKKTAIKI
QSVYRGIRVRRHIQHMHRAATFIKAMFKMHQSRISYHTMRKAAIVIQVRCRAYYQGKMQR
EKYLTILKAVKVLQASFRGVRVRRTLRKMQTAATLIQSNYRRYRQQTYFNKLKKITKTVQ
QRYWAMKERNIQFQRYNKLRHSVIYIQAIFRGKKARRHLKMMHIAATLIQRRFRTLMMRR
RFLSLKKTAILIQRKYRAHLCTKHHLQFLQVQNAVIKIQSSYRRWMIRKRMREMHRAATF
IQSTFRMHRLHMRYQALKQASVVIQQQYQANRAAKLQRQHYLRQRHSAVILQAAFRGMKT
RRHLKSMHSSATLIQSRFRSLLVRRRFISLKKATIFVQRKYRATICAKHKLYQFLHLRKA
AITIQSSYRRLMVKKKLQEMQRAAVLIQATFRMYRTYITFQTWKHASILIQQHYRTYRAA
KLQRENYIRQWHSAVVIQAAYKGMKARQLLREKHKASIVIQSTYRMYRQYCFYQKLQWAT
KIIQEKYRANKKKQKVFQHNELKKETCVQAGFQDMNIKKQIQEQHQAAIIIQKHCKAFKI
RKHYLHLRATVVSIQRRYRKLTAVRTQAVICIQSYYRGFKVRKDIQNMHRAATLIQSFYR
MHRAKVDYETKKTAIVVIQNYYRLYVRVKTERKNFLAVQKSVRTIQAAFRGMKVRQKLKN
VSEEKMAAIVNQSALCCYRSKTQYEAVQSEGVMIQEWYKASGLACSQEAEYHSQSRAAVT
IQKAFCRMVTRKLETQKCAALRIQFFLQMAVYRRRFVQQKRAAITLQHYFRTWQTRKQFL
LYRKAAVVLQNHYRAFLSAKHQRQVYLQIRSSVIIIQARSKGFIQKRKFQEIKNSTIKIQ
AMWRRYRAKKYLCKVKAACKIQAWYRCWRAHKEYLAILKAVKIIQGCFYTKLERTRFLNV
RASAIIIQRKWRAILPAKIAHEHFLMIKRHRAACLIQAHYRGYKGRQVFLRQKSAALIIQ
KYIRAREAGKHERIKYIEFKKSTVILQALVRGWLVRKRFLEQRAKIRLLHFTAAAYYHLN
AVRIQRAYKLYLAVKNANKQVNSVICIQRWFRARLQEKRFIQKYHSIKKIEHEGQECLSQ
RNRAASVIQKAVRHFLLRKKQEKFTSGIIKIQALWRGYSWRKKNDCTKIKAIRLSLQVVN

REIREENKLYKRTALALHYLLTYKHLSAILEALKHLEVVTRLSPLCCENMAQSGAISKIF
VLIRSCNRSIPCMEVIRYAVQVLLNVSKYEKTTSAVYDVENCIDILLELLQIYREKPGNK
VADKGGSIFTKTCCLLAILLKTTNRASDVRSRSKVVDRIYSLYKLTAHKHKMNTERILYK
QKKNSSISIPFIPETPVRTRIVSRLKPDWVLRRDNMEEITNPLQAIQMVMDTLGIPY
>sp|C9JJH3|U17LA_HUMAN Ubiquitin carboxyl-terminal hydrolase 17-like protein 10 OS=Homo sapiens OX=9606 GN=USP17L10 PE=3 SV=1
MEDDSLYLGGEWQFNHFSKLTSSRPDAAFAEIQRTSLPEKSPLSCETRVDLCDDLAPVAR
QLAPREKPPLSSRRPAAVGAGLQNMGNTCYVNASLQCLTYKPPLANYMLFREHSQTCHRH
KGCMLCTMQAHITRALHIPGHVIQPSQALAAGFHRGKQEDAHEFLMFTVDAMRKACLPGH
KQVDRHSKDTTLIHQIFGGYWRSQIKCLHCHGISDTFDPYLDIALDIQAAQSVQQALEQL
VKPEELNGENAYHCGVCLQRAPASKTLTLHNSAKVLILVLKRFPDVTGNKIAKNVQYPEC
LDMQPYMSQQNTGPLVYVLYAVLVHAGWSCHNGHYSSYVKAQEGQWYKMDDAEVTASSIT
SVLSQQAYVLFYIQKSEWERHSESVSRGREPRALGVEDTDRRATQGELKRDHPCLQAPEL
DEHLVERATQESTLDHWKFLQEQNKTKPEFNVRRVEGTVPPDVLVIHQSKYKCRMKNHHP
EQQSSLLNLSSTTPTDQESMNTGTLASLRGRTRRSKGKNKHSKRALLVCQ
>sp|Q7RTZ2|U17L1_HUMAN Ubiquitin carboxyl-terminal hydrolase 17-like protein 1 OS=Homo sapiens OX=9606 GN=USP17L1 PE=3 SV=1
MGDDSLYLGGEWQFNHFSKLTSSRPDAAFAEIQRTSLPEKSPLSSETRVDLCDDLAPVAR
QLAPREKLPLSSRRPAAVGAGLQNMGNTCYENASLQCLTYTLPLANYMLSREHSQTCQRP
KCCMLCTMQAHITWALHSPGHVIQPSQALAAGFHRGKQEDVHEFLMFTVDAMKKACLPGH
KQVDHHCKDTTLIHQIFGGCWRSQIKCLHCHGISDTFDPYLDIALDIQAAQSVKQALEQL
VKPEELNGENAYHCGLCLQRAPASNTLTLHTSAKVLILVLKRFSDVAGNKLAKNVQYPEC
LDMQPYMSQQNTGPLVYVLYAVLVHAGWSCHDGHYFSYVKAQEVQWYKMDDAEVTVCSII
SVLSQQAYVLFYIQKSEWERHSESVSRGREPRALGAEDTDRRAKQGELKRDHPCLQAPEL
DEHLVERATQESTLDHWKFLQEQNKTKPEFNVGKVEGTLPPNALVIHQSKYKCGMKNHHP
EQQSSLLNLSSTTRTDQESMNTGTLASLQGRTRRAKGKNKHSKRALLVCQ
>sp|A6NCW0|U17L3_HUMAN Ubiquitin carboxyl-terminal hydrolase 17-like protein 3 OS=Homo sapiens OX=9606 GN=USP17L3 PE=3 SV=1
MGDDSLYLGGEWQFNHFSKLTSSRPDAAFAEIQRTSLPEKSPLSSETRVDLCDDLAPVAR
QLAPREKLPLSSRRPAAVGAGLQNMGNTCYENASLQCLTYTLPLANYMLSREHSQTCQRP
KCCMLCTMQAHITWALHSPGHVIQPSQALASGFHRGKQEDVHEFLMFTVDAMKKACLPGH
KQVDHHSKDTTLIHQIFGGCWRSQIKCLHCHGISDTFDPYLDIALDIQAAQSVKQALEQL
VKPEELNGENAYHCGLCLQRAPASNTLTLHTSAKVLILVLKRFSDVAGNKLAKNVQYPEC
LDMQPYMSQQNTGPLVYVLYAVLVHAGWSCHDGHYFSYVKAQEGQWYKMDDAEVTVCSIT
SVLSQQAYVLFYIQKSEWERHSESVSRGREPRALGAEDTDRRAKQGELKRDHPCLQAPEL
DEHLVERATQESTLDHWKFLQEQNKTKPEFNVGKVEGTLPPNALVIHQSKYKCGMKNHHP
EQQSSLLNLSSTTRTDQESMNTGTLASLQGRTRRAKGKNKHSKRALLVCQ
>sp|D6RA61|U17LM_HUMAN Ubiquitin carboxyl-terminal hydrolase 17-like protein 22 OS=Homo sapiens OX=9606 GN=USP17L22 PE=3 SV=1
MEDDSLYLGGEWQFNHFSKLTSSRPDAAFAEIQRTSLPEKSPLSCETRVDLCDDLAPVAR
QLAPREKLPLSSRRPAAVGAGLQNMGNTCYVNASLQCLTYTPPLANYMLSREHSQTCHRH
KGCMLCTMQAHITRALHNPGHVIQPSQALAAGFHRGKQEDAHEFLMFTVDAMKKACLPGH
KQVDHHSKDTTLIHQIFGGYWRSQIKCLHCHGISDTFDPYLDIALDIQAAQSVQQALEQL
VKPEELNGENAYHCGVCLQRAPASKTLTLHTSAKVLILVLKRFSDVTGNKIAKNVQYPEC
LDMQPYMSQQNTGPLVYVLYAVLVHAGWSCHNGHYFSYVKAQEGQWYKMDDAEVTASSIT
SVLSQQAYVLFYIQKSEWERHSESVSRGREPRALGAEDTDRRATQGELKRDHPCLQAPEL
DEHLVERATQESTLDHWKFLQEQNKTKPEFNVRKVEGTLPPDVLVIHQSKYKCGMKNHHP

EQQSSLLKLSSTTPTHQESMNTGTLASLRGRARRSKGKNKHSKRALLVCQ
>sp|D6RCP7|U17LJ_HUMAN Ubiquitin carboxyl-terminal hydrolase 17-like protein 19 OS=Homo sapiens OX=9606 GN=USP17L19 PE=3 SV=1
MEEDSLYLGGEWQFNHFSKLTSSRPDAAFAEIQRTSLPEKSPLSCETRVDLCDDLAPVAR
QLAPREKLPLSSRRPAAVGAGLQNMGNTCYVNASLQCLTYTPPLANYMLSREHSQTCHRH
KGCMLCTMQAHITRALHNPGHVIQPSQALAAGFHRGKQEDAHEFLMFTVDAMKKACLPGH
KQVDHHSKDTTLIHQIFGGYWRSQIKCLHCHGISDTFDPYLDIALDIQAAQSVQQALEQL
VKPEELNGENAYHCGVCLQRAPASKTLTLHTSAKVLILVLKRFSDVTGNKIAKNVQYPEC
LDMQPYMSQTNTGPLVYVLYAVLVHAGWSCHNGHYFSYVKAQEGQWYKMDDAEVTASSIT
SVLSQQAYVLFYIQKSEWERHSESVSRGREPRALGAEDTDRRATQGELKRDHPCLQAPEL
DEHLVERATQESTLDHWKFLQEQNKTKPEFNVRKVEGTLPPDVLVIHQSKYKCGMKNHHP
EQQSSLLKLSSTTPTHQESMNTGTLASLRGRARRSKGKNKHSKRALLVCQ
>sp|D6RBQ6|U17LH_HUMAN Ubiquitin carboxyl-terminal hydrolase 17-like protein 17 OS=Homo sapiens OX=9606 GN=USP17L17 PE=3 SV=1
MEDDSLYLGGEWQFNHFSKLTSSRPDAAFAEIQRTSLPEKSPLSCETRVDLCDDLAPVAR
QLAPREKLPLSSRRPAAVGAGLQNMGNTCYVNASLQCLTYTPPLANYMLSREHSQTCHRH
KGCMLCTMQAHITRALHNPGHVIQPSQALAAGFHRGKQEDAHEFLMFTVDAMKKACLPGH
KQVDHHSKDTTLIHQIFGGYWRSQIKCLHCHGISDTFDPYLDIALDIQAAQSVQQALEQL
VKPEELNGENAYHCGVCLQRAPASKTLTLHTSAKVLILVLKRFSDVTGNKIAKNVQYPEC
LDMQPYMSQQNTGPLVYVLYAVLVHAGWSCHNGHYFSYVKAQEGQWYKMDDAEVTAASIT
SVLSQQAYVLFYIQKSEWERHSESVSRGREPRALGAEDTDRRATQGELKRDHPCLQAPEL
DEHLVERATQESTLDHWKFLQEQNKTKPEFNVRKVEGTLPPDVLVIHQSKYKCGMKNHHP
EQQSSLLNLSSSTPTHQESMNTGTLASLRGRARRSKGKNKHSKRALLVCQ
>sp|C9JVI0|U17LB_HUMAN Ubiquitin carboxyl-terminal hydrolase 17-like protein 11 OS=Homo sapiens OX=9606 GN=USP17L11 PE=3 SV=1
MEDDSLYLGGEWQFNHFSKLTSSRPDAAFAEIQRTSLPEKSPLSCETRVDLCDDLAPVAR
QLAPREKLPLSSRRPAAVGAGLQNMGNTCYVNASLQCLTYTPPLANYMLSREHSQTCHRH
KGCMLCTMQAHITRALHNPGHVIQPSQALAAGFHRGKQEDAHEFLMFTVDAMKKACLPGH
KQVDHHSKDTTLIHQIFGGYWRSQIKCLHCHGISDTFDPYLDIALDIQAAQSVQQALEQL
VKPEELNGENAYHCGVCLQRAPASKTLTLHTSAKVLILVLKRFSDVTGNKIAKNVQYPEC
LDMQPYMSQTNTGPLVYVLYAVLVHAGWSCHNGHYFSYVKAQEGQWYKMDDAEVTASSIT
SVLSQQAYVLFYIQKSEWERHSESVSRGREPRALGAEDTDRRATQGELKRDHPCLQAPEL
DEHLVERATQESTLDHWKFLQEQNKTKPEFNVRKVEGTLPPDVLVIHQSKYKCGMKNHHP
EQQSSLLNLSSTTPTHQESMNTGTLASLRGRARRSKGKNKHSKRALLVCQ
>sp|A8MUK1|U17L5_HUMAN Ubiquitin carboxyl-terminal hydrolase 17-like protein 5 OS=Homo sapiens OX=9606 GN=USP17L5 PE=3 SV=2
MEDDSLYLRGEWQFNHFSKLTSSRPDAAFAEIQRTSLPEKSPLSCETRVDLCDDLAPVAR
QLAPREKLPLSSRRPAAVGAGLQNMGNTCYVNASLQCLTYTPPLANYMLSREHSQTCHRH
KGCMLCTMQAHITRALHNPGHVIQPSQALAAGFHRGKQEDAHEFLMFTVDAMKKACLPGH
KQVDHHSKDTTLIHQIFGGYWRSQIKCLHCHGISDTFDPYLDIALDIQAAQSVQQALEQL
AKPEELNGENAYHCGVCLQRAPASKTLTLHTSAKVLILVLKRFSDVTGNKIAKNVQYPEC
LDMQPYMSQPNTGPLVYVLYAVLVHAGWSCHNGHYFSYVKAQEGQWYKMDDAEVTASSIT
SVLSQQAYVLFYIQKSEWERHSESVSRGREPRALGAEDTDRRATQGELKRDHPCLQAPEL
DEHLVERATQESTLDHWKFLQEQNKTKPEFNVRKVEGTLPPDVLVIHQSKYKCGMKNHHP
EQQSSLLNLSSSTPTHQESMNTGTLASLRGRARRSKGKNKHSKRALLVCQ
>sp|D6RJB6|U17LK_HUMAN Ubiquitin carboxyl-terminal hydrolase 17-like protein 20 OS=Homo sapiens OX=9606 GN=USP17L20 PE=3 SV=1

MEDDSLYLGGEWQFNHFSKLTSSRPDAAFAEIQRTSLPEKSPLSCETRVDLCDDLAPVAR
QLAPREKLPLSSRRPAAVGAGLQNMGNTCYVNASLQCLTYTPPLANYMLSREHSQTCHRH
KGCMLCTMQAHITRALHNPGHVIQPSQALAAGFHRGKQEDAHEFLMFTVDAMKKACLPGH
KQVDHHSKDTTLIHQIFGGYWRSQIKCLHCHGISDTFDPYLDIALDIQAAQSVQQALEQL
VKPEELNGENAYHCGVCLQRAPASKTLTLHTSAKVLILVLKRFSDVTGNKIAKNVQYPEC
LDMQPYMSQPNTGPLVYVLYAVLVHAGWSCHNGHYFSYVKAQEGQWYKMDDAEVTASSIT
SVLSQQAYVLFYIQKSEWERHSESVSRGREPRALGAEDTDRRATQGELKRDHPCLQAPEL
DEHLVERATQESTLDHWKFLQEQNKTKPEFNVRKVEGTLPPDVLVIHQSKYKCGMKNHHP
EQQSSLLNLSSTTPTHQESMNTGTLASLRGRARRSKGKNKHSKRALLVCQ
>sp|D6R9N7|U17LI_HUMAN Ubiquitin carboxyl-terminal hydrolase 17-like protein 18 OS=Homo sapiens OX=9606 GN=USP17L18 PE=3 SV=1
MEDDSLYLGGEWQFNHFSKLTSSRPDAAFAEIQRTSLPEKSPLSCETRVDLCDDLAPVAR
QLAPREKLPLSSRRPAAVGAGLQNMGNTCYVNASLQCLTYTPPLANYMLSREHSQTCHRH
KGCMLCTMQAHITRALHNPGHVIQPSQALAAGFHRGKQEDAHEFLMFTVDAMKKACLPGH
KQVDHHSKDTTLIHQIFGGYWRSQIKCLHCHGISDTFDPYLDIALDIQAAQSVQQALEQL
VKPEELNGENAYHCGVCLQRAPASKTLTLHTSAKVLILVLKRFSDVTGNKIAKNVQYPEC
LDMQPYMSQTNTGPLVYVLYAVLVHAGWSCHNGHYFSYVKAQEGQWYKMDDAEVTASSIT
SVLSQQAYVLFYIQKSEWERHSESVSRGREPRALGAEDTDRRAKQGELKRDHPCLQAPEL
DEHLVERATQESTLDHWKFLQEQNKTKPEFNVRKVEGTLPPDVLVIHQSKYKCGMKNHHP
EQQSSLLNLSSTTPTHQESMNTGTLASLRGRARRSKGKNKHSKRALLVCQ
>sp|C9J2P7|U17LF_HUMAN Ubiquitin carboxyl-terminal hydrolase 17-like protein 15 OS=Homo sapiens OX=9606 GN=USP17L15 PE=3 SV=2
MEDDSLYLGGEWQFNHFSKLTSSRPDAAFAEIQRTSLPEKSPLSCETRVDLCDDLAPVAR
QLAPREKLPLSSRRPAAVGAGLQNMGNTCYVNASLQCLTYTPPLANYMLSREHSQTCHRH
KGCMLCTMQAHITRALHNPGHVIQPSQALAAGFHRGKQEDAHEFLMFTVDAMKKACLPGH
KQVDHHSKDTTLIHQIFGGYWRSQIKCLHCHGISDTFDPYLDIALDIQAAQSVQQALEQL
VKPEELNGENAYHCGVCLQRAPASKTLTLHTSAKVLILVLKRFSDVTGNKIDKNVQYPEC
LDMKLYMSQTNSGPLVYVLYAVLVHAGWSCHNGHYFSYVKAQEGQWYKMDDAEVTASSIT
SVLSQQAYVLFYIQKSEWERHSESVSRGREPRALGAEDTDRRATQGELKRDHPCLQAPEL
DEHLVERATQESTLDHWKFLQEQNKTKPEFNVRKVEGTLPPDVLVIHQSKYKCGMKNHHP
EQQSSLLNLSSTTPTHQESMNTGTLASLRGRARRSKGKNKHSKRALLVCQWSQWKYRPTR
RGAHTHAHTQTHT
>sp|Q0WX57|U17LO_HUMAN Ubiquitin carboxyl-terminal hydrolase 17-like protein 24 OS=Homo sapiens OX=9606 GN=USP17L30 PE=1 SV=2
MEDDSLYLRGEWQFNHFSKLTSSRPDAAFAEIQRTSLPEKSPLSCETRVDLCDDLAPVAR
QLAPREKLPLSSRRPAAVGAGLQNMGNTCYVNASLQCLTYTPPLANYMLSREHSQTCHRH
KGCMLCTMQAHITRALHNPGHVIQPSQALAAGFHRGKQEDAHEFLMFTVDAMKKACLPGH
KQVDHHSKDTTLIHQIFGGYWRSQIKCLHCHGISDTFDPYLDIALDIQAAQSVQQALEQL
VKPEELNGENAYHCGVCLQRAPASKTLTLHTSAKVLILVLKRFSDVTGNKIAKNVQYPEC
LDMQPYMSQPNTGPLVYVLYAVLVHAGWSCHNGHYFSYVKAQEGQWYKMDDAEVTASSIT
SVLSQQAYVLFYIQKSEWERHSESVSRGREPRALGAEDTDRRATQGELKRDHPCLQAPEL
DEHLVERATQESTLDHWKFLQEQNKTKPEFNVRKVEGTLPPDVLVIHQSKYKCGMKNHHP
EQQSSLLNLSSSTPTHQESMNTGTLASLRGRARRSKGKNKHSKRALLVCQ
>sp|C9JLJ4|U17LD_HUMAN Ubiquitin carboxyl-terminal hydrolase 17-like protein 13 OS=Homo sapiens OX=9606 GN=USP17L13 PE=3 SV=1
MEEDSLYLGGEWQFNHFSKLTSSRLDAAFAEIQRTSLPEKSPLSCETRVDLCDDLVPEAR
QLAPREKLPLSSRRPAAVGAGLQNMGNTCYVNASLQCLTYTPPLANYMLSREHSQTCHRH

KGCMLCTMQAHITRALHNPGHVIQPSQALAAGFHRGKQEDAHEFLMFTVDAMKKACLPGH
KQVDHPSKDTTLIHQIFGGYWRSQIKCLHCHGISDTFDPYLDIALDIQAAQSVQQALEQL
VKPEELNGENAYHCGVCLQRAPASKTLTLHTSAKVLILVLKRFSDVTGNKIAKNVQYPEC
LDMQPYMSQQNTGPLVYVLYAVLVHAGWSCHNGHYFSYVKAQEGQWYKMDDAEVTAASIT
SVLSQQAYVLFYIQKSEWERHSESVSRGREPRALGAEDTDRRATQGELKRDHPCLQAPEL
DEHLVERATQESTLDRWKFLQEQNKTKPEFNVRKVEGTLPPDVLVIHQSKYKCGMKNHHP
EQQSSLLNLSSSTPTHQESMNTGTLASLRGRARRSKGKNKHSKRALLVCQ
>sp|D6R901|U17LL_HUMAN Ubiquitin carboxyl-terminal hydrolase 17-like protein 21 OS=Homo sapiens OX=9606 GN=USP17L21 PE=3 SV=1
MEEDSLYLGGEWQFNHFSKLTSSRPDAAFAEIQRTSLPEKSPLSCETRVDLCDDLAPVAR
QLAPREKLPLSNRRPAAVGAGLQNMGNTCYVNASLQCLTYTPPLANYMLSREHSQTCHRH
KGCMLCTMQAHITRALHNPGHVIQPSQALAAGFHRGKQEDAHEFLMFTVDAMKKACLPGH
KQVDHHSKDTTLIHQIFGGYWRSQIKCLHCHGISDTFDPYLDIALDIQAAQSVQQALEQL
VKPEELNGENAYHCGVCLQRAPASKMLTLLTSAKVLILVLKRFSDVTGNKIAKNVQYPEC
LDMQPYMSQPNTGPLVYVLYAVLVHAGWSCHNGHYFSYVKAQEGQWYKMDDAEVTASSIT
SVLSQQAYVLFYIQKSEWERHSESVSRGREPRALGAEDTDRRATQGELKRDHPCLQAPEL
DEHLVERATQESTLDHWKFLQEQNKTKPEFNVRKVEGTLPPDVLVIHQSKYKCGMKNHHP
EQQSSLLNLSSSTPTHQESMNTGTLASLRGRARRSKGKNKHSKRALLVCQ
>sp|C9JPN9|UL17C_HUMAN Ubiquitin carboxyl-terminal hydrolase 17-like protein 12 OS=Homo sapiens OX=9606 GN=USP17L12 PE=3 SV=1
MEEDSLYLGGEWQFNHFSKLTSSRPDAAFAEIQRTSLPEKSPLSCETRVDLCDDLAPVAR
QLAPREKLPLSNRRPAAVGAGLQNMGNTCYVNASLQCLTYTPPLANYMLSREHSQTCHRH
KGCMLCTMQAHITRALHNPGHVIQPSQALAAGFHRGKQEDAHEFLMFTVDAMKKACLPGH
KQVDHHSKDTTLIHQIFGGYWRSQIKCLHCHGISDTFDPYLDIALDIQAAQSVQQALEQL
VKPEELNGENAYHCGVCLQRAPASKMLTLLTSAKVLILVLKRFSDVTGNKIAKNVQYPEC
LDMQPYMSQPNTGPLVYVLYAVLVHAGWSCHNGHYFSYVKAQEGQWYKMDDAEVTASSIT
SVLSQQAYVLFYIQKSEWERHSESVSRGREPRALGAEDTDRRATQGELKRDHPCLQAPEL
DEHLVERATQESTLDHWKFLQEQNKTKPEFNVRKVEGTLPPDVLVIHQSKYKCGMKNHHP
EQQSSLLKLSSTTPTHQESMNTGTLASLRGRARRSKGKNKHSKRALLVCQ
>sp|O15067|PUR4_HUMAN Phosphoribosylformylglycinamidine synthase OS=Homo sapiens OX=9606 GN=PFAS PE=1 SV=4
MSPVLHFYVRPSGHEGAAPGHTRRKLQGKLPELQGVETELCYNVNWTAEALPSAEETKKL
MWLFGCPLLLDDVARESWLLPGSNDLLLEVGPRLNFSTPTSTNIVSVCRATGLGPVDRVE
TTRRYRLSFAHPPSAEVEAIALATLHDRMTEQHFPHPIQSFSPESMPEPLNGPINILGEG
RLALEKANQELGLALDSWDLDFYTKRFQELQRNPSTVEAFDLAQSNSEHSRHWFFKGQLH
VDGQKLVHSLFESIMSTQESSNPNNVLKFCDNSSAIQGKEVRFLRPEDPTRPSRFQQQQG
LRHVVFTAETHNFPTGVCPFSGATTGTGGRIRDVQCTGRGAHVVAGTAGYCFGNLHIPGY
NLPWEDPSFQYPGNFARPLEVAIEASNGASDYGNKFGEPVLAGFARSLGLQLPDGQRREW
IKPIMFSGGIGSMEADHISKEAPEPGMEVVKVGGPVYRIGVGGGAASSVQVQGDNTSDLD
FGAVQRGDPEMEQKMNRVIRACVEAPKGNPICSLHDQGAGGNGNVLKELSDPAGAIIYTS
RFQLGDPTLNALEIWGAEYQESNALLLRSPNRDFLTHVSARERCPACFVGTITGDRRIVL
VDDRECPVRRNGQGDAPPTPLPTPVDLELEWVLGKMPRKEFFLQRKPPMLQPLALPPGLS
VHQALERVLRLPAVASKRYLTNKVDRSVGGLVAQQQCVGPLQTPLADVAVVALSHEELIG
AATALGEQPVKSLLDPKVAARLAVAEALTNLVFALVTDLRDVKCSGNWMWAAKLPGEGAA
LADACEAMVAVMAALGVAVDGGKDSLSMAARVGTETVRAPGSLVISAYAVCPDITATVTP
DLKHPEGRGHLLYVALSPGQHRLGGTALAQCFSQLGEHPPDLDLPENLVRAFSITQGLLK
DRLLCSGHDVSDGGLVTCLLEMAFAGNCGLQVDVPVPRVDVLSVLFAEEPGLVLEVQEPD

LAQVLKRYRDAGLHCLELGHTGEAGPHAMVRVSVNGAVVLEEPVGELRALWEETSFQLDR
LQAEPRCVAEEERGLRERMGPSYCLPPTFPKASVPREPGGPSPRVAILREEGSNGDREMA
DAFHLAGFEVWDVTMQDLCSGAIGLDTFRGVAFVGGFSYADVLGSAKGWAAAVTFHPRAG
AELRRFRKRPDTFSLGVCNGCQLLALLGWVGGDPNEDAAEMGPDSQPARPGLLLRHNLSG
RYESRWASVRVGPGPALMLRGMEGAVLPVWSAHGEGYVAFSSPELQAQIEARGLAPLHWA
DDDGNPTEQYPLNPNGSPGGVAGICSCDGRHLAVMPHPERAVRPWQWAWRPPPFDTLTTS
PWLQLFINARNWTLEGSC
>sp|Q5JTV8|TOIP1_HUMAN Torsin-1A-interacting protein 1 OS=Homo sapiens OX=9606 GN=TOR1AIP1 PE=1 SV=2
MAGDGRRAEAVREGWGVYVTPRAPIREGRGRLAPQNGGSSDAPAYRTPPSRQGRREVRFS
DEPPEVYGDFEPLVAKERSPVGKRTRLEEFRSDSAKEEVRESAYYLRSRQRRQPRPQETE
EMKTRRTTRLQQQHSEQPPLQPSPVMTRRGLRDSHSSEEDEASSQTDLSQTISKKTVRSI
QEAPVSEDLVIRLRRPPLRYPRYEATSVQQKVNFSEEGETEEDDQDSSHSSVTTVKARSR
DSDESGDKTTRSSSQYIESFWQSSQSQNFTAHDKQPSVLSSGYQKTPQEWAPQTARIRTR
MQNDSILKSELGNQSPSTSSRQVTGQPQNASFVKRNRWWLLPLIAALASGSFWFFSTPEV
ETTAVQEFQNQMNQLKNKYQGQDEKLWKRSQTFLEKHLNSSHPRSQPAILLLTAARDAEE
ALRCLSEQIADAYSSFRSVRAIRIDGTDKATQDSDTVKLEVDQELSNGFKNGQNAAVVHR
FESFPAGSTLIFYKYCDHENAAFKDVALVLTVLLEEETLGTSLGLKEVEEKVRDFLKVKF
TNSNTPNSYNHMDPDKLNGLWSRISHLVLPVQPENALKRGICL
>sp|Q5VZ46|K1614_HUMAN Uncharacterized protein KIAA1614 OS=Homo sapiens OX=9606 GN=KIAA1614 PE=2 SV=3
MEGTEAAAAKPAGGSPQGPKTGSGTASPVEGTSAVEWSGPEPQLDNGHPPRPWPCPQENR
TSSLMAPQPPRVWGVQLQGPSVLESKVRALKEKMTVAKQGVSPCSASQEWSSPKKPQCRR
GKAGRAGTPSEGSFLPGAVVAPRTQNLPDGQLDGSINEEQPARDGGPRLPRPPAPGREYC
NRGSPWPPEAEWTLPDHDRGPLLGPSSLQQSPIHGVTPGRPGGPGHCNKIIHIPSPRTGR
SYPFPDGVVTEADLDSTSLTSEEVFVPRTALLGERWRAGDLEALGAGSSVLSLSDRVERN
RLLLQEMLNVSGQSPRKVGTPAWTPSWDTAAPERPVGDVDWASGTSLQDSGQNRTVGPNP
EPVLSPRHEEATHLLQRARMKARTRPLRASHDIVPTITQGSRDGHRSPARDPRTTPACRD
SLQNGHTSDSSSGESSGGHRPRRGPSPSHVRFEDESAREAEFRHLERLQQRQRQVLSTVL
QAADQGPLRSKPDLADYINGAPRLRDAGQGTFHRLVGSLDRRGHPAPPAPGSERRCQACG
SCIDDPRPAQGKAPPVPRTLQELQAACGMERVLGGLSSPLRLLPAEPRLHMEWIRETHIG
DTVCPAEVDSALDSTDNSDNCRTDSEEAGTSQAGWACGRTQGSSPRLRLRGSRPRGHRWS
KKAEAELPWGLQAQQHLPRADDVEVENEVKEGRGHTPEGTLFLREDAKPPDLELKRVSLG
PQWQPGPGLGSHQPHPLDSRTPCRTAYATTAPMTPESSGPGGQAQVTESHESLEIVSPSS
LQQSHAEPSAPHQAWQPTASLCPEGWAPTPPPSRKTTSPVSHRKAALAGLLRLGDQTEPV
GIPRPPSRSAVLRTCELPPSQTQPSRPQVRHPLLALSTNNCNNSAPRGLQEPYGGAVHEG
RVERGPCSREPEPPLENSRDGGPQGFLGSADVATINSTGITLSLSSEESESSKESEGSLQ
RTGSGSGGHVLSRASAGAGTGPGSPSAAPLDQNKKRSSSIASTLGLKKLFSALGQSSRPK
LGKSRSYSVEQLQPAPPGLTSQSRAPSLQSLHPVSPSHQRRKAASFQNLHSLLSSKGNRS
SLYLVAGPGDHSAAGRPAKTSPRRALSVEDVGAPSLARTVGRLVEVFPDGTSQLQLQRSP
GGTFGFCVASGNGRPDSGMPSPLPQPHGWGGLSKQGRAFWLWSEAFLVFG
>sp|Q8IVF1|NTM2A_HUMAN NUT family member 2A OS=Homo sapiens OX=9606 GN=NUTM2A PE=2 SV=4
MEVKGPSGRSFCCESEGQFKSCLKRHTPSLLLPSSWKGNSGSCLMAKALHRMSPTPNSCP
LPLPLCRMSGVLCSRNLFTFKFSLFQLDSGASGEPGHSLGLTLGFSHCGNCQTAVVSAQP
EGMASNGAYPALGPGVTANPGTSLSVFTALPFTTPAPGPAHGPLLVTAGAPPGGPLVLST
LPSTPLVTEQDGCGPSGAGASNVFVQMRTEVGPVKAAQAQTLVLTQAPLVWQAPGALCGG

VVCPPPLLLAAAPVVPVMAAQVVGGTQACEGGWSQGLPLPPPPPPAAQLPPIVSQGNAGP
WPQGAHGEGSLASSQAKAPPDDSCNPRSVYENFRLWQHYKPLARRHLPQSPDTEALSCFL
IPVLRSLARRKPTMTLEEGLWRAMREWQHTSNFDRMIFYEMAEKFLEFEAEEEMQIQKSQ
WMKGPQCLPPPATPRLEPRGPPAPEVVKQPVYLPSKAGPKAPTACLPPPRPQRPVTKARR
PPPRPHRRAETKARLPPPRPQRPAETKVPEEIPPEVVQEYVDIMEELLGPSLGATGEPEK
QREEGEVKQPQEEDWTPPDPGLLSYTDKLCSQKDFVTKVEAVIHPQFLEELLSPDPQMDF
LALSQELEQEEGLTLAQLVEKRLLPLKEKQHARAAPSRGTARLDSSSSKFAAGQGAERDV
PVPQQGVGMETCPPQTTARDSQGRGRAHTGMARSKDSVVLLGCQDSPGLRAARPTSPPQD
HRPTCPGVGTKDALDLPGGSPVRESHGLAQGSSEEEELPSLAFLLGSQHKLLPWWLPQSP
VPASGLLSPEKWGPQGTHQFPSAERRGLNLAPSPANKAKKRPLFGSLSPAEKTPHPGPGL
RVSGEQSLTWGLGGPSQSQKRKGDPLVSRKEKKQRCSQ
>sp|Q92954|PRG4_HUMAN Proteoglycan 4 OS=Homo sapiens OX=9606 GN=PRG4 PE=1 SV=3
MAWKTLPIYLLLLLSVFVIQQVSSQDLSSCAGRCGEGYSRDATCNCDYNCQHYMECCPDF
KRVCTAELSCKGRCFESFERGRECDCDAQCKKYDKCCPDYESFCAEVHNPTSPPSSKKAP
PPSGASQTIKSTTKRSPKPPNKKKTKKVIESEEITEEHSVSENQESSSSSSSSSSSSTIR
KIKSSKNSAANRELQKKLKVKDNKKNRTKKKPTPKPPVVDEAGSGLDNGDFKVTTPDTST
TQHNKVSTSPKITTAKPINPRPSLPPNSDTSKETSLTVNKETTVETKETTTTNKQTSTDG
KEKTTSAKETQSIEKTSAKDLAPTSKVLAKPTPKAETTTKGPALTTPKEPTPTTPKEPAS
TTPKEPTPTTIKSAPTTPKEPAPTTTKSAPTTPKEPAPTTTKEPAPTTPKEPAPTTTKEP
APTTTKSAPTTPKEPAPTTPKKPAPTTPKEPAPTTPKEPTPTTPKEPAPTTKEPAPTTPK
EPAPTAPKKPAPTTPKEPAPTTPKEPAPTTTKEPSPTTPKEPAPTTTKSAPTTTKEPAPT
TTKSAPTTPKEPSPTTTKEPAPTTPKEPAPTTPKKPAPTTPKEPAPTTPKEPAPTTTKKP
APTTPKEPAPTTPKETAPTTPKKLTPTTPEKLAPTTPEKPAPTTPEELAPTTPEEPTPTT
PEEPAPTTPKAAAPNTPKEPAPTTPKEPAPTTPKEPAPTTPKETAPTTPKGTAPTTLKEP
APTTPKKPAPKELAPTTTKEPTSTTSDKPAPTTPKGTAPTTPKEPAPTTPKEPAPTTPKG
TAPTTLKEPAPTTPKKPAPKELAPTTTKGPTSTTSDKPAPTTPKETAPTTPKEPAPTTPK
KPAPTTPETPPPTTSEVSTPTTTKEPTTIHKSPDESTPELSAEPTPKALENSPKEPGVPT
TKTPAATKPEMTTTAKDKTTERDLRTTPETTTAAPKMTKETATTTEKTTESKITATTTQV
TSTTTQDTTPFKITTLKTTTLAPKVTTTKKTITTTEIMNKPEETAKPKDRATNSKATTPK
PQKPTKAPKKPTSTKKPKTMPRVRKPKTTPTPRKMTSTMPELNPTSRIAEAMLQTTTRPN
QTPNSKLVEVNPKSEDAGGAEGETPHMLLRPHVFMPEVTPDMDYLPRVPNQGIIINPMLS
DETNICNGKPVDGLTTLRNGTLVAFRGHYFWMLSPFSPPSPARRITEVWGIPSPIDTVFT
RCNCEGKTFFFKDSQYWRFTNDIKDAGYPKPIFKGFGGLTGQIVAALSTAKYKNWPESVY
FFKRGGSIQQYIYKQEPVQKCPGRRPALNYPVYGETTQVRRRRFERAIGPSQTHTIRIQY
SPARLAYQDKGVLHNEVKVSILWRGLPNVVTSAISLPNIRKPDGYDYYAFSKDQYYNIDV
PSRTARAITTRSGQTLSKVWYNCP
>sp|Q15413|RYR3_HUMAN Ryanodine receptor 3 OS=Homo sapiens OX=9606 GN=RYR3 PE=1 SV=3
MAEGGEGGEDEIQFLRTEDEVVLQCIATIHKEQRKFCLAAEGLGNRLCFLEPTSEAKYIP
PDLCVCNFVLEQSLSVRALQEMLANTGENGGEGAAQGGGHRTLLYGHAVLLRHSFSGMYL
TCLTTSRSQTDKLAFDVGLREHATGEACWWTIHPASKQRSEGEKVRIGDDLILVSVSSER
YLHLSVSNGNIQVDASFMQTLWNVHPTCSGSSIEEGYLLGGHVVRLFHGHDECLTIPSTD
QNDSQHRRIFYEAGGAGTRARSLWRVEPLRISWSGSNIRWGQAFRLRHLTTGHYLALTED
QGLILQDRAKSDTKSTAFSFRASKELKEKLDSSHKRDIEGMGVPEIKYGDSVCFVQHIAS
GLWVTYKAQDAKTSRLGPLKRKVILHQEGHMDDGLTLQRCQREESQAARIIRNTTALFSQ
FVSGNNRTAAPITLPIEEVLQTLQDLIAYFQPPEEEMRHEDKQNKLRSLKNRQNLFKEEG

MLALVLNCIDRLNVYNSVAHFAGIAREESGMAWKEILNLLYKLLAALIRGNRNNCAQFSN
NLDWLISKLDRLESSSGILEVLHCILTESPEALNLIAEGHIKSIISLLDKHGRNHKVLDI
LCSLCLCNGVAVRANQNLICDNLLPRRNLLLQTRLINDVTSIRPNIFLGVAEGSAQYKKW
YFELIIDQVDPFLTAEPTHLRVGWASSSGYAPYPGGGEGWGGNGVGDDLYSYGFDGLHLW
SGRIPRAVASINQHLLRSDDVVSCCLDLGVPSISFRINGQPVQGMFENFNTDGLFFPVMS
FSAGVKVRFLMGGRHGEFKFLPPSGYAPCYEALLPKEKMRLEPVKEYKRDADGIRDLLGT
TQFLSQASFIPCPVDTSQVILPPHLEKIRDRLAENIHELWGMNKIELGWTFGKIRDDNKR
QHPCLVEFSKLPETEKNYNLQMSTETLKTLLALGCHIAHVNPAAEEDLKKVKLPKNYMMS
NGYKPAPLDLSDVKLLPPQEILVDKLAENAHNVWAKDRIKQGWTYGIQQDLKNKRNPRLV
PYALLDERTKKSNRDSLREAVRTFVGYGYNIEPSDQELADSAVEKVSIDKIRFFRVERSY
AVRSGKWYFEFEVVTGGDMRVGWARPGCRPDVELGADDQAFVFEGNRGQRWHQGSGYFGR
TWQPGDVVGCMINLDDASMIFTLNGELLITNKGSELAFADYEIENGFVPICCLGLSQIGR
MNLGTDASTFKFYTMCGLQEGFEPFAVNMNRDVAMWFSKRLPTFVNVPKDHPHIEVMRID
GTMDSPPCLKVTHKTFGTQNSNADMIYCRLSMPVECHSSFSHSPCLDSEAFQKRKQMQEI
LSHTTTQCYYAIRIFAGQDPSCVWVGWVTPDYHLYSEKFDLNKNCTVTVTLGDERGRVHE
SVKRSNCYMVWGGDIVASSQRSNRSNVDLEIGCLVDLAMGMLSFSANGKELGTCYQVEPN
TKVFPAVFLQPTSTSLFQFELGKLKNAMPLSAAIFRSEEKNPVPQCPPRLDVQTIQPVLW
SRMPNSFLKVETERVSERHGWVVQCLEPLQMMALHIPEENRCVDILELCEQEDLMRFHYH
TLRLYSAVCALGNSRVAYALCSHVDLSQLFYAIDNKYLPGLLRSGFYDLLISIHLASAKE
RKLMMKNEYIIPITSTTRNIRLFPDESKRHGLPGVGLRTCLKPGFRFSTPCFVVTGEDHQ
KQSPEIPLESLRTKALSMLTEAVQCSGAHIRDPVGGSVEFQFVPVLKLIGTLLVMGVFDD
DDVRQILLLIDPSVFGEHSAGTEEGAEKEEVTQVEEKAVEAGEKAGKEAPVKGLLQTRLP
ESVKLQMCELLSYLCDCELQHRVEAIVAFGDIYVSKLQANQKFRYNELMQALNMSAALTA
RKTKEFRSPPQEQINMLLNFQLGENCPCPEEIREELYDFHEDLLLHCGVPLEEEEEEED
TSWTGKLCALVYKIKGPPKPEKEQPTEEEERCPTTLKELISQTMICWAQEDQIQDSELVR
MMFNLLRRQYDSIGELLQALRKTYTISHTSVSDTINLLAALGQIRSLLSVRMGKEEELLM
INGLGDIMNNKVFYQHPNLMRVLGMHETVMEVMVNVLGTEKSQIAFPKMVASCCRFLCYF
CRISRQNQKAMFEHLSYLLENSSVGLASPSMRGSTPLDVAASSVMDNNELALSLEEPDLE
KVVTYLAGCGLQSCPMLLAKGYPDVGWNPIEGERYLSFLRFAVFVNSESVEENASVVVKL
LIRRPECFGPALRGEGGNGLLAAMQGAIKISENPALDLPSQGYKREVSTGDDEEEEEIVH
MGNAIMSFYSALIDLLGRCAPEMHLIQTGKGEAIRIRSILRSLVPTEDLVGIISIPLKLP
SLNKDGSVSEPDMAANFCPDHKAPMVLFLDRVYGIKDQTFLLHLLEVGFLPDLRASASLD
TVSLSTTEAALALNRYICSAVLPLLTRCAPLFAGTEHCTSLIDSTLQTIYRLSKGRSLTK
AQRDTIEECLLAICNHLRPSMLQQLLRRLVFDVPQLNEYCKMPLKLLTNHYEQCWKYYCL
PSGWGSYGLAVEEELHLTEKLFWGIFDSLSHKKYDPDLFRMALPCLSAIAGALPPDYLDT
RITATLEKQISVDADGNFDPKPINTMNFSLPEKLEYIVTKYAEHSHDKWACDKSQSGWKY
GISLDENVKTHPLIRPFKTLTEKEKEIYRWPARESLKTMLAVGWTVERTKEGEALVQQRE
NEKLRSVSQANQGNSYSPAPLDLSNVVLSRELQGMVEVVAENYHNIWAKKKKLELESKGG
GSHPLLVPYDTLTAKEKFKDREKAQDLFKFLQVNGIIVSRGMKDMELDASSMEKRFAYKF
LKKILKYVDSAQEFIAHLEAIVSSGKTEKSPRDQEIKFFAKVLLPLVDQYFTSHCLYFLS
SPLKPLSSSGYASHKEKEMVAGLFCKLAALVRHRISLFGSDSTTMVSCLHILAQTLDTRT
VMKSGSELVKAGLRAFFENAAEDLEKTSENLKLGKFTHSRTQIKGVSQNINYTTVALLPI
LTSIFEHVTQHQFGMDLLLGDVQISCYHILCSLYSLGTGKNIYVERQRPALGECLASLAA
AIPVAFLEPTLNRYNPLSVFNTKTPRERSILGMPDTVEDMCPDIPQLEGLMKEINDLAES
GARYTEMPHVIEVILPMLCNYLSYWWERGPENLPPSTGPCCTKVTSEHLSLILGNILKII
NNNLGIDEASWMKRIAVYAQPIISKARPDLLRSHFIPTLEKLKKKAVKTVQEEEQLKADG
KGDTQEAELLILDEFAVLCRDLYAFYPMLIRYVDNNRSNWLKSPDADSDQLFRMVAEVFI

LWCKSHNFKREEQNFVIQNEINNLAFLTGDSKSKMSKAMQVKSGGQDQERKKTKRRGDLY
SIQTSLIVAALKKMLPIGLNMCTPGDQELISLAKSRYSHRDTDEEVREHLRNNLHLQEKS
DDPAVKWQLNLYKDVLKSEEPFNPEKTVERVQRISAAVFHLEQVEQPLRSKKAVWHKLLS
KQRKRAVVACFRMAPLYNLPRHRSINLFLHGYQRFWIETEEYSFEEKLVQDLAKSPKVEE
EEEEETEKQPDPLHQIILYFSRNALTERSKLEDDPLYTSYSSMMAKSCQSGEDEEEDEDK
EKTFEEKEMEKQKTLYQQARLHERGAAEMVLQMISASKGEMSPMVVETLKLGIAILNGGN
AGVQQKMLDYLKEKKDAGFFQSLSGLMQSCSVLDLNAFERQNKAEGLGMVTEEGTLIVRE
RGEKVLQNDEFTRDLFRFLQLLCEGHNSDFQNFLRTQMGNTTTVNVIISTVDYLLRLQES
ISDFYWYYSGKDIIDESGQHNFSKALAVTKQIFNSLTEYIQGPCIGNQQSLAHSRLWDAV
VGFLHVFANMQMKLSQDSSQIELLKELLDLLQDMVVMLLSLLEGNVVNGTIGKQMVDTLV
ESSTNVEMILKFFDMFLKLKDLTSSDTFKEYDPDGKGIISKKEFQKAMEGQKQYTQSEID
FLLSCAEADENDMFNYVDFVDRFHEPAKDIGFNVAVLLTNLSEHMPNDSRLKCLLDPAES
VLNYFEPYLGRIEIMGGAKKIERVYFEISESSRTQWEKPQVKESKRQFIFDVVNEGGEQE
KMELFVNFCEDTIFEMQLASQISESDSADRPEEEEEDEDSSYVLEIAGEEEEDGSLEPAS
AFAMACASVKRNVTDFLKRATLKNLRKQYRNVKKMTAKELVKVLFSFFWMLFVGLFQLLF
TILGGIFQILWSTVFGGGLVEGAKNIRVTKILGDMPDPTQFGIHDDTMEAERAEVMEPGI
TTELVHFIKGEKGDTDIMSDLFGLHPKKEGSLKHGPEVGLGDLSEIIGKDEPPTLESTVQ
KKRKAQAAEMKAANEAEGKVESEKADMEDGEKEDKDKEEEQAEYLWTEVTKKKKRRCGQK
VEKPEAFTANFFKGLEIYQTKLLHYLARNFYNLRFLALFVAFAINFILLFYKVTEEPLEE
ETEDVANLWNSFNDEEEEEAMVFFVLQESTGYMAPTLRALAIIHTIISLVCVVGYYCLKV
PLVVFKREKEIARKLEFDGLYITEQPSEDDIKGQWDRLVINTPSFPNNYWDKFVKRKVIN
KYGDLYGAERIAELLGLDKNALDFSPVEETKAEAASLVSWLSSIDMKYHIWKLGVVFTDN
SFLYLAWYTTMSVLGHYNNFFFAAHLLDIAMGFKTLRTILSSVTHNGKQLVLTVGLLAVV
VYLYTVVAFNFFRKFYNKSEDDDEPDMKCDDMMTCYLFHMYVGVRAGGGIGDEIEDPAGD
PYEMYRIVFDITFFFFVIVILLAIIQGLIIDAFGELRDQQEQVREDMETKCFICGIGNDY
FDTTPHGFETHTLQEHNLANYLFFLMYLINKDETEHTGQESYVWKMYQERCWDFFPAGDC
FRKQYEDQLG
>sp|Q12772|SRBP2_HUMAN Sterol regulatory element-binding protein 2 OS=Homo sapiens OX=9606 GN=SREBF2 PE=1 SV=2
MDDSGELGGLETMETLTELGDELTLGDIDEMLQFVSNQVGEFPDLFSEQLCSSFPGSGGS
GSSSGSSGSSSSSSNGRGSSSGAVDPSVQRSFTQVTLPSFSPSAASPQAPTLQVKVSPTS
VPTTPRATPILQPRPQPQPQPQTQLQQQTVMITPTFSTTPQTRIIQQPLIYQNAATSFQV
LQPQVQSLVTSSQVQPVTIQQQVQTVQAQRVLTQTANGTLQTLAPATVQTVAAPQVQQVP
VLVQPQIIKTDSLVLTTLKTDGSPVMAAVQNPALTALTTPIQTAALQVPTLVGSSGTILT
TMPVMMGQEKVPIKQVPGGVKQLEPPKEGERRTTHNIIEKRYRSSINDKIIELKDLVMGT
DAKMHKSGVLRKAIDYIKYLQQVNHKLRQENMVLKLANQKNKLLKGIDLGSLVDNEVDLK
IEDFNQNVLLMSPPASDSGSQAGFSPYSIDSEPGSPLLDDAKVKDEPDSPPVALGMVDRS
RILLCVLTFLCLSFNPLTSLLQWGGAHDSDQHPHSGSGRSVLSFESGSGGWFDWMMPTLL
LWLVNGVIVLSVFVKLLVHGEPVIRPHSRSSVTFWRHRKQADLDLARGDFAAAAGNLQTC
LAVLGRALPTSRLDLACSLSWNVIRYSLQKLRLVRWLLKKVFQCRRATPATEAGFEDEAK
TSARDAALAYHRLHQLHITGKLPAGSACSDVHMALCAVNLAECAEEKIPPSTLVEIHLTA
AMGLKTRCGGKLGFLASYFLSRAQSLCGPEHSAVPDSLRWLCHPLGQKFFMERSWSVKSA
AKESLYCAQRNPADPIAQVHQAFCKNLLERAIESLVKPQAKKKAGDQEEESCEFSSALEY
LKLLHSFVDSVGVMSPPLSRSSVLKSALGPDIICRWWTSAITVAISWLQGDDAAVRSHFT
KVERIPKALEVTESPLVKAIFHACRAMHASLPGKADGQQSSFCHCERASGHLWSSLNVSG
ATSDPALNHVVQLLTCDLLLSLRTALWQKQASASQAVGETYHASGAELAGFQRDLGSLRR
LAHSFRPAYRKVFLHEATVRLMAGASPTRTHQLLEHSLRRRTTQSTKHGEVDAWPGQRER

ATAILLACRHLPLSFLSSPGQRAVLLAEAARTLEKVGDRRSCNDCQQMIVKLGGGTAIAA
S
>sp|O94822|LTN1_HUMAN E3 ubiquitin-protein ligase listerin OS=Homo sapiens OX=9606 GN=LTN1 PE=1 SV=6
MGGKNKQRTKGNLRPSNSGRAAELLAKEQGTVPGFIGFGTSQSDLGYVPAIQGAEEIDSL
VDSDFRMVLRKLSKKDVTTKLKAMQEFGTMCTERDTETVKGVLPYWPRIFCKISLDHDRR
VREATQQAFEKLILKVKKQLAPYLKSLMGYWLMAQCDTYTPAAFAAKDAFEAAFPPSKQP
EAIAFCKDEITSVLQDHLIKETPDTLSDPQTVPEEEREAKFYRVVTCSLLALKRLLCLLP
DNELDSLEEKFKSLLSQNKFWKYGKHSVPQIRSAYFELVSALCQRIPQLMKEEASKVSPS
VLLSIDDSDPIVCPALWEAVLYTLTTIEDCWLHVNAKKSVFPKLSTVIREGGRGLATVIY
PYLLPFISKLPQSITNPKLDFFKNFLTSLVAGLSTERTKTSSLESSAVISAFFECLRFIM
QQNLGEEEIEQMLVNDQLIPFIDAVLKDPGLQHGQLFNHLAETLSSWEAKADTEKDEKTA
HNLENVLIHFWERLSEICVAKISEPEADVESVLGVSNLLQVLQKPKSSLKSSKKKNGKVR
FADEILESNKENEKCVSSEGEKIEGWELTTEPSLTHNSSGLLSPLRKKPLEDLVCKLADI
SINYVNERKSEQHLRFLSTLLDSFSSSRVFKMLLGDEKQSIVQAKPLEIAKLVQKNPAVQ
FLYQKLIGWLNEDQRKDFGFLVDILYSALRCCDNDMERKKVLDDLTKVDLKWNSLLKIIE
KACPSSDKHALVTPWLKGDILGEKLVNLADCLCNEDLESRVSSESHFSERWTLLSLVLSQ
HVKNDYLIGDVYVERIIVRLHETLFKTKKLSEAESSDSSVSFICDVAYNYFSSAKGCLLM
PSSEDLLLTLFQLCAQSKEKTHLPDFLICKLKNTWLSGVNLLVHQTDSSYKESTFLHLSA
LWLKNQVQASSLDINSLQVLLSAVDDLLNTLLESEDSYLMGVYIGSVMPNDSEWEKMRQS
LPMQWLHRPLLEGRLSLNYECFKTDFKEQDIKTLPSHLCTSALLSKMVLIALRKETVLEN
NELEKIIAELLYSLQWCEELDNPPIFLIGFCEILQKMNITYDNLRVLGNTSGLLQLLFNR
SREHGTLWSLIIAKLILSRSISSDEVKPHYKRKESFFPLTEGNLHTIQSLCPFLSKEEKK
EFSAQCIPALLGWTKKDLCSTNGGFGHLAIFNSCLQTKSIDDGELLHGILKIIISWKKEH
EDIFLFSCNLSEASPEVLGVNIEIIRFLSLFLKYCSSPLAESEWDFIMCSMLAWLETTSE
NQALYSIPLVQLFACVSCDLACDLSAFFDSTTLDTIGNLPVNLISEWKEFFSQGIHSLLL
PILVTVTGENKDVSETSFQNAMLKPMCETLTYISKEQLLSHKLPARLVADQKTNLPEYLQ
TLLNTLAPLLLFRARPVQIAVYHMLYKLMPELPQYDQDNLKSYGDEEEEPALSPPAALMS
LLSIQEDLLENVLGCIPVGQIVTIKPLSEDFCYVLGYLLTWKLILTFFKAASSQLRALYS
MYLRKTKSLNKLLYHLFRLMPENPTYAETAVEVPNKDPKTFFTEELQLSIRETTMLPYHI
PHLACSVYHMTLKDLPAMVRLWWNSSEKRVFNIVDRFTSKYVSSVLSFQEISSVQTSTQL
FNGMTVKARATTREVMATYTIEDIVIELIIQLPSNYPLGSIIVESGKRVGVAVQQWRNWM
LQLSTYLTHQNGSIMEGLALWKNNVDKRFEGVEDCMICFSVIHGFNYSLPKKACRTCKKK
FHSACLYKWFTSSNKSTCPLCRETFF
>sp|Q9Y6A5|TACC3_HUMAN Transforming acidic coiled-coil-containing protein 3 OS=Homo sapiens OX=9606 GN=TACC3 PE=1 SV=1
MSLQVLNDKNVSNEKNTENCDFLFSPPEVTGRSSVLRVSQKENVPPKNLAKAMKVTFQTP
LRDPQTHRILSPSMASKLEAPFTQDDTLGLENSHPVWTQKENQQLIKEVDAKTTHGILQK
PVEADTDLLGDASPAFGSGSSSESGPGALADLDCSSSSQSPGSSENQMVSPGKVSGSPEQ
AVEENLSSYSLDRRVTPASETLEDPCRTESQHKAETPHGAEEECKAETPHGAEEECRHGG
VCAPAAVATSPPGAIPKEACGGAPLQGLPGEALGCPAGVGTPVPADGTQTLTCAHTSAPE
STAPTNHLVAGRAMTLSPQEEVAAGQMASSSRSGPVKLEFDVSDGATSKRAPPPRRLGER
SGLKPPLRKAAVRQQKAPQEVEEDDGRSGAGEDPPMPASRGSYHLDWDKMDDPNFIPFGG
DTKSGCSEAQPPESPETRLGQPAAEQLHAGPATEEPGPCLSQQLHSASAEDTPVVQLAAE
TPTAESKERALNSASTSLPTSCPGSEPVPTHQQGQPALELKEESFRDPAEVLGTGAEVDY
LEQFGTSSFKESALRKQSLYLKFDPLLRDSPGRPVPVATETSSMHGANETPSGRPREAKL
VEFDFLGALDIPVPGPPPGVPAPGGPPLSTGPIVDLLQYSQKDLDAVVKATQEENRELRS

RCEELHGKNLELGKIMDRFEEVVYQAMEEVQKQKELSKAEIQKVLKEKDQLTTDLNSMEK
SFSDLFKRFEKQKEVIEGYRKNEESLKKCVEDYLARITQEGQRYQALKAHAEEKLQLANE
EIAQVRSKAQAEALALQASLRKEQMRIQSLEKTVEQKTKENEELTRICDDLISKMEKI
>sp|Q8N3K9|CMYA5_HUMAN Cardiomyopathy-associated protein 5 OS=Homo sapiens OX=9606 GN=CMYA5 PE=1 SV=3
MASRDSNHAGESFLGSDGDEEATRELETEEESEGEEDETAAESEEEPDSRLSDQDEEGKI
KQEYIISDPSFSMVTVQREDSGITWETNSSRSSTPWASEESQTSGVCSREGSTVNSPPGN
VSFIVDEVKKVRKRTHKSKHGSPSLRRKGNRKRNSFESQDVPTNKKGSPLTSASQVLTTE
KEKSYTGIYDKARKKKTTSNTPPITGAIYKEHKPLVLRPVYIGTVQYKIKMFNSVKEELI
PLQFYGTLPKGYVIKEIHYRKGKDASISLEPDLDNSGSNTVSKTRKLVAQSIEDKVKEVF
PPWRGALSKGSESLTLMFSHEDQKKIYADSPLNATSALEHTVPSYSSSGRAEQGIQLRHS
QSVPQQPEDEAKPHEVEPPSVTPDTPATMFLRTTKEECELASPGTAASENDSSVSPSFAN
EVKKEDVYSAHHSISLEAASPGLAASTQDGLDPDQEQPDLTSIERAEPVSAKLTPTHPSV
KGEKEENMLEPSISLSEPLMLEEPEKEEIETSLPIAITPEPEDSNLVEEEIVELDYPESP
LVSEKPFPPHMSPEVEHKEEELILPLLAASSPEHVALSEEEREEIASVSTGSAFVSEYSV
PQDLNHELQEQEGEPVPPSNVEAIAEHAVLSEEENEEFEAYSPAAAPTSESSLSPSTTEK
TSENQSPLFSTVTPEYMVLSGDEASESGCYTPDSTSASEYSVPSLATKESLKKTIDRKSP
LILKGVSEYMIPSEEKEDTGSFTPAVAPASEPSLSPSTTEKTSECQSPLPSTATSEHVVP
SEGEDLGSERFTPDSKLISKYAAPLNATQESQKKIINEASQFKPKGISEHTVLSVDGKEV
IGPSSPDLVVASEHSFPPHTTEMTSECQAPPLSATPSEYVVLSDEEAVELERYTPSSTSA
SEFSVPPYATPEAQEEEIVHRSLNLKGASSPMNLSEEDQEDIGPFSPDSAFVSEFSFPPY
ATQEAEKREFECDSPICLTSPSEHTILSDEDTEEAELFSPDSASQVSIPPFRISETEKNE
LEPDSLLTAVSASGYSCFSEADEEDIGSTAATPVSEQFSSSQKQKAETFPLMSPLEDLSL
PPSTDKSEKAEIKPEIPTTSTSVSEYLILAQKQKTQAYLEPESEDLIPSHLTSEVEKGER
EASSSVAAIPAALPAQSSIVKEETKPASPHSVLPDSVPAIKKEQEPTAALTLKAADEQMA
LSKVRKEEIVPDSQEATAHVSQDQKMEPQPPNVPESEMKYSVLPDMVDEPKKGVKPKLVL
NVTSELEQRKLSKNEPEVIKPYSPLKETSLSGPEALSAVKMEMKHDSKITTTPIVLHSAS
SGVEKQVEHGPPALAFSALSEEIKKEIEPSSSTTTASVTKLDSNLTRAVKEEIPTDSSLI
TPVDRPVLTKVGKGELGSGLPPLVTSADEHSVLAEEDKVAIKGASPIETSSKHLAWSEAE
KEIKFDSLPSVSSIAEHSVLSEVEAKEVKAGLPVIKTSSSQHSDKSEEARVEDKQDLLFS
TVCDSERLVSSQKKSLMSTSEVLEPEHELPLSLWGEIKKKETELPSSQNVSPASKHIIPK
GKDEETASSSPELENLASGLAPTLLLLSDDKNKPAVEVSSTAQGDFPSEKQDVALAELSL
EPEKKDKPHQPLELPNAGSEFSSDLGRQSGSIGTKQAKSPITETEDSVLEKGPAELRSRE
GKEENRELCASSTMPAISELSSLLREESQNEEIKPFSPKIISLESKEPPASVAEGGNPEE
FQPFTFSLKGLSEEVSHPADFKKGGNQEIGPLPPTGNLKAQVMGDILDKLSEETGHPNSS
QVLQSITEPSKIAPSDLLVEQKKTEKALHSDQTVKLPDVSTSSEDKQDLGIKQFSLMREN
LPLEQSKSFMTTKPADVKETKMEEFFISPKDENWMLGKPENVASQHEQRIAGSVQLDSSS
SNELRPGQLKAAVSSKDHTCEVRKQVLPHSAEESHLSSQEAVSALDTSSGNTETLSSKSY
SSEEVKLAEEPKSLVLAGNVERNIAEGKEIHSLMESESLLLEKANTELSWPSKEDSQEKI
KLPPERFFQKPVSGLSVEQVKSETISSSVKTAHFPAEGVEPALGNEKEAHRSTPPFPEEK
PLEESKMVQSKVIDDADEGKKPSPEVKIPTQRKPISSIHAREPQSPESPEVTQNPPTQPK
VAKPDLPEEKGKKGISSFKSWMSSLFFGSSTPDNKVAEQEDLETQPSPSVEKAVTVIDPE
GTIPTNFNVAEKPADHSLSEVKLKTADEPRGTLVKSGDGQNVKEKSMILSNVEDLQQPKF
ISEVSREDYGKKEISGDSEEMNINSVVTSADGENLEIQSYSLIGEKLVMEEAKTIVPPHV
TDSKRVQKPAIAPPSKWNISIFKEEPRSDQKQKSLLSFDVVDKVPQQPKSASSNFASKNI
TKESEKPESIILPVEESKGSLIDFSEDRLKKEMQNPTSLKISEEETKLRSVSPTEKKDNL
ENRSYTLAEKKVLAEKQNSVAPLELRDSNEIGKTQITLGSRSTELKESKADAMPQHFYQN

EDYNERPKIIVGSEKEKGEEKENQVYVLSEGKKQQEHQPYSVNVAESMSRESDISLGHSL
GETQSFSLVKATSVTEKSEAMLAEAHPEIREAKAVGTQPHPLEESKVLVEKTKTFLPVAL
SCRDEIENHSLSQEGNLVLEKSSRDMPDHSEEKEQFRESELSKGGSVDITKETVKQGFQE
KAVGTQPRPLEESKVLVEKTKTFLPVVLSCHDEIENHSLSQEGNLVLEKSSRDMPDHSEE
KEQFKESELWKGGSVDITKESMKEGFPSKESERTLARPFDETKSSETPPYLLSPVKPQTL
ASGASPEINAVKKKEMPRSELTPERHTVHTIQTSKDDTSDVPKQSVLVSKHHLEAAEDTR
VKEPLSSAKSNYAQFISNTSASNADKMVSNKEMPKEPEDTYAKGEDFTVTSKPAGLSEDQ
KTAFSIISEGCEILNIHAPAFISSIDQEESEQMQDKLEYLEEKASFKTIPLPDDSETVAC
HKTLKSRLEDEKVTPLKENKQKETHKTKEEISTDSETDLSFIQPTIPSEEDYFEKYTLID
YNISPDPEKQKAPQKLNVEEKLSKEVTEETISFPVSSVESALEHEYDLVKLDESFYGPEK
GHNILSHPETQSQNSADRNVSKDTKRDVDSKSPGMPLFEAEEGVLSRTQIFPTTIKVIDP
EFLEEPPALAFLYKDLYEEAVGEKKKEEETASEGDSVNSEASFPSRNSDTDDGTGIYFEK
YILKDDILHDTSLTQKDQGQGLEEKRVGKDDSYQPIAAEGEIWGKFGTICREKSLEEQKG
VYGEGESVDHVETVGNVAMQKKAPITEDVRVATQKISYAVPFEDTHHVLERADEAGSHGN
EVGNASPEVNLNVPVQVSFPEEEFASGATHVQETSLEEPKILVPPEPSEERLRNSPVQDE
YEFTESLHNEVVPQDILSEELSSESTPEDVLSQGKESFEHISENEFASEAEQSTPAEQKE
LGSERKEEDQLSSEVVTEKAQKELKKSQIDTYCYTCKCPISATDKVFGTHKDHEVSTLDT
AISAVKVQLAEFLENLQEKSLRIEAFVSEIESFFNTIEENCSKNEKRLEEQNEEMMKKVL
AQYDEKAQSFEEVKKKKMEFLHEQMVHFLQSMDTAKDTLETIVREAEELDEAVFLTSFEE
INERLLSAMESTASLEKMPAAFSLFEHYDDSSARSDQMLKQVAVPQPPRLEPQEPNSATS
TTIAVYWSMNKEDVIDSFQVYCMEEPQDDQEVNELVEEYRLTVKESYCIFEDLEPDRCYQ
VWVMAVNFTGCSLPSERAIFRTAPSTPVIRAEDCTVCWNTATIRWRPTTPEATETYTLEY
CRQHSPEGEGLRSFSGIKGLQLKVNLQPNDNYFFYVRAINAFGTSEQSEAALISTRGTRF
LLLRETAHPALHISSSGTVISFGERRRLTEIPSVLGEELPSCGQHYWETTVTDCPAYRLG
ICSSSAVQAGALGQGETSWYMHCSEPQRYTFFYSGIVSDVHVTERPARVGILLDYNNQRL
IFINAESEQLLFIIRHRFNEGVHPAFALEKPGKCTLHLGIEPPDSVRHK
>sp|Q92608|DOCK2_HUMAN Dedicator of cytokinesis protein 2 OS=Homo sapiens OX=9606 GN=DOCK2 PE=1 SV=2
MAPWRKADKERHGVAIYNFQGSGAPQLSLQIGDVVRIQETCGDWYRGYLIKHKMLQGIFP
KSFIHIKEVTVEKRRNTENIIPAEIPLAQEVTTTLWEWGSIWKQLYVASKKERFLQVQSM
MYDLMEWRSQLLSGTLPKDELKELKQKVTSKIDYGNKILELDLIVRDEDGNILDPDNTSV
ISLFHAHEEATDKITERIKEEMSKDQPDYAMYSRISSSPTHSLYVFVRNFVCRIGEDAEL
FMSLYDPNKQTVISENYLVRWGSRGFPKEIEMLNNLKVVFTDLGNKDLNRDKIYLICQIV
RVGKMDLKDTGAKKCTQGLRRPFGVAVMDITDIIKGKAESDEEKQHFIPFHPVTAENDFL
HSLLGKVIASKGDSGGQGLWVTMKMLVGDIIQIRKDYPHLVDRTTVVARKLGFPEIIMPG
DVRNDIYITLLQGDFDKYNKTTQRNVEVIMCVCAEDGKTLPNAICVGAGDKPMNEYRSVV
YYQVKQPRWMETVKVAVPIEDMQRIHLRFMFRHRSSLESKDKGEKNFAMSYVKLMKEDGT
TLHDGFHDLVVLKGDSKKMEDASAYLTLPSYRHHVENKGATLSRSSSSVGGLSVSSRDVF
SISTLVCSTKLTQNVGLLGLLKWRMKPQLLQENLEKLKIVDGEEVVKFLQDTLDALFNIM
MEHSQSDEYDILVFDALIYIIGLIADRKFQHFNTVLEAYIQQHFSATLAYKKLMTVLKTY
LDTSSRGEQCEPILRTLKALEYVFKFIVRSRTLFSQLYEGKEQMEFEESMRRLFESINNL
MKSQYKTTILLQVAALKYIPSVLHDVEMVFDAKLLSQLLYEFYTCIPPVKLQKQKVQSMN
EIVQSNLFKKQECRDILLPVITKELKELLEQKDDMQHQVLERKYCVELLNSILEVLSYQD
AAFTYHHIQEIMVQLLRTVNRTVITMGRDHILISHFVACMTAILNQMGDQHYSFYIETFQ
TSSELVDFLMETFIMFKDLIGKNVYPGDWMAMSMVQNRVFLRAINKFAETMNQKFLEHTN
FEFQLWNNYFHLAVAFITQDSLQLEQFSHAKYNKILNKYGDMRRLIGFSIRDMWYKLGQN
KICFIPGMVGPILEMTLIPEAELRKATIPIFFDMMLCEYQRSGDFKKFENEIILKLDHEV

EGGRGDEQYMQLLESILMECAAEHPTIAKSVENFVNLVKGLLEKLLDYRGVMTDESKDNR
MSCTVNLLNFYKDNNREEMYIRYLYKLRDLHLDCDNYTEAAYTLLLHTWLLKWSDEQCAS
QVMQTGQQHPQTHRQLKETLYETIIGYFDKGKMWEEAISLCKELAEQYEMEIFDYELLSQ
NLIQQAKFYESIMKILRPKPDYFAVGYYGQGFPSFLRNKVFIYRGKEYERREDFQMQLMT
QFPNAEKMNTTSAPGDDVKNAPGQYIQCFTVQPVLDEHPRFKNKPVPDQIINFYKSNYVQ
RFHYSRPVRRGTVDPENEFASMWIERTSFVTAYKLPGILRWFEVVHMSQTTISPLENAIE
TMSTANEKILMMINQYQSDETLPINPLSMLLNGIVDPAVMGGFAKYEKAFFTEEYVRDHP
EDQDKLTHLKDLIAWQIPFLGAGIKIHEKRVSDNLRPFHDRMEECFKNLKMKVEKEYGVR
EMPDFDDRRVGRPRSMLRSYRQMSIISLASMNSDCSTPSKPTSESFDLELASPKTPRVEQ
EEPISPGSTLPEVKLRRSKKRTKRSSVVFADEKAAAESDLKRLSRKHEFMSDTNLSEHAA
IPLKASVLSQMSFASQSMPTIPALALSVAGIPGLDEANTSPRLSQTFLQLSDGDKKTLTR
KKVNQFFKTMLASKSAEEGKQIPDSLSTDL
>sp|Q8WVM8|SCFD1_HUMAN Sec1 family domain-containing protein 1 OS=Homo sapiens OX=9606 GN=SCFD1 PE=1 SV=4
MAAAAAATAAAAASIRERQTVALKRMLNFNVPHIKNSTGEPVWKVLIYDRFGQDIISPLL
SVKELRDMGITLHLLLHSDRDPIPDVPAVYFVMPTEENIDRMCQDLRNQLYESYYLNFIS
AISRSKLEDIANAALAASAVTQVAKVFDQYLNFITLEDDMFVLCNQNKELVSYRAINRPD
ITDTEMETVMDTIVDSLFCFFVTLGAVPIIRCSRGTAAEMVAVKLDKKLRENLRDARNSL
FTGDTLGAGQFSFQRPLLVLVDRNIDLATPLHHTWTYQALVHDVLDFHLNRVNLEESSGV
ENSPAGARPKRKNKKSYDLTPVDKFWQKHKGSPFPEVAESVQQELESYRAQEDEVKRLKS
IMGLEGEDEGAISMLSDNTAKLTSAVSSLPELLEKKRLIDLHTNVATAVLEHIKARKLDV
YFEYEEKIMSKTTLDKSLLDIISDPDAGTPEDKMRLFLIYYISTQQAPSEADLEQYKKAL
TDAGCNLNPLQYIKQWKAFTKMASAPASYGSTTTKPMGLLSRVMNTGSQFVMEGVKNLVL
KQQNLPVTRILDNLMEMKSNPETDDYRYFDPKMLRGNDSSVPRNKNPFQEAIVFVVGGGN
YIEYQNLVDYIKGKQGKHILYGCSELFNATQFIKQLSQLGQK
>sp|O75874|IDHC_HUMAN Isocitrate dehydrogenase [NADP] cytoplasmic OS=Homo sapiens OX=9606 GN=IDH1 PE=1 SV=2
MSKKISGGSVVEMQGDEMTRIIWELIKEKLIFPYVELDLHSYDLGIENRDATNDQVTKDA
AEAIKKHNVGVKCATITPDEKRVEEFKLKQMWKSPNGTIRNILGGTVFREAIICKNIPRL
VSGWVKPIIIGRHAYGDQYRATDFVVPGPGKVEITYTPSDGTQKVTYLVHNFEEGGGVAM
GMYNQDKSIEDFAHSSFQMALSKGWPLYLSTKNTILKKYDGRFKDIFQEIYDKQYKSQFE
AQKIWYEHRLIDDMVAQAMKSEGGFIWACKNYDGDVQSDSVAQGYGSLGMMTSVLVCPDG
KTVEAEAAHGTVTRHYRMYQKGQETSTNPIASIFAWTRGLAHRAKLDNNKELAFFANALE
EVSIETIEAGFMTKDLAACIKGLPNVQRSDYLNTFEFMDKLGENLKIKLAQAKL
>sp|Q13595|TRA2A_HUMAN Transformer-2 protein homolog alpha OS=Homo sapiens OX=9606 GN=TRA2A PE=1 SV=1
MSDVEENNFEGRESRSQSKSPTGTPARVKSESRSGSRSPSRVSKHSESHSRSRSKSRSRS
RRHSHRRYTRSRSHSHSHRRRSRSRSYTPEYRRRRSRSHSPMSNRRRHTGSRANPDPNTC
LGVFGLSLYTTERDLREVFSRYGPLSGVNVVYDQRTGRSRGFAFVYFERIDDSKEAMERA
NGMELDGRRIRVDYSITKRAHTPTPGIYMGRPTHSGGGGGGGGGGGGGGGGRRRDSYYDR
GYDRGYDRYEDYDYRYRRRSPSPYYSRYRSRSRSRSYSPRRY
>sp|Q9UKK3|PARP4_HUMAN Protein mono-ADP-ribosyltransferase PARP4 OS=Homo sapiens OX=9606 GN=PARP4 PE=1 SV=3
MVMGIFANCIFCLKVKYLPQQQKKKLQTDIKENGGKFSFSLNPQCTHIILDNADVLSQYQ
LNSIQKNHVHIANPDFIWKSIREKRLLDVKNYDPYKPLDITPPPDQKASSSEVKTEGLCP
DSATEEEDTVELTEFGMQNVEIPHLPQDFEVAKYNTLEKVGMEGGQEAVVVELQCSRDSR
DCPFLISSHFLLDDGMETRRQFAIKKTSEDASEYFENYIEELKKQGFLLREHFTPEATQL

ASEQLQALLLEEVMNSSTLSQEVSDLVEMIWAEALGHLEHMLLKPVNRISLNDVSKAEGI
LLLVKAALKNGETAEQLQKMMTEFYRLIPHKGTMPKEVNLGLLAKKADLCQLIRDMVNVC
ETNLSKPNPPSLAKYRALRCKIEHVEQNTEEFLRVRKEVLQNHHSKSPVDVLQIFRVGRV
NETTEFLSKLGNVRPLLHGSPVQNIVGILCRGLLLPKVVEDRGVQRTDVGNLGSGIYFSD
SLSTSIKYSHPGETDGTRLLLICDVALGKCMDLHEKDFSLTEAPPGYDSVHGVSQTASVT
TDFEDDEFVVYKTNQVKMKYIIKFSMPGDQIKDFHPSDHTELEEYRPEFSNFSKVEDYQL
PDAKTSSSTKAGLQDASGNLVPLEDVHIKGRIIDTVAQVIVFQTYTNKSHVPIEAKYIFP
LDDKAAVCGFEAFINGKHIVGEIKEKEEAQQEYLEAVTQGHGAYLMSQDAPDVFTVSVGN
LPPKAKVLIKITYITELSILGTVGVFFMPATVAPWQQDKALNENLQDTVEKICIKEIGTK
QSFSLTMSIEMPYVIEFIFSDTHELKQKRTDCKAVISTMEGSSLDSSGFSLHIGLSAAYL
PRMWVEKHPEKESEACMLVFQPDLDVDLPDLASESEVIICLDCSSSMEGVTFLQAKQIAL
HALSLVGEKQKVNIIQFGTGYKELFSYPKHITSNTMAAEFIMSATPTMGNTDFWKTLRYL
SLLYPARGSRNILLVSDGHLQDESLTLQLVKRSRPHTRLFACGIGSTANRHVLRILSQCG
AGVFEYFNAKSKHSWRKQIEDQMTRLCSPSCHSVSVKWQQLNPDVPEALQAPAQVPSLFL
NDRLLVYGFIPHCTQATLCALIQEKEFRTMVSTTELQKTTGTMIHKLAARALIRDYEDGI
LHENETSHEMKKQTLKSLIIKLSKENSLITQFTSFVAVEKRDENESPFPDIPKVSELIAK
EDVDFLPYMSWQGEPQEAVRNQSLLASSEWPELRLSKRKHRKIPFSKRKMELSQPEVSED
FEEDGLGVLPAFTSNLERGGVEKLLDLSWTESCKPTATEPLFKKVSPWETSTSSFFPILA
PAVGSYLPPTARAHSPASLSFASYRQVASFGSAAPPRQFDASQFSQGPVPGTCADWIPQS
ASCPTGPPQNPPSSPYCGIVFSGSSLSSAQSAPLQHPGGFTTRPSAGTFPELDSPQLHFS
LPTDPDPIRGFGSYHPSASSPFHFQPSAASLTANLRLPMASALPEALCSQSRTTPVDLCL
LEESVGSLEGSRCPVFAFQSSDTESDELSEVLQDSCFLQIKCDTKDDSILCFLEVKEEDE
IVCIQHWQDAVPWTELLSLQTEDGFWKLTPELGLILNLNTNGLHSFLKQKGIQSLGVKGR
ECLLDLIATMLVLQFIRTRLEKEGIVFKSLMKMDDASISRNIPWAFEAIKQASEWVRRTE
GQYPSICPRLELGNDWDSATKQLLGLQPISTVSPLHRVLHYSQG
>sp|O60229|KALRN_HUMAN Kalirin OS=Homo sapiens OX=9606 GN=KALRN
PE=1 SV=3
MTDRFWDQWYLWYLRLLRLLDRGSFRNDGLKASDVLPILKEKVAFVSGGRDKRGGPILTF
PARSNHDRIRQEDLRKLVTYLASVPSEDVCKRGFTVIIDMRGSKWDLIKPLLKTLQEAFP
AEIHVALIIKPDNFWQKQKTNFGSSKFIFETSMVSVEGLTKLVDPSQLTEEFDGSLDYNH
EEWIELRLSLEEFFNSAVHLLSRLEDLQEMLARKEFPVDVEGSRRLIDEHTQLKKKVLKA
PVEELDREGQRLLQCIRCSDGFSGRNCIPGSADFQSLVPKITSLLDKLHSTRQHLHQMWH
VRKLKLDQCFQLRLFEQDAEKMFDWISHNKELFLQSHTEIGVSYQYALDLQTQHNHFAMN
SMNAYVNINRIMSVASRLSEAGHYASQQIKQISTQLDQEWKSFAAALDERSTILAMSAVF
HQKAEQFLSGVDAWCKMCSEGGLPSEMQDLELAIHHHQTLYEQVTQAYTEVSQDGKALLD
VLQRPLSPGNSESLTATANYSKAVHQVLDVVHEVLHHQRRLESIWQHRKVRLHQRLQLCV
FQQDVQQVLDWIENHGEAFLSKHTGVGKSLHRARALQKRHDDFEEVAQNTYTNADKLLEA
AEQLAQTGECDPEEIYKAARHLEVRIQDFVRRVEQRKLLLDMSVSFHTHTKELWTWMEDL
QKEMLEDVCADSVDAVQELIKQFQQQTATLDATLNVIKEGEDLIQQLRSAPPSLGEPSE
ARDSAVSNNKTPHSSSISHIESVLQQLDDAQVQMEELFHERKIKLDIFLQLRIFEQYTIE
VTAELDAWNEDLLRQMNDFNTEDLTLAEQRLQRHTERKLAMNNMTFEVIQQGQDLHQYIT
EVQASGIELICEKDIDLAAQVQELLEFLHEKQHELELNAEQTHKRLEQCLQLRHLQAEVK
QVLGWIRNGESMLNASLVNASSLSEAEQLQREHEQFQLAIESLFHATSLQKTHQSALQVQ
QKAEVLLQAGHYDADAIRECAEKVALHWQQLMLKMEDRLKLVNASVAFYKTSEQVCSVLE
SLEQEYRRDEDWCGGRDKLGPAAEIDHVIPLISKHLEQKEAFLKACTLARRNAEVFLKYI
HRNNVSMPSVASHTRGPEQQVKAILSELLQRENRVLHFWTLKKRRLDQCQQYVVFERSAK
QALDWIQETGEFYLSTHTSTGETTEETQELLKEYGEFRVPAKQTKEKVKLLIQLADSFVE

KGHIHATEIRKWVTTVDKHYRDFSLRMGKYRYSLEKALGVNTEDNKDLELDIIPASLSDR
EVKLRDANHEVNEEKRKSARKKEFIMAELLQTEKAYVRDLHECLETYLWEMTSGVEEIPP
GILNKEHIIFGNIQEIYDFHNNIFLKELEKYEQLPEDVGHCFVTWADKFQMYVTYCKNKP
DSNQLILEHAGTFFDEIQQRHGLANSISSYLIKPVQRITKYQLLLKELLTCCEEGKGELK
DGLEVMLSVPKKANDAMHVSMLEGFDENLDVQGELILQDAFQVWDPKSLIRKGRERHLFL
FEISLVFSKEIKDSSGHTKYVYKNKLLTSELGVTEHVEGDPCKFALWSGRTPSSDNKTVL
KASNIETKQEWIKNIREVIQERIIHLKGALKEPLQLPKTPAKQRNNSKRDGVEDIDSQGD
GSSQPDTISIASRTSQNTVDSDKLSGGCELTVVLQDFSAGHSSELTIQVGQTVELLERPS
ERPGWCLVRTTERSPPLEGLVPSSALCISHSRSSVEMDCFFPLVKDAYSHSSSENGGKSE
SVANLQAQPSLNSIHSSPGPKRSTNTLKKWLTSPVRRLNSGKADGNIKKQKKVRDGRKSF
DLGSPKPGDETTPQGDSADEKSKKGWGEDEPDEESHTPLPPPMKIFDNDPTQDEMSSSLL
AARQASTEVPTAADLVNAIEKLVKNKLSLEGSSYRGSLKDPAGCLNEGMAPPTPPKNPEE
EQKAKALRGRMFVLNELVQTEKDYVKDLGIVVEGFMKRIEEKGVPEDMRGKDKIVFGNIH
QIYDWHKDFFLAELEKCIQEQDRLAQLFIKHERKLHIYVWYCQNKPRSEYIVAEYDAYFE
EVKQEINQRLTLSDFLIKPIQRITKYQLLLKDFLRYSEKAGLECSDIEKAVELMCLVPKR
CNDMMNLGRLQGFEGTLTAQGKLLQQDTFYVIELDAGMQSRTKERRVFLFEQIVIFSELL
RKGSLTPGYMFKRSIKMNYLVLEENVDNDPCKFALMNRETSERVVLQAANADIQQAWVQD
INQVLETQRDFLNALQSPIEYQRKERSTAVMRSQPARLPQASPRPYSSVPAGSEKPPKGS
SYNPPLPPLKISTSNGSPGFEYHQPGDKFEASKQNDLGGCNGTSSMAVIKDYYALKENEI
CVSQGEVVQVLAVNQQNMCLVYQPASDHSPAAEGWVPGSILAPLTKATAAESSDGSIKKS
CSWHTLRMRKRAEVENTGKNEATGPRKPKDILGNKVSVKETNSSEESECDDLDPNTSMEI
LNPNFIQEVAPEFLVPLVDVTCLLGDTVILQCKVCGRPKPTITWKGPDQNILDTDNSSAT
YTVSSCDSGEITLKICNLMPQDSGIYTCIATNDHGTTSTSATVKVQGVPAAPNRPIAQER
SCTSVILRWLPPSSTGNCTISGYTVEYREEGSQIWQQSVASTLDTYLVIEDLSPGCPYQF
RVSASNPWGISLPSEPSEFVRLPEYDAAADGATISWKENFDSAYTELNEIGRGRFSIVKK
CIHKATRKDVAVKFVSKKMKKKEQAAHEAALLQHLQHPQYITLHDTYESPTSYILILELM
DDGRLLDYLMNHDELMEEKVAFYIRDIMEALQYLHNCRVAHLDIKPENLLIDLRIPVPRV
KLIDLEDAVQISGHFHIHHLLGNPEFAAPEVIQGIPVSLGTDIWSIGVLTYVMLSGVSPF
LDESKEETCINVCRVDFSFPHEYFCGVSNAARDFINVILQEDFRRRPTAATCLQHPWLQP
HNGSYSKIPLDTSRLACFIERRKHQNDVRPIPNVKSYIVNRVNQGT
>sp|Q9H307|PININ_HUMAN Pinin OS=Homo sapiens OX=9606 GN=PNN PE=1 SV=5
MAVAVRTLQEQLEKAKESLKNVDENIRKLTGRDPNDVRPIQARLLALSGPGGGRGRGSLL
LRRGFSDSGGGPPAKQRDLEGAVSRLGGERRTRRESRQESDPEDDDVKKPALQSSVVATS
KERTRRDLIQDQNMDEKGKQRNRRIFGLLMGTLQKFKQESTVATERQKRRQEIEQKLEVQ
AEEERKQVENERRELFEERRAKQTELRLLEQKVELAQLQEEWNEHNAKIIKYIRTKTKPH
LFYIPGRMCPATQKLIEESQRKMNALFEGRRIEFAEQINKMEARPRRQSMKEKEHQVVRN
EEQKAEQEEGKVAQREEELEETGNQHNDVEIEEAGEEEEKEIAIVHSDAEKEQEEEEQKQ
EMEVKMEEETEVRESEKQQDSQPEEVMDVLEMVENVKHVIADQEVMETNRVESVEPSENE
ASKELEPEMEFEIEPDKECKTLSPGKENVSALDMEKESEEKEEKESEPQPEPVAQPQPQS
QPQLQLQSQSQPVLQSQPPSQPEDLSLAVLQPTPQVTQEQGHLLPERKDFPVESVKLTEV
PVEPVLTVHPESKSKTKTRSRSRGRARNKTSKSRSRSSSSSSSSSSSTSSSSGSSSSSGS
SSSRSSSSSSSSTSGSSSRDSSSSTSSSSESRSRSRGRGHNRDRKHRRSVDRKRRDTSGL
ERSHKSSKGGSSRDTKGSKDKNSRSDRKRSISESSRSGKRSSRSERDRKSDRKDKRR
